# Role of inflammatory signaling pathways involving the CD40–CD40L–TRAF cascade in diabetes and hypertension—insights from animal and human studies

**DOI:** 10.1007/s00395-024-01045-1

**Published:** 2024-03-30

**Authors:** Lea Strohm, Andreas Daiber, Henning Ubbens, Roopesh Krishnankutty, Matthias Oelze, Marin Kuntic, Omar Hahad, Veronique Klein, Imo E. Hoefer, Alex von Kriegsheim, Hartmut Kleinert, Dorothee Atzler, Philipp Lurz, Christian Weber, Philipp S. Wild, Thomas Münzel, Christoph Knosalla, Esther Lutgens, Steffen Daub

**Affiliations:** 1 Department of Cardiology, Cardiology I, University Medical Center of the Johannes Gutenberg-University, Mainz, Germany; 2 German Center for Cardiovascular Research (DZHK), Partnersite Rhine-Main, Mainz, Germany; 3 Institute of Genetics and Cancer, University of Edinburgh, Edinburgh, UK; 4 Central Diagnostic Laboratory, UMC Utrecht, Utrecht, The Netherlands; 5 Department of Pharmacology, University Medical Center of the Johannes Gutenberg-University, Mainz, Germany; 6 Institute for Cardiovascular Prevention (IPEK), Ludwig-Maximilians Universität, Munich, Germany; 7 German Center for Cardiovascular Research (DZHK), Partner Site Munich Heart Alliance, Munich, Germany; 8 Walther Straub Institute of Pharmacology and Toxicology, LMU Munich, Munich, Germany; 9 Munich Cluster for Systems Neurology (SyNergy), Munich, Germany; 10 Department of Biochemistry, Cardiovascular Research Institute Maastricht (CARIM), Maastricht University, Maastricht, The Netherlands; 11 Preventive Cardiology and Preventive Medicine, Department of Cardiology, University Medical Mainz, Johannes Gutenberg-University Mainz, Mainz, Germany; 12 Department of Cardiothoracic and Vascular Surgery, Deutsches Herzzentrum der Charité, Berlin, Germany; 13 Charité-Universitätsmedizin Berlin, Corporate Member of Freie Universität Berlin and Humboldt-Universität zu Berlin, Berlin, Germany; 14 German Center for Cardiovascular Research (DZHK), Partner Site Berlin, Berlin, Germany; 15 Department Cardiovascular Medicine and Immunology, Mayo Clinic, Rochester, MN USA; 16 Universitätsmedizin der Johannes Gutenberg-Universität Zentrum für Kardiologie 1, Labor für Molekulare Kardiologie, Geb. 605, Raum 3.262, Langenbeckstr. 1, 55131 Mainz, Germany; 17 Clinical Epidemiology and Systems Medicine, Center for Thrombosis and Hemostasis, University Medical Center Mainz, Johannes Gutenberg University Mainz, Mainz, Germany; 18 German Center for Cardiovascular Research (DZHK), Partnersite Rhine-Main, University Medical Center Mainz, Johannes Gutenberg University Mainz, Mainz, Germany; 19 Systems Medicine, Institute of Molecular Biology (IMB), Mainz, Germany

**Keywords:** Coronary heart disease, Comorbidities, Diabetes, Hypertension, Inflammation, CD40L–CD40–TRAF6 axis, Oxidative stress

## Abstract

CD40L–CD40–TRAF signaling plays a role in atherosclerosis progression and affects the pathogenesis of coronary heart disease (CHD). We tested the hypothesis that CD40L–CD40–TRAF signaling is a potential therapeutic target in hyperlipidemia, diabetes, and hypertension. In mouse models of hyperlipidemia plus diabetes (db/db mice) or hypertension (1 mg/kg/d angiotensin-II for 7 days), TRAF6 inhibitor treatment (2.5 mg/kg/d for 7 or 14 days) normalized markers of oxidative stress and inflammation. As diabetes and hypertension are important comorbidities aggravating CHD, we explored whether the CD40L–CD40–TRAF signaling cascade and their associated inflammatory pathways are expressed in CHD patients suffering from comorbidities. Therefore, we analyzed vascular bypass material (aorta or internal mammary artery) and plasma from patients with CHD with diabetes and/or hypertension. Our Olink targeted plasma proteomic analysis using the IMMUNO-ONCOLOGY panel revealed a pattern of step-wise increase for 13/92 markers of low-grade inflammation with significant changes. CD40L or CD40 significantly correlated with 38 or 56 other inflammatory targets. In addition, specific gene clusters that correlate with the comorbidities were identified in isolated aortic mRNA of CHD patients through RNA-sequencing. These signaling clusters comprised CD40L–CD40–TRAF, immune system, hemostasis, muscle contraction, metabolism of lipids, developmental biology, and apoptosis. Finally, immunological analysis revealed key markers correlated with comorbidities in CHD patients, such as CD40L, NOX2, CD68, and 3-nitrotyrosine. These data indicate that comorbidities increase inflammatory pathways in CHD, and targeting these pathways will be beneficial in reducing cardiovascular events in CHD patients with comorbidities.

**Supplementary Information:**

The online version contains supplementary material available at 10.1007/s00395-024-01045-1.

## Introduction

Cardiovascular diseases (CVD), notably ischemic heart disease and heart failure, are the leading causes of disease burden and the primary causes of death worldwide [12]. CVD patients often have comorbidities, including type 2 diabetes mellitus (T2DM), obesity, and hypertension, which increase the prevalence of CVD and mitigate the benefits of effective cardiovascular interventions [9]. In a large-scale population study containing 55,099,280 patients, a strong association of hypertension with myocardial infarction (MI) with an odds ratio of 3.11 (95% CI 3.05–3.17) was shown. Also, T2DM increases the risk of MI with an odds ratio of 1.89 (95% CI 1.86–1.91) [16]. These comorbidities result in increased myocardial oxidative stress, mainly from increased activity of nicotinamide adenine dinucleotide phosphate (NADPH) oxidases, uncoupled endothelial nitric oxide synthase (eNOS), mitochondria, as well as downregulation of antioxidant defense systems [2]. Moreover, hypertension [18, 43] and T2DM [4, 46] trigger inflammation, an independent cardiovascular risk factor [22, 45]. Inflammation and oxidative stress are known to enhance each other in a cross-talk fashion [11, 44] and prevention of excessive reactive oxygen species is a hallmark of most cardioprotective therapies [21].

The CD40L–CD40 co-stimulatory dyad is an important driver of cardiovascular disease. Inhibition of CD40 or its ligand CD40L reduces the extent of atherosclerosis and induces plaque stability in hyperlipidemic mice by reducing major inflammatory pathways. Likewise, genetic deletion of CD40L prevents cardiovascular oxidative stress and inflammation as well as endothelial dysfunction in hypertensive and diabetic/obese mice [19, 38]. Targeting downstream CD40 signaling molecules has been proven beneficial and safe to treat CVD [10, 25, 27]. Treatment with a small molecule inhibitor that targets CD40-TRAF6 signaling can reduce atherosclerosis without causing immune suppression [37]. Incorporation of this small molecule inhibitor into high-density lipoprotein (HDL) nanoparticles allowed targeted delivery in macrophages, induced plaque stabilization, and was proven safe in non-human primates [25].

The present study investigated whether targeting CD40-TRAF6 signaling is a potential therapeutic target in diabetic and hypertensive mice. We also provide evidence that the CD40L–CD40–TRAF6 cascade is involved in the network of low-grade inflammation in coronary heart disease (CHD) patients with comorbidities such as hypertension and diabetes. Whether targeting the CD40L–CD40–TRAF6 axis can ameliorate comorbidity-associated aggravation of CVD in humans remains to be established.

## Materials and methods

### Animals and treatment

Animal treatment was under the Guide for the Care and Use of Laboratory Animals as adopted and promulgated by the US National Institutes of Health and was approved by the Ethics Commission according to the German Law on the Protection of Animals (Landesuntersuchungsamt Rheinland-Pfalz, Koblenz, Germany; No.: G19-1–068 and extension from March 2022 for hypertensive (ATII) mice; No.: 23 177–07/G 11–1-020 and extension from June 2016 for diabetic (db/db) mice).

Male db/db mice (BKS.Cg-Dock7^m^ + / + Lepr^db^/J) and respective control mice (BKS.CgTemoin) at the age of 12 weeks from Charles River (Sulzfeld, Germany) were treated for two weeks with a TRAF6 inhibitor (compound 6877002, Tocris, Bristol, UK) at a dose of 10 µmol/kg/d (2.5 mg/kg/d) in 25% DMSO by osmotic minipumps (Alzet, model 1002). Here we use as terminology for the diabetic animals “db/db” and for the control mice the term “WT”. The pumps were implanted *s.c.* under ketamine/xylazine anesthesia (1 ml/kg of a ready-to-use solution containing 80 mg/ml ketamine and 12 mg/ml xylazine, Sigma-Aldrich) [38]. The mice were sacrificed after the treatment by exsanguination under isoflurane anesthesia, and the blood, kidney, and heart were collected for analysis. In total, 18 mice per group were used.

In another study, male B6 mice (C57BL/6) from Charles River (Sulzfeld Germany) at the age of 9–10 weeks were treated with either angiotensin II (ATII) at a dose of 1 mg/kg/d in 0,9% NaCl as described previously [19], TRAF6 inhibitor (compound 6877002, Tocris, Bristol, UK) at a dose of 2,5 mg/kg/d in 25% DMSO or both of it by osmotic minipumps (Alzet model 1007D). TRAF6 inhibitor and ATII are administered in two separate pumps because of the different solubility of the two drugs. The pumps were implanted *s.c.* under ketamine/xylazine anesthesia (120 mg/kg; 16 mg/kg body weight; injected fluid volume 0.1–0.2 ml). The mice were killed under ketamine/xylazine anesthesia by opening the thorax and rapid exsanguination by heart puncture. In total, six mice per group were used.

### Human samples

All human studies were following the Declaration of Helsinki. Human aortic, internal mammary artery (IMA), and plasma samples were provided from the DZHK Heart Bank at the “Deutsches Herzzentrum der Charité” in Berlin under ethical permission Numbers EA4/035/18 and EA4/032/23. They are fully characterized regarding clinical chemistry parameters (e.g., blood cholesterol, triglycerides, glucose, and creatinine) and clinical functional, lifestyle and physiological parameters (e.g., blood pressure, heart rate, smoking status, and body mass index) and disease state (e.g., multi-vessel disease, acute coronary syndrome). For patient characteristics, see Table 1. Patients eligible for elective coronary artery bypass graft (CABG) were enrolled in the study after giving informed consent to their samples being stored in the biobank. With two exceptions, all patients had multivessel disease with the two exceptions being concomitant surgical aortic valve replacement and coronary arterial dissection, respectively. As far as available, there was no significant difference in the prevalence of chronic coronary syndrome, acute coronary syndrome, non-ST-segment elevation myocardial infarction, and angina pectoris among the different patient groups.

**Table 1:** Patient characteristics

	CHD (*n* = 20)	CHD + HT (*n* = 49)	CHD + HT + T2DM (*n* = 25)	*p *value
General parameters				
Age [y] (SD)	64 (10)	58 (6)	60 (5)	0.001
Sex (male)	70% (14/20)	84% (41/49)	72% (18/25)	0.34
Current Smoker	40% (8/20)	45% (21/47)	38% (9/24)	0.891
Ex-smoker	20% (4/20)	17% (8/47)	29% (7/24)	0.489
Physiological parameters				
Height [cm] (SD)	173 (8)	176 (8)	171 (9)	0.104
BMI [kg/m^2^] (SD)	25 (4)	28 (3)	31 (4)	< 0.001
SBP [mmHg] (SD)	125 (15)	136 (26)	143 (17)	0.02
DBP [mmHg] (SD)	70 (7)	75 (13)	75 (10)	0.155
Heart rate [bpm] (SD)	73 (13)	68 (11)	74 (11)	0.107
CHD				
Multivessel disease	100% (20/20)	96% (47/49)	100% (25/25)	0.391
LMCA affected	40% (8/20)	31% (15/49)	36% (9/25)	0.735
Comorbidities				
HT	0% (0/20)	100% (49/49)	100% (25/25)	
T2DM	0% (0/20)	0% (0/49)	100% (25/25)	
Laboratory parameters				
Cholesterol [mg/dl]	190 (9/20)	158 (28/49)	173 (15/25)	0.284
Creatinine [mg/dl]	0.91 (20/20)	0.99 (49/49)	1.09 (25/25)	0.038
CRP [mg/dl]	1.32(18/20)	1.4 (38/49)	0.48 (22/25)	0.269
Glucose [mg/dl]	113 (20/20)	111 (49/49)	246 (25/25)	< 0.001
HDL [mg/dl]	40 (9/20)	36 (28/49)	36 (15/25)	0.784
LDL cholesterol [mg/dl]	124 (9/20)	95 (28/49)	103 (15/25)	0.247
Triglycerides [mg/dl]	169 (9/20))	143 (28/49)	209 (14/25)	0.082
Medication				
ASS	65% (13/20)	51% (25/49)	76% (19/25)	0.104
ACEi/ARB	50% (10/20)	88% (43/49)	80% (20/25)	0.003
BB	65% (13/20)	84% (41/49)	76% (19/25)	0.234
Nitrates	15% (3/20)	20% (10/49)	28% (7/25)	0.558
CCB	0% (0/20)	14% (7/49)	36% (9/25)	0.005
MRA	20% (4/20)	29% (14/49)	16% (4/25)	0.444
Statin	75% (15/20)	78% (38/49)	84% (21/25)	0.733
Sulfonylureas	0% (0/20)	0% (0/49)	12% (3/25)	0.014
Thiazide/distal loop	10% (2/20)	14% (7/49)	24% (6/25)	0.399
Loop diuretic	15% (3/20)	22% (11/49)	16% (4/25)	0.695
RA insulin	0% (0/20)	0% (0/49)	60% (15/25)	< 0.001
LA insulin	0% (0/20)	0% (0/49)	48% (12/25)	< 0.001
Metformin	0% (0/20)	0% (0/49)	36% (9/25)	< 0.001
SGLT2i	0% (0/22)	0% (0/49)	4% (1/25)	0.248
DPP4i	0% (0/20)	0% (0/49)	12% (3/25)	0.014
GLP1i	0% (0/20)	0% (0/49)	8% (2/25)	0.06

*BMI* body mass index, *SBP* systolic blood pressure, *DBP* diastolic blood pressure, *CHD* coronary heart disease, *LMCA* left main coronary artery, *HT* arterial hypertension, *T2DM* type 2 diabetes, *LDL* low density lipoprotein, *ASS* acetylsalicylic acid, *ACEi* angiotensin-converting enzyme inhibitor, *ARB* angiotensin receptor blocker, *BB* beta blockers, *CCB* calcium channel blocker, *MRA* mineralocorticoid receptor antagonist, *RA Insulin* rapid-acting insulin, *LA insulin* long-acting insulin, *SGLT2i* sodium-glucose cotransporter-2 inhibitors, *DPP4i* dipeptidyl peptidase-4 inhibitors, *GLP1* glucagon-like peptide-1 antagonist

### Protein and gene expression analysis, proteomics, RNA sequencing, and bioinformatical analysis

These methods are described in full detail in the online supplemental data file. For RNA sequencing data, canonical pathway analyses to envisage the biochemical processes that are changed by the two comorbidities arterial hypertension and diabetes in CHD patients were performed with IPA as shown in Suppl. Figs. S7 and S8.

### Statistical analysis

Immunoblotting, Olink, and qRT-PCR Data are shown as mean with ± SD. The normality test One-way ANOVA (with Tukey’s correction for multiple comparisons) was used. If the normality test failed, the Kruskal–Wallis test (with Dunn’s correction for multiple comparisons) was performed. Outliers were identified and removed with the ROUT (Q = 1%) method. *P* values > 0.05 were considered statistically significant. *p ≤ 0.05, **p ≤ 0.01, ***p ≤ 0.001 and **** *p* ≤ 0.0001. For statistical analysis, GraphPad Prism 9.4.1 was utilized.

## Results

### TRAF6 inhibition in diabetic (db/db) mice reduces expression of inflammatory, apoptotic, and oxidative stress markers

**Fig. 1:**
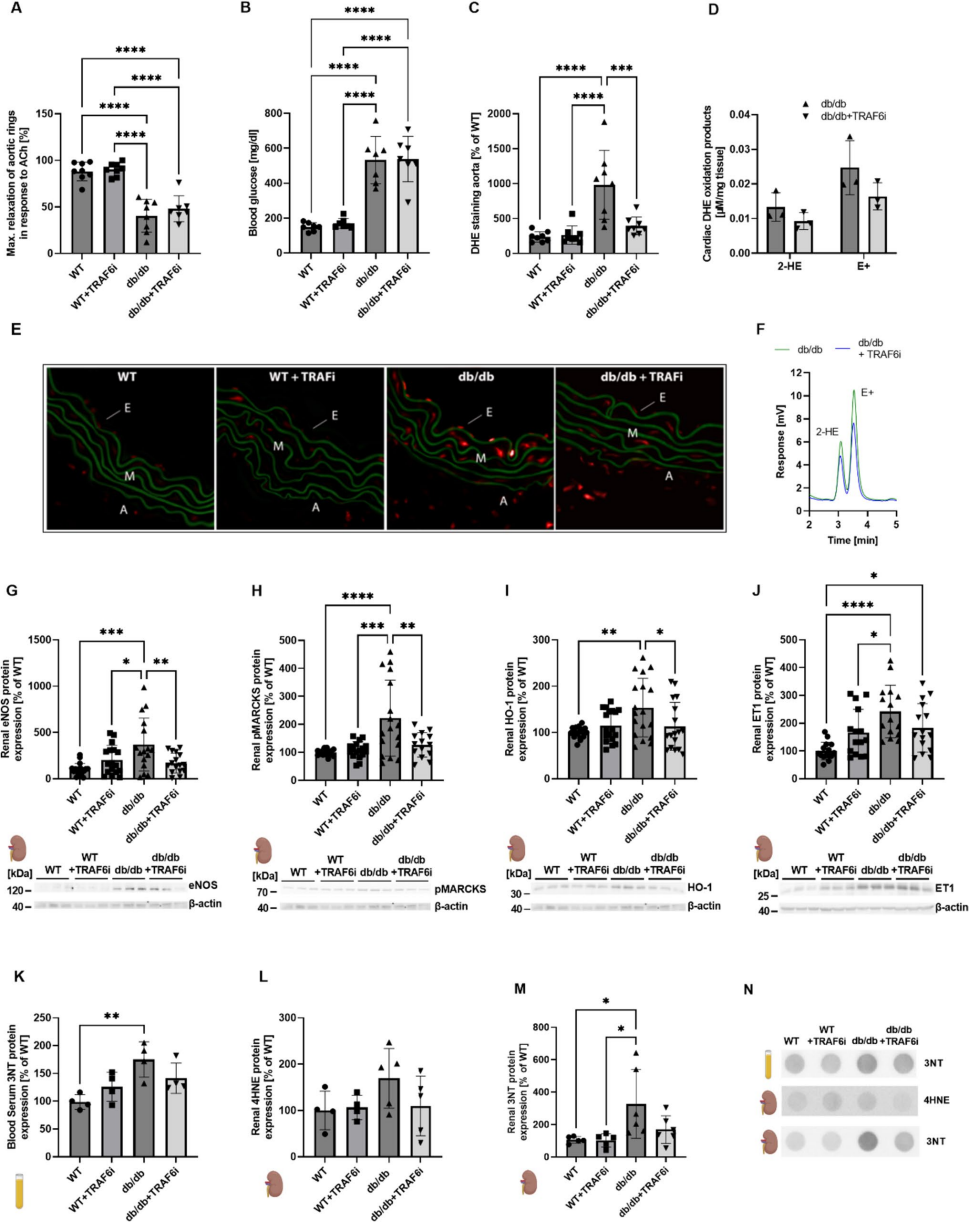
TRAF6 inhibition in diabetic (db/db) mice leads to decreased renal and sera protein expression of representative markers for inflammation and oxidative stress. Endothelial function was measured by isometric tension studies (data reproduced from published work [38]) (**A**), and glucose levels were determined in whole blood samples without previous fasting (**B**). Aortic ROS levels were analyzed in cryo-sections by DHE staining (**C**). Representative pictures are shown in (**E**). Superoxide anion levels (2-hydroxyethidium, 2-HE) and unspecific ROS formation (ethidium, E +) in cardiac tissue were measured via HPLC analysis, and corresponding representative chromatograms are shown (**D**, **F**). Renal protein expression of eNOS (**G**), pMARCKS (**H**), HO-1 (**I**), and ET-1 (**J**) was determined by immunoblotting. The densitometric quantifications are shown together with the original representative blots below. In addition, Dot Blot analysis was performed to determine the serum content of 3NT-positive proteins (**K**) and the renal levels of 4HNE- (L) and 3NT-positive proteins (**M**). Representative original blots that belong to the quantification of (**K**, **M**) are shown in (**N**). Data are mean ± SD of *n* = 7–8 (**A**–**C**), *n* = 3 (**D**), *n* = 18 (**G**–**I**), *n* = 15 (**J**), *n* = 4 (**K**, **L**), and *n* = 5 (**M**) animals per group. *p ≤ 0.05, **p ≤ 0.01, ***p ≤ 0.001 and ****p ≤ 0.0001. *WT* wild type (BKS.CgTemoin mice), *TRAF6i* TRAF6 inhibitor, *ACh* acetylcholine, *DHE* dihydroethidium, *2-HE* 2-hydroxy-ethidium, *E+*  ethidium, *E* endothel, *M* media, *A* adventitia, *ROS* reactive oxygen species, *eNOS* endothelial nitric oxide synthase, *pMARCKS* myristolated alanine-rich C kinase substrate, *HO-1* heme oxygenase 1, *ET-1* endothelin1, *3NT* 3-nitrotyrosine, *4HNE* 4-hydroxynonenal

**Fig. 2:**
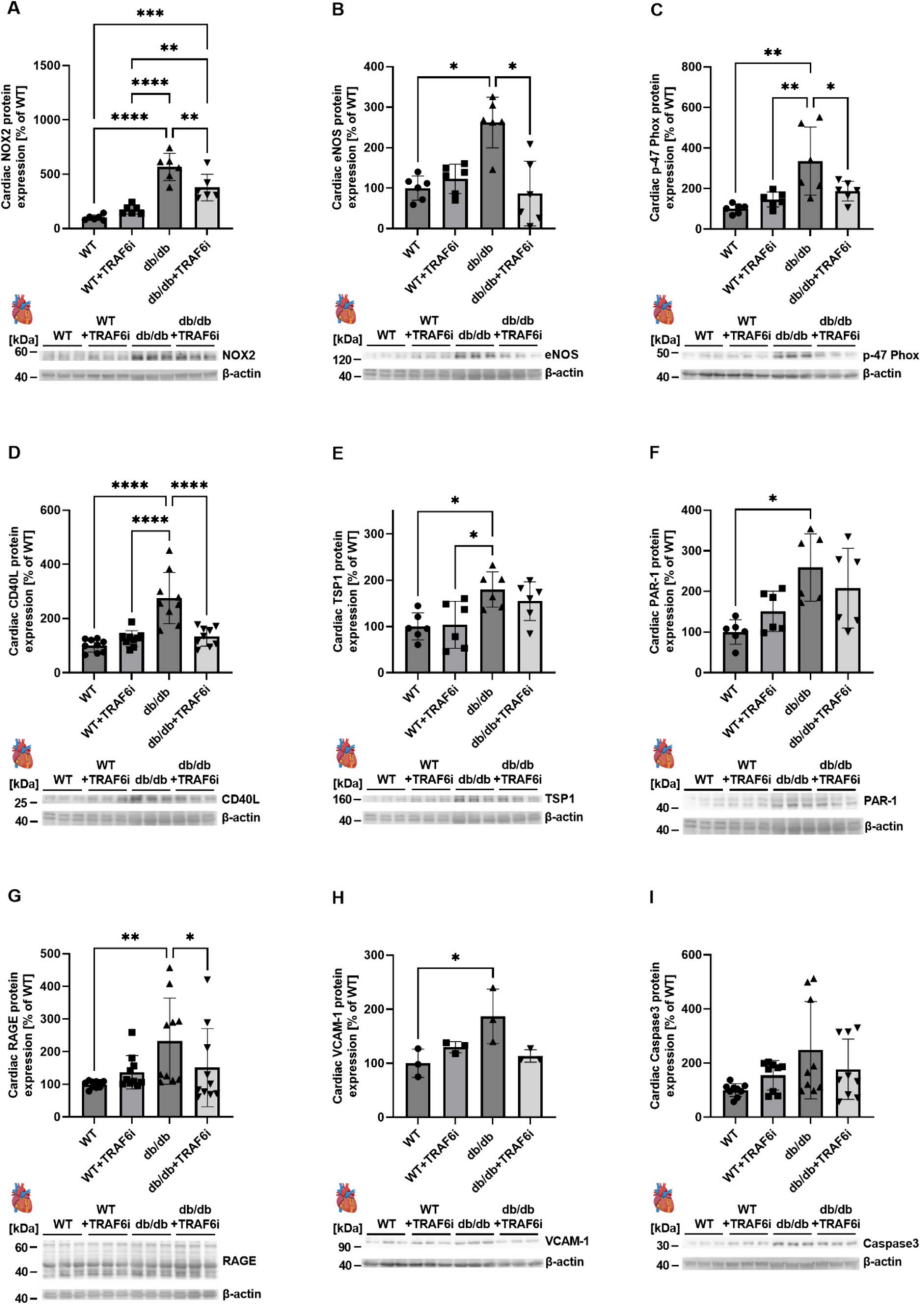
TRAF6 inhibition in diabetic (db/db) mice leads to decreased cardiac protein expression of representative markers for inflammation, apoptosis, and oxidative stress. Cardiac protein expression of NOX2 (**A**), eNOS (**B**), p47phox (**C**), CD40L (**D**), TSP1 (**E**), PAR-1 (**F**), RAGE (**G**), VCAM-1 (**H**) and Caspase3 (**I**) was determined by immunoblotting. Below the densitometric quantification, original representative blots are shown. Untreated control mice (BKS.CgTemoin) and TRAF6 inhibitor-treated mice were used as controls. Data are mean ± SD of *n* = 6 (**A**–**C**), *n* = 9 (D), *n* = 6 (**E**, **F**), *n* = 10 (G), *n* = 3 (H), and *n* = 9 (**I**) animals per group. *p ≤ 0.05, **p ≤ 0.01, ***p ≤ 0.001 and ****p ≤ 0.0001. *WT* wild type (BKS.CgTemoin mice), *TRAF6i* tumor necrosis factor receptor-associated factor 6 inhibitor, *TSP1* throbospondin-1, *eNOS* endothelial nitric oxide synthase, p47phox (NCF1) neutrophil cytosol factor 1, *NOX2* NADPH oxidase 2, *PAR-1* protease activated receptor 1, *RAGE* receptor for advanced glycosylation end products, *VCAM-1* vascular cell adhesion molecule 1

Db/db mice were used to model T2DM and obesity. The mice were treated with TRAF6 inhibitor compound 6877002 (referring to inhibition of the “CD40-TRAF6” signaling pathway) to attenuate their inflammatory phenotype, as described before [38]. Significantly reduced endothelial function (Fig. 1A) and significantly increased blood glucose levels (Fig. 1B) were observed in db/db mice compared to WT animals without significant improvement by TRAF6 inhibitor treatment. The overall ROS production in aortic tissue was measured by DHE staining, which showed a significant increase in diabetic mice and suppressed ROS formation by TRAF6 inhibition (Fig. 1C, E). Superoxide anion levels were measured in cardiac tissue by HPLC analysis. In diabetic mice, TRAF6 inhibition resulted in somewhat reduced superoxide anion levels (Fig. 1D, F). Cardiac protein expression of inflammatory, apoptotic, and oxidative stress markers was measured by immunoblotting and significantly increased in db/db mice compared to WT control mice for most of the markers. Renal protein expression of eNOS, pMARCKS, HO-1, and ET-1 (Fig. 1G–J) was significantly decreased in db/db mice after TRAF6 inhibitor treatment. Serum and renal levels of oxidative stress markers like 3NT and 4HNE were somewhat reduced after TRAF6 inhibition (Fig. 1K–N). In db/db mice, cardiac protein levels of NOX2, eNOS, p47phox, CD40L, and RAGE (Fig. 2A–D, G) were significantly decreased after TRAF6 inhibition. The protein expression of inflammatory and apoptotic markers like Tsp1, Par1, VCAM-1, and caspase3 (Fig. 2E, F, H, I) were not significantly decreased in db/db mice treated with TRAF6 inhibitor, although a trend was noticed for VCAM-1 (*p* = 0.0612). TRAF6 inhibitor treatment in WT mice did not significantly affect the expression levels of the utilized marker proteins.

### TRAF6 inhibition in hypertensive mice decreases expression of inflammatory and oxidative stress markers

**Fig. 3:**
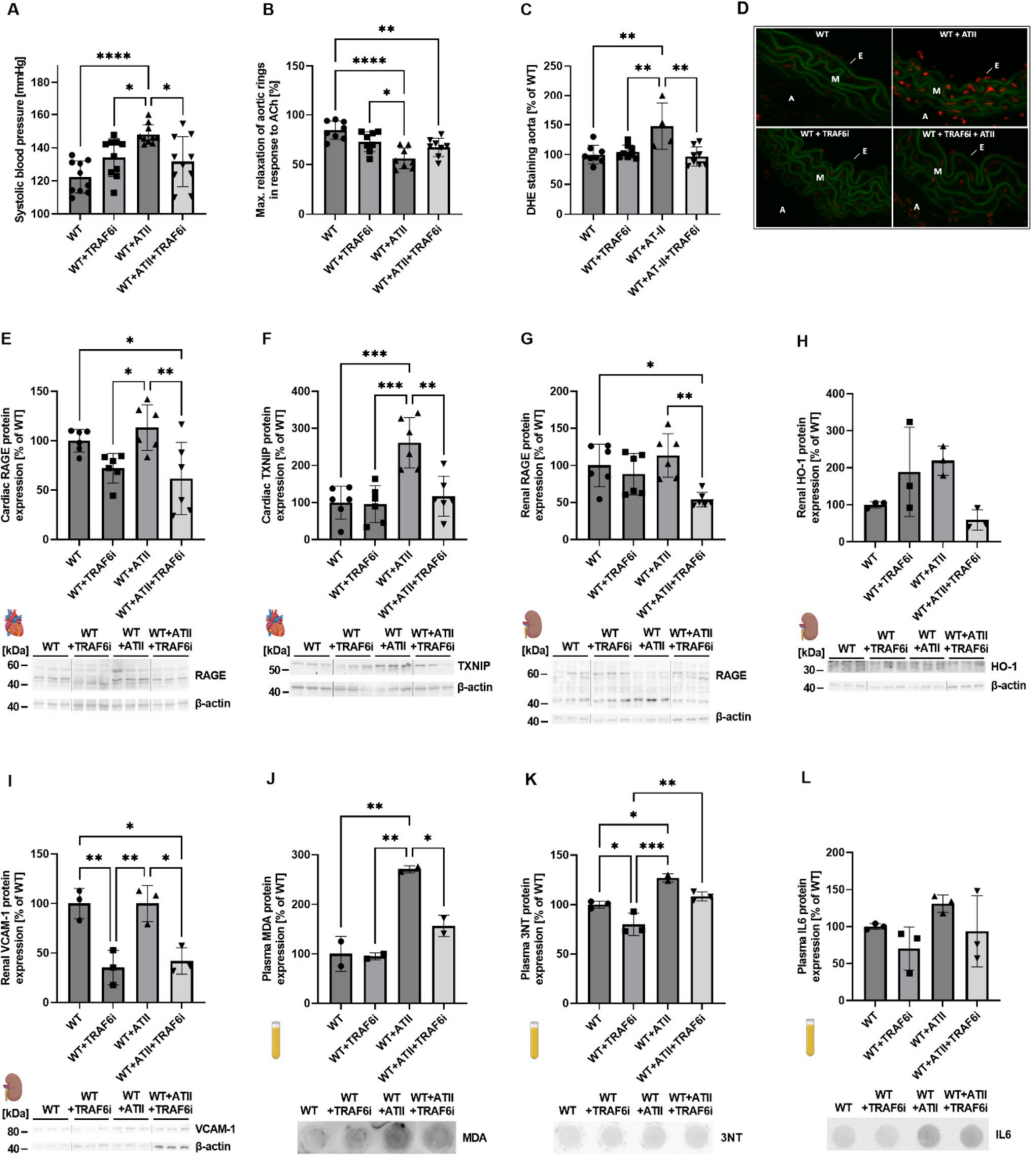
Pharmacological TRAF6 inhibition in hypertensive mice induced by angiotensin-II leads to decreased cardiac, renal, and plasma protein expression of representative markers for inflammation and oxidative stress. Systolic blood pressure was measured non-invasively by tail-cuff recordings (**A**). Isometric tension studies were performed to analyze the endothelial function (**B**). Aortic ROS levels were analyzed with DHE stainings (**C**), and representative pictures are shown in (**D**). Cardiac protein expression of RAGE (**E**) and TXNIP (**F**), as well as renal protein expression of RAGE (**G**), HO-1 (**H**), and VCAM-1 (**I**), was determined by immunoblotting. Representative blots are shown together with the densitometric quantification below. In addition, the protein expression of MDA (**J**), 3NT (**K**), and IL6 (**L**) was determined in plasma by dot blot analysis. Representative original dot blots are shown below the densiometric quantification. Data are mean ± SD of *n* = 10 (**A**), *n* = 8 (**B**), *n* = 4–8 (**C**), *n* = 6 (**E**–**G**), *n* = 3 (**H**, **I**), and *n* = 3 (**J**–**L**) animals per group. *p ≤ 0.05, **p ≤ 0.01 and ***p ≤ *0.001.* WT wild type (C57BL/6 J), *TRAF6i* TRAF6 inhibitor, *ACh* acetylcholine, *DHE* dihydroethidium, *E* endothel, *M* media, *A* adventitia, *ROS* reactive oxygen species, *RAGE* receptor for advanced glycosylation endproducts, *TXNIP* thioredoxin interacting protein, *HO-1* heme oxygenase, *VCAM-1* vascular cell adhesion protein, *MDA* malondialdehyde, *3NT* 3-nitrotyrosine, *IL6* interleukin 6

Arterial hypertension in WT mice was induced by angiotensin-II (ATII) application, leading to an increase in systolic blood pressure of 122 to 148 mmHg. TRAF6 inhibition in hypertensive animals significantly decreased the systolic blood pressure from 148 to 132 mmHg (Fig. 3A). Endothelial function was significantly impaired in hypertensive animals, which was prevented by trend by TRAF6 inhibitor treatment (*p* = 0.129) (Fig. 3B). Aortic ROS production in hypertensive animals was increased, which was normalized by TRAF6 inhibition (Fig. 3C, D). Cardiac RAGE, TXNIP, renal RAGE, and VCAM-1 expression significantly decreased in hypertensive animals after TRAF6 inhibitor treatment (Fig. 3E–G, I). Renal HO-1 expression was reduced in these animals by trend (*p* = 0.066) (Fig. 3H). Also, TRAF6 inhibition decreased the expression of the inflammatory marker 3NT by trend (*p* = 0.081), whereas IL6 was not changed significantly (Fig. 3K, L). Levels of MDA-positive proteins were reduced significantly (Fig. 3J).

### CHD patients with either hypertension or hypertension and diabetes show increased expression of oxidative stress and inflammatory markers in plasma and vascular bypass tissue

**Fig. 4:**
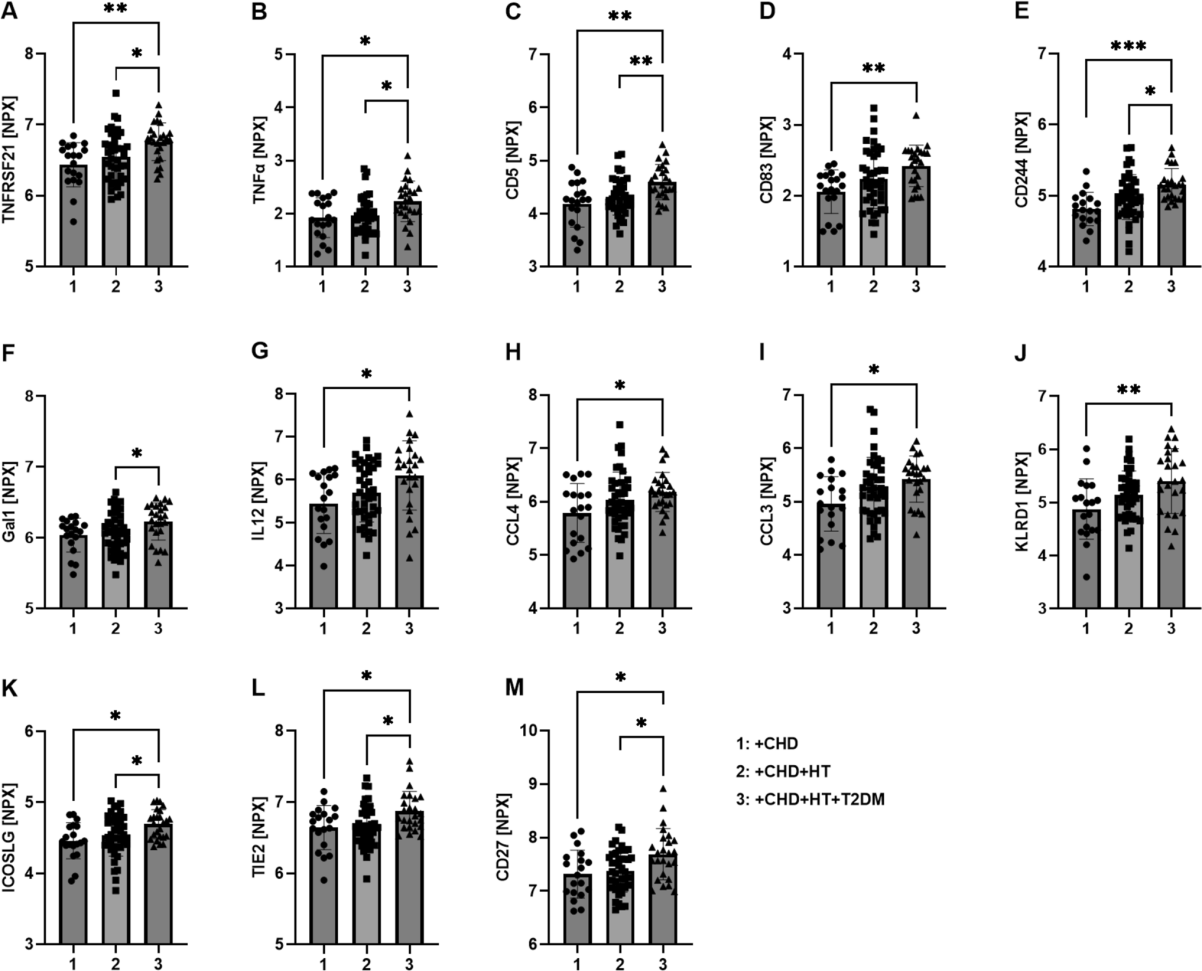
Selected markers of low-grade inflammation show a pattern of step-wise increase in plasma from CHD patients with either hypertension or hypertension and diabetes. Olink analysis in plasma from CHD patients with either no comorbidity (1) hypertension (2) or hypertension and diabetes (3) was performed, and the following step-wise increased targets (at least by trend) were identified: TNFRSF21 (**A**), TNFα (**B**), CD5 (**C**), CD83 (**D**), CD244 (**E**), Gal1 (**F**), IL12 (**G**), CCL4 (**H**), CCL3 (**I**), KLRD1 (**J**), ICOSLG (**K**), TIE2 (**L**), and CD27 (**M**). Data are mean ± SD CHD n = 19, CHD + HT *n* = 42, and CHD + HT + T2DM = 25 patients per group. *p ≤ 0.05, **p ≤ 0.01 and ***p ≤ *0.001.* Outliers were identified and removed with the ROUT (*Q* = 1%) method. *CHD* coronary heart disease, *HT* hypertension, *T2DM* type 2 diabetes mellitus, *TNFRSF* tumor necrosis factor receptor superfamily, *TNF* tumor necrosis factor, *Gal1* galectin1, *CCL* C–C motif chemokine ligand, *KLRD* killer cell lectin-like receptor D, *ICOSLG* inducible T cell co-stimulatory ligand, TIE2 angiopoietin 1 receptor

To explore whether the CD40L–CD40 pathway, as well as associated pathways, were upregulated in patients with CHD suffering from comorbidities, we performed plasma proteomic analysis of CHD patients with either hypertension (HT) or hypertension and diabetes (HT + T2DM) using the Olink IMMUNO-ONCOLOGY panel. A total of 23/92 markers showed significant changes compatible with a worse phenotype in the presence of comorbidities. Some members of the TNF(R)SF family, including CD27 and DR6 (TNFRSF21), followed a step-wise increasing pattern, although the increase in hypertension did not reach significance in contrast to patients with HT + T2DM. Also, other markers of inflammation, including TNFα, IL12, CCL4, and CCL3, followed a similar pattern. Immune cell markers, including CD83, CD5, CD244, ICOSLG, and KLRD1, were increased in patients suffering from comorbidities, as were TIE2 and Gal1 (Fig. 4A–M). In summary, a total of 13/92 markers showed significant changes and a pattern of step-wise increase with each additional comorbidity. The online supplemental data display other markers that are at least elevated by minor trend with one comorbidity without a clear pattern of step-wise increase (Suppl. Figs. S1, S2).

**Fig. 5:**
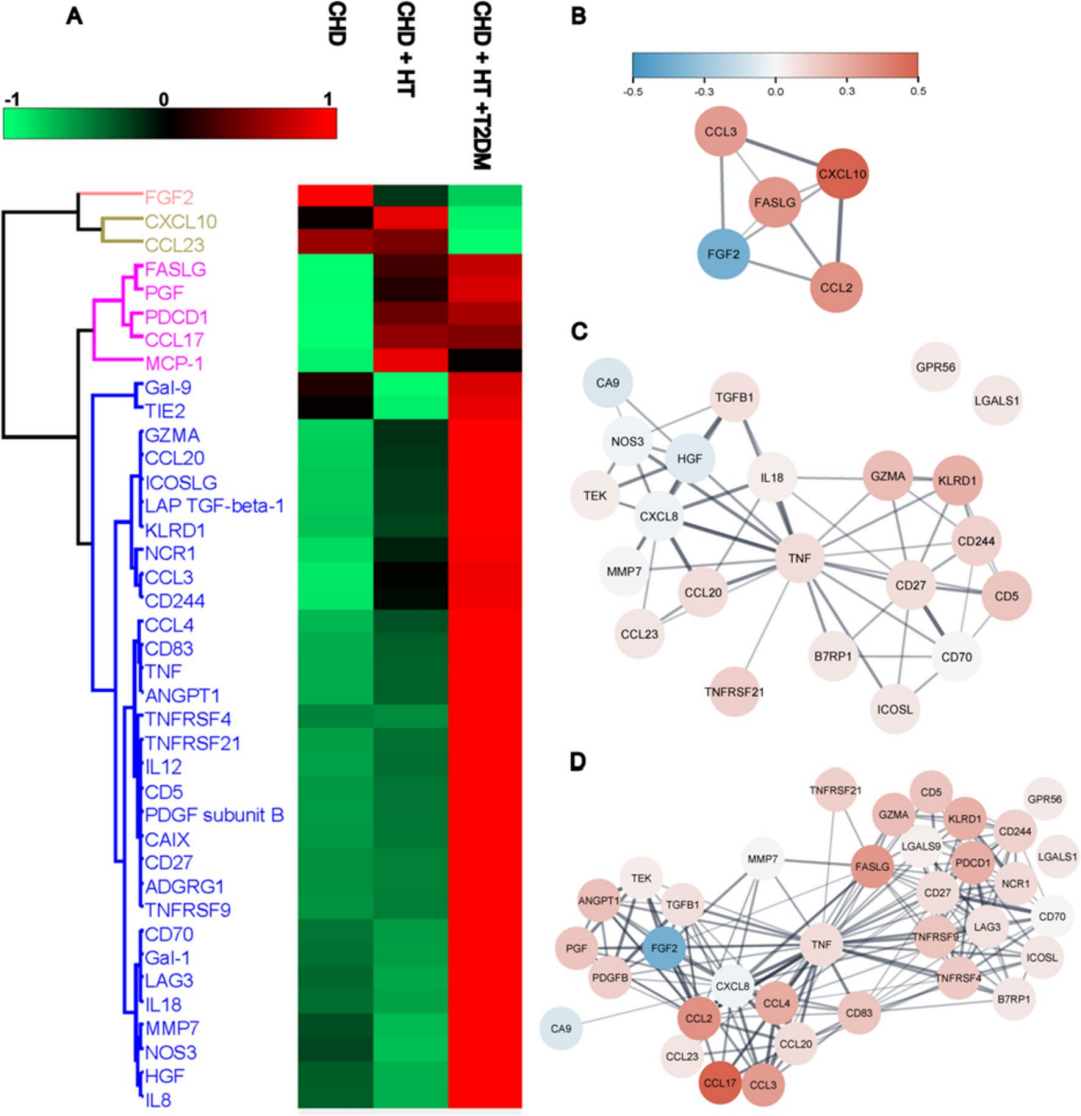
Markers of inflammation are increased in plasma from CHD patients with either hypertension or hypertension and diabetes. Olink analysis in plasma from CHD patients with either hypertension or hypertension and diabetes was performed, and the dataset was filtered for differentially expressed proteins. Median values were determined for each group and Z-normalised. **A** Euclidean Clustering of median expression. **B**–**D** Protein network of differentially expressed proteins with B comparing CHD to CHD + HT, C comparing CHD + HT to CHD + HT + T2DM, and D comparing CHD to CHD + HT + T2DM. *FGF* fibroblast growth factor, *CXCL* C-X-X motif chemokine ligand, *CCL* C–C motif chemokine ligand, *FASLG* Fas ligand, *PGF* placental growth factor, *PDCD* programmed cell death ligand, *MCP* monocyte chemoattractant protein, *Gal* galectin, *TIE2* angiopoietin receptor-2, *GZMA* granzyme A, *ICOSLG* inducible T cell co-stimulatory ligand, *LAP TGF* latency-associated peptide transforming growth factor, *KLRD* killer cell lectin-like receptor D, *NCR* natural cytotoxicity triggering receptor, *CD* cluster of differentiation, *TNF* tumor necrosis factor, *ANGPT1* angiopoietin-1, *TNFRSF* tumor necrosis factor receptor superfamily, *IL* interleukin, *PDGF* platelet derived growth factor, *CAIX* carbonic anhydrase, *ADGRG1* adhesion G-protein-coupled receptor, *LAG* lymphocyte activation gene, *MMP* matrix metalloproteinase, *NOS* nitric oxide synthase, *HGF* hepatocyte growth factor. Data are mean ± SD CHD *n* = 21, CHD + HT *n* = 42, and CHD + HT + T2DM = 25 patients per group

Cluster analysis of the proteins differentially expressed in the plasma revealed that T2DM elicited the most significant changes. Overall, we identified three clusters (Fig. 5A). Cluster one contains proteins downregulated by T2DM, cluster two proteins induced by HT and T2DM, whereas cluster three encompasses proteins upregulated by T2DM. Cluster three contains numerous cytokines linked to TNF, interleukins, and immune-cell surface markers. Network analysis of the proteins differentially expressed when comparing the comorbidities revealed that T2DM dysregulated the plasma proteome most significantly (Figs. 5B–D). Analysis of proteins regulated by T2DM revealed a closely connected network. When comparing CHD to CHD + HT + T2DM, we detected four subnetworks containing either growth factors (TGFB1, PGF, PDGFB, etc.), chemokines (CCL2, CCL3, etc.) or cell surface proteins (TNF receptors, PDCD1, CD5, etc.). Intriguingly, all three networks were linked by TNF, suggesting a potential central role for this cytokine/adipokine in the observed expression change linked to T2DM. In order to demonstrate the involvement of CD40L and CD40 in this low-grade inflammatory cluster, linear regression analysis identified a significant correlation of CD40L or CD40 with 38 or 56 other inflammatory targets (see Suppl. Tables S1 and S2). Those correlated targets that are central in the cluster analysis are highlighted in suppl. Figs. S3 and S4. Targets that were not included in the TNF-centered cluster depicted in Fig. 5 mostly showed weak linear correlation without reaching significance (e.g. TRAIL, TWEAK, IL33, DCN, PTN, MIC-A/B or ARG1).

Further dot blot analysis with the patient’s plasma was performed. The protein expression of NOX2 increased by trend in CHD patients with comorbidities (*p* = 0.0787 (NOX2)), whereas CD40L only showed a similar pattern (Suppl. Fig. S5). CD68 protein expression was significantly increased in CHD patients with HT + T2DM, whereas 3NT levels were significantly decreased in CHD patients with HT compared to CHD patients with HT + T2DM.

These data reveal that patients with CHD suffering from HT or HT + T2DM display elevated levels of markers of low-grade inflammation, also linked to the CD40L–CD40 co-stimulatory dyad.

### Hypertension or hypertension and diabetes show marked changes in mRNA expression levels of multiple genes

Aortic tissue samples from CHD, CHD + HT, or CHD + HT + T2DM patients were analyzed for total mRNA expression using the RNA-Seq method and analyzed with CLC genomics workbench (see Suppl. Fig. S6 for the Volcano plots). We found 2074 different expressed genes (DEG, p < 0.05) for the comparison of CHD + HT vs. CHD, 1695 DEGs for CHD + HT + T2DM vs. CHD, and 2928 DEGs for CHD + HT + T2DM vs. CHD + HT (see suppl. Tables S3, S4, S5). These DEGs were used for the pathway analyses using the Ingenuity Pathway Analysis (IPA, Qiagen). The regulated DEGs can also be assigned to certain signaling clusters such as CD40L–CD40–TRAF, immune system, hemostasis, muscle contraction, metabolism of lipids, developmental biology, and apoptosis (Suppl. Table S6).

The graphical summary of the IPA analysis is shown in Suppl. Fig. S7A–C. As expected, most pathways indicated as activated in CHD + HT vs. CHD patients (Suppl. Fig. S7A) are important for inflammatory reactions (e.g., the response of myeloid cells and phagocytes, activation of T lymphocytes and mononuclear leukocytes). Interestingly, one of the central pathways indicated to be activated is centered around CD40L. In the disease complexes of the patients, T2DM centrally changed the regulated pathways (Suppl. Fig. S7B). The function and contractility of different muscles are indicated to be inhibited. Also, the comparison CHD + HT + T2DM versus CHD + HT (Suppl. Fig. S7C) showed mainly inhibition of pathways, and also muscle activity is indicated to be regulated negatively.

Analyses of the top 5 regulated canonical pathways showed markedly enhanced mitochondrial dysfunction, oxidative phosphorylation, sirtuin pathways activation, neutrophil extracellular trap dependent signaling, and granzyme A activity in the CHD + HT patients compared to the CHD patients control (Suppl. Fig. S8A). Comparison of the CHD + HT + T2DM group with the CHD control group showed enhanced granulocyte diapedesis, reduced calcium pathway activity, reduced dilated cardiomyopathy, enhanced hepatic fibrosis, and reduced effects of sildenafil (Suppl. Fig. S8B). Finally, the comparison of the CHD + HT + T2DM group with the CHD + HT group showed a marked reduction of oxidative phosphorylation, mitochondrial dysfunction, sirtuin signaling, estrogen receptor activity, and calcium-dependent regulations (Suppl. Fig. S8C).

The IPA analysis of the top 5 upstream regulators showed enhanced activities of the factors TEAD (transcription factor), PPARGC1A (transcriptional coactivator), CD40 (receptor of CD40L), INSR (insulin receptor), and Hbb-b1 (hemoglobin, beta adult major chain) in the CHD + HT compared to the CHD group (Suppl. Fig. S9A). Compared to the CHD control group, the CHD + HT + T2DM group showed enhanced effects of SIX1 (transcription factor) and reduced activities of DMD (human dystrophin gene), MYOD1 (transcription factor), MEF2C (transcription factor), and beta-estradiol (sex hormone; Suppl. Fig. S9B). Finally, the CHD + HT + T2DM group reduced TEAD and DMD activities, whereas enhanced SIX1, CLPP (mitochondrial protease component), and CPT1B (carnitine palmitoyltransferase 1B) activities were observed compared to the CHD + HT group (Suppl. Fig. S9C).

## Discussion

This study establishes that inflammatory signaling via the CD40L–CD40–TRAF6 axis plays a crucial role in diabetic (db/db) and hypertensive (ATII) mice. We also demonstrate that TRAF6 inhibition has beneficial effects in these animal models. In addition, we show that some markers (like CD40L, NOX2, and 3NT) identified in the mouse models showed a pattern of step-wise upregulation in CHD patients by the presence of comorbidities such as hypertension or hypertension + diabetes. CD40L and CD40 were also significantly correlated with an appreciable number of targets of a low-grade inflammatory cluster in hypertensive/diabetic patients.

### Animal data

A previous study showed that TRAF6 inhibition (often also termed “CD40-TRAF6” inhibition) by compound 6877002 suppressed the expression of TNFα, CCL2, and different interleukins in activated macrophages and diminished the expression of TNFα, CD40, and CD40L by trend in isolated B cells [37]. We showed that TRAF6 inhibition in diabetic mice (db/db), at least by trend, normalizes the upregulation of proteins centered around platelet activation (e.g., CD40L, TSP1, PAR1). Previously, we established that TRAF6 inhibition partially prevented endothelial dysfunction, weight gain, increase in HbA1c values, oxidative burst of whole blood leukocytes, cardiac RAGE signaling, vascular ROS formation, and impaired catalase/glutathione peroxidase 1 expression in db/db mice [38]. It is already known that the release of soluble CD40L and mainly CD40L–CD40 mediated signaling of platelets, endothelial cells, and monocytes strongly affects CHD [1, 13, 39]. Indeed, one way of expressing CD40 and mobilizing CD40 on the membrane in endothelial cells is induced via PAR-1, which could represent another link between thrombosis and inflammation [30]. Besides sCD40L, TSP-1 could be a potential biomarker in diagnosing acute coronary syndrome (ACS) [15]. We conclude from this data that inhibition of the CD40–TRAF6 signaling cascade in diabetic mice is suitable for suppressing thrombosis and the progression of CD40L–CD40-mediated inflammation.

Inhibition of TRAF6 also decreased the expression of proteins that are involved in reactive oxygen species formation (e.g., p47phox, NOX2), inflammatory signaling (e.g., VCAM-1, RAGE), and cell death (e.g., caspase-3) in diabetic animals. NADPH oxidase (NOX2) activation is associated with superoxide production, activation of NF-κB signaling, and release of pro-thrombotic molecules (e.g., sCD40L). Also, high CD40 surface protein levels affect the activation of NOX2 [32] [28]. In addition, RAGE could be activated via advanced glycation end products (AGE) to enhance the expression of adhesion and inflammatory molecules, including CD40 expression and mobilization to the surface of monocytes, which results in increased T cell activation and TNFα and interferon-γ levels [40]. With our present data, we could show that these processes are suppressed by TRAF6 inhibition in diabetic mice. Also, upregulated eNOS, reflecting compensatory upregulation of the uncoupled enzyme, was normalized by inhibition of the CD40–TRAF6 pathway in the heart and kidney. Furthermore, renal phosphorylation of MARCKS, reflecting p47phox-mediated NOX2 activation, and antioxidant response (e.g., HO-1) and vasoconstrictor expression (e.g., ET-1) was suppressed by TRAF6 inhibition in db/db mice. Also, aortic ROS formation was increased in diabetic mice, leading to endothelial dysfunction, all of which was improved, at least by trend, by TRAF6 inhibitor treatment. In contrast, the severely increased blood glucose levels in db/db mice were not decreased by TRAF6 inhibition.

Previous data indicate that ATII-mediated arterial hypertension is related to increased CD40L expression, most likely mediated by monocytes [43] and endothelial cells [19, 24]. Accordingly, we here observed a similar trend for the protective effects of TRAF6 inhibition in hypertensive mice as documented in diabetic mice. Indeed, in hypertensive mice with TRAF6 inhibitor treatment, cardiac RAGE and TXNIP expression were significantly reduced. TXNIP is a key component of high-mobility group box 1 protein (HMGB1) and NLR family pyrin domain containing 3 protein (NLRP3) activation, leading to inflammation. In addition, renal RAGE, HO-1, and VCAM-1 showed a similar trend in hypertensive mice. Oxidative stress markers such as 3NT, MDA, and 4HNE were upregulated in the blood or kidney of diabetic or hypertensive mice and normalized by CD40-TRAF6 inhibition. Of note, these biochemical changes were also mirrored by functional changes—TRAF6 inhibition partially normalized increased systolic blood pressure, endothelial dysfunction, and aortic ROS formation in hypertensive mice. Moreover, some of the mentioned biochemical markers (like CD40L, NOX2, and 3NT) identified in the mouse models were also step-wise upregulated in CHD patients by the presence of comorbidities such as hypertension or hypertension + diabetes.

### Human protein data

Increased cardiovascular risk is observed in patients suffering from systemic inflammatory diseases [6, 31]. Therefore, CVDs were classified as an inflammatory-related entity [3]. This correlation was also demonstrated in patients with ACS in which C-reactive protein (CRP) levels positively correlated with adverse clinical outcomes [33]. In the CANTOS trial, pharmacological agents like Canakinumab, an IL-1β antagonist, are employed in patients with high cardiovascular risk as anti-inflammatory therapy to improve their outcomes [34]. Besides inflammation, oxidative stress represents a hallmark of most CVDs, characterized by a mismatch between ROS formation and degradation [17, 20], e.g., as shown by a positive correlation between glutathione peroxidase-1 and outcomes in patients with CVD [8] as well as markers of oxidative stress and cardiovascular mortality [36].

In our proteomic analysis of CHD patients’ plasma, we identified some step-wise increased targets in association with the number of their comorbidities (CHD < CHD + HT < CHD + HT + T2DM). Cluster analysis identified TNFα as a central player. Detailed extended discussion of all regulated targets and the cluster data are provided in the online Supplemental Data file. Taken together, the central position of TNFα in the plasma protein cluster analysis provides evidence for an involvement of the CD40(L)-TRAF6 pathway in patients with HT and T2DM as comorbidities, which is further supported by the substantial correlations of CD40L and CD40 with a majority of other targets of the low-grade inflammatory cluster (Suppl. Tables S1 and S2, Figs. S3 and S4). Previous work showed that silencing of CD40 or TNF receptor (TNFR)-associated factor 6 (TRAF6) gene largely abrogated phosphorylation and nuclear translocation of NF-κB (p65), and hence TNFα expression [5]. Blockade of CD40 and CD40L interactions with neutralizing antibodies significantly reduced monocyte release of inflammatory mediators and migration by CCL2 (MCP-1). Another study reported upregulation of CD40, TRAF2, and TRAF6 in patients with diabetic retinopathy, where CD40 was associated with the expression of pro-inflammatory molecules such as intercellular adhesion molecule 1 (CD54), CCL2, and TNFα [42]. Several other factors identified in the cluster analysis of patients with HT and T2DM were previously reported for pathways associated with CD40L and other tumor necrosis factor superfamily ligands in HIV infection [23]. It is also well established that TNFα cooperates with CD40L, RANK, and IL1β to regulate TRAF6/NF-κB signaling [29], which is also supported by the observation that TRAF6 inhibition by compound 6877002 prevents CD40L-dependent activation (nuclear translocation) of NF-κB [7].

### Human RNA sequencing data

In accordance with our proteomic data, we found in CHD patients with comorbidities (CHD < CHD + HT + T2DM) increased aortic gene expression of NK cell-specific markers (e.g., CD244, KLRD1, NCR1), apoptotic markers (e.g., Gal1, TNFRSF21), cytokines/chemokines (e.g., TNF, CCL3, CCL4, IL12, IL8), T cell activation-associated markers (e.g., TNFRSF4, TNFRSF9, CD5, ICOSLG, CD70), and pro-angiogenic markers (e.g., AngPT1, TIE2).

The comparison of our proteomic data with our RNA-seq data (see Suppl. Table S8) showed only a small overlap in the regulated genes and differences in the regulation direction. In the RNA-seq analysis of the CHD + HT vs. CHD group, only CD8 and CD5 were upregulated, and CCL13 (MCP4) and CA9 (CAIX) were downregulated. There were no significant changes in the mRNA expression of the other genes. The comparative analysis of the proteomic data with the RNA-seq data of the comparison CHD + HT + T2DM vs. CHD shows more genes regulated on the mRNA expression level. However, in this comparison, several genes are downregulated on the mRNA level, which has been found to be upregulated on the protein level in the plasma. In this group 4 genes (PGF, AngPT1, CXCL8, NCR1) showed enhanced mRNA expression, whereas 5 genes (CA9, GAL, CXCL11, TNFRSF21, LAG3) showed reduced mRNA expression. However, as protein expression in the plasma (whole body) and mRNA expression in aorta samples are supposed only to show a minor overlap, our data are not surprising. Detailed extended discussion of IPA analysis of RNA-seq data is provided in the online Supplemental Data file, including suppl. Figs. S7,S8,S9.

The proteomic and transcriptomic data of human samples are further supported in this study by protein expression analysis. In patients’ plasma, we could observe the same trend of upregulation for proteins involved in reactive oxygen formation/oxidative stress (e.g., NOX-2, 3NT) and inflammation (e.g., CD40L, CD68), like in the proteomic analysis before (Suppl. Fig. S5).

### Limitations of the study

The chosen mouse model for diabetes (db/db) develops a quite severe vascular phenotype, as reported previously [38]. However, even a short-term TRAF6 inhibition partially reversed vascular complications such as impaired endothelial function, vascular ROS formation, and increased HbA1c and RAGE signaling [38]. Our mouse model of induced arterial hypertension via ATII treatment reflects only one possible pathway underlying the development of hypertension. In addition, the model of hypertension with one week of ATII infusion does not fully reflect hypertension-triggered end-organ damage by remodeling and accumulating oxidative modifications (e.g., in the kidney and heart). Accordingly, the impact of TRAF6 inhibition on end-organ damage could not be tested to a full extent here. However, we have previously shown that CD40L deficiency prevents all major complications induced by ATII treatment [19]. Hypertension development is multifactorial in humans, with the renin–angiotensin–aldosterone system playing a substantial role. Nevertheless, we think this model is suitable for our studies, especially in the context of the CD40L–CD40–TRAF signaling. We could show in the past that the ATII model is primarily driven by the immigration of (CD40L expressing) inflammatory cells. The negative influence of ATII-induced arterial hypertension on vascular function and severe blood pressure increase is well documented and has been intensively investigated, particularly in our research group [19, 43] and by others [14, 26]. Although the ATII-based hypertension mouse or rat model causes severe hypertension and drastic elevation of oxidative stress and inflammation, it is frequently used to study the pathophysiology of arterial hypertension in a short time window of 1–2 weeks. The beneficial action of all classical antihypertensive drugs, such as AT1-receptor blockers and angiotensin-converting enzyme inhibitors, supports the relevance of these models for human hypertension.

Concerning the Olink proteomic data, the variability within individuals arises from several factors aside from the differentiating morbidities/comorbidities. Circadian rhythms, environmental stressors, age, diet, and known or unknown factors can regulate the plasma proteome. The cohort size was too small to account for these confounding factors. They can be overcome by increasing the sample number. We quantified the plasma proteome of 88 individuals with a range of comorbidities. The number of patients in the individual cohorts is likely limiting; we also cannot rule out unexpected confounding factors. In addition, we monitored a small number of proteins in a targeted fashion, limiting the breadth of analysis.

Compared to RNA-Seq data obtained with inbred animals, there is a greater heterogeneity in the transcriptomes of tissue samples obtained from human beings. These heterogeneities are likely related to differences in the DNA sequences of the different participants and, therefore, genetically related differences in the transcriptional activity. This fact results in lower p-levels in gene expression compared to healthy and diseased participants. This higher variance may result in undetected disease-related transcriptomic changes. To circumvent the limitations of the RNA-Seq method, larger numbers of healthy/ill tissues are needed. However, the obtainment of healthy aorta samples is quite problematic. The analysis of our RNA-Seq data showed relatively high heterogeneity in the mRNA expression in samples of the participants of one group. However, for example, the IPA analyses of the CHD + HT vs. CHD comparison resulted in detecting the CD40L–CD40 pathway as a major regulator of the gene expression changes, which fits quite well with our animal data.

Another limitation of our RNA-Seq studies is the usage of whole aorta specimens. As in this specimen, different cells (endothelial cells, smooth muscle cells, fibroblasts, immune cells, etc.) are included; the specific gene expression cannot be related to a specific cell type. In addition, infections may change the number of immune cells in the specimen and disturb the data.

As all participants in the study were treated with several drugs (partially overlapping between the different groups), the effects of the drugs on gene expression may also mask disease-related transcriptional changes.

Finally, since samples were used from a biobank, the included patients had no follow-up since this was not foreseen. Also, the sample size in two of the patient groups was quite low (*n* = 21 for CHD and *n* = 25 for HT + Dia), making the human study an exploratory pilot study. Also, the lack of the CHD patient group with only diabetes as a comorbidity represents a major limitation of the human part of the study.

### Conclusion and clinical impact

**Fig. 6:**
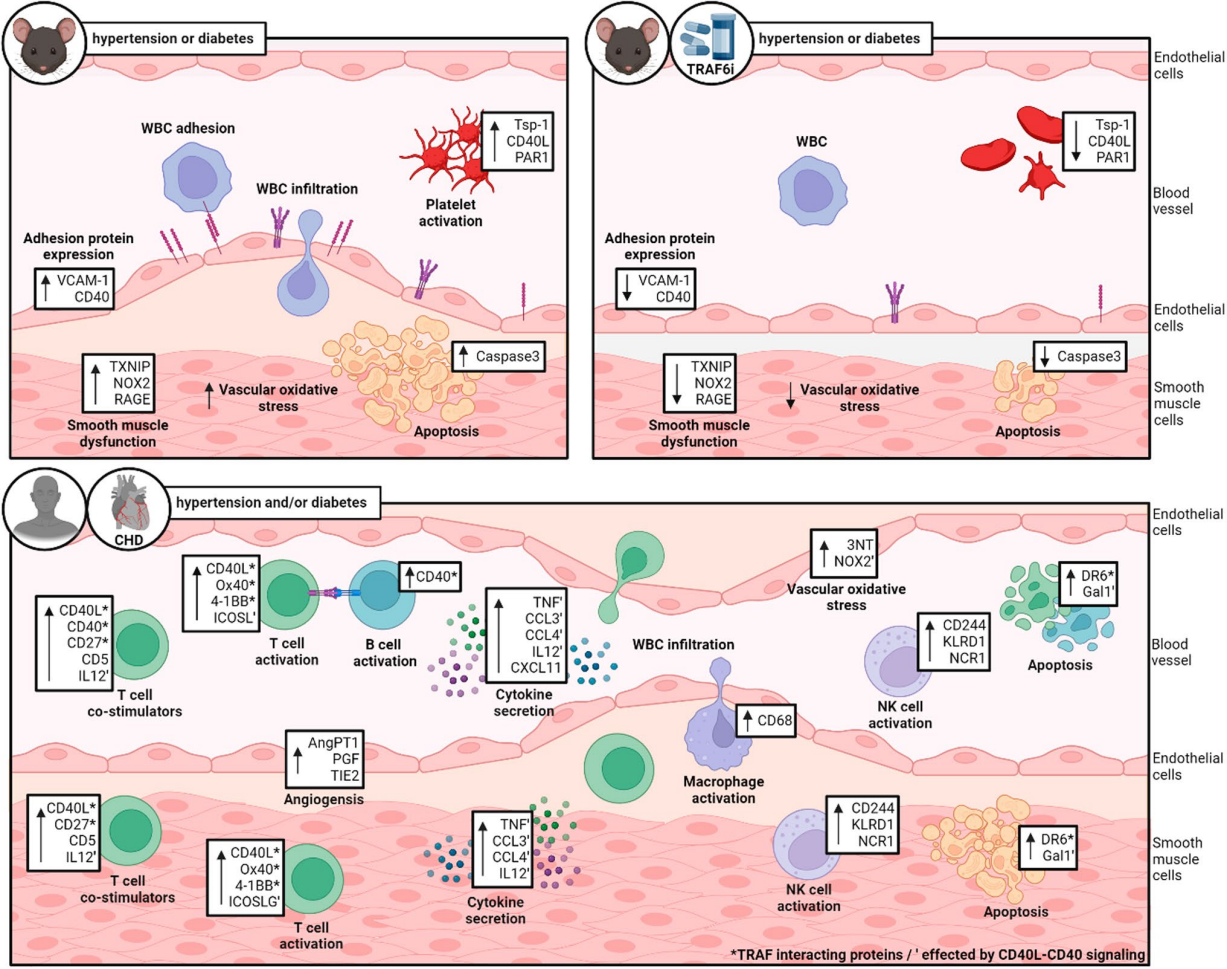
Central scheme. Upper left panel: the central pathomechanisms associated with CD40(L)–TRAF signaling in hypertensive or diabetic mice. Upper right panel: protective effects of TRAF6i treatment in hypertensive or diabetic mice. Lower panel: markers associated with CD40(L)–TRAF signaling observed in CHD patients with no comorbidity or hypertension or hypertension + diabetes. It must be stressed that an appreciable part of the targets mentioned here were only changed by trend. *WBC* white blood cell, *Tsp-1* thrombospondin-1, *CD* cluster of differentiation, *CD40L* CD40 ligand, *PAR1* protease-activated receptor 1, *VCAM-1* vascular cell adhesion molecule-1, *TXNIP* thioredoxin-interacting protein, *NOX2* NADPH oxidase 2, *RAGE* receptor for advanced-glycation-end-products, *TNF* tumor necrosis factor, *TRAF6i* TNF receptor associated factor-6 inhibitor, *CHD* coronary heart disease, *IL12* interleukin-12, *TNFRSF4 (or ox40 or CD134)* TNF receptor superfamily member-4, *TNFRSF9 (or 4-1BB or CD137)* TNF receptor superfamily member-9, *ICOSL* inducible costimulator ligand, *AngPT1* angiopoietin-1, *PGF* placental growth factor, *TIE2* angiopoietin receptor-2, *CCL3/CCL4* C–C motif chemokine ligand-3/-4, *CXCL11* C-X-C motif chemokine ligand-11, *3NT* 3-nitrotyrosine, *KLRD1* killer cell lectin like receptor D-1, *NCR1* natural cytotoxicity triggering receptor-1, *DR6* death receptor-6, *Gal1* galactin-1, *NK* natural killer cell. Created with BioRender.com

Recent large clinical trials demonstrated that anti-inflammatory therapy can reduce cardiovascular risk by broad suppression of inflammatory pathways, as shown by the COLCOT study [41], or by highly specific targeting of the inflammatory cascade, e.g., as shown by the CANTOS study [34, 35]. Our present study could link CD40L–CD40-mediated signaling and platelet activation, the activation of different leukocytes, oxidative stress, inflammation, and angiogenesis, thereby generating new hypotheses. The major pathomechanisms in hypertensive or diabetic mice are associated with CD40(L)–TRAF signaling. Also, in patients with CHD, some key mediators of this pathway were found (summarized in Fig. 6). We conclude that CD40L–CD40 signaling is important in mediating cardiovascular complications in mouse models of obesity, dyslipidemia, hyperglycemia, and arterial hypertension. In addition, the proteomic and genomic analysis in plasma and vascular bypass tissue of CHD patients without comorbidities or with the comorbidities hypertension and/or hypertension + diabetes identified some key mediators of CD40(L)–TRAF signaling, centered around TNFα. However, the present data cannot clarify whether the step-wise increase in levels of low-grade inflammation markers is mediated directly by the comorbidities hypertension and/or diabetes or by the more severe CHD/events driven by these comorbidities; however, the disease state characteristics did not change significantly by the comorbidities.

In conclusion, hypertension and lipid metabolic disorders are important risk factors and leading causes of atherothrombotic vascular diseases like MI and cerebral ischemia. A targeted CD40-TRAF6 inhibitor treatment could provide a novel therapeutic strategy for these diseases, which circumvents the potential risks of a general blockade of CD40L or CD40-like thromboembolic events and solid immune suppression. It remains to be established if a long-term CD40-TRAF6 inhibitor treatment also provides some risks, like the blockade of CD40L or CD40. Finally, human studies are needed to test the beneficial effects of TRAF6 inhibition in hypertension and diabetes.

### Supplementary Information

Below is the link to the electronic supplementary material.

Supplementary file1 (PDF 2609 KB)

Supplementary file2 (DOCX 261 KB)

Supplementary file3 (DOCX 218 KB)

Supplementary file4 (DOCX 400 KB)

Supplementary file5 (DOCX 526 KB)

Supplementary file6 (DOCX 20 KB)

## Data Availability

All data are available in the manuscript or the supplementary materials.

## Abbreviations

3NT: 3-Nitrotyrosine
4HNE: 4-Hydroxynonenal
ACS: Acute coronary syndrome
AngPT1: Angiopoetin 1
ATII: Angiotensin-II
CAIX: Carbonic anhydrase
CCL: C–C-chemokine ligand
CD: Cluster of differentiation
CD40L: CD40 Ligand
CHD: Coronary heart disease
CLPP: Caseinolytic mitochondrial matrix peptidase
CPT1B: Carnitine palmitoyltransferase 1B
CVD: Cardiovascular disease
CXCL: C–X–C chemokine ligand
DR6: Death receptor 6
DMD: Dystrophin
eNOS: Endothelial-nitric-oxide-synthase
ET-1: Endothelin-1
FASLG: Fas ligand
Gal: Galactin
GZMA: Granzyme A
HDL: High-density lipoprotein
HT: Hypertension
HMGB1: High mobility group box 1
HO-1: Heme oxygenase 1
ICOSLG: Inducible T cell co-stimulatory ligand
IL6: Interleukin 6
IMA: Internal mammary artery
IPA: Ingenuity pathway analysis
KLRD1: Killer cell lectin-like receptor subfamily D, member 1
Lag3: Lymphocyte-activation gene 3
MDA: Malondialdehyde
MCP: Monocyte chemoattractant peptide
MI: Myocardial infarction
MMP: Matrix metalloproteinase
MYOD1: Myogenic differentiation 1
NADPH: Nicotinamide adenine dinucleotide phosphate
NCR1: Natural cytotoxicity triggering receptor 1
NF-κB: Nuclear factor kappa light chain enhancer of activated B cells
NLRP3: NLR family pyrin domain containing 3
NOX2: NADPH oxidase 2
p47phox: Neutrophil cytosol factor 1
PAR1: Protease-activated receptor 1
PGF: Placental growth factor
pMARCKS: P-myristoylated-glycation-end-products
RAGE: Receptor for advanced glycosylation end products
ROS: Reactive oxygen species
SIX1: Sine oculis homeobox homolog 1
T2DM: Type 2 diabetes mellitus
TEAD: TEA domain transcription factor
TGFB1: Transforming growth factor beta 1
TIE2: Angiopoietin receptor-2
TNF: Tumor necrosis factor
TNFRSF: TNF receptor superfamily
TRAF: TNF receptor-associated factor
TSP-1: Thrombospondin-1
TXNIP: Thioredoxin-interacting protein
VCAM-1: Vascular cell adhesion molecule-1
