# Supplementary material for: Role of inflammatory signaling pathways involving the CD40–CD40L–TRAF cascade in diabetes and hypertension—insights from animal and human studies
Source: Basic Res Cardiol. 2024 Mar 30;119(4):1–18. doi: 10.1007/s00395-024-01045-1 (PMC11319409; doi:10.1007/s00395-024-01045-1)
Supplement: Supplementary file 1 — Supplementary file1 (PDF 2609 KB) [file 395_2024_1045_MOESM1_ESM.pdf]

# Online supplemental data

# Role of inflammatory signaling pathways involving the CD40-CD40L-TRAF cascade in diabetes and hypertension - insights from animal and human studies

Lea Strohm[1*], Andreas Daiber[1,2*], Henning Ubbens[1], Roopesh Krishnankutty[3], Matthias Oelze[1], Marin Kuntic[1], Omar Hahad[1,2], Veronique Klein[1], Imo E. Hoefer[4], Alex von Kriegsheim[3], Hartmut Kleinert[5], Dorothee Atzler[6,7,8], Philipp Lurz[1,2], Christian Weber[6,7,9,10], Philipp Wild[2,11], Thomas Münzel[1,2], Christoph Knosalla[12,13,14], Esther Lutgens[6,7,15], and Steffen Daub[1]

[1] Department of Cardiology, Cardiology I, University Medical Center of the Johannes Gutenberg-University, Mainz, Germany; [2] German Center for Cardiovascular Research (DZHK), Partnersite Rhine-Main, Mainz, Germany; [3] Institute of Genetics and Cancer, University of Edinburgh, UK; [4] Laboratory for Experimental Cardiology, UMC Utrecht, Utrecht, The Netherlands; [5] Department of Pharmacology, University Medical Center of the Johannes Gutenberg-University, Mainz, Germany; [6] Institute for Cardiovascular Prevention (IPEK), Ludwig-Maximilians Universität, Munich, Germany; [7] German Center for Cardiovascular Research (DZHK), Partner Site Munich Heart Alliance, Germany; [8] Walther Straub Institute of Pharmacology and Toxicology, LMU Munich; [9] Munich Cluster for Systems Neurology (SyNergy), Munich, Germany; [10] Department of Biochemistry, Cardiovascular Research Institute Maastricht (CARIM), Maastricht University, the Netherlands; [11] Preventive Cardiology and Preventive Medicine, Department of Cardiology, University Medical Center of the Johannes Gutenberg-University, Mainz, Germany; [12] Department of Cardiothoracic and Vascular Surgery, Deutsches Herzzentrum der Charité, Berlin, Germany; [13] Charité-Universitätsmedizin Berlin, corporate member of Freie Universität Berlin and Humboldt-Universität zu Berlin, Germany; [14] German Center for Cardiovascular Research (DZHK), Partner Site Berlin, Berlin, Germany; [15] Mayo Clinic, Dept Cardiovascular Medicine and Immunology, Rochester, MN, USA.

## Supplemental Tables

**Table S1. Significant correlations between CD40L and other targets of inflammation (based on the olink plasma proteome data). Linear regression graphs are shown for selected targets in suppl. Figure S3.**

| Target | R squared | p-value |
|---|---|---|
| IL8 | 0.09705 | 0.0031 |
| MCP-1 | 0.1513 | 0.0002 |
| CD244 | 0.2221 | <0.0001 |
| EGF | 0.6973 | <0.0001 |
| ANGPT1 | 0.209 | <0.0001 |
| IL7 | 0.1756 | <0.0001 |
| CXCL11 | 0.1933 | <0.0001 |
| FGF2 | 0.05776 | 0.0258 |
| CXCL9 | 0.05972 | 0.0234 |
| CAIX | 0.05022 | 0.0381 |
| NOS3 | 0.05776 | 0.0258 |
| GAL9 | 0.08883 | 0.0053 |
| CD40 | 0.2517 | <0.0001 |
| IL18 | 0.05412 | 0.0311 |
| LAP TGF-beta-1 | 0.3965 | <0.0001 |
| CXCL1 | 0.3197 | <0.0001 |
| TNFSF14 | 0.2454 | <0.0001 |
| PGF subunit B | 0.2756 | <0.0001 |
| CD28 | 0.06351 | 0.0192 |
| CCL4 | 0.08462 | 0.0066 |
| PD-L1 | 0.08668 | 0.0059 |
| CXCL5 | 0.1935 | <0.0001 |
| HGF | 0.04982 | 0.0389 |
| CXCL10 | 0.05249 | 0.0339 |
| CCL3 | 0.09938 | 0.0031 |
| TNFRSF4 | 0.07841 | 0.009 |

| | | |
|---|---|---|
| CCL17 | 0.2312 | <0.0001 |
| ANGPT2 | 0.07227 | 0.0123 |
| CXCL12 | 0.07453 | 0.011 |
| CASP-8 | 0.1192 | 0.0011 |
| CXCL13 | 0.09615 | 0.0037 |
| PD-L2 | 0.05594 | 0.0283 |
| VEGFA | 0.1566 | 0.0002 |
| IL12RB1 | 0.05627 | 0.0279 |
| CCL20 | 0.05141 | 0.0358 |
| TNF | 0.05947 | 0.0237 |
| GZMB | 0.05339 | 0.0323 |
| CSF1 | 0.0503 | 0.0379 |

**Table S2. Significant correlations between CD40 and other targets of inflammation (based on the olink plasma proteome data). Linear regression graphs are shown for selected targets in suppl. Figure S4.**

| Target | R squared | p-value |
|---|---|---|
| IL8 | 0.09362 | 0.0042 |
| TNFRSF9 | 0.414 | <0.0001 |
| TIE2 | 0.09603 | 0.0037 |
| CD40L | 0.2517 | <0.0001 |
| CD244 | 0.2327 | <0.0001 |
| EGF | 0.2454 | <0.0001 |
| PGF | 0.4176 | <0.0001 |
| IL6 | 0.05072 | 0.0371 |
| MCP-1 | 0.0587 | 0.0246 |
| CRTAM | 0.1673 | <0.0001 |
| CXCL11 | 0.1491 | 0.0002 |
| FGF2 | 0.04644 | 0.0463 |
| CXCL9 | 0.0927 | 0.0044 |
| CAIX | 0.06003 | 0.023 |
| MUC-16 | 0.1234 | 0.0009 |
| ADA | 0.1346 | 0.0005 |
| CD4 | 0.1181 | 0.0012 |
| Gal-9 | 0.2652 | <0.0001 |
| IL18 | 0.1053 | 0.0023 |
| LAP-TGF-beta-1 | 0.4704 | <0.0001 |
| CXCL1 | 0.1603 | 0.0001 |
| TNFSF14 | 0.0489 | 0.0407 |
| PDGF subunit B | 0.0471 | 0.0447 |
| PDCD1 | 0.1142 | 0.0015 |
| CCL4 | 0.06077 | 0.0221 |
| IL15 | 0.1968 | <0.0001 |
| Gal-1 | 0.3141 | <0.0001 |

| | | |
|---|---|---|
| PD-L1 | 0.4087 | <0.0001 |
| CD27 | 0.3411 | <0.0001 |
| CXCL5 | 0.08074 | 0.008 |
| HO-1 | 0.06589 | 0.017 |
| CX3CL1 | 0.3466 | <0.0001 |
| CD70 | 0.1976 | 0.0006 |
| TNFRSF12A | 0.3756 | <0.0001 |
| CCL23 | 0.1308 | <0.0001 |
| CD5 | 0.3371 | <0.0001 |
| CCL3 | 0.2436 | <0.0001 |
| MMP7 | 0.2312 | <0.0001 |
| NCR1 | 0.2424 | <0.0001 |
| TNFRSF21 | 0.3708 | <0.0001 |
| TNFRSF4 | 0.47 | <0.0001 |
| CCL17 | 0.09791 | 0.0034 |
| ANGPT2 | 0.2127 | <0.0001 |
| INF-gamma | 0.09656 | 0.0036 |
| LAMP3 | 0.1811 | <0.0001 |
| CASP-8 | 0.09823 | 0.0033 |
| ICOSLG | 0.1678 | <0.0001 |
| MMP12 | 0.155 | 0.0002 |
| CXCL13 | 0.04937 | 0.0398 |
| VEGFA | 0.3901 | <0.0001 |
| CCL20 | 0.06299 | 0.0198 |
| TNF | 0.211 | <0.0001 |
| KLRD1 | 0.05902 | 0.0242 |
| CD83 | 0.2423 | <0.0001 |
| IL12 | 0.07972 | 0.0084 |
| CSF-1 | 0.2701 | <0.0001 |

## Supplemental figures

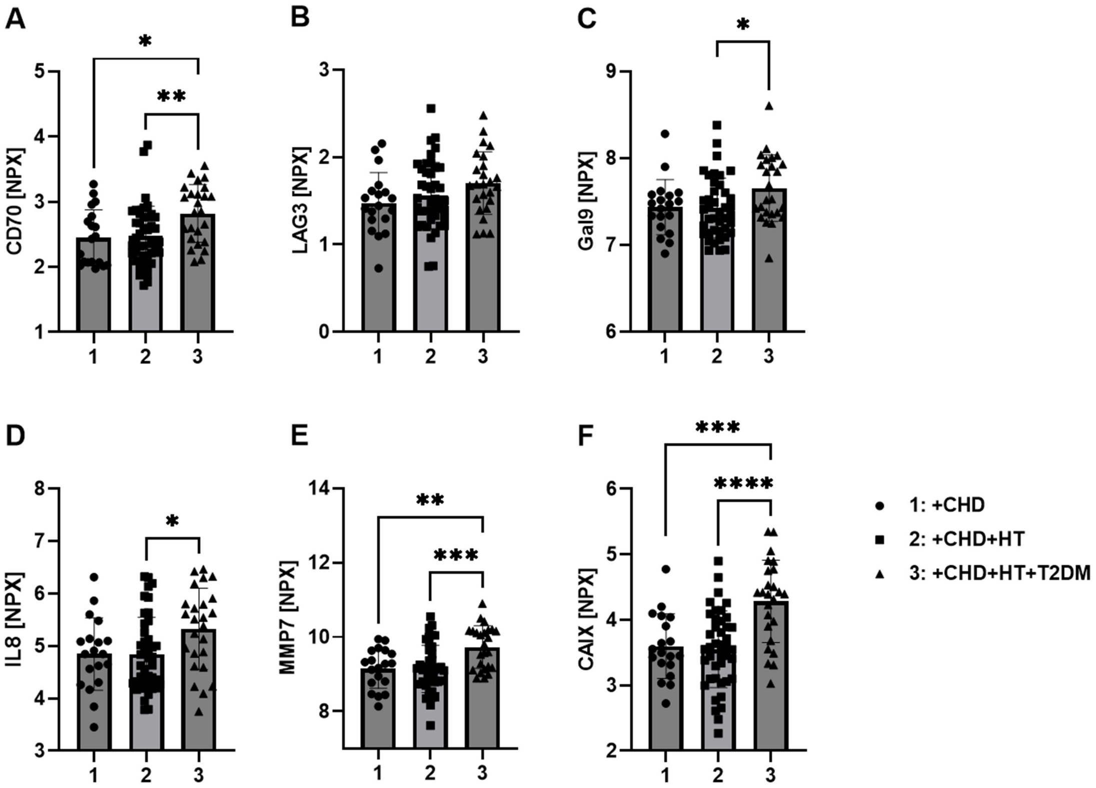


**Supplemental Figure S1: CD40L-CD40-TRAF cascade markers and selected markers of inflammation are increased (two by minor trend) in sera from CHD patients with hypertension plus diabetes as co-morbidity.** Olink analysis in sera from CHD patients with either hypertension or hypertension and diabetes was performed. The following six increased targets in CHD patients with hypertension and diabetes were identified: CD70 (A), LAG3 (B), Gal9 (C), IL8 (D), MMP7 (E), and CAIX (F). One-way ANOVA (with Tukey's test) or Kruskal-Wallis test (with Dunn's correction) were performed. Data are mean±SD CHD n=19, CHD+HT n=42, and CHD+HT+T2DM=25 patients per group. * *p ≤ 0.05,* ** *p ≤ 0.01 and* *** *p ≤ 0.001 .* CHD=coronary heart disease, HT=hypertension, T2DM=type 2 diabetes mellitus, LAG=lymphocyte activation gene, Gal9=galectin 9, IL8=interleukin 8, MMP7=matrix metalloproteinase-7, CAIX=carbonic anhydrase IX.

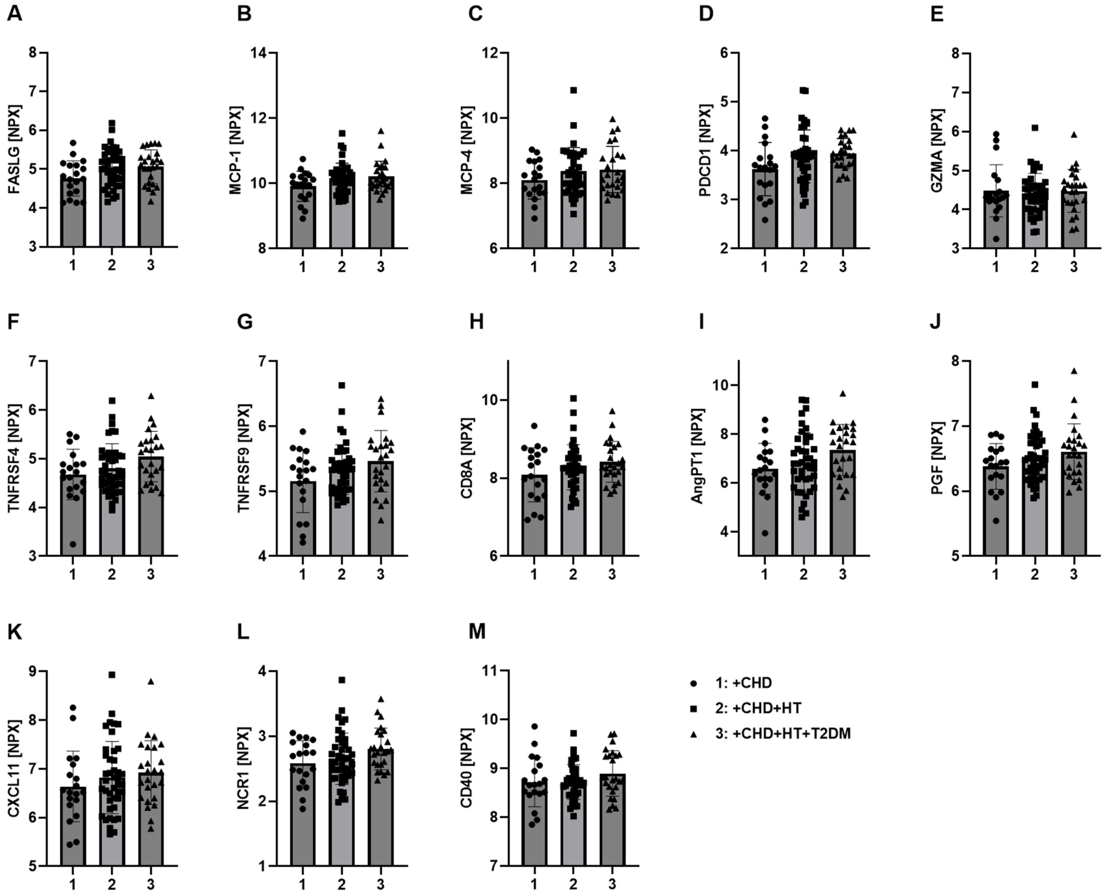


**Supplemental Figure S2: CD40L-CD40-TRAF cascade markers and selected markers of inflammation show a pattern compatible with an increase in sera from CHD patients with hypertension and/or diabetes as co-morbidity.** Olink analysis in sera from CHD patients with either hypertension or hypertension plus diabetes was performed. The following 13 targets showing an increase by minor trend in CHD patients with hypertension and/or diabetes were identified: FASLG (A), MCP-1 (B), MCP-4 (C), PDCD1 (D), GZMA (E), TNFRSF4 (F), TNFRSF9 (G), CD8A (H), AngPT1 (I), PGF (J), CXCL11 (K), NCR1 (L), and CD40 (M). One-way ANOVA (with Tukey's test) or Kruskal-Wallis test (with Dunn's correction) were performed. Data are mean±SD CHD n=19, CHD+HT n=42, and CHD+HT+T2DM=25 patients per group. * *p ≤ 0.05,* ** *p ≤ 0.01 and* *** *p ≤ 0.001* . CHD=coronary heart disease, HT=hypertension, T2DM=type 2 diabetes mellitus, FASLG=Fas ligand, MCP=monocyte chemoattractant protein, PDCD1=programmed cell death protein 1, GZMA=granzyme A, TNFRSF=tumor necrosis factor receptor superfamily, AngPT1=angiopoetin-1, PGF=placental growth factor, CXCL11=C-X-X motif chemokine ligand, NCR1=natural cytotoxicity triggering receptor.

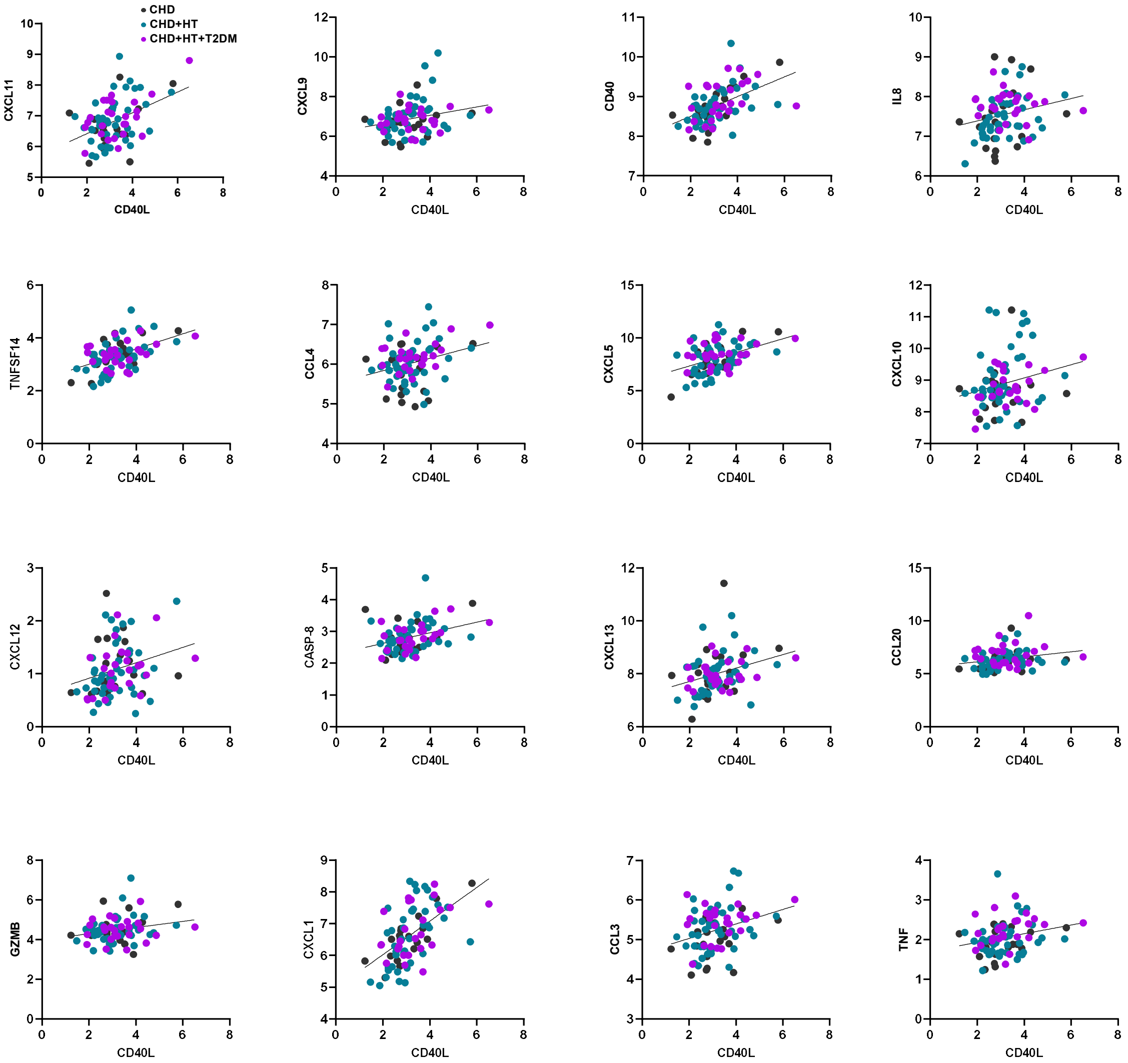


**Supplemental Figure S3: Correlations of CD40L levels with other selected markers of inflammation.** Linear regression analysis revealed significant correlation of CD40L with other markers of inflammation in sera from CHD patients with either hypertension or hypertension and diabetes. Graphical presentation of correlations were selected from **suppl. Table S1** for specific targets that are central to TNF signaling as shown by cluster analysis in **Figure 5** of the main manuscript.

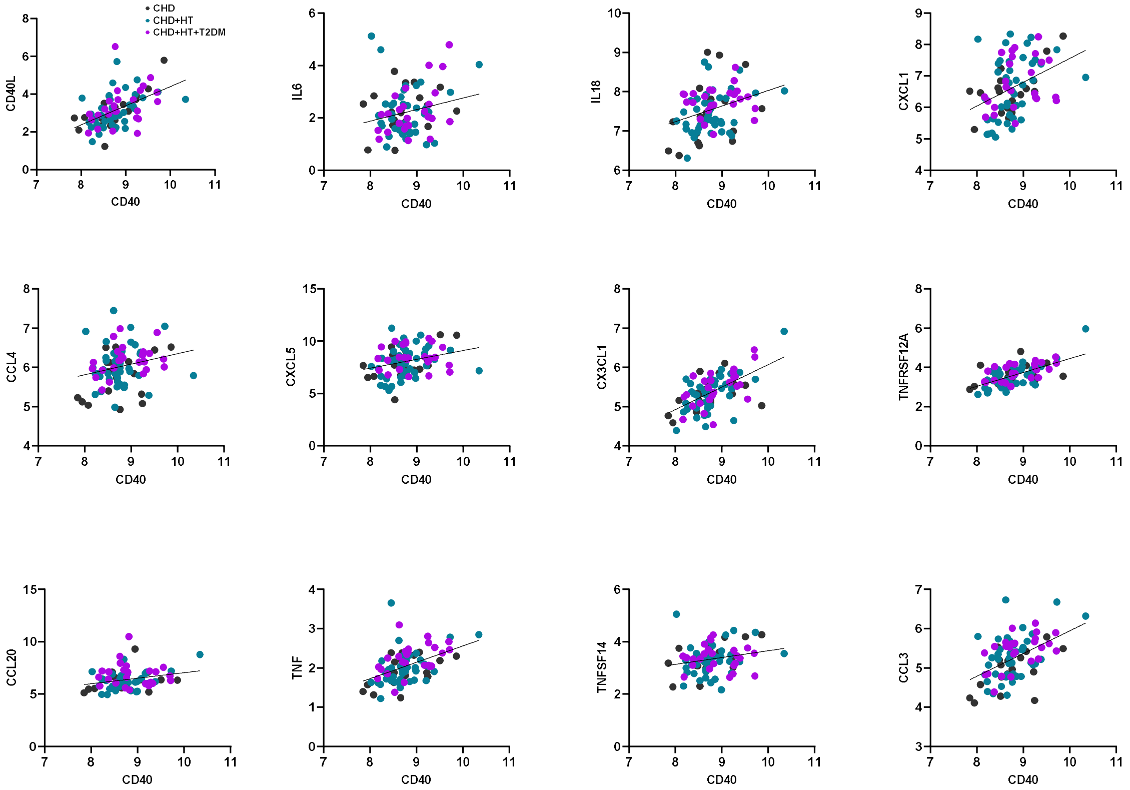


**Supplemental Figure S4: Correlations of CD40 levels with other selected markers of inflammation.** Linear regression analysis revealed significant correlation of CD40 with other markers of inflammation in sera from CHD patients with either hypertension or hypertension and diabetes. Graphical presentation of correlations were selected from **suppl. Table S2** for specific targets that are central to TNF signaling as shown by cluster analysis in **Figure 5** of the main manuscript.

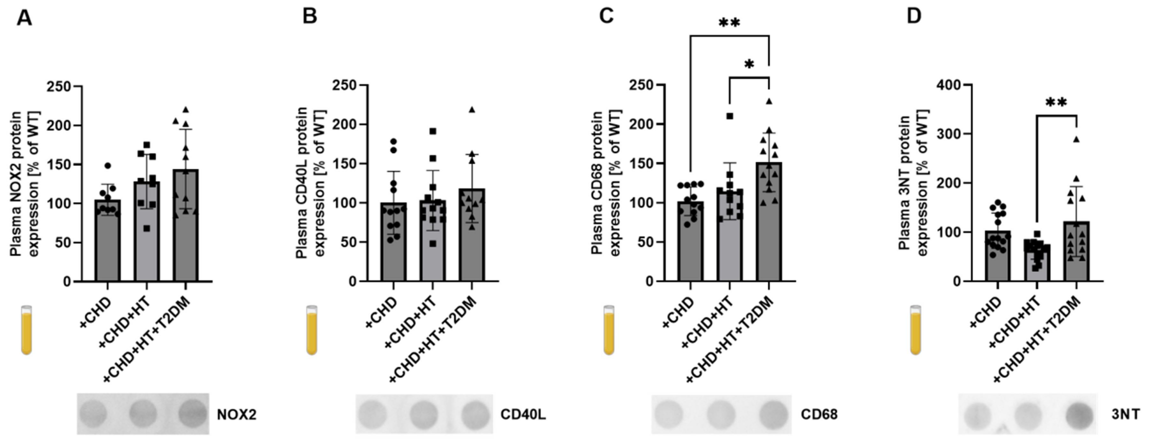


**Supplemental Figure S5: In plasma from CHD patients with either hypertension or hypertension and diabetes, protein expression levels of selected inflammatory markers, are stepwise increased with each additional comorbidity.** Protein expression analysis of NOX2 (A), CD40L (B), CD68 (C), and 3NT (D) was determined by dot blot analysis in the plasma of CHD patients with either hypertension or diabetes or hypertension and diabetes. Original representative blots are shown below. Data are mean±SD CHD n=8-15, CHD+HT n=9-15, and CHD+HT+T2DM n=11-14 patients per group. * *p≤0.05, ** p≤0.01 and *** p≤0.001.* CHD=coronary heart disease, HT=hypertension, T2DM=type 2 diabetes mellitus, NOX2=NADPH oxidase 2, 3NT=3-nitrotyrosine.

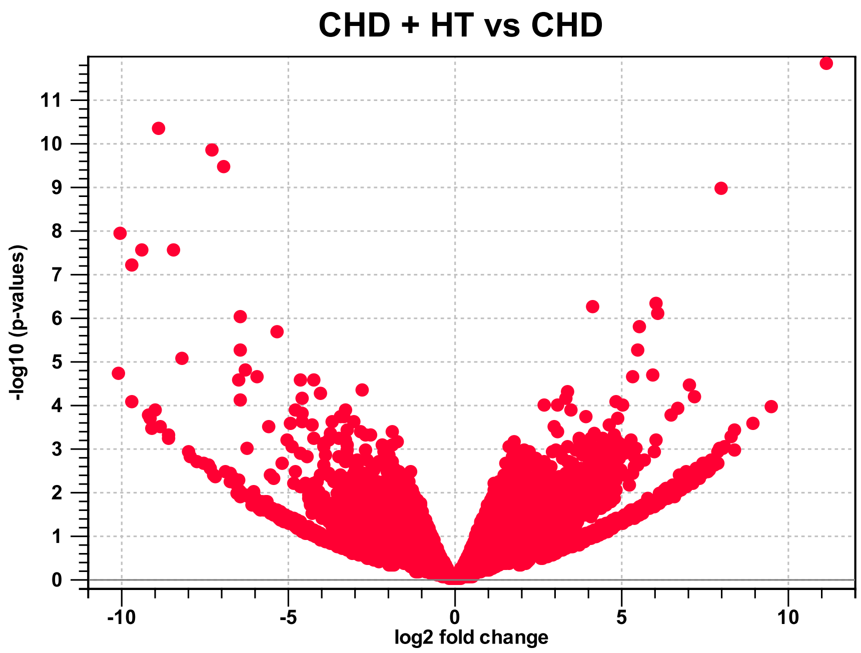
CHD + HT vs CHD
-log10 (p-values)
0
1
2
3
4
5
6
7
8
9
10
11
-10
-5
0
5
10
log2 fold change

**A**

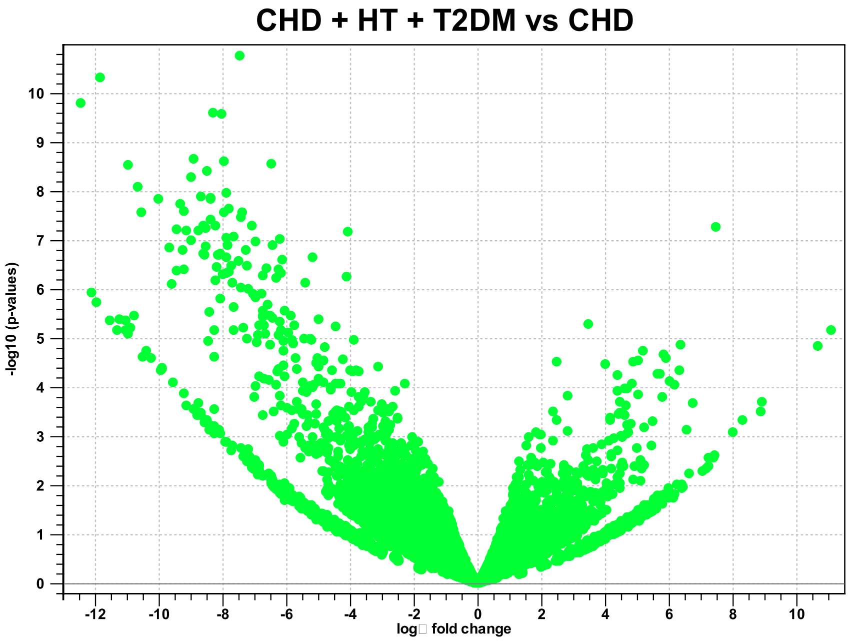
CHD + HT + T2DM vs CHD
-log10 (p-values)
0
1
2
3
4
5
6
7
8
9
10
-12
-10
-8
-6
-4
-2
0
2
4
6
8
10
log□ fold change

**B**

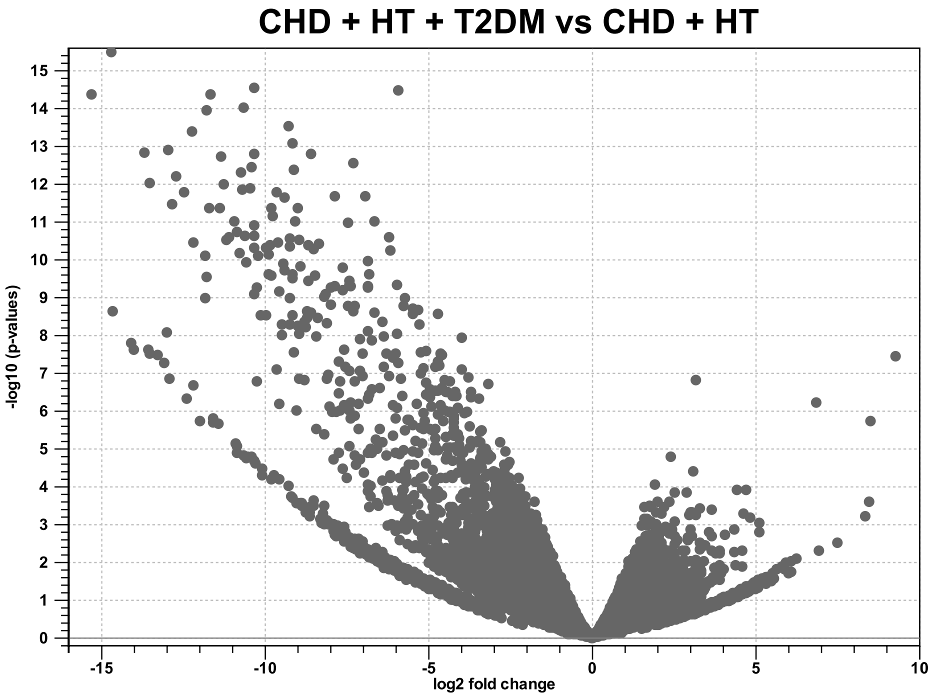


C

**Supplemental Figure S6: Comparison of the gene expression in the aortic samples from (A) CHD+HT versus CHD patients, (B) CHD+HT+T2DM versus CHD patients, and (C) CHD+HT+T2DM versus CHD+HT patients.** Aortic tissue samples from CHD, CHD+HT, or CHD+HT+T2DM patients were analyzed for total mRNA expression using the RNA-Seq method and analyzed with CLC genomics workbench. Shown are the Volcano plots (provided by the CLC genomic workbench) of the comparison (A) CHD+HT versus CHD, (B) CHD+HT+T2DM versus CHD, and (C) CHD+HT+T2DM versus CHD+HT. CHD=coronary heart disease, HT=hypertension, T2DM= type 2 diabetes mellitus.

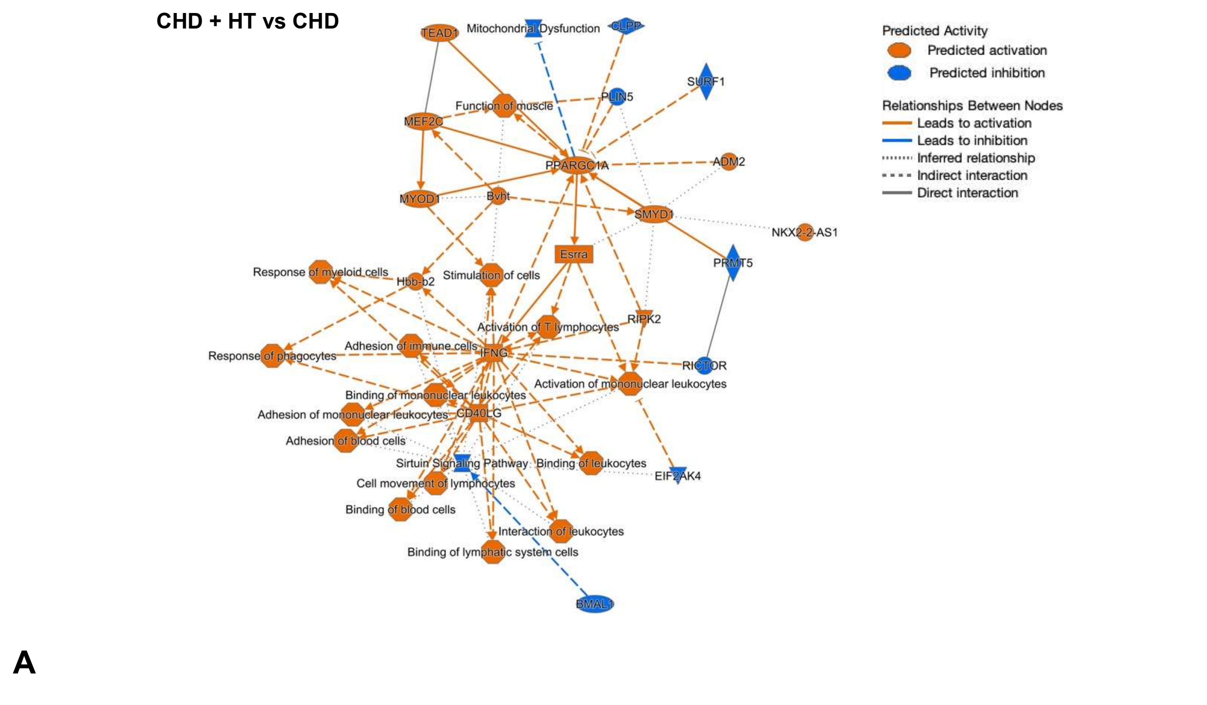
CHD + HT vs CHD
Predicted Activity
Predicted activation
Predicted inhibition
Relationships Between Nodes
Leads to activation
Leads to inhibition
Inferred relationship
Indirect interaction
Direct interaction
TEAD1
Mitochondrial Dysfunction
SURF1
Function of muscle
PLIN5
MEF2C
PPARGC1A
ADM2
MYOD1
Bvht
SMYD1
NKX2-2-AS1
Esrra
PRMT5
Response of myeloid cells
Hbb-b2
Stimulation of cells
RIPK2
Activation of T lymphocytes
Adhesion of immune cells
Response of phagocytes
IFNG
RICTOR
Activation of mononuclear leukocytes
Binding of mononuclear leukocytes
Adhesion of mononuclear leukocytes
CD40LG
Adhesion of blood cells
Sirtuin Signaling Pathway
Binding of leukocytes
EIF2AK4
Cell movement of lymphocytes
Binding of blood cells
Interaction of leukocytes
Binding of lymphatic system cells

**A**

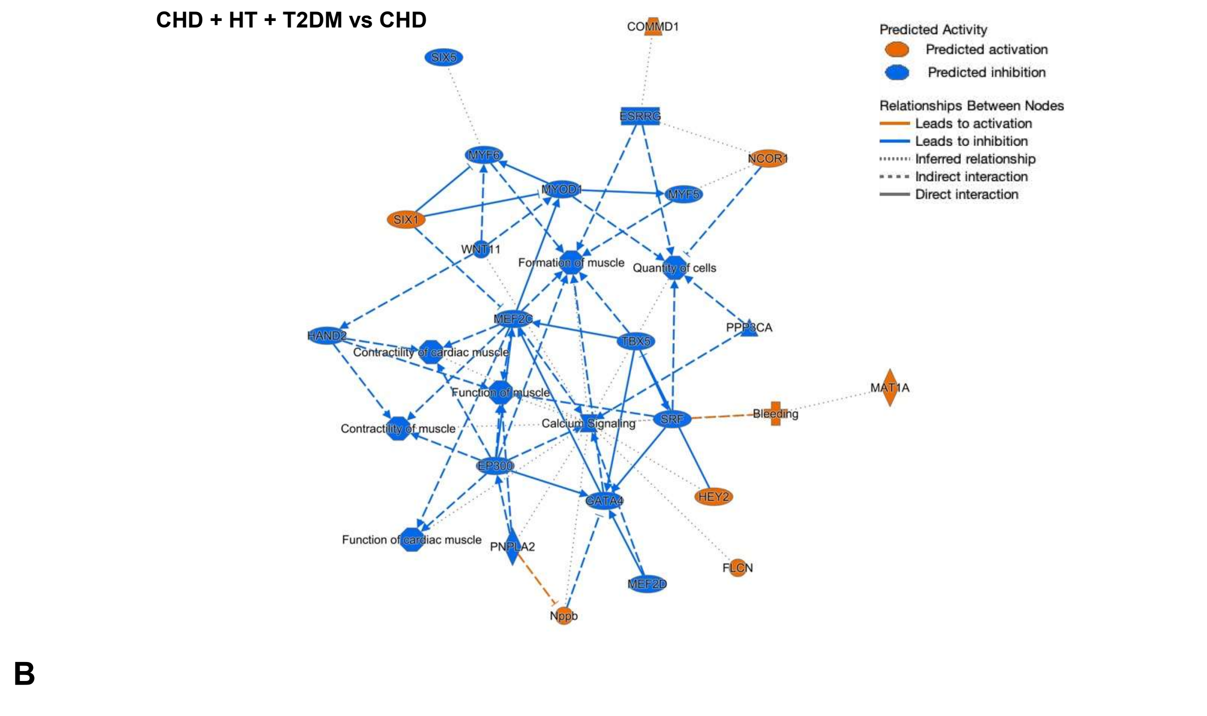
CHD + HT + T2DM vs CHD
COMMD1
Predicted Activity
Predicted activation
Predicted inhibition
Relationships Between Nodes
Leads to activation
Leads to inhibition
Inferred relationship
Indirect interaction
Direct interaction
SIX5
ESRRG
NCOR1
MYF6
MYOD1
MYF5
SIX1
WNT11
Formation of muscle
Quantity of cells
MEF2C
PPP3CA
HAND2
TBX5
Contractility of cardiac muscle
MAT1A
Function of muscle
Bleeding
Contractility of muscle
Calcium Signaling
SRF
EP300
HEY2
GATA4
Function of cardiac muscle
PNPLA2
FLCN
MEF2D
Nppb

**B**

**CHD + HT + T2DM vs CHD + HT**

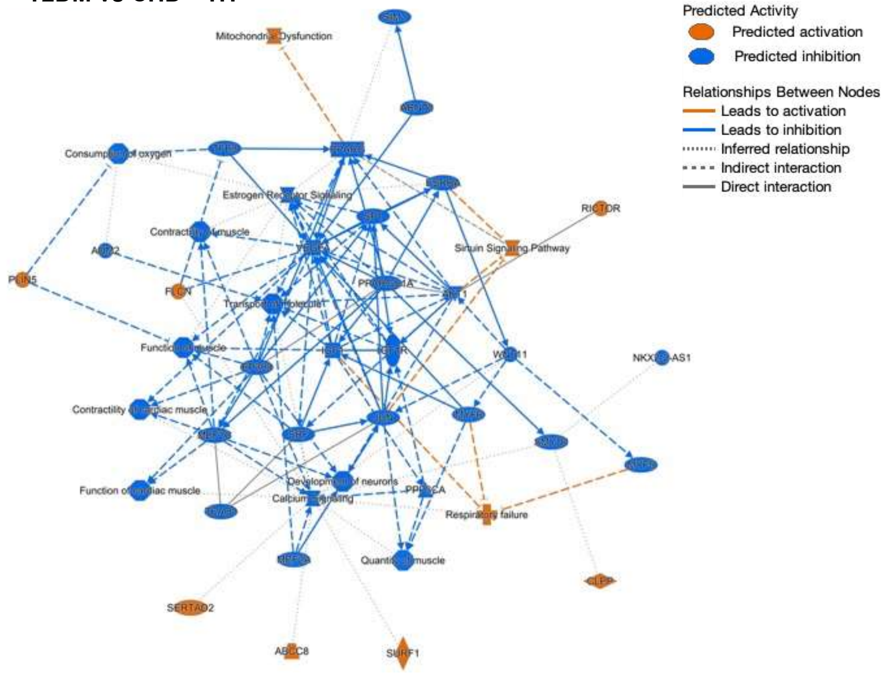


**C**

**Supplemental Figure S7: Graphical summary of the pathways identified by the ingenuity software involved in the regulation of gene expression comparing the aortic samples from (A) CHD+HT versus CHD patients, (B) CHD+HT+T2DM versus CHD patients and (C) CHD+HT+T2DM versus CHD+HT patients.** Aortic tissue samples from CHD, CHD+HT, or CHD+HT+T2DM patients were analyzed for total mRNA expression using the RNA-Seq method and analyzed with CLC genomics workbench. The DEG ($p < 0.05$) lists were imported into the ingenuity software and analyzed. Shown are the summaries of the comparison (A) CHD+HT versus CHD, (B) CHD+HT+T2DM versus CHD, and (C) CHD+HT+T2DM versus CHD+HT. Activated pathways are indicated in red; inhibited pathways are shown in blue. CHD=coronary heart disease, HT=hypertension, T2DM= type 2 diabetes mellitus.

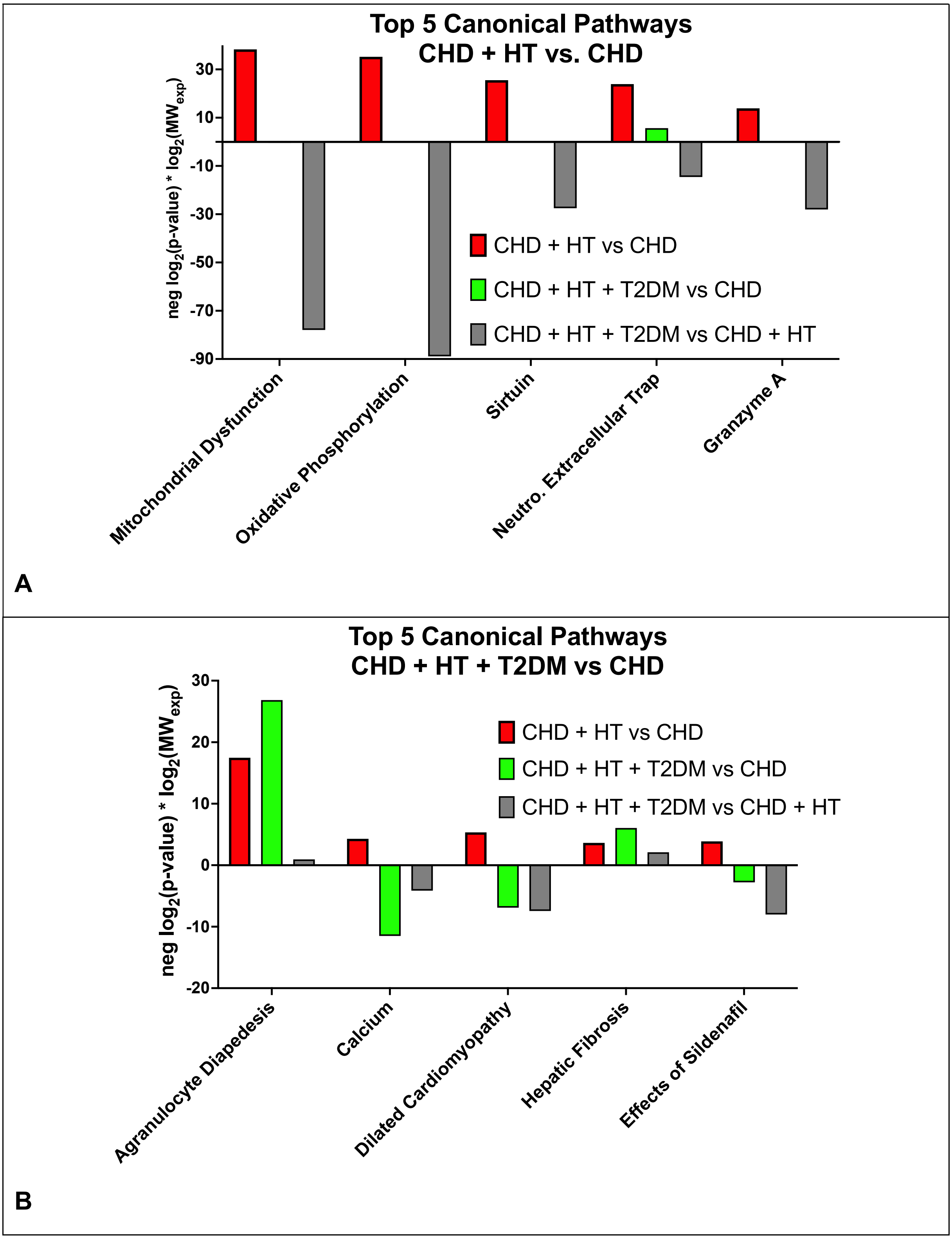
Top 5 Canonical Pathways
CHD + HT vs. CHD
neg log2(p-value) * log2(MWexp)
30
10
-10
-30
-50
-70
-90
CHD + HT vs CHD
CHD + HT + T2DM vs CHD
CHD + HT + T2DM vs CHD + HT
Mitochondrial Dysfunction
Oxidative Phosphorylation
Sirtuin
Neutro. Extracellular Trap
Granzyme A
A
Top 5 Canonical Pathways
CHD + HT + T2DM vs CHD
neg log2(p-value) * log2(MWexp)
30
20
10
0
-10
-20
CHD + HT vs CHD
CHD + HT + T2DM vs CHD
CHD + HT + T2DM vs CHD + HT
Agranulocyte Diapedesis
Calcium
Dilated Cardiomyopathy
Hepatic Fibrosis
Effects of Sildenafil
B

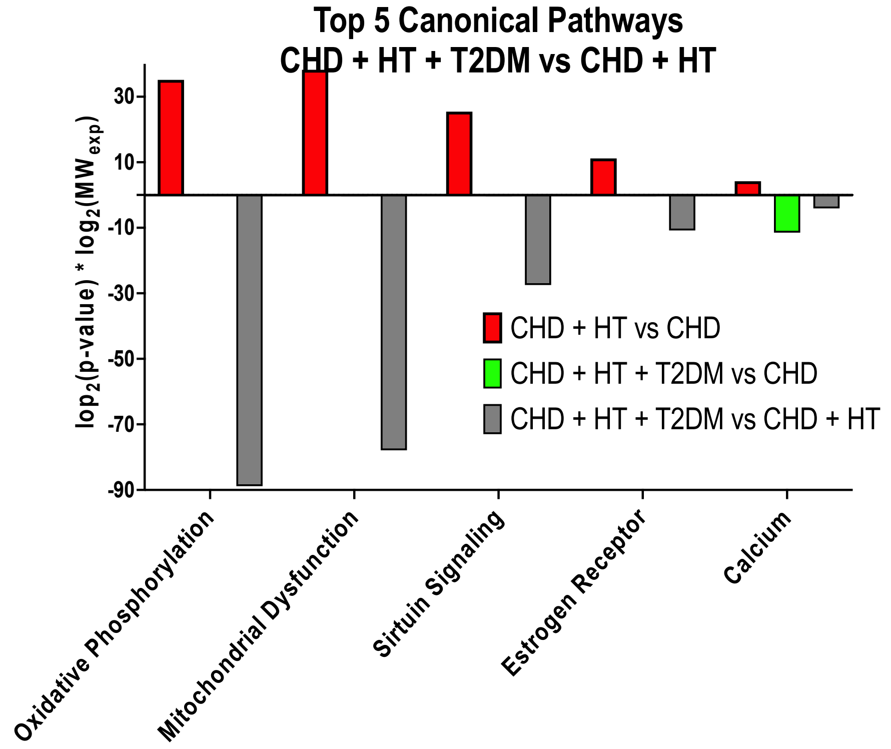


C

**Supplemental Figure S8: Top 5 regulated signal pathways identified by the ingenuity software comparing the gene expression in the aortic samples from (A) CHD+HT versus CHD patients, (B) CHD+HT+T2DM versus CHD patients, and (C) CHD+HT+T2DM versus CHD+HT patients.** Aortic tissue samples from CHD, CHD+HT, or CHD+HT+T2DM patients were analyzed for total mRNA expression using the RNA-Seq method and analyzed with CLC genomics workbench. The DEG lists were imported into the ingenuity software and analyzed for the top 5 regulated canonical signal pathways. To normalize the negative $\log_{2,}$ p-values were multiplicated with the $\log_2$ expression values. Shown are the top 5 pathways for the comparison (A) CHD+HT versus CHD, (B) CHD+HT+T2DM versus CHD, and (C) CHD+HT+T2DM versus CHD+HT. In each case, the values of the other comparisons were included as well. CHD=coronary heart disease, HT=hypertension, T2DM= type 2 diabetes mellitus.

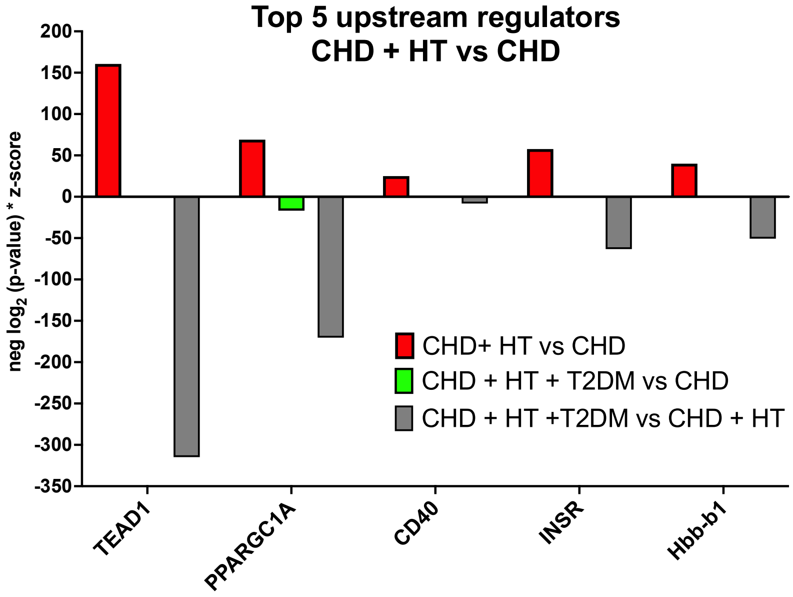
Top 5 upstream regulators
CHD + HT vs CHD
neg log2 (p-value) * z-score
200
150
100
50
0
-50
-100
-150
-200
-250
-300
-350
CHD+ HT vs CHD
CHD + HT + T2DM vs CHD
CHD + HT +T2DM vs CHD + HT
TEAD1
PPARGC1A
CD40
INSR
Hbb-b1

A

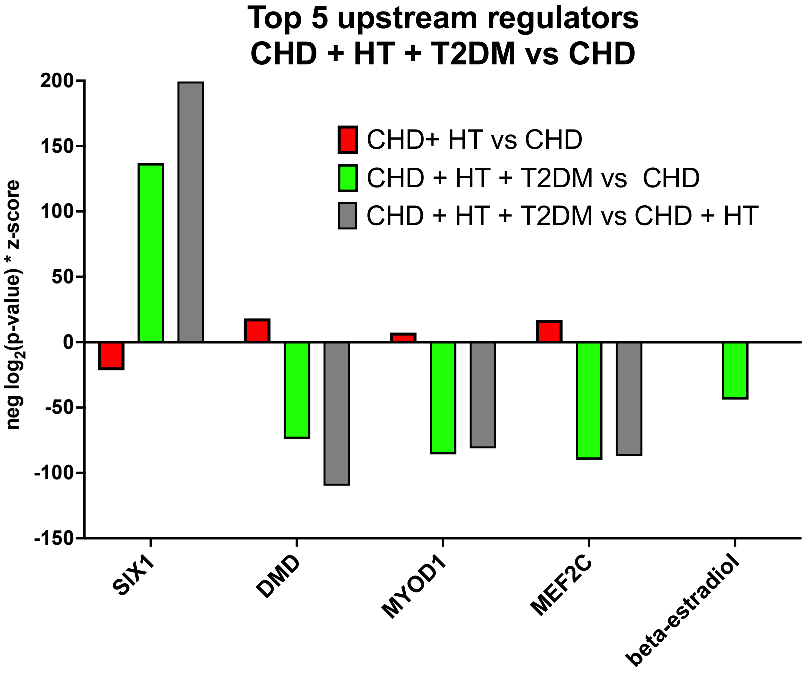
Top 5 upstream regulators
CHD + HT + T2DM vs CHD
neg log2(p-value) * z-score
200
150
100
50
0
-50
-100
-150
CHD+ HT vs CHD
CHD + HT + T2DM vs CHD
CHD + HT + T2DM vs CHD + HT
SIX1
DMD
MYOD1
MEF2C
beta-estradiol

B

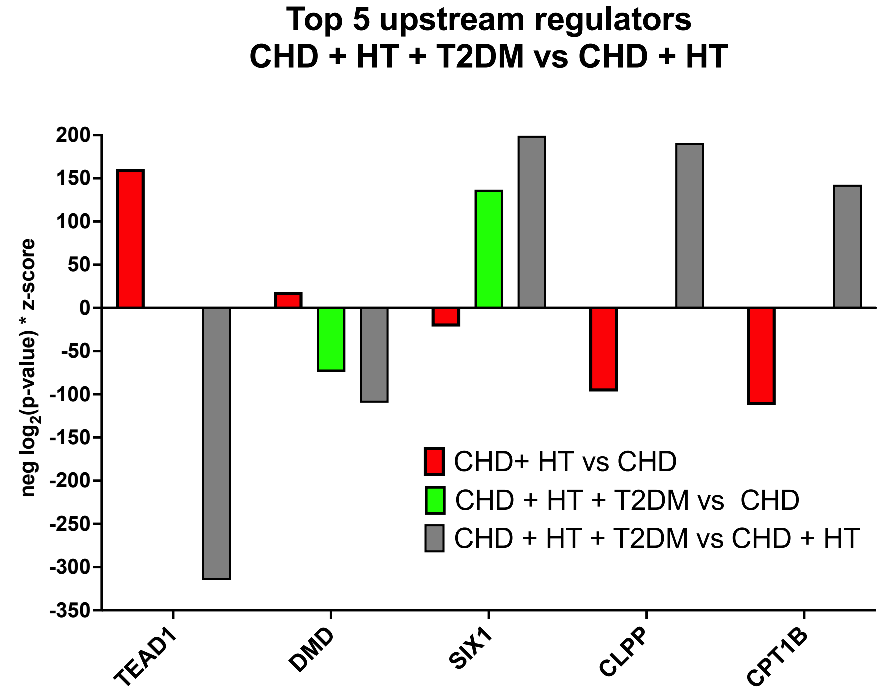


C

**Supplemental Figure S9: Top 5 upstream regulating factors identified by the ingenuity software comparing the gene expression in the aortic samples from (A) CHD+HT versus CHD patients, (B) CHD+HT+T2DM versus CHD patients, and (C) CHD+HT+T2DM versus CHD+HT patients.** Aortic tissue samples from CHD, CHD+HT, or CHD+HT+T2DM patients were analyzed for total mRNA expression using the RNA-Seq method and analyzed with CLC genomics workbench. The DEG lists were imported in the ingenuity software and analyzed for the top 5 upstream regulating factors were identified. To normalize the negative $\log_{2,}$ p-values were multiplicated with the z-score values. Shown are the top 5 upstream regulators for the comparison (A) CHD+HT versus CHD, (B) CHD+HT+T2DM versus CHD and (C) CHD+HT+T2DM versus CHD+HT. In each case, the values of the other comparisons were included as well. CHD=coronary heart disease, HT=hypertension, T2DM= type 2 diabetes mellitus.

## Extended Methods

### 2.3 Measurement of glucose levels in whole blood

The blood glucose was measured in the whole blood of non-fasting animals using the ACCU-CHEK sensor system from Roche Diagnostics.

### 2.4 Non-invasive blood pressure recordings

Before the final non-invasive blood pressure measurement, the mice were trained at least three times to minimize stress reactions and to adapt the mice to the process. Blood pressure was recorded on day 6 after the beginning of the treatment. The final data points reflect a mean value of 5 independent measurements. The accuracy of the tail-cuff method was already demonstrated compared to the radio telemetry measurement [13].

### 2.5 Vascular isometric tension studies

Isometric tension recordings were performed as previously described in reference [46, 51]. Endothelia intact and fat-free isolated aortic rings (thoracic aorta, 3 mm in length) were mounted in organ chambers, and vasodilator responses to acetylcholine (ACh) were assessed. Maximal relaxation was determined after stimulation with 3.3 µM ACh.

### 2.6 Vascular superoxide production

Aortic rings were embedded in Tissue-Tek® O.C.T compound, and cryo-sections (8 µm) were incubated with DHE (1 µM) for 30 min at 37°C. The images were taken using a Zeiss Axiovert 40 CFF microscope and quantified as integrated optical density (IOD) [18, 39].

### 2.7 Superoxide anion detection by HPLC

Heart tissue was incubated in 50 µM DHE for 30 min at 37°C. After weight determination, the heart pieces were homogenized in 300 µl 50 % acetonitrile/50 %

PBS, centrifuged at 20000 g for 20 min, and 50 µl of the supernatant was used for HPLC analysis. The HPLC analysis was performed as described in reference [15]. 2-hydroxyethidium (superoxide-specific product) and ethidium (unspecific ROS-dependent product) concentrations were determined by external and internal standards.

## 2.8 Western Blot Analysis of Protein Expression

Western blot analysis was performed as described previously [11, 14, 47]. The protein was isolated from frozen tissue through homogenization with liquid nitrogen, followed by 60 min incubation in a homogenization buffer containing protease and phosphatase inhibitors on ice with intermittent vortexing. After vortexing and centrifugation (10 min, 10000 g, 4 °C), the protein-rich supernatant was transferred to fresh tubes, and the concentration was measured by Bradford assay. The samples were prepared and diluted to 5 µg/µl in Lämmli buffer. Gel electrophoresis was performed with 30 µg protein on 10% SDS pages and transferred onto nitrocellulose blotting membranes (Amersham Protran, Catalog-No.: 10600002). Afterward, a Ponceau S staining (Sigma Aldrich, Catalog-No.: P7170) was done, and the membranes were blocked with 3 % BSA in TBS-T or PBS-T for 1-2 h. The following primary antibodies were incubated overnight at 4 °C: rabbit polyclonal anti-β-actin antibody (dilution 1:2500, Catalog-No.: A5060) from Sigma Aldrich (St. Louis, MO, USA) as a loading control. Mouse monoclonal anti-thrombospondin-1 (TSP-1) antibody (dilution 1:250, Catalog-No.: sc-59886), mouse monoclonal anti-vascular-cell-adhesion-molecule-1 (VCAM-1) antibody (dilution 1:200, Catalog-No.: sc-13160) and mouse monoclonal anti-endothelin-1 (ET-1) antibody (dilution 1:250, Catalog-No.: sc-517436) were purchased from Santa Cruz (Dallas, TX, USA). Mouse monoclonal anti-endothelial-nitric-oxide-synthase (eNOS) antibody (dilution 1:1000, Catalog-No.: 610297), mouse monoclonal anti-p47phox antibody (dilution 1:250, Catalog-No.: 610354), mouse monoclonal anti-gp91phox antibody (NOX2, dilution 1:500, Catalog-No.: 611414) and mouse monoclonal anti-protease-activated receptor (PAR1) antibody (dilution 1:300, Catalog-No.: 611522) were purchased from BD bioscience (San Jose, CA, USA). Rabbit polyclonal anti-caspase-3 antibody (dilution 1:1000, Catalog-N0.: 9662S), rabbit polyclonal anti-receptor for advanced-glycation-end-products (RAGE) antibody (dilution 1:1000, Catalog-No.: 42544), rabbit

polyclonal anti-p-myristoylated alanine-rich-C-kinase-substrate S152/156 (pMARCKS) antibody (dilution 1:1000, Catalog-No.: 2741) and rabbit monoclonal anti-thioredoxin-interacting protein (TXNIP) antibody (dilution 1:1000, Catalog-No.: 14715) were purchased from Cell Signaling Technology (Danvers, MA, USA). In addition, rabbit monoclonal anti-CD40L antibody (dilution 1:5000, Catalog-No.: NB110-57627) from NovusBio (Littleton, CO, USA) and rabbit monoclonal anti-heme-oxygenase-1 (HO-1) antibody (dilution 1:2000, Catalog-No.: ab68477) from Abcam (Cambridge, UK) was used. After the primary antibody incubation, the membranes were washed three times in TBS-T or PBS-T and incubated with respective anti-mouse or anti-rabbit peroxidase coupled secondary antibody (dilution 1:10000, Catalog-No.: PI-2000 or PI-1000, Vector Lab, Newark, CA, USA) for 1 h at room temperature. Bands were detected using ECL development from Thermo Fisher Scientific (Waltham, MA, USA) (Catalog-No.: 32106 or 34096) and Chemostar M6 imager from Intas Science imaging. Densitometric quantification was performed using GelProAnalyser software (Version 3.0.00.00).

## 2.9 Dot Blot Analysis of Protein Modification and Expression

The renal protein isolation was performed as described before in the Western Blot analysis part, whereas a Bradford assay was directly done from blood plasma or serum. Dot blot analysis was performed according to our previous reports [11, 27, 38]. After two washing steps with 200 µl PBS, 25 µg protein was transferred into each well of the Minifold-I vacuum Dot Blot system (Schleicher & Schuell, 10484138CP) and transferred via vacuum on a nitrocellulose membrane. The membrane was washed twice with PBS, dried for 60 min at 60°C to fix the protein, and incubated in Ponceau S solution [14, 27]. The stain was removed, and the membranes were blocked for 1 h at room temperature with 5 % milk in PBS-T. The membranes were incubated with the following purchased primary antibodies overnight at 4 °C: rabbit monoclonal anti-CD40L antibody (dilution 1:5000, Catalog-No.: NB110-57627) from NovusBio (Littleton, CO, USA), mouse monoclonal anti-gp91phox antibody (NOX2, dilution 1:500, Catalog-No.: 611414) from BD bioscience (San Jose, CA, USA), rabbit polyclonal anti-3-nitrotyrosine (3NT) antibody (dilution 1:2000, Catalog-No.: 06-284) from Merck Millipore (Burlington, MA, USA), goat polyclonal anti-4-hydroxynonenal (4HNE) antibody (dilution 1:1000, Catalog-No.: ab5605) from Merck Millipore

(Burlington, MA, USA), mouse monoclonal anti-CD68 antibody (dilution 1:1000, Catalog-No.: ab31630) from Abcam (Cambridge, UK), rabbit polyclonal anti-IL6 antibody (dilution 1:1000, Catalog-No.: ab6672) from Abcam (Cambridge, UK), and rabbit polyclonal anti-malondialdehyde (MDA) antibody (dilution 1:1000, Catalog-No.: 442730) from Merck Millipore. Afterward, the membranes were treated in the same way as the Western Blot membranes. Secondary antibodies were the respective peroxidase coupled anti-mouse or anti-rabbit and anti-goat (dilution 1:5000, Catalog-No.: sc-2354) from Santa Cruz (Dallas, TX, USA).

### 2.10 Targeted proteomics

To address the role of co-morbidities arterial hypertension and diabetes on the severity of coronary artery disease and thromboinflammation, the 92 CVD-related human protein biomarkers of the Olink T96 IMMUNO-ONCOLOGY panel were measured using the Proximity Extension Assay (PEA) technology (Olink Biosciences, Uppsala, Sweden), as described elsewhere [3, 19, 33]. In brief, once-thawed ethylenediaminetetraacetic acid (EDTA)-blood plasma was used for analysis. For each target antigen, the affinity-based PEA technique uses a pair of antibodies linked to unique, partially complementary single-stranded DNA oligonucleotides. After the simultaneous binding of both antibodies to an antigen molecule, close proximity allows for the formation of a PCR target sequence by hybridization. After unspecific preamplification, amplicons were quantified by qPCR using protein-specific primer pairs. The resulting $C_t$-value of each protein (Fluidigm Real-Time PCR Analysis Software, Version 4.3.1, San Francisco, USA) was transformed to normalized protein expression (NPX) units using software from the manufacturer (Olink® NPX Manager, Version 1.1.4.0, Uppsala, Sweden). NPX units represent relative quantifications of protein concentrations on a log2-scale (i.e., an increase by 1 NPX represents a duplication of protein concentration).

### 2.11 Bioinformatical analysis of proteomic data

Samples were grouped, and differential expression analysis was performed using the Perseus software suite. Briefly, differential expression was determined by applying the Student's t-test using a cut-off of $p<0.05$. Proteins that were differentially

expressed in at least one group were retained. To visualize expression changes across the groups, we determined the mean value, Z-normalised, and proteins were clustered using Euclidean distance. Network analysis and data representation were performed using Cytoscape. Differentially expressed proteins were mapped onto a network generated by String. Edges represent interactions based on genetic, experimental, biochemical, and text-mining data using a threshold of >0.4.

**2.12 RNA-sequencing**

RNA sequencing was carried out at Novogene Bioinformatics Technology Co., Ltd., in Cambridge (UK), and in analogy to our previous studies [27, 38], except that we used the human library for the present analysis. According to the manufacturer's protocol, aortic mRNA was isolated using the RNeasy Mini kit (Qiagen, Hilden, Germany). RNA sample quality control, mRNA library preparation (poly A enrichment), NovaSeq PE150 sequencing (9 G raw data per sample), and data quality control were performed by Novogene. Data analysis was performed using CLC genomic workbench (22.0.2) and IPA (both from Qiagen, Hilden, Germany). The data (fastq.gz) were imported in the CLC data format (.clc). from the Novogen sequencing results report (RAW data). Then the reads were trimmed using the parameter provided by the manufacturer (quality score: 0.05; the maximum number of ambiguities: 2). These trimmed RNA-Seq data were mapped to the human genome (Homo_sapiens_hg38-2022-11-25-22-33, ENSEMBL) using the following parameters (Mismatch cost: 3; Insertion cost: 3; Deletion cost: 3; Length fraction: 0.9; Similarity fraction: 0.9; Maximum number of hits for a read: 10). The rpkm data were used for calculation of the fold enhancement of the mRNA expression. The statistical comparisons provided by CLC genomics workbench were uploaded to the IPA server to analyze relevant changes in signal pathways (upload p < 0,05; analysis p < 0,05). Volcano plots to envisage the number of changed genes by the three different therapeutic interventions created by the CLC genomic workbench. Canonical pathways analyses to envisage the biochemical processes that are changed by the two co-morbidities arterial hypertension and diabetes in CHD patients were performed with IPA (**suppl. Figures S7 and S8**).

## Extended Discussion of Human Protein Data

In our proteomic analysis of CHD patients' plasma, we identified some step-wise increased targets in association with the number of their comorbidities (CHD<CHD+HT<CHD+HT+T2DM). Cluster analysis identified TNFα as a central player. TNFRSF9 (CD137 / 4-1BB) and TNFRSF4 (CD134 / ox40) belong to the TNF receptor superfamily, like CD40 (TNFRSF5). Both proteins are present on the surface of activated T cells. Like CD40, TNFRSF9 and TNFRSF4 can directly interact with TRAF2 and promote NF-κB activation [21, 24, 34]. Further, TNFRSF9 expression is enhanced by pro-inflammatory stimuli via CD40 or IL12 and functions as a T cell co-stimulator [16]. CD27 belongs to the TRAF interacting receptors and mediates T-T and T-B cell interactions. Like other co-stimulatory receptors, CD27 functions similarly and is involved in NF-κB activation via TRAF2/5 signaling [1]. Inflammatory cytokines like TNFα also belong to the group of TRAF interacting proteins, and we observed a significantly increased protein expression in our CHD patients. Besides TRAF2-mediated signaling, TNFα can induce apoptosis via caspase8/caspase3 signaling [6]. In addition, TNFRSF21 (or DR6), significantly increased in the patient's plasma, contains a death domain and induces apoptosis via NF-κB signaling. Since TNFRSF21 also belongs to the family of TNF receptors superfamily, it may also play a role in inflammation and regulation of the immune system [41].

Gal1 is another significantly upregulated protein that can induce apoptosis, especially in T cells. Among others, the upregulation of Gal1 expression and secretion is upregulated in activated B-cells via CD40L (on T-cells) and CD40 (on B-cells) interaction, which could lead to apoptosis in T-cells and increased cytokine production in B-cells. Gal1 and, therefore, indirect CD40L-CD40 mediated signaling seems to be an important modulator for B-cell-regulated T-cell function and survival [54]. ICOSLG, which is also step-wise increased in our proteomic studies, is expressed on germinal center B-cells and interacts with its receptor ICOS (on T follicular helper cells), which mediates calcium flux. The altered calcium flux in T follicular helper cells leads to CD40L externalization, which triggers CD40 on germinal center B cells and leads to ICOSLG expression. The positive feedback mechanism of upregulated ICOSLG by CD40 signaling is important in bone marrow plasma cell development and T follicular helper cell-controlled, long-lived humoral immunity [32]. Recently, through RNA-seq analysis in human tonsils, a new

population of CCL4/CCL3 chemokine-expressing B cells was described. This cell population's activation and chemokine expression are accompanied by CD40 co-stimulation [12], which indicates the significant increase in CVD patients' plasma of CCL4 and CCL3 is probably mediated by CD40L-CD40 signaling. For example, CD40 signaling in macrophages is activated after interaction with T cell-expressed CD40L, leading to p38 MAPK activation that induces the pro-inflammatory cytokine IL12 expression [36]. CD5 is upregulated after strong T and B cell activation. On CD5+ lymphocytes, the IL10 secretion is mediated through CD40 signaling [52]. CD244 and KLRD1 are involved in NK (natural killer) cell function and NK cell-mediated immunity [2]. It is not clear to what extent CD40L-CD40 signaling is involved in their signaling. Also, the significantly increased CD68 expression in our human plasma is linked to the CD40L-CD40 signaling pathway. Indeed, enhanced CD40 expression co-stimulates CD68 expression, indicating an involvement of monocytes and macrophages in inflammation [10].

## Extended Discussion of Human RNA-seq Data

The summary of IPA analysis of the comparison of CHD+HT vs. CHD showed the upregulation of pro-inflammatory and pro-oxidative gene expression (see **suppl. Fig. S7A**). Also, the top 5 regulated canonical pathways (see **suppl. Fig. S8A**) showed activation of mitochondrial dysfunction, oxidative phosphorylation, enhanced neutrophil extracellular trap generation, and enhanced granzyme A activation. These pathways are important for pro-inflammatory and pro-oxidative mechanisms leading to cardiovascular diseases. The IPA-analysis of the top 5 upstream regulators (**suppl. Fig S9A**) revealed the importance of the transcription factor TEAD1, the transcriptional coactivator PPARGC1A, CD40 (receptor of CD40L), the insulin receptor INSR, and the beta adult major chain of hemoglobin Hbb-b1 (human HBB) for the observed gene expression changes. Inhibition of the Yes-TEAD interaction markedly inhibited the Angiotensin-II-mediated vascular remodeling in a rat hypertension model [31]. The peroxisome proliferator-activated receptor gamma coactivator-1 alpha (PPARGC1A) has been linked to energy metabolism, which may affect heart function. PPARGC1A is dysregulated in heart failure [40, 45] and plays a role in endothelial regulation [9] and atherosclerotic lesions generation [22]. The IPA-noted inclusion of CD40 in the top 5 upstream regulators of the expression changes

seen in the CHD HT vs. the CHD group matched perfectly with our animal data of this study and already published data [46]. Also, the presence of the insulin receptor INSR in the 5 top upstream regulators is in good accordance with the published literature [37]. Only the presence of the Hbb-b1 gene (human HBB) in the top 5 upstream regulators does not fit well in the literature. However, several data suggest sickle cell disease (SCD; mutation in the β-globin gene HBB) can be viewed as pan-vasculopathy associated with multiple mechanisms [50].

Interestingly, **suppl. Fig. S8A** showed that the effects of all these upstream regulators are markedly reduced in comparing CHD+HT+T2DM vs. CHD+HT. This unexpected result likely correlates to the drugs used in the CHD+HT+T2DM group. All patients in this group received glucose-lowering drugs (insulin, metformin). High glucose is well noticed to be a pro-inflammatory and pro-oxidative factor [8]. Therefore, it is reasonable that drug-mediated blood-glucose-lowering resulted in the reversion of glucose-induced gene expression regulation. In addition, there also anti-oxidative effects described for metformin (via AMPK activation and inhibition of mTOR pathways [4, 17]) and insulin (via NO [48]) that may explain the reversion of gene expression in CHD HT+T2DM vs. CHD+HT patients.

The summaries of the IPA analyses of the comparisons CHD+HT+T2DM vs. CHD and CHD+HT+T2DM vs. CHD+HT (**suppl. Fig. S7B and C**) both showed mainly inhibitory effects of the inclusion of T2DM in the disease complex on gene expressional regulation. This is also primarily true for the top 5 canonical pathways identified by IPA for these comparisons (**suppl. Fig. S8B and C**). Also, most of the top 5 upstream regulators (**suppl. Fig S9B and C**) identified for the comparisons CHD+HT+T2DM vs. CHD and CHD+HT+T2DM vs. CHD+HT are downregulated (DMD, MYOD1, MEFC1, and beta-estradiol for CHD+HT+T2D; M vs. CHD; **suppl. Fig. S9B**; TEAD1 and DMD for CHD+HT+T2DM vs. CHD+HT; **suppl. Fig. S9B**).

In both comparisons, the IPA program identified T2DM-induced activation of the pathway involving the transcription factor Sine Oculis Homeobox Homolog 1 (SIX1). SIX1 is a homeobox transcription factor important for development and differentiation. SIX1 regulates the expression of different proteins involved in organ development and cancer [43]. SIX1 is upregulated in a murine asthma model, and silencing of SIX1 resulted in reduced NF-κB signaling as SIX1 has been identified as an integral component of the non-canonical NF-κB signaling cascade [53]. No data

about the specific involvement of SIX1 in CHD, hypertension, or T2DM have been published. Also, in the comparisons of CHD+HT+T2DM vs. CHD and CHD+HT+T2DM vs. CHD+HT, the IPA-analysis showed reduced effects of the cytoskeletal/membrane protein dystrophin (DMD). It is well known that mutations in dystrophin can result in familial forms of dilated cardiomyopathy [7]. In viral myocarditis, the transcription factor STAT3 blocks the dilated cardiac phenotype by maintaining membrane integrity resulting from STAT3-regulated dystrophin expression [28].

In comparing CHD+HT+T2DM vs. CHD (**suppl. Fig. S9B**), IPA indicated reduced effects of both transcription factors MYOD1 and MEF2C. In hypertensive rats, the cardiac remodeling induced by the lncRNA MALAT1 was related to the inhibition of MyoD expression [30]. The transcription factor MEF2C is mainly involved in cell proliferation, migration, and cellular homeostasis in cardiovascular systems, which play important roles in modulating cell phenotype [42]. MEF2C has also been described as an important regulator of smooth muscle differentiation, and dysregulation of MEF2C has been linked to hypertension-related vascular changes [44]. The IPA analysis also indicated reduced effects of beta-estradiol in this group. Estrogen treatment is commonly believed to reduce/block the development of atherosclerosis. Estrogen has been shown to enhance endothelial NOS expression and thereby enhance bioactive NO [26], cause vasodilation, suppress arterial collagen secretion and extracellular matrix deposition, act as calcium antagonist properties, diminish calcification, and decrease the secretion of inflammatory cytokines (reviewed in [20]). However, studies with estrogens in human cardiovascular disease have resulted in conflicting results [5, 35].

In the comparison of CHD+HT+T2DM vs. CHD+HT (**suppl. Fig. S9C**), IPA indicated enhanced effects of the serine protease caseinolytic protease proteolytic subunit (CLPP) and the fatty acid oxidation enzyme carnitine palmitoyl transferase 1B (CPT1B). In rats treated with a high-fat diet for 18 weeks, systemic insulin resistance was induced, and the CLPP expression in slow-twitch muscle was enhanced compared to the control animals [29]. As stated by the authors of this study, insulin sensitivity in skeletal muscle is related to mitochondrial homeostasis. As CLPP is a marker protein for the mitochondrial unfolded protein response (UPRmt), the enhancement of CLPP may be a mechanism to counteract mitochondrial dysfunction. In a cardiac hypertrophy mouse model, overexpression of CPT1B could reverse

cardiomyocyte hypertrophy induced by the transcribed ultra-conserved region us.323 [49]. Gastric bypass surgery of T2DM patients improved whole-body insulin sensitivity and enhanced expression of fatty acid oxidation enzymes like CPT1B [23]. Finally, CPTB1 deficiency protected mice from diet-induced Insulin resistance [25]. Therefore, CPT1B seems to be involved in T2DM pathogenesis.

## Extended references


1. Akiba H, Nakano H, Nishinaka S, Shindo M, Kobata T, Atsuta M, Morimoto C, Ware CF, Malinin NL, Wallach D, Yagita H, Okumura K (1998) CD27, a member of the tumor necrosis factor receptor superfamily, activates NF-kappaB and stress-activated protein kinase/c-Jun N-terminal kinase via TRAF2, TRAF5, and NF-kappaB-inducing kinase. J Biol Chem 273:13353-13358 doi:10.1074/jbc.273.21.13353
2. Almansa R, Heredia-Rodriguez M, Gomez-Sanchez E, Andaluz-Ojeda D, Iglesias V, Rico L, Ortega A, Gomez-Pesquera E, Liu P, Aragon M, Eiros JM, Jimenez-Sousa MA, Resino S, Gomez-Herreras I, Bermejo-Martin JF, Tamayo E (2015) Transcriptomic correlates of organ failure extent in sepsis. J Infect 70:445-456 doi:10.1016/j.jinf.2014.12.010
3. Assarsson E, Lundberg M, Holmquist G, Bjorkesten J, Thorsen SB, Ekman D, Eriksson A, Rennel Dickens E, Ohlsson S, Edfeldt G, Andersson AC, Lindstedt P, Stenvang J, Gullberg M, Fredriksson S (2014) Homogenous 96-plex PEA immunoassay exhibiting high sensitivity, specificity, and excellent scalability. PLoS One 9:e95192 doi:10.1371/journal.pone.0095192
4. Bharath LP, Nikolajczyk BS (2021) The intersection of metformin and inflammation. Am J Physiol Cell Physiol 320:C873-C879 doi:10.1152/ajpcell.00604.2020
5. Boardman HM, Hartley L, Eisinga A, Main C, Roque i Figuls M, Bonfill Cosp X, Gabriel Sanchez R, Knight B (2015) Hormone therapy for preventing cardiovascular disease in post-menopausal women. Cochrane Database Syst Rev 2015:CD002229 doi:10.1002/14651858.CD002229.pub4
6. Borghi A, Verstrepen L, Beyaert R (2016) TRAF2 multitasking in TNF receptor-induced signaling to NF-kappaB, MAP kinases and cell death. Biochem Pharmacol 116:1-10 doi:10.1016/j.bcp.2016.03.009
7. Chien KR (1999) Stress pathways and heart failure. Cell 98:555-558 doi:10.1016/s0092-8674(00)80043-4
8. Christou MA, Christou PA, Kyriakopoulos C, Christou GA, Tigas S (2023) Effects of Hypoglycemia on Cardiovascular Function in Patients with Diabetes. Int J Mol Sci 24 doi:10.3390/ijms24119357
9. Craige SM, Kroller-Schon S, Li C, Kant S, Cai S, Chen K, Contractor MM, Pei Y, Schulz E, Keaney JF, Jr. (2016) PGC-1alpha dictates endothelial function through regulation of eNOS expression. Sci Rep 6:38210 doi:10.1038/srep38210
10. de Ramon L, Ripoll E, Merino A, Lucia M, Aran JM, Perez-Rentero S, Lloberas N, Cruzado JM, Grinyo JM, Torras J (2015) CD154-CD40 T-cell co-stimulation pathway is a key mechanism in kidney ischemia-reperfusion injury. Kidney Int 88:538-549 doi:10.1038/ki.2015.146
11. Eckrich J, Frenis K, Rodriguez-Blanco G, Ruan Y, Jiang S, Bayo Jimenez MT, Kuntic M, Oelze M, Hahad O, Li H, Gericke A, Steven S, Strieth S, von Kriegsheim A, Munzel T, Ernst BP, Daiber A (2021) Aircraft noise exposure drives the activation of white blood cells and induces microvascular dysfunction in mice. Redox Biol 46:102063 doi:10.1016/j.redox.2021.102063
12. Espinoza DA, Le Coz C, Cruz Cabrera E, Romberg N, Bar-Or A, Li R (2023) Distinct stage-specific transcriptional states of B cells derived from human tonsillar tissue. JCI insight 8 doi:10.1172/jci.insight.155199
13. Feng M, Whitesall S, Zhang Y, Beibel M, D'Alecy L, DiPetrillo K (2008) Validation of volume-pressure recording tail-cuff blood pressure measurements. Am J Hypertens 21:1288-1291 doi:10.1038/ajh.2008.301
14. Frenis K, Helmstadter J, Ruan Y, Schramm E, Kalinovic S, Kroller-Schon S, Bayo Jimenez MT, Hahad O, Oelze M, Jiang S, Wenzel P, Sommer CJ, Frauenknecht KBM, Waisman A, Gericke A, Daiber A, Munzel T, Steven S (2021) Ablation of lysozyme M-positive cells prevents aircraft noise-induced vascular damage without improving cerebral side effects. Basic Res Cardiol 116:31 doi:10.1007/s00395-021-00869-5
15. Frenis K, Kalinovic S, Ernst BP, Kvandova M, Al Zuabi A, Kuntic M, Oelze M, Stamm P, Bayo Jimenez MT, Kij A, Keppeler K, Klein V, Strohm L, Ubbens H, Daub S, Hahad O, Kroller-Schon S, Schmeisser MJ, Chlopicki S, Eckrich J, Strieth S, Daiber A, Steven S, Munzel T (2021) Long-

Term Effects of Aircraft Noise Exposure on Vascular Oxidative Stress, Endothelial Function and Blood Pressure: No Evidence for Adaptation or Tolerance Development. Front Mol Biosci 8:814921 doi:10.3389/fmolb.2021.814921
16. Futagawa T, Akiba H, Kodama T, Takeda K, Hosoda Y, Yagita H, Okumura K (2002) Expression and function of 4-1BB and 4-1BB ligand on murine dendritic cells. Int Immunol 14:275-286 doi:10.1093/intimm/14.3.275
17. Goel S, Singh R, Singh V, Singh H, Kumari P, Chopra H, Sharma R, Nepovimova E, Valis M, Kuca K, Emran TB (2022) Metformin: Activation of 5' AMP-activated protein kinase and its emerging potential beyond anti-hyperglycemic action. Front Genet 13:1022739 doi:10.3389/fgene.2022.1022739
18. Helmstadter J, Frenis K, Filippou K, Grill A, Dib M, Kalinovic S, Pawelke F, Kus K, Kroller-Schon S, Oelze M, Chlopicki S, Schuppan D, Wenzel P, Ruf W, Drucker DJ, Munzel T, Daiber A, Steven S (2020) Endothelial GLP-1 (Glucagon-Like Peptide-1) Receptor Mediates Cardiovascular Protection by Liraglutide In Mice With Experimental Arterial Hypertension. Arterioscler Thromb Vasc Biol 40:145-158 doi:10.1161/atv.0000615456.97862.30
19. Herzog J, Schmidt FP, Hahad O, Mahmoudpour SH, Mangold AK, Garcia Andreo P, Prochaska J, Koeck T, Wild PS, Sorensen M, Daiber A, Munzel T (2019) Acute exposure to nocturnal train noise induces endothelial dysfunction and pro-thromboinflammatory changes of the plasma proteome in healthy subjects. Basic Res Cardiol 114:46 doi:10.1007/s00395-019-0753-y
20. Hsu SP, Lee WS (2020) Effects of female sex hormones on the development of atherosclerosis. Chin J Physiol 63:256-262 doi:10.4103/CJP.CJP_69_20
21. Jang IK, Lee ZH, Kim YJ, Kim SH, Kwon BS (1998) Human 4-1BB (CD137) signals are mediated by TRAF2 and activate nuclear factor-kappa B. Biochem Biophys Res Commun 242:613-620 doi:10.1006/bbrc.1997.8016
22. Kadlec AO, Chabowski DS, Ait-Aissa K, Gutterman DD (2016) Role of PGC-1alpha in Vascular Regulation: Implications for Atherosclerosis. Arterioscler Thromb Vasc Biol 36:1467-1474 doi:10.1161/ATVBAHA.116.307123
23. Katsogiannos P, Kamble PG, Boersma GJ, Karlsson FA, Lundkvist P, Sundbom M, Pereira MJ, Eriksson JW (2019) Early Changes in Adipose Tissue Morphology, Gene Expression, and Metabolism After RYGB in Patients With Obesity and T2D. J Clin Endocrinol Metab 104:2601-2613 doi:10.1210/jc.2018-02165
24. Kawamata S, Hori T, Imura A, Takaori-Kondo A, Uchiyama T (1998) Activation of OX40 signal transduction pathways leads to tumor necrosis factor receptor-associated factor (TRAF) 2- and TRAF5-mediated NF-kappaB activation. J Biol Chem 273:5808-5814 doi:10.1074/jbc.273.10.5808
25. Kim T, He L, Johnson MS, Li Y, Zeng L, Ding Y, Long Q, Moore JF, Sharer JD, Nagy TR, Young ME, Wood PA, Yang Q (2014) Carnitine Palmitoyltransferase 1b Deficiency Protects Mice from Diet-Induced Insulin Resistance. J Diabetes Metab 5:361 doi:10.4172/2155-6156.1000361
26. Kleinert H, Wallerath T, Euchenhofer C, Ihrig-Biedert I, Li H, Forstermann U (1998) Estrogens increase transcription of the human endothelial NO synthase gene: analysis of the transcription factors involved. Hypertension 31:582-588 doi:10.1161/01.hyp.31.2.582
27. Kroller-Schon S, Daiber A, Steven S, Oelze M, Frenis K, Kalinovic S, Heimann A, Schmidt FP, Pinto A, Kvandova M, Vujacic-Mirski K, Filippou K, Dudek M, Bosmann M, Klein M, Bopp T, Hahad O, Wild PS, Frauenknecht K, Methner A, Schmidt ER, Rapp S, Mollnau H, Munzel T (2018) Crucial role for Nox2 and sleep deprivation in aircraft noise-induced vascular and cerebral oxidative stress, inflammation, and gene regulation. Eur Heart J 39:3528-3539 doi:10.1093/eurheartj/ehy333
28. Kurdi M, Zgheib C, Booz GW (2018) Recent Developments on the Crosstalk Between STAT3 and Inflammation in Heart Function and Disease. Front Immunol 9:3029 doi:10.3389/fimmu.2018.03029

29. Li C, Li N, Zhang Z, Song Y, Li J, Wang Z, Bo H, Zhang Y (2023) The specific mitochondrial unfolded protein response in fast- and slow-twitch muscles of high-fat diet-induced insulin-resistant rats. Front Endocrinol (Lausanne) 14:1127524 doi:10.3389/fendo.2023.1127524
30. Li D, Zhang C, Li J, Che J, Yang X, Xian Y, Li X, Cao C (2019) Long non-coding RNA MALAT1 promotes cardiac remodeling in hypertensive rats by inhibiting the transcription of MyoD. Aging (Albany NY) 11:8792-8809 doi:10.18632/aging.102265
31. Lin M, Yuan W, Su Z, Lin C, Huang T, Chen Y, Wang J (2018) Yes-associated protein mediates angiotensin II-induced vascular smooth muscle cell phenotypic modulation and hypertensive vascular remodelling. Cell Prolif 51:e12517 doi:10.1111/cpr.12517
32. Liu D, Xu H, Shih C, Wan Z, Ma X, Ma W, Luo D, Qi H (2015) T-B-cell entanglement and ICOSL-driven feed-forward regulation of germinal centre reaction. Nature 517:214–218 doi:10.1038/nature13803
33. Lundberg M, Eriksson A, Tran B, Assarsson E, Fredriksson S (2011) Homogeneous antibody-based proximity extension assays provide sensitive and specific detection of low-abundant proteins in human blood. Nucleic Acids Res 39:e102 doi:10.1093/nar/gkr424
34. Ma BY, Mikolajczak SA, Danesh A, Hosiawa KA, Cameron CM, Takaori-Kondo A, Uchiyama T, Kelvin DJ, Ochi A (2005) The expression and the regulatory role of OX40 and 4-1BB heterodimer in activated human T cells. Blood 106:2002–2010 doi:10.1182/blood-2004-04-1622
35. Marjoribanks J, Farquhar C, Roberts H, Lethaby A, Lee J (2017) Long-term hormone therapy for perimenopausal and postmenopausal women. Cochrane Database Syst Rev 1:CD004143 doi:10.1002/14651858.CD004143.pub5
36. Mathur RK, Awasthi A, Wadhone P, Ramanamurthy B, Saha B (2004) Reciprocal CD40 signals through p38MAPK and ERK-1/2 induce counteracting immune responses. Nat Med 10:540-544 doi:10.1038/nm1045
37. Morris BJ (1997) Insulin receptor gene in hypertension. Clin Exp Hypertens 19:551-565 doi:10.3109/10641969709083169
38. Munzel T, Daiber A, Steven S, Tran LP, Ullmann E, Kossmann S, Schmidt FP, Oelze M, Xia N, Li H, Pinto A, Wild P, Pies K, Schmidt ER, Rapp S, Kroller-Schon S (2017) Effects of noise on vascular function, oxidative stress, and inflammation: mechanistic insight from studies in mice. Eur Heart J 38:2838-2849 doi:10.1093/eurheartj/ehx081
39. Oelze M, Daiber A, Brandes RP, Hortmann M, Wenzel P, Hink U, Schulz E, Mollnau H, von Sandersleben A, Kleschyov AL, Mulsch A, Li H, Forstermann U, Munzel T (2006) Nebivolol inhibits superoxide formation by NADPH oxidase and endothelial dysfunction in angiotensin II-treated rats. Hypertension 48:677-684 doi:10.1161/01.HYP.0000239207.82326.29
40. Oka SI, Sabry AD, Cawley KM, Warren JS (2020) Multiple Levels of PGC-1alpha Dysregulation in Heart Failure. Front Cardiovasc Med 7:2 doi:10.3389/fcvm.2020.00002
41. Pan G, Bauer JH, Haridas V, Wang S, Liu D, Yu G, Vincenz C, Aggarwal BB, Ni J, Dixit VM (1998) Identification and functional characterization of DR6, a novel death domain-containing TNF receptor. FEBS letters 431:351–356 doi:10.1016/s0014-5793(98)00791-1
42. Potthoff MJ, Olson EN (2007) MEF2: a central regulator of diverse developmental programs. Development 134:4131-4140 doi:10.1242/dev.008367
43. Rafiq A, Aashaq S, Jan I, Beigh MA (2021) SIX1 transcription factor: A review of cellular functions and regulatory dynamics. Int J Biol Macromol 193:1151-1164 doi:10.1016/j.ijbiomac.2021.10.133
44. Sahoo S, Meijles DN, Al Ghouleh I, Tandon M, Cifuentes-Pagano E, Sembrat J, Rojas M, Goncharova E, Pagano PJ (2016) MEF2C-MYOCD and Leiomodin1 Suppression by miRNA-214 Promotes Smooth Muscle Cell Phenotype Switching in Pulmonary Arterial Hypertension. PLoS One 11:e0153780 doi:10.1371/journal.pone.0153780
45. Sihag S, Cresci S, Li AY, Sucharov CC, Lehman JJ (2009) PGC-1alpha and ERRalpha target gene downregulation is a signature of the failing human heart. J Mol Cell Cardiol 46:201-212 doi:10.1016/j.yjmcc.2008.10.025

46. Steven S, Dib M, Hausding M, Kashani F, Oelze M, Kroller-Schon S, Hanf A, Daub S, Roohani S, Gramlich Y, Lutgens E, Schulz E, Becker C, Lackner KJ, Kleinert H, Knosalla C, Niesler B, Wild PS, Munzel T, Daiber A (2018) CD40L controls obesity-associated vascular inflammation, oxidative stress, and endothelial dysfunction in high fat diet-treated and db/db mice. Cardiovasc Res 114:312-323 doi:10.1093/cvr/cvx197
47. Steven S, Frenis K, Kalinovic S, Kvandova M, Oelze M, Helmstadter J, Hahad O, Filippou K, Kus K, Trevisan C, Schluter KD, Boengler K, Chlopicki S, Frauenknecht K, Schulz R, Sorensen M, Daiber A, Kroller-Schon S, Munzel T (2020) Exacerbation of adverse cardiovascular effects of aircraft noise in an animal model of arterial hypertension. Redox Biol 34:101515 doi:10.1016/j.redox.2020.101515
48. Sun Q, Li J, Gao F (2014) New insights into insulin: The anti-inflammatory effect and its clinical relevance. World J Diabetes 5:89-96 doi:10.4239/wjd.v5.i2.89
49. Sun Y, Fan W, Xue R, Dong B, Liang Z, Chen C, Li J, Wang Y, Zhao J, Huang H, Jiang J, Wu Z, Dai G, Fang R, Yan Y, Yang T, Huang ZP, Dong Y, Liu C (2020) Transcribed Ultraconserved Regions, Uc.323, Ameliorates Cardiac Hypertrophy by Regulating the Transcription of CPT1b (Carnitine Palmitoyl transferase 1b). Hypertension 75:79-90 doi:10.1161/HYPERTENSIONAHA.119.13173
50. Usmani A, Machado RF (2018) Vascular complications of sickle cell disease. Clin Hemorheol Microcirc 68:205-221 doi:10.3233/CH-189008
51. Wenzel P, Schulz E, Oelze M, Muller J, Schuhmacher S, Alhamdani MS, Debrezion J, Hortmann M, Reifenberg K, Fleming I, Munzel T, Daiber A (2008) AT1-receptor blockade by telmisartan upregulates GTP-cyclohydrolase I and protects eNOS in diabetic rats. Free Radic Biol Med 45:619-626 doi:10.1016/j.freeradbiomed.2008.05.009
52. Yanaba K, Bouaziz J-D, Haas KM, Poe JC, Fujimoto M, Tedder TF (2008) A regulatory B cell subset with a unique CD1dhiCD5+ phenotype controls T cell-dependent inflammatory responses. Immunity 28:639–650 doi:10.1016/j.immuni.2008.03.017
53. Zhu S, Li W, Zhang H, Yan Y, Mei Q, Wu K (2023) Retinal determination gene networks: from biological functions to therapeutic strategies. Biomark Res 11:18 doi:10.1186/s40364-023-00459-8
54. Zuniga E, Rabinovich GA, Iglesias MM, Gruppi A (2001) Regulated expression of galectin-1 during B-cell activation and implications for T-cell apoptosis. J Leukoc Biol 70:73-79
