# Supplementary material for: Role of inflammatory signaling pathways involving the CD40–CD40L–TRAF cascade in diabetes and hypertension—insights from animal and human studies
Source: Basic Res Cardiol. 2024 Mar 30;119(4):1–18. doi: 10.1007/s00395-024-01045-1 (PMC11319409; doi:10.1007/s00395-024-01045-1)
Supplement: Supplementary file 2 — Supplementary file2 (DOCX 261 KB) [file 395_2024_1045_MOESM2_ESM.docx]

| **Suppl. Table S3. RNA-Seq data: 2074 different expressed genes (DEG, p < 0.05) for the comparison CHD + HT vs. CHD.** | | | |
| --- | --- | --- | --- |
| **Gene name** | **Identifier** | **Gene description** | **CHD + HT vs. CHD - Log fold change** |
| A2ML1 | [ENSG00000166535](https://www.ensembl.org/id/ENSG00000166535) | alpha-2-macroglobulin like 1 [Source:HGNC Symbol;Acc:HGNC:23336] | 3,99 |
| AADAC | [ENSG00000114771](https://www.ensembl.org/id/ENSG00000114771) | arylacetamide deacetylase [Source:HGNC Symbol;Acc:HGNC:17] | -8,56 |
| AADACL2 | [ENSG00000197953](https://www.ensembl.org/id/ENSG00000197953) | arylacetamide deacetylase like 2 [Source:HGNC Symbol;Acc:HGNC:24427] | -5,83 |
| AAMDC | [ENSG00000087884](https://www.ensembl.org/id/ENSG00000087884) | adipogenesis associated Mth938 domain containing [Source:HGNC Symbol;Acc:HGNC:30205] | 1,27 |
| ABCB4 | [ENSG00000005471](https://www.ensembl.org/id/ENSG00000005471) | ATP binding cassette subfamily B member 4 [Source:HGNC Symbol;Acc:HGNC:45] | 1,83 |
| ABCB7 | [ENSG00000131269](https://www.ensembl.org/id/ENSG00000131269) | ATP binding cassette subfamily B member 7 [Source:HGNC Symbol;Acc:HGNC:48] | 0,93 |
| ABCD2 | [ENSG00000173208](https://www.ensembl.org/id/ENSG00000173208) | ATP binding cassette subfamily D member 2 [Source:HGNC Symbol;Acc:HGNC:66] | -1,74 |
| ABCF2-H2BK1 | [ENSG00000285292](https://www.ensembl.org/id/ENSG00000285292) | ABCF2-H2BK1 readthrough [Source:HGNC Symbol;Acc:HGNC:54751] | 1,38 |
| ABLIM2 | [ENSG00000163995](https://www.ensembl.org/id/ENSG00000163995) | actin binding LIM protein family member 2 [Source:HGNC Symbol;Acc:HGNC:19195] | 1,92 |
| ACAA2 | [ENSG00000167315](https://www.ensembl.org/id/ENSG00000167315) | acetyl-CoA acyltransferase 2 [Source:HGNC Symbol;Acc:HGNC:83] | 1,19 |
| ACADM | [ENSG00000117054](https://www.ensembl.org/id/ENSG00000117054) | acyl-CoA dehydrogenase medium chain [Source:HGNC Symbol;Acc:HGNC:89] | 1,74 |
| ACADS | [ENSG00000122971](https://www.ensembl.org/id/ENSG00000122971) | acyl-CoA dehydrogenase short chain [Source:HGNC Symbol;Acc:HGNC:90] | 1,47 |
| ACADSB | [ENSG00000196177](https://www.ensembl.org/id/ENSG00000196177) | acyl-CoA dehydrogenase short/branched chain [Source:HGNC Symbol;Acc:HGNC:91] | 1,78 |
| ACAN | [ENSG00000157766](https://www.ensembl.org/id/ENSG00000157766) | aggrecan [Source:HGNC Symbol;Acc:HGNC:319] | -1,71 |
| ACAT1 | [ENSG00000075239](https://www.ensembl.org/id/ENSG00000075239) | acetyl-CoA acetyltransferase 1 [Source:HGNC Symbol;Acc:HGNC:93] | 1,95 |
| ACHE | [ENSG00000087085](https://www.ensembl.org/id/ENSG00000087085) | acetylcholinesterase (Cartwright blood group) [Source:HGNC Symbol;Acc:HGNC:108] | 2,52 |
| ACKR4 | [ENSG00000129048](https://www.ensembl.org/id/ENSG00000129048) | atypical chemokine receptor 4 [Source:HGNC Symbol;Acc:HGNC:1611] | -2,13 |
| ACO2 | [ENSG00000100412](https://www.ensembl.org/id/ENSG00000100412) | aconitase 2 [Source:HGNC Symbol;Acc:HGNC:118] | 1,86 |
| ACOT11 | [ENSG00000162390](https://www.ensembl.org/id/ENSG00000162390) | acyl-CoA thioesterase 11 [Source:HGNC Symbol;Acc:HGNC:18156] | 1,95 |
| ACRBP | [ENSG00000111644](https://www.ensembl.org/id/ENSG00000111644) | acrosin binding protein [Source:HGNC Symbol;Acc:HGNC:17195] | 4,11 |
| ACSL6 | [ENSG00000164398](https://www.ensembl.org/id/ENSG00000164398) | acyl-CoA synthetase long chain family member 6 [Source:HGNC Symbol;Acc:HGNC:16496] | 1,95 |
| ACSS1 | [ENSG00000154930](https://www.ensembl.org/id/ENSG00000154930) | acyl-CoA synthetase short chain family member 1 [Source:HGNC Symbol;Acc:HGNC:16091] | 1,81 |
| ACTN2 | [ENSG00000077522](https://www.ensembl.org/id/ENSG00000077522) | actinin alpha 2 [Source:HGNC Symbol;Acc:HGNC:164] | 2,91 |
| ACVR1C | [ENSG00000123612](https://www.ensembl.org/id/ENSG00000123612) | activin A receptor type 1C [Source:HGNC Symbol;Acc:HGNC:18123] | -3,21 |
| ACY3 | [ENSG00000132744](https://www.ensembl.org/id/ENSG00000132744) | aminoacylase 3 [Source:HGNC Symbol;Acc:HGNC:24104] | 3,87 |
| ACYP2 | [ENSG00000170634](https://www.ensembl.org/id/ENSG00000170634) | acylphosphatase 2 [Source:HGNC Symbol;Acc:HGNC:180] | 1,82 |
| ADAM12 | [ENSG00000148848](https://www.ensembl.org/id/ENSG00000148848) | ADAM metallopeptidase domain 12 [Source:HGNC Symbol;Acc:HGNC:190] | -2,63 |
| ADAMTS7 | [ENSG00000136378](https://www.ensembl.org/id/ENSG00000136378) | ADAM metallopeptidase with thrombospondin type 1 motif 7 [Source:HGNC Symbol;Acc:HGNC:223] | -1,35 |
| ADAMTS9 | [ENSG00000163638](https://www.ensembl.org/id/ENSG00000163638) | ADAM metallopeptidase with thrombospondin type 1 motif 9 [Source:HGNC Symbol;Acc:HGNC:13202] | -1,84 |
| ADCY2 | [ENSG00000078295](https://www.ensembl.org/id/ENSG00000078295) | adenylate cyclase 2 [Source:HGNC Symbol;Acc:HGNC:233] | 2,51 |
| ADGRE2 | [ENSG00000127507](https://www.ensembl.org/id/ENSG00000127507) | adhesion G protein-coupled receptor E2 [Source:HGNC Symbol;Acc:HGNC:3337] | 2,89 |
| ADGRE3 | [ENSG00000131355](https://www.ensembl.org/id/ENSG00000131355) | adhesion G protein-coupled receptor E3 [Source:HGNC Symbol;Acc:HGNC:23647] | 4,71 |
| ADGRG3 | [ENSG00000182885](https://www.ensembl.org/id/ENSG00000182885) | adhesion G protein-coupled receptor G3 [Source:HGNC Symbol;Acc:HGNC:13728] | 2,72 |
| ADGRG7 | [ENSG00000144820](https://www.ensembl.org/id/ENSG00000144820) | adhesion G protein-coupled receptor G7 [Source:HGNC Symbol;Acc:HGNC:19241] | -10,07 |
| ADGRL1 | [ENSG00000072071](https://www.ensembl.org/id/ENSG00000072071) | adhesion G protein-coupled receptor L1 [Source:HGNC Symbol;Acc:HGNC:20973] | -0,98 |
| ADHFE1 | [ENSG00000147576](https://www.ensembl.org/id/ENSG00000147576) | alcohol dehydrogenase iron containing 1 [Source:HGNC Symbol;Acc:HGNC:16354] | 1,10 |
| ADIPOQ | [ENSG00000181092](https://www.ensembl.org/id/ENSG00000181092) | adiponectin, C1Q and collagen domain containing [Source:HGNC Symbol;Acc:HGNC:13633] | -3,20 |
| ADIRF-AS1 | [ENSG00000272734](https://www.ensembl.org/id/ENSG00000272734) | ADIRF antisense RNA 1 [Source:HGNC Symbol;Acc:HGNC:45127] | -1,26 |
| ADPGK-AS1 | [ENSG00000260898](https://www.ensembl.org/id/ENSG00000260898) | ADPGK antisense RNA 1 [Source:HGNC Symbol;Acc:HGNC:44144] | 3,07 |
| ADPRHL1 | [ENSG00000153531](https://www.ensembl.org/id/ENSG00000153531) | ADP-ribosylhydrolase like 1 [Source:HGNC Symbol;Acc:HGNC:21303] | 3,07 |
| ADRA2A | [ENSG00000150594](https://www.ensembl.org/id/ENSG00000150594) | adrenoceptor alpha 2A [Source:HGNC Symbol;Acc:HGNC:281] | -1,45 |
| ADSL | [ENSG00000239900](https://www.ensembl.org/id/ENSG00000239900) | adenylosuccinate lyase [Source:HGNC Symbol;Acc:HGNC:291] | 1,51 |
| ADSS1 | [ENSG00000185100](https://www.ensembl.org/id/ENSG00000185100) | adenylosuccinate synthase 1 [Source:HGNC Symbol;Acc:HGNC:20093] | 1,94 |
| AFG3L2 | [ENSG00000141385](https://www.ensembl.org/id/ENSG00000141385) | AFG3 like matrix AAA peptidase subunit 2 [Source:HGNC Symbol;Acc:HGNC:315] | 1,22 |
| AGBL1 | [ENSG00000273540](https://www.ensembl.org/id/ENSG00000273540) | AGBL carboxypeptidase 1 [Source:HGNC Symbol;Acc:HGNC:26504] | 3,27 |
| AGK-DT | [ENSG00000261570](https://www.ensembl.org/id/ENSG00000261570) | AGK divergent transcript [Source:HGNC Symbol;Acc:HGNC:55356] | 3,99 |
| AGL | [ENSG00000162688](https://www.ensembl.org/id/ENSG00000162688) | amylo-alpha-1, 6-glucosidase, 4-alpha-glucanotransferase [Source:HGNC Symbol;Acc:HGNC:321] | 1,56 |
| AGTR2 | [ENSG00000180772](https://www.ensembl.org/id/ENSG00000180772) | angiotensin II receptor type 2 [Source:HGNC Symbol;Acc:HGNC:338] | 3,73 |
| AHCY | [ENSG00000101444](https://www.ensembl.org/id/ENSG00000101444) | adenosylhomocysteinase [Source:HGNC Symbol;Acc:HGNC:343] | 0,84 |
| AHR | [ENSG00000106546](https://www.ensembl.org/id/ENSG00000106546) | aryl hydrocarbon receptor [Source:HGNC Symbol;Acc:HGNC:348] | -0,98 |
| AKR1B1 | [ENSG00000085662](https://www.ensembl.org/id/ENSG00000085662) | aldo-keto reductase family 1 member B [Source:HGNC Symbol;Acc:HGNC:381] | 1,84 |
| ALDH1L1-AS2 | [ENSG00000246022](https://www.ensembl.org/id/ENSG00000246022) | ALDH1L1 antisense RNA 2 [Source:HGNC Symbol;Acc:HGNC:42446] | 2,53 |
| ALDH5A1 | [ENSG00000112294](https://www.ensembl.org/id/ENSG00000112294) | aldehyde dehydrogenase 5 family member A1 [Source:HGNC Symbol;Acc:HGNC:408] | 1,83 |
| ALDH6A1 | [ENSG00000119711](https://www.ensembl.org/id/ENSG00000119711) | aldehyde dehydrogenase 6 family member A1 [Source:HGNC Symbol;Acc:HGNC:7179] | 1,81 |
| ALOX12 | [ENSG00000108839](https://www.ensembl.org/id/ENSG00000108839) | arachidonate 12-lipoxygenase, 12S type [Source:HGNC Symbol;Acc:HGNC:429] | 2,85 |
| ALOX15 | [ENSG00000161905](https://www.ensembl.org/id/ENSG00000161905) | arachidonate 15-lipoxygenase [Source:HGNC Symbol;Acc:HGNC:433] | -8,41 |
| ALOX5 | [ENSG00000012779](https://www.ensembl.org/id/ENSG00000012779) | arachidonate 5-lipoxygenase [Source:HGNC Symbol;Acc:HGNC:435] | 2,29 |
| ALOX5AP | [ENSG00000132965](https://www.ensembl.org/id/ENSG00000132965) | arachidonate 5-lipoxygenase activating protein [Source:HGNC Symbol;Acc:HGNC:436] | 2,48 |
| ALPK3 | [ENSG00000136383](https://www.ensembl.org/id/ENSG00000136383) | alpha kinase 3 [Source:HGNC Symbol;Acc:HGNC:17574] | 1,48 |
| ALX1 | [ENSG00000180318](https://www.ensembl.org/id/ENSG00000180318) | ALX homeobox 1 [Source:HGNC Symbol;Acc:HGNC:1494] | -2,67 |
| AMACR | [ENSG00000242110](https://www.ensembl.org/id/ENSG00000242110) | alpha-methylacyl-CoA racemase [Source:HGNC Symbol;Acc:HGNC:451] | 1,47 |
| AMPD3 | [ENSG00000133805](https://www.ensembl.org/id/ENSG00000133805) | adenosine monophosphate deaminase 3 [Source:HGNC Symbol;Acc:HGNC:470] | 2,20 |
| ANGPTL2 | [ENSG00000136859](https://www.ensembl.org/id/ENSG00000136859) | angiopoietin like 2 [Source:HGNC Symbol;Acc:HGNC:490] | -1,20 |
| ANGPTL4 | [ENSG00000167772](https://www.ensembl.org/id/ENSG00000167772) | angiopoietin like 4 [Source:HGNC Symbol;Acc:HGNC:16039] | -2,13 |
| ANK1 | [ENSG00000029534](https://www.ensembl.org/id/ENSG00000029534) | ankyrin 1 [Source:HGNC Symbol;Acc:HGNC:492] | 1,91 |
| ANK3 | [ENSG00000151150](https://www.ensembl.org/id/ENSG00000151150) | ankyrin 3 [Source:HGNC Symbol;Acc:HGNC:494] | 1,47 |
| ANKRD18B | [ENSG00000230453](https://www.ensembl.org/id/ENSG00000230453) | ankyrin repeat domain 18B [Source:HGNC Symbol;Acc:HGNC:23644] | 3,59 |
| ANKRD2 | [ENSG00000165887](https://www.ensembl.org/id/ENSG00000165887) | ankyrin repeat domain 2 [Source:HGNC Symbol;Acc:HGNC:495] | 3,69 |
| ANKRD39 | [ENSG00000213337](https://www.ensembl.org/id/ENSG00000213337) | ankyrin repeat domain 39 [Source:HGNC Symbol;Acc:HGNC:28640] | 1,55 |
| ANKRD9 | [ENSG00000156381](https://www.ensembl.org/id/ENSG00000156381) | ankyrin repeat domain 9 [Source:HGNC Symbol;Acc:HGNC:20096] | 1,36 |
| ANO5 | [ENSG00000171714](https://www.ensembl.org/id/ENSG00000171714) | anoctamin 5 [Source:HGNC Symbol;Acc:HGNC:27337] | 1,40 |
| ANXA13 | [ENSG00000104537](https://www.ensembl.org/id/ENSG00000104537) | annexin A13 [Source:HGNC Symbol;Acc:HGNC:536] | -4,38 |
| ANXA8 | [ENSG00000265190](https://www.ensembl.org/id/ENSG00000265190) | annexin A8 [Source:HGNC Symbol;Acc:HGNC:546] | -10,03 |
| ANXA8L1 | [ENSG00000264230](https://www.ensembl.org/id/ENSG00000264230) | annexin A8 like 1 [Source:HGNC Symbol;Acc:HGNC:23334] | -5,94 |
| AOAH | [ENSG00000136250](https://www.ensembl.org/id/ENSG00000136250) | acyloxyacyl hydrolase [Source:HGNC Symbol;Acc:HGNC:548] | 2,16 |
| AP1M2 | [ENSG00000129354](https://www.ensembl.org/id/ENSG00000129354) | adaptor related protein complex 1 subunit mu 2 [Source:HGNC Symbol;Acc:HGNC:558] | -4,18 |
| AP1S2 | [ENSG00000182287](https://www.ensembl.org/id/ENSG00000182287) | adaptor related protein complex 1 subunit sigma 2 [Source:HGNC Symbol;Acc:HGNC:560] | 1,21 |
| APCDD1 | [ENSG00000154856](https://www.ensembl.org/id/ENSG00000154856) | APC down-regulated 1 [Source:HGNC Symbol;Acc:HGNC:15718] | -1,75 |
| APEH | [ENSG00000164062](https://www.ensembl.org/id/ENSG00000164062) | acylaminoacyl-peptide hydrolase [Source:HGNC Symbol;Acc:HGNC:586] | 0,97 |
| APOBEC3A | [ENSG00000128383](https://www.ensembl.org/id/ENSG00000128383) | apolipoprotein B mRNA editing enzyme catalytic subunit 3A [Source:HGNC Symbol;Acc:HGNC:17343] | 3,84 |
| APOC2 | [ENSG00000234906](https://www.ensembl.org/id/ENSG00000234906) | apolipoprotein C2 [Source:HGNC Symbol;Acc:HGNC:609] | 5,24 |
| AQP4 | [ENSG00000171885](https://www.ensembl.org/id/ENSG00000171885) | aquaporin 4 [Source:HGNC Symbol;Acc:HGNC:637] | 3,49 |
| AQP4-AS1 | [ENSG00000260372](https://www.ensembl.org/id/ENSG00000260372) | AQP4 antisense RNA 1 [Source:HGNC Symbol;Acc:HGNC:26399] | 4,37 |
| ARHGAP30 | [ENSG00000186517](https://www.ensembl.org/id/ENSG00000186517) | Rho GTPase activating protein 30 [Source:HGNC Symbol;Acc:HGNC:27414] | 1,96 |
| ARL14EP | [ENSG00000152219](https://www.ensembl.org/id/ENSG00000152219) | ADP ribosylation factor like GTPase 14 effector protein [Source:HGNC Symbol;Acc:HGNC:26798] | 1,00 |
| ARL2-SNX15 | [ENSG00000273003](https://www.ensembl.org/id/ENSG00000273003) | ARL2-SNX15 readthrough (NMD candidate) [Source:HGNC Symbol;Acc:HGNC:49197] | 5,41 |
| ARMS2 | [ENSG00000254636](https://www.ensembl.org/id/ENSG00000254636) | age-related maculopathy susceptibility 2 [Source:HGNC Symbol;Acc:HGNC:32685] | 3,57 |
| ART5 | [ENSG00000167311](https://www.ensembl.org/id/ENSG00000167311) | ADP-ribosyltransferase 5 [Source:HGNC Symbol;Acc:HGNC:24049] | 2,66 |
| ASB8 | [ENSG00000177981](https://www.ensembl.org/id/ENSG00000177981) | ankyrin repeat and SOCS box containing 8 [Source:HGNC Symbol;Acc:HGNC:17183] | 1,61 |
| ASCL2 | [ENSG00000183734](https://www.ensembl.org/id/ENSG00000183734) | achaete-scute family bHLH transcription factor 2 [Source:HGNC Symbol;Acc:HGNC:739] | 2,80 |
| ASPHD1 | [ENSG00000174939](https://www.ensembl.org/id/ENSG00000174939) | aspartate beta-hydroxylase domain containing 1 [Source:HGNC Symbol;Acc:HGNC:27380] | -4,20 |
| ATG9B | [ENSG00000181652](https://www.ensembl.org/id/ENSG00000181652) | autophagy related 9B [Source:HGNC Symbol;Acc:HGNC:21899] | -2,00 |
| ATP12A | [ENSG00000075673](https://www.ensembl.org/id/ENSG00000075673) | ATPase H+/K+ transporting non-gastric alpha2 subunit [Source:HGNC Symbol;Acc:HGNC:13816] | -2,89 |
| ATP1A2 | [ENSG00000018625](https://www.ensembl.org/id/ENSG00000018625) | ATPase Na+/K+ transporting subunit alpha 2 [Source:HGNC Symbol;Acc:HGNC:800] | 1,59 |
| ATP1B1 | [ENSG00000143153](https://www.ensembl.org/id/ENSG00000143153) | ATPase Na+/K+ transporting subunit beta 1 [Source:HGNC Symbol;Acc:HGNC:804] | 1,97 |
| ATP2A2 | [ENSG00000174437](https://www.ensembl.org/id/ENSG00000174437) | ATPase sarcoplasmic/endoplasmic reticulum Ca2+ transporting 2 [Source:HGNC Symbol;Acc:HGNC:812] | 2,25 |
| ATP5F1B | [ENSG00000110955](https://www.ensembl.org/id/ENSG00000110955) | ATP synthase F1 subunit beta [Source:HGNC Symbol;Acc:HGNC:830] | 1,88 |
| ATP5F1C | [ENSG00000165629](https://www.ensembl.org/id/ENSG00000165629) | ATP synthase F1 subunit gamma [Source:HGNC Symbol;Acc:HGNC:833] | 1,22 |
| ATP5IF1 | [ENSG00000130770](https://www.ensembl.org/id/ENSG00000130770) | ATP synthase inhibitory factor subunit 1 [Source:HGNC Symbol;Acc:HGNC:871] | 1,53 |
| ATP5MC1 | [ENSG00000159199](https://www.ensembl.org/id/ENSG00000159199) | ATP synthase membrane subunit c locus 1 [Source:HGNC Symbol;Acc:HGNC:841] | 1,53 |
| ATP5MC3 | [ENSG00000154518](https://www.ensembl.org/id/ENSG00000154518) | ATP synthase membrane subunit c locus 3 [Source:HGNC Symbol;Acc:HGNC:843] | 1,51 |
| ATP5MF-PTCD1 | [ENSG00000248919](https://www.ensembl.org/id/ENSG00000248919) | ATP5MF-PTCD1 readthrough [Source:HGNC Symbol;Acc:HGNC:38844] | 8,29 |
| ATP5MJ | [ENSG00000156411](https://www.ensembl.org/id/ENSG00000156411) | ATP synthase membrane subunit j [Source:HGNC Symbol;Acc:HGNC:1188] | 1,14 |
| ATP5MK | [ENSG00000173915](https://www.ensembl.org/id/ENSG00000173915) | ATP synthase membrane subunit k [Source:HGNC Symbol;Acc:HGNC:30889] | 1,03 |
| ATP5PB | [ENSG00000116459](https://www.ensembl.org/id/ENSG00000116459) | ATP synthase peripheral stalk-membrane subunit b [Source:HGNC Symbol;Acc:HGNC:840] | 1,31 |
| ATP5PD | [ENSG00000167863](https://www.ensembl.org/id/ENSG00000167863) | ATP synthase peripheral stalk subunit d [Source:HGNC Symbol;Acc:HGNC:845] | 1,24 |
| ATP5PF | [ENSG00000154723](https://www.ensembl.org/id/ENSG00000154723) | ATP synthase peripheral stalk subunit F6 [Source:HGNC Symbol;Acc:HGNC:847] | 1,21 |
| ATP5PO | [ENSG00000241837](https://www.ensembl.org/id/ENSG00000241837) | ATP synthase peripheral stalk subunit OSCP [Source:HGNC Symbol;Acc:HGNC:850] | 1,18 |
| ATP8A1 | [ENSG00000124406](https://www.ensembl.org/id/ENSG00000124406) | ATPase phospholipid transporting 8A1 [Source:HGNC Symbol;Acc:HGNC:13531] | 1,34 |
| ATP8B3 | [ENSG00000130270](https://www.ensembl.org/id/ENSG00000130270) | ATPase phospholipid transporting 8B3 [Source:HGNC Symbol;Acc:HGNC:13535] | -1,93 |
| ATPAF1 | [ENSG00000123472](https://www.ensembl.org/id/ENSG00000123472) | ATP synthase mitochondrial F1 complex assembly factor 1 [Source:HGNC Symbol;Acc:HGNC:18803] | 1,43 |
| AURKB | [ENSG00000178999](https://www.ensembl.org/id/ENSG00000178999) | aurora kinase B [Source:HGNC Symbol;Acc:HGNC:11390] | 3,45 |
| AZGP1 | [ENSG00000160862](https://www.ensembl.org/id/ENSG00000160862) | alpha-2-glycoprotein 1, zinc-binding [Source:HGNC Symbol;Acc:HGNC:910] | -2,38 |
| AZU1 | [ENSG00000172232](https://www.ensembl.org/id/ENSG00000172232) | azurocidin 1 [Source:HGNC Symbol;Acc:HGNC:913] | -2,99 |
| B3GNT3 | [ENSG00000179913](https://www.ensembl.org/id/ENSG00000179913) | UDP-GlcNAc:betaGal beta-1,3-N-acetylglucosaminyltransferase 3 [Source:HGNC Symbol;Acc:HGNC:13528] | 3,50 |
| BACH1-IT2 | [ENSG00000228817](https://www.ensembl.org/id/ENSG00000228817) | BACH1 intronic transcript 2 [Source:HGNC Symbol;Acc:HGNC:40007] | -3,49 |
| BCKDHA | [ENSG00000248098](https://www.ensembl.org/id/ENSG00000248098) | branched chain keto acid dehydrogenase E1 subunit alpha [Source:HGNC Symbol;Acc:HGNC:986] | 1,73 |
| BCL11B | [ENSG00000127152](https://www.ensembl.org/id/ENSG00000127152) | BAF chromatin remodeling complex subunit BCL11B [Source:HGNC Symbol;Acc:HGNC:13222] | 2,66 |
| BCL2A1 | [ENSG00000140379](https://www.ensembl.org/id/ENSG00000140379) | BCL2 related protein A1 [Source:HGNC Symbol;Acc:HGNC:991] | 3,51 |
| BDH1 | [ENSG00000161267](https://www.ensembl.org/id/ENSG00000161267) | 3-hydroxybutyrate dehydrogenase 1 [Source:HGNC Symbol;Acc:HGNC:1027] | 3,35 |
| BDKRB1 | [ENSG00000100739](https://www.ensembl.org/id/ENSG00000100739) | bradykinin receptor B1 [Source:HGNC Symbol;Acc:HGNC:1029] | -4,92 |
| BEAN1 | [ENSG00000166546](https://www.ensembl.org/id/ENSG00000166546) | brain expressed associated with NEDD4 1 [Source:HGNC Symbol;Acc:HGNC:24160] | -1,87 |
| BEND4 | [ENSG00000188848](https://www.ensembl.org/id/ENSG00000188848) | BEN domain containing 4 [Source:HGNC Symbol;Acc:HGNC:23815] | 4,35 |
| BICD1 | [ENSG00000151746](https://www.ensembl.org/id/ENSG00000151746) | BICD cargo adaptor 1 [Source:HGNC Symbol;Acc:HGNC:1049] | 1,06 |
| BICDL1 | [ENSG00000135127](https://www.ensembl.org/id/ENSG00000135127) | BICD family like cargo adaptor 1 [Source:HGNC Symbol;Acc:HGNC:28095] | -3,01 |
| BIN1 | [ENSG00000136717](https://www.ensembl.org/id/ENSG00000136717) | bridging integrator 1 [Source:HGNC Symbol;Acc:HGNC:1052] | 2,16 |
| BIN2 | [ENSG00000110934](https://www.ensembl.org/id/ENSG00000110934) | bridging integrator 2 [Source:HGNC Symbol;Acc:HGNC:1053] | 2,31 |
| BIVM-ERCC5 | [ENSG00000270181](https://www.ensembl.org/id/ENSG00000270181) | BIVM-ERCC5 readthrough [Source:HGNC Symbol;Acc:HGNC:43690] | 6,96 |
| BMP1 | [ENSG00000168487](https://www.ensembl.org/id/ENSG00000168487) | bone morphogenetic protein 1 [Source:HGNC Symbol;Acc:HGNC:1067] | -1,24 |
| BMP2 | [ENSG00000125845](https://www.ensembl.org/id/ENSG00000125845) | bone morphogenetic protein 2 [Source:HGNC Symbol;Acc:HGNC:1069] | -1,77 |
| BNC1 | [ENSG00000169594](https://www.ensembl.org/id/ENSG00000169594) | basonuclin 1 [Source:HGNC Symbol;Acc:HGNC:1081] | -4,85 |
| BOK | [ENSG00000176720](https://www.ensembl.org/id/ENSG00000176720) | BCL2 family apoptosis regulator BOK [Source:HGNC Symbol;Acc:HGNC:1087] | -2,11 |
| BOLA3 | [ENSG00000163170](https://www.ensembl.org/id/ENSG00000163170) | bolA family member 3 [Source:HGNC Symbol;Acc:HGNC:24415] | 1,19 |
| BRCA2 | [ENSG00000139618](https://www.ensembl.org/id/ENSG00000139618) | BRCA2 DNA repair associated [Source:HGNC Symbol;Acc:HGNC:1101] | -3,27 |
| BRINP1 | [ENSG00000078725](https://www.ensembl.org/id/ENSG00000078725) | BMP/retinoic acid inducible neural specific 1 [Source:HGNC Symbol;Acc:HGNC:2687] | 1,99 |
| BSN-DT | [ENSG00000226913](https://www.ensembl.org/id/ENSG00000226913) | BSN divergent transcript [Source:HGNC Symbol;Acc:HGNC:42445] | 3,46 |
| BTBD1 | [ENSG00000064726](https://www.ensembl.org/id/ENSG00000064726) | BTB domain containing 1 [Source:HGNC Symbol;Acc:HGNC:1120] | 1,61 |
| BZW2 | [ENSG00000136261](https://www.ensembl.org/id/ENSG00000136261) | basic leucine zipper and W2 domains 2 [Source:HGNC Symbol;Acc:HGNC:18808] | 2,22 |
| C10orf105 | [ENSG00000214688](https://www.ensembl.org/id/ENSG00000214688) | chromosome 10 open reading frame 105 [Source:HGNC Symbol;Acc:HGNC:20304] | -3,03 |
| C10orf67 | [ENSG00000179133](https://www.ensembl.org/id/ENSG00000179133) | chromosome 10 open reading frame 67 [Source:HGNC Symbol;Acc:HGNC:28716] | 4,42 |
| C11orf65 | [ENSG00000166323](https://www.ensembl.org/id/ENSG00000166323) | chromosome 11 open reading frame 65 [Source:HGNC Symbol;Acc:HGNC:28519] | 3,67 |
| C11orf87 | [ENSG00000185742](https://www.ensembl.org/id/ENSG00000185742) | chromosome 11 open reading frame 87 [Source:HGNC Symbol;Acc:HGNC:33788] | 5,05 |
| C15orf61 | [ENSG00000189227](https://www.ensembl.org/id/ENSG00000189227) | chromosome 15 open reading frame 61 [Source:HGNC Symbol;Acc:HGNC:34453] | 1,38 |
| C16orf54 | [ENSG00000185905](https://www.ensembl.org/id/ENSG00000185905) | chromosome 16 open reading frame 54 [Source:HGNC Symbol;Acc:HGNC:26649] | 2,81 |
| C19orf33 | [ENSG00000167644](https://www.ensembl.org/id/ENSG00000167644) | chromosome 19 open reading frame 33 [Source:HGNC Symbol;Acc:HGNC:16668] | -2,23 |
| C19orf38 | [ENSG00000214212](https://www.ensembl.org/id/ENSG00000214212) | chromosome 19 open reading frame 38 [Source:HGNC Symbol;Acc:HGNC:34073] | 2,74 |
| C19orf47 | [ENSG00000160392](https://www.ensembl.org/id/ENSG00000160392) | chromosome 19 open reading frame 47 [Source:HGNC Symbol;Acc:HGNC:26723] | 1,21 |
| C1orf105 | [ENSG00000180999](https://www.ensembl.org/id/ENSG00000180999) | chromosome 1 open reading frame 105 [Source:HGNC Symbol;Acc:HGNC:29591] | 4,62 |
| C1orf21 | [ENSG00000116667](https://www.ensembl.org/id/ENSG00000116667) | chromosome 1 open reading frame 21 [Source:HGNC Symbol;Acc:HGNC:15494] | 1,40 |
| C1QBP | [ENSG00000108561](https://www.ensembl.org/id/ENSG00000108561) | complement C1q binding protein [Source:HGNC Symbol;Acc:HGNC:1243] | 1,10 |
| C1QTNF9B | [ENSG00000205863](https://www.ensembl.org/id/ENSG00000205863) | C1q and TNF related 9B [Source:HGNC Symbol;Acc:HGNC:34072] | 2,15 |
| C20orf203 | [ENSG00000198547](https://www.ensembl.org/id/ENSG00000198547) | chromosome 20 open reading frame 203 [Source:HGNC Symbol;Acc:HGNC:26592] | -3,03 |
| C22orf42 | [ENSG00000205856](https://www.ensembl.org/id/ENSG00000205856) | chromosome 22 open reading frame 42 [Source:HGNC Symbol;Acc:HGNC:27160] | -5,04 |
| C4A | [ENSG00000244731](https://www.ensembl.org/id/ENSG00000244731) | complement C4A (Rodgers blood group) [Source:HGNC Symbol;Acc:HGNC:1323] | -1,62 |
| C4A-AS1 | [ENSG00000233627](https://www.ensembl.org/id/ENSG00000233627) | C4A antisense RNA 1 [Source:HGNC Symbol;Acc:HGNC:39753] | -2,13 |
| C4B | [ENSG00000224389](https://www.ensembl.org/id/ENSG00000224389) | complement C4B (Chido blood group) [Source:HGNC Symbol;Acc:HGNC:1324] | -1,87 |
| C4orf47 | [ENSG00000205129](https://www.ensembl.org/id/ENSG00000205129) | chromosome 4 open reading frame 47 [Source:HGNC Symbol;Acc:HGNC:34346] | -3,10 |
| C5AR1 | [ENSG00000197405](https://www.ensembl.org/id/ENSG00000197405) | complement C5a receptor 1 [Source:HGNC Symbol;Acc:HGNC:1338] | 1,97 |
| C6orf47-AS1 | [ENSG00000227198](https://www.ensembl.org/id/ENSG00000227198) | C6orf47 antisense RNA 1 [Source:HGNC Symbol;Acc:HGNC:39767] | -5,20 |
| C9 | [ENSG00000113600](https://www.ensembl.org/id/ENSG00000113600) | complement C9 [Source:HGNC Symbol;Acc:HGNC:1358] | -4,90 |
| CA7 | [ENSG00000168748](https://www.ensembl.org/id/ENSG00000168748) | carbonic anhydrase 7 [Source:HGNC Symbol;Acc:HGNC:1381] | 3,91 |
| CA9 | [ENSG00000107159](https://www.ensembl.org/id/ENSG00000107159) | carbonic anhydrase 9 [Source:HGNC Symbol;Acc:HGNC:1383] | -8,56 |
| CABLES1 | [ENSG00000134508](https://www.ensembl.org/id/ENSG00000134508) | Cdk5 and Abl enzyme substrate 1 [Source:HGNC Symbol;Acc:HGNC:25097] | -1,18 |
| CABP5 | [ENSG00000105507](https://www.ensembl.org/id/ENSG00000105507) | calcium binding protein 5 [Source:HGNC Symbol;Acc:HGNC:13714] | 4,12 |
| CACNA1F | [ENSG00000102001](https://www.ensembl.org/id/ENSG00000102001) | calcium voltage-gated channel subunit alpha1 F [Source:HGNC Symbol;Acc:HGNC:1393] | -2,54 |
| CACNA1S | [ENSG00000081248](https://www.ensembl.org/id/ENSG00000081248) | calcium voltage-gated channel subunit alpha1 S [Source:HGNC Symbol;Acc:HGNC:1397] | 3,18 |
| CACNA2D1 | [ENSG00000153956](https://www.ensembl.org/id/ENSG00000153956) | calcium voltage-gated channel auxiliary subunit alpha2delta 1 [Source:HGNC Symbol;Acc:HGNC:1399] | 1,29 |
| CACNG2 | [ENSG00000166862](https://www.ensembl.org/id/ENSG00000166862) | calcium voltage-gated channel auxiliary subunit gamma 2 [Source:HGNC Symbol;Acc:HGNC:1406] | 5,68 |
| CALB2 | [ENSG00000172137](https://www.ensembl.org/id/ENSG00000172137) | calbindin 2 [Source:HGNC Symbol;Acc:HGNC:1435] | -3,71 |
| CALHM5 | [ENSG00000178033](https://www.ensembl.org/id/ENSG00000178033) | calcium homeostasis modulator family member 5 [Source:HGNC Symbol;Acc:HGNC:21568] | -1,15 |
| CALML3-AS1 | [ENSG00000205488](https://www.ensembl.org/id/ENSG00000205488) | CALML3 antisense RNA 1 [Source:HGNC Symbol;Acc:HGNC:44682] | -2,88 |
| CAMK1G | [ENSG00000008118](https://www.ensembl.org/id/ENSG00000008118) | calcium/calmodulin dependent protein kinase IG [Source:HGNC Symbol;Acc:HGNC:14585] | 3,10 |
| CAMK2D | [ENSG00000145349](https://www.ensembl.org/id/ENSG00000145349) | calcium/calmodulin dependent protein kinase II delta [Source:HGNC Symbol;Acc:HGNC:1462] | 1,02 |
| CAP2 | [ENSG00000112186](https://www.ensembl.org/id/ENSG00000112186) | cyclase associated actin cytoskeleton regulatory protein 2 [Source:HGNC Symbol;Acc:HGNC:20039] | 1,71 |
| CAPG | [ENSG00000042493](https://www.ensembl.org/id/ENSG00000042493) | capping actin protein, gelsolin like [Source:HGNC Symbol;Acc:HGNC:1474] | -1,04 |
| CAPN3 | [ENSG00000092529](https://www.ensembl.org/id/ENSG00000092529) | calpain 3 [Source:HGNC Symbol;Acc:HGNC:1480] | 1,83 |
| CASC19 | [ENSG00000254166](https://www.ensembl.org/id/ENSG00000254166) | cancer susceptibility 19 [Source:HGNC Symbol;Acc:HGNC:49476] | -3,55 |
| CAV3 | [ENSG00000182533](https://www.ensembl.org/id/ENSG00000182533) | caveolin 3 [Source:HGNC Symbol;Acc:HGNC:1529] | 2,14 |
| CBLB | [ENSG00000114423](https://www.ensembl.org/id/ENSG00000114423) | Cbl proto-oncogene B [Source:HGNC Symbol;Acc:HGNC:1542] | -1,00 |
| CBY3 | [ENSG00000204659](https://www.ensembl.org/id/ENSG00000204659) | chibby family member 3 [Source:HGNC Symbol;Acc:HGNC:33278] | 5,12 |
| CCDC136 | [ENSG00000128596](https://www.ensembl.org/id/ENSG00000128596) | coiled-coil domain containing 136 [Source:HGNC Symbol;Acc:HGNC:22225] | 1,71 |
| CCDC141 | [ENSG00000163492](https://www.ensembl.org/id/ENSG00000163492) | coiled-coil domain containing 141 [Source:HGNC Symbol;Acc:HGNC:26821] | 3,04 |
| CCDC43 | [ENSG00000180329](https://www.ensembl.org/id/ENSG00000180329) | coiled-coil domain containing 43 [Source:HGNC Symbol;Acc:HGNC:26472] | 1,26 |
| CCDC81 | [ENSG00000149201](https://www.ensembl.org/id/ENSG00000149201) | coiled-coil domain containing 81 [Source:HGNC Symbol;Acc:HGNC:26281] | -2,40 |
| CCL13 | [ENSG00000181374](https://www.ensembl.org/id/ENSG00000181374) | C-C motif chemokine ligand 13 [Source:HGNC Symbol;Acc:HGNC:10611] | -2,62 |
| CCL21 | [ENSG00000137077](https://www.ensembl.org/id/ENSG00000137077) | C-C motif chemokine ligand 21 [Source:HGNC Symbol;Acc:HGNC:10620] | -2,59 |
| CCL25 | [ENSG00000131142](https://www.ensembl.org/id/ENSG00000131142) | C-C motif chemokine ligand 25 [Source:HGNC Symbol;Acc:HGNC:10624] | 5,85 |
| CCL5 | [ENSG00000271503](https://www.ensembl.org/id/ENSG00000271503) | C-C motif chemokine ligand 5 [Source:HGNC Symbol;Acc:HGNC:10632] | 2,07 |
| CCL7 | [ENSG00000108688](https://www.ensembl.org/id/ENSG00000108688) | C-C motif chemokine ligand 7 [Source:HGNC Symbol;Acc:HGNC:10634] | -4,34 |
| CCL8 | [ENSG00000108700](https://www.ensembl.org/id/ENSG00000108700) | C-C motif chemokine ligand 8 [Source:HGNC Symbol;Acc:HGNC:10635] | -1,83 |
| CCR2 | [ENSG00000121807](https://www.ensembl.org/id/ENSG00000121807) | C-C motif chemokine receptor 2 [Source:HGNC Symbol;Acc:HGNC:1603] | 2,43 |
| CCR3 | [ENSG00000183625](https://www.ensembl.org/id/ENSG00000183625) | C-C motif chemokine receptor 3 [Source:HGNC Symbol;Acc:HGNC:1604] | 5,07 |
| CCR5AS | [ENSG00000223552](https://www.ensembl.org/id/ENSG00000223552) | CCR5 antisense RNA [Source:HGNC Symbol;Acc:HGNC:54398] | 6,78 |
| CCR6 | [ENSG00000112486](https://www.ensembl.org/id/ENSG00000112486) | C-C motif chemokine receptor 6 [Source:HGNC Symbol;Acc:HGNC:1607] | 5,34 |
| CD177 | [ENSG00000204936](https://www.ensembl.org/id/ENSG00000204936) | CD177 molecule [Source:HGNC Symbol;Acc:HGNC:30072] | -4,56 |
| CD200 | [ENSG00000091972](https://www.ensembl.org/id/ENSG00000091972) | CD200 molecule [Source:HGNC Symbol;Acc:HGNC:7203] | -1,17 |
| CD300C | [ENSG00000167850](https://www.ensembl.org/id/ENSG00000167850) | CD300c molecule [Source:HGNC Symbol;Acc:HGNC:19320] | 5,52 |
| CD300E | [ENSG00000186407](https://www.ensembl.org/id/ENSG00000186407) | CD300e molecule [Source:HGNC Symbol;Acc:HGNC:28874] | 4,82 |
| CD3E | [ENSG00000198851](https://www.ensembl.org/id/ENSG00000198851) | CD3 epsilon subunit of T-cell receptor complex [Source:HGNC Symbol;Acc:HGNC:1674] | 2,18 |
| CD3G | [ENSG00000160654](https://www.ensembl.org/id/ENSG00000160654) | CD3 gamma subunit of T-cell receptor complex [Source:HGNC Symbol;Acc:HGNC:1675] | 2,66 |
| CD48 | [ENSG00000117091](https://www.ensembl.org/id/ENSG00000117091) | CD48 molecule [Source:HGNC Symbol;Acc:HGNC:1683] | 3,33 |
| CD5 | [ENSG00000110448](https://www.ensembl.org/id/ENSG00000110448) | CD5 molecule [Source:HGNC Symbol;Acc:HGNC:1685] | 2,25 |
| CD52 | [ENSG00000169442](https://www.ensembl.org/id/ENSG00000169442) | CD52 molecule [Source:HGNC Symbol;Acc:HGNC:1804] | 2,36 |
| CD53 | [ENSG00000143119](https://www.ensembl.org/id/ENSG00000143119) | CD53 molecule [Source:HGNC Symbol;Acc:HGNC:1686] | 2,56 |
| CD69 | [ENSG00000110848](https://www.ensembl.org/id/ENSG00000110848) | CD69 molecule [Source:HGNC Symbol;Acc:HGNC:1694] | 2,17 |
| CD86 | [ENSG00000114013](https://www.ensembl.org/id/ENSG00000114013) | CD86 molecule [Source:HGNC Symbol;Acc:HGNC:1705] | 2,27 |
| CD8A | [ENSG00000153563](https://www.ensembl.org/id/ENSG00000153563) | CD8a molecule [Source:HGNC Symbol;Acc:HGNC:1706] | 2,66 |
| CDC20 | [ENSG00000117399](https://www.ensembl.org/id/ENSG00000117399) | cell division cycle 20 [Source:HGNC Symbol;Acc:HGNC:1723] | -3,13 |
| CDC42BPG | [ENSG00000171219](https://www.ensembl.org/id/ENSG00000171219) | CDC42 binding protein kinase gamma [Source:HGNC Symbol;Acc:HGNC:29829] | -2,54 |
| CDHR4 | [ENSG00000187492](https://www.ensembl.org/id/ENSG00000187492) | cadherin related family member 4 [Source:HGNC Symbol;Acc:HGNC:34527] | -2,77 |
| CDHR5 | [ENSG00000099834](https://www.ensembl.org/id/ENSG00000099834) | cadherin related family member 5 [Source:HGNC Symbol;Acc:HGNC:7521] | -2,77 |
| CDIN1 | [ENSG00000186073](https://www.ensembl.org/id/ENSG00000186073) | CDAN1 interacting nuclease 1 [Source:HGNC Symbol;Acc:HGNC:26929] | 1,27 |
| CDK11A | [ENSG00000008128](https://www.ensembl.org/id/ENSG00000008128) | cyclin dependent kinase 11A [Source:HGNC Symbol;Acc:HGNC:1730] | 0,82 |
| CDNF | [ENSG00000185267](https://www.ensembl.org/id/ENSG00000185267) | cerebral dopamine neurotrophic factor [Source:HGNC Symbol;Acc:HGNC:24913] | 2,33 |
| CDR1 | [ENSG00000288642](https://www.ensembl.org/id/ENSG00000288642) | cerebellar degeneration related protein 1 [Source:HGNC Symbol;Acc:HGNC:1798] | -6,48 |
| CDR2-DT | [ENSG00000260790](https://www.ensembl.org/id/ENSG00000260790) | CDR2 divergent transcript [Source:HGNC Symbol;Acc:HGNC:55381] | 6,63 |
| CDR2L | [ENSG00000109089](https://www.ensembl.org/id/ENSG00000109089) | cerebellar degeneration related protein 2 like [Source:HGNC Symbol;Acc:HGNC:29999] | -1,09 |
| CEACAM4 | [ENSG00000105352](https://www.ensembl.org/id/ENSG00000105352) | CEA cell adhesion molecule 4 [Source:HGNC Symbol;Acc:HGNC:1816] | 3,79 |
| CEACAM8 | [ENSG00000124469](https://www.ensembl.org/id/ENSG00000124469) | CEA cell adhesion molecule 8 [Source:HGNC Symbol;Acc:HGNC:1820] | 6,99 |
| CEBPA | [ENSG00000245848](https://www.ensembl.org/id/ENSG00000245848) | CCAAT enhancer binding protein alpha [Source:HGNC Symbol;Acc:HGNC:1833] | -1,85 |
| CELF2-AS1 | [ENSG00000181800](https://www.ensembl.org/id/ENSG00000181800) | CELF2 antisense RNA 1 [Source:HGNC Symbol;Acc:HGNC:23515] | 3,26 |
| CEMIP2 | [ENSG00000135048](https://www.ensembl.org/id/ENSG00000135048) | cell migration inducing hyaluronidase 2 [Source:HGNC Symbol;Acc:HGNC:11869] | -1,70 |
| CENPM | [ENSG00000100162](https://www.ensembl.org/id/ENSG00000100162) | centromere protein M [Source:HGNC Symbol;Acc:HGNC:18352] | 2,81 |
| CENPS-CORT | [ENSG00000251503](https://www.ensembl.org/id/ENSG00000251503) | CENPS-CORT readthrough [Source:HGNC Symbol;Acc:HGNC:38843] | -2,94 |
| CEP85 | [ENSG00000130695](https://www.ensembl.org/id/ENSG00000130695) | centrosomal protein 85 [Source:HGNC Symbol;Acc:HGNC:25309] | 1,82 |
| CERS1 | [ENSG00000223802](https://www.ensembl.org/id/ENSG00000223802) | ceramide synthase 1 [Source:HGNC Symbol;Acc:HGNC:14253] | 1,89 |
| CFAP206 | [ENSG00000272514](https://www.ensembl.org/id/ENSG00000272514) | cilia and flagella associated protein 206 [Source:HGNC Symbol;Acc:HGNC:21405] | 2,97 |
| CFAP221 | [ENSG00000163075](https://www.ensembl.org/id/ENSG00000163075) | cilia and flagella associated protein 221 [Source:HGNC Symbol;Acc:HGNC:33720] | -3,92 |
| CFAP54 | [ENSG00000188596](https://www.ensembl.org/id/ENSG00000188596) | cilia and flagella associated protein 54 [Source:HGNC Symbol;Acc:HGNC:26456] | -6,72 |
| CFAP57 | [ENSG00000243710](https://www.ensembl.org/id/ENSG00000243710) | cilia and flagella associated protein 57 [Source:HGNC Symbol;Acc:HGNC:26485] | -2,58 |
| CFAP61 | [ENSG00000089101](https://www.ensembl.org/id/ENSG00000089101) | cilia and flagella associated protein 61 [Source:HGNC Symbol;Acc:HGNC:15872] | 3,67 |
| CFAP95 | [ENSG00000204711](https://www.ensembl.org/id/ENSG00000204711) | cilia and flagella associated protein 95 [Source:HGNC Symbol;Acc:HGNC:31422] | 5,67 |
| CFB | [ENSG00000243649](https://www.ensembl.org/id/ENSG00000243649) | complement factor B [Source:HGNC Symbol;Acc:HGNC:1037] | -1,65 |
| CFC1 | [ENSG00000136698](https://www.ensembl.org/id/ENSG00000136698) | cripto, FRL-1, cryptic family 1 [Source:HGNC Symbol;Acc:HGNC:18292] | -7,19 |
| CFI | [ENSG00000205403](https://www.ensembl.org/id/ENSG00000205403) | complement factor I [Source:HGNC Symbol;Acc:HGNC:5394] | -1,40 |
| CFL2 | [ENSG00000165410](https://www.ensembl.org/id/ENSG00000165410) | cofilin 2 [Source:HGNC Symbol;Acc:HGNC:1875] | 1,95 |
| CGN | [ENSG00000143375](https://www.ensembl.org/id/ENSG00000143375) | cingulin [Source:HGNC Symbol;Acc:HGNC:17429] | -6,41 |
| CHAC1 | [ENSG00000128965](https://www.ensembl.org/id/ENSG00000128965) | ChaC glutathione specific gamma-glutamylcyclotransferase 1 [Source:HGNC Symbol;Acc:HGNC:28680] | -2,80 |
| CHCHD10 | [ENSG00000250479](https://www.ensembl.org/id/ENSG00000250479) | coiled-coil-helix-coiled-coil-helix domain containing 10 [Source:HGNC Symbol;Acc:HGNC:15559] | 1,80 |
| CHCHD3 | [ENSG00000106554](https://www.ensembl.org/id/ENSG00000106554) | coiled-coil-helix-coiled-coil-helix domain containing 3 [Source:HGNC Symbol;Acc:HGNC:21906] | 1,47 |
| CHCHD4 | [ENSG00000163528](https://www.ensembl.org/id/ENSG00000163528) | coiled-coil-helix-coiled-coil-helix domain containing 4 [Source:HGNC Symbol;Acc:HGNC:26467] | 1,13 |
| CHCHD5 | [ENSG00000125611](https://www.ensembl.org/id/ENSG00000125611) | coiled-coil-helix-coiled-coil-helix domain containing 5 [Source:HGNC Symbol;Acc:HGNC:17840] | 1,04 |
| CHCHD7 | [ENSG00000170791](https://www.ensembl.org/id/ENSG00000170791) | coiled-coil-helix-coiled-coil-helix domain containing 7 [Source:HGNC Symbol;Acc:HGNC:28314] | 0,93 |
| CHIT1 | [ENSG00000133063](https://www.ensembl.org/id/ENSG00000133063) | chitinase 1 [Source:HGNC Symbol;Acc:HGNC:1936] | 3,10 |
| CHODL | [ENSG00000154645](https://www.ensembl.org/id/ENSG00000154645) | chondrolectin [Source:HGNC Symbol;Acc:HGNC:17807] | 1,59 |
| CHRDL2 | [ENSG00000054938](https://www.ensembl.org/id/ENSG00000054938) | chordin like 2 [Source:HGNC Symbol;Acc:HGNC:24168] | 2,28 |
| CHRNB1 | [ENSG00000170175](https://www.ensembl.org/id/ENSG00000170175) | cholinergic receptor nicotinic beta 1 subunit [Source:HGNC Symbol;Acc:HGNC:1961] | 1,70 |
| CHST5 | [ENSG00000135702](https://www.ensembl.org/id/ENSG00000135702) | carbohydrate sulfotransferase 5 [Source:HGNC Symbol;Acc:HGNC:1973] | 3,06 |
| CIDEA | [ENSG00000176194](https://www.ensembl.org/id/ENSG00000176194) | cell death inducing DFFA like effector a [Source:HGNC Symbol;Acc:HGNC:1976] | -3,00 |
| CIDEC | [ENSG00000187288](https://www.ensembl.org/id/ENSG00000187288) | cell death inducing DFFA like effector c [Source:HGNC Symbol;Acc:HGNC:24229] | -2,92 |
| CISD1 | [ENSG00000122873](https://www.ensembl.org/id/ENSG00000122873) | CDGSH iron sulfur domain 1 [Source:HGNC Symbol;Acc:HGNC:30880] | 1,50 |
| CKAP5 | [ENSG00000175216](https://www.ensembl.org/id/ENSG00000175216) | cytoskeleton associated protein 5 [Source:HGNC Symbol;Acc:HGNC:28959] | 1,05 |
| CKMT1A | [ENSG00000223572](https://www.ensembl.org/id/ENSG00000223572) | creatine kinase, mitochondrial 1A [Source:HGNC Symbol;Acc:HGNC:31736] | -6,21 |
| CKMT1B | [ENSG00000237289](https://www.ensembl.org/id/ENSG00000237289) | creatine kinase, mitochondrial 1B [Source:HGNC Symbol;Acc:HGNC:1995] | -5,19 |
| CKMT2 | [ENSG00000131730](https://www.ensembl.org/id/ENSG00000131730) | creatine kinase, mitochondrial 2 [Source:HGNC Symbol;Acc:HGNC:1996] | 2,71 |
| CLC | [ENSG00000105205](https://www.ensembl.org/id/ENSG00000105205) | Charcot-Leyden crystal galectin [Source:HGNC Symbol;Acc:HGNC:2014] | 3,63 |
| CLCNKA | [ENSG00000186510](https://www.ensembl.org/id/ENSG00000186510) | chloride voltage-gated channel Ka [Source:HGNC Symbol;Acc:HGNC:2026] | -2,89 |
| CLDN1 | [ENSG00000163347](https://www.ensembl.org/id/ENSG00000163347) | claudin 1 [Source:HGNC Symbol;Acc:HGNC:2032] | -2,01 |
| CLDN15 | [ENSG00000106404](https://www.ensembl.org/id/ENSG00000106404) | claudin 15 [Source:HGNC Symbol;Acc:HGNC:2036] | -1,75 |
| CLEC18A | [ENSG00000157322](https://www.ensembl.org/id/ENSG00000157322) | C-type lectin domain family 18 member A [Source:HGNC Symbol;Acc:HGNC:30388] | -1,70 |
| CLEC1B | [ENSG00000165682](https://www.ensembl.org/id/ENSG00000165682) | C-type lectin domain family 1 member B [Source:HGNC Symbol;Acc:HGNC:24356] | 7,10 |
| CLEC2L | [ENSG00000236279](https://www.ensembl.org/id/ENSG00000236279) | C-type lectin domain family 2 member L [Source:HGNC Symbol;Acc:HGNC:21969] | 3,18 |
| CLEC3A | [ENSG00000166509](https://www.ensembl.org/id/ENSG00000166509) | C-type lectin domain family 3 member A [Source:HGNC Symbol;Acc:HGNC:2052] | -3,22 |
| CLEC4E | [ENSG00000166523](https://www.ensembl.org/id/ENSG00000166523) | C-type lectin domain family 4 member E [Source:HGNC Symbol;Acc:HGNC:14555] | 3,31 |
| CLEC5A | [ENSG00000258227](https://www.ensembl.org/id/ENSG00000258227) | C-type lectin domain containing 5A [Source:HGNC Symbol;Acc:HGNC:2054] | 2,77 |
| CLEC7A | [ENSG00000172243](https://www.ensembl.org/id/ENSG00000172243) | C-type lectin domain containing 7A [Source:HGNC Symbol;Acc:HGNC:14558] | 2,55 |
| CLEC9A | [ENSG00000197992](https://www.ensembl.org/id/ENSG00000197992) | C-type lectin domain containing 9A [Source:HGNC Symbol;Acc:HGNC:26705] | 4,74 |
| CLGN | [ENSG00000153132](https://www.ensembl.org/id/ENSG00000153132) | calmegin [Source:HGNC Symbol;Acc:HGNC:2060] | 2,41 |
| CLIC3 | [ENSG00000169583](https://www.ensembl.org/id/ENSG00000169583) | chloride intracellular channel 3 [Source:HGNC Symbol;Acc:HGNC:2064] | -2,00 |
| CLIC5 | [ENSG00000112782](https://www.ensembl.org/id/ENSG00000112782) | chloride intracellular channel 5 [Source:HGNC Symbol;Acc:HGNC:13517] | 2,03 |
| CLMP | [ENSG00000166250](https://www.ensembl.org/id/ENSG00000166250) | CXADR like membrane protein [Source:HGNC Symbol;Acc:HGNC:24039] | -1,47 |
| CLPX | [ENSG00000166855](https://www.ensembl.org/id/ENSG00000166855) | caseinolytic mitochondrial matrix peptidase chaperone subunit X [Source:HGNC Symbol;Acc:HGNC:2088] | 1,04 |
| CMTM2 | [ENSG00000140932](https://www.ensembl.org/id/ENSG00000140932) | CKLF like MARVEL transmembrane domain containing 2 [Source:HGNC Symbol;Acc:HGNC:19173] | 4,06 |
| CNIH2 | [ENSG00000174871](https://www.ensembl.org/id/ENSG00000174871) | cornichon family AMPA receptor auxiliary protein 2 [Source:HGNC Symbol;Acc:HGNC:28744] | -2,19 |
| CNMD | [ENSG00000136110](https://www.ensembl.org/id/ENSG00000136110) | chondromodulin [Source:HGNC Symbol;Acc:HGNC:17005] | 5,98 |
| CNNM2 | [ENSG00000148842](https://www.ensembl.org/id/ENSG00000148842) | cyclin and CBS domain divalent metal cation transport mediator 2 [Source:HGNC Symbol;Acc:HGNC:103] | -1,38 |
| CNNM4 | [ENSG00000158158](https://www.ensembl.org/id/ENSG00000158158) | cyclin and CBS domain divalent metal cation transport mediator 4 [Source:HGNC Symbol;Acc:HGNC:105] | 1,75 |
| CNOT7 | [ENSG00000198791](https://www.ensembl.org/id/ENSG00000198791) | CCR4-NOT transcription complex subunit 7 [Source:HGNC Symbol;Acc:HGNC:14101] | 0,83 |
| COA8 | [ENSG00000256053](https://www.ensembl.org/id/ENSG00000256053) | cytochrome c oxidase assembly factor 8 [Source:HGNC Symbol;Acc:HGNC:20492] | 0,99 |
| COBLL1 | [ENSG00000082438](https://www.ensembl.org/id/ENSG00000082438) | cordon-bleu WH2 repeat protein like 1 [Source:HGNC Symbol;Acc:HGNC:23571] | -1,07 |
| COL17A1 | [ENSG00000065618](https://www.ensembl.org/id/ENSG00000065618) | collagen type XVII alpha 1 chain [Source:HGNC Symbol;Acc:HGNC:2194] | -3,16 |
| COL22A1 | [ENSG00000169436](https://www.ensembl.org/id/ENSG00000169436) | collagen type XXII alpha 1 chain [Source:HGNC Symbol;Acc:HGNC:22989] | 5,48 |
| COL6A6 | [ENSG00000206384](https://www.ensembl.org/id/ENSG00000206384) | collagen type VI alpha 6 chain [Source:HGNC Symbol;Acc:HGNC:27023] | -3,71 |
| COL9A2 | [ENSG00000049089](https://www.ensembl.org/id/ENSG00000049089) | collagen type IX alpha 2 chain [Source:HGNC Symbol;Acc:HGNC:2218] | -1,45 |
| COLGALT2 | [ENSG00000198756](https://www.ensembl.org/id/ENSG00000198756) | collagen beta(1-O)galactosyltransferase 2 [Source:HGNC Symbol;Acc:HGNC:16790] | 1,62 |
| COLQ | [ENSG00000206561](https://www.ensembl.org/id/ENSG00000206561) | collagen like tail subunit of asymmetric acetylcholinesterase [Source:HGNC Symbol;Acc:HGNC:2226] | 1,19 |
| COMMD3-BMI1 | [ENSG00000269897](https://www.ensembl.org/id/ENSG00000269897) | COMMD3-BMI1 readthrough [Source:HGNC Symbol;Acc:HGNC:48326] | 7,63 |
| COMMD9 | [ENSG00000110442](https://www.ensembl.org/id/ENSG00000110442) | COMM domain containing 9 [Source:HGNC Symbol;Acc:HGNC:25014] | 1,24 |
| COMP | [ENSG00000105664](https://www.ensembl.org/id/ENSG00000105664) | cartilage oligomeric matrix protein [Source:HGNC Symbol;Acc:HGNC:2227] | 1,25 |
| COPS4 | [ENSG00000138663](https://www.ensembl.org/id/ENSG00000138663) | COP9 signalosome subunit 4 [Source:HGNC Symbol;Acc:HGNC:16702] | 1,06 |
| COQ10A | [ENSG00000135469](https://www.ensembl.org/id/ENSG00000135469) | coenzyme Q10A [Source:HGNC Symbol;Acc:HGNC:26515] | 2,26 |
| COQ3 | [ENSG00000132423](https://www.ensembl.org/id/ENSG00000132423) | coenzyme Q3, methyltransferase [Source:HGNC Symbol;Acc:HGNC:18175] | 1,70 |
| COQ5 | [ENSG00000110871](https://www.ensembl.org/id/ENSG00000110871) | coenzyme Q5, methyltransferase [Source:HGNC Symbol;Acc:HGNC:28722] | 1,30 |
| COQ7 | [ENSG00000167186](https://www.ensembl.org/id/ENSG00000167186) | coenzyme Q7, hydroxylase [Source:HGNC Symbol;Acc:HGNC:2244] | 1,02 |
| COQ7-DT | [ENSG00000261465](https://www.ensembl.org/id/ENSG00000261465) | COQ7 divergent transcript [Source:HGNC Symbol;Acc:HGNC:55362] | 3,25 |
| COQ8A | [ENSG00000163050](https://www.ensembl.org/id/ENSG00000163050) | coenzyme Q8A [Source:HGNC Symbol;Acc:HGNC:16812] | 2,21 |
| COQ9 | [ENSG00000088682](https://www.ensembl.org/id/ENSG00000088682) | coenzyme Q9 [Source:HGNC Symbol;Acc:HGNC:25302] | 1,52 |
| CORO1A | [ENSG00000102879](https://www.ensembl.org/id/ENSG00000102879) | coronin 1A [Source:HGNC Symbol;Acc:HGNC:2252] | 2,09 |
| CORO6 | [ENSG00000167549](https://www.ensembl.org/id/ENSG00000167549) | coronin 6 [Source:HGNC Symbol;Acc:HGNC:21356] | 1,77 |
| COX10 | [ENSG00000006695](https://www.ensembl.org/id/ENSG00000006695) | cytochrome c oxidase assembly factor heme A:farnesyltransferase COX10 [Source:HGNC Symbol;Acc:HGNC:2260] | 1,56 |
| COX5A | [ENSG00000178741](https://www.ensembl.org/id/ENSG00000178741) | cytochrome c oxidase subunit 5A [Source:HGNC Symbol;Acc:HGNC:2267] | 1,87 |
| COX5B | [ENSG00000135940](https://www.ensembl.org/id/ENSG00000135940) | cytochrome c oxidase subunit 5B [Source:HGNC Symbol;Acc:HGNC:2269] | 1,51 |
| COX6B1 | [ENSG00000126267](https://www.ensembl.org/id/ENSG00000126267) | cytochrome c oxidase subunit 6B1 [Source:HGNC Symbol;Acc:HGNC:2280] | 1,40 |
| COX7A1 | [ENSG00000161281](https://www.ensembl.org/id/ENSG00000161281) | cytochrome c oxidase subunit 7A1 [Source:HGNC Symbol;Acc:HGNC:2287] | 1,48 |
| COX7B | [ENSG00000131174](https://www.ensembl.org/id/ENSG00000131174) | cytochrome c oxidase subunit 7B [Source:HGNC Symbol;Acc:HGNC:2291] | 1,41 |
| CPB1 | [ENSG00000153002](https://www.ensembl.org/id/ENSG00000153002) | carboxypeptidase B1 [Source:HGNC Symbol;Acc:HGNC:2299] | -2,66 |
| CPT1A | [ENSG00000110090](https://www.ensembl.org/id/ENSG00000110090) | carnitine palmitoyltransferase 1A [Source:HGNC Symbol;Acc:HGNC:2328] | -1,01 |
| CPT1B | [ENSG00000205560](https://www.ensembl.org/id/ENSG00000205560) | carnitine palmitoyltransferase 1B [Source:HGNC Symbol;Acc:HGNC:2329] | 2,56 |
| CPT2 | [ENSG00000157184](https://www.ensembl.org/id/ENSG00000157184) | carnitine palmitoyltransferase 2 [Source:HGNC Symbol;Acc:HGNC:2330] | 1,34 |
| CPVL | [ENSG00000106066](https://www.ensembl.org/id/ENSG00000106066) | carboxypeptidase vitellogenic like [Source:HGNC Symbol;Acc:HGNC:14399] | 1,42 |
| CRAT | [ENSG00000095321](https://www.ensembl.org/id/ENSG00000095321) | carnitine O-acetyltransferase [Source:HGNC Symbol;Acc:HGNC:2342] | 1,52 |
| CRB2 | [ENSG00000148204](https://www.ensembl.org/id/ENSG00000148204) | crumbs cell polarity complex component 2 [Source:HGNC Symbol;Acc:HGNC:18688] | -3,26 |
| CREB5 | [ENSG00000146592](https://www.ensembl.org/id/ENSG00000146592) | cAMP responsive element binding protein 5 [Source:HGNC Symbol;Acc:HGNC:16844] | -1,35 |
| CRHR2 | [ENSG00000106113](https://www.ensembl.org/id/ENSG00000106113) | corticotropin releasing hormone receptor 2 [Source:HGNC Symbol;Acc:HGNC:2358] | 2,31 |
| CRY1 | [ENSG00000008405](https://www.ensembl.org/id/ENSG00000008405) | cryptochrome circadian regulator 1 [Source:HGNC Symbol;Acc:HGNC:2384] | -1,66 |
| CRYAB | [ENSG00000109846](https://www.ensembl.org/id/ENSG00000109846) | crystallin alpha B [Source:HGNC Symbol;Acc:HGNC:2389] | 1,78 |
| CRYBB3 | [ENSG00000100053](https://www.ensembl.org/id/ENSG00000100053) | crystallin beta B3 [Source:HGNC Symbol;Acc:HGNC:2400] | -6,85 |
| CRYBG2 | [ENSG00000176092](https://www.ensembl.org/id/ENSG00000176092) | crystallin beta-gamma domain containing 2 [Source:HGNC Symbol;Acc:HGNC:17295] | 5,30 |
| CRYGN | [ENSG00000127377](https://www.ensembl.org/id/ENSG00000127377) | crystallin gamma N [Source:HGNC Symbol;Acc:HGNC:20458] | -2,05 |
| CRYGS | [ENSG00000213139](https://www.ensembl.org/id/ENSG00000213139) | crystallin gamma S [Source:HGNC Symbol;Acc:HGNC:2417] | -1,67 |
| CSF3R | [ENSG00000119535](https://www.ensembl.org/id/ENSG00000119535) | colony stimulating factor 3 receptor [Source:HGNC Symbol;Acc:HGNC:2439] | 3,28 |
| CSN1S1 | [ENSG00000126545](https://www.ensembl.org/id/ENSG00000126545) | casein alpha s1 [Source:HGNC Symbol;Acc:HGNC:2445] | -5,24 |
| CSN2 | [ENSG00000135222](https://www.ensembl.org/id/ENSG00000135222) | casein beta [Source:HGNC Symbol;Acc:HGNC:2447] | 6,20 |
| CT55 | [ENSG00000169551](https://www.ensembl.org/id/ENSG00000169551) | cancer/testis antigen 55 [Source:HGNC Symbol;Acc:HGNC:26047] | 6,02 |
| CTAGE9 | [ENSG00000236761](https://www.ensembl.org/id/ENSG00000236761) | CTAGE family member 9 [Source:HGNC Symbol;Acc:HGNC:37275] | -5,13 |
| CTDNEP1 | [ENSG00000175826](https://www.ensembl.org/id/ENSG00000175826) | CTD nuclear envelope phosphatase 1 [Source:HGNC Symbol;Acc:HGNC:19085] | 1,19 |
| CTLA4 | [ENSG00000163599](https://www.ensembl.org/id/ENSG00000163599) | cytotoxic T-lymphocyte associated protein 4 [Source:HGNC Symbol;Acc:HGNC:2505] | 4,11 |
| CTNNA3 | [ENSG00000183230](https://www.ensembl.org/id/ENSG00000183230) | catenin alpha 3 [Source:HGNC Symbol;Acc:HGNC:2511] | 1,56 |
| CUTC | [ENSG00000119929](https://www.ensembl.org/id/ENSG00000119929) | cutC copper transporter [Source:HGNC Symbol;Acc:HGNC:24271] | 2,08 |
| CUX2 | [ENSG00000111249](https://www.ensembl.org/id/ENSG00000111249) | cut like homeobox 2 [Source:HGNC Symbol;Acc:HGNC:19347] | 2,92 |
| CUZD1 | [ENSG00000138161](https://www.ensembl.org/id/ENSG00000138161) | CUB and zona pellucida like domains 1 [Source:HGNC Symbol;Acc:HGNC:17937] | 2,65 |
| CX3CR1 | [ENSG00000168329](https://www.ensembl.org/id/ENSG00000168329) | C-X3-C motif chemokine receptor 1 [Source:HGNC Symbol;Acc:HGNC:2558] | 3,50 |
| CXADR | [ENSG00000154639](https://www.ensembl.org/id/ENSG00000154639) | CXADR Ig-like cell adhesion molecule [Source:HGNC Symbol;Acc:HGNC:2559] | -2,96 |
| CXCL13 | [ENSG00000156234](https://www.ensembl.org/id/ENSG00000156234) | C-X-C motif chemokine ligand 13 [Source:HGNC Symbol;Acc:HGNC:10639] | -4,80 |
| CXCL5 | [ENSG00000163735](https://www.ensembl.org/id/ENSG00000163735) | C-X-C motif chemokine ligand 5 [Source:HGNC Symbol;Acc:HGNC:10642] | 3,13 |
| CXCR1 | [ENSG00000163464](https://www.ensembl.org/id/ENSG00000163464) | C-X-C motif chemokine receptor 1 [Source:HGNC Symbol;Acc:HGNC:6026] | 4,41 |
| CXCR2 | [ENSG00000180871](https://www.ensembl.org/id/ENSG00000180871) | C-X-C motif chemokine receptor 2 [Source:HGNC Symbol;Acc:HGNC:6027] | 3,97 |
| CXCR4 | [ENSG00000121966](https://www.ensembl.org/id/ENSG00000121966) | C-X-C motif chemokine receptor 4 [Source:HGNC Symbol;Acc:HGNC:2561] | 2,75 |
| CYB5R1 | [ENSG00000159348](https://www.ensembl.org/id/ENSG00000159348) | cytochrome b5 reductase 1 [Source:HGNC Symbol;Acc:HGNC:13397] | 2,17 |
| CYC1 | [ENSG00000179091](https://www.ensembl.org/id/ENSG00000179091) | cytochrome c1 [Source:HGNC Symbol;Acc:HGNC:2579] | 1,66 |
| CYP26B1 | [ENSG00000003137](https://www.ensembl.org/id/ENSG00000003137) | cytochrome P450 family 26 subfamily B member 1 [Source:HGNC Symbol;Acc:HGNC:20581] | -1,27 |
| CYP26C1 | [ENSG00000187553](https://www.ensembl.org/id/ENSG00000187553) | cytochrome P450 family 26 subfamily C member 1 [Source:HGNC Symbol;Acc:HGNC:20577] | 6,03 |
| CYP2A6 | [ENSG00000255974](https://www.ensembl.org/id/ENSG00000255974) | cytochrome P450 family 2 subfamily A member 6 [Source:HGNC Symbol;Acc:HGNC:2610] | -3,80 |
| CYP4A11 | [ENSG00000187048](https://www.ensembl.org/id/ENSG00000187048) | cytochrome P450 family 4 subfamily A member 11 [Source:HGNC Symbol;Acc:HGNC:2642] | 3,81 |
| CYP4F26P | [ENSG00000226562](https://www.ensembl.org/id/ENSG00000226562) | cytochrome P450 family 4 subfamily F member 26, pseudogene [Source:HGNC Symbol;Acc:HGNC:39948] | 3,77 |
| CYP4F29P | [ENSG00000290541](https://www.ensembl.org/id/ENSG00000290541) | cytochrome P450 family 4 subfamily F member 29, pseudogene [Source:NCBI gene (formerly Entrezgene);Acc:54055] | -3,00 |
| CYTIP | [ENSG00000115165](https://www.ensembl.org/id/ENSG00000115165) | cytohesin 1 interacting protein [Source:HGNC Symbol;Acc:HGNC:9506] | 2,30 |
| DACH2 | [ENSG00000126733](https://www.ensembl.org/id/ENSG00000126733) | dachshund family transcription factor 2 [Source:HGNC Symbol;Acc:HGNC:16814] | 3,67 |
| DAPP1 | [ENSG00000070190](https://www.ensembl.org/id/ENSG00000070190) | dual adaptor of phosphotyrosine and 3-phosphoinositides 1 [Source:HGNC Symbol;Acc:HGNC:16500] | 2,49 |
| DBT | [ENSG00000137992](https://www.ensembl.org/id/ENSG00000137992) | dihydrolipoamide branched chain transacylase E2 [Source:HGNC Symbol;Acc:HGNC:2698] | 1,17 |
| DCAF11 | [ENSG00000100897](https://www.ensembl.org/id/ENSG00000100897) | DDB1 and CUL4 associated factor 11 [Source:HGNC Symbol;Acc:HGNC:20258] | 1,24 |
| DCAF6 | [ENSG00000143164](https://www.ensembl.org/id/ENSG00000143164) | DDB1 and CUL4 associated factor 6 [Source:HGNC Symbol;Acc:HGNC:30002] | 1,83 |
| DCHS1-AS1 | [ENSG00000255390](https://www.ensembl.org/id/ENSG00000255390) | DCHS1 antisense RNA 1 [Source:HGNC Symbol;Acc:HGNC:40650] | -1,44 |
| DCLK2 | [ENSG00000170390](https://www.ensembl.org/id/ENSG00000170390) | doublecortin like kinase 2 [Source:HGNC Symbol;Acc:HGNC:19002] | -1,07 |
| DCPS | [ENSG00000110063](https://www.ensembl.org/id/ENSG00000110063) | decapping enzyme, scavenger [Source:HGNC Symbol;Acc:HGNC:29812] | 1,10 |
| DCUN1D2 | [ENSG00000150401](https://www.ensembl.org/id/ENSG00000150401) | defective in cullin neddylation 1 domain containing 2 [Source:HGNC Symbol;Acc:HGNC:20328] | 1,85 |
| DDN | [ENSG00000181418](https://www.ensembl.org/id/ENSG00000181418) | dendrin [Source:HGNC Symbol;Acc:HGNC:24458] | 2,93 |
| DDX11L16_1 | [ENSG00000290824](https://www.ensembl.org/id/ENSG00000290824) | DEAD/H-box helicase 11 like 16 (pseudogene) [Source:NCBI gene (formerly Entrezgene);Acc:727856] | 4,16 |
| DDX11L16_2 | [ENSG00000290355](https://www.ensembl.org/id/ENSG00000290355) | DEAD/H-box helicase 11 like 16 (pseudogene) [Source:NCBI gene (formerly Entrezgene);Acc:727856] | 5,99 |
| DDX39A | [ENSG00000123136](https://www.ensembl.org/id/ENSG00000123136) | DExD-box helicase 39A [Source:HGNC Symbol;Acc:HGNC:17821] | -1,03 |
| DECR1 | [ENSG00000104325](https://www.ensembl.org/id/ENSG00000104325) | 2,4-dienoyl-CoA reductase 1 [Source:HGNC Symbol;Acc:HGNC:2753] | 1,12 |
| DEFA1 | [ENSG00000206047](https://www.ensembl.org/id/ENSG00000206047) | defensin alpha 1 [Source:HGNC Symbol;Acc:HGNC:2761] | -7,39 |
| DEFA1B | [ENSG00000240247](https://www.ensembl.org/id/ENSG00000240247) | defensin alpha 1B [Source:HGNC Symbol;Acc:HGNC:33596] | 2,83 |
| DGAT2 | [ENSG00000062282](https://www.ensembl.org/id/ENSG00000062282) | diacylglycerol O-acyltransferase 2 [Source:HGNC Symbol;Acc:HGNC:16940] | -2,06 |
| DGCR6 | [ENSG00000183628](https://www.ensembl.org/id/ENSG00000183628) | DiGeorge syndrome critical region gene 6 [Source:HGNC Symbol;Acc:HGNC:2846] | 2,25 |
| DGKI | [ENSG00000157680](https://www.ensembl.org/id/ENSG00000157680) | diacylglycerol kinase iota [Source:HGNC Symbol;Acc:HGNC:2855] | -2,92 |
| DGKK | [ENSG00000274588](https://www.ensembl.org/id/ENSG00000274588) | diacylglycerol kinase kappa [Source:HGNC Symbol;Acc:HGNC:32395] | 5,21 |
| DGLUCY | [ENSG00000133943](https://www.ensembl.org/id/ENSG00000133943) | D-glutamate cyclase [Source:HGNC Symbol;Acc:HGNC:20498] | 1,83 |
| DHDDS-AS1 | [ENSG00000225891](https://www.ensembl.org/id/ENSG00000225891) | DHDDS antisense RNA 1 [Source:HGNC Symbol;Acc:HGNC:40925] | 3,79 |
| DHTKD1 | [ENSG00000181192](https://www.ensembl.org/id/ENSG00000181192) | dehydrogenase E1 and transketolase domain containing 1 [Source:HGNC Symbol;Acc:HGNC:23537] | 1,55 |
| DIRAS1 | [ENSG00000176490](https://www.ensembl.org/id/ENSG00000176490) | DIRAS family GTPase 1 [Source:HGNC Symbol;Acc:HGNC:19127] | 3,04 |
| DIS3L | [ENSG00000166938](https://www.ensembl.org/id/ENSG00000166938) | DIS3 like exosome 3'-5' exoribonuclease [Source:HGNC Symbol;Acc:HGNC:28698] | 1,02 |
| DLAT | [ENSG00000150768](https://www.ensembl.org/id/ENSG00000150768) | dihydrolipoamide S-acetyltransferase [Source:HGNC Symbol;Acc:HGNC:2896] | 1,40 |
| DLD | [ENSG00000091140](https://www.ensembl.org/id/ENSG00000091140) | dihydrolipoamide dehydrogenase [Source:HGNC Symbol;Acc:HGNC:2898] | 1,60 |
| DMAC2L | [ENSG00000125375](https://www.ensembl.org/id/ENSG00000125375) | distal membrane arm assembly component 2 like [Source:HGNC Symbol;Acc:HGNC:18799] | 1,50 |
| DMKN | [ENSG00000161249](https://www.ensembl.org/id/ENSG00000161249) | dermokine [Source:HGNC Symbol;Acc:HGNC:25063] | -1,34 |
| DMRT2 | [ENSG00000173253](https://www.ensembl.org/id/ENSG00000173253) | doublesex and mab-3 related transcription factor 2 [Source:HGNC Symbol;Acc:HGNC:2935] | -2,31 |
| DMRTC1B | [ENSG00000184911](https://www.ensembl.org/id/ENSG00000184911) | DMRT like family C1B [Source:HGNC Symbol;Acc:HGNC:31686] | 4,84 |
| DNAH8-AS1 | [ENSG00000231150](https://www.ensembl.org/id/ENSG00000231150) | DNAH8 antisense RNA 1 [Source:HGNC Symbol;Acc:HGNC:40188] | 5,28 |
| DNAH9 | [ENSG00000007174](https://www.ensembl.org/id/ENSG00000007174) | dynein axonemal heavy chain 9 [Source:HGNC Symbol;Acc:HGNC:2953] | -2,88 |
| DNAJA3 | [ENSG00000103423](https://www.ensembl.org/id/ENSG00000103423) | DnaJ heat shock protein family (Hsp40) member A3 [Source:HGNC Symbol;Acc:HGNC:11808] | 1,15 |
| DNAJC12 | [ENSG00000108176](https://www.ensembl.org/id/ENSG00000108176) | DnaJ heat shock protein family (Hsp40) member C12 [Source:HGNC Symbol;Acc:HGNC:28908] | 1,92 |
| DNAJC19 | [ENSG00000205981](https://www.ensembl.org/id/ENSG00000205981) | DnaJ heat shock protein family (Hsp40) member C19 [Source:HGNC Symbol;Acc:HGNC:30528] | 0,97 |
| DNAJC24 | [ENSG00000170946](https://www.ensembl.org/id/ENSG00000170946) | DnaJ heat shock protein family (Hsp40) member C24 [Source:HGNC Symbol;Acc:HGNC:26979] | 0,96 |
| DNAJC5B | [ENSG00000147570](https://www.ensembl.org/id/ENSG00000147570) | DnaJ heat shock protein family (Hsp40) member C5 beta [Source:HGNC Symbol;Acc:HGNC:24138] | 3,34 |
| DNASE1L1 | [ENSG00000013563](https://www.ensembl.org/id/ENSG00000013563) | deoxyribonuclease 1 like 1 [Source:HGNC Symbol;Acc:HGNC:2957] | 1,55 |
| DNASE1L3 | [ENSG00000163687](https://www.ensembl.org/id/ENSG00000163687) | deoxyribonuclease 1 like 3 [Source:HGNC Symbol;Acc:HGNC:2959] | -2,52 |
| DND1 | [ENSG00000256453](https://www.ensembl.org/id/ENSG00000256453) | DND microRNA-mediated repression inhibitor 1 [Source:HGNC Symbol;Acc:HGNC:23799] | -6,73 |
| DNER | [ENSG00000187957](https://www.ensembl.org/id/ENSG00000187957) | delta/notch like EGF repeat containing [Source:HGNC Symbol;Acc:HGNC:24456] | 1,75 |
| DPF3 | [ENSG00000205683](https://www.ensembl.org/id/ENSG00000205683) | double PHD fingers 3 [Source:HGNC Symbol;Acc:HGNC:17427] | 1,26 |
| DPP10 | [ENSG00000175497](https://www.ensembl.org/id/ENSG00000175497) | dipeptidyl peptidase like 10 [Source:HGNC Symbol;Acc:HGNC:20823] | -5,23 |
| DSC3 | [ENSG00000134762](https://www.ensembl.org/id/ENSG00000134762) | desmocollin 3 [Source:HGNC Symbol;Acc:HGNC:3037] | -3,09 |
| DSCR8 | [ENSG00000198054](https://www.ensembl.org/id/ENSG00000198054) | Down syndrome critical region 8 [Source:HGNC Symbol;Acc:HGNC:16707] | 3,43 |
| DSP-AS1 | [ENSG00000261189](https://www.ensembl.org/id/ENSG00000261189) | DSP antisense RNA 1 [Source:HGNC Symbol;Acc:HGNC:56039] | -4,27 |
| DTX1 | [ENSG00000135144](https://www.ensembl.org/id/ENSG00000135144) | deltex E3 ubiquitin ligase 1 [Source:HGNC Symbol;Acc:HGNC:3060] | -2,13 |
| DUOXA1 | [ENSG00000140254](https://www.ensembl.org/id/ENSG00000140254) | dual oxidase maturation factor 1 [Source:HGNC Symbol;Acc:HGNC:26507] | -2,76 |
| DUS3L | [ENSG00000141994](https://www.ensembl.org/id/ENSG00000141994) | dihydrouridine synthase 3 like [Source:HGNC Symbol;Acc:HGNC:26920] | -0,84 |
| DUSP26 | [ENSG00000133878](https://www.ensembl.org/id/ENSG00000133878) | dual specificity phosphatase 26 [Source:HGNC Symbol;Acc:HGNC:28161] | 1,75 |
| DUXA | [ENSG00000258873](https://www.ensembl.org/id/ENSG00000258873) | double homeobox A [Source:HGNC Symbol;Acc:HGNC:32179] | -3,16 |
| DVL1 | [ENSG00000107404](https://www.ensembl.org/id/ENSG00000107404) | dishevelled segment polarity protein 1 [Source:HGNC Symbol;Acc:HGNC:3084] | 1,43 |
| EBF2 | [ENSG00000221818](https://www.ensembl.org/id/ENSG00000221818) | EBF transcription factor 2 [Source:HGNC Symbol;Acc:HGNC:19090] | -1,17 |
| EBF4 | [ENSG00000088881](https://www.ensembl.org/id/ENSG00000088881) | EBF family member 4 [Source:HGNC Symbol;Acc:HGNC:29278] | -1,10 |
| ECE2 | [ENSG00000145194](https://www.ensembl.org/id/ENSG00000145194) | endothelin converting enzyme 2 [Source:HGNC Symbol;Acc:HGNC:13275] | 4,73 |
| ECI1 | [ENSG00000167969](https://www.ensembl.org/id/ENSG00000167969) | enoyl-CoA delta isomerase 1 [Source:HGNC Symbol;Acc:HGNC:2703] | 1,21 |
| ECI2 | [ENSG00000198721](https://www.ensembl.org/id/ENSG00000198721) | enoyl-CoA delta isomerase 2 [Source:HGNC Symbol;Acc:HGNC:14601] | 1,34 |
| ECSIT | [ENSG00000130159](https://www.ensembl.org/id/ENSG00000130159) | ECSIT signaling integrator [Source:HGNC Symbol;Acc:HGNC:29548] | 0,98 |
| EDNRB | [ENSG00000136160](https://www.ensembl.org/id/ENSG00000136160) | endothelin receptor type B [Source:HGNC Symbol;Acc:HGNC:3180] | -1,02 |
| EFCAB8 | [ENSG00000215529](https://www.ensembl.org/id/ENSG00000215529) | EF-hand calcium binding domain 8 [Source:HGNC Symbol;Acc:HGNC:34532] | -2,75 |
| EFNB3 | [ENSG00000108947](https://www.ensembl.org/id/ENSG00000108947) | ephrin B3 [Source:HGNC Symbol;Acc:HGNC:3228] | -2,79 |
| EFR3B | [ENSG00000084710](https://www.ensembl.org/id/ENSG00000084710) | EFR3 homolog B [Source:HGNC Symbol;Acc:HGNC:29155] | 1,90 |
| EGFLAM | [ENSG00000164318](https://www.ensembl.org/id/ENSG00000164318) | EGF like, fibronectin type III and laminin G domains [Source:HGNC Symbol;Acc:HGNC:26810] | 1,34 |
| EGLN1 | [ENSG00000135766](https://www.ensembl.org/id/ENSG00000135766) | egl-9 family hypoxia inducible factor 1 [Source:HGNC Symbol;Acc:HGNC:1232] | 1,51 |
| EGOT | [ENSG00000235947](https://www.ensembl.org/id/ENSG00000235947) | eosinophil granule ontogeny transcript [Source:HGNC Symbol;Acc:HGNC:37129] | 5,67 |
| EHD4 | [ENSG00000103966](https://www.ensembl.org/id/ENSG00000103966) | EH domain containing 4 [Source:HGNC Symbol;Acc:HGNC:3245] | -1,11 |
| EHD4-AS1 | [ENSG00000259883](https://www.ensembl.org/id/ENSG00000259883) | EHD4 antisense RNA 1 [Source:HGNC Symbol;Acc:HGNC:51418] | 6,57 |
| EIF2B3 | [ENSG00000070785](https://www.ensembl.org/id/ENSG00000070785) | eukaryotic translation initiation factor 2B subunit gamma [Source:HGNC Symbol;Acc:HGNC:3259] | 1,09 |
| ELAVL4 | [ENSG00000162374](https://www.ensembl.org/id/ENSG00000162374) | ELAV like RNA binding protein 4 [Source:HGNC Symbol;Acc:HGNC:3315] | 3,95 |
| ELF4 | [ENSG00000102034](https://www.ensembl.org/id/ENSG00000102034) | E74 like ETS transcription factor 4 [Source:HGNC Symbol;Acc:HGNC:3319] | -1,02 |
| ELFN1 | [ENSG00000225968](https://www.ensembl.org/id/ENSG00000225968) | extracellular leucine rich repeat and fibronectin type III domain containing 1 [Source:HGNC Symbol;Acc:HGNC:33154] | -2,41 |
| ELMOD1 | [ENSG00000110675](https://www.ensembl.org/id/ENSG00000110675) | ELMO domain containing 1 [Source:HGNC Symbol;Acc:HGNC:25334] | 2,60 |
| ELN-AS1 | [ENSG00000232415](https://www.ensembl.org/id/ENSG00000232415) | ELN antisense RNA 1 [Source:HGNC Symbol;Acc:HGNC:40212] | -2,53 |
| EMC6 | [ENSG00000127774](https://www.ensembl.org/id/ENSG00000127774) | ER membrane protein complex subunit 6 [Source:HGNC Symbol;Acc:HGNC:28430] | 1,26 |
| EMC9 | [ENSG00000100908](https://www.ensembl.org/id/ENSG00000100908) | ER membrane protein complex subunit 9 [Source:HGNC Symbol;Acc:HGNC:20273] | 1,17 |
| EMSLR | [ENSG00000232445](https://www.ensembl.org/id/ENSG00000232445) | E2F1 mRNA stabilizing lncRNA [Source:HGNC Symbol;Acc:HGNC:54408] | -5,70 |
| ENDOG | [ENSG00000167136](https://www.ensembl.org/id/ENSG00000167136) | endonuclease G [Source:HGNC Symbol;Acc:HGNC:3346] | 1,69 |
| EPB41L4B | [ENSG00000095203](https://www.ensembl.org/id/ENSG00000095203) | erythrocyte membrane protein band 4.1 like 4B [Source:HGNC Symbol;Acc:HGNC:19818] | -2,15 |
| EPHX3 | [ENSG00000105131](https://www.ensembl.org/id/ENSG00000105131) | epoxide hydrolase 3 [Source:HGNC Symbol;Acc:HGNC:23760] | -1,95 |
| EPM2A | [ENSG00000112425](https://www.ensembl.org/id/ENSG00000112425) | EPM2A glucan phosphatase, laforin [Source:HGNC Symbol;Acc:HGNC:3413] | 1,29 |
| EPN3 | [ENSG00000049283](https://www.ensembl.org/id/ENSG00000049283) | epsin 3 [Source:HGNC Symbol;Acc:HGNC:18235] | -3,90 |
| EPS8L1 | [ENSG00000131037](https://www.ensembl.org/id/ENSG00000131037) | EPS8 like 1 [Source:HGNC Symbol;Acc:HGNC:21295] | -2,40 |
| ERVH48-1 | [ENSG00000233056](https://www.ensembl.org/id/ENSG00000233056) | endogenous retrovirus group 48 member 1 [Source:HGNC Symbol;Acc:HGNC:17216] | 3,20 |
| ESRRA | [ENSG00000173153](https://www.ensembl.org/id/ENSG00000173153) | estrogen related receptor alpha [Source:HGNC Symbol;Acc:HGNC:3471] | 1,67 |
| ESRRB | [ENSG00000119715](https://www.ensembl.org/id/ENSG00000119715) | estrogen related receptor beta [Source:HGNC Symbol;Acc:HGNC:3473] | 2,46 |
| ESYT3 | [ENSG00000158220](https://www.ensembl.org/id/ENSG00000158220) | extended synaptotagmin 3 [Source:HGNC Symbol;Acc:HGNC:24295] | -1,92 |
| ETFA | [ENSG00000140374](https://www.ensembl.org/id/ENSG00000140374) | electron transfer flavoprotein subunit alpha [Source:HGNC Symbol;Acc:HGNC:3481] | 1,42 |
| ETFDH | [ENSG00000171503](https://www.ensembl.org/id/ENSG00000171503) | electron transfer flavoprotein dehydrogenase [Source:HGNC Symbol;Acc:HGNC:3483] | 1,21 |
| ETFRF1 | [ENSG00000205707](https://www.ensembl.org/id/ENSG00000205707) | electron transfer flavoprotein regulatory factor 1 [Source:HGNC Symbol;Acc:HGNC:27052] | 0,99 |
| EVI2A | [ENSG00000126860](https://www.ensembl.org/id/ENSG00000126860) | ecotropic viral integration site 2A [Source:HGNC Symbol;Acc:HGNC:3499] | 2,33 |
| EVI2B | [ENSG00000185862](https://www.ensembl.org/id/ENSG00000185862) | ecotropic viral integration site 2B [Source:HGNC Symbol;Acc:HGNC:3500] | 2,59 |
| EVPL | [ENSG00000167880](https://www.ensembl.org/id/ENSG00000167880) | envoplakin [Source:HGNC Symbol;Acc:HGNC:3503] | -3,11 |
| EXOC3L2 | [ENSG00000283632](https://www.ensembl.org/id/ENSG00000283632) | exocyst complex component 3 like 2 [Source:HGNC Symbol;Acc:HGNC:30162] | -1,94 |
| EXOC6 | [ENSG00000138190](https://www.ensembl.org/id/ENSG00000138190) | exocyst complex component 6 [Source:HGNC Symbol;Acc:HGNC:23196] | 1,51 |
| EXOSC5 | [ENSG00000077348](https://www.ensembl.org/id/ENSG00000077348) | exosome component 5 [Source:HGNC Symbol;Acc:HGNC:24662] | 1,14 |
| EXOSC7 | [ENSG00000075914](https://www.ensembl.org/id/ENSG00000075914) | exosome component 7 [Source:HGNC Symbol;Acc:HGNC:28112] | 1,78 |
| EXTL1 | [ENSG00000158008](https://www.ensembl.org/id/ENSG00000158008) | exostosin like glycosyltransferase 1 [Source:HGNC Symbol;Acc:HGNC:3515] | 2,89 |
| F2RL2 | [ENSG00000164220](https://www.ensembl.org/id/ENSG00000164220) | coagulation factor II thrombin receptor like 2 [Source:HGNC Symbol;Acc:HGNC:3539] | 2,53 |
| F2RL3 | [ENSG00000127533](https://www.ensembl.org/id/ENSG00000127533) | F2R like thrombin or trypsin receptor 3 [Source:HGNC Symbol;Acc:HGNC:3540] | -2,50 |
| FABP3 | [ENSG00000121769](https://www.ensembl.org/id/ENSG00000121769) | fatty acid binding protein 3 [Source:HGNC Symbol;Acc:HGNC:3557] | 2,07 |
| FABP4 | [ENSG00000170323](https://www.ensembl.org/id/ENSG00000170323) | fatty acid binding protein 4 [Source:HGNC Symbol;Acc:HGNC:3559] | -2,01 |
| FABP7 | [ENSG00000164434](https://www.ensembl.org/id/ENSG00000164434) | fatty acid binding protein 7 [Source:HGNC Symbol;Acc:HGNC:3562] | 3,29 |
| FALEC | [ENSG00000228126](https://www.ensembl.org/id/ENSG00000228126) | focally amplified long non-coding RNA in epithelial cancer [Source:HGNC Symbol;Acc:HGNC:43713] | 4,82 |
| FAM107B | [ENSG00000065809](https://www.ensembl.org/id/ENSG00000065809) | family with sequence similarity 107 member B [Source:HGNC Symbol;Acc:HGNC:23726] | -1,21 |
| FAM110C | [ENSG00000184731](https://www.ensembl.org/id/ENSG00000184731) | family with sequence similarity 110 member C [Source:HGNC Symbol;Acc:HGNC:33340] | -1,67 |
| FAM135B | [ENSG00000147724](https://www.ensembl.org/id/ENSG00000147724) | family with sequence similarity 135 member B [Source:HGNC Symbol;Acc:HGNC:28029] | 3,81 |
| FAM153B | [ENSG00000289731](https://www.ensembl.org/id/ENSG00000289731) | family with sequence similarity 153 member B [Source:HGNC Symbol;Acc:HGNC:27323] | -3,08 |
| FAM153CP | [ENSG00000204677](https://www.ensembl.org/id/ENSG00000204677) | family with sequence similarity 153 member C, pseudogene [Source:HGNC Symbol;Acc:HGNC:33936] | -2,78 |
| FAM167A | [ENSG00000154319](https://www.ensembl.org/id/ENSG00000154319) | family with sequence similarity 167 member A [Source:HGNC Symbol;Acc:HGNC:15549] | -3,50 |
| FAM180A | [ENSG00000189320](https://www.ensembl.org/id/ENSG00000189320) | family with sequence similarity 180 member A [Source:HGNC Symbol;Acc:HGNC:33773] | -1,44 |
| FAM182B | [ENSG00000175170](https://www.ensembl.org/id/ENSG00000175170) | family with sequence similarity 182 member B [Source:HGNC Symbol;Acc:HGNC:34503] | 2,44 |
| FAM209A | [ENSG00000124103](https://www.ensembl.org/id/ENSG00000124103) | family with sequence similarity 209 member A [Source:HGNC Symbol;Acc:HGNC:16100] | 3,15 |
| FAM220A | [ENSG00000178397](https://www.ensembl.org/id/ENSG00000178397) | family with sequence similarity 220 member A [Source:HGNC Symbol;Acc:HGNC:22422] | 1,44 |
| FAM222A-AS1 | [ENSG00000255650](https://www.ensembl.org/id/ENSG00000255650) | FAM222A antisense RNA 1 [Source:HGNC Symbol;Acc:HGNC:28223] | 3,69 |
| FAM240C | [ENSG00000216921](https://www.ensembl.org/id/ENSG00000216921) | family with sequence similarity 240 member C [Source:HGNC Symbol;Acc:HGNC:54200] | 5,36 |
| FAM47E-STBD1 | [ENSG00000272414](https://www.ensembl.org/id/ENSG00000272414) | FAM47E-STBD1 readthrough [Source:HGNC Symbol;Acc:HGNC:44667] | 2,53 |
| FAM50B | [ENSG00000145945](https://www.ensembl.org/id/ENSG00000145945) | family with sequence similarity 50 member B [Source:HGNC Symbol;Acc:HGNC:18789] | 1,00 |
| FAM78A | [ENSG00000126882](https://www.ensembl.org/id/ENSG00000126882) | family with sequence similarity 78 member A [Source:HGNC Symbol;Acc:HGNC:25465] | 2,13 |
| FAM83B | [ENSG00000168143](https://www.ensembl.org/id/ENSG00000168143) | family with sequence similarity 83 member B [Source:HGNC Symbol;Acc:HGNC:21357] | 3,13 |
| FAM83G | [ENSG00000188522](https://www.ensembl.org/id/ENSG00000188522) | family with sequence similarity 83 member G [Source:HGNC Symbol;Acc:HGNC:32554] | -1,55 |
| FAM83H | [ENSG00000180921](https://www.ensembl.org/id/ENSG00000180921) | family with sequence similarity 83 member H [Source:HGNC Symbol;Acc:HGNC:24797] | -2,42 |
| FASN | [ENSG00000169710](https://www.ensembl.org/id/ENSG00000169710) | fatty acid synthase [Source:HGNC Symbol;Acc:HGNC:3594] | -1,82 |
| FBXO3 | [ENSG00000110429](https://www.ensembl.org/id/ENSG00000110429) | F-box protein 3 [Source:HGNC Symbol;Acc:HGNC:13582] | 0,98 |
| FBXO40 | [ENSG00000163833](https://www.ensembl.org/id/ENSG00000163833) | F-box protein 40 [Source:HGNC Symbol;Acc:HGNC:29816] | 3,27 |
| FCAR | [ENSG00000186431](https://www.ensembl.org/id/ENSG00000186431) | Fc alpha receptor [Source:HGNC Symbol;Acc:HGNC:3608] | 4,95 |
| FCER1A | [ENSG00000179639](https://www.ensembl.org/id/ENSG00000179639) | Fc epsilon receptor Ia [Source:HGNC Symbol;Acc:HGNC:3609] | 2,43 |
| FCGBP | [ENSG00000275395](https://www.ensembl.org/id/ENSG00000275395) | Fc gamma binding protein [Source:HGNC Symbol;Acc:HGNC:13572] | 4,15 |
| FCGR1A | [ENSG00000150337](https://www.ensembl.org/id/ENSG00000150337) | Fc gamma receptor Ia [Source:HGNC Symbol;Acc:HGNC:3613] | 2,35 |
| FCGR3A | [ENSG00000203747](https://www.ensembl.org/id/ENSG00000203747) | Fc gamma receptor IIIa [Source:HGNC Symbol;Acc:HGNC:3619] | 2,99 |
| FCGR3B | [ENSG00000162747](https://www.ensembl.org/id/ENSG00000162747) | Fc gamma receptor IIIb [Source:HGNC Symbol;Acc:HGNC:3620] | 4,83 |
| FCMR | [ENSG00000162894](https://www.ensembl.org/id/ENSG00000162894) | Fc mu receptor [Source:HGNC Symbol;Acc:HGNC:14315] | 2,48 |
| FCN1 | [ENSG00000085265](https://www.ensembl.org/id/ENSG00000085265) | ficolin 1 [Source:HGNC Symbol;Acc:HGNC:3623] | 3,67 |
| FCN2 | [ENSG00000160339](https://www.ensembl.org/id/ENSG00000160339) | ficolin 2 [Source:HGNC Symbol;Acc:HGNC:3624] | -3,51 |
| FCRL1 | [ENSG00000163534](https://www.ensembl.org/id/ENSG00000163534) | Fc receptor like 1 [Source:HGNC Symbol;Acc:HGNC:18509] | 5,36 |
| FCRL3 | [ENSG00000160856](https://www.ensembl.org/id/ENSG00000160856) | Fc receptor like 3 [Source:HGNC Symbol;Acc:HGNC:18506] | 3,84 |
| FDX1 | [ENSG00000137714](https://www.ensembl.org/id/ENSG00000137714) | ferredoxin 1 [Source:HGNC Symbol;Acc:HGNC:3638] | 1,01 |
| FECH | [ENSG00000066926](https://www.ensembl.org/id/ENSG00000066926) | ferrochelatase [Source:HGNC Symbol;Acc:HGNC:3647] | 1,39 |
| FEM1A | [ENSG00000141965](https://www.ensembl.org/id/ENSG00000141965) | fem-1 homolog A [Source:HGNC Symbol;Acc:HGNC:16934] | 2,30 |
| FER1L5 | [ENSG00000249715](https://www.ensembl.org/id/ENSG00000249715) | fer-1 like family member 5 [Source:HGNC Symbol;Acc:HGNC:19044] | 3,90 |
| FEZ2 | [ENSG00000171055](https://www.ensembl.org/id/ENSG00000171055) | fasciculation and elongation protein zeta 2 [Source:HGNC Symbol;Acc:HGNC:3660] | 1,80 |
| FEZF1-AS1 | [ENSG00000230316](https://www.ensembl.org/id/ENSG00000230316) | FEZF1 antisense RNA 1 [Source:HGNC Symbol;Acc:HGNC:41001] | 4,78 |
| FFAR4 | [ENSG00000186188](https://www.ensembl.org/id/ENSG00000186188) | free fatty acid receptor 4 [Source:HGNC Symbol;Acc:HGNC:19061] | -3,51 |
| FGF10 | [ENSG00000070193](https://www.ensembl.org/id/ENSG00000070193) | fibroblast growth factor 10 [Source:HGNC Symbol;Acc:HGNC:3666] | -1,80 |
| FGF8 | [ENSG00000107831](https://www.ensembl.org/id/ENSG00000107831) | fibroblast growth factor 8 [Source:HGNC Symbol;Acc:HGNC:3686] | -5,95 |
| FGFBP1 | [ENSG00000137440](https://www.ensembl.org/id/ENSG00000137440) | fibroblast growth factor binding protein 1 [Source:HGNC Symbol;Acc:HGNC:19695] | -4,86 |
| FGFR2 | [ENSG00000066468](https://www.ensembl.org/id/ENSG00000066468) | fibroblast growth factor receptor 2 [Source:HGNC Symbol;Acc:HGNC:3689] | -1,68 |
| FH | [ENSG00000091483](https://www.ensembl.org/id/ENSG00000091483) | fumarate hydratase [Source:HGNC Symbol;Acc:HGNC:3700] | 1,48 |
| FHAD1-AS1 | [ENSG00000233485](https://www.ensembl.org/id/ENSG00000233485) | FHAD1 antisense RNA 1 [Source:HGNC Symbol;Acc:HGNC:41241] | -3,62 |
| FHIP1A | [ENSG00000164142](https://www.ensembl.org/id/ENSG00000164142) | FHF complex subunit HOOK interacting protein 1A [Source:HGNC Symbol;Acc:HGNC:34237] | 2,17 |
| FHL2 | [ENSG00000115641](https://www.ensembl.org/id/ENSG00000115641) | four and a half LIM domains 2 [Source:HGNC Symbol;Acc:HGNC:3703] | 1,01 |
| FHL3 | [ENSG00000183386](https://www.ensembl.org/id/ENSG00000183386) | four and a half LIM domains 3 [Source:HGNC Symbol;Acc:HGNC:3704] | 2,22 |
| FHOD1 | [ENSG00000135723](https://www.ensembl.org/id/ENSG00000135723) | formin homology 2 domain containing 1 [Source:HGNC Symbol;Acc:HGNC:17905] | 1,37 |
| FHOD3 | [ENSG00000134775](https://www.ensembl.org/id/ENSG00000134775) | formin homology 2 domain containing 3 [Source:HGNC Symbol;Acc:HGNC:26178] | 1,46 |
| FITM1 | [ENSG00000139914](https://www.ensembl.org/id/ENSG00000139914) | fat storage inducing transmembrane protein 1 [Source:HGNC Symbol;Acc:HGNC:33714] | 2,81 |
| FKBP14-AS1 | [ENSG00000227014](https://www.ensembl.org/id/ENSG00000227014) | FKBP14 antisense RNA 1 [Source:HGNC Symbol;Acc:HGNC:40990] | -2,55 |
| FKBP3 | [ENSG00000100442](https://www.ensembl.org/id/ENSG00000100442) | FKBP prolyl isomerase 3 [Source:HGNC Symbol;Acc:HGNC:3719] | 1,87 |
| FMC1 | [ENSG00000164898](https://www.ensembl.org/id/ENSG00000164898) | formation of mitochondrial complex V assembly factor 1 homolog [Source:HGNC Symbol;Acc:HGNC:26946] | 1,18 |
| FNDC4 | [ENSG00000115226](https://www.ensembl.org/id/ENSG00000115226) | fibronectin type III domain containing 4 [Source:HGNC Symbol;Acc:HGNC:20239] | -1,25 |
| FOLR3 | [ENSG00000110203](https://www.ensembl.org/id/ENSG00000110203) | folate receptor gamma [Source:HGNC Symbol;Acc:HGNC:3795] | 5,40 |
| FOSL1 | [ENSG00000175592](https://www.ensembl.org/id/ENSG00000175592) | FOS like 1, AP-1 transcription factor subunit [Source:HGNC Symbol;Acc:HGNC:13718] | -2,92 |
| FOXO4 | [ENSG00000184481](https://www.ensembl.org/id/ENSG00000184481) | forkhead box O4 [Source:HGNC Symbol;Acc:HGNC:7139] | 1,15 |
| FOXS1 | [ENSG00000179772](https://www.ensembl.org/id/ENSG00000179772) | forkhead box S1 [Source:HGNC Symbol;Acc:HGNC:3735] | -1,23 |
| FPR1 | [ENSG00000171051](https://www.ensembl.org/id/ENSG00000171051) | formyl peptide receptor 1 [Source:HGNC Symbol;Acc:HGNC:3826] | 3,60 |
| FPR2 | [ENSG00000171049](https://www.ensembl.org/id/ENSG00000171049) | formyl peptide receptor 2 [Source:HGNC Symbol;Acc:HGNC:3827] | 4,76 |
| FRAT1 | [ENSG00000165879](https://www.ensembl.org/id/ENSG00000165879) | FRAT regulator of WNT signaling pathway 1 [Source:HGNC Symbol;Acc:HGNC:3944] | 1,76 |
| FRMD1 | [ENSG00000153303](https://www.ensembl.org/id/ENSG00000153303) | FERM domain containing 1 [Source:HGNC Symbol;Acc:HGNC:21240] | 1,71 |
| FSD1L | [ENSG00000106701](https://www.ensembl.org/id/ENSG00000106701) | fibronectin type III and SPRY domain containing 1 like [Source:HGNC Symbol;Acc:HGNC:13753] | 1,74 |
| FSTL3 | [ENSG00000070404](https://www.ensembl.org/id/ENSG00000070404) | follistatin like 3 [Source:HGNC Symbol;Acc:HGNC:3973] | -1,08 |
| FTCDNL1 | [ENSG00000226124](https://www.ensembl.org/id/ENSG00000226124) | formiminotransferase cyclodeaminase N-terminal like [Source:HGNC Symbol;Acc:HGNC:48661] | 2,63 |
| FUNDC2 | [ENSG00000165775](https://www.ensembl.org/id/ENSG00000165775) | FUN14 domain containing 2 [Source:HGNC Symbol;Acc:HGNC:24925] | 1,24 |
| FXR1 | [ENSG00000114416](https://www.ensembl.org/id/ENSG00000114416) | FMR1 autosomal homolog 1 [Source:HGNC Symbol;Acc:HGNC:4023] | 1,39 |
| FXYD4 | [ENSG00000150201](https://www.ensembl.org/id/ENSG00000150201) | FXYD domain containing ion transport regulator 4 [Source:HGNC Symbol;Acc:HGNC:4028] | 2,98 |
| FYB1 | [ENSG00000082074](https://www.ensembl.org/id/ENSG00000082074) | FYN binding protein 1 [Source:HGNC Symbol;Acc:HGNC:4036] | 2,46 |
| FYCO1 | [ENSG00000163820](https://www.ensembl.org/id/ENSG00000163820) | FYVE and coiled-coil domain autophagy adaptor 1 [Source:HGNC Symbol;Acc:HGNC:14673] | 1,71 |
| FZD2 | [ENSG00000180340](https://www.ensembl.org/id/ENSG00000180340) | frizzled class receptor 2 [Source:HGNC Symbol;Acc:HGNC:4040] | -1,35 |
| FZD9 | [ENSG00000188763](https://www.ensembl.org/id/ENSG00000188763) | frizzled class receptor 9 [Source:HGNC Symbol;Acc:HGNC:4047] | 2,10 |
| GABRA4 | [ENSG00000109158](https://www.ensembl.org/id/ENSG00000109158) | gamma-aminobutyric acid type A receptor subunit alpha4 [Source:HGNC Symbol;Acc:HGNC:4078] | -5,37 |
| GABRB2 | [ENSG00000145864](https://www.ensembl.org/id/ENSG00000145864) | gamma-aminobutyric acid type A receptor subunit beta2 [Source:HGNC Symbol;Acc:HGNC:4082] | 3,07 |
| GABRE | [ENSG00000102287](https://www.ensembl.org/id/ENSG00000102287) | gamma-aminobutyric acid type A receptor subunit epsilon [Source:HGNC Symbol;Acc:HGNC:4085] | -1,59 |
| GADL1 | [ENSG00000144644](https://www.ensembl.org/id/ENSG00000144644) | glutamate decarboxylase like 1 [Source:HGNC Symbol;Acc:HGNC:27949] | 2,08 |
| GALNT13 | [ENSG00000144278](https://www.ensembl.org/id/ENSG00000144278) | polypeptide N-acetylgalactosaminyltransferase 13 [Source:HGNC Symbol;Acc:HGNC:23242] | 1,89 |
| GALNT18 | [ENSG00000110328](https://www.ensembl.org/id/ENSG00000110328) | polypeptide N-acetylgalactosaminyltransferase 18 [Source:HGNC Symbol;Acc:HGNC:30488] | -1,33 |
| GALNT9 | [ENSG00000182870](https://www.ensembl.org/id/ENSG00000182870) | polypeptide N-acetylgalactosaminyltransferase 9 [Source:HGNC Symbol;Acc:HGNC:4131] | -6,28 |
| GAPT | [ENSG00000175857](https://www.ensembl.org/id/ENSG00000175857) | GRB2 binding adaptor protein, transmembrane [Source:HGNC Symbol;Acc:HGNC:26588] | 6,48 |
| GARIN5A | [ENSG00000142530](https://www.ensembl.org/id/ENSG00000142530) | golgi associated RAB2 interactor 5A [Source:HGNC Symbol;Acc:HGNC:25107] | 2,25 |
| GATA4 | [ENSG00000136574](https://www.ensembl.org/id/ENSG00000136574) | GATA binding protein 4 [Source:HGNC Symbol;Acc:HGNC:4173] | -4,82 |
| GATA5 | [ENSG00000130700](https://www.ensembl.org/id/ENSG00000130700) | GATA binding protein 5 [Source:HGNC Symbol;Acc:HGNC:15802] | -2,73 |
| GATB | [ENSG00000059691](https://www.ensembl.org/id/ENSG00000059691) | glutamyl-tRNA amidotransferase subunit B [Source:HGNC Symbol;Acc:HGNC:8849] | 1,62 |
| GATD3 | [ENSG00000160221](https://www.ensembl.org/id/ENSG00000160221) | glutamine amidotransferase class 1 domain containing 3 [Source:HGNC Symbol;Acc:HGNC:1273] | 3,39 |
| GATM | [ENSG00000171766](https://www.ensembl.org/id/ENSG00000171766) | glycine amidinotransferase [Source:HGNC Symbol;Acc:HGNC:4175] | 1,34 |
| GBP3 | [ENSG00000117226](https://www.ensembl.org/id/ENSG00000117226) | guanylate binding protein 3 [Source:HGNC Symbol;Acc:HGNC:4184] | 1,26 |
| GCAT | [ENSG00000100116](https://www.ensembl.org/id/ENSG00000100116) | glycine C-acetyltransferase [Source:HGNC Symbol;Acc:HGNC:4188] | 1,41 |
| GCDH | [ENSG00000105607](https://www.ensembl.org/id/ENSG00000105607) | glutaryl-CoA dehydrogenase [Source:HGNC Symbol;Acc:HGNC:4189] | 0,97 |
| GDF3 | [ENSG00000184344](https://www.ensembl.org/id/ENSG00000184344) | growth differentiation factor 3 [Source:HGNC Symbol;Acc:HGNC:4218] | -4,97 |
| gene:ENSG00000093100 | [ENSG00000093100](https://www.ensembl.org/id/ENSG00000093100) | novel transcript | -0,98 |
| gene:ENSG00000167774 | [ENSG00000167774](https://www.ensembl.org/id/ENSG00000167774) | novel transcript | 2,29 |
| gene:ENSG00000196826 | [ENSG00000196826](https://www.ensembl.org/id/ENSG00000196826) | novel zinc finger protein | -6,50 |
| gene:ENSG00000197813 | [ENSG00000197813](https://www.ensembl.org/id/ENSG00000197813) | novel transcript | -2,77 |
| gene:ENSG00000214970 | [ENSG00000214970](https://www.ensembl.org/id/ENSG00000214970) | novel transcript, antisense to MYH2, MYH8, MYH1 and MYH2 | 2,93 |
| gene:ENSG00000221857 | [ENSG00000221857](https://www.ensembl.org/id/ENSG00000221857) | novel transcript | 3,91 |
| gene:ENSG00000223774 | [ENSG00000223774](https://www.ensembl.org/id/ENSG00000223774) | novel transcript | 2,70 |
| gene:ENSG00000223930 | [ENSG00000223930](https://www.ensembl.org/id/ENSG00000223930) | novel transcript, antisense to KCNMB2 | 3,25 |
| gene:ENSG00000224635 | [ENSG00000224635](https://www.ensembl.org/id/ENSG00000224635) | novel transcript | -3,34 |
| gene:ENSG00000224945 | [ENSG00000224945](https://www.ensembl.org/id/ENSG00000224945) | novel transcript | -3,76 |
| gene:ENSG00000225325 | [ENSG00000225325](https://www.ensembl.org/id/ENSG00000225325) | novel transcript | 4,88 |
| gene:ENSG00000225528 | [ENSG00000225528](https://www.ensembl.org/id/ENSG00000225528) | novel protein similar to translation machinery associated 7 homolog (S. cerevisiae) TMA7 | 4,12 |
| gene:ENSG00000225929 | [ENSG00000225929](https://www.ensembl.org/id/ENSG00000225929) | novel transcript | -2,90 |
| gene:ENSG00000226087 | [ENSG00000226087](https://www.ensembl.org/id/ENSG00000226087) | novel transcript | 2,92 |
| gene:ENSG00000226143 | [ENSG00000226143](https://www.ensembl.org/id/ENSG00000226143) | novel transcript | -6,07 |
| gene:ENSG00000226332 | [ENSG00000226332](https://www.ensembl.org/id/ENSG00000226332) | novel transcript | -1,82 |
| gene:ENSG00000226578 | [ENSG00000226578](https://www.ensembl.org/id/ENSG00000226578) | novel transcript | 6,00 |
| gene:ENSG00000227017 | [ENSG00000227017](https://www.ensembl.org/id/ENSG00000227017) | novel transcript | 6,04 |
| gene:ENSG00000227706 | [ENSG00000227706](https://www.ensembl.org/id/ENSG00000227706) | novel transcript | 6,31 |
| gene:ENSG00000228162 | [ENSG00000228162](https://www.ensembl.org/id/ENSG00000228162) | novel transcript | 4,99 |
| gene:ENSG00000228318 | [ENSG00000228318](https://www.ensembl.org/id/ENSG00000228318) | novel transcript | 3,51 |
| gene:ENSG00000228392 | [ENSG00000228392](https://www.ensembl.org/id/ENSG00000228392) | novel transcript | 5,83 |
| gene:ENSG00000228714 | [ENSG00000228714](https://www.ensembl.org/id/ENSG00000228714) | novel transcript, antisense to DEC1 | 4,88 |
| gene:ENSG00000229167 | [ENSG00000229167](https://www.ensembl.org/id/ENSG00000229167) | novel transcript | -5,20 |
| gene:ENSG00000230385 | [ENSG00000230385](https://www.ensembl.org/id/ENSG00000230385) | novel transcript | 5,19 |
| gene:ENSG00000230387 | [ENSG00000230387](https://www.ensembl.org/id/ENSG00000230387) | novel transcript | 2,31 |
| gene:ENSG00000230423 | [ENSG00000230423](https://www.ensembl.org/id/ENSG00000230423) | novel transcript | -3,21 |
| gene:ENSG00000230955 | [ENSG00000230955](https://www.ensembl.org/id/ENSG00000230955) | novel transcript | -3,23 |
| gene:ENSG00000231412 | [ENSG00000231412](https://www.ensembl.org/id/ENSG00000231412) | novel transcript | -4,05 |
| gene:ENSG00000231482 | [ENSG00000231482](https://www.ensembl.org/id/ENSG00000231482) | novel transcript | -4,02 |
| gene:ENSG00000231536 | [ENSG00000231536](https://www.ensembl.org/id/ENSG00000231536) | novel transcript | 4,09 |
| gene:ENSG00000232058 | [ENSG00000232058](https://www.ensembl.org/id/ENSG00000232058) | novel transcript | 6,39 |
| gene:ENSG00000232719 | [ENSG00000232719](https://www.ensembl.org/id/ENSG00000232719) | novel transcript | 4,86 |
| gene:ENSG00000233005 | [ENSG00000233005](https://www.ensembl.org/id/ENSG00000233005) | novel transcript | -4,21 |
| gene:ENSG00000233397 | [ENSG00000233397](https://www.ensembl.org/id/ENSG00000233397) | novel transcript | 5,44 |
| gene:ENSG00000233547 | [ENSG00000233547](https://www.ensembl.org/id/ENSG00000233547) | novel transcript | -5,60 |
| gene:ENSG00000234132 | [ENSG00000234132](https://www.ensembl.org/id/ENSG00000234132) | novel transcript, antisense CACNA1S | 5,03 |
| gene:ENSG00000234139 | [ENSG00000234139](https://www.ensembl.org/id/ENSG00000234139) | novel transcript | 5,77 |
| gene:ENSG00000235020 | [ENSG00000235020](https://www.ensembl.org/id/ENSG00000235020) | novel transcript | 2,78 |
| gene:ENSG00000235027 | [ENSG00000235027](https://www.ensembl.org/id/ENSG00000235027) | PRC2 and DDX5 associated lncRNA [Source:HGNC Symbol;Acc:HGNC:40168] | 6,13 |
| gene:ENSG00000235151 | [ENSG00000235151](https://www.ensembl.org/id/ENSG00000235151) | novel transcript | 5,51 |
| gene:ENSG00000235288 | [ENSG00000235288](https://www.ensembl.org/id/ENSG00000235288) | novel transcript, antisense to CCBP2 | 3,85 |
| gene:ENSG00000235351 | [ENSG00000235351](https://www.ensembl.org/id/ENSG00000235351) | novel transcript | -3,19 |
| gene:ENSG00000235994 | [ENSG00000235994](https://www.ensembl.org/id/ENSG00000235994) | novel transcript | 5,23 |
| gene:ENSG00000236494 | [ENSG00000236494](https://www.ensembl.org/id/ENSG00000236494) | novel transcript | 3,26 |
| gene:ENSG00000236627 | [ENSG00000236627](https://www.ensembl.org/id/ENSG00000236627) | novel transcript, antisense to PDE10A | 6,61 |
| gene:ENSG00000236842 | [ENSG00000236842](https://www.ensembl.org/id/ENSG00000236842) | novel transcript, antisense to C10orf11 | 5,43 |
| gene:ENSG00000236883 | [ENSG00000236883](https://www.ensembl.org/id/ENSG00000236883) | novel transcript | 4,05 |
| gene:ENSG00000236936 | [ENSG00000236936](https://www.ensembl.org/id/ENSG00000236936) | novel transcript | 6,84 |
| gene:ENSG00000237419 | [ENSG00000237419](https://www.ensembl.org/id/ENSG00000237419) | novel transcript | 6,60 |
| gene:ENSG00000237720 | [ENSG00000237720](https://www.ensembl.org/id/ENSG00000237720) | novel transcript | 5,34 |
| gene:ENSG00000238007 | [ENSG00000238007](https://www.ensembl.org/id/ENSG00000238007) | novel transcript | -5,06 |
| gene:ENSG00000238279 | [ENSG00000238279](https://www.ensembl.org/id/ENSG00000238279) | novel transcript | -2,43 |
| gene:ENSG00000239381 | [ENSG00000239381](https://www.ensembl.org/id/ENSG00000239381) | novel transcript | 6,95 |
| gene:ENSG00000239775 | [ENSG00000239775](https://www.ensembl.org/id/ENSG00000239775) | novel transcript, sense overlapping DBNL | 2,43 |
| gene:ENSG00000243179 | [ENSG00000243179](https://www.ensembl.org/id/ENSG00000243179) | novel transcript | 4,88 |
| gene:ENSG00000243762 | [ENSG00000243762](https://www.ensembl.org/id/ENSG00000243762) | novel transcript, antisense to DGCR8 | 7,03 |
| gene:ENSG00000246477 | [ENSG00000246477](https://www.ensembl.org/id/ENSG00000246477) | novel transcript, antisense to MTMR9 | 2,50 |
| gene:ENSG00000247765 | [ENSG00000247765](https://www.ensembl.org/id/ENSG00000247765) | novel transcript | -3,43 |
| gene:ENSG00000248015 | [ENSG00000248015](https://www.ensembl.org/id/ENSG00000248015) | novel transcript, antisense to NDUFS7 | 2,91 |
| gene:ENSG00000248544 | [ENSG00000248544](https://www.ensembl.org/id/ENSG00000248544) | novel transcript, antisense to CYFIP2 | -4,04 |
| gene:ENSG00000248964 | [ENSG00000248964](https://www.ensembl.org/id/ENSG00000248964) | novel transcript | 3,54 |
| gene:ENSG00000249209 | [ENSG00000249209](https://www.ensembl.org/id/ENSG00000249209) | novel protein similar to ATP synthase delta (OSCP) subunit domain | 3,94 |
| gene:ENSG00000249388 | [ENSG00000249388](https://www.ensembl.org/id/ENSG00000249388) | novel transcript | 5,19 |
| gene:ENSG00000249593 | [ENSG00000249593](https://www.ensembl.org/id/ENSG00000249593) | novel transcript | 3,92 |
| gene:ENSG00000249971 | [ENSG00000249971](https://www.ensembl.org/id/ENSG00000249971) | novel transcript | -5,51 |
| gene:ENSG00000250264 | [ENSG00000250264](https://www.ensembl.org/id/ENSG00000250264) | novel protein, TAP2-HLA-DOB readthrough | 1,95 |
| gene:ENSG00000250286 | [ENSG00000250286](https://www.ensembl.org/id/ENSG00000250286) | novel transcript, antisense to EPN3 | -5,41 |
| gene:ENSG00000250348 | [ENSG00000250348](https://www.ensembl.org/id/ENSG00000250348) | novel transcript | 5,83 |
| gene:ENSG00000250424 | [ENSG00000250424](https://www.ensembl.org/id/ENSG00000250424) | novel protein, MINDY4 and AQP1 readthrough | -5,30 |
| gene:ENSG00000250511 | [ENSG00000250511](https://www.ensembl.org/id/ENSG00000250511) | novel transcript, antisense to ENPEP | 6,03 |
| gene:ENSG00000250697 | [ENSG00000250697](https://www.ensembl.org/id/ENSG00000250697) | novel transcript | 5,43 |
| gene:ENSG00000250863 | [ENSG00000250863](https://www.ensembl.org/id/ENSG00000250863) | novel transcript | 5,96 |
| gene:ENSG00000251081 | [ENSG00000251081](https://www.ensembl.org/id/ENSG00000251081) | novel transcript | 3,39 |
| gene:ENSG00000251257 | [ENSG00000251257](https://www.ensembl.org/id/ENSG00000251257) | novel transcript | 3,02 |
| gene:ENSG00000253348 | [ENSG00000253348](https://www.ensembl.org/id/ENSG00000253348) | novel transcript | -5,20 |
| gene:ENSG00000253476 | [ENSG00000253476](https://www.ensembl.org/id/ENSG00000253476) | novel transcript | -3,49 |
| gene:ENSG00000254054 | [ENSG00000254054](https://www.ensembl.org/id/ENSG00000254054) | novel transcript | 6,14 |
| gene:ENSG00000254165 | [ENSG00000254165](https://www.ensembl.org/id/ENSG00000254165) | novel transcript | -2,31 |
| gene:ENSG00000254501 | [ENSG00000254501](https://www.ensembl.org/id/ENSG00000254501) | novel transcript | -3,98 |
| gene:ENSG00000254692 | [ENSG00000254692](https://www.ensembl.org/id/ENSG00000254692) | novel protein | -9,05 |
| gene:ENSG00000254706 | [ENSG00000254706](https://www.ensembl.org/id/ENSG00000254706) | novel protein | 4,27 |
| gene:ENSG00000255130 | [ENSG00000255130](https://www.ensembl.org/id/ENSG00000255130) | novel transcript | 4,88 |
| gene:ENSG00000255176 | [ENSG00000255176](https://www.ensembl.org/id/ENSG00000255176) | novel transcript | -2,49 |
| gene:ENSG00000255202 | [ENSG00000255202](https://www.ensembl.org/id/ENSG00000255202) | novel transcript | -4,91 |
| gene:ENSG00000255246 | [ENSG00000255246](https://www.ensembl.org/id/ENSG00000255246) | novel transcript | 5,99 |
| gene:ENSG00000255292 | [ENSG00000255292](https://www.ensembl.org/id/ENSG00000255292) | novel transcript | 3,18 |
| gene:ENSG00000255372 | [ENSG00000255372](https://www.ensembl.org/id/ENSG00000255372) | novel transcript | 4,96 |
| gene:ENSG00000255432 | [ENSG00000255432](https://www.ensembl.org/id/ENSG00000255432) | novel protein | 4,88 |
| gene:ENSG00000255455 | [ENSG00000255455](https://www.ensembl.org/id/ENSG00000255455) | novel transcript | 5,19 |
| gene:ENSG00000256538 | [ENSG00000256538](https://www.ensembl.org/id/ENSG00000256538) | novel transcript | 5,34 |
| gene:ENSG00000256654 | [ENSG00000256654](https://www.ensembl.org/id/ENSG00000256654) | novel transcript, sense overlapping KCNA1 | -3,81 |
| gene:ENSG00000256861 | [ENSG00000256861](https://www.ensembl.org/id/ENSG00000256861) | novel protein | 4,17 |
| gene:ENSG00000256897 | [ENSG00000256897](https://www.ensembl.org/id/ENSG00000256897) | novel transcript | 3,14 |
| gene:ENSG00000257042 | [ENSG00000257042](https://www.ensembl.org/id/ENSG00000257042) | novel transcript, antisense to PTHLH | 3,19 |
| gene:ENSG00000257277 | [ENSG00000257277](https://www.ensembl.org/id/ENSG00000257277) | novel transcript, antisense to ARHGAP15 | -4,44 |
| gene:ENSG00000257279 | [ENSG00000257279](https://www.ensembl.org/id/ENSG00000257279) | novel transcript | -5,51 |
| gene:ENSG00000257298 | [ENSG00000257298](https://www.ensembl.org/id/ENSG00000257298) | novel transcript, sense intronic to LIMA1 | -3,22 |
| gene:ENSG00000257386 | [ENSG00000257386](https://www.ensembl.org/id/ENSG00000257386) | novel transcript, antisense to ATXN7L3B | 5,19 |
| gene:ENSG00000257390 | [ENSG00000257390](https://www.ensembl.org/id/ENSG00000257390) | novel protein | -6,55 |
| gene:ENSG00000257431 | [ENSG00000257431](https://www.ensembl.org/id/ENSG00000257431) | novel transcript | -5,19 |
| gene:ENSG00000257964 | [ENSG00000257964](https://www.ensembl.org/id/ENSG00000257964) | novel transcript, antisense to PRPF40B | 4,40 |
| gene:ENSG00000258084 | [ENSG00000258084](https://www.ensembl.org/id/ENSG00000258084) | novel transcript | 5,83 |
| gene:ENSG00000258504 | [ENSG00000258504](https://www.ensembl.org/id/ENSG00000258504) | novel transcript | -2,86 |
| gene:ENSG00000258529 | [ENSG00000258529](https://www.ensembl.org/id/ENSG00000258529) | novel protein | -5,80 |
| gene:ENSG00000258695 | [ENSG00000258695](https://www.ensembl.org/id/ENSG00000258695) | novel transcript, antisense to HEATR4 | 3,83 |
| gene:ENSG00000258875 | [ENSG00000258875](https://www.ensembl.org/id/ENSG00000258875) | novel transcript, antisense to GPR68 | -2,72 |
| gene:ENSG00000259006 | [ENSG00000259006](https://www.ensembl.org/id/ENSG00000259006) | novel transcript, antisense to MC1R | 5,03 |
| gene:ENSG00000259033 | [ENSG00000259033](https://www.ensembl.org/id/ENSG00000259033) | novel transcript | 3,29 |
| gene:ENSG00000259132 | [ENSG00000259132](https://www.ensembl.org/id/ENSG00000259132) | novel protein | -6,41 |
| gene:ENSG00000259198 | [ENSG00000259198](https://www.ensembl.org/id/ENSG00000259198) | novel transcript, antisense to PLCB2 | 5,19 |
| gene:ENSG00000259250 | [ENSG00000259250](https://www.ensembl.org/id/ENSG00000259250) | novel transcript, antisense to ADAM10 | -3,09 |
| gene:ENSG00000259357 | [ENSG00000259357](https://www.ensembl.org/id/ENSG00000259357) | novel transcript, antisense to CERS2 | -2,41 |
| gene:ENSG00000259553 | [ENSG00000259553](https://www.ensembl.org/id/ENSG00000259553) | novel transcript | 5,21 |
| gene:ENSG00000259560 | [ENSG00000259560](https://www.ensembl.org/id/ENSG00000259560) | novel transcript | 4,28 |
| gene:ENSG00000259616 | [ENSG00000259616](https://www.ensembl.org/id/ENSG00000259616) | novel transcript | 6,15 |
| gene:ENSG00000259645 | [ENSG00000259645](https://www.ensembl.org/id/ENSG00000259645) | novel transcript, sense intronic to PAQR5 | -5,23 |
| gene:ENSG00000259668 | [ENSG00000259668](https://www.ensembl.org/id/ENSG00000259668) | novel transcript, antisense to DMXL2 | -2,11 |
| gene:ENSG00000259840 | [ENSG00000259840](https://www.ensembl.org/id/ENSG00000259840) | novel transcript | -4,68 |
| gene:ENSG00000260100 | [ENSG00000260100](https://www.ensembl.org/id/ENSG00000260100) | novel transcript | 5,68 |
| gene:ENSG00000260121 | [ENSG00000260121](https://www.ensembl.org/id/ENSG00000260121) | novel transcript, antisense to FAM38A | -2,15 |
| gene:ENSG00000260137 | [ENSG00000260137](https://www.ensembl.org/id/ENSG00000260137) | novel transcript | 5,42 |
| gene:ENSG00000260188 | [ENSG00000260188](https://www.ensembl.org/id/ENSG00000260188) | novel transcript, antisense to SLC22A16 | -5,06 |
| gene:ENSG00000260269 | [ENSG00000260269](https://www.ensembl.org/id/ENSG00000260269) | novel transcript | 3,86 |
| gene:ENSG00000260401 | [ENSG00000260401](https://www.ensembl.org/id/ENSG00000260401) | novel transcript, overlapping to P2RY2 | 1,86 |
| gene:ENSG00000260534 | [ENSG00000260534](https://www.ensembl.org/id/ENSG00000260534) | novel transcript | -3,11 |
| gene:ENSG00000260542 | [ENSG00000260542](https://www.ensembl.org/id/ENSG00000260542) | novel transcript | 2,98 |
| gene:ENSG00000260578 | [ENSG00000260578](https://www.ensembl.org/id/ENSG00000260578) | novel transcript | -2,32 |
| gene:ENSG00000260599 | [ENSG00000260599](https://www.ensembl.org/id/ENSG00000260599) | novel transcript | 3,83 |
| gene:ENSG00000260750 | [ENSG00000260750](https://www.ensembl.org/id/ENSG00000260750) | novel transcript | 4,44 |
| gene:ENSG00000260816 | [ENSG00000260816](https://www.ensembl.org/id/ENSG00000260816) | novel transcript, sense intronic to WWOX | -2,88 |
| gene:ENSG00000260838 | [ENSG00000260838](https://www.ensembl.org/id/ENSG00000260838) | novel transcript | -3,88 |
| gene:ENSG00000260914 | [ENSG00000260914](https://www.ensembl.org/id/ENSG00000260914) | novel protein | 1,42 |
| gene:ENSG00000260996 | [ENSG00000260996](https://www.ensembl.org/id/ENSG00000260996) | novel transcript, overlapping NRARP | -3,28 |
| gene:ENSG00000261055 | [ENSG00000261055](https://www.ensembl.org/id/ENSG00000261055) | novel transcript | 6,43 |
| gene:ENSG00000261159 | [ENSG00000261159](https://www.ensembl.org/id/ENSG00000261159) | novel transcript | -2,46 |
| gene:ENSG00000261168 | [ENSG00000261168](https://www.ensembl.org/id/ENSG00000261168) | novel transcript, sense overlapping SEMA6C | 2,80 |
| gene:ENSG00000261298 | [ENSG00000261298](https://www.ensembl.org/id/ENSG00000261298) | novel transcript | 6,60 |
| gene:ENSG00000261433 | [ENSG00000261433](https://www.ensembl.org/id/ENSG00000261433) | novel transcript | -5,57 |
| gene:ENSG00000261434 | [ENSG00000261434](https://www.ensembl.org/id/ENSG00000261434) | novel transcript, overlapping LRRC14B | 4,86 |
| gene:ENSG00000261476 | [ENSG00000261476](https://www.ensembl.org/id/ENSG00000261476) | novel transcript | 6,00 |
| gene:ENSG00000261553 | [ENSG00000261553](https://www.ensembl.org/id/ENSG00000261553) | novel transcript | 2,06 |
| gene:ENSG00000261578 | [ENSG00000261578](https://www.ensembl.org/id/ENSG00000261578) | novel transcript, overlapping to TSKU | -1,79 |
| gene:ENSG00000261625 | [ENSG00000261625](https://www.ensembl.org/id/ENSG00000261625) | novel transcript, overlapping to MRGPRF | -2,10 |
| gene:ENSG00000261641 | [ENSG00000261641](https://www.ensembl.org/id/ENSG00000261641) | novel transcript, antisense to CLCN7 | -2,15 |
| gene:ENSG00000261783 | [ENSG00000261783](https://www.ensembl.org/id/ENSG00000261783) | novel transcript, sense intronic to CFDP1 | 4,88 |
| gene:ENSG00000261795 | [ENSG00000261795](https://www.ensembl.org/id/ENSG00000261795) | novel transcript | 6,01 |
| gene:ENSG00000261822 | [ENSG00000261822](https://www.ensembl.org/id/ENSG00000261822) | novel transcript, antisense to HAUS2 | -6,03 |
| gene:ENSG00000261832 | [ENSG00000261832](https://www.ensembl.org/id/ENSG00000261832) | novel protein | 2,78 |
| gene:ENSG00000262633 | [ENSG00000262633](https://www.ensembl.org/id/ENSG00000262633) | novel protein | 5,33 |
| gene:ENSG00000262979 | [ENSG00000262979](https://www.ensembl.org/id/ENSG00000262979) | novel transcript | 3,58 |
| gene:ENSG00000263020 | [ENSG00000263020](https://www.ensembl.org/id/ENSG00000263020) | novel protein | -1,85 |
| gene:ENSG00000263154 | [ENSG00000263154](https://www.ensembl.org/id/ENSG00000263154) | novel transcript | 4,88 |
| gene:ENSG00000263489 | [ENSG00000263489](https://www.ensembl.org/id/ENSG00000263489) | novel transcript | 5,17 |
| gene:ENSG00000263585 | [ENSG00000263585](https://www.ensembl.org/id/ENSG00000263585) | novel transcript, antisense PYCR1 | 6,13 |
| gene:ENSG00000263603 | [ENSG00000263603](https://www.ensembl.org/id/ENSG00000263603) | novel transcript | 5,24 |
| gene:ENSG00000264116 | [ENSG00000264116](https://www.ensembl.org/id/ENSG00000264116) | novel transcript, antisense to SMIM21 | 3,52 |
| gene:ENSG00000264578 | [ENSG00000264578](https://www.ensembl.org/id/ENSG00000264578) | novel transcript, antisense to AGPAT6 | 4,86 |
| gene:ENSG00000264668 | [ENSG00000264668](https://www.ensembl.org/id/ENSG00000264668) | novel protein | 7,24 |
| gene:ENSG00000265618 | [ENSG00000265618](https://www.ensembl.org/id/ENSG00000265618) | novel transcript, antisense to TMEM199 | 8,43 |
| gene:ENSG00000266369 | [ENSG00000266369](https://www.ensembl.org/id/ENSG00000266369) | novel transcript, antisense to CCDC144NL | 4,16 |
| gene:ENSG00000266469 | [ENSG00000266469](https://www.ensembl.org/id/ENSG00000266469) | novel transcript, antisense to MED1 & FBXL20 | 2,73 |
| gene:ENSG00000266997 | [ENSG00000266997](https://www.ensembl.org/id/ENSG00000266997) | novel protein | 7,67 |
| gene:ENSG00000267016 | [ENSG00000267016](https://www.ensembl.org/id/ENSG00000267016) | novel transcript, antisense to SEPT9 | -5,20 |
| gene:ENSG00000267022 | [ENSG00000267022](https://www.ensembl.org/id/ENSG00000267022) | novel protein | -5,75 |
| gene:ENSG00000267069 | [ENSG00000267069](https://www.ensembl.org/id/ENSG00000267069) | novel transcript | -2,06 |
| gene:ENSG00000267096 | [ENSG00000267096](https://www.ensembl.org/id/ENSG00000267096) | novel transcript | -3,20 |
| gene:ENSG00000267122 | [ENSG00000267122](https://www.ensembl.org/id/ENSG00000267122) | novel transcript, antisense to DOT1L | -2,55 |
| gene:ENSG00000267160 | [ENSG00000267160](https://www.ensembl.org/id/ENSG00000267160) | novel transcript, antisense to C17orf104 | 2,16 |
| gene:ENSG00000267179 | [ENSG00000267179](https://www.ensembl.org/id/ENSG00000267179) | novel protein | 3,55 |
| gene:ENSG00000267197 | [ENSG00000267197](https://www.ensembl.org/id/ENSG00000267197) | novel transcript, sense intronic with KEAP1 | 5,43 |
| gene:ENSG00000267287 | [ENSG00000267287](https://www.ensembl.org/id/ENSG00000267287) | novel transcript | -2,38 |
| gene:ENSG00000267360 | [ENSG00000267360](https://www.ensembl.org/id/ENSG00000267360) | novel protein | 7,35 |
| gene:ENSG00000267417 | [ENSG00000267417](https://www.ensembl.org/id/ENSG00000267417) | novel transcript, antisense to NFIX | 3,39 |
| gene:ENSG00000267436 | [ENSG00000267436](https://www.ensembl.org/id/ENSG00000267436) | novel transcript, antisense to MFSD12 | -3,91 |
| gene:ENSG00000267439 | [ENSG00000267439](https://www.ensembl.org/id/ENSG00000267439) | novel transcript | -2,18 |
| gene:ENSG00000267466 | [ENSG00000267466](https://www.ensembl.org/id/ENSG00000267466) | novel transcript | -3,29 |
| gene:ENSG00000267560 | [ENSG00000267560](https://www.ensembl.org/id/ENSG00000267560) | novel transcript, antisense to KIAA1468 | 4,87 |
| gene:ENSG00000267561 | [ENSG00000267561](https://www.ensembl.org/id/ENSG00000267561) | novel protein | -6,16 |
| gene:ENSG00000267645 | [ENSG00000267645](https://www.ensembl.org/id/ENSG00000267645) | novel protein, POLR2J2-UPK3BL readthrough | 2,22 |
| gene:ENSG00000267707 | [ENSG00000267707](https://www.ensembl.org/id/ENSG00000267707) | novel transcript, antisense to CELF4 | -2,31 |
| gene:ENSG00000267769 | [ENSG00000267769](https://www.ensembl.org/id/ENSG00000267769) | novel transcript, antisense to UBX6 | -3,51 |
| gene:ENSG00000267882 | [ENSG00000267882](https://www.ensembl.org/id/ENSG00000267882) | novel transcript, antisense to ZMYND8 | -5,36 |
| gene:ENSG00000267892 | [ENSG00000267892](https://www.ensembl.org/id/ENSG00000267892) | novel transcript, antisense to CAPN12 | -2,62 |
| gene:ENSG00000268047 | [ENSG00000268047](https://www.ensembl.org/id/ENSG00000268047) | novel transcript, antisense to PTOV1 | 3,67 |
| gene:ENSG00000268083 | [ENSG00000268083](https://www.ensembl.org/id/ENSG00000268083) | novel protein | -5,81 |
| gene:ENSG00000268189 | [ENSG00000268189](https://www.ensembl.org/id/ENSG00000268189) | novel transcript, antisense to AKAP8 and AKAP8L | -2,32 |
| gene:ENSG00000268199 | [ENSG00000268199](https://www.ensembl.org/id/ENSG00000268199) | novel transcript, antisense to ELL | 5,43 |
| gene:ENSG00000268400 | [ENSG00000268400](https://www.ensembl.org/id/ENSG00000268400) | novel protein | 6,51 |
| gene:ENSG00000268401 | [ENSG00000268401](https://www.ensembl.org/id/ENSG00000268401) | novel transcript | -2,87 |
| gene:ENSG00000268536 | [ENSG00000268536](https://www.ensembl.org/id/ENSG00000268536) | novel transcript | 5,53 |
| gene:ENSG00000268603 | [ENSG00000268603](https://www.ensembl.org/id/ENSG00000268603) | novel transcript, antisense to SPEG | -1,66 |
| gene:ENSG00000268926 | [ENSG00000268926](https://www.ensembl.org/id/ENSG00000268926) | novel transcript | 2,40 |
| gene:ENSG00000269091 | [ENSG00000269091](https://www.ensembl.org/id/ENSG00000269091) | novel transcript, antisense to ZNF473 | -4,89 |
| gene:ENSG00000269107 | [ENSG00000269107](https://www.ensembl.org/id/ENSG00000269107) | novel transcript | 5,50 |
| gene:ENSG00000269271 | [ENSG00000269271](https://www.ensembl.org/id/ENSG00000269271) | novel transcript | 4,88 |
| gene:ENSG00000269289 | [ENSG00000269289](https://www.ensembl.org/id/ENSG00000269289) | novel transcript, antisense to ZNF726 | 5,19 |
| gene:ENSG00000269349 | [ENSG00000269349](https://www.ensembl.org/id/ENSG00000269349) | novel transcript, antisense to ZNF578 | 4,88 |
| gene:ENSG00000269427 | [ENSG00000269427](https://www.ensembl.org/id/ENSG00000269427) | novel transcript, antisense C19orf42 | -6,59 |
| gene:ENSG00000269560 | [ENSG00000269560](https://www.ensembl.org/id/ENSG00000269560) | novel transcript, sense intronic to ZNF564 | 6,27 |
| gene:ENSG00000269604 | [ENSG00000269604](https://www.ensembl.org/id/ENSG00000269604) | novel transcript, antisense to FEM1A | 2,19 |
| gene:ENSG00000269711 | [ENSG00000269711](https://www.ensembl.org/id/ENSG00000269711) | novel protein | 9,53 |
| gene:ENSG00000269980 | [ENSG00000269980](https://www.ensembl.org/id/ENSG00000269980) | novel transcript, antisense to CDK2AP1 | -4,72 |
| gene:ENSG00000270082 | [ENSG00000270082](https://www.ensembl.org/id/ENSG00000270082) | novel transcript, antisense to FBXO31 and C16orf95 readthrough | -3,40 |
| gene:ENSG00000271011 | [ENSG00000271011](https://www.ensembl.org/id/ENSG00000271011) | novel transcript, antisense to TTN | -5,46 |
| gene:ENSG00000271228 | [ENSG00000271228](https://www.ensembl.org/id/ENSG00000271228) | novel transcript, antisense to DPY30 | 3,68 |
| gene:ENSG00000271522 | [ENSG00000271522](https://www.ensembl.org/id/ENSG00000271522) | novel transcript | 5,34 |
| gene:ENSG00000271553 | [ENSG00000271553](https://www.ensembl.org/id/ENSG00000271553) | novel transcript | -2,14 |
| gene:ENSG00000271761 | [ENSG00000271761](https://www.ensembl.org/id/ENSG00000271761) | novel transcript | 4,89 |
| gene:ENSG00000271781 | [ENSG00000271781](https://www.ensembl.org/id/ENSG00000271781) | novel transcript, antisense to TPPP | -2,56 |
| gene:ENSG00000271787 | [ENSG00000271787](https://www.ensembl.org/id/ENSG00000271787) | novel transcript, antisense to KLF11 | -1,55 |
| gene:ENSG00000271871 | [ENSG00000271871](https://www.ensembl.org/id/ENSG00000271871) | novel transcript, antisense to PCDH12 | -2,53 |
| gene:ENSG00000271998 | [ENSG00000271998](https://www.ensembl.org/id/ENSG00000271998) | novel transcript | 3,41 |
| gene:ENSG00000272078 | [ENSG00000272078](https://www.ensembl.org/id/ENSG00000272078) | novel transcript, antisense to CASZ1 | -1,67 |
| gene:ENSG00000272234 | [ENSG00000272234](https://www.ensembl.org/id/ENSG00000272234) | novel transcript, antisense to SEPP1 | 4,80 |
| gene:ENSG00000272235 | [ENSG00000272235](https://www.ensembl.org/id/ENSG00000272235) | novel transcript | -2,22 |
| gene:ENSG00000272247 | [ENSG00000272247](https://www.ensembl.org/id/ENSG00000272247) | novel transcript, antisense to MFSD1 | 3,35 |
| gene:ENSG00000272384 | [ENSG00000272384](https://www.ensembl.org/id/ENSG00000272384) | novel transcript | 2,18 |
| gene:ENSG00000272416 | [ENSG00000272416](https://www.ensembl.org/id/ENSG00000272416) | novel transcript | -2,60 |
| gene:ENSG00000272468 | [ENSG00000272468](https://www.ensembl.org/id/ENSG00000272468) | novel transcript | -3,92 |
| gene:ENSG00000272509 | [ENSG00000272509](https://www.ensembl.org/id/ENSG00000272509) | novel transcript, antisense to CCNE2 | 5,81 |
| gene:ENSG00000272541 | [ENSG00000272541](https://www.ensembl.org/id/ENSG00000272541) | novel transcript | -2,41 |
| gene:ENSG00000272622 | [ENSG00000272622](https://www.ensembl.org/id/ENSG00000272622) | novel transcript | 2,36 |
| gene:ENSG00000272688 | [ENSG00000272688](https://www.ensembl.org/id/ENSG00000272688) | novel transcript | 2,05 |
| gene:ENSG00000272777 | [ENSG00000272777](https://www.ensembl.org/id/ENSG00000272777) | novel transcript | -2,29 |
| gene:ENSG00000272787 | [ENSG00000272787](https://www.ensembl.org/id/ENSG00000272787) | novel transcript | 3,62 |
| gene:ENSG00000272789 | [ENSG00000272789](https://www.ensembl.org/id/ENSG00000272789) | novel transcript, antisense to MYO7B | -2,51 |
| gene:ENSG00000272885 | [ENSG00000272885](https://www.ensembl.org/id/ENSG00000272885) | novel transcript | 3,70 |
| gene:ENSG00000273013 | [ENSG00000273013](https://www.ensembl.org/id/ENSG00000273013) | novel transcript, antisense to RNF168 | 3,85 |
| gene:ENSG00000273026 | [ENSG00000273026](https://www.ensembl.org/id/ENSG00000273026) | novel transcript, antisense to SLC39A1 | -3,14 |
| gene:ENSG00000273259 | [ENSG00000273259](https://www.ensembl.org/id/ENSG00000273259) | novel protein | 7,34 |
| gene:ENSG00000273387 | [ENSG00000273387](https://www.ensembl.org/id/ENSG00000273387) | novel transcript, antisense to SMTN | -2,58 |
| gene:ENSG00000273402 | [ENSG00000273402](https://www.ensembl.org/id/ENSG00000273402) | novel transcript, antisense to ZNF596 | -4,64 |
| gene:ENSG00000273590 | [ENSG00000273590](https://www.ensembl.org/id/ENSG00000273590) | small integral membrane protein 11B | 7,36 |
| gene:ENSG00000273669 | [ENSG00000273669](https://www.ensembl.org/id/ENSG00000273669) | novel transcript | 3,19 |
| gene:ENSG00000273796 | [ENSG00000273796](https://www.ensembl.org/id/ENSG00000273796) | novel transcript | -1,90 |
| gene:ENSG00000273998 | [ENSG00000273998](https://www.ensembl.org/id/ENSG00000273998) | novel transcript | -5,24 |
| gene:ENSG00000274276 | [ENSG00000274276](https://www.ensembl.org/id/ENSG00000274276) | cystathionine-beta-synthase like | 7,12 |
| gene:ENSG00000274414 | [ENSG00000274414](https://www.ensembl.org/id/ENSG00000274414) | novel transcript | -2,32 |
| gene:ENSG00000274987 | [ENSG00000274987](https://www.ensembl.org/id/ENSG00000274987) | novel transcript, antisense to KRAS | -2,78 |
| gene:ENSG00000275178 | [ENSG00000275178](https://www.ensembl.org/id/ENSG00000275178) | novel transcript, antisense to MBP | 5,47 |
| gene:ENSG00000275180 | [ENSG00000275180](https://www.ensembl.org/id/ENSG00000275180) | novel transcript | -2,10 |
| gene:ENSG00000275202 | [ENSG00000275202](https://www.ensembl.org/id/ENSG00000275202) | novel transcript, antisense to FNDC3A | -2,20 |
| gene:ENSG00000275413 | [ENSG00000275413](https://www.ensembl.org/id/ENSG00000275413) | novel transcript, antisense to TTC19 | 5,33 |
| gene:ENSG00000275485 | [ENSG00000275485](https://www.ensembl.org/id/ENSG00000275485) | novel transcript, sense intronic to N6AMT2 | 6,00 |
| gene:ENSG00000275532 | [ENSG00000275532](https://www.ensembl.org/id/ENSG00000275532) | novel transcript, antisense to MLLT6 | 3,73 |
| gene:ENSG00000276166 | [ENSG00000276166](https://www.ensembl.org/id/ENSG00000276166) | novel transcript, antisense to KIFC3 | -4,80 |
| gene:ENSG00000276417 | [ENSG00000276417](https://www.ensembl.org/id/ENSG00000276417) | novel transcript | 3,34 |
| gene:ENSG00000276850 | [ENSG00000276850](https://www.ensembl.org/id/ENSG00000276850) | novel transcript | -7,70 |
| gene:ENSG00000276952 | [ENSG00000276952](https://www.ensembl.org/id/ENSG00000276952) | novel transcript, antisense to PYGB | -2,65 |
| gene:ENSG00000277135 | [ENSG00000277135](https://www.ensembl.org/id/ENSG00000277135) | novel transcript | -2,84 |
| gene:ENSG00000277152 | [ENSG00000277152](https://www.ensembl.org/id/ENSG00000277152) | novel transcript | -2,56 |
| gene:ENSG00000277299 | [ENSG00000277299](https://www.ensembl.org/id/ENSG00000277299) | novel transcript, antisense to GIT2 | -5,14 |
| gene:ENSG00000277701 | [ENSG00000277701](https://www.ensembl.org/id/ENSG00000277701) | novel transcript | -1,38 |
| gene:ENSG00000277999 | [ENSG00000277999](https://www.ensembl.org/id/ENSG00000277999) | novel transcript | 3,61 |
| gene:ENSG00000278022 | [ENSG00000278022](https://www.ensembl.org/id/ENSG00000278022) | novel transcript, sense intronic to IGF1R | -3,74 |
| gene:ENSG00000278254 | [ENSG00000278254](https://www.ensembl.org/id/ENSG00000278254) | novel transcript | 3,93 |
| gene:ENSG00000278396 | [ENSG00000278396](https://www.ensembl.org/id/ENSG00000278396) | novel transcript, sense intronic UNC79 | -3,67 |
| gene:ENSG00000278831 | [ENSG00000278831](https://www.ensembl.org/id/ENSG00000278831) | novel transcript, antisense to FAM53B | -2,82 |
| gene:ENSG00000278903 | [ENSG00000278903](https://www.ensembl.org/id/ENSG00000278903) | novel transcript | 2,66 |
| gene:ENSG00000278946 | [ENSG00000278946](https://www.ensembl.org/id/ENSG00000278946) | novel transcript, antisense to PCDHA10 | 6,19 |
| gene:ENSG00000279182 | [ENSG00000279182](https://www.ensembl.org/id/ENSG00000279182) | novel transcript, antisense to DENND6B | -3,18 |
| gene:ENSG00000279232 | [ENSG00000279232](https://www.ensembl.org/id/ENSG00000279232) | novel transcript, antisense to RGMB | -4,93 |
| gene:ENSG00000279686 | [ENSG00000279686](https://www.ensembl.org/id/ENSG00000279686) | novel transcript | -6,44 |
| gene:ENSG00000280145 | [ENSG00000280145](https://www.ensembl.org/id/ENSG00000280145) | novel transcript | 2,61 |
| gene:ENSG00000280341 | [ENSG00000280341](https://www.ensembl.org/id/ENSG00000280341) | novel transcript | 6,27 |
| gene:ENSG00000280433 | [ENSG00000280433](https://www.ensembl.org/id/ENSG00000280433) | novel protein, similar to trafficking protein particle complex 10 TRAPPC10 | -1,05 |
| gene:ENSG00000280445 | [ENSG00000280445](https://www.ensembl.org/id/ENSG00000280445) | novel transcript | 5,19 |
| gene:ENSG00000280660 | [ENSG00000280660](https://www.ensembl.org/id/ENSG00000280660) | novel transcript, sense intronic to LGI1 | 6,37 |
| gene:ENSG00000281383 | [ENSG00000281383](https://www.ensembl.org/id/ENSG00000281383) | novel transcript, similar to YY1 associated myogenesis RNA 1 YAM1 | 1,11 |
| gene:ENSG00000282033 | [ENSG00000282033](https://www.ensembl.org/id/ENSG00000282033) | novel transcript | 1,86 |
| gene:ENSG00000282572 | [ENSG00000282572](https://www.ensembl.org/id/ENSG00000282572) | family with sequence similarity 157 member D [Source:HGNC Symbol;Acc:HGNC:56252] | 6,18 |
| gene:ENSG00000282859 | [ENSG00000282859](https://www.ensembl.org/id/ENSG00000282859) | novel transcript, sense intronic to FOXP2 | 5,25 |
| gene:ENSG00000283064 | [ENSG00000283064](https://www.ensembl.org/id/ENSG00000283064) | novel transcript, antisense to HIST1H2BD | 4,22 |
| gene:ENSG00000283213 | [ENSG00000283213](https://www.ensembl.org/id/ENSG00000283213) | novel transcript | -2,47 |
| gene:ENSG00000283403 | [ENSG00000283403](https://www.ensembl.org/id/ENSG00000283403) | novel transcript | 4,87 |
| gene:ENSG00000283459 | [ENSG00000283459](https://www.ensembl.org/id/ENSG00000283459) | novel transcript | 4,09 |
| gene:ENSG00000283662 | [ENSG00000283662](https://www.ensembl.org/id/ENSG00000283662) | novel transcript | 2,72 |
| gene:ENSG00000283757 | [ENSG00000283757](https://www.ensembl.org/id/ENSG00000283757) | novel transcript | 3,12 |
| gene:ENSG00000283782 | [ENSG00000283782](https://www.ensembl.org/id/ENSG00000283782) | novel protein | 1,79 |
| gene:ENSG00000284685 | [ENSG00000284685](https://www.ensembl.org/id/ENSG00000284685) | novel transcript | 4,08 |
| gene:ENSG00000284720 | [ENSG00000284720](https://www.ensembl.org/id/ENSG00000284720) | novel transcript | 5,19 |
| gene:ENSG00000284948 | [ENSG00000284948](https://www.ensembl.org/id/ENSG00000284948) | novel transcript | 3,72 |
| gene:ENSG00000284952 | [ENSG00000284952](https://www.ensembl.org/id/ENSG00000284952) | novel transcript | 6,32 |
| gene:ENSG00000284977 | [ENSG00000284977](https://www.ensembl.org/id/ENSG00000284977) | novel transcript | 2,55 |
| gene:ENSG00000285043 | [ENSG00000285043](https://www.ensembl.org/id/ENSG00000285043) | novel protein | 1,57 |
| gene:ENSG00000285082 | [ENSG00000285082](https://www.ensembl.org/id/ENSG00000285082) | novel protein | 5,00 |
| gene:ENSG00000285238 | [ENSG00000285238](https://www.ensembl.org/id/ENSG00000285238) | novel transcript | -8,96 |
| gene:ENSG00000285555 | [ENSG00000285555](https://www.ensembl.org/id/ENSG00000285555) | novel transcript, antisense to CACNA1C | 5,13 |
| gene:ENSG00000285563 | [ENSG00000285563](https://www.ensembl.org/id/ENSG00000285563) | novel transcript | 7,17 |
| gene:ENSG00000285610 | [ENSG00000285610](https://www.ensembl.org/id/ENSG00000285610) | novel transcript, antisense to PACRG | 5,28 |
| gene:ENSG00000285658 | [ENSG00000285658](https://www.ensembl.org/id/ENSG00000285658) | novel transcript, antisense to AMBRA1 | 6,87 |
| gene:ENSG00000285681 | [ENSG00000285681](https://www.ensembl.org/id/ENSG00000285681) | novel transcript, antisense to MC4R | 5,44 |
| gene:ENSG00000285713 | [ENSG00000285713](https://www.ensembl.org/id/ENSG00000285713) | novel transcript | 4,46 |
| gene:ENSG00000285750 | [ENSG00000285750](https://www.ensembl.org/id/ENSG00000285750) | novel transcript | 6,39 |
| gene:ENSG00000285868 | [ENSG00000285868](https://www.ensembl.org/id/ENSG00000285868) | Novel protein | -4,20 |
| gene:ENSG00000285920 | [ENSG00000285920](https://www.ensembl.org/id/ENSG00000285920) | novel protein | 4,02 |
| gene:ENSG00000285987 | [ENSG00000285987](https://www.ensembl.org/id/ENSG00000285987) | Novel transcript, antisense to SLC28A3 | 6,27 |
| gene:ENSG00000286009 | [ENSG00000286009](https://www.ensembl.org/id/ENSG00000286009) | novel transcript, antisense to USP9Y | 3,49 |
| gene:ENSG00000286164 | [ENSG00000286164](https://www.ensembl.org/id/ENSG00000286164) | novel transcript, antisense to OXCT1 | 5,83 |
| gene:ENSG00000286174 | [ENSG00000286174](https://www.ensembl.org/id/ENSG00000286174) | novel transcript, antisense to CNIH3 | 5,20 |
| gene:ENSG00000286185 | [ENSG00000286185](https://www.ensembl.org/id/ENSG00000286185) | novel protein, identical to neuroblastoma breakpoint family, member 19 NBPF19 | -2,94 |
| gene:ENSG00000286194 | [ENSG00000286194](https://www.ensembl.org/id/ENSG00000286194) | Novel transcript | 5,69 |
| gene:ENSG00000286235 | [ENSG00000286235](https://www.ensembl.org/id/ENSG00000286235) | novel protein | 7,79 |
| gene:ENSG00000286257 | [ENSG00000286257](https://www.ensembl.org/id/ENSG00000286257) | novel transcript | 8,13 |
| gene:ENSG00000286292 | [ENSG00000286292](https://www.ensembl.org/id/ENSG00000286292) | novel transcript | 2,44 |
| gene:ENSG00000286293 | [ENSG00000286293](https://www.ensembl.org/id/ENSG00000286293) | novel transcript | -5,51 |
| gene:ENSG00000286305 | [ENSG00000286305](https://www.ensembl.org/id/ENSG00000286305) | novel transcript, antisense to TRRAP | -2,26 |
| gene:ENSG00000286326 | [ENSG00000286326](https://www.ensembl.org/id/ENSG00000286326) | novel transcript, antisense to CRYBB1 | 6,17 |
| gene:ENSG00000286415 | [ENSG00000286415](https://www.ensembl.org/id/ENSG00000286415) | novel transcript | 5,14 |
| gene:ENSG00000286445 | [ENSG00000286445](https://www.ensembl.org/id/ENSG00000286445) | novel transcript | 3,38 |
| gene:ENSG00000286458 | [ENSG00000286458](https://www.ensembl.org/id/ENSG00000286458) | novel transcript, antisense to AGBL3and CALD1 | 2,57 |
| gene:ENSG00000286463 | [ENSG00000286463](https://www.ensembl.org/id/ENSG00000286463) | novel transcript, antisense to ZBTB16 | 4,98 |
| gene:ENSG00000286512 | [ENSG00000286512](https://www.ensembl.org/id/ENSG00000286512) | novel transcript | -3,53 |
| gene:ENSG00000286534 | [ENSG00000286534](https://www.ensembl.org/id/ENSG00000286534) | novel transcript | -4,89 |
| gene:ENSG00000286561 | [ENSG00000286561](https://www.ensembl.org/id/ENSG00000286561) | novel transcript, antisense to SBF2 | 5,98 |
| gene:ENSG00000286618 | [ENSG00000286618](https://www.ensembl.org/id/ENSG00000286618) | novel transcript, antisense to PKD2and SPP1 | 5,28 |
| gene:ENSG00000286658 | [ENSG00000286658](https://www.ensembl.org/id/ENSG00000286658) | novel transcript, antisense to GRB10 | 3,75 |
| gene:ENSG00000286690 | [ENSG00000286690](https://www.ensembl.org/id/ENSG00000286690) | novel transcript, antisense to SGCD | 4,91 |
| gene:ENSG00000286713 | [ENSG00000286713](https://www.ensembl.org/id/ENSG00000286713) | novel transcript | 6,91 |
| gene:ENSG00000286729 | [ENSG00000286729](https://www.ensembl.org/id/ENSG00000286729) | novel transcript, antisense to COPG1 | -2,61 |
| gene:ENSG00000286788 | [ENSG00000286788](https://www.ensembl.org/id/ENSG00000286788) | novel transcript, antisense to LPO | 5,46 |
| gene:ENSG00000286907 | [ENSG00000286907](https://www.ensembl.org/id/ENSG00000286907) | novel transcript | 5,12 |
| gene:ENSG00000286986 | [ENSG00000286986](https://www.ensembl.org/id/ENSG00000286986) | novel transcript | 6,04 |
| gene:ENSG00000287026 | [ENSG00000287026](https://www.ensembl.org/id/ENSG00000287026) | novel transcript | 6,79 |
| gene:ENSG00000287048 | [ENSG00000287048](https://www.ensembl.org/id/ENSG00000287048) | novel transcript | 6,04 |
| gene:ENSG00000287081 | [ENSG00000287081](https://www.ensembl.org/id/ENSG00000287081) | novel transcript, antisense to LDB2 | -5,20 |
| gene:ENSG00000287092 | [ENSG00000287092](https://www.ensembl.org/id/ENSG00000287092) | novel transcript | 5,55 |
| gene:ENSG00000287124 | [ENSG00000287124](https://www.ensembl.org/id/ENSG00000287124) | novel transcript | 5,47 |
| gene:ENSG00000287130 | [ENSG00000287130](https://www.ensembl.org/id/ENSG00000287130) | novel transcript | 5,62 |
| gene:ENSG00000287193 | [ENSG00000287193](https://www.ensembl.org/id/ENSG00000287193) | novel transcript | 6,39 |
| gene:ENSG00000287214 | [ENSG00000287214](https://www.ensembl.org/id/ENSG00000287214) | novel transcript, sense intronic to ARFGEF1 | 5,59 |
| gene:ENSG00000287237 | [ENSG00000287237](https://www.ensembl.org/id/ENSG00000287237) | novel transcript, antisense to PLXNC1 | 4,27 |
| gene:ENSG00000287252 | [ENSG00000287252](https://www.ensembl.org/id/ENSG00000287252) | novel transcript | -3,74 |
| gene:ENSG00000287258 | [ENSG00000287258](https://www.ensembl.org/id/ENSG00000287258) | novel transcript, antisense to PKIB | -5,78 |
| gene:ENSG00000287272 | [ENSG00000287272](https://www.ensembl.org/id/ENSG00000287272) | novel transcript | 5,51 |
| gene:ENSG00000287292 | [ENSG00000287292](https://www.ensembl.org/id/ENSG00000287292) | novel transcript | 4,70 |
| gene:ENSG00000287303 | [ENSG00000287303](https://www.ensembl.org/id/ENSG00000287303) | novel transcript, antisense to PDGFRL | -5,27 |
| gene:ENSG00000287307 | [ENSG00000287307](https://www.ensembl.org/id/ENSG00000287307) | novel transcript | 5,43 |
| gene:ENSG00000287309 | [ENSG00000287309](https://www.ensembl.org/id/ENSG00000287309) | novel transcript | 3,96 |
| gene:ENSG00000287381 | [ENSG00000287381](https://www.ensembl.org/id/ENSG00000287381) | novel transcript, antisense to MAPK4 | 3,24 |
| gene:ENSG00000287385 | [ENSG00000287385](https://www.ensembl.org/id/ENSG00000287385) | novel transcript, antisense to RAD51B | -4,98 |
| gene:ENSG00000287437 | [ENSG00000287437](https://www.ensembl.org/id/ENSG00000287437) | novel transcript, antisense to NRDE2 | 3,38 |
| gene:ENSG00000287458 | [ENSG00000287458](https://www.ensembl.org/id/ENSG00000287458) | novel transcript, antisense to FKBP5 | 4,97 |
| gene:ENSG00000287469 | [ENSG00000287469](https://www.ensembl.org/id/ENSG00000287469) | novel transcript | 5,45 |
| gene:ENSG00000287490 | [ENSG00000287490](https://www.ensembl.org/id/ENSG00000287490) | novel transcript | -5,05 |
| gene:ENSG00000287502 | [ENSG00000287502](https://www.ensembl.org/id/ENSG00000287502) | novel transcript | 5,43 |
| gene:ENSG00000287535 | [ENSG00000287535](https://www.ensembl.org/id/ENSG00000287535) | novel transcript | 5,90 |
| gene:ENSG00000287670 | [ENSG00000287670](https://www.ensembl.org/id/ENSG00000287670) | novel transcript | 5,71 |
| gene:ENSG00000287672 | [ENSG00000287672](https://www.ensembl.org/id/ENSG00000287672) | novel transcript | 5,04 |
| gene:ENSG00000287692 | [ENSG00000287692](https://www.ensembl.org/id/ENSG00000287692) | novel transcript | 4,10 |
| gene:ENSG00000287707 | [ENSG00000287707](https://www.ensembl.org/id/ENSG00000287707) | novel transcript | -2,79 |
| gene:ENSG00000287712 | [ENSG00000287712](https://www.ensembl.org/id/ENSG00000287712) | novel transcript | -3,36 |
| gene:ENSG00000287715 | [ENSG00000287715](https://www.ensembl.org/id/ENSG00000287715) | novel transcript | 4,89 |
| gene:ENSG00000287804 | [ENSG00000287804](https://www.ensembl.org/id/ENSG00000287804) | novel transcript | -2,68 |
| gene:ENSG00000287811 | [ENSG00000287811](https://www.ensembl.org/id/ENSG00000287811) | novel transcript | -2,48 |
| gene:ENSG00000287891 | [ENSG00000287891](https://www.ensembl.org/id/ENSG00000287891) | novel transcript | 6,70 |
| gene:ENSG00000287908 | [ENSG00000287908](https://www.ensembl.org/id/ENSG00000287908) | novel protein | -1,31 |
| gene:ENSG00000287919 | [ENSG00000287919](https://www.ensembl.org/id/ENSG00000287919) | novel transcript | 6,14 |
| gene:ENSG00000287958 | [ENSG00000287958](https://www.ensembl.org/id/ENSG00000287958) | novel transcript | 5,12 |
| gene:ENSG00000287979 | [ENSG00000287979](https://www.ensembl.org/id/ENSG00000287979) | novel transcript | -2,78 |
| gene:ENSG00000288068 | [ENSG00000288068](https://www.ensembl.org/id/ENSG00000288068) | novel transcript, sense intronic to NOX5 | 6,83 |
| gene:ENSG00000288582 | [ENSG00000288582](https://www.ensembl.org/id/ENSG00000288582) | Novel transcript | 5,67 |
| gene:ENSG00000288587 | [ENSG00000288587](https://www.ensembl.org/id/ENSG00000288587) | novel transcript | 6,38 |
| gene:ENSG00000288598 | [ENSG00000288598](https://www.ensembl.org/id/ENSG00000288598) | novel transcript | 1,96 |
| gene:ENSG00000288636 | [ENSG00000288636](https://www.ensembl.org/id/ENSG00000288636) | novel protein | 6,14 |
| gene:ENSG00000288637 | [ENSG00000288637](https://www.ensembl.org/id/ENSG00000288637) | novel protein | -5,17 |
| gene:ENSG00000288663 | [ENSG00000288663](https://www.ensembl.org/id/ENSG00000288663) | novel protein, readthrough PFDN5-C12orf10 | -1,22 |
| gene:ENSG00000288670 | [ENSG00000288670](https://www.ensembl.org/id/ENSG00000288670) | novel transcript | 1,62 |
| gene:ENSG00000288684 | [ENSG00000288684](https://www.ensembl.org/id/ENSG00000288684) | novel protein | 8,43 |
| gene:ENSG00000288703 | [ENSG00000288703](https://www.ensembl.org/id/ENSG00000288703) | novel transcript | 6,55 |
| gene:ENSG00000288758 | [ENSG00000288758](https://www.ensembl.org/id/ENSG00000288758) | novel transcript, antisense to FSTL4 | 3,17 |
| gene:ENSG00000288761 | [ENSG00000288761](https://www.ensembl.org/id/ENSG00000288761) | novel transcript, antisense to SNAI2 | -2,66 |
| gene:ENSG00000288777 | [ENSG00000288777](https://www.ensembl.org/id/ENSG00000288777) | novel transcript, sense intronic to ANKRD54and MYH9 | 8,01 |
| gene:ENSG00000288790 | [ENSG00000288790](https://www.ensembl.org/id/ENSG00000288790) | novel transcript | 5,85 |
| gene:ENSG00000288794 | [ENSG00000288794](https://www.ensembl.org/id/ENSG00000288794) | novel transcript, sense intronic to LPP | 2,61 |
| gene:ENSG00000288796 | [ENSG00000288796](https://www.ensembl.org/id/ENSG00000288796) | novel protein | 3,95 |
| gene:ENSG00000288826 | [ENSG00000288826](https://www.ensembl.org/id/ENSG00000288826) | novel transcript | -2,27 |
| gene:ENSG00000288849 | [ENSG00000288849](https://www.ensembl.org/id/ENSG00000288849) | novel transcript | 1,50 |
| gene:ENSG00000288854 | [ENSG00000288854](https://www.ensembl.org/id/ENSG00000288854) | novel transcript | 4,26 |
| gene:ENSG00000288882 | [ENSG00000288882](https://www.ensembl.org/id/ENSG00000288882) | novel transcript | 7,95 |
| gene:ENSG00000288891 | [ENSG00000288891](https://www.ensembl.org/id/ENSG00000288891) | novel transcript | 2,33 |
| gene:ENSG00000288894 | [ENSG00000288894](https://www.ensembl.org/id/ENSG00000288894) | novel protein | -5,50 |
| gene:ENSG00000288900 | [ENSG00000288900](https://www.ensembl.org/id/ENSG00000288900) | novel transcript | -4,74 |
| gene:ENSG00000288906 | [ENSG00000288906](https://www.ensembl.org/id/ENSG00000288906) | novel transcript | -3,58 |
| gene:ENSG00000288912 | [ENSG00000288912](https://www.ensembl.org/id/ENSG00000288912) | novel transcript | 5,56 |
| gene:ENSG00000288935 | [ENSG00000288935](https://www.ensembl.org/id/ENSG00000288935) | novel transcript | 3,00 |
| gene:ENSG00000288960 | [ENSG00000288960](https://www.ensembl.org/id/ENSG00000288960) | novel transcript | 5,83 |
| gene:ENSG00000289027 | [ENSG00000289027](https://www.ensembl.org/id/ENSG00000289027) | novel protein | 4,17 |
| gene:ENSG00000289062 | [ENSG00000289062](https://www.ensembl.org/id/ENSG00000289062) | novel transcript, antisense to IVL | 6,80 |
| gene:ENSG00000289097 | [ENSG00000289097](https://www.ensembl.org/id/ENSG00000289097) | novel transcript | 5,38 |
| gene:ENSG00000289100 | [ENSG00000289100](https://www.ensembl.org/id/ENSG00000289100) | novel transcript, antisense to TAPBP | -0,99 |
| gene:ENSG00000289120 | [ENSG00000289120](https://www.ensembl.org/id/ENSG00000289120) | novel transcript | 5,00 |
| gene:ENSG00000289130 | [ENSG00000289130](https://www.ensembl.org/id/ENSG00000289130) | novel transcript, antisense to THRB | 4,45 |
| gene:ENSG00000289137 | [ENSG00000289137](https://www.ensembl.org/id/ENSG00000289137) | novel transcript | 6,57 |
| gene:ENSG00000289146 | [ENSG00000289146](https://www.ensembl.org/id/ENSG00000289146) | novel transcript | 4,78 |
| gene:ENSG00000289176 | [ENSG00000289176](https://www.ensembl.org/id/ENSG00000289176) | novel transcript | 4,67 |
| gene:ENSG00000289187 | [ENSG00000289187](https://www.ensembl.org/id/ENSG00000289187) | novel transcript, sense intronic to ZNF536 | 5,43 |
| gene:ENSG00000289208 | [ENSG00000289208](https://www.ensembl.org/id/ENSG00000289208) | novel transcript, sense intronic to GPHN | 6,85 |
| gene:ENSG00000289216 | [ENSG00000289216](https://www.ensembl.org/id/ENSG00000289216) | novel transcript, sense intronic to GUCA1B | 4,45 |
| gene:ENSG00000289251 | [ENSG00000289251](https://www.ensembl.org/id/ENSG00000289251) | novel transcript | -3,05 |
| gene:ENSG00000289258 | [ENSG00000289258](https://www.ensembl.org/id/ENSG00000289258) | novel protein | -3,49 |
| gene:ENSG00000289269 | [ENSG00000289269](https://www.ensembl.org/id/ENSG00000289269) | novel transcript, sense intronic to CDK13 | -3,56 |
| gene:ENSG00000289288 | [ENSG00000289288](https://www.ensembl.org/id/ENSG00000289288) | novel transcript | 3,54 |
| gene:ENSG00000289298 | [ENSG00000289298](https://www.ensembl.org/id/ENSG00000289298) | novel transcript | -2,68 |
| gene:ENSG00000289328 | [ENSG00000289328](https://www.ensembl.org/id/ENSG00000289328) | novel transcript | 4,13 |
| gene:ENSG00000289351 | [ENSG00000289351](https://www.ensembl.org/id/ENSG00000289351) | novel transcript | 5,44 |
| gene:ENSG00000289365 | [ENSG00000289365](https://www.ensembl.org/id/ENSG00000289365) | novel transcript | -3,15 |
| gene:ENSG00000289376 | [ENSG00000289376](https://www.ensembl.org/id/ENSG00000289376) | novel transcript, sense overlapping FRK | -1,94 |
| gene:ENSG00000289379 | [ENSG00000289379](https://www.ensembl.org/id/ENSG00000289379) | novel transcript | 5,65 |
| gene:ENSG00000289405 | [ENSG00000289405](https://www.ensembl.org/id/ENSG00000289405) | novel transcript | -3,18 |
| gene:ENSG00000289423 | [ENSG00000289423](https://www.ensembl.org/id/ENSG00000289423) | novel transcript | -4,33 |
| gene:ENSG00000289446 | [ENSG00000289446](https://www.ensembl.org/id/ENSG00000289446) | novel transcript | 4,87 |
| gene:ENSG00000289463 | [ENSG00000289463](https://www.ensembl.org/id/ENSG00000289463) | novel transcript, sense intronic to ZNF638 | -3,10 |
| gene:ENSG00000289511 | [ENSG00000289511](https://www.ensembl.org/id/ENSG00000289511) | novel transcript | -2,81 |
| gene:ENSG00000289526 | [ENSG00000289526](https://www.ensembl.org/id/ENSG00000289526) | novel transcript | 8,02 |
| gene:ENSG00000289527 | [ENSG00000289527](https://www.ensembl.org/id/ENSG00000289527) | novel transcript, antisense to PRKG1 | -5,28 |
| gene:ENSG00000289528 | [ENSG00000289528](https://www.ensembl.org/id/ENSG00000289528) | novel transcript, sense intronic to MLLT10 | 7,02 |
| gene:ENSG00000289546 | [ENSG00000289546](https://www.ensembl.org/id/ENSG00000289546) | novel transcript, sense intronic to C21orf33and AGPAT3 | 3,64 |
| gene:ENSG00000289550 | [ENSG00000289550](https://www.ensembl.org/id/ENSG00000289550) | novel transcript | -6,02 |
| gene:ENSG00000289587 | [ENSG00000289587](https://www.ensembl.org/id/ENSG00000289587) | novel transcript, antisense to PRKCA | 6,60 |
| gene:ENSG00000289594 | [ENSG00000289594](https://www.ensembl.org/id/ENSG00000289594) | novel transcript, antisense to TMTC4 | 3,72 |
| gene:ENSG00000289601 | [ENSG00000289601](https://www.ensembl.org/id/ENSG00000289601) | novel transcript, antisense to CDH6 | 3,35 |
| gene:ENSG00000289621 | [ENSG00000289621](https://www.ensembl.org/id/ENSG00000289621) | novel transcript | 5,10 |
| gene:ENSG00000289690 | [ENSG00000289690](https://www.ensembl.org/id/ENSG00000289690) | novel transcript | 5,72 |
| gene:ENSG00000289692 | [ENSG00000289692](https://www.ensembl.org/id/ENSG00000289692) | novel protein | 5,40 |
| gene:ENSG00000289747 | [ENSG00000289747](https://www.ensembl.org/id/ENSG00000289747) | novel protein | 6,40 |
| gene:ENSG00000289840 | [ENSG00000289840](https://www.ensembl.org/id/ENSG00000289840) | novel transcript, antisense to KIF26A | -5,10 |
| gene:ENSG00000289874 | [ENSG00000289874](https://www.ensembl.org/id/ENSG00000289874) | novel transcript | -5,51 |
| gene:ENSG00000289884 | [ENSG00000289884](https://www.ensembl.org/id/ENSG00000289884) | novel transcript | -5,20 |
| gene:ENSG00000289909 | [ENSG00000289909](https://www.ensembl.org/id/ENSG00000289909) | novel transcript | -3,11 |
| gene:ENSG00000289973 | [ENSG00000289973](https://www.ensembl.org/id/ENSG00000289973) | novel transcript, sense intronic to MYH6 | 7,06 |
| gene:ENSG00000290007 | [ENSG00000290007](https://www.ensembl.org/id/ENSG00000290007) | novel transcript | -4,59 |
| gene:ENSG00000290029 | [ENSG00000290029](https://www.ensembl.org/id/ENSG00000290029) | novel transcript | 6,13 |
| gene:ENSG00000290034 | [ENSG00000290034](https://www.ensembl.org/id/ENSG00000290034) | novel transcript, antisense to TREM2 | 4,58 |
| gene:ENSG00000290126 | [ENSG00000290126](https://www.ensembl.org/id/ENSG00000290126) | novel transcript | 3,32 |
| gene:ENSG00000290242 | [ENSG00000290242](https://www.ensembl.org/id/ENSG00000290242) | novel transcript | -5,48 |
| gene:ENSG00000290315 | [ENSG00000290315](https://www.ensembl.org/id/ENSG00000290315) | novel protein | -2,18 |
| gene:ENSG00000290455 | [ENSG00000290455](https://www.ensembl.org/id/ENSG00000290455) | novel transcript | -3,08 |
| gene:ENSG00000290537 | [ENSG00000290537](https://www.ensembl.org/id/ENSG00000290537) | novel transcript | -2,18 |
| gene:ENSG00000290547 | [ENSG00000290547](https://www.ensembl.org/id/ENSG00000290547) | novel transcript | 3,39 |
| gene:ENSG00000290585 | [ENSG00000290585](https://www.ensembl.org/id/ENSG00000290585) | novel transcript | 3,29 |
| gene:ENSG00000290586 | [ENSG00000290586](https://www.ensembl.org/id/ENSG00000290586) | novel transcript | 3,36 |
| gene:ENSG00000290655 | [ENSG00000290655](https://www.ensembl.org/id/ENSG00000290655) | novel transcript | 3,63 |
| gene:ENSG00000290685 | [ENSG00000290685](https://www.ensembl.org/id/ENSG00000290685) | novel transcript | 5,87 |
| gene:ENSG00000290731 | [ENSG00000290731](https://www.ensembl.org/id/ENSG00000290731) | novel transcript | -1,84 |
| gene:ENSG00000290787 | [ENSG00000290787](https://www.ensembl.org/id/ENSG00000290787) | novel transcript | 3,88 |
| gene:ENSG00000290884 | [ENSG00000290884](https://www.ensembl.org/id/ENSG00000290884) | novel transcript | 5,19 |
| gene:ENSG00000290990 | [ENSG00000290990](https://www.ensembl.org/id/ENSG00000290990) | novel transcript | 4,95 |
| gene:ENSG00000291048 | [ENSG00000291048](https://www.ensembl.org/id/ENSG00000291048) | novel transcript | 2,99 |
| gene:ENSG00000291061 | [ENSG00000291061](https://www.ensembl.org/id/ENSG00000291061) | novel transcript | -6,01 |
| gene:ENSG00000291144 | [ENSG00000291144](https://www.ensembl.org/id/ENSG00000291144) | novel transcript | 4,37 |
| gene:ENSG00000291166 | [ENSG00000291166](https://www.ensembl.org/id/ENSG00000291166) | novel transcript | -4,79 |
| gene:ENSG00000291184 | [ENSG00000291184](https://www.ensembl.org/id/ENSG00000291184) | novel transcript | 6,27 |
| gene:ENSG00000291194 | [ENSG00000291194](https://www.ensembl.org/id/ENSG00000291194) | novel transcript | -1,82 |
| gene:ENSG00000291209 | [ENSG00000291209](https://www.ensembl.org/id/ENSG00000291209) | novel transcript | 5,65 |
| gene:ENSG00000291211 | [ENSG00000291211](https://www.ensembl.org/id/ENSG00000291211) | novel transcript | -1,50 |
| gene:ENSG00000291230 | [ENSG00000291230](https://www.ensembl.org/id/ENSG00000291230) | novel transcript | 3,38 |
| gene:ENSG00000291233 | [ENSG00000291233](https://www.ensembl.org/id/ENSG00000291233) | novel transcript | 2,13 |
| gene:ENSG00000291236 | [ENSG00000291236](https://www.ensembl.org/id/ENSG00000291236) | novel transcript | 7,06 |
| gene:ENSG00000291260 | [ENSG00000291260](https://www.ensembl.org/id/ENSG00000291260) | novel transcript | 3,82 |
| GFM1 | [ENSG00000168827](https://www.ensembl.org/id/ENSG00000168827) | G elongation factor mitochondrial 1 [Source:HGNC Symbol;Acc:HGNC:13780] | 1,01 |
| GFM2 | [ENSG00000164347](https://www.ensembl.org/id/ENSG00000164347) | GTP dependent ribosome recycling factor mitochondrial 2 [Source:HGNC Symbol;Acc:HGNC:29682] | 1,28 |
| GFPT2 | [ENSG00000131459](https://www.ensembl.org/id/ENSG00000131459) | glutamine-fructose-6-phosphate transaminase 2 [Source:HGNC Symbol;Acc:HGNC:4242] | -1,97 |
| GHET1 | [ENSG00000281189](https://www.ensembl.org/id/ENSG00000281189) | gastric carcinoma proliferation enhancing transcript 1 [Source:HGNC Symbol;Acc:HGNC:49425] | 4,09 |
| GHRLOS | [ENSG00000240288](https://www.ensembl.org/id/ENSG00000240288) | ghrelin opposite strand/antisense RNA [Source:HGNC Symbol;Acc:HGNC:33885] | -1,88 |
| GINS1 | [ENSG00000101003](https://www.ensembl.org/id/ENSG00000101003) | GINS complex subunit 1 [Source:HGNC Symbol;Acc:HGNC:28980] | -2,20 |
| GINS2 | [ENSG00000131153](https://www.ensembl.org/id/ENSG00000131153) | GINS complex subunit 2 [Source:HGNC Symbol;Acc:HGNC:24575] | 1,94 |
| GJA1 | [ENSG00000152661](https://www.ensembl.org/id/ENSG00000152661) | gap junction protein alpha 1 [Source:HGNC Symbol;Acc:HGNC:4274] | -1,52 |
| GJB2 | [ENSG00000165474](https://www.ensembl.org/id/ENSG00000165474) | gap junction protein beta 2 [Source:HGNC Symbol;Acc:HGNC:4284] | -3,09 |
| GJB5 | [ENSG00000189280](https://www.ensembl.org/id/ENSG00000189280) | gap junction protein beta 5 [Source:HGNC Symbol;Acc:HGNC:4287] | 3,52 |
| GJD2 | [ENSG00000159248](https://www.ensembl.org/id/ENSG00000159248) | gap junction protein delta 2 [Source:HGNC Symbol;Acc:HGNC:19154] | 5,90 |
| GJD2-DT | [ENSG00000250007](https://www.ensembl.org/id/ENSG00000250007) | GJD2 divergent transcript [Source:HGNC Symbol;Acc:HGNC:55560] | -1,81 |
| GK3P | [ENSG00000229894](https://www.ensembl.org/id/ENSG00000229894) | glycerol kinase 3 pseudogene [Source:HGNC Symbol;Acc:HGNC:4292] | 6,07 |
| GLRX5 | [ENSG00000182512](https://www.ensembl.org/id/ENSG00000182512) | glutaredoxin 5 [Source:HGNC Symbol;Acc:HGNC:20134] | 1,13 |
| GLYAT | [ENSG00000149124](https://www.ensembl.org/id/ENSG00000149124) | glycine-N-acyltransferase [Source:HGNC Symbol;Acc:HGNC:13734] | -3,19 |
| GLYATL1 | [ENSG00000166840](https://www.ensembl.org/id/ENSG00000166840) | glycine-N-acyltransferase like 1 [Source:HGNC Symbol;Acc:HGNC:30519] | -4,96 |
| GMPR | [ENSG00000137198](https://www.ensembl.org/id/ENSG00000137198) | guanosine monophosphate reductase [Source:HGNC Symbol;Acc:HGNC:4376] | 1,61 |
| GNAT1 | [ENSG00000114349](https://www.ensembl.org/id/ENSG00000114349) | G protein subunit alpha transducin 1 [Source:HGNC Symbol;Acc:HGNC:4393] | -4,06 |
| GNLY | [ENSG00000115523](https://www.ensembl.org/id/ENSG00000115523) | granulysin [Source:HGNC Symbol;Acc:HGNC:4414] | 2,57 |
| GNPAT | [ENSG00000116906](https://www.ensembl.org/id/ENSG00000116906) | glyceronephosphate O-acyltransferase [Source:HGNC Symbol;Acc:HGNC:4416] | 1,18 |
| GOLGA6L4 | [ENSG00000184206](https://www.ensembl.org/id/ENSG00000184206) | golgin A6 family like 4 [Source:HGNC Symbol;Acc:HGNC:27256] | -1,45 |
| GOLGA8M_1 | [ENSG00000188626](https://www.ensembl.org/id/ENSG00000188626) | golgin A8 family member M [Source:HGNC Symbol;Acc:HGNC:44404] | -1,73 |
| GOLGA8Q | [ENSG00000178115](https://www.ensembl.org/id/ENSG00000178115) | golgin A8 family member Q [Source:HGNC Symbol;Acc:HGNC:44408] | -1,66 |
| GOT1 | [ENSG00000120053](https://www.ensembl.org/id/ENSG00000120053) | glutamic-oxaloacetic transaminase 1 [Source:HGNC Symbol;Acc:HGNC:4432] | 2,35 |
| GOT2 | [ENSG00000125166](https://www.ensembl.org/id/ENSG00000125166) | glutamic-oxaloacetic transaminase 2 [Source:HGNC Symbol;Acc:HGNC:4433] | 2,13 |
| GPA33 | [ENSG00000143167](https://www.ensembl.org/id/ENSG00000143167) | glycoprotein A33 [Source:HGNC Symbol;Acc:HGNC:4445] | 3,67 |
| GPAM | [ENSG00000119927](https://www.ensembl.org/id/ENSG00000119927) | glycerol-3-phosphate acyltransferase, mitochondrial [Source:HGNC Symbol;Acc:HGNC:24865] | -1,62 |
| GPAT3 | [ENSG00000138678](https://www.ensembl.org/id/ENSG00000138678) | glycerol-3-phosphate acyltransferase 3 [Source:HGNC Symbol;Acc:HGNC:28157] | 1,64 |
| GPAT4-AS1 | [ENSG00000253133](https://www.ensembl.org/id/ENSG00000253133) | GPAT4 and GINS4 antisense RNA 1 [Source:HGNC Symbol;Acc:HGNC:55539] | -5,69 |
| GPD1L | [ENSG00000152642](https://www.ensembl.org/id/ENSG00000152642) | glycerol-3-phosphate dehydrogenase 1 like [Source:HGNC Symbol;Acc:HGNC:28956] | 1,57 |
| GPM6A | [ENSG00000150625](https://www.ensembl.org/id/ENSG00000150625) | glycoprotein M6A [Source:HGNC Symbol;Acc:HGNC:4460] | -1,95 |
| GPN3 | [ENSG00000111231](https://www.ensembl.org/id/ENSG00000111231) | GPN-loop GTPase 3 [Source:HGNC Symbol;Acc:HGNC:30186] | 1,19 |
| GPR157 | [ENSG00000180758](https://www.ensembl.org/id/ENSG00000180758) | G protein-coupled receptor 157 [Source:HGNC Symbol;Acc:HGNC:23687] | 2,07 |
| GPR18 | [ENSG00000125245](https://www.ensembl.org/id/ENSG00000125245) | G protein-coupled receptor 18 [Source:HGNC Symbol;Acc:HGNC:4472] | 3,68 |
| GPR26 | [ENSG00000154478](https://www.ensembl.org/id/ENSG00000154478) | G protein-coupled receptor 26 [Source:HGNC Symbol;Acc:HGNC:4481] | -2,77 |
| GPR6 | [ENSG00000146360](https://www.ensembl.org/id/ENSG00000146360) | G protein-coupled receptor 6 [Source:HGNC Symbol;Acc:HGNC:4515] | 5,19 |
| GPR61 | [ENSG00000156097](https://www.ensembl.org/id/ENSG00000156097) | G protein-coupled receptor 61 [Source:HGNC Symbol;Acc:HGNC:13300] | 5,41 |
| GPRIN3 | [ENSG00000185477](https://www.ensembl.org/id/ENSG00000185477) | GPRIN family member 3 [Source:HGNC Symbol;Acc:HGNC:27733] | 2,06 |
| GPSM3 | [ENSG00000213654](https://www.ensembl.org/id/ENSG00000213654) | G protein signaling modulator 3 [Source:HGNC Symbol;Acc:HGNC:13945] | 2,19 |
| GPT | [ENSG00000167701](https://www.ensembl.org/id/ENSG00000167701) | glutamic--pyruvic transaminase [Source:HGNC Symbol;Acc:HGNC:4552] | 2,45 |
| GPT2 | [ENSG00000166123](https://www.ensembl.org/id/ENSG00000166123) | glutamic--pyruvic transaminase 2 [Source:HGNC Symbol;Acc:HGNC:18062] | 2,60 |
| GPX2 | [ENSG00000176153](https://www.ensembl.org/id/ENSG00000176153) | glutathione peroxidase 2 [Source:HGNC Symbol;Acc:HGNC:4554] | -5,17 |
| GRAP2 | [ENSG00000100351](https://www.ensembl.org/id/ENSG00000100351) | GRB2 related adaptor protein 2 [Source:HGNC Symbol;Acc:HGNC:4563] | 3,39 |
| GRB7 | [ENSG00000141738](https://www.ensembl.org/id/ENSG00000141738) | growth factor receptor bound protein 7 [Source:HGNC Symbol;Acc:HGNC:4567] | -2,35 |
| GRHL1 | [ENSG00000134317](https://www.ensembl.org/id/ENSG00000134317) | grainyhead like transcription factor 1 [Source:HGNC Symbol;Acc:HGNC:17923] | -1,89 |
| GRIK1-AS1 | [ENSG00000174680](https://www.ensembl.org/id/ENSG00000174680) | GRIK1 antisense RNA 1 [Source:HGNC Symbol;Acc:HGNC:16458] | -3,21 |
| GRIP2 | [ENSG00000144596](https://www.ensembl.org/id/ENSG00000144596) | glutamate receptor interacting protein 2 [Source:HGNC Symbol;Acc:HGNC:23841] | 1,63 |
| GRK3 | [ENSG00000100077](https://www.ensembl.org/id/ENSG00000100077) | G protein-coupled receptor kinase 3 [Source:HGNC Symbol;Acc:HGNC:290] | -1,44 |
| GRM3-AS1 | [ENSG00000233073](https://www.ensembl.org/id/ENSG00000233073) | GRM3 antisense RNA 1 [Source:HGNC Symbol;Acc:HGNC:40264] | 5,90 |
| GRM5-AS1 | [ENSG00000255082](https://www.ensembl.org/id/ENSG00000255082) | GRM5 antisense RNA 1 [Source:HGNC Symbol;Acc:HGNC:40265] | -5,60 |
| GRTP1-AS1 | [ENSG00000225083](https://www.ensembl.org/id/ENSG00000225083) | GRTP1 antisense RNA 1 [Source:HGNC Symbol;Acc:HGNC:39917] | 3,04 |
| GSDMB | [ENSG00000073605](https://www.ensembl.org/id/ENSG00000073605) | gasdermin B [Source:HGNC Symbol;Acc:HGNC:23690] | -1,21 |
| GSG1L | [ENSG00000169181](https://www.ensembl.org/id/ENSG00000169181) | GSG1 like [Source:HGNC Symbol;Acc:HGNC:28283] | -2,09 |
| GSR | [ENSG00000104687](https://www.ensembl.org/id/ENSG00000104687) | glutathione-disulfide reductase [Source:HGNC Symbol;Acc:HGNC:4623] | 1,01 |
| GSTM4 | [ENSG00000168765](https://www.ensembl.org/id/ENSG00000168765) | glutathione S-transferase mu 4 [Source:HGNC Symbol;Acc:HGNC:4636] | 1,18 |
| GSTO1 | [ENSG00000148834](https://www.ensembl.org/id/ENSG00000148834) | glutathione S-transferase omega 1 [Source:HGNC Symbol;Acc:HGNC:13312] | 0,89 |
| GTF3A | [ENSG00000122034](https://www.ensembl.org/id/ENSG00000122034) | general transcription factor IIIA [Source:HGNC Symbol;Acc:HGNC:4662] | 1,14 |
| GTF3C6 | [ENSG00000155115](https://www.ensembl.org/id/ENSG00000155115) | general transcription factor IIIC subunit 6 [Source:HGNC Symbol;Acc:HGNC:20872] | 1,04 |
| GTSF1 | [ENSG00000170627](https://www.ensembl.org/id/ENSG00000170627) | gametocyte specific factor 1 [Source:HGNC Symbol;Acc:HGNC:26565] | 5,64 |
| GUCY1A2 | [ENSG00000152402](https://www.ensembl.org/id/ENSG00000152402) | guanylate cyclase 1 soluble subunit alpha 2 [Source:HGNC Symbol;Acc:HGNC:4684] | 1,46 |
| GUF1 | [ENSG00000151806](https://www.ensembl.org/id/ENSG00000151806) | GTP binding elongation factor GUF1 [Source:HGNC Symbol;Acc:HGNC:25799] | 1,59 |
| GYG1 | [ENSG00000163754](https://www.ensembl.org/id/ENSG00000163754) | glycogenin 1 [Source:HGNC Symbol;Acc:HGNC:4699] | 1,50 |
| GYG2 | [ENSG00000056998](https://www.ensembl.org/id/ENSG00000056998) | glycogenin 2 [Source:HGNC Symbol;Acc:HGNC:4700] | -2,43 |
| GYS1 | [ENSG00000104812](https://www.ensembl.org/id/ENSG00000104812) | glycogen synthase 1 [Source:HGNC Symbol;Acc:HGNC:4706] | 1,69 |
| GZMH | [ENSG00000100450](https://www.ensembl.org/id/ENSG00000100450) | granzyme H [Source:HGNC Symbol;Acc:HGNC:4710] | 3,53 |
| H2AC6 | [ENSG00000180573](https://www.ensembl.org/id/ENSG00000180573) | H2A clustered histone 6 [Source:HGNC Symbol;Acc:HGNC:4733] | 1,30 |
| H2AX | [ENSG00000188486](https://www.ensembl.org/id/ENSG00000188486) | H2A.X variant histone [Source:HGNC Symbol;Acc:HGNC:4739] | -1,08 |
| H3-7 | [ENSG00000273213](https://www.ensembl.org/id/ENSG00000273213) | H3.7 histone (putative) [Source:HGNC Symbol;Acc:HGNC:32060] | -1,76 |
| H3C10 | [ENSG00000278828](https://www.ensembl.org/id/ENSG00000278828) | H3 clustered histone 10 [Source:HGNC Symbol;Acc:HGNC:4775] | 1,96 |
| HADH | [ENSG00000138796](https://www.ensembl.org/id/ENSG00000138796) | hydroxyacyl-CoA dehydrogenase [Source:HGNC Symbol;Acc:HGNC:4799] | 1,30 |
| HADHB | [ENSG00000138029](https://www.ensembl.org/id/ENSG00000138029) | hydroxyacyl-CoA dehydrogenase trifunctional multienzyme complex subunit beta [Source:HGNC Symbol;Acc:HGNC:4803] | 1,97 |
| HAGH | [ENSG00000063854](https://www.ensembl.org/id/ENSG00000063854) | hydroxyacylglutathione hydrolase [Source:HGNC Symbol;Acc:HGNC:4805] | 1,05 |
| HAS1 | [ENSG00000105509](https://www.ensembl.org/id/ENSG00000105509) | hyaluronan synthase 1 [Source:HGNC Symbol;Acc:HGNC:4818] | -3,24 |
| HBG1 | [ENSG00000213934](https://www.ensembl.org/id/ENSG00000213934) | hemoglobin subunit gamma 1 [Source:HGNC Symbol;Acc:HGNC:4831] | 6,56 |
| HBG2 | [ENSG00000196565](https://www.ensembl.org/id/ENSG00000196565) | hemoglobin subunit gamma 2 [Source:HGNC Symbol;Acc:HGNC:4832] | 6,74 |
| HBS1L | [ENSG00000112339](https://www.ensembl.org/id/ENSG00000112339) | HBS1 like translational GTPase [Source:HGNC Symbol;Acc:HGNC:4834] | 1,09 |
| HCG17 | [ENSG00000270604](https://www.ensembl.org/id/ENSG00000270604) | HLA complex group 17 [Source:HGNC Symbol;Acc:HGNC:31339] | 2,66 |
| HCG25 | [ENSG00000232940](https://www.ensembl.org/id/ENSG00000232940) | HLA complex group 25 [Source:HGNC Symbol;Acc:HGNC:20196] | -2,24 |
| HCK | [ENSG00000101336](https://www.ensembl.org/id/ENSG00000101336) | HCK proto-oncogene, Src family tyrosine kinase [Source:HGNC Symbol;Acc:HGNC:4840] | 2,23 |
| HDHD5 | [ENSG00000069998](https://www.ensembl.org/id/ENSG00000069998) | haloacid dehalogenase like hydrolase domain containing 5 [Source:HGNC Symbol;Acc:HGNC:1843] | 1,22 |
| HEPACAM | [ENSG00000165478](https://www.ensembl.org/id/ENSG00000165478) | hepatic and glial cell adhesion molecule [Source:HGNC Symbol;Acc:HGNC:26361] | -2,77 |
| HEPN1 | [ENSG00000221932](https://www.ensembl.org/id/ENSG00000221932) | hepatocellular carcinoma, down-regulated 1 [Source:HGNC Symbol;Acc:HGNC:34400] | -3,05 |
| HES5 | [ENSG00000197921](https://www.ensembl.org/id/ENSG00000197921) | hes family bHLH transcription factor 5 [Source:HGNC Symbol;Acc:HGNC:19764] | -2,38 |
| HGFAC | [ENSG00000109758](https://www.ensembl.org/id/ENSG00000109758) | HGF activator [Source:HGNC Symbol;Acc:HGNC:4894] | -3,53 |
| HHATL | [ENSG00000010282](https://www.ensembl.org/id/ENSG00000010282) | hedgehog acyltransferase like [Source:HGNC Symbol;Acc:HGNC:13242] | 3,23 |
| HIBADH | [ENSG00000106049](https://www.ensembl.org/id/ENSG00000106049) | 3-hydroxyisobutyrate dehydrogenase [Source:HGNC Symbol;Acc:HGNC:4907] | 1,21 |
| HIGD2A | [ENSG00000146066](https://www.ensembl.org/id/ENSG00000146066) | HIG1 hypoxia inducible domain family member 2A [Source:HGNC Symbol;Acc:HGNC:28311] | 1,22 |
| HILPDA-AS1 | [ENSG00000240758](https://www.ensembl.org/id/ENSG00000240758) | HILPDA antisense RNA 1 [Source:HGNC Symbol;Acc:HGNC:55641] | -2,64 |
| HISLA | [ENSG00000258867](https://www.ensembl.org/id/ENSG00000258867) | HIF1A stabilizing long noncoding RNA [Source:HGNC Symbol;Acc:HGNC:49467] | 6,57 |
| HLA-A | [ENSG00000206503](https://www.ensembl.org/id/ENSG00000206503) | major histocompatibility complex, class I, A [Source:HGNC Symbol;Acc:HGNC:4931] | -2,25 |
| HLA-DQA2 | [ENSG00000237541](https://www.ensembl.org/id/ENSG00000237541) | major histocompatibility complex, class II, DQ alpha 2 [Source:HGNC Symbol;Acc:HGNC:4943] | 4,88 |
| HLA-DRB1 | [ENSG00000196126](https://www.ensembl.org/id/ENSG00000196126) | major histocompatibility complex, class II, DR beta 1 [Source:HGNC Symbol;Acc:HGNC:4948] | -4,00 |
| HLA-DRB5 | [ENSG00000198502](https://www.ensembl.org/id/ENSG00000198502) | major histocompatibility complex, class II, DR beta 5 [Source:HGNC Symbol;Acc:HGNC:4953] | -9,68 |
| HLX-AS1 | [ENSG00000257551](https://www.ensembl.org/id/ENSG00000257551) | HLX antisense RNA 1 [Source:HGNC Symbol;Acc:HGNC:42509] | 3,04 |
| HMGCL | [ENSG00000117305](https://www.ensembl.org/id/ENSG00000117305) | 3-hydroxy-3-methylglutaryl-CoA lyase [Source:HGNC Symbol;Acc:HGNC:5005] | 1,00 |
| HOMER2 | [ENSG00000103942](https://www.ensembl.org/id/ENSG00000103942) | homer scaffold protein 2 [Source:HGNC Symbol;Acc:HGNC:17513] | 2,97 |
| HOMER3 | [ENSG00000051128](https://www.ensembl.org/id/ENSG00000051128) | homer scaffold protein 3 [Source:HGNC Symbol;Acc:HGNC:17514] | 1,60 |
| HOPX | [ENSG00000171476](https://www.ensembl.org/id/ENSG00000171476) | HOP homeobox [Source:HGNC Symbol;Acc:HGNC:24961] | 1,61 |
| HP | [ENSG00000257017](https://www.ensembl.org/id/ENSG00000257017) | haptoglobin [Source:HGNC Symbol;Acc:HGNC:5141] | -6,91 |
| HPN | [ENSG00000105707](https://www.ensembl.org/id/ENSG00000105707) | hepsin [Source:HGNC Symbol;Acc:HGNC:5155] | 2,24 |
| HRC | [ENSG00000130528](https://www.ensembl.org/id/ENSG00000130528) | histidine rich calcium binding protein [Source:HGNC Symbol;Acc:HGNC:5178] | 1,92 |
| HRG-AS1 | [ENSG00000197099](https://www.ensembl.org/id/ENSG00000197099) | HRG and FETUB antisense RNA 1 [Source:HGNC Symbol;Acc:HGNC:55915] | 5,83 |
| HRH1 | [ENSG00000196639](https://www.ensembl.org/id/ENSG00000196639) | histamine receptor H1 [Source:HGNC Symbol;Acc:HGNC:5182] | -2,15 |
| HS3ST5 | [ENSG00000249853](https://www.ensembl.org/id/ENSG00000249853) | heparan sulfate-glucosamine 3-sulfotransferase 5 [Source:HGNC Symbol;Acc:HGNC:19419] | 3,84 |
| HSD17B2 | [ENSG00000086696](https://www.ensembl.org/id/ENSG00000086696) | hydroxysteroid 17-beta dehydrogenase 2 [Source:HGNC Symbol;Acc:HGNC:5211] | 4,09 |
| HSD17B3 | [ENSG00000130948](https://www.ensembl.org/id/ENSG00000130948) | hydroxysteroid 17-beta dehydrogenase 3 [Source:HGNC Symbol;Acc:HGNC:5212] | -2,03 |
| HSD17B6 | [ENSG00000025423](https://www.ensembl.org/id/ENSG00000025423) | hydroxysteroid 17-beta dehydrogenase 6 [Source:HGNC Symbol;Acc:HGNC:23316] | -3,33 |
| HSDL2 | [ENSG00000119471](https://www.ensembl.org/id/ENSG00000119471) | hydroxysteroid dehydrogenase like 2 [Source:HGNC Symbol;Acc:HGNC:18572] | 1,56 |
| HSFX1 | [ENSG00000171116](https://www.ensembl.org/id/ENSG00000171116) | heat shock transcription factor family, X-linked 1 [Source:HGNC Symbol;Acc:HGNC:29603] | -6,48 |
| HTATSF1 | [ENSG00000102241](https://www.ensembl.org/id/ENSG00000102241) | HIV-1 Tat specific factor 1 [Source:HGNC Symbol;Acc:HGNC:5276] | 1,21 |
| HTD2 | [ENSG00000255154](https://www.ensembl.org/id/ENSG00000255154) | hydroxyacyl-thioester dehydratase type 2 [Source:HGNC Symbol;Acc:HGNC:53111] | 1,61 |
| HTN3 | [ENSG00000205649](https://www.ensembl.org/id/ENSG00000205649) | histatin 3 [Source:HGNC Symbol;Acc:HGNC:5284] | 7,52 |
| HTR6 | [ENSG00000158748](https://www.ensembl.org/id/ENSG00000158748) | 5-hydroxytryptamine receptor 6 [Source:HGNC Symbol;Acc:HGNC:5301] | -2,81 |
| IBA57 | [ENSG00000181873](https://www.ensembl.org/id/ENSG00000181873) | iron-sulfur cluster assembly factor IBA57 [Source:HGNC Symbol;Acc:HGNC:27302] | 1,28 |
| ICA1-AS1 | [ENSG00000244239](https://www.ensembl.org/id/ENSG00000244239) | ICA1 antisense RNA 1 [Source:HGNC Symbol;Acc:HGNC:55606] | 3,66 |
| ICOS | [ENSG00000163600](https://www.ensembl.org/id/ENSG00000163600) | inducible T cell costimulator [Source:HGNC Symbol;Acc:HGNC:5351] | 3,05 |
| IDH2 | [ENSG00000182054](https://www.ensembl.org/id/ENSG00000182054) | isocitrate dehydrogenase (NADP(+)) 2 [Source:HGNC Symbol;Acc:HGNC:5383] | 2,38 |
| IDI2-AS1 | [ENSG00000232656](https://www.ensembl.org/id/ENSG00000232656) | IDI2 antisense RNA 1 [Source:HGNC Symbol;Acc:HGNC:30885] | 2,37 |
| IDUA | [ENSG00000127415](https://www.ensembl.org/id/ENSG00000127415) | alpha-L-iduronidase [Source:HGNC Symbol;Acc:HGNC:5391] | -1,04 |
| IFI30 | [ENSG00000216490](https://www.ensembl.org/id/ENSG00000216490) | IFI30 lysosomal thiol reductase [Source:HGNC Symbol;Acc:HGNC:5398] | 1,87 |
| IGFL2-AS1 | [ENSG00000268621](https://www.ensembl.org/id/ENSG00000268621) | IGFL2 antisense RNA 1 [Source:HGNC Symbol;Acc:HGNC:52559] | 4,54 |
| IGSF1 | [ENSG00000147255](https://www.ensembl.org/id/ENSG00000147255) | immunoglobulin superfamily member 1 [Source:HGNC Symbol;Acc:HGNC:5948] | 2,69 |
| IGSF11 | [ENSG00000144847](https://www.ensembl.org/id/ENSG00000144847) | immunoglobulin superfamily member 11 [Source:HGNC Symbol;Acc:HGNC:16669] | 2,59 |
| IGSF21 | [ENSG00000117154](https://www.ensembl.org/id/ENSG00000117154) | immunoglobin superfamily member 21 [Source:HGNC Symbol;Acc:HGNC:28246] | 2,45 |
| IGSF9 | [ENSG00000085552](https://www.ensembl.org/id/ENSG00000085552) | immunoglobulin superfamily member 9 [Source:HGNC Symbol;Acc:HGNC:18132] | -5,58 |
| IL12RB2 | [ENSG00000081985](https://www.ensembl.org/id/ENSG00000081985) | interleukin 12 receptor subunit beta 2 [Source:HGNC Symbol;Acc:HGNC:5972] | 2,24 |
| IL17B | [ENSG00000127743](https://www.ensembl.org/id/ENSG00000127743) | interleukin 17B [Source:HGNC Symbol;Acc:HGNC:5982] | -1,47 |
| IL17D | [ENSG00000172458](https://www.ensembl.org/id/ENSG00000172458) | interleukin 17D [Source:HGNC Symbol;Acc:HGNC:5984] | 1,92 |
| IL1RAPL1 | [ENSG00000169306](https://www.ensembl.org/id/ENSG00000169306) | interleukin 1 receptor accessory protein like 1 [Source:HGNC Symbol;Acc:HGNC:5996] | 3,22 |
| IL1RL1 | [ENSG00000115602](https://www.ensembl.org/id/ENSG00000115602) | interleukin 1 receptor like 1 [Source:HGNC Symbol;Acc:HGNC:5998] | -3,77 |
| IL1RN | [ENSG00000136689](https://www.ensembl.org/id/ENSG00000136689) | interleukin 1 receptor antagonist [Source:HGNC Symbol;Acc:HGNC:6000] | 2,32 |
| IL20RA | [ENSG00000016402](https://www.ensembl.org/id/ENSG00000016402) | interleukin 20 receptor subunit alpha [Source:HGNC Symbol;Acc:HGNC:6003] | 1,74 |
| IL24 | [ENSG00000162892](https://www.ensembl.org/id/ENSG00000162892) | interleukin 24 [Source:HGNC Symbol;Acc:HGNC:11346] | 3,80 |
| IL7R | [ENSG00000168685](https://www.ensembl.org/id/ENSG00000168685) | interleukin 7 receptor [Source:HGNC Symbol;Acc:HGNC:6024] | 2,94 |
| INE2 | [ENSG00000281371](https://www.ensembl.org/id/ENSG00000281371) | inactivation escape 2 [Source:HGNC Symbol;Acc:HGNC:6061] | -1,97 |
| INHBB | [ENSG00000163083](https://www.ensembl.org/id/ENSG00000163083) | inhibin subunit beta B [Source:HGNC Symbol;Acc:HGNC:6067] | -1,55 |
| INKA2-AS1 | [ENSG00000227811](https://www.ensembl.org/id/ENSG00000227811) | INKA2 antisense RNA 1 [Source:HGNC Symbol;Acc:HGNC:49446] | 1,76 |
| IP6K3 | [ENSG00000161896](https://www.ensembl.org/id/ENSG00000161896) | inositol hexakisphosphate kinase 3 [Source:HGNC Symbol;Acc:HGNC:17269] | 2,34 |
| IPO13 | [ENSG00000117408](https://www.ensembl.org/id/ENSG00000117408) | importin 13 [Source:HGNC Symbol;Acc:HGNC:16853] | 1,13 |
| IQCJ-SCHIP1 | [ENSG00000283154](https://www.ensembl.org/id/ENSG00000283154) | IQCJ-SCHIP1 readthrough [Source:HGNC Symbol;Acc:HGNC:38842] | -1,66 |
| IRAG2 | [ENSG00000118308](https://www.ensembl.org/id/ENSG00000118308) | inositol 1,4,5-triphosphate receptor associated 2 [Source:HGNC Symbol;Acc:HGNC:6690] | 2,01 |
| IRX4 | [ENSG00000113430](https://www.ensembl.org/id/ENSG00000113430) | iroquois homeobox 4 [Source:HGNC Symbol;Acc:HGNC:6129] | 5,53 |
| IRX4-AS1 | [ENSG00000249116](https://www.ensembl.org/id/ENSG00000249116) | IRX4 antisense RNA 1 [Source:HGNC Symbol;Acc:HGNC:40305] | 5,95 |
| ISM2 | [ENSG00000100593](https://www.ensembl.org/id/ENSG00000100593) | isthmin 2 [Source:HGNC Symbol;Acc:HGNC:23176] | -5,15 |
| ITGA2B | [ENSG00000005961](https://www.ensembl.org/id/ENSG00000005961) | integrin subunit alpha 2b [Source:HGNC Symbol;Acc:HGNC:6138] | 3,12 |
| ITGA4 | [ENSG00000115232](https://www.ensembl.org/id/ENSG00000115232) | integrin subunit alpha 4 [Source:HGNC Symbol;Acc:HGNC:6140] | 2,38 |
| ITGAX | [ENSG00000140678](https://www.ensembl.org/id/ENSG00000140678) | integrin subunit alpha X [Source:HGNC Symbol;Acc:HGNC:6152] | 2,81 |
| ITGB2 | [ENSG00000160255](https://www.ensembl.org/id/ENSG00000160255) | integrin subunit beta 2 [Source:HGNC Symbol;Acc:HGNC:6155] | 2,32 |
| ITK | [ENSG00000113263](https://www.ensembl.org/id/ENSG00000113263) | IL2 inducible T cell kinase [Source:HGNC Symbol;Acc:HGNC:6171] | 2,57 |
| ITLN2 | [ENSG00000158764](https://www.ensembl.org/id/ENSG00000158764) | intelectin 2 [Source:HGNC Symbol;Acc:HGNC:20599] | -2,31 |
| IVD | [ENSG00000128928](https://www.ensembl.org/id/ENSG00000128928) | isovaleryl-CoA dehydrogenase [Source:HGNC Symbol;Acc:HGNC:6186] | 1,13 |
| IZUMO3 | [ENSG00000205442](https://www.ensembl.org/id/ENSG00000205442) | IZUMO family member 3 [Source:HGNC Symbol;Acc:HGNC:31421] | 6,54 |
| JAKMIP1 | [ENSG00000152969](https://www.ensembl.org/id/ENSG00000152969) | janus kinase and microtubule interacting protein 1 [Source:HGNC Symbol;Acc:HGNC:26460] | 3,36 |
| JAML | [ENSG00000160593](https://www.ensembl.org/id/ENSG00000160593) | junction adhesion molecule like [Source:HGNC Symbol;Acc:HGNC:19084] | 2,45 |
| KAT14 | [ENSG00000149474](https://www.ensembl.org/id/ENSG00000149474) | lysine acetyltransferase 14 [Source:HGNC Symbol;Acc:HGNC:15904] | 1,07 |
| KAZN | [ENSG00000189337](https://www.ensembl.org/id/ENSG00000189337) | kazrin, periplakin interacting protein [Source:HGNC Symbol;Acc:HGNC:29173] | -1,69 |
| KBTBD12 | [ENSG00000187715](https://www.ensembl.org/id/ENSG00000187715) | kelch repeat and BTB domain containing 12 [Source:HGNC Symbol;Acc:HGNC:25731] | 2,07 |
| KCNA3 | [ENSG00000177272](https://www.ensembl.org/id/ENSG00000177272) | potassium voltage-gated channel subfamily A member 3 [Source:HGNC Symbol;Acc:HGNC:6221] | 3,36 |
| KCNAB2 | [ENSG00000069424](https://www.ensembl.org/id/ENSG00000069424) | potassium voltage-gated channel subfamily A regulatory beta subunit 2 [Source:HGNC Symbol;Acc:HGNC:6229] | 1,50 |
| KCNF1 | [ENSG00000162975](https://www.ensembl.org/id/ENSG00000162975) | potassium voltage-gated channel modifier subfamily F member 1 [Source:HGNC Symbol;Acc:HGNC:6246] | 6,39 |
| KCNH5 | [ENSG00000140015](https://www.ensembl.org/id/ENSG00000140015) | potassium voltage-gated channel subfamily H member 5 [Source:HGNC Symbol;Acc:HGNC:6254] | -5,39 |
| KCNIP2 | [ENSG00000120049](https://www.ensembl.org/id/ENSG00000120049) | potassium voltage-gated channel interacting protein 2 [Source:HGNC Symbol;Acc:HGNC:15522] | -2,68 |
| KCNJ1 | [ENSG00000151704](https://www.ensembl.org/id/ENSG00000151704) | potassium inwardly rectifying channel subfamily J member 1 [Source:HGNC Symbol;Acc:HGNC:6255] | -3,35 |
| KCNJ11 | [ENSG00000187486](https://www.ensembl.org/id/ENSG00000187486) | potassium inwardly rectifying channel subfamily J member 11 [Source:HGNC Symbol;Acc:HGNC:6257] | 2,09 |
| KCNJ9 | [ENSG00000162728](https://www.ensembl.org/id/ENSG00000162728) | potassium inwardly rectifying channel subfamily J member 9 [Source:HGNC Symbol;Acc:HGNC:6270] | -2,38 |
| KCNK13 | [ENSG00000152315](https://www.ensembl.org/id/ENSG00000152315) | potassium two pore domain channel subfamily K member 13 [Source:HGNC Symbol;Acc:HGNC:6275] | 6,08 |
| KCNK15-AS1 | [ENSG00000244558](https://www.ensembl.org/id/ENSG00000244558) | KCNK15 and WISP2 antisense RNA 1 [Source:HGNC Symbol;Acc:HGNC:49901] | 2,55 |
| KDM4E | [ENSG00000235268](https://www.ensembl.org/id/ENSG00000235268) | lysine demethylase 4E [Source:HGNC Symbol;Acc:HGNC:37098] | 6,14 |
| KHDC1L | [ENSG00000256980](https://www.ensembl.org/id/ENSG00000256980) | KH domain containing 1 like [Source:HGNC Symbol;Acc:HGNC:37274] | -5,24 |
| KHDRBS2 | [ENSG00000112232](https://www.ensembl.org/id/ENSG00000112232) | KH RNA binding domain containing, signal transduction associated 2 [Source:HGNC Symbol;Acc:HGNC:18114] | 3,92 |
| KIAA0040 | [ENSG00000235750](https://www.ensembl.org/id/ENSG00000235750) | KIAA0040 [Source:HGNC Symbol;Acc:HGNC:28950] | -1,32 |
| KIAA1217 | [ENSG00000120549](https://www.ensembl.org/id/ENSG00000120549) | KIAA1217 [Source:HGNC Symbol;Acc:HGNC:25428] | 1,41 |
| KIF20A | [ENSG00000112984](https://www.ensembl.org/id/ENSG00000112984) | kinesin family member 20A [Source:HGNC Symbol;Acc:HGNC:9787] | -3,83 |
| KLB | [ENSG00000134962](https://www.ensembl.org/id/ENSG00000134962) | klotho beta [Source:HGNC Symbol;Acc:HGNC:15527] | -2,73 |
| KLF15 | [ENSG00000163884](https://www.ensembl.org/id/ENSG00000163884) | KLF transcription factor 15 [Source:HGNC Symbol;Acc:HGNC:14536] | 1,06 |
| KLHDC7B | [ENSG00000130487](https://www.ensembl.org/id/ENSG00000130487) | kelch domain containing 7B [Source:HGNC Symbol;Acc:HGNC:25145] | 3,41 |
| KLHDC8B | [ENSG00000185909](https://www.ensembl.org/id/ENSG00000185909) | kelch domain containing 8B [Source:HGNC Symbol;Acc:HGNC:28557] | 1,04 |
| KLHL33 | [ENSG00000185271](https://www.ensembl.org/id/ENSG00000185271) | kelch like family member 33 [Source:HGNC Symbol;Acc:HGNC:31952] | 1,94 |
| KLHL34 | [ENSG00000185915](https://www.ensembl.org/id/ENSG00000185915) | kelch like family member 34 [Source:HGNC Symbol;Acc:HGNC:26634] | 3,32 |
| KLHL38 | [ENSG00000175946](https://www.ensembl.org/id/ENSG00000175946) | kelch like family member 38 [Source:HGNC Symbol;Acc:HGNC:34435] | 2,11 |
| KLHL40 | [ENSG00000157119](https://www.ensembl.org/id/ENSG00000157119) | kelch like family member 40 [Source:HGNC Symbol;Acc:HGNC:30372] | 3,34 |
| KLK10 | [ENSG00000129451](https://www.ensembl.org/id/ENSG00000129451) | kallikrein related peptidase 10 [Source:HGNC Symbol;Acc:HGNC:6358] | -3,85 |
| KLK11 | [ENSG00000167757](https://www.ensembl.org/id/ENSG00000167757) | kallikrein related peptidase 11 [Source:HGNC Symbol;Acc:HGNC:6359] | -6,42 |
| KLK8 | [ENSG00000129455](https://www.ensembl.org/id/ENSG00000129455) | kallikrein related peptidase 8 [Source:HGNC Symbol;Acc:HGNC:6369] | -4,24 |
| KPNA2 | [ENSG00000182481](https://www.ensembl.org/id/ENSG00000182481) | karyopherin subunit alpha 2 [Source:HGNC Symbol;Acc:HGNC:6395] | -1,53 |
| KPNA3 | [ENSG00000102753](https://www.ensembl.org/id/ENSG00000102753) | karyopherin subunit alpha 3 [Source:HGNC Symbol;Acc:HGNC:6396] | 1,13 |
| KREMEN1 | [ENSG00000183762](https://www.ensembl.org/id/ENSG00000183762) | kringle containing transmembrane protein 1 [Source:HGNC Symbol;Acc:HGNC:17550] | 1,42 |
| KRT1 | [ENSG00000167768](https://www.ensembl.org/id/ENSG00000167768) | keratin 1 [Source:HGNC Symbol;Acc:HGNC:6412] | 4,57 |
| KRT18 | [ENSG00000111057](https://www.ensembl.org/id/ENSG00000111057) | keratin 18 [Source:HGNC Symbol;Acc:HGNC:6430] | -2,68 |
| KRT19 | [ENSG00000171345](https://www.ensembl.org/id/ENSG00000171345) | keratin 19 [Source:HGNC Symbol;Acc:HGNC:6436] | -2,46 |
| KRT23 | [ENSG00000108244](https://www.ensembl.org/id/ENSG00000108244) | keratin 23 [Source:HGNC Symbol;Acc:HGNC:6438] | 7,19 |
| KRT31 | [ENSG00000094796](https://www.ensembl.org/id/ENSG00000094796) | keratin 31 [Source:HGNC Symbol;Acc:HGNC:6448] | 4,49 |
| KRT32 | [ENSG00000108759](https://www.ensembl.org/id/ENSG00000108759) | keratin 32 [Source:HGNC Symbol;Acc:HGNC:6449] | 4,77 |
| KRT5 | [ENSG00000186081](https://www.ensembl.org/id/ENSG00000186081) | keratin 5 [Source:HGNC Symbol;Acc:HGNC:6442] | -5,43 |
| KRT8 | [ENSG00000170421](https://www.ensembl.org/id/ENSG00000170421) | keratin 8 [Source:HGNC Symbol;Acc:HGNC:6446] | -2,77 |
| KRTAP27-1 | [ENSG00000206107](https://www.ensembl.org/id/ENSG00000206107) | keratin associated protein 27-1 [Source:HGNC Symbol;Acc:HGNC:33864] | 6,50 |
| KY | [ENSG00000174611](https://www.ensembl.org/id/ENSG00000174611) | kyphoscoliosis peptidase [Source:HGNC Symbol;Acc:HGNC:26576] | 1,61 |
| L2HGDH | [ENSG00000087299](https://www.ensembl.org/id/ENSG00000087299) | L-2-hydroxyglutarate dehydrogenase [Source:HGNC Symbol;Acc:HGNC:20499] | 1,43 |
| LACTB2 | [ENSG00000147592](https://www.ensembl.org/id/ENSG00000147592) | lactamase beta 2 [Source:HGNC Symbol;Acc:HGNC:18512] | 1,48 |
| LAMA1 | [ENSG00000101680](https://www.ensembl.org/id/ENSG00000101680) | laminin subunit alpha 1 [Source:HGNC Symbol;Acc:HGNC:6481] | -3,13 |
| LAMA4 | [ENSG00000112769](https://www.ensembl.org/id/ENSG00000112769) | laminin subunit alpha 4 [Source:HGNC Symbol;Acc:HGNC:6484] | -1,17 |
| LAPTM4B | [ENSG00000104341](https://www.ensembl.org/id/ENSG00000104341) | lysosomal protein transmembrane 4 beta [Source:HGNC Symbol;Acc:HGNC:13646] | 1,69 |
| LBP | [ENSG00000129988](https://www.ensembl.org/id/ENSG00000129988) | lipopolysaccharide binding protein [Source:HGNC Symbol;Acc:HGNC:6517] | 3,07 |
| LCK | [ENSG00000182866](https://www.ensembl.org/id/ENSG00000182866) | LCK proto-oncogene, Src family tyrosine kinase [Source:HGNC Symbol;Acc:HGNC:6524] | 2,83 |
| LCN10 | [ENSG00000187922](https://www.ensembl.org/id/ENSG00000187922) | lipocalin 10 [Source:HGNC Symbol;Acc:HGNC:20892] | -2,32 |
| LCN2 | [ENSG00000148346](https://www.ensembl.org/id/ENSG00000148346) | lipocalin 2 [Source:HGNC Symbol;Acc:HGNC:6526] | 2,92 |
| LCP1 | [ENSG00000136167](https://www.ensembl.org/id/ENSG00000136167) | lymphocyte cytosolic protein 1 [Source:HGNC Symbol;Acc:HGNC:6528] | 2,48 |
| LDAF1 | [ENSG00000011638](https://www.ensembl.org/id/ENSG00000011638) | lipid droplet assembly factor 1 [Source:HGNC Symbol;Acc:HGNC:30136] | 1,17 |
| LDB3 | [ENSG00000122367](https://www.ensembl.org/id/ENSG00000122367) | LIM domain binding 3 [Source:HGNC Symbol;Acc:HGNC:15710] | 2,06 |
| LDHB | [ENSG00000111716](https://www.ensembl.org/id/ENSG00000111716) | lactate dehydrogenase B [Source:HGNC Symbol;Acc:HGNC:6541] | 1,66 |
| LDHC | [ENSG00000166796](https://www.ensembl.org/id/ENSG00000166796) | lactate dehydrogenase C [Source:HGNC Symbol;Acc:HGNC:6544] | 3,81 |
| LDHD | [ENSG00000166816](https://www.ensembl.org/id/ENSG00000166816) | lactate dehydrogenase D [Source:HGNC Symbol;Acc:HGNC:19708] | 2,36 |
| LDLR | [ENSG00000130164](https://www.ensembl.org/id/ENSG00000130164) | low density lipoprotein receptor [Source:HGNC Symbol;Acc:HGNC:6547] | -1,87 |
| LDLRAD2 | [ENSG00000187942](https://www.ensembl.org/id/ENSG00000187942) | low density lipoprotein receptor class A domain containing 2 [Source:HGNC Symbol;Acc:HGNC:32071] | -1,37 |
| LEFTY1 | [ENSG00000243709](https://www.ensembl.org/id/ENSG00000243709) | left-right determination factor 1 [Source:HGNC Symbol;Acc:HGNC:6552] | -2,15 |
| LEP | [ENSG00000174697](https://www.ensembl.org/id/ENSG00000174697) | leptin [Source:HGNC Symbol;Acc:HGNC:6553] | -1,86 |
| LGALS12 | [ENSG00000133317](https://www.ensembl.org/id/ENSG00000133317) | galectin 12 [Source:HGNC Symbol;Acc:HGNC:15788] | -2,96 |
| LGALS7B | [ENSG00000178934](https://www.ensembl.org/id/ENSG00000178934) | galectin 7B [Source:HGNC Symbol;Acc:HGNC:34447] | 5,38 |
| LGI1 | [ENSG00000108231](https://www.ensembl.org/id/ENSG00000108231) | leucine rich glioma inactivated 1 [Source:HGNC Symbol;Acc:HGNC:6572] | 1,82 |
| LGR5 | [ENSG00000139292](https://www.ensembl.org/id/ENSG00000139292) | leucine rich repeat containing G protein-coupled receptor 5 [Source:HGNC Symbol;Acc:HGNC:4504] | 2,65 |
| LHFPL5 | [ENSG00000197753](https://www.ensembl.org/id/ENSG00000197753) | LHFPL tetraspan subfamily member 5 [Source:HGNC Symbol;Acc:HGNC:21253] | -2,17 |
| LHX1-DT | [ENSG00000277268](https://www.ensembl.org/id/ENSG00000277268) | LHX1 divergent transcript [Source:HGNC Symbol;Acc:HGNC:53778] | 5,44 |
| LHX3 | [ENSG00000107187](https://www.ensembl.org/id/ENSG00000107187) | LIM homeobox 3 [Source:HGNC Symbol;Acc:HGNC:6595] | 5,65 |
| LIAS | [ENSG00000121897](https://www.ensembl.org/id/ENSG00000121897) | lipoic acid synthetase [Source:HGNC Symbol;Acc:HGNC:16429] | 1,11 |
| LIF | [ENSG00000128342](https://www.ensembl.org/id/ENSG00000128342) | LIF interleukin 6 family cytokine [Source:HGNC Symbol;Acc:HGNC:6596] | -2,80 |
| LILRA1 | [ENSG00000104974](https://www.ensembl.org/id/ENSG00000104974) | leukocyte immunoglobulin like receptor A1 [Source:HGNC Symbol;Acc:HGNC:6602] | 4,82 |
| LILRA2 | [ENSG00000239998](https://www.ensembl.org/id/ENSG00000239998) | leukocyte immunoglobulin like receptor A2 [Source:HGNC Symbol;Acc:HGNC:6603] | 2,81 |
| LILRA5 | [ENSG00000187116](https://www.ensembl.org/id/ENSG00000187116) | leukocyte immunoglobulin like receptor A5 [Source:HGNC Symbol;Acc:HGNC:16309] | 4,05 |
| LINC00165 | [ENSG00000261706](https://www.ensembl.org/id/ENSG00000261706) | long intergenic non-protein coding RNA 165 [Source:HGNC Symbol;Acc:HGNC:33166] | -3,41 |
| LINC00216 | [ENSG00000279636](https://www.ensembl.org/id/ENSG00000279636) | long intergenic non-protein coding RNA 216 [Source:NCBI gene (formerly Entrezgene);Acc:55451] | -2,78 |
| LINC00339 | [ENSG00000218510](https://www.ensembl.org/id/ENSG00000218510) | long intergenic non-protein coding RNA 339 [Source:HGNC Symbol;Acc:HGNC:25011] | 1,95 |
| LINC00343 | [ENSG00000226620](https://www.ensembl.org/id/ENSG00000226620) | long intergenic non-protein coding RNA 343 [Source:HGNC Symbol;Acc:HGNC:42500] | 3,94 |
| LINC00402 | [ENSG00000235532](https://www.ensembl.org/id/ENSG00000235532) | long intergenic non-protein coding RNA 402 [Source:HGNC Symbol;Acc:HGNC:42732] | 6,75 |
| LINC00486_1 | [ENSG00000230876](https://www.ensembl.org/id/ENSG00000230876) | long intergenic non-protein coding RNA 486 [Source:HGNC Symbol;Acc:HGNC:42946] | 4,73 |
| LINC00501 | [ENSG00000203645](https://www.ensembl.org/id/ENSG00000203645) | long intergenic non-protein coding RNA 501 [Source:HGNC Symbol;Acc:HGNC:43439] | 5,25 |
| LINC00528 | [ENSG00000269220](https://www.ensembl.org/id/ENSG00000269220) | long intergenic non-protein coding RNA 528 [Source:HGNC Symbol;Acc:HGNC:26875] | -3,03 |
| LINC00545 | [ENSG00000236094](https://www.ensembl.org/id/ENSG00000236094) | long intergenic non-protein coding RNA 545 [Source:HGNC Symbol;Acc:HGNC:43680] | 5,06 |
| LINC00581 | [ENSG00000280989](https://www.ensembl.org/id/ENSG00000280989) | long intergenic non-protein coding RNA 581 [Source:HGNC Symbol;Acc:HGNC:43840] | 4,05 |
| LINC00652 | [ENSG00000179935](https://www.ensembl.org/id/ENSG00000179935) | long intergenic non-protein coding RNA 652 [Source:HGNC Symbol;Acc:HGNC:25003] | 2,91 |
| LINC00661 | [ENSG00000205396](https://www.ensembl.org/id/ENSG00000205396) | long intergenic non-protein coding RNA 661 [Source:HGNC Symbol;Acc:HGNC:27002] | 2,62 |
| LINC00944 | [ENSG00000256128](https://www.ensembl.org/id/ENSG00000256128) | long intergenic non-protein coding RNA 944 [Source:HGNC Symbol;Acc:HGNC:48640] | 4,14 |
| LINC00954 | [ENSG00000228784](https://www.ensembl.org/id/ENSG00000228784) | long intergenic non-protein coding RNA 954 [Source:HGNC Symbol;Acc:HGNC:48668] | -2,30 |
| LINC01036 | [ENSG00000230426](https://www.ensembl.org/id/ENSG00000230426) | long intergenic non-protein coding RNA 1036 [Source:HGNC Symbol;Acc:HGNC:49024] | 4,91 |
| LINC01063 | [ENSG00000232065](https://www.ensembl.org/id/ENSG00000232065) | long intergenic non-protein coding RNA 1063 [Source:HGNC Symbol;Acc:HGNC:49092] | 6,18 |
| LINC01070 | [ENSG00000260102](https://www.ensembl.org/id/ENSG00000260102) | long intergenic non-protein coding RNA 1070 [Source:HGNC Symbol;Acc:HGNC:49110] | -5,17 |
| LINC01091 | [ENSG00000249464](https://www.ensembl.org/id/ENSG00000249464) | long intergenic non-protein coding RNA 1091 [Source:HGNC Symbol;Acc:HGNC:27721] | 2,14 |
| LINC01152 | [ENSG00000256124](https://www.ensembl.org/id/ENSG00000256124) | long intergenic non-protein coding RNA 1152 [Source:HGNC Symbol;Acc:HGNC:16752] | 5,55 |
| LINC01176 | [ENSG00000281404](https://www.ensembl.org/id/ENSG00000281404) | long intergenic non-protein coding RNA 1176 [Source:HGNC Symbol;Acc:HGNC:49548] | -2,14 |
| LINC01198 | [ENSG00000231817](https://www.ensembl.org/id/ENSG00000231817) | long intergenic non-protein coding RNA 1198 [Source:HGNC Symbol;Acc:HGNC:49598] | -3,33 |
| LINC01215 | [ENSG00000271856](https://www.ensembl.org/id/ENSG00000271856) | long intergenic non-protein coding RNA 1215 [Source:HGNC Symbol;Acc:HGNC:49651] | 4,33 |
| LINC01229 | [ENSG00000260876](https://www.ensembl.org/id/ENSG00000260876) | long intergenic non-protein coding RNA 1229 [Source:HGNC Symbol;Acc:HGNC:49682] | -2,54 |
| LINC01230 | [ENSG00000281769](https://www.ensembl.org/id/ENSG00000281769) | long intergenic non-protein coding RNA 1230 [Source:HGNC Symbol;Acc:HGNC:49686] | -3,41 |
| LINC01273 | [ENSG00000231742](https://www.ensembl.org/id/ENSG00000231742) | long intergenic non-protein coding RNA 1273 [Source:HGNC Symbol;Acc:HGNC:50329] | 1,91 |
| LINC01289 | [ENSG00000253734](https://www.ensembl.org/id/ENSG00000253734) | long intergenic non-protein coding RNA 1289 [Source:HGNC Symbol;Acc:HGNC:50354] | 5,44 |
| LINC01303 | [ENSG00000250548](https://www.ensembl.org/id/ENSG00000250548) | long intergenic non-protein coding RNA 1303 [Source:HGNC Symbol;Acc:HGNC:50470] | 2,61 |
| LINC01304 | [ENSG00000237401](https://www.ensembl.org/id/ENSG00000237401) | long intergenic non-protein coding RNA 1304 [Source:HGNC Symbol;Acc:HGNC:50472] | 5,83 |
| LINC01366 | [ENSG00000235172](https://www.ensembl.org/id/ENSG00000235172) | long intergenic non-protein coding RNA 1366 [Source:HGNC Symbol;Acc:HGNC:27416] | 3,51 |
| LINC01405 | [ENSG00000185847](https://www.ensembl.org/id/ENSG00000185847) | long intergenic non-protein coding RNA 1405 [Source:HGNC Symbol;Acc:HGNC:50688] | 3,10 |
| LINC01484 | [ENSG00000253686](https://www.ensembl.org/id/ENSG00000253686) | long intergenic non-protein coding RNA 1484 [Source:HGNC Symbol;Acc:HGNC:51136] | -6,28 |
| LINC01635 | [ENSG00000228397](https://www.ensembl.org/id/ENSG00000228397) | long intergenic non-protein coding RNA 1635 [Source:HGNC Symbol;Acc:HGNC:52422] | 5,82 |
| LINC01687 | [ENSG00000233215](https://www.ensembl.org/id/ENSG00000233215) | long intergenic non-protein coding RNA 1687 [Source:HGNC Symbol;Acc:HGNC:52474] | 5,99 |
| LINC01762 | [ENSG00000233154](https://www.ensembl.org/id/ENSG00000233154) | long intergenic non-protein coding RNA 1762 [Source:HGNC Symbol;Acc:HGNC:52552] | 3,82 |
| LINC01779 | [ENSG00000235933](https://www.ensembl.org/id/ENSG00000235933) | long intergenic non-protein coding RNA 1779 [Source:HGNC Symbol;Acc:HGNC:52569] | 3,37 |
| LINC01828 | [ENSG00000235885](https://www.ensembl.org/id/ENSG00000235885) | long intergenic non-protein coding RNA 1828 [Source:HGNC Symbol;Acc:HGNC:52634] | -5,04 |
| LINC01854 | [ENSG00000204460](https://www.ensembl.org/id/ENSG00000204460) | long intergenic non-protein coding RNA 1854 [Source:HGNC Symbol;Acc:HGNC:52670] | 2,96 |
| LINC01871 | [ENSG00000235576](https://www.ensembl.org/id/ENSG00000235576) | long intergenic non-protein coding RNA 1871 [Source:HGNC Symbol;Acc:HGNC:52690] | 4,11 |
| LINC01954 | [ENSG00000271952](https://www.ensembl.org/id/ENSG00000271952) | long intergenic non-protein coding RNA 1954 [Source:HGNC Symbol;Acc:HGNC:52779] | 3,47 |
| LINC01963 | [ENSG00000260804](https://www.ensembl.org/id/ENSG00000260804) | long intergenic non-protein coding RNA 1963 [Source:HGNC Symbol;Acc:HGNC:25283] | 1,40 |
| LINC01973 | [ENSG00000204283](https://www.ensembl.org/id/ENSG00000204283) | long intergenic non-protein coding RNA 1973 [Source:HGNC Symbol;Acc:HGNC:52800] | 4,67 |
| LINC02004 | [ENSG00000240006](https://www.ensembl.org/id/ENSG00000240006) | long intergenic non-protein coding RNA 2004 [Source:HGNC Symbol;Acc:HGNC:52838] | -5,31 |
| LINC02006 | [ENSG00000238755](https://www.ensembl.org/id/ENSG00000238755) | long intergenic non-protein coding RNA 2006 [Source:HGNC Symbol;Acc:HGNC:52842] | -5,49 |
| LINC02012 | [ENSG00000272989](https://www.ensembl.org/id/ENSG00000272989) | long intergenic non-protein coding RNA 2012 [Source:HGNC Symbol;Acc:HGNC:52847] | 5,31 |
| LINC02082 | [ENSG00000242268](https://www.ensembl.org/id/ENSG00000242268) | long intergenic non-protein coding RNA 2082 [Source:HGNC Symbol;Acc:HGNC:52931] | -3,98 |
| LINC02145 | [ENSG00000250490](https://www.ensembl.org/id/ENSG00000250490) | long intergenic non-protein coding RNA 2145 [Source:HGNC Symbol;Acc:HGNC:53005] | -2,93 |
| LINC02166 | [ENSG00000260259](https://www.ensembl.org/id/ENSG00000260259) | long intergenic non-protein coding RNA 2166 [Source:HGNC Symbol;Acc:HGNC:53027] | 5,65 |
| LINC02273 | [ENSG00000245954](https://www.ensembl.org/id/ENSG00000245954) | long intergenic non-protein coding RNA 2273 [Source:HGNC Symbol;Acc:HGNC:53188] | 3,57 |
| LINC02301 | [ENSG00000258743](https://www.ensembl.org/id/ENSG00000258743) | long intergenic non-protein coding RNA 2301 [Source:HGNC Symbol;Acc:HGNC:53220] | 5,83 |
| LINC02363 | [ENSG00000180712](https://www.ensembl.org/id/ENSG00000180712) | long intergenic non-protein coding RNA 2363 [Source:HGNC Symbol;Acc:HGNC:53286] | 4,72 |
| LINC02384 | [ENSG00000251301](https://www.ensembl.org/id/ENSG00000251301) | long intergenic non-protein coding RNA 2384 [Source:HGNC Symbol;Acc:HGNC:53308] | -2,34 |
| LINC02388 | [ENSG00000257259](https://www.ensembl.org/id/ENSG00000257259) | long intergenic non-protein coding RNA 2388 [Source:HGNC Symbol;Acc:HGNC:53315] | 3,47 |
| LINC02405 | [ENSG00000249345](https://www.ensembl.org/id/ENSG00000249345) | long intergenic non-protein coding RNA 2405 [Source:HGNC Symbol;Acc:HGNC:53333] | -3,32 |
| LINC02432 | [ENSG00000248810](https://www.ensembl.org/id/ENSG00000248810) | long intergenic non-protein coding RNA 2432 [Source:HGNC Symbol;Acc:HGNC:53363] | 4,23 |
| LINC02446 | [ENSG00000256039](https://www.ensembl.org/id/ENSG00000256039) | long intergenic non-protein coding RNA 2446 [Source:HGNC Symbol;Acc:HGNC:53378] | 5,11 |
| LINC02457 | [ENSG00000258018](https://www.ensembl.org/id/ENSG00000258018) | long intergenic non-protein coding RNA 2457 [Source:HGNC Symbol;Acc:HGNC:53393] | 4,18 |
| LINC02478 | [ENSG00000285373](https://www.ensembl.org/id/ENSG00000285373) | long intergenic non-protein coding RNA 2478 [Source:HGNC Symbol;Acc:HGNC:53446] | 2,46 |
| LINC02502 | [ENSG00000250392](https://www.ensembl.org/id/ENSG00000250392) | long intergenic non-protein coding RNA 2502 [Source:HGNC Symbol;Acc:HGNC:53491] | 4,51 |
| LINC02515 | [ENSG00000250620](https://www.ensembl.org/id/ENSG00000250620) | long intergenic non-protein coding RNA 2515 [Source:HGNC Symbol;Acc:HGNC:53504] | -2,26 |
| LINC02574 | [ENSG00000233975](https://www.ensembl.org/id/ENSG00000233975) | long intergenic non-protein coding RNA 2574 [Source:HGNC Symbol;Acc:HGNC:53746] | 2,83 |
| LINC02669 | [ENSG00000233321](https://www.ensembl.org/id/ENSG00000233321) | long intergenic non-protein coding RNA 2669 [Source:HGNC Symbol;Acc:HGNC:54155] | -2,68 |
| LINC02688 | [ENSG00000254872](https://www.ensembl.org/id/ENSG00000254872) | long intergenic non-protein coding RNA 2688 [Source:HGNC Symbol;Acc:HGNC:54184] | -3,28 |
| LINC02718 | [ENSG00000255418](https://www.ensembl.org/id/ENSG00000255418) | long intergenic non-protein coding RNA 2718 [Source:HGNC Symbol;Acc:HGNC:54235] | -4,29 |
| LINC02783 | [ENSG00000204362](https://www.ensembl.org/id/ENSG00000204362) | long intergenic non-protein coding RNA 2783 [Source:HGNC Symbol;Acc:HGNC:54303] | 7,03 |
| LINC02926 | [ENSG00000230310](https://www.ensembl.org/id/ENSG00000230310) | long intergenic non-protein coding RNA 2926 [Source:HGNC Symbol;Acc:HGNC:55776] | 6,08 |
| LINC03020 | [ENSG00000253967](https://www.ensembl.org/id/ENSG00000253967) | long intergenic non-protein coding RNA 3020 [Source:HGNC Symbol;Acc:HGNC:56148] | 6,35 |
| LINCADL | [ENSG00000287148](https://www.ensembl.org/id/ENSG00000287148) | lincRNA adipogenesis and lipogenesis associated [Source:HGNC Symbol;Acc:HGNC:53956] | -2,87 |
| LIPE | [ENSG00000079435](https://www.ensembl.org/id/ENSG00000079435) | lipase E, hormone sensitive type [Source:HGNC Symbol;Acc:HGNC:6621] | -2,90 |
| LIPJ | [ENSG00000204022](https://www.ensembl.org/id/ENSG00000204022) | lipase family member J [Source:HGNC Symbol;Acc:HGNC:21773] | 5,93 |
| LIPN | [ENSG00000204020](https://www.ensembl.org/id/ENSG00000204020) | lipase family member N [Source:HGNC Symbol;Acc:HGNC:23452] | 4,90 |
| LMOD3 | [ENSG00000163380](https://www.ensembl.org/id/ENSG00000163380) | leiomodin 3 [Source:HGNC Symbol;Acc:HGNC:6649] | 2,47 |
| LNCOG | [ENSG00000257219](https://www.ensembl.org/id/ENSG00000257219) | lncRNA osteogenesis associated [Source:HGNC Symbol;Acc:HGNC:53336] | 3,80 |
| LOXL1 | [ENSG00000129038](https://www.ensembl.org/id/ENSG00000129038) | lysyl oxidase like 1 [Source:HGNC Symbol;Acc:HGNC:6665] | -1,07 |
| LPAR1 | [ENSG00000198121](https://www.ensembl.org/id/ENSG00000198121) | lysophosphatidic acid receptor 1 [Source:HGNC Symbol;Acc:HGNC:3166] | -0,99 |
| LPIN3 | [ENSG00000132793](https://www.ensembl.org/id/ENSG00000132793) | lipin 3 [Source:HGNC Symbol;Acc:HGNC:14451] | -1,10 |
| LRP1B | [ENSG00000168702](https://www.ensembl.org/id/ENSG00000168702) | LDL receptor related protein 1B [Source:HGNC Symbol;Acc:HGNC:6693] | 2,62 |
| LRP2 | [ENSG00000081479](https://www.ensembl.org/id/ENSG00000081479) | LDL receptor related protein 2 [Source:HGNC Symbol;Acc:HGNC:6694] | -8,81 |
| LRRC14B | [ENSG00000185028](https://www.ensembl.org/id/ENSG00000185028) | leucine rich repeat containing 14B [Source:HGNC Symbol;Acc:HGNC:37268] | 3,64 |
| LRRC20 | [ENSG00000172731](https://www.ensembl.org/id/ENSG00000172731) | leucine rich repeat containing 20 [Source:HGNC Symbol;Acc:HGNC:23421] | 2,00 |
| LRRC25 | [ENSG00000175489](https://www.ensembl.org/id/ENSG00000175489) | leucine rich repeat containing 25 [Source:HGNC Symbol;Acc:HGNC:29806] | 2,80 |
| LRRC32 | [ENSG00000137507](https://www.ensembl.org/id/ENSG00000137507) | leucine rich repeat containing 32 [Source:HGNC Symbol;Acc:HGNC:4161] | -1,32 |
| LRRC52 | [ENSG00000162763](https://www.ensembl.org/id/ENSG00000162763) | leucine rich repeat containing 52 [Source:HGNC Symbol;Acc:HGNC:32156] | 3,10 |
| LRRC8E | [ENSG00000171017](https://www.ensembl.org/id/ENSG00000171017) | leucine rich repeat containing 8 VRAC subunit E [Source:HGNC Symbol;Acc:HGNC:26272] | 3,00 |
| LRRN4 | [ENSG00000125872](https://www.ensembl.org/id/ENSG00000125872) | leucine rich repeat neuronal 4 [Source:HGNC Symbol;Acc:HGNC:16208] | -4,60 |
| LSAMP-AS1 | [ENSG00000240922](https://www.ensembl.org/id/ENSG00000240922) | LSAMP antisense RNA 1 [Source:HGNC Symbol;Acc:HGNC:40350] | -4,97 |
| LSMEM2 | [ENSG00000179564](https://www.ensembl.org/id/ENSG00000179564) | leucine rich single-pass membrane protein 2 [Source:HGNC Symbol;Acc:HGNC:26781] | 1,96 |
| LST1 | [ENSG00000204482](https://www.ensembl.org/id/ENSG00000204482) | leukocyte specific transcript 1 [Source:HGNC Symbol;Acc:HGNC:14189] | 2,26 |
| LTB | [ENSG00000227507](https://www.ensembl.org/id/ENSG00000227507) | lymphotoxin beta [Source:HGNC Symbol;Acc:HGNC:6711] | 2,49 |
| LVRN | [ENSG00000172901](https://www.ensembl.org/id/ENSG00000172901) | laeverin [Source:HGNC Symbol;Acc:HGNC:26904] | -1,78 |
| LY6G6F | [ENSG00000204424](https://www.ensembl.org/id/ENSG00000204424) | lymphocyte antigen 6 family member G6F [Source:HGNC Symbol;Acc:HGNC:13933] | 7,44 |
| LY75-CD302 | [ENSG00000248672](https://www.ensembl.org/id/ENSG00000248672) | LY75-CD302 readthrough [Source:HGNC Symbol;Acc:HGNC:38828] | 6,11 |
| LYPD1 | [ENSG00000150551](https://www.ensembl.org/id/ENSG00000150551) | LY6/PLAUR domain containing 1 [Source:HGNC Symbol;Acc:HGNC:28431] | 2,91 |
| LYRM7 | [ENSG00000186687](https://www.ensembl.org/id/ENSG00000186687) | LYR motif containing 7 [Source:HGNC Symbol;Acc:HGNC:28072] | 1,17 |
| LYZ | [ENSG00000090382](https://www.ensembl.org/id/ENSG00000090382) | lysozyme [Source:HGNC Symbol;Acc:HGNC:6740] | 2,55 |
| MACORIS | [ENSG00000237797](https://www.ensembl.org/id/ENSG00000237797) | macrophage enriched lincRNA repressor of IFN-gamma signaling [Source:HGNC Symbol;Acc:HGNC:53963] | 5,17 |
| MACROD1 | [ENSG00000133315](https://www.ensembl.org/id/ENSG00000133315) | mono-ADP ribosylhydrolase 1 [Source:HGNC Symbol;Acc:HGNC:29598] | 1,72 |
| MAGEA8-AS1 | [ENSG00000230899](https://www.ensembl.org/id/ENSG00000230899) | MAGEA8 antisense RNA 1 [Source:HGNC Symbol;Acc:HGNC:45093] | 5,99 |
| MAGEC3 | [ENSG00000165509](https://www.ensembl.org/id/ENSG00000165509) | MAGE family member C3 [Source:HGNC Symbol;Acc:HGNC:23798] | 2,46 |
| MAGED4B | [ENSG00000187243](https://www.ensembl.org/id/ENSG00000187243) | MAGE family member D4B [Source:HGNC Symbol;Acc:HGNC:22880] | -2,12 |
| MAL2 | [ENSG00000147676](https://www.ensembl.org/id/ENSG00000147676) | mal, T cell differentiation protein 2 [Source:HGNC Symbol;Acc:HGNC:13634] | -3,91 |
| MALL | [ENSG00000144063](https://www.ensembl.org/id/ENSG00000144063) | mal, T cell differentiation protein like [Source:HGNC Symbol;Acc:HGNC:6818] | -1,16 |
| MAMSTR | [ENSG00000176909](https://www.ensembl.org/id/ENSG00000176909) | MEF2 activating motif and SAP domain containing transcriptional regulator [Source:HGNC Symbol;Acc:HGNC:26689] | 1,72 |
| MANSC4 | [ENSG00000205693](https://www.ensembl.org/id/ENSG00000205693) | MANSC domain containing 4 [Source:HGNC Symbol;Acc:HGNC:40023] | -5,24 |
| MAP2K1 | [ENSG00000169032](https://www.ensembl.org/id/ENSG00000169032) | mitogen-activated protein kinase kinase 1 [Source:HGNC Symbol;Acc:HGNC:6840] | 1,08 |
| MAP2K5 | [ENSG00000137764](https://www.ensembl.org/id/ENSG00000137764) | mitogen-activated protein kinase kinase 5 [Source:HGNC Symbol;Acc:HGNC:6845] | 0,85 |
| MAP2K6 | [ENSG00000108984](https://www.ensembl.org/id/ENSG00000108984) | mitogen-activated protein kinase kinase 6 [Source:HGNC Symbol;Acc:HGNC:6846] | 2,29 |
| MAP3K19 | [ENSG00000176601](https://www.ensembl.org/id/ENSG00000176601) | mitogen-activated protein kinase kinase kinase 19 [Source:HGNC Symbol;Acc:HGNC:26249] | 6,70 |
| MAP3K20 | [ENSG00000091436](https://www.ensembl.org/id/ENSG00000091436) | mitogen-activated protein kinase kinase kinase 20 [Source:HGNC Symbol;Acc:HGNC:17797] | 1,53 |
| MAP3K7CL | [ENSG00000156265](https://www.ensembl.org/id/ENSG00000156265) | MAP3K7 C-terminal like [Source:HGNC Symbol;Acc:HGNC:16457] | 1,27 |
| MAPK12 | [ENSG00000188130](https://www.ensembl.org/id/ENSG00000188130) | mitogen-activated protein kinase 12 [Source:HGNC Symbol;Acc:HGNC:6874] | 2,17 |
| MAPKAPK3 | [ENSG00000114738](https://www.ensembl.org/id/ENSG00000114738) | MAPK activated protein kinase 3 [Source:HGNC Symbol;Acc:HGNC:6888] | 1,64 |
| MAPT | [ENSG00000186868](https://www.ensembl.org/id/ENSG00000186868) | microtubule associated protein tau [Source:HGNC Symbol;Acc:HGNC:6893] | 1,84 |
| MAPT-IT1 | [ENSG00000279685](https://www.ensembl.org/id/ENSG00000279685) | MAPT intronic transcript 1 [Source:HGNC Symbol;Acc:HGNC:43741] | 3,17 |
| MARCKSL1 | [ENSG00000175130](https://www.ensembl.org/id/ENSG00000175130) | MARCKS like 1 [Source:HGNC Symbol;Acc:HGNC:7142] | -1,14 |
| MARK1 | [ENSG00000116141](https://www.ensembl.org/id/ENSG00000116141) | microtubule affinity regulating kinase 1 [Source:HGNC Symbol;Acc:HGNC:6896] | -1,21 |
| MARVELD3 | [ENSG00000140832](https://www.ensembl.org/id/ENSG00000140832) | MARVEL domain containing 3 [Source:HGNC Symbol;Acc:HGNC:30525] | 3,30 |
| MBP | [ENSG00000197971](https://www.ensembl.org/id/ENSG00000197971) | myelin basic protein [Source:HGNC Symbol;Acc:HGNC:6925] | 1,73 |
| MC1R | [ENSG00000258839](https://www.ensembl.org/id/ENSG00000258839) | melanocortin 1 receptor [Source:HGNC Symbol;Acc:HGNC:6929] | -1,64 |
| MCCC2 | [ENSG00000131844](https://www.ensembl.org/id/ENSG00000131844) | methylcrotonyl-CoA carboxylase subunit 2 [Source:HGNC Symbol;Acc:HGNC:6937] | 1,03 |
| MCEE | [ENSG00000124370](https://www.ensembl.org/id/ENSG00000124370) | methylmalonyl-CoA epimerase [Source:HGNC Symbol;Acc:HGNC:16732] | 1,28 |
| MCEMP1 | [ENSG00000183019](https://www.ensembl.org/id/ENSG00000183019) | mast cell expressed membrane protein 1 [Source:HGNC Symbol;Acc:HGNC:27291] | 4,73 |
| MCF2L2 | [ENSG00000053524](https://www.ensembl.org/id/ENSG00000053524) | MCF.2 cell line derived transforming sequence-like 2 [Source:HGNC Symbol;Acc:HGNC:30319] | 2,93 |
| MCRIP2 | [ENSG00000172366](https://www.ensembl.org/id/ENSG00000172366) | MAPK regulated corepressor interacting protein 2 [Source:HGNC Symbol;Acc:HGNC:14142] | 1,54 |
| MDFI | [ENSG00000112559](https://www.ensembl.org/id/ENSG00000112559) | MyoD family inhibitor [Source:HGNC Symbol;Acc:HGNC:6967] | -2,60 |
| MDH1 | [ENSG00000014641](https://www.ensembl.org/id/ENSG00000014641) | malate dehydrogenase 1 [Source:HGNC Symbol;Acc:HGNC:6970] | 1,75 |
| MDH2 | [ENSG00000146701](https://www.ensembl.org/id/ENSG00000146701) | malate dehydrogenase 2 [Source:HGNC Symbol;Acc:HGNC:6971] | 1,59 |
| ME1 | [ENSG00000065833](https://www.ensembl.org/id/ENSG00000065833) | malic enzyme 1 [Source:HGNC Symbol;Acc:HGNC:6983] | 1,03 |
| MEF2B | [ENSG00000213999](https://www.ensembl.org/id/ENSG00000213999) | myocyte enhancer factor 2B [Source:HGNC Symbol;Acc:HGNC:6995] | -1,71 |
| MEF2C | [ENSG00000081189](https://www.ensembl.org/id/ENSG00000081189) | myocyte enhancer factor 2C [Source:HGNC Symbol;Acc:HGNC:6996] | 1,60 |
| MEFV | [ENSG00000103313](https://www.ensembl.org/id/ENSG00000103313) | MEFV innate immuity regulator, pyrin [Source:HGNC Symbol;Acc:HGNC:6998] | 3,56 |
| Metazoa_SRP_142 | [ENSG00000276002](https://www.ensembl.org/id/ENSG00000276002) | Metazoan signal recognition particle RNA [Source:RFAM;Acc:RF00017] | 6,14 |
| Metazoa_SRP_157 | [ENSG00000273866](https://www.ensembl.org/id/ENSG00000273866) | Metazoan signal recognition particle RNA [Source:RFAM;Acc:RF00017] | 4,54 |
| METTL21C | [ENSG00000139780](https://www.ensembl.org/id/ENSG00000139780) | methyltransferase 21C, AARS1 lysine [Source:HGNC Symbol;Acc:HGNC:33717] | 11,14 |
| MFAP3L | [ENSG00000198948](https://www.ensembl.org/id/ENSG00000198948) | microfibril associated protein 3 like [Source:HGNC Symbol;Acc:HGNC:29083] | -1,12 |
| MFHAS1 | [ENSG00000147324](https://www.ensembl.org/id/ENSG00000147324) | multifunctional ROCO family signaling regulator 1 [Source:HGNC Symbol;Acc:HGNC:16982] | -1,18 |
| MFN2 | [ENSG00000116688](https://www.ensembl.org/id/ENSG00000116688) | mitofusin 2 [Source:HGNC Symbol;Acc:HGNC:16877] | 1,50 |
| MFSD2A | [ENSG00000168389](https://www.ensembl.org/id/ENSG00000168389) | major facilitator superfamily domain containing 2A [Source:HGNC Symbol;Acc:HGNC:25897] | -3,09 |
| MGST1 | [ENSG00000008394](https://www.ensembl.org/id/ENSG00000008394) | microsomal glutathione S-transferase 1 [Source:HGNC Symbol;Acc:HGNC:7061] | -2,03 |
| MIA2-AS1 | [ENSG00000258940](https://www.ensembl.org/id/ENSG00000258940) | MIA2 antisense RNA 1 [Source:HGNC Symbol;Acc:HGNC:55436] | 2,83 |
| MICA | [ENSG00000204520](https://www.ensembl.org/id/ENSG00000204520) | MHC class I polypeptide-related sequence A [Source:HGNC Symbol;Acc:HGNC:7090] | -1,39 |
| MIF-AS1 | [ENSG00000218537](https://www.ensembl.org/id/ENSG00000218537) | MIF antisense RNA 1 [Source:HGNC Symbol;Acc:HGNC:27669] | -3,00 |
| MIR122HG | [ENSG00000267391](https://www.ensembl.org/id/ENSG00000267391) | MIR122 host gene [Source:HGNC Symbol;Acc:HGNC:53821] | 5,28 |
| MIR17HG | [ENSG00000215417](https://www.ensembl.org/id/ENSG00000215417) | miR-17-92a-1 cluster host gene [Source:HGNC Symbol;Acc:HGNC:23564] | -3,20 |
| MIR193BHG | [ENSG00000262454](https://www.ensembl.org/id/ENSG00000262454) | MIR193b-365a host gene [Source:HGNC Symbol;Acc:HGNC:51945] | 1,23 |
| MIR222HG | [ENSG00000270069](https://www.ensembl.org/id/ENSG00000270069) | miR222/221 cluster host gene [Source:HGNC Symbol;Acc:HGNC:49555] | -2,73 |
| MIR223HG | [ENSG00000274536](https://www.ensembl.org/id/ENSG00000274536) | MIR223 host gene [Source:HGNC Symbol;Acc:HGNC:54520] | 4,42 |
| MIR4435-2HG | [ENSG00000172965](https://www.ensembl.org/id/ENSG00000172965) | MIR4435-2 host gene [Source:HGNC Symbol;Acc:HGNC:35163] | -1,21 |
| MIRLET7BHG | [ENSG00000197182](https://www.ensembl.org/id/ENSG00000197182) | MIRLET7B host gene [Source:HGNC Symbol;Acc:HGNC:37189] | -1,41 |
| MKRN2OS | [ENSG00000225526](https://www.ensembl.org/id/ENSG00000225526) | MKRN2 opposite strand [Source:HGNC Symbol;Acc:HGNC:40375] | 4,23 |
| MKRN3 | [ENSG00000179455](https://www.ensembl.org/id/ENSG00000179455) | makorin ring finger protein 3 [Source:HGNC Symbol;Acc:HGNC:7114] | 2,95 |
| MLLT11 | [ENSG00000213190](https://www.ensembl.org/id/ENSG00000213190) | MLLT11 transcription factor 7 cofactor [Source:HGNC Symbol;Acc:HGNC:16997] | 2,22 |
| MMADHC | [ENSG00000168288](https://www.ensembl.org/id/ENSG00000168288) | metabolism of cobalamin associated D [Source:HGNC Symbol;Acc:HGNC:25221] | 1,00 |
| MMP25 | [ENSG00000008516](https://www.ensembl.org/id/ENSG00000008516) | matrix metallopeptidase 25 [Source:HGNC Symbol;Acc:HGNC:14246] | 2,66 |
| MNDA | [ENSG00000163563](https://www.ensembl.org/id/ENSG00000163563) | myeloid cell nuclear differentiation antigen [Source:HGNC Symbol;Acc:HGNC:7183] | 2,82 |
| MPC1 | [ENSG00000060762](https://www.ensembl.org/id/ENSG00000060762) | mitochondrial pyruvate carrier 1 [Source:HGNC Symbol;Acc:HGNC:21606] | 1,21 |
| MPC2 | [ENSG00000143158](https://www.ensembl.org/id/ENSG00000143158) | mitochondrial pyruvate carrier 2 [Source:HGNC Symbol;Acc:HGNC:24515] | 1,27 |
| MPIG6B | [ENSG00000204420](https://www.ensembl.org/id/ENSG00000204420) | megakaryocyte and platelet inhibitory receptor G6b [Source:HGNC Symbol;Acc:HGNC:13937] | 2,42 |
| MPP2 | [ENSG00000108852](https://www.ensembl.org/id/ENSG00000108852) | MAGUK p55 scaffold protein 2 [Source:HGNC Symbol;Acc:HGNC:7220] | -1,18 |
| MRAP | [ENSG00000170262](https://www.ensembl.org/id/ENSG00000170262) | melanocortin 2 receptor accessory protein [Source:HGNC Symbol;Acc:HGNC:1304] | -3,43 |
| MRPL1 | [ENSG00000169288](https://www.ensembl.org/id/ENSG00000169288) | mitochondrial ribosomal protein L1 [Source:HGNC Symbol;Acc:HGNC:14275] | 1,17 |
| MRPL12 | [ENSG00000262814](https://www.ensembl.org/id/ENSG00000262814) | mitochondrial ribosomal protein L12 [Source:HGNC Symbol;Acc:HGNC:10378] | 1,24 |
| MRPL14 | [ENSG00000180992](https://www.ensembl.org/id/ENSG00000180992) | mitochondrial ribosomal protein L14 [Source:HGNC Symbol;Acc:HGNC:14279] | 1,48 |
| MRPL15 | [ENSG00000137547](https://www.ensembl.org/id/ENSG00000137547) | mitochondrial ribosomal protein L15 [Source:HGNC Symbol;Acc:HGNC:14054] | 1,33 |
| MRPL2 | [ENSG00000112651](https://www.ensembl.org/id/ENSG00000112651) | mitochondrial ribosomal protein L2 [Source:HGNC Symbol;Acc:HGNC:14056] | 0,96 |
| MRPL21 | [ENSG00000197345](https://www.ensembl.org/id/ENSG00000197345) | mitochondrial ribosomal protein L21 [Source:HGNC Symbol;Acc:HGNC:14479] | 0,93 |
| MRPL33 | [ENSG00000243147](https://www.ensembl.org/id/ENSG00000243147) | mitochondrial ribosomal protein L33 [Source:HGNC Symbol;Acc:HGNC:14487] | 1,04 |
| MRPL37 | [ENSG00000116221](https://www.ensembl.org/id/ENSG00000116221) | mitochondrial ribosomal protein L37 [Source:HGNC Symbol;Acc:HGNC:14034] | 1,34 |
| MRPL40 | [ENSG00000185608](https://www.ensembl.org/id/ENSG00000185608) | mitochondrial ribosomal protein L40 [Source:HGNC Symbol;Acc:HGNC:14491] | 1,14 |
| MRPL46 | [ENSG00000259494](https://www.ensembl.org/id/ENSG00000259494) | mitochondrial ribosomal protein L46 [Source:HGNC Symbol;Acc:HGNC:1192] | 1,15 |
| MRPL57 | [ENSG00000173141](https://www.ensembl.org/id/ENSG00000173141) | mitochondrial ribosomal protein L57 [Source:HGNC Symbol;Acc:HGNC:14514] | 1,01 |
| MRPS11 | [ENSG00000181991](https://www.ensembl.org/id/ENSG00000181991) | mitochondrial ribosomal protein S11 [Source:HGNC Symbol;Acc:HGNC:14050] | 0,94 |
| MRPS15 | [ENSG00000116898](https://www.ensembl.org/id/ENSG00000116898) | mitochondrial ribosomal protein S15 [Source:HGNC Symbol;Acc:HGNC:14504] | 1,01 |
| MRPS18B | [ENSG00000204568](https://www.ensembl.org/id/ENSG00000204568) | mitochondrial ribosomal protein S18B [Source:HGNC Symbol;Acc:HGNC:14516] | 1,31 |
| MRPS24 | [ENSG00000062582](https://www.ensembl.org/id/ENSG00000062582) | mitochondrial ribosomal protein S24 [Source:HGNC Symbol;Acc:HGNC:14510] | 1,06 |
| MRPS28 | [ENSG00000147586](https://www.ensembl.org/id/ENSG00000147586) | mitochondrial ribosomal protein S28 [Source:HGNC Symbol;Acc:HGNC:14513] | 1,43 |
| MRPS30 | [ENSG00000112996](https://www.ensembl.org/id/ENSG00000112996) | mitochondrial ribosomal protein S30 [Source:HGNC Symbol;Acc:HGNC:8769] | 1,06 |
| MRPS33 | [ENSG00000090263](https://www.ensembl.org/id/ENSG00000090263) | mitochondrial ribosomal protein S33 [Source:HGNC Symbol;Acc:HGNC:16634] | 1,41 |
| MRPS35-DT | [ENSG00000256377](https://www.ensembl.org/id/ENSG00000256377) | MRPS35 divergent transcript [Source:HGNC Symbol;Acc:HGNC:55490] | 3,74 |
| MRPS7 | [ENSG00000125445](https://www.ensembl.org/id/ENSG00000125445) | mitochondrial ribosomal protein S7 [Source:HGNC Symbol;Acc:HGNC:14499] | 1,51 |
| MRS2 | [ENSG00000124532](https://www.ensembl.org/id/ENSG00000124532) | magnesium transporter MRS2 [Source:HGNC Symbol;Acc:HGNC:13785] | 1,03 |
| MSLN | [ENSG00000102854](https://www.ensembl.org/id/ENSG00000102854) | mesothelin [Source:HGNC Symbol;Acc:HGNC:7371] | -8,88 |
| MSRB1 | [ENSG00000198736](https://www.ensembl.org/id/ENSG00000198736) | methionine sulfoxide reductase B1 [Source:HGNC Symbol;Acc:HGNC:14133] | 1,10 |
| MT-RNR1 | [ENSG00000211459](https://www.ensembl.org/id/ENSG00000211459) | mitochondrially encoded 12S rRNA [Source:HGNC Symbol;Acc:HGNC:7470] | 1,81 |
| MT1G | [ENSG00000125144](https://www.ensembl.org/id/ENSG00000125144) | metallothionein 1G [Source:HGNC Symbol;Acc:HGNC:7399] | 2,69 |
| MT1X | [ENSG00000187193](https://www.ensembl.org/id/ENSG00000187193) | metallothionein 1X [Source:HGNC Symbol;Acc:HGNC:7405] | 1,24 |
| MTFR1L | [ENSG00000117640](https://www.ensembl.org/id/ENSG00000117640) | mitochondrial fission regulator 1 like [Source:HGNC Symbol;Acc:HGNC:28836] | 1,04 |
| MTIF2 | [ENSG00000085760](https://www.ensembl.org/id/ENSG00000085760) | mitochondrial translational initiation factor 2 [Source:HGNC Symbol;Acc:HGNC:7441] | 1,14 |
| MTLN | [ENSG00000175701](https://www.ensembl.org/id/ENSG00000175701) | mitoregulin [Source:HGNC Symbol;Acc:HGNC:27339] | 1,71 |
| MUC1 | [ENSG00000185499](https://www.ensembl.org/id/ENSG00000185499) | mucin 1, cell surface associated [Source:HGNC Symbol;Acc:HGNC:7508] | -1,99 |
| MUC16 | [ENSG00000181143](https://www.ensembl.org/id/ENSG00000181143) | mucin 16, cell surface associated [Source:HGNC Symbol;Acc:HGNC:15582] | -9,67 |
| MUC6 | [ENSG00000184956](https://www.ensembl.org/id/ENSG00000184956) | mucin 6, oligomeric mucus/gel-forming [Source:HGNC Symbol;Acc:HGNC:7517] | -2,02 |
| MYH14 | [ENSG00000105357](https://www.ensembl.org/id/ENSG00000105357) | myosin heavy chain 14 [Source:HGNC Symbol;Acc:HGNC:23212] | 2,17 |
| MYH15 | [ENSG00000144821](https://www.ensembl.org/id/ENSG00000144821) | myosin heavy chain 15 [Source:HGNC Symbol;Acc:HGNC:31073] | 3,34 |
| MYH3 | [ENSG00000109063](https://www.ensembl.org/id/ENSG00000109063) | myosin heavy chain 3 [Source:HGNC Symbol;Acc:HGNC:7573] | 2,27 |
| MYH7B | [ENSG00000078814](https://www.ensembl.org/id/ENSG00000078814) | myosin heavy chain 7B [Source:HGNC Symbol;Acc:HGNC:15906] | 2,21 |
| MYL12A | [ENSG00000101608](https://www.ensembl.org/id/ENSG00000101608) | myosin light chain 12A [Source:HGNC Symbol;Acc:HGNC:16701] | 2,12 |
| MYL3 | [ENSG00000160808](https://www.ensembl.org/id/ENSG00000160808) | myosin light chain 3 [Source:HGNC Symbol;Acc:HGNC:7584] | 2,78 |
| MYL6B | [ENSG00000196465](https://www.ensembl.org/id/ENSG00000196465) | myosin light chain 6B [Source:HGNC Symbol;Acc:HGNC:29823] | 3,96 |
| MYLK3 | [ENSG00000140795](https://www.ensembl.org/id/ENSG00000140795) | myosin light chain kinase 3 [Source:HGNC Symbol;Acc:HGNC:29826] | 2,82 |
| MYMX | [ENSG00000262179](https://www.ensembl.org/id/ENSG00000262179) | myomixer, myoblast fusion factor [Source:HGNC Symbol;Acc:HGNC:52391] | -2,39 |
| MYO18B | [ENSG00000133454](https://www.ensembl.org/id/ENSG00000133454) | myosin XVIIIB [Source:HGNC Symbol;Acc:HGNC:18150] | 2,12 |
| MYO1F | [ENSG00000142347](https://www.ensembl.org/id/ENSG00000142347) | myosin IF [Source:HGNC Symbol;Acc:HGNC:7600] | 2,01 |
| MYO1G | [ENSG00000136286](https://www.ensembl.org/id/ENSG00000136286) | myosin IG [Source:HGNC Symbol;Acc:HGNC:13880] | 2,12 |
| MYO5B | [ENSG00000167306](https://www.ensembl.org/id/ENSG00000167306) | myosin VB [Source:HGNC Symbol;Acc:HGNC:7603] | -2,17 |
| MYOM1 | [ENSG00000101605](https://www.ensembl.org/id/ENSG00000101605) | myomesin 1 [Source:HGNC Symbol;Acc:HGNC:7613] | 2,50 |
| MYOM2 | [ENSG00000036448](https://www.ensembl.org/id/ENSG00000036448) | myomesin 2 [Source:HGNC Symbol;Acc:HGNC:7614] | 3,45 |
| MYOT | [ENSG00000120729](https://www.ensembl.org/id/ENSG00000120729) | myotilin [Source:HGNC Symbol;Acc:HGNC:12399] | 2,78 |
| MYOZ2 | [ENSG00000172399](https://www.ensembl.org/id/ENSG00000172399) | myozenin 2 [Source:HGNC Symbol;Acc:HGNC:1330] | 3,19 |
| MYOZ3 | [ENSG00000164591](https://www.ensembl.org/id/ENSG00000164591) | myozenin 3 [Source:HGNC Symbol;Acc:HGNC:18565] | 2,41 |
| MYRF | [ENSG00000124920](https://www.ensembl.org/id/ENSG00000124920) | myelin regulatory factor [Source:HGNC Symbol;Acc:HGNC:1181] | -5,31 |
| MZB1 | [ENSG00000170476](https://www.ensembl.org/id/ENSG00000170476) | marginal zone B and B1 cell specific protein [Source:HGNC Symbol;Acc:HGNC:30125] | 3,28 |
| NACAD | [ENSG00000136274](https://www.ensembl.org/id/ENSG00000136274) | NAC alpha domain containing [Source:HGNC Symbol;Acc:HGNC:22196] | -1,03 |
| NAIP | [ENSG00000249437](https://www.ensembl.org/id/ENSG00000249437) | NLR family apoptosis inhibitory protein [Source:HGNC Symbol;Acc:HGNC:7634] | -1,23 |
| NARF-IT1 | [ENSG00000266236](https://www.ensembl.org/id/ENSG00000266236) | NARF intronic transcript 1 [Source:HGNC Symbol;Acc:HGNC:43651] | -2,79 |
| NARS2 | [ENSG00000137513](https://www.ensembl.org/id/ENSG00000137513) | asparaginyl-tRNA synthetase 2, mitochondrial [Source:HGNC Symbol;Acc:HGNC:26274] | 1,25 |
| NATD1 | [ENSG00000274180](https://www.ensembl.org/id/ENSG00000274180) | N-acetyltransferase domain containing 1 [Source:HGNC Symbol;Acc:HGNC:30770] | 1,28 |
| NAV2-AS1 | [ENSG00000254894](https://www.ensembl.org/id/ENSG00000254894) | NAV2 antisense RNA 1 [Source:HGNC Symbol;Acc:HGNC:40744] | -5,44 |
| NAV2-AS2 | [ENSG00000254453](https://www.ensembl.org/id/ENSG00000254453) | NAV2 antisense RNA 2 [Source:HGNC Symbol;Acc:HGNC:40743] | 3,50 |
| NCAM1 | [ENSG00000149294](https://www.ensembl.org/id/ENSG00000149294) | neural cell adhesion molecule 1 [Source:HGNC Symbol;Acc:HGNC:7656] | 1,72 |
| NCKAP1L | [ENSG00000123338](https://www.ensembl.org/id/ENSG00000123338) | NCK associated protein 1 like [Source:HGNC Symbol;Acc:HGNC:4862] | 1,98 |
| NCR3 | [ENSG00000204475](https://www.ensembl.org/id/ENSG00000204475) | natural cytotoxicity triggering receptor 3 [Source:HGNC Symbol;Acc:HGNC:19077] | 5,13 |
| NDUFA1 | [ENSG00000125356](https://www.ensembl.org/id/ENSG00000125356) | NADH:ubiquinone oxidoreductase subunit A1 [Source:HGNC Symbol;Acc:HGNC:7683] | 1,29 |
| NDUFA12 | [ENSG00000184752](https://www.ensembl.org/id/ENSG00000184752) | NADH:ubiquinone oxidoreductase subunit A12 [Source:HGNC Symbol;Acc:HGNC:23987] | 1,34 |
| NDUFA2 | [ENSG00000131495](https://www.ensembl.org/id/ENSG00000131495) | NADH:ubiquinone oxidoreductase subunit A2 [Source:HGNC Symbol;Acc:HGNC:7685] | 0,94 |
| NDUFA3 | [ENSG00000170906](https://www.ensembl.org/id/ENSG00000170906) | NADH:ubiquinone oxidoreductase subunit A3 [Source:HGNC Symbol;Acc:HGNC:7686] | 1,35 |
| NDUFA5 | [ENSG00000128609](https://www.ensembl.org/id/ENSG00000128609) | NADH:ubiquinone oxidoreductase subunit A5 [Source:HGNC Symbol;Acc:HGNC:7688] | 1,18 |
| NDUFA7 | [ENSG00000267855](https://www.ensembl.org/id/ENSG00000267855) | NADH:ubiquinone oxidoreductase subunit A7 [Source:HGNC Symbol;Acc:HGNC:7691] | 1,57 |
| NDUFA8 | [ENSG00000119421](https://www.ensembl.org/id/ENSG00000119421) | NADH:ubiquinone oxidoreductase subunit A8 [Source:HGNC Symbol;Acc:HGNC:7692] | 1,51 |
| NDUFA9 | [ENSG00000139180](https://www.ensembl.org/id/ENSG00000139180) | NADH:ubiquinone oxidoreductase subunit A9 [Source:HGNC Symbol;Acc:HGNC:7693] | 1,61 |
| NDUFAB1 | [ENSG00000004779](https://www.ensembl.org/id/ENSG00000004779) | NADH:ubiquinone oxidoreductase subunit AB1 [Source:HGNC Symbol;Acc:HGNC:7694] | 1,36 |
| NDUFAF1 | [ENSG00000137806](https://www.ensembl.org/id/ENSG00000137806) | NADH:ubiquinone oxidoreductase complex assembly factor 1 [Source:HGNC Symbol;Acc:HGNC:18828] | 1,38 |
| NDUFB10 | [ENSG00000140990](https://www.ensembl.org/id/ENSG00000140990) | NADH:ubiquinone oxidoreductase subunit B10 [Source:HGNC Symbol;Acc:HGNC:7696] | 1,34 |
| NDUFB3 | [ENSG00000119013](https://www.ensembl.org/id/ENSG00000119013) | NADH:ubiquinone oxidoreductase subunit B3 [Source:HGNC Symbol;Acc:HGNC:7698] | 1,81 |
| NDUFB5 | [ENSG00000136521](https://www.ensembl.org/id/ENSG00000136521) | NADH:ubiquinone oxidoreductase subunit B5 [Source:HGNC Symbol;Acc:HGNC:7700] | 1,31 |
| NDUFC1 | [ENSG00000109390](https://www.ensembl.org/id/ENSG00000109390) | NADH:ubiquinone oxidoreductase subunit C1 [Source:HGNC Symbol;Acc:HGNC:7705] | 1,06 |
| NDUFC2-KCTD14 | [ENSG00000259112](https://www.ensembl.org/id/ENSG00000259112) | NDUFC2-KCTD14 readthrough [Source:HGNC Symbol;Acc:HGNC:42956] | 2,80 |
| NDUFS1 | [ENSG00000023228](https://www.ensembl.org/id/ENSG00000023228) | NADH:ubiquinone oxidoreductase core subunit S1 [Source:HGNC Symbol;Acc:HGNC:7707] | 2,04 |
| NDUFS2 | [ENSG00000158864](https://www.ensembl.org/id/ENSG00000158864) | NADH:ubiquinone oxidoreductase core subunit S2 [Source:HGNC Symbol;Acc:HGNC:7708] | 1,57 |
| NDUFS3 | [ENSG00000213619](https://www.ensembl.org/id/ENSG00000213619) | NADH:ubiquinone oxidoreductase core subunit S3 [Source:HGNC Symbol;Acc:HGNC:7710] | 1,33 |
| NDUFS4 | [ENSG00000164258](https://www.ensembl.org/id/ENSG00000164258) | NADH:ubiquinone oxidoreductase subunit S4 [Source:HGNC Symbol;Acc:HGNC:7711] | 1,30 |
| NDUFS7 | [ENSG00000115286](https://www.ensembl.org/id/ENSG00000115286) | NADH:ubiquinone oxidoreductase core subunit S7 [Source:HGNC Symbol;Acc:HGNC:7714] | 1,51 |
| NDUFV2 | [ENSG00000178127](https://www.ensembl.org/id/ENSG00000178127) | NADH:ubiquinone oxidoreductase core subunit V2 [Source:HGNC Symbol;Acc:HGNC:7717] | 1,36 |
| NDUFV3 | [ENSG00000160194](https://www.ensembl.org/id/ENSG00000160194) | NADH:ubiquinone oxidoreductase subunit V3 [Source:HGNC Symbol;Acc:HGNC:7719] | 1,25 |
| NECAB3 | [ENSG00000125967](https://www.ensembl.org/id/ENSG00000125967) | N-terminal EF-hand calcium binding protein 3 [Source:HGNC Symbol;Acc:HGNC:15851] | 1,06 |
| NECTIN1 | [ENSG00000110400](https://www.ensembl.org/id/ENSG00000110400) | nectin cell adhesion molecule 1 [Source:HGNC Symbol;Acc:HGNC:9706] | -1,29 |
| NEDD1 | [ENSG00000139350](https://www.ensembl.org/id/ENSG00000139350) | NEDD1 gamma-tubulin ring complex targeting factor [Source:HGNC Symbol;Acc:HGNC:7723] | 1,33 |
| NEK10 | [ENSG00000163491](https://www.ensembl.org/id/ENSG00000163491) | NIMA related kinase 10 [Source:HGNC Symbol;Acc:HGNC:18592] | 2,30 |
| NEU3 | [ENSG00000162139](https://www.ensembl.org/id/ENSG00000162139) | neuraminidase 3 [Source:HGNC Symbol;Acc:HGNC:7760] | 1,34 |
| NEXMIF | [ENSG00000050030](https://www.ensembl.org/id/ENSG00000050030) | neurite extension and migration factor [Source:HGNC Symbol;Acc:HGNC:29433] | -1,75 |
| NFAM1 | [ENSG00000235568](https://www.ensembl.org/id/ENSG00000235568) | NFAT activating protein with ITAM motif 1 [Source:HGNC Symbol;Acc:HGNC:29872] | 3,35 |
| NIPAL4-DT | [ENSG00000251405](https://www.ensembl.org/id/ENSG00000251405) | NIPAL4 divergent transcript [Source:HGNC Symbol;Acc:HGNC:55542] | -4,28 |
| NIPSNAP2 | [ENSG00000146729](https://www.ensembl.org/id/ENSG00000146729) | nipsnap homolog 2 [Source:HGNC Symbol;Acc:HGNC:4179] | 1,81 |
| NIPSNAP3B | [ENSG00000165028](https://www.ensembl.org/id/ENSG00000165028) | nipsnap homolog 3B [Source:HGNC Symbol;Acc:HGNC:23641] | 1,32 |
| NKG7 | [ENSG00000105374](https://www.ensembl.org/id/ENSG00000105374) | natural killer cell granule protein 7 [Source:HGNC Symbol;Acc:HGNC:7830] | 2,81 |
| NLRC4 | [ENSG00000091106](https://www.ensembl.org/id/ENSG00000091106) | NLR family CARD domain containing 4 [Source:HGNC Symbol;Acc:HGNC:16412] | 3,40 |
| NLRP12 | [ENSG00000142405](https://www.ensembl.org/id/ENSG00000142405) | NLR family pyrin domain containing 12 [Source:HGNC Symbol;Acc:HGNC:22938] | 3,30 |
| NLRP3 | [ENSG00000162711](https://www.ensembl.org/id/ENSG00000162711) | NLR family pyrin domain containing 3 [Source:HGNC Symbol;Acc:HGNC:16400] | 2,68 |
| NMB | [ENSG00000197696](https://www.ensembl.org/id/ENSG00000197696) | neuromedin B [Source:HGNC Symbol;Acc:HGNC:7842] | -1,50 |
| NMRK2 | [ENSG00000077009](https://www.ensembl.org/id/ENSG00000077009) | nicotinamide riboside kinase 2 [Source:HGNC Symbol;Acc:HGNC:17871] | 3,15 |
| NNT | [ENSG00000112992](https://www.ensembl.org/id/ENSG00000112992) | nicotinamide nucleotide transhydrogenase [Source:HGNC Symbol;Acc:HGNC:7863] | 1,89 |
| NNT-AS1 | [ENSG00000248092](https://www.ensembl.org/id/ENSG00000248092) | NNT antisense RNA 1 [Source:HGNC Symbol;Acc:HGNC:49005] | 1,11 |
| NOL3 | [ENSG00000140939](https://www.ensembl.org/id/ENSG00000140939) | nucleolar protein 3 [Source:HGNC Symbol;Acc:HGNC:7869] | 1,00 |
| NOS3 | [ENSG00000164867](https://www.ensembl.org/id/ENSG00000164867) | nitric oxide synthase 3 [Source:HGNC Symbol;Acc:HGNC:7876] | -1,59 |
| NPAS1 | [ENSG00000130751](https://www.ensembl.org/id/ENSG00000130751) | neuronal PAS domain protein 1 [Source:HGNC Symbol;Acc:HGNC:7894] | -2,64 |
| NPFF | [ENSG00000139574](https://www.ensembl.org/id/ENSG00000139574) | neuropeptide FF-amide peptide precursor [Source:HGNC Symbol;Acc:HGNC:7901] | -1,56 |
| NPIPA8 | [ENSG00000214940](https://www.ensembl.org/id/ENSG00000214940) | nuclear pore complex interacting protein family member A8 [Source:HGNC Symbol;Acc:HGNC:41983] | 8,98 |
| NQO1-DT | [ENSG00000262136](https://www.ensembl.org/id/ENSG00000262136) | NQO1 divergent transcript [Source:HGNC Symbol;Acc:HGNC:55344] | 5,34 |
| NR0B2 | [ENSG00000131910](https://www.ensembl.org/id/ENSG00000131910) | nuclear receptor subfamily 0 group B member 2 [Source:HGNC Symbol;Acc:HGNC:7961] | 3,62 |
| NRP2 | [ENSG00000118257](https://www.ensembl.org/id/ENSG00000118257) | neuropilin 2 [Source:HGNC Symbol;Acc:HGNC:8005] | -1,09 |
| NSUN7 | [ENSG00000179299](https://www.ensembl.org/id/ENSG00000179299) | NOP2/Sun RNA methyltransferase family member 7 [Source:HGNC Symbol;Acc:HGNC:25857] | -2,13 |
| NT5C1A | [ENSG00000116981](https://www.ensembl.org/id/ENSG00000116981) | 5'-nucleotidase, cytosolic IA [Source:HGNC Symbol;Acc:HGNC:17819] | 2,05 |
| NTS | [ENSG00000133636](https://www.ensembl.org/id/ENSG00000133636) | neurotensin [Source:HGNC Symbol;Acc:HGNC:8038] | -4,99 |
| NUPR2 | [ENSG00000185290](https://www.ensembl.org/id/ENSG00000185290) | nuclear protein 2, transcriptional regulator [Source:HGNC Symbol;Acc:HGNC:44164] | -2,55 |
| OBI1-AS1 | [ENSG00000234377](https://www.ensembl.org/id/ENSG00000234377) | OBI1 antisense RNA 1 [Source:HGNC Symbol;Acc:HGNC:42700] | 5,65 |
| OBSCN | [ENSG00000154358](https://www.ensembl.org/id/ENSG00000154358) | obscurin, cytoskeletal calmodulin and titin-interacting RhoGEF [Source:HGNC Symbol;Acc:HGNC:15719] | 2,80 |
| ODAD2 | [ENSG00000169126](https://www.ensembl.org/id/ENSG00000169126) | outer dynein arm docking complex subunit 2 [Source:HGNC Symbol;Acc:HGNC:25583] | 2,37 |
| ODAM | [ENSG00000109205](https://www.ensembl.org/id/ENSG00000109205) | odontogenic, ameloblast associated [Source:HGNC Symbol;Acc:HGNC:26043] | -5,84 |
| OGDH | [ENSG00000105953](https://www.ensembl.org/id/ENSG00000105953) | oxoglutarate dehydrogenase [Source:HGNC Symbol;Acc:HGNC:8124] | 1,61 |
| OLFM2 | [ENSG00000105088](https://www.ensembl.org/id/ENSG00000105088) | olfactomedin 2 [Source:HGNC Symbol;Acc:HGNC:17189] | -1,71 |
| OLIG1 | [ENSG00000184221](https://www.ensembl.org/id/ENSG00000184221) | oligodendrocyte transcription factor 1 [Source:HGNC Symbol;Acc:HGNC:16983] | 3,28 |
| OR10Z1 | [ENSG00000198967](https://www.ensembl.org/id/ENSG00000198967) | olfactory receptor family 10 subfamily Z member 1 [Source:HGNC Symbol;Acc:HGNC:14996] | -6,32 |
| OR1J1 | [ENSG00000136834](https://www.ensembl.org/id/ENSG00000136834) | olfactory receptor family 1 subfamily J member 1 [Source:HGNC Symbol;Acc:HGNC:8208] | 4,88 |
| OR2AT4 | [ENSG00000171561](https://www.ensembl.org/id/ENSG00000171561) | olfactory receptor family 2 subfamily AT member 4 [Source:HGNC Symbol;Acc:HGNC:19620] | 5,83 |
| OR2B2 | [ENSG00000168131](https://www.ensembl.org/id/ENSG00000168131) | olfactory receptor family 2 subfamily B member 2 [Source:HGNC Symbol;Acc:HGNC:13966] | 5,65 |
| OR51A7 | [ENSG00000176895](https://www.ensembl.org/id/ENSG00000176895) | olfactory receptor family 51 subfamily A member 7 [Source:HGNC Symbol;Acc:HGNC:15188] | 6,27 |
| OR51E1 | [ENSG00000180785](https://www.ensembl.org/id/ENSG00000180785) | olfactory receptor family 51 subfamily E member 1 [Source:HGNC Symbol;Acc:HGNC:15194] | 2,56 |
| OR51V1 | [ENSG00000176742](https://www.ensembl.org/id/ENSG00000176742) | olfactory receptor family 51 subfamily V member 1 [Source:HGNC Symbol;Acc:HGNC:19597] | 6,50 |
| OR7C1 | [ENSG00000127530](https://www.ensembl.org/id/ENSG00000127530) | olfactory receptor family 7 subfamily C member 1 [Source:HGNC Symbol;Acc:HGNC:8373] | 3,24 |
| ORAI1 | [ENSG00000276045](https://www.ensembl.org/id/ENSG00000276045) | ORAI calcium release-activated calcium modulator 1 [Source:HGNC Symbol;Acc:HGNC:25896] | 1,88 |
| OSCAR | [ENSG00000170909](https://www.ensembl.org/id/ENSG00000170909) | osteoclast associated Ig-like receptor [Source:HGNC Symbol;Acc:HGNC:29960] | 2,93 |
| OSGEPL1-AS1 | [ENSG00000253559](https://www.ensembl.org/id/ENSG00000253559) | OSGEPL1 antisense RNA 1 [Source:HGNC Symbol;Acc:HGNC:41009] | -2,85 |
| OSGIN2 | [ENSG00000164823](https://www.ensembl.org/id/ENSG00000164823) | oxidative stress induced growth inhibitor family member 2 [Source:HGNC Symbol;Acc:HGNC:1355] | 1,19 |
| OSM | [ENSG00000099985](https://www.ensembl.org/id/ENSG00000099985) | oncostatin M [Source:HGNC Symbol;Acc:HGNC:8506] | 2,83 |
| OSMR | [ENSG00000145623](https://www.ensembl.org/id/ENSG00000145623) | oncostatin M receptor [Source:HGNC Symbol;Acc:HGNC:8507] | -1,25 |
| OTUD1 | [ENSG00000165312](https://www.ensembl.org/id/ENSG00000165312) | OTU deubiquitinase 1 [Source:HGNC Symbol;Acc:HGNC:27346] | 1,76 |
| OXA1L | [ENSG00000155463](https://www.ensembl.org/id/ENSG00000155463) | OXA1L mitochondrial inner membrane protein [Source:HGNC Symbol;Acc:HGNC:8526] | 0,99 |
| OXCT1 | [ENSG00000083720](https://www.ensembl.org/id/ENSG00000083720) | 3-oxoacid CoA-transferase 1 [Source:HGNC Symbol;Acc:HGNC:8527] | 1,19 |
| P2RX5 | [ENSG00000083454](https://www.ensembl.org/id/ENSG00000083454) | purinergic receptor P2X 5 [Source:HGNC Symbol;Acc:HGNC:8536] | 2,82 |
| P2RY10 | [ENSG00000078589](https://www.ensembl.org/id/ENSG00000078589) | P2Y receptor family member 10 [Source:HGNC Symbol;Acc:HGNC:19906] | 3,09 |
| P2RY2 | [ENSG00000175591](https://www.ensembl.org/id/ENSG00000175591) | purinergic receptor P2Y2 [Source:HGNC Symbol;Acc:HGNC:8541] | 2,43 |
| PACERR | [ENSG00000273129](https://www.ensembl.org/id/ENSG00000273129) | PTGS2 antisense NFKB1 complex-mediated expression regulator RNA [Source:HGNC Symbol;Acc:HGNC:50552] | -3,23 |
| PACSIN3 | [ENSG00000165912](https://www.ensembl.org/id/ENSG00000165912) | protein kinase C and casein kinase substrate in neurons 3 [Source:HGNC Symbol;Acc:HGNC:8572] | 1,84 |
| PADI4 | [ENSG00000159339](https://www.ensembl.org/id/ENSG00000159339) | peptidyl arginine deiminase 4 [Source:HGNC Symbol;Acc:HGNC:18368] | 4,95 |
| PAIP2B | [ENSG00000124374](https://www.ensembl.org/id/ENSG00000124374) | poly(A) binding protein interacting protein 2B [Source:HGNC Symbol;Acc:HGNC:29200] | 3,00 |
| PANCR | [ENSG00000250103](https://www.ensembl.org/id/ENSG00000250103) | PITX2 adjacent non-coding RNA [Source:HGNC Symbol;Acc:HGNC:52282] | 4,91 |
| PANK1 | [ENSG00000152782](https://www.ensembl.org/id/ENSG00000152782) | pantothenate kinase 1 [Source:HGNC Symbol;Acc:HGNC:8598] | 1,58 |
| PANO1 | [ENSG00000288675](https://www.ensembl.org/id/ENSG00000288675) | proapoptotic nucleolar protein 1 [Source:HGNC Symbol;Acc:HGNC:51237] | -2,30 |
| PAQR9-AS1 | [ENSG00000241570](https://www.ensembl.org/id/ENSG00000241570) | PAQR9 antisense RNA 1 [Source:HGNC Symbol;Acc:HGNC:50861] | 5,46 |
| PARAL1 | [ENSG00000243961](https://www.ensembl.org/id/ENSG00000243961) | PPARG activating RBM14 associated lncRNA 1 [Source:HGNC Symbol;Acc:HGNC:53772] | -3,78 |
| PARVB | [ENSG00000188677](https://www.ensembl.org/id/ENSG00000188677) | parvin beta [Source:HGNC Symbol;Acc:HGNC:14653] | 1,50 |
| PATJ | [ENSG00000132849](https://www.ensembl.org/id/ENSG00000132849) | PATJ crumbs cell polarity complex component [Source:HGNC Symbol;Acc:HGNC:28881] | 1,08 |
| PCBD2 | [ENSG00000132570](https://www.ensembl.org/id/ENSG00000132570) | pterin-4 alpha-carbinolamine dehydratase 2 [Source:HGNC Symbol;Acc:HGNC:24474] | 1,32 |
| PCCB | [ENSG00000114054](https://www.ensembl.org/id/ENSG00000114054) | propionyl-CoA carboxylase subunit beta [Source:HGNC Symbol;Acc:HGNC:8654] | 1,15 |
| PCDH8 | [ENSG00000136099](https://www.ensembl.org/id/ENSG00000136099) | protocadherin 8 [Source:HGNC Symbol;Acc:HGNC:8660] | -2,58 |
| PCDHA10 | [ENSG00000250120](https://www.ensembl.org/id/ENSG00000250120) | protocadherin alpha 10 [Source:HGNC Symbol;Acc:HGNC:8664] | -2,51 |
| PCDHB16 | [ENSG00000272674](https://www.ensembl.org/id/ENSG00000272674) | protocadherin beta 16 [Source:HGNC Symbol;Acc:HGNC:14546] | -1,20 |
| PCDHGC5 | [ENSG00000240764](https://www.ensembl.org/id/ENSG00000240764) | protocadherin gamma subfamily C, 5 [Source:HGNC Symbol;Acc:HGNC:8718] | -2,71 |
| PCF11-AS1 | [ENSG00000269939](https://www.ensembl.org/id/ENSG00000269939) | PCF11 antisense RNA 1 [Source:HGNC Symbol;Acc:HGNC:52263] | -4,96 |
| PCK1 | [ENSG00000124253](https://www.ensembl.org/id/ENSG00000124253) | phosphoenolpyruvate carboxykinase 1 [Source:HGNC Symbol;Acc:HGNC:8724] | -3,14 |
| PCMT1 | [ENSG00000120265](https://www.ensembl.org/id/ENSG00000120265) | protein-L-isoaspartate (D-aspartate) O-methyltransferase [Source:HGNC Symbol;Acc:HGNC:8728] | 1,26 |
| PCMTD1-DT | [ENSG00000228801](https://www.ensembl.org/id/ENSG00000228801) | PCMTD1 divergent transcript [Source:HGNC Symbol;Acc:HGNC:55791] | 1,71 |
| PCOTH | [ENSG00000205861](https://www.ensembl.org/id/ENSG00000205861) | prostate and testis expressed opposite C1QTNF9B and MIPEP [Source:HGNC Symbol;Acc:HGNC:39839] | -2,29 |
| PDCL3 | [ENSG00000115539](https://www.ensembl.org/id/ENSG00000115539) | phosducin like 3 [Source:HGNC Symbol;Acc:HGNC:28860] | 1,14 |
| PDE3B | [ENSG00000152270](https://www.ensembl.org/id/ENSG00000152270) | phosphodiesterase 3B [Source:HGNC Symbol;Acc:HGNC:8779] | -2,00 |
| PDE4DIP | [ENSG00000178104](https://www.ensembl.org/id/ENSG00000178104) | phosphodiesterase 4D interacting protein [Source:HGNC Symbol;Acc:HGNC:15580] | 2,21 |
| PDE7A | [ENSG00000205268](https://www.ensembl.org/id/ENSG00000205268) | phosphodiesterase 7A [Source:HGNC Symbol;Acc:HGNC:8791] | 1,50 |
| PDHA1 | [ENSG00000131828](https://www.ensembl.org/id/ENSG00000131828) | pyruvate dehydrogenase E1 subunit alpha 1 [Source:HGNC Symbol;Acc:HGNC:8806] | 1,22 |
| PDHB | [ENSG00000168291](https://www.ensembl.org/id/ENSG00000168291) | pyruvate dehydrogenase E1 subunit beta [Source:HGNC Symbol;Acc:HGNC:8808] | 1,64 |
| PDHX | [ENSG00000110435](https://www.ensembl.org/id/ENSG00000110435) | pyruvate dehydrogenase complex component X [Source:HGNC Symbol;Acc:HGNC:21350] | 1,68 |
| PDLIM4 | [ENSG00000131435](https://www.ensembl.org/id/ENSG00000131435) | PDZ and LIM domain 4 [Source:HGNC Symbol;Acc:HGNC:16501] | -1,16 |
| PDXDC2P-NPIPB14P | [ENSG00000196696](https://www.ensembl.org/id/ENSG00000196696) | PDXDC2P-NPIPB14P readthrough [Source:NCBI gene (formerly Entrezgene);Acc:283970] | -1,28 |
| PDXP | [ENSG00000241360](https://www.ensembl.org/id/ENSG00000241360) | pyridoxal phosphatase [Source:HGNC Symbol;Acc:HGNC:30259] | 1,25 |
| PDZD3 | [ENSG00000172367](https://www.ensembl.org/id/ENSG00000172367) | PDZ domain containing 3 [Source:HGNC Symbol;Acc:HGNC:19891] | -3,25 |
| PDZD4 | [ENSG00000067840](https://www.ensembl.org/id/ENSG00000067840) | PDZ domain containing 4 [Source:HGNC Symbol;Acc:HGNC:21167] | -1,14 |
| PEBP4 | [ENSG00000134020](https://www.ensembl.org/id/ENSG00000134020) | phosphatidylethanolamine binding protein 4 [Source:HGNC Symbol;Acc:HGNC:28319] | 2,12 |
| PERM1 | [ENSG00000187642](https://www.ensembl.org/id/ENSG00000187642) | PPARGC1 and ESRR induced regulator, muscle 1 [Source:HGNC Symbol;Acc:HGNC:28208] | 2,37 |
| PF4 | [ENSG00000163737](https://www.ensembl.org/id/ENSG00000163737) | platelet factor 4 [Source:HGNC Symbol;Acc:HGNC:8861] | 5,54 |
| PF4V1 | [ENSG00000109272](https://www.ensembl.org/id/ENSG00000109272) | platelet factor 4 variant 1 [Source:HGNC Symbol;Acc:HGNC:8862] | 7,90 |
| PFKFB2 | [ENSG00000123836](https://www.ensembl.org/id/ENSG00000123836) | 6-phosphofructo-2-kinase/fructose-2,6-biphosphatase 2 [Source:HGNC Symbol;Acc:HGNC:8873] | 1,22 |
| PFKFB3 | [ENSG00000170525](https://www.ensembl.org/id/ENSG00000170525) | 6-phosphofructo-2-kinase/fructose-2,6-biphosphatase 3 [Source:HGNC Symbol;Acc:HGNC:8874] | -1,28 |
| PGLYRP1 | [ENSG00000008438](https://www.ensembl.org/id/ENSG00000008438) | peptidoglycan recognition protein 1 [Source:HGNC Symbol;Acc:HGNC:8904] | 5,22 |
| PGPEP1 | [ENSG00000130517](https://www.ensembl.org/id/ENSG00000130517) | pyroglutamyl-peptidase I [Source:HGNC Symbol;Acc:HGNC:13568] | 1,12 |
| PHACTR3 | [ENSG00000087495](https://www.ensembl.org/id/ENSG00000087495) | phosphatase and actin regulator 3 [Source:HGNC Symbol;Acc:HGNC:15833] | -4,44 |
| PHLDA1 | [ENSG00000139289](https://www.ensembl.org/id/ENSG00000139289) | pleckstrin homology like domain family A member 1 [Source:HGNC Symbol;Acc:HGNC:8933] | -1,93 |
| PHPT1 | [ENSG00000054148](https://www.ensembl.org/id/ENSG00000054148) | phosphohistidine phosphatase 1 [Source:HGNC Symbol;Acc:HGNC:30033] | 1,22 |
| PHYH | [ENSG00000107537](https://www.ensembl.org/id/ENSG00000107537) | phytanoyl-CoA 2-hydroxylase [Source:HGNC Symbol;Acc:HGNC:8940] | 1,80 |
| PI3 | [ENSG00000124102](https://www.ensembl.org/id/ENSG00000124102) | peptidase inhibitor 3 [Source:HGNC Symbol;Acc:HGNC:8947] | 3,30 |
| PICSAR | [ENSG00000275874](https://www.ensembl.org/id/ENSG00000275874) | P38 inhibited cutaneous squamous cell carcinoma associated lincRNA [Source:HGNC Symbol;Acc:HGNC:19725] | -2,69 |
| PIK3AP1 | [ENSG00000155629](https://www.ensembl.org/id/ENSG00000155629) | phosphoinositide-3-kinase adaptor protein 1 [Source:HGNC Symbol;Acc:HGNC:30034] | 2,06 |
| PINK1 | [ENSG00000158828](https://www.ensembl.org/id/ENSG00000158828) | PTEN induced kinase 1 [Source:HGNC Symbol;Acc:HGNC:14581] | 1,99 |
| PIP | [ENSG00000159763](https://www.ensembl.org/id/ENSG00000159763) | prolactin induced protein [Source:HGNC Symbol;Acc:HGNC:8993] | 5,19 |
| PITPNA-AS1 | [ENSG00000236618](https://www.ensembl.org/id/ENSG00000236618) | PITPNA antisense RNA 1 [Source:HGNC Symbol;Acc:HGNC:44116] | 1,56 |
| PKD2L2-DT | [ENSG00000250159](https://www.ensembl.org/id/ENSG00000250159) | PKD2L2 divergent transcript [Source:HGNC Symbol;Acc:HGNC:55557] | 2,64 |
| PKHD1 | [ENSG00000170927](https://www.ensembl.org/id/ENSG00000170927) | PKHD1 ciliary IPT domain containing fibrocystin/polyductin [Source:HGNC Symbol;Acc:HGNC:9016] | 3,84 |
| PKHD1L1 | [ENSG00000205038](https://www.ensembl.org/id/ENSG00000205038) | PKHD1 like 1 [Source:HGNC Symbol;Acc:HGNC:20313] | -4,60 |
| PLA2G4C | [ENSG00000105499](https://www.ensembl.org/id/ENSG00000105499) | phospholipase A2 group IVC [Source:HGNC Symbol;Acc:HGNC:9037] | 1,32 |
| PLAAT5 | [ENSG00000168004](https://www.ensembl.org/id/ENSG00000168004) | phospholipase A and acyltransferase 5 [Source:HGNC Symbol;Acc:HGNC:24978] | -2,28 |
| PLCXD1 | [ENSG00000182378](https://www.ensembl.org/id/ENSG00000182378) | phosphatidylinositol specific phospholipase C X domain containing 1 [Source:HGNC Symbol;Acc:HGNC:23148] | -1,48 |
| PLEK | [ENSG00000115956](https://www.ensembl.org/id/ENSG00000115956) | pleckstrin [Source:HGNC Symbol;Acc:HGNC:9070] | 2,31 |
| PLEKHF1 | [ENSG00000166289](https://www.ensembl.org/id/ENSG00000166289) | pleckstrin homology and FYVE domain containing 1 [Source:HGNC Symbol;Acc:HGNC:20764] | 1,05 |
| PLEKHG6 | [ENSG00000008323](https://www.ensembl.org/id/ENSG00000008323) | pleckstrin homology and RhoGEF domain containing G6 [Source:HGNC Symbol;Acc:HGNC:25562] | -3,26 |
| PLIN1 | [ENSG00000166819](https://www.ensembl.org/id/ENSG00000166819) | perilipin 1 [Source:HGNC Symbol;Acc:HGNC:9076] | -3,19 |
| PLK2 | [ENSG00000145632](https://www.ensembl.org/id/ENSG00000145632) | polo like kinase 2 [Source:HGNC Symbol;Acc:HGNC:19699] | -1,04 |
| PLPP7 | [ENSG00000160539](https://www.ensembl.org/id/ENSG00000160539) | phospholipid phosphatase 7 (inactive) [Source:HGNC Symbol;Acc:HGNC:28174] | 1,88 |
| PLXNB3 | [ENSG00000198753](https://www.ensembl.org/id/ENSG00000198753) | plexin B3 [Source:HGNC Symbol;Acc:HGNC:9105] | -2,02 |
| PNISR-AS1 | [ENSG00000228506](https://www.ensembl.org/id/ENSG00000228506) | PNISR antisense RNA 1 [Source:HGNC Symbol;Acc:HGNC:40958] | 1,91 |
| PNPLA4 | [ENSG00000006757](https://www.ensembl.org/id/ENSG00000006757) | patatin like phospholipase domain containing 4 [Source:HGNC Symbol;Acc:HGNC:24887] | 1,30 |
| POLB | [ENSG00000070501](https://www.ensembl.org/id/ENSG00000070501) | DNA polymerase beta [Source:HGNC Symbol;Acc:HGNC:9174] | 1,25 |
| POLR2D | [ENSG00000144231](https://www.ensembl.org/id/ENSG00000144231) | RNA polymerase II subunit D [Source:HGNC Symbol;Acc:HGNC:9191] | 1,09 |
| POTED | [ENSG00000166351](https://www.ensembl.org/id/ENSG00000166351) | POTE ankyrin domain family member D [Source:HGNC Symbol;Acc:HGNC:23822] | 5,17 |
| PPA2 | [ENSG00000138777](https://www.ensembl.org/id/ENSG00000138777) | inorganic pyrophosphatase 2 [Source:HGNC Symbol;Acc:HGNC:28883] | 0,98 |
| PPARA | [ENSG00000186951](https://www.ensembl.org/id/ENSG00000186951) | peroxisome proliferator activated receptor alpha [Source:HGNC Symbol;Acc:HGNC:9232] | 1,32 |
| PPARG | [ENSG00000132170](https://www.ensembl.org/id/ENSG00000132170) | peroxisome proliferator activated receptor gamma [Source:HGNC Symbol;Acc:HGNC:9236] | -1,36 |
| PPBP | [ENSG00000163736](https://www.ensembl.org/id/ENSG00000163736) | pro-platelet basic protein [Source:HGNC Symbol;Acc:HGNC:9240] | 6,05 |
| PPFIA4 | [ENSG00000143847](https://www.ensembl.org/id/ENSG00000143847) | PTPRF interacting protein alpha 4 [Source:HGNC Symbol;Acc:HGNC:9248] | 2,04 |
| PPIP5K1 | [ENSG00000168781](https://www.ensembl.org/id/ENSG00000168781) | diphosphoinositol pentakisphosphate kinase 1 [Source:HGNC Symbol;Acc:HGNC:29023] | 0,90 |
| PPP1R16A | [ENSG00000160972](https://www.ensembl.org/id/ENSG00000160972) | protein phosphatase 1 regulatory subunit 16A [Source:HGNC Symbol;Acc:HGNC:14941] | 1,13 |
| PPP1R1B | [ENSG00000131771](https://www.ensembl.org/id/ENSG00000131771) | protein phosphatase 1 regulatory inhibitor subunit 1B [Source:HGNC Symbol;Acc:HGNC:9287] | -3,48 |
| PPP1R1C | [ENSG00000150722](https://www.ensembl.org/id/ENSG00000150722) | protein phosphatase 1 regulatory inhibitor subunit 1C [Source:HGNC Symbol;Acc:HGNC:14940] | 3,25 |
| PPP2R1B | [ENSG00000137713](https://www.ensembl.org/id/ENSG00000137713) | protein phosphatase 2 scaffold subunit Abeta [Source:HGNC Symbol;Acc:HGNC:9303] | -1,49 |
| PPP2R3A | [ENSG00000073711](https://www.ensembl.org/id/ENSG00000073711) | protein phosphatase 2 regulatory subunit B''alpha [Source:HGNC Symbol;Acc:HGNC:9307] | 2,04 |
| PPP2R3B | [ENSG00000167393](https://www.ensembl.org/id/ENSG00000167393) | protein phosphatase 2 regulatory subunit B''beta [Source:HGNC Symbol;Acc:HGNC:13417] | 1,79 |
| PPRC1 | [ENSG00000148840](https://www.ensembl.org/id/ENSG00000148840) | PPARG related coactivator 1 [Source:HGNC Symbol;Acc:HGNC:30025] | -1,46 |
| PRDX3 | [ENSG00000165672](https://www.ensembl.org/id/ENSG00000165672) | peroxiredoxin 3 [Source:HGNC Symbol;Acc:HGNC:9354] | 1,38 |
| PRECSIT | [ENSG00000255874](https://www.ensembl.org/id/ENSG00000255874) | p53 regulated carcinoma associated Stat3 activating long intergenic non-protein coding transcript [Source:HGNC Symbol;Acc:HGNC:27492] | 1,77 |
| PRF1 | [ENSG00000180644](https://www.ensembl.org/id/ENSG00000180644) | perforin 1 [Source:HGNC Symbol;Acc:HGNC:9360] | 2,35 |
| PRG4 | [ENSG00000116690](https://www.ensembl.org/id/ENSG00000116690) | proteoglycan 4 [Source:HGNC Symbol;Acc:HGNC:9364] | -2,37 |
| PRICKLE4 | [ENSG00000278224](https://www.ensembl.org/id/ENSG00000278224) | prickle planar cell polarity protein 4 [Source:HGNC Symbol;Acc:HGNC:16805] | -3,77 |
| PRKAA2 | [ENSG00000162409](https://www.ensembl.org/id/ENSG00000162409) | protein kinase AMP-activated catalytic subunit alpha 2 [Source:HGNC Symbol;Acc:HGNC:9377] | 1,94 |
| PRKAB2 | [ENSG00000131791](https://www.ensembl.org/id/ENSG00000131791) | protein kinase AMP-activated non-catalytic subunit beta 2 [Source:HGNC Symbol;Acc:HGNC:9379] | 1,74 |
| PRKAR2B | [ENSG00000005249](https://www.ensembl.org/id/ENSG00000005249) | protein kinase cAMP-dependent type II regulatory subunit beta [Source:HGNC Symbol;Acc:HGNC:9392] | -1,47 |
| PRKCG | [ENSG00000126583](https://www.ensembl.org/id/ENSG00000126583) | protein kinase C gamma [Source:HGNC Symbol;Acc:HGNC:9402] | -2,46 |
| PRKCQ-AS1 | [ENSG00000237943](https://www.ensembl.org/id/ENSG00000237943) | PRKCQ antisense RNA 1 [Source:HGNC Symbol;Acc:HGNC:44689] | 2,48 |
| PRKN | [ENSG00000185345](https://www.ensembl.org/id/ENSG00000185345) | parkin RBR E3 ubiquitin protein ligase [Source:HGNC Symbol;Acc:HGNC:8607] | 1,58 |
| PROB1 | [ENSG00000228672](https://www.ensembl.org/id/ENSG00000228672) | proline rich basic protein 1 [Source:HGNC Symbol;Acc:HGNC:41906] | 2,11 |
| PROC | [ENSG00000115718](https://www.ensembl.org/id/ENSG00000115718) | protein C, inactivator of coagulation factors Va and VIIIa [Source:HGNC Symbol;Acc:HGNC:9451] | -3,28 |
| PROK2 | [ENSG00000163421](https://www.ensembl.org/id/ENSG00000163421) | prokineticin 2 [Source:HGNC Symbol;Acc:HGNC:18455] | 4,77 |
| PRPF19-DT | [ENSG00000257052](https://www.ensembl.org/id/ENSG00000257052) | PRPF19 divergent transcript [Source:HGNC Symbol;Acc:HGNC:55484] | 5,08 |
| PRR15-DT | [ENSG00000223813](https://www.ensembl.org/id/ENSG00000223813) | PRR15 divergent transcript [Source:HGNC Symbol;Acc:HGNC:55866] | -4,81 |
| PRR16 | [ENSG00000184838](https://www.ensembl.org/id/ENSG00000184838) | proline rich 16 [Source:HGNC Symbol;Acc:HGNC:29654] | 1,58 |
| PRR32 | [ENSG00000183631](https://www.ensembl.org/id/ENSG00000183631) | proline rich 32 [Source:HGNC Symbol;Acc:HGNC:34498] | -7,97 |
| PRR34-AS1 | [ENSG00000241990](https://www.ensembl.org/id/ENSG00000241990) | PRR34 antisense RNA 1 [Source:HGNC Symbol;Acc:HGNC:50499] | 1,07 |
| PRR5 | [ENSG00000186654](https://www.ensembl.org/id/ENSG00000186654) | proline rich 5 [Source:HGNC Symbol;Acc:HGNC:31682] | -1,84 |
| PRRX2 | [ENSG00000167157](https://www.ensembl.org/id/ENSG00000167157) | paired related homeobox 2 [Source:HGNC Symbol;Acc:HGNC:21338] | -1,44 |
| PRSS50 | [ENSG00000283706](https://www.ensembl.org/id/ENSG00000283706) | serine protease 50 [Source:HGNC Symbol;Acc:HGNC:17910] | -2,73 |
| PSG8-AS1 | [ENSG00000225877](https://www.ensembl.org/id/ENSG00000225877) | PSG8 antisense RNA 1 [Source:HGNC Symbol;Acc:HGNC:52550] | 3,22 |
| PSME4 | [ENSG00000068878](https://www.ensembl.org/id/ENSG00000068878) | proteasome activator subunit 4 [Source:HGNC Symbol;Acc:HGNC:20635] | 1,18 |
| PTCD3 | [ENSG00000132300](https://www.ensembl.org/id/ENSG00000132300) | pentatricopeptide repeat domain 3 [Source:HGNC Symbol;Acc:HGNC:24717] | 0,97 |
| PTCRA | [ENSG00000171611](https://www.ensembl.org/id/ENSG00000171611) | pre T cell antigen receptor alpha [Source:HGNC Symbol;Acc:HGNC:21290] | 7,63 |
| PTGR2 | [ENSG00000140043](https://www.ensembl.org/id/ENSG00000140043) | prostaglandin reductase 2 [Source:HGNC Symbol;Acc:HGNC:20149] | 2,05 |
| PTP4A1 | [ENSG00000112245](https://www.ensembl.org/id/ENSG00000112245) | protein tyrosine phosphatase 4A1 [Source:HGNC Symbol;Acc:HGNC:9634] | 1,74 |
| PTPN3 | [ENSG00000070159](https://www.ensembl.org/id/ENSG00000070159) | protein tyrosine phosphatase non-receptor type 3 [Source:HGNC Symbol;Acc:HGNC:9655] | 1,26 |
| PTPRC | [ENSG00000081237](https://www.ensembl.org/id/ENSG00000081237) | protein tyrosine phosphatase receptor type C [Source:HGNC Symbol;Acc:HGNC:9666] | 2,91 |
| PTPRF | [ENSG00000142949](https://www.ensembl.org/id/ENSG00000142949) | protein tyrosine phosphatase receptor type F [Source:HGNC Symbol;Acc:HGNC:9670] | -1,17 |
| PTPRH | [ENSG00000080031](https://www.ensembl.org/id/ENSG00000080031) | protein tyrosine phosphatase receptor type H [Source:HGNC Symbol;Acc:HGNC:9672] | -3,34 |
| PTPRQ | [ENSG00000139304](https://www.ensembl.org/id/ENSG00000139304) | protein tyrosine phosphatase receptor type Q [Source:HGNC Symbol;Acc:HGNC:9679] | -3,26 |
| PTTG2 | [ENSG00000250254](https://www.ensembl.org/id/ENSG00000250254) | pituitary tumor-transforming 2 [Source:HGNC Symbol;Acc:HGNC:9691] | -3,32 |
| PVRIG | [ENSG00000213413](https://www.ensembl.org/id/ENSG00000213413) | PVR related immunoglobulin domain containing [Source:HGNC Symbol;Acc:HGNC:32190] | 3,42 |
| PXDN | [ENSG00000130508](https://www.ensembl.org/id/ENSG00000130508) | peroxidasin [Source:HGNC Symbol;Acc:HGNC:14966] | -1,05 |
| PXMP2 | [ENSG00000176894](https://www.ensembl.org/id/ENSG00000176894) | peroxisomal membrane protein 2 [Source:HGNC Symbol;Acc:HGNC:9716] | 1,33 |
| QKI | [ENSG00000112531](https://www.ensembl.org/id/ENSG00000112531) | QKI, KH domain containing RNA binding [Source:HGNC Symbol;Acc:HGNC:21100] | 1,20 |
| QRSL1 | [ENSG00000130348](https://www.ensembl.org/id/ENSG00000130348) | glutaminyl-tRNA amidotransferase subunit QRSL1 [Source:HGNC Symbol;Acc:HGNC:21020] | 1,13 |
| RAB26 | [ENSG00000167964](https://www.ensembl.org/id/ENSG00000167964) | RAB26, member RAS oncogene family [Source:HGNC Symbol;Acc:HGNC:14259] | 2,38 |
| RAB37 | [ENSG00000172794](https://www.ensembl.org/id/ENSG00000172794) | RAB37, member RAS oncogene family [Source:HGNC Symbol;Acc:HGNC:30268] | 1,89 |
| RAB39A | [ENSG00000179331](https://www.ensembl.org/id/ENSG00000179331) | RAB39A, member RAS oncogene family [Source:HGNC Symbol;Acc:HGNC:16521] | 4,09 |
| RAB3A | [ENSG00000105649](https://www.ensembl.org/id/ENSG00000105649) | RAB3A, member RAS oncogene family [Source:HGNC Symbol;Acc:HGNC:9777] | 1,43 |
| RAB3C | [ENSG00000152932](https://www.ensembl.org/id/ENSG00000152932) | RAB3C, member RAS oncogene family [Source:HGNC Symbol;Acc:HGNC:30269] | -1,94 |
| RAB40A | [ENSG00000172476](https://www.ensembl.org/id/ENSG00000172476) | RAB40A, member RAS oncogene family [Source:HGNC Symbol;Acc:HGNC:18283] | -2,50 |
| RAB40AL | [ENSG00000102128](https://www.ensembl.org/id/ENSG00000102128) | RAB40A like [Source:HGNC Symbol;Acc:HGNC:25410] | -5,76 |
| RABEP1 | [ENSG00000029725](https://www.ensembl.org/id/ENSG00000029725) | rabaptin, RAB GTPase binding effector protein 1 [Source:HGNC Symbol;Acc:HGNC:17677] | 1,01 |
| RAC2 | [ENSG00000128340](https://www.ensembl.org/id/ENSG00000128340) | Rac family small GTPase 2 [Source:HGNC Symbol;Acc:HGNC:9802] | 2,38 |
| RAET1E | [ENSG00000164520](https://www.ensembl.org/id/ENSG00000164520) | retinoic acid early transcript 1E [Source:HGNC Symbol;Acc:HGNC:16793] | -2,48 |
| RALYL | [ENSG00000184672](https://www.ensembl.org/id/ENSG00000184672) | RALY RNA binding protein like [Source:HGNC Symbol;Acc:HGNC:27036] | 3,57 |
| RASD1 | [ENSG00000108551](https://www.ensembl.org/id/ENSG00000108551) | ras related dexamethasone induced 1 [Source:HGNC Symbol;Acc:HGNC:15828] | -1,76 |
| RASGEF1C | [ENSG00000146090](https://www.ensembl.org/id/ENSG00000146090) | RasGEF domain family member 1C [Source:HGNC Symbol;Acc:HGNC:27400] | 2,60 |
| RASGRF1 | [ENSG00000058335](https://www.ensembl.org/id/ENSG00000058335) | Ras protein specific guanine nucleotide releasing factor 1 [Source:HGNC Symbol;Acc:HGNC:9875] | 3,12 |
| RASGRP1 | [ENSG00000172575](https://www.ensembl.org/id/ENSG00000172575) | RAS guanyl releasing protein 1 [Source:HGNC Symbol;Acc:HGNC:9878] | 1,96 |
| RBL1 | [ENSG00000080839](https://www.ensembl.org/id/ENSG00000080839) | RB transcriptional corepressor like 1 [Source:HGNC Symbol;Acc:HGNC:9893] | 1,52 |
| RBM38 | [ENSG00000132819](https://www.ensembl.org/id/ENSG00000132819) | RNA binding motif protein 38 [Source:HGNC Symbol;Acc:HGNC:15818] | 1,42 |
| RBM38-AS1 | [ENSG00000218018](https://www.ensembl.org/id/ENSG00000218018) | RBM38 antisense RNA 1 [Source:HGNC Symbol;Acc:HGNC:40725] | 2,43 |
| RBP4 | [ENSG00000138207](https://www.ensembl.org/id/ENSG00000138207) | retinol binding protein 4 [Source:HGNC Symbol;Acc:HGNC:9922] | -3,03 |
| RCAN1 | [ENSG00000159200](https://www.ensembl.org/id/ENSG00000159200) | regulator of calcineurin 1 [Source:HGNC Symbol;Acc:HGNC:3040] | -1,75 |
| RCC1L | [ENSG00000274523](https://www.ensembl.org/id/ENSG00000274523) | RCC1 like [Source:HGNC Symbol;Acc:HGNC:14948] | 1,35 |
| RCSD1 | [ENSG00000198771](https://www.ensembl.org/id/ENSG00000198771) | RCSD domain containing 1 [Source:HGNC Symbol;Acc:HGNC:28310] | 1,57 |
| RDH5 | [ENSG00000135437](https://www.ensembl.org/id/ENSG00000135437) | retinol dehydrogenase 5 [Source:HGNC Symbol;Acc:HGNC:9940] | -1,28 |
| REL-DT | [ENSG00000228414](https://www.ensembl.org/id/ENSG00000228414) | REL divergent transcript [Source:HGNC Symbol;Acc:HGNC:49572] | 5,65 |
| RETNLB | [ENSG00000163515](https://www.ensembl.org/id/ENSG00000163515) | resistin like beta [Source:HGNC Symbol;Acc:HGNC:20388] | 3,67 |
| REXO4 | [ENSG00000148300](https://www.ensembl.org/id/ENSG00000148300) | REX4 homolog, 3'-5' exonuclease [Source:HGNC Symbol;Acc:HGNC:12820] | 1,06 |
| RFLNA | [ENSG00000178882](https://www.ensembl.org/id/ENSG00000178882) | refilin A [Source:HGNC Symbol;Acc:HGNC:27051] | 2,16 |
| RFPL4A | [ENSG00000223638](https://www.ensembl.org/id/ENSG00000223638) | ret finger protein like 4A [Source:HGNC Symbol;Acc:HGNC:16449] | 6,15 |
| RFPL4AL1 | [ENSG00000229292](https://www.ensembl.org/id/ENSG00000229292) | ret finger protein like 4A like 1 [Source:HGNC Symbol;Acc:HGNC:45147] | 5,71 |
| RFX8 | [ENSG00000196460](https://www.ensembl.org/id/ENSG00000196460) | regulatory factor X8 [Source:HGNC Symbol;Acc:HGNC:37253] | -3,20 |
| RGPD1 | [ENSG00000187627](https://www.ensembl.org/id/ENSG00000187627) | RANBP2 like and GRIP domain containing 1 [Source:HGNC Symbol;Acc:HGNC:32414] | 2,69 |
| RGS1 | [ENSG00000090104](https://www.ensembl.org/id/ENSG00000090104) | regulator of G protein signaling 1 [Source:HGNC Symbol;Acc:HGNC:9991] | 2,52 |
| RGS11 | [ENSG00000076344](https://www.ensembl.org/id/ENSG00000076344) | regulator of G protein signaling 11 [Source:HGNC Symbol;Acc:HGNC:9993] | -1,17 |
| RGS18 | [ENSG00000150681](https://www.ensembl.org/id/ENSG00000150681) | regulator of G protein signaling 18 [Source:HGNC Symbol;Acc:HGNC:14261] | 6,13 |
| RGS4 | [ENSG00000117152](https://www.ensembl.org/id/ENSG00000117152) | regulator of G protein signaling 4 [Source:HGNC Symbol;Acc:HGNC:10000] | -1,67 |
| RHD | [ENSG00000187010](https://www.ensembl.org/id/ENSG00000187010) | Rh blood group D antigen [Source:HGNC Symbol;Acc:HGNC:10009] | 3,67 |
| RHOA-IT1 | [ENSG00000235908](https://www.ensembl.org/id/ENSG00000235908) | RHOA intronic transcript 1 [Source:HGNC Symbol;Acc:HGNC:41308] | 3,29 |
| RIBC2 | [ENSG00000128408](https://www.ensembl.org/id/ENSG00000128408) | RIB43A domain with coiled-coils 2 [Source:HGNC Symbol;Acc:HGNC:13241] | 3,71 |
| RIMBP3B | [ENSG00000274600](https://www.ensembl.org/id/ENSG00000274600) | RIMS binding protein 3B [Source:HGNC Symbol;Acc:HGNC:33891] | -3,42 |
| RIPOR2 | [ENSG00000111913](https://www.ensembl.org/id/ENSG00000111913) | RHO family interacting cell polarization regulator 2 [Source:HGNC Symbol;Acc:HGNC:13872] | 1,38 |
| RITA1 | [ENSG00000139405](https://www.ensembl.org/id/ENSG00000139405) | RBPJ interacting and tubulin associated 1 [Source:HGNC Symbol;Acc:HGNC:25925] | 1,24 |
| RN7SL144P | [ENSG00000242559](https://www.ensembl.org/id/ENSG00000242559) | RNA, 7SL, cytoplasmic 144, pseudogene [Source:HGNC Symbol;Acc:HGNC:46160] | -5,13 |
| RN7SL230P | [ENSG00000264916](https://www.ensembl.org/id/ENSG00000264916) | RNA, 7SL, cytoplasmic 230, pseudogene [Source:HGNC Symbol;Acc:HGNC:46246] | 5,46 |
| RN7SL395P | [ENSG00000244307](https://www.ensembl.org/id/ENSG00000244307) | RNA, 7SL, cytoplasmic 395, pseudogene [Source:HGNC Symbol;Acc:HGNC:46411] | 4,87 |
| RN7SL497P | [ENSG00000240847](https://www.ensembl.org/id/ENSG00000240847) | RNA, 7SL, cytoplasmic 497, pseudogene [Source:HGNC Symbol;Acc:HGNC:46513] | 4,57 |
| RN7SL689P | [ENSG00000263432](https://www.ensembl.org/id/ENSG00000263432) | RNA, 7SL, cytoplasmic 689, pseudogene [Source:HGNC Symbol;Acc:HGNC:46705] | -5,28 |
| RNF103-CHMP3 | [ENSG00000249884](https://www.ensembl.org/id/ENSG00000249884) | RNF103-CHMP3 readthrough [Source:HGNC Symbol;Acc:HGNC:38847] | 2,53 |
| RNF123 | [ENSG00000164068](https://www.ensembl.org/id/ENSG00000164068) | ring finger protein 123 [Source:HGNC Symbol;Acc:HGNC:21148] | 1,58 |
| RNF141 | [ENSG00000110315](https://www.ensembl.org/id/ENSG00000110315) | ring finger protein 141 [Source:HGNC Symbol;Acc:HGNC:21159] | 0,93 |
| RNF34 | [ENSG00000170633](https://www.ensembl.org/id/ENSG00000170633) | ring finger protein 34 [Source:HGNC Symbol;Acc:HGNC:17297] | 1,17 |
| ROPN1L | [ENSG00000145491](https://www.ensembl.org/id/ENSG00000145491) | rhophilin associated tail protein 1 like [Source:HGNC Symbol;Acc:HGNC:24060] | 3,36 |
| RPA2 | [ENSG00000117748](https://www.ensembl.org/id/ENSG00000117748) | replication protein A2 [Source:HGNC Symbol;Acc:HGNC:10290] | 1,02 |
| RPAP1 | [ENSG00000103932](https://www.ensembl.org/id/ENSG00000103932) | RNA polymerase II associated protein 1 [Source:HGNC Symbol;Acc:HGNC:24567] | 0,90 |
| RPRM | [ENSG00000177519](https://www.ensembl.org/id/ENSG00000177519) | reprimo, TP53 dependent G2 arrest mediator homolog [Source:HGNC Symbol;Acc:HGNC:24201] | -3,44 |
| RPRML | [ENSG00000179673](https://www.ensembl.org/id/ENSG00000179673) | reprimo like [Source:HGNC Symbol;Acc:HGNC:32422] | -2,89 |
| RPSAP58 | [ENSG00000288920](https://www.ensembl.org/id/ENSG00000288920) | ribosomal protein SA pseudogene 58 [Source:HGNC Symbol;Acc:HGNC:36809] | -1,45 |
| RPUSD4 | [ENSG00000165526](https://www.ensembl.org/id/ENSG00000165526) | RNA pseudouridine synthase D4 [Source:HGNC Symbol;Acc:HGNC:25898] | 1,42 |
| RSPO1 | [ENSG00000169218](https://www.ensembl.org/id/ENSG00000169218) | R-spondin 1 [Source:HGNC Symbol;Acc:HGNC:21679] | -6,47 |
| RTCA | [ENSG00000137996](https://www.ensembl.org/id/ENSG00000137996) | RNA 3'-terminal phosphate cyclase [Source:HGNC Symbol;Acc:HGNC:17981] | 0,93 |
| RTEL1-TNFRSF6B | [ENSG00000026036](https://www.ensembl.org/id/ENSG00000026036) | RTEL1-TNFRSF6B readthrough (NMD candidate) [Source:HGNC Symbol;Acc:HGNC:44095] | -1,11 |
| RTL3 | [ENSG00000179300](https://www.ensembl.org/id/ENSG00000179300) | retrotransposon Gag like 3 [Source:HGNC Symbol;Acc:HGNC:22997] | -2,92 |
| RTN2 | [ENSG00000125744](https://www.ensembl.org/id/ENSG00000125744) | reticulon 2 [Source:HGNC Symbol;Acc:HGNC:10468] | 1,91 |
| RTN4IP1 | [ENSG00000130347](https://www.ensembl.org/id/ENSG00000130347) | reticulon 4 interacting protein 1 [Source:HGNC Symbol;Acc:HGNC:18647] | 1,72 |
| RTN4R | [ENSG00000040608](https://www.ensembl.org/id/ENSG00000040608) | reticulon 4 receptor [Source:HGNC Symbol;Acc:HGNC:18601] | -1,81 |
| RTP1 | [ENSG00000175077](https://www.ensembl.org/id/ENSG00000175077) | receptor transporter protein 1 [Source:HGNC Symbol;Acc:HGNC:28580] | 4,91 |
| RUNX3 | [ENSG00000020633](https://www.ensembl.org/id/ENSG00000020633) | RUNX family transcription factor 3 [Source:HGNC Symbol;Acc:HGNC:10473] | 2,21 |
| RYR3 | [ENSG00000198838](https://www.ensembl.org/id/ENSG00000198838) | ryanodine receptor 3 [Source:HGNC Symbol;Acc:HGNC:10485] | 2,10 |
| S100A12 | [ENSG00000163221](https://www.ensembl.org/id/ENSG00000163221) | S100 calcium binding protein A12 [Source:HGNC Symbol;Acc:HGNC:10489] | 3,67 |
| S100A14 | [ENSG00000189334](https://www.ensembl.org/id/ENSG00000189334) | S100 calcium binding protein A14 [Source:HGNC Symbol;Acc:HGNC:18901] | -3,39 |
| S100A8 | [ENSG00000143546](https://www.ensembl.org/id/ENSG00000143546) | S100 calcium binding protein A8 [Source:HGNC Symbol;Acc:HGNC:10498] | 3,89 |
| S100A9 | [ENSG00000163220](https://www.ensembl.org/id/ENSG00000163220) | S100 calcium binding protein A9 [Source:HGNC Symbol;Acc:HGNC:10499] | 3,87 |
| S100P | [ENSG00000163993](https://www.ensembl.org/id/ENSG00000163993) | S100 calcium binding protein P [Source:HGNC Symbol;Acc:HGNC:10504] | 4,38 |
| S1PR5 | [ENSG00000180739](https://www.ensembl.org/id/ENSG00000180739) | sphingosine-1-phosphate receptor 5 [Source:HGNC Symbol;Acc:HGNC:14299] | 1,90 |
| SAA1 | [ENSG00000173432](https://www.ensembl.org/id/ENSG00000173432) | serum amyloid A1 [Source:HGNC Symbol;Acc:HGNC:10513] | -3,33 |
| SAA2 | [ENSG00000134339](https://www.ensembl.org/id/ENSG00000134339) | serum amyloid A2 [Source:HGNC Symbol;Acc:HGNC:10514] | -6,44 |
| SAGSIN1 | [ENSG00000286075](https://www.ensembl.org/id/ENSG00000286075) | salivary gland specific protein SAGSIN1 [Source:NCBI gene (formerly Entrezgene);Acc:122526779] | 1,52 |
| SALRNA2 | [ENSG00000280515](https://www.ensembl.org/id/ENSG00000280515) | senescence associated long non-coding RNA 2 [Source:HGNC Symbol;Acc:HGNC:49003] | -3,05 |
| SAMD13 | [ENSG00000203943](https://www.ensembl.org/id/ENSG00000203943) | sterile alpha motif domain containing 13 [Source:HGNC Symbol;Acc:HGNC:24582] | 2,37 |
| SAMSN1 | [ENSG00000155307](https://www.ensembl.org/id/ENSG00000155307) | SAM domain, SH3 domain and nuclear localization signals 1 [Source:HGNC Symbol;Acc:HGNC:10528] | 2,77 |
| SATB1 | [ENSG00000182568](https://www.ensembl.org/id/ENSG00000182568) | SATB homeobox 1 [Source:HGNC Symbol;Acc:HGNC:10541] | 1,04 |
| SBK1 | [ENSG00000188322](https://www.ensembl.org/id/ENSG00000188322) | SH3 domain binding kinase 1 [Source:HGNC Symbol;Acc:HGNC:17699] | 3,19 |
| SBK2 | [ENSG00000187550](https://www.ensembl.org/id/ENSG00000187550) | SH3 domain binding kinase family member 2 [Source:HGNC Symbol;Acc:HGNC:34416] | 3,27 |
| SCAT8 | [ENSG00000236345](https://www.ensembl.org/id/ENSG00000236345) | S-phase cancer associated transcript 8 [Source:HGNC Symbol;Acc:HGNC:40967] | 3,16 |
| SCD | [ENSG00000099194](https://www.ensembl.org/id/ENSG00000099194) | stearoyl-CoA desaturase [Source:HGNC Symbol;Acc:HGNC:10571] | -3,66 |
| SCEL | [ENSG00000136155](https://www.ensembl.org/id/ENSG00000136155) | sciellin [Source:HGNC Symbol;Acc:HGNC:10573] | -5,54 |
| SCG3 | [ENSG00000104112](https://www.ensembl.org/id/ENSG00000104112) | secretogranin III [Source:HGNC Symbol;Acc:HGNC:13707] | -3,64 |
| SCGB1D2 | [ENSG00000124935](https://www.ensembl.org/id/ENSG00000124935) | secretoglobin family 1D member 2 [Source:HGNC Symbol;Acc:HGNC:18396] | 4,89 |
| SCGB2A2 | [ENSG00000110484](https://www.ensembl.org/id/ENSG00000110484) | secretoglobin family 2A member 2 [Source:HGNC Symbol;Acc:HGNC:7050] | 6,71 |
| SCHIP1 | [ENSG00000151967](https://www.ensembl.org/id/ENSG00000151967) | schwannomin interacting protein 1 [Source:HGNC Symbol;Acc:HGNC:15678] | 1,66 |
| SCML4 | [ENSG00000146285](https://www.ensembl.org/id/ENSG00000146285) | Scm polycomb group protein like 4 [Source:HGNC Symbol;Acc:HGNC:21397] | 3,75 |
| SCN1B | [ENSG00000105711](https://www.ensembl.org/id/ENSG00000105711) | sodium voltage-gated channel beta subunit 1 [Source:HGNC Symbol;Acc:HGNC:10586] | 1,72 |
| SCN2B | [ENSG00000149575](https://www.ensembl.org/id/ENSG00000149575) | sodium voltage-gated channel beta subunit 2 [Source:HGNC Symbol;Acc:HGNC:10589] | 2,55 |
| SCN4A | [ENSG00000007314](https://www.ensembl.org/id/ENSG00000007314) | sodium voltage-gated channel alpha subunit 4 [Source:HGNC Symbol;Acc:HGNC:10591] | 1,88 |
| SCT | [ENSG00000070031](https://www.ensembl.org/id/ENSG00000070031) | secretin [Source:HGNC Symbol;Acc:HGNC:10607] | 3,79 |
| SDC3 | [ENSG00000162512](https://www.ensembl.org/id/ENSG00000162512) | syndecan 3 [Source:HGNC Symbol;Acc:HGNC:10660] | -1,13 |
| SDHA | [ENSG00000073578](https://www.ensembl.org/id/ENSG00000073578) | succinate dehydrogenase complex flavoprotein subunit A [Source:HGNC Symbol;Acc:HGNC:10680] | 1,95 |
| SDHB | [ENSG00000117118](https://www.ensembl.org/id/ENSG00000117118) | succinate dehydrogenase complex iron sulfur subunit B [Source:HGNC Symbol;Acc:HGNC:10681] | 1,55 |
| SDHC | [ENSG00000143252](https://www.ensembl.org/id/ENSG00000143252) | succinate dehydrogenase complex subunit C [Source:HGNC Symbol;Acc:HGNC:10682] | 1,07 |
| SEC16B | [ENSG00000120341](https://www.ensembl.org/id/ENSG00000120341) | SEC16 homolog B, endoplasmic reticulum export factor [Source:HGNC Symbol;Acc:HGNC:30301] | -1,94 |
| SELL | [ENSG00000188404](https://www.ensembl.org/id/ENSG00000188404) | selectin L [Source:HGNC Symbol;Acc:HGNC:10720] | 2,28 |
| SEMA3F-AS1 | [ENSG00000235016](https://www.ensembl.org/id/ENSG00000235016) | SEMA3F antisense RNA 1 [Source:HGNC Symbol;Acc:HGNC:40518] | -1,69 |
| SEMA4D | [ENSG00000187764](https://www.ensembl.org/id/ENSG00000187764) | semaphorin 4D [Source:HGNC Symbol;Acc:HGNC:10732] | 1,37 |
| SEMA6C | [ENSG00000143434](https://www.ensembl.org/id/ENSG00000143434) | semaphorin 6C [Source:HGNC Symbol;Acc:HGNC:10740] | 2,30 |
| SERINC2 | [ENSG00000168528](https://www.ensembl.org/id/ENSG00000168528) | serine incorporator 2 [Source:HGNC Symbol;Acc:HGNC:23231] | 1,97 |
| SERPINA1 | [ENSG00000197249](https://www.ensembl.org/id/ENSG00000197249) | serpin family A member 1 [Source:HGNC Symbol;Acc:HGNC:8941] | 3,74 |
| SERTM1 | [ENSG00000180440](https://www.ensembl.org/id/ENSG00000180440) | serine rich and transmembrane domain containing 1 [Source:HGNC Symbol;Acc:HGNC:33792] | -4,75 |
| SGCG | [ENSG00000102683](https://www.ensembl.org/id/ENSG00000102683) | sarcoglycan gamma [Source:HGNC Symbol;Acc:HGNC:10809] | 2,53 |
| SGK1 | [ENSG00000118515](https://www.ensembl.org/id/ENSG00000118515) | serum/glucocorticoid regulated kinase 1 [Source:HGNC Symbol;Acc:HGNC:10810] | -1,04 |
| SGK2 | [ENSG00000101049](https://www.ensembl.org/id/ENSG00000101049) | serum/glucocorticoid regulated kinase 2 [Source:HGNC Symbol;Acc:HGNC:13900] | -3,20 |
| SGMS2 | [ENSG00000164023](https://www.ensembl.org/id/ENSG00000164023) | sphingomyelin synthase 2 [Source:HGNC Symbol;Acc:HGNC:28395] | -2,02 |
| SH2B2 | [ENSG00000160999](https://www.ensembl.org/id/ENSG00000160999) | SH2B adaptor protein 2 [Source:HGNC Symbol;Acc:HGNC:17381] | 1,79 |
| SH3BGR | [ENSG00000185437](https://www.ensembl.org/id/ENSG00000185437) | SH3 domain binding glutamate rich protein [Source:HGNC Symbol;Acc:HGNC:10822] | 1,80 |
| SH3BP5-AS1 | [ENSG00000224660](https://www.ensembl.org/id/ENSG00000224660) | SH3BP5 antisense RNA 1 [Source:HGNC Symbol;Acc:HGNC:44501] | -0,96 |
| SH3PXD2B | [ENSG00000174705](https://www.ensembl.org/id/ENSG00000174705) | SH3 and PX domains 2B [Source:HGNC Symbol;Acc:HGNC:29242] | -0,94 |
| SHB | [ENSG00000107338](https://www.ensembl.org/id/ENSG00000107338) | SH2 domain containing adaptor protein B [Source:HGNC Symbol;Acc:HGNC:10838] | -1,06 |
| SHISA4 | [ENSG00000198892](https://www.ensembl.org/id/ENSG00000198892) | shisa family member 4 [Source:HGNC Symbol;Acc:HGNC:27139] | 1,42 |
| SHMT2 | [ENSG00000182199](https://www.ensembl.org/id/ENSG00000182199) | serine hydroxymethyltransferase 2 [Source:HGNC Symbol;Acc:HGNC:10852] | 0,84 |
| SIGLEC10 | [ENSG00000142512](https://www.ensembl.org/id/ENSG00000142512) | sialic acid binding Ig like lectin 10 [Source:HGNC Symbol;Acc:HGNC:15620] | 4,44 |
| SIGLEC7 | [ENSG00000168995](https://www.ensembl.org/id/ENSG00000168995) | sialic acid binding Ig like lectin 7 [Source:HGNC Symbol;Acc:HGNC:10876] | 7,44 |
| SIK2 | [ENSG00000170145](https://www.ensembl.org/id/ENSG00000170145) | salt inducible kinase 2 [Source:HGNC Symbol;Acc:HGNC:21680] | -0,99 |
| SILC1 | [ENSG00000232044](https://www.ensembl.org/id/ENSG00000232044) | sciatic injury induced lincRNA upregulator of SOX11 [Source:HGNC Symbol;Acc:HGNC:26403] | -7,15 |
| SIRPB1 | [ENSG00000101307](https://www.ensembl.org/id/ENSG00000101307) | signal regulatory protein beta 1 [Source:HGNC Symbol;Acc:HGNC:15928] | 4,80 |
| SIRPG | [ENSG00000089012](https://www.ensembl.org/id/ENSG00000089012) | signal regulatory protein gamma [Source:HGNC Symbol;Acc:HGNC:15757] | 3,54 |
| SIRT2 | [ENSG00000068903](https://www.ensembl.org/id/ENSG00000068903) | sirtuin 2 [Source:HGNC Symbol;Acc:HGNC:10886] | 1,81 |
| SIRT4 | [ENSG00000089163](https://www.ensembl.org/id/ENSG00000089163) | sirtuin 4 [Source:HGNC Symbol;Acc:HGNC:14932] | 1,93 |
| SIRT5 | [ENSG00000124523](https://www.ensembl.org/id/ENSG00000124523) | sirtuin 5 [Source:HGNC Symbol;Acc:HGNC:14933] | 1,23 |
| SIX1 | [ENSG00000126778](https://www.ensembl.org/id/ENSG00000126778) | SIX homeobox 1 [Source:HGNC Symbol;Acc:HGNC:10887] | 2,03 |
| SLA2 | [ENSG00000101082](https://www.ensembl.org/id/ENSG00000101082) | Src like adaptor 2 [Source:HGNC Symbol;Acc:HGNC:17329] | 2,95 |
| SLAMF1 | [ENSG00000117090](https://www.ensembl.org/id/ENSG00000117090) | signaling lymphocytic activation molecule family member 1 [Source:HGNC Symbol;Acc:HGNC:10903] | 2,43 |
| SLC10A1 | [ENSG00000100652](https://www.ensembl.org/id/ENSG00000100652) | solute carrier family 10 member 1 [Source:HGNC Symbol;Acc:HGNC:10905] | -4,89 |
| SLC11A1 | [ENSG00000018280](https://www.ensembl.org/id/ENSG00000018280) | solute carrier family 11 member 1 [Source:HGNC Symbol;Acc:HGNC:10907] | 2,75 |
| SLC16A12 | [ENSG00000152779](https://www.ensembl.org/id/ENSG00000152779) | solute carrier family 16 member 12 [Source:HGNC Symbol;Acc:HGNC:23094] | 2,55 |
| SLC17A9 | [ENSG00000101194](https://www.ensembl.org/id/ENSG00000101194) | solute carrier family 17 member 9 [Source:HGNC Symbol;Acc:HGNC:16192] | -1,43 |
| SLC18A2-AS1 | [ENSG00000225936](https://www.ensembl.org/id/ENSG00000225936) | SLC18A2 antisense RNA 1 [Source:HGNC Symbol;Acc:HGNC:55843] | 7,25 |
| SLC19A3 | [ENSG00000135917](https://www.ensembl.org/id/ENSG00000135917) | solute carrier family 19 member 3 [Source:HGNC Symbol;Acc:HGNC:16266] | -2,08 |
| SLC22A18AS | [ENSG00000254827](https://www.ensembl.org/id/ENSG00000254827) | SLC22A18 antisense RNA [Source:HGNC Symbol;Acc:HGNC:10965] | -2,05 |
| SLC22A25 | [ENSG00000196600](https://www.ensembl.org/id/ENSG00000196600) | solute carrier family 22 member 25 [Source:HGNC Symbol;Acc:HGNC:32935] | -5,04 |
| SLC24A2 | [ENSG00000155886](https://www.ensembl.org/id/ENSG00000155886) | solute carrier family 24 member 2 [Source:HGNC Symbol;Acc:HGNC:10976] | 4,49 |
| SLC25A11 | [ENSG00000108528](https://www.ensembl.org/id/ENSG00000108528) | solute carrier family 25 member 11 [Source:HGNC Symbol;Acc:HGNC:10981] | 1,53 |
| SLC25A34 | [ENSG00000162461](https://www.ensembl.org/id/ENSG00000162461) | solute carrier family 25 member 34 [Source:HGNC Symbol;Acc:HGNC:27653] | 1,75 |
| SLC25A4 | [ENSG00000151729](https://www.ensembl.org/id/ENSG00000151729) | solute carrier family 25 member 4 [Source:HGNC Symbol;Acc:HGNC:10990] | 1,80 |
| SLC25A42 | [ENSG00000181035](https://www.ensembl.org/id/ENSG00000181035) | solute carrier family 25 member 42 [Source:HGNC Symbol;Acc:HGNC:28380] | 1,84 |
| SLC26A9 | [ENSG00000174502](https://www.ensembl.org/id/ENSG00000174502) | solute carrier family 26 member 9 [Source:HGNC Symbol;Acc:HGNC:14469] | 2,77 |
| SLC28A3 | [ENSG00000197506](https://www.ensembl.org/id/ENSG00000197506) | solute carrier family 28 member 3 [Source:HGNC Symbol;Acc:HGNC:16484] | -7,90 |
| SLC29A4 | [ENSG00000164638](https://www.ensembl.org/id/ENSG00000164638) | solute carrier family 29 member 4 [Source:HGNC Symbol;Acc:HGNC:23097] | -2,16 |
| SLC2A4 | [ENSG00000181856](https://www.ensembl.org/id/ENSG00000181856) | solute carrier family 2 member 4 [Source:HGNC Symbol;Acc:HGNC:11009] | 2,22 |
| SLC30A10 | [ENSG00000196660](https://www.ensembl.org/id/ENSG00000196660) | solute carrier family 30 member 10 [Source:HGNC Symbol;Acc:HGNC:25355] | 6,37 |
| SLC30A2 | [ENSG00000158014](https://www.ensembl.org/id/ENSG00000158014) | solute carrier family 30 member 2 [Source:HGNC Symbol;Acc:HGNC:11013] | 3,52 |
| SLC35F4 | [ENSG00000151812](https://www.ensembl.org/id/ENSG00000151812) | solute carrier family 35 member F4 [Source:HGNC Symbol;Acc:HGNC:19845] | 6,13 |
| SLC35G3 | [ENSG00000164729](https://www.ensembl.org/id/ENSG00000164729) | solute carrier family 35 member G3 [Source:HGNC Symbol;Acc:HGNC:26848] | 6,36 |
| SLC37A3 | [ENSG00000157800](https://www.ensembl.org/id/ENSG00000157800) | solute carrier family 37 member 3 [Source:HGNC Symbol;Acc:HGNC:20651] | -0,91 |
| SLC38A3 | [ENSG00000188338](https://www.ensembl.org/id/ENSG00000188338) | solute carrier family 38 member 3 [Source:HGNC Symbol;Acc:HGNC:18044] | 2,42 |
| SLC39A8 | [ENSG00000138821](https://www.ensembl.org/id/ENSG00000138821) | solute carrier family 39 member 8 [Source:HGNC Symbol;Acc:HGNC:20862] | -1,44 |
| SLC6A12 | [ENSG00000111181](https://www.ensembl.org/id/ENSG00000111181) | solute carrier family 6 member 12 [Source:HGNC Symbol;Acc:HGNC:11045] | -3,24 |
| SLC6A15 | [ENSG00000072041](https://www.ensembl.org/id/ENSG00000072041) | solute carrier family 6 member 15 [Source:HGNC Symbol;Acc:HGNC:13621] | -2,64 |
| SLC6A7 | [ENSG00000011083](https://www.ensembl.org/id/ENSG00000011083) | solute carrier family 6 member 7 [Source:HGNC Symbol;Acc:HGNC:11054] | -4,76 |
| SLC7A1 | [ENSG00000139514](https://www.ensembl.org/id/ENSG00000139514) | solute carrier family 7 member 1 [Source:HGNC Symbol;Acc:HGNC:11057] | -1,08 |
| SLC7A10 | [ENSG00000130876](https://www.ensembl.org/id/ENSG00000130876) | solute carrier family 7 member 10 [Source:HGNC Symbol;Acc:HGNC:11058] | -2,57 |
| SLC7A11-AS1 | [ENSG00000250033](https://www.ensembl.org/id/ENSG00000250033) | SLC7A11 antisense RNA 1 [Source:HGNC Symbol;Acc:HGNC:44064] | 4,87 |
| SLC7A4 | [ENSG00000099960](https://www.ensembl.org/id/ENSG00000099960) | solute carrier family 7 member 4 [Source:HGNC Symbol;Acc:HGNC:11062] | -3,67 |
| SLCO1C1 | [ENSG00000139155](https://www.ensembl.org/id/ENSG00000139155) | solute carrier organic anion transporter family member 1C1 [Source:HGNC Symbol;Acc:HGNC:13819] | -2,48 |
| SLIT1 | [ENSG00000187122](https://www.ensembl.org/id/ENSG00000187122) | slit guidance ligand 1 [Source:HGNC Symbol;Acc:HGNC:11085] | 2,66 |
| SLURP2 | [ENSG00000283992](https://www.ensembl.org/id/ENSG00000283992) | secreted LY6/PLAUR domain containing 2 [Source:HGNC Symbol;Acc:HGNC:25549] | -2,38 |
| SMAD5-AS1 | [ENSG00000164621](https://www.ensembl.org/id/ENSG00000164621) | SMAD5 antisense RNA 1 [Source:HGNC Symbol;Acc:HGNC:30586] | 2,61 |
| SMAD6 | [ENSG00000137834](https://www.ensembl.org/id/ENSG00000137834) | SMAD family member 6 [Source:HGNC Symbol;Acc:HGNC:6772] | -1,14 |
| SMIM20 | [ENSG00000250317](https://www.ensembl.org/id/ENSG00000250317) | small integral membrane protein 20 [Source:HGNC Symbol;Acc:HGNC:37260] | 1,19 |
| SMIM26 | [ENSG00000232388](https://www.ensembl.org/id/ENSG00000232388) | small integral membrane protein 26 [Source:HGNC Symbol;Acc:HGNC:43430] | 0,85 |
| SMIM30 | [ENSG00000214194](https://www.ensembl.org/id/ENSG00000214194) | small integral membrane protein 30 [Source:HGNC Symbol;Acc:HGNC:48953] | 1,05 |
| SMIM32 | [ENSG00000271824](https://www.ensembl.org/id/ENSG00000271824) | small integral membrane protein 32 [Source:HGNC Symbol;Acc:HGNC:53640] | 3,38 |
| SMN2 | [ENSG00000205571](https://www.ensembl.org/id/ENSG00000205571) | survival of motor neuron 2, centromeric [Source:HGNC Symbol;Acc:HGNC:11118] | 1,01 |
| SMOC1 | [ENSG00000198732](https://www.ensembl.org/id/ENSG00000198732) | SPARC related modular calcium binding 1 [Source:HGNC Symbol;Acc:HGNC:20318] | 1,33 |
| SMPDL3B | [ENSG00000130768](https://www.ensembl.org/id/ENSG00000130768) | sphingomyelin phosphodiesterase acid like 3B [Source:HGNC Symbol;Acc:HGNC:21416] | -3,68 |
| SNAI3 | [ENSG00000185669](https://www.ensembl.org/id/ENSG00000185669) | snail family transcriptional repressor 3 [Source:HGNC Symbol;Acc:HGNC:18411] | 4,18 |
| SNAI3-AS1 | [ENSG00000260630](https://www.ensembl.org/id/ENSG00000260630) | SNAI3 antisense RNA 1 [Source:HGNC Symbol;Acc:HGNC:28327] | 1,79 |
| SNAP25 | [ENSG00000132639](https://www.ensembl.org/id/ENSG00000132639) | synaptosome associated protein 25 [Source:HGNC Symbol;Acc:HGNC:11132] | -2,20 |
| SNCB | [ENSG00000074317](https://www.ensembl.org/id/ENSG00000074317) | synuclein beta [Source:HGNC Symbol;Acc:HGNC:11140] | 3,44 |
| SNN | [ENSG00000184602](https://www.ensembl.org/id/ENSG00000184602) | stannin [Source:HGNC Symbol;Acc:HGNC:11149] | 1,16 |
| SNUPN | [ENSG00000169371](https://www.ensembl.org/id/ENSG00000169371) | snurportin 1 [Source:HGNC Symbol;Acc:HGNC:14245] | 1,00 |
| SNX20 | [ENSG00000167208](https://www.ensembl.org/id/ENSG00000167208) | sorting nexin 20 [Source:HGNC Symbol;Acc:HGNC:30390] | 2,22 |
| SOGA3 | [ENSG00000214338](https://www.ensembl.org/id/ENSG00000214338) | SOGA family member 3 [Source:HGNC Symbol;Acc:HGNC:21494] | 2,47 |
| SORD2P | [ENSG00000290387](https://www.ensembl.org/id/ENSG00000290387) | sorbitol dehydrogenase 2, pseudogene [Source:NCBI gene (formerly Entrezgene);Acc:653381] | 6,97 |
| SOX21-AS1 | [ENSG00000227640](https://www.ensembl.org/id/ENSG00000227640) | SOX21 antisense divergent transcript 1 [Source:HGNC Symbol;Acc:HGNC:39807] | 5,71 |
| SOX4 | [ENSG00000124766](https://www.ensembl.org/id/ENSG00000124766) | SRY-box transcription factor 4 [Source:HGNC Symbol;Acc:HGNC:11200] | -1,10 |
| SOX9-AS1 | [ENSG00000234899](https://www.ensembl.org/id/ENSG00000234899) | SOX9 antisense RNA 1 [Source:HGNC Symbol;Acc:HGNC:49321] | 3,73 |
| SP7 | [ENSG00000170374](https://www.ensembl.org/id/ENSG00000170374) | Sp7 transcription factor [Source:HGNC Symbol;Acc:HGNC:17321] | 6,59 |
| SPATA31A6 | [ENSG00000185775](https://www.ensembl.org/id/ENSG00000185775) | SPATA31 subfamily A member 6 [Source:HGNC Symbol;Acc:HGNC:32006] | 5,43 |
| SPATA45 | [ENSG00000185523](https://www.ensembl.org/id/ENSG00000185523) | spermatogenesis associated 45 [Source:HGNC Symbol;Acc:HGNC:33709] | 3,58 |
| SPATS1 | [ENSG00000249481](https://www.ensembl.org/id/ENSG00000249481) | spermatogenesis associated serine rich 1 [Source:HGNC Symbol;Acc:HGNC:22957] | 4,05 |
| SPC25 | [ENSG00000152253](https://www.ensembl.org/id/ENSG00000152253) | SPC25 component of NDC80 kinetochore complex [Source:HGNC Symbol;Acc:HGNC:24031] | -3,93 |
| SPHK1 | [ENSG00000176170](https://www.ensembl.org/id/ENSG00000176170) | sphingosine kinase 1 [Source:HGNC Symbol;Acc:HGNC:11240] | -1,65 |
| SPI1 | [ENSG00000066336](https://www.ensembl.org/id/ENSG00000066336) | Spi-1 proto-oncogene [Source:HGNC Symbol;Acc:HGNC:11241] | 2,18 |
| SPINK2 | [ENSG00000128040](https://www.ensembl.org/id/ENSG00000128040) | serine peptidase inhibitor Kazal type 2 [Source:HGNC Symbol;Acc:HGNC:11245] | 4,39 |
| SPN | [ENSG00000197471](https://www.ensembl.org/id/ENSG00000197471) | sialophorin [Source:HGNC Symbol;Acc:HGNC:11249] | 2,79 |
| SPNS2 | [ENSG00000183018](https://www.ensembl.org/id/ENSG00000183018) | sphingolipid transporter 2 [Source:HGNC Symbol;Acc:HGNC:26992] | -1,16 |
| SPOCK2 | [ENSG00000107742](https://www.ensembl.org/id/ENSG00000107742) | SPARC (osteonectin), cwcv and kazal like domains proteoglycan 2 [Source:HGNC Symbol;Acc:HGNC:13564] | 1,71 |
| SPRR2F | [ENSG00000244094](https://www.ensembl.org/id/ENSG00000244094) | small proline rich protein 2F [Source:HGNC Symbol;Acc:HGNC:11266] | -7,51 |
| SPTB | [ENSG00000070182](https://www.ensembl.org/id/ENSG00000070182) | spectrin beta, erythrocytic [Source:HGNC Symbol;Acc:HGNC:11274] | 1,86 |
| SRGAP1 | [ENSG00000196935](https://www.ensembl.org/id/ENSG00000196935) | SLIT-ROBO Rho GTPase activating protein 1 [Source:HGNC Symbol;Acc:HGNC:17382] | -1,28 |
| SRGAP2B | [ENSG00000196369](https://www.ensembl.org/id/ENSG00000196369) | SLIT-ROBO Rho GTPase activating protein 2B [Source:HGNC Symbol;Acc:HGNC:35237] | -0,88 |
| SRL | [ENSG00000185739](https://www.ensembl.org/id/ENSG00000185739) | sarcalumenin [Source:HGNC Symbol;Acc:HGNC:11295] | 2,18 |
| SRPK3 | [ENSG00000184343](https://www.ensembl.org/id/ENSG00000184343) | SRSF protein kinase 3 [Source:HGNC Symbol;Acc:HGNC:11402] | 1,43 |
| SRPX2 | [ENSG00000102359](https://www.ensembl.org/id/ENSG00000102359) | sushi repeat containing protein X-linked 2 [Source:HGNC Symbol;Acc:HGNC:30668] | -1,50 |
| SSTR4 | [ENSG00000132671](https://www.ensembl.org/id/ENSG00000132671) | somatostatin receptor 4 [Source:HGNC Symbol;Acc:HGNC:11333] | 5,28 |
| ST3GAL3 | [ENSG00000126091](https://www.ensembl.org/id/ENSG00000126091) | ST3 beta-galactoside alpha-2,3-sialyltransferase 3 [Source:HGNC Symbol;Acc:HGNC:10866] | 1,25 |
| ST6GALNAC3 | [ENSG00000184005](https://www.ensembl.org/id/ENSG00000184005) | ST6 N-acetylgalactosaminide alpha-2,6-sialyltransferase 3 [Source:HGNC Symbol;Acc:HGNC:19343] | -1,53 |
| ST8SIA2 | [ENSG00000140557](https://www.ensembl.org/id/ENSG00000140557) | ST8 alpha-N-acetyl-neuraminide alpha-2,8-sialyltransferase 2 [Source:HGNC Symbol;Acc:HGNC:10870] | 2,76 |
| ST8SIA5 | [ENSG00000101638](https://www.ensembl.org/id/ENSG00000101638) | ST8 alpha-N-acetyl-neuraminide alpha-2,8-sialyltransferase 5 [Source:HGNC Symbol;Acc:HGNC:17827] | 2,85 |
| ST8SIA5-DT | [ENSG00000270112](https://www.ensembl.org/id/ENSG00000270112) | ST8SIA5 divergent transcript [Source:HGNC Symbol;Acc:HGNC:55267] | 2,38 |
| STAB2 | [ENSG00000136011](https://www.ensembl.org/id/ENSG00000136011) | stabilin 2 [Source:HGNC Symbol;Acc:HGNC:18629] | -4,56 |
| STAC | [ENSG00000144681](https://www.ensembl.org/id/ENSG00000144681) | SH3 and cysteine rich domain [Source:HGNC Symbol;Acc:HGNC:11353] | 1,58 |
| STATH | [ENSG00000126549](https://www.ensembl.org/id/ENSG00000126549) | statherin [Source:HGNC Symbol;Acc:HGNC:11369] | 6,68 |
| STAU2 | [ENSG00000040341](https://www.ensembl.org/id/ENSG00000040341) | staufen double-stranded RNA binding protein 2 [Source:HGNC Symbol;Acc:HGNC:11371] | 1,19 |
| STC1 | [ENSG00000159167](https://www.ensembl.org/id/ENSG00000159167) | stanniocalcin 1 [Source:HGNC Symbol;Acc:HGNC:11373] | -2,07 |
| STK31 | [ENSG00000196335](https://www.ensembl.org/id/ENSG00000196335) | serine/threonine kinase 31 [Source:HGNC Symbol;Acc:HGNC:11407] | -3,11 |
| STMP1 | [ENSG00000243317](https://www.ensembl.org/id/ENSG00000243317) | short transmembrane mitochondrial protein 1 [Source:HGNC Symbol;Acc:HGNC:41909] | 0,94 |
| STPG2-AS1 | [ENSG00000251620](https://www.ensembl.org/id/ENSG00000251620) | STPG2 antisense RNA 1 [Source:HGNC Symbol;Acc:HGNC:41209] | 5,11 |
| STRADB | [ENSG00000082146](https://www.ensembl.org/id/ENSG00000082146) | STE20 related adaptor beta [Source:HGNC Symbol;Acc:HGNC:13205] | 1,42 |
| STRCP1 | [ENSG00000290691](https://www.ensembl.org/id/ENSG00000290691) | stereocilin pseudogene 1 [Source:NCBI gene (formerly Entrezgene);Acc:554225] | -2,51 |
| STYXL2 | [ENSG00000198842](https://www.ensembl.org/id/ENSG00000198842) | serine/threonine/tyrosine interacting like 2 [Source:HGNC Symbol;Acc:HGNC:25034] | 1,86 |
| SUCLA2 | [ENSG00000136143](https://www.ensembl.org/id/ENSG00000136143) | succinate-CoA ligase ADP-forming subunit beta [Source:HGNC Symbol;Acc:HGNC:11448] | 1,51 |
| SUCLG1 | [ENSG00000163541](https://www.ensembl.org/id/ENSG00000163541) | succinate-CoA ligase GDP/ADP-forming subunit alpha [Source:HGNC Symbol;Acc:HGNC:11449] | 1,15 |
| SUCLG2 | [ENSG00000172340](https://www.ensembl.org/id/ENSG00000172340) | succinate-CoA ligase GDP-forming subunit beta [Source:HGNC Symbol;Acc:HGNC:11450] | 1,09 |
| SUCLG2-DT | [ENSG00000241316](https://www.ensembl.org/id/ENSG00000241316) | SUCLG2 divergent transcript [Source:HGNC Symbol;Acc:HGNC:49643] | 2,37 |
| SULT2B1 | [ENSG00000088002](https://www.ensembl.org/id/ENSG00000088002) | sulfotransferase family 2B member 1 [Source:HGNC Symbol;Acc:HGNC:11459] | 3,19 |
| SUNO1 | [ENSG00000277013](https://www.ensembl.org/id/ENSG00000277013) | S-phase upregulated non-coding 1 [Source:HGNC Symbol;Acc:HGNC:55385] | 2,96 |
| SYNJ2BP | [ENSG00000213463](https://www.ensembl.org/id/ENSG00000213463) | synaptojanin 2 binding protein [Source:HGNC Symbol;Acc:HGNC:18955] | 0,96 |
| SYNPO2L-AS1 | [ENSG00000271848](https://www.ensembl.org/id/ENSG00000271848) | SYNPO2L antisense RNA 1 [Source:HGNC Symbol;Acc:HGNC:55242] | 3,77 |
| SYPL2 | [ENSG00000143028](https://www.ensembl.org/id/ENSG00000143028) | synaptophysin like 2 [Source:HGNC Symbol;Acc:HGNC:27638] | 1,73 |
| SYT4 | [ENSG00000132872](https://www.ensembl.org/id/ENSG00000132872) | synaptotagmin 4 [Source:HGNC Symbol;Acc:HGNC:11512] | -9,13 |
| SYTL4 | [ENSG00000102362](https://www.ensembl.org/id/ENSG00000102362) | synaptotagmin like 4 [Source:HGNC Symbol;Acc:HGNC:15588] | -1,21 |
| TACC2 | [ENSG00000138162](https://www.ensembl.org/id/ENSG00000138162) | transforming acidic coiled-coil containing protein 2 [Source:HGNC Symbol;Acc:HGNC:11523] | 1,40 |
| TARID | [ENSG00000227954](https://www.ensembl.org/id/ENSG00000227954) | TCF21 antisense RNA inducing promoter demethylation [Source:HGNC Symbol;Acc:HGNC:50506] | -3,40 |
| TARS2 | [ENSG00000143374](https://www.ensembl.org/id/ENSG00000143374) | threonyl-tRNA synthetase 2, mitochondrial [Source:HGNC Symbol;Acc:HGNC:30740] | 1,29 |
| TARS3 | [ENSG00000185418](https://www.ensembl.org/id/ENSG00000185418) | threonyl-tRNA synthetase 3 [Source:HGNC Symbol;Acc:HGNC:24728] | 1,13 |
| TAS2R42 | [ENSG00000186136](https://www.ensembl.org/id/ENSG00000186136) | taste 2 receptor member 42 [Source:HGNC Symbol;Acc:HGNC:18888] | 5,44 |
| TASL | [ENSG00000120280](https://www.ensembl.org/id/ENSG00000120280) | TLR adaptor interacting with endolysosomal SLC15A4 [Source:HGNC Symbol;Acc:HGNC:25667] | 7,47 |
| TATDN1 | [ENSG00000147687](https://www.ensembl.org/id/ENSG00000147687) | TatD DNase domain containing 1 [Source:HGNC Symbol;Acc:HGNC:24220] | 0,98 |
| TBC1D10A | [ENSG00000099992](https://www.ensembl.org/id/ENSG00000099992) | TBC1 domain family member 10A [Source:HGNC Symbol;Acc:HGNC:23609] | -1,08 |
| TBC1D3 | [ENSG00000274611](https://www.ensembl.org/id/ENSG00000274611) | TBC1 domain family member 3 [Source:HGNC Symbol;Acc:HGNC:19031] | 7,55 |
| TBC1D3E | [ENSG00000278599](https://www.ensembl.org/id/ENSG00000278599) | TBC1 domain family member 3E [Source:HGNC Symbol;Acc:HGNC:27071] | 7,70 |
| TBC1D3F | [ENSG00000275954](https://www.ensembl.org/id/ENSG00000275954) | TBC1 domain family member 3F [Source:HGNC Symbol;Acc:HGNC:18257] | 7,36 |
| TBC1D3L | [ENSG00000274512](https://www.ensembl.org/id/ENSG00000274512) | TBC1 domain family member 3L [Source:HGNC Symbol;Acc:HGNC:51246] | -1,30 |
| TBC1D4 | [ENSG00000136111](https://www.ensembl.org/id/ENSG00000136111) | TBC1 domain family member 4 [Source:HGNC Symbol;Acc:HGNC:19165] | 1,47 |
| TBILA | [ENSG00000261488](https://www.ensembl.org/id/ENSG00000261488) | TGF-beta induced lncRNA [Source:HGNC Symbol;Acc:HGNC:53943] | -4,40 |
| TBL1XR1-AS1 | [ENSG00000231310](https://www.ensembl.org/id/ENSG00000231310) | TBL1XR1 antisense RNA 1 [Source:HGNC Symbol;Acc:HGNC:41243] | 5,20 |
| TBRG4 | [ENSG00000136270](https://www.ensembl.org/id/ENSG00000136270) | transforming growth factor beta regulator 4 [Source:HGNC Symbol;Acc:HGNC:17443] | 1,04 |
| TBX1 | [ENSG00000184058](https://www.ensembl.org/id/ENSG00000184058) | T-box transcription factor 1 [Source:HGNC Symbol;Acc:HGNC:11592] | 2,44 |
| TBX15 | [ENSG00000092607](https://www.ensembl.org/id/ENSG00000092607) | T-box transcription factor 15 [Source:HGNC Symbol;Acc:HGNC:11594] | 1,96 |
| TBX21 | [ENSG00000073861](https://www.ensembl.org/id/ENSG00000073861) | T-box transcription factor 21 [Source:HGNC Symbol;Acc:HGNC:11599] | 2,79 |
| TBX3-AS1 | [ENSG00000257817](https://www.ensembl.org/id/ENSG00000257817) | TBX3 antisense RNA 1 [Source:HGNC Symbol;Acc:HGNC:55471] | 3,50 |
| TBXAS1 | [ENSG00000059377](https://www.ensembl.org/id/ENSG00000059377) | thromboxane A synthase 1 [Source:HGNC Symbol;Acc:HGNC:11609] | 2,22 |
| TCAIM | [ENSG00000179152](https://www.ensembl.org/id/ENSG00000179152) | T cell activation inhibitor, mitochondrial [Source:HGNC Symbol;Acc:HGNC:25241] | 0,94 |
| TCEA3 | [ENSG00000204219](https://www.ensembl.org/id/ENSG00000204219) | transcription elongation factor A3 [Source:HGNC Symbol;Acc:HGNC:11615] | 1,65 |
| TCERG1L | [ENSG00000176769](https://www.ensembl.org/id/ENSG00000176769) | transcription elongation regulator 1 like [Source:HGNC Symbol;Acc:HGNC:23533] | 5,00 |
| TDRD5 | [ENSG00000162782](https://www.ensembl.org/id/ENSG00000162782) | tudor domain containing 5 [Source:HGNC Symbol;Acc:HGNC:20614] | -2,65 |
| TECRL | [ENSG00000205678](https://www.ensembl.org/id/ENSG00000205678) | trans-2,3-enoyl-CoA reductase like [Source:HGNC Symbol;Acc:HGNC:27365] | 3,60 |
| TERLR1 | [ENSG00000249201](https://www.ensembl.org/id/ENSG00000249201) | TERT regulating lncRNA 1 [Source:HGNC Symbol;Acc:HGNC:52952] | -3,92 |
| TF | [ENSG00000091513](https://www.ensembl.org/id/ENSG00000091513) | transferrin [Source:HGNC Symbol;Acc:HGNC:11740] | -3,28 |
| TFAP2A-AS2 | [ENSG00000285278](https://www.ensembl.org/id/ENSG00000285278) | TFAP2A antisense RNA 2 [Source:NCBI gene (formerly Entrezgene);Acc:109729173] | -4,38 |
| TFF3 | [ENSG00000160180](https://www.ensembl.org/id/ENSG00000160180) | trefoil factor 3 [Source:HGNC Symbol;Acc:HGNC:11757] | -2,79 |
| TGM1 | [ENSG00000092295](https://www.ensembl.org/id/ENSG00000092295) | transglutaminase 1 [Source:HGNC Symbol;Acc:HGNC:11777] | -2,73 |
| THBS1 | [ENSG00000137801](https://www.ensembl.org/id/ENSG00000137801) | thrombospondin 1 [Source:HGNC Symbol;Acc:HGNC:11785] | -2,19 |
| THBS2-AS1 | [ENSG00000226445](https://www.ensembl.org/id/ENSG00000226445) | THBS2 antisense RNA 1 [Source:HGNC Symbol;Acc:HGNC:56059] | -1,61 |
| THBS4 | [ENSG00000113296](https://www.ensembl.org/id/ENSG00000113296) | thrombospondin 4 [Source:HGNC Symbol;Acc:HGNC:11788] | 2,22 |
| THEMIS | [ENSG00000172673](https://www.ensembl.org/id/ENSG00000172673) | thymocyte selection associated [Source:HGNC Symbol;Acc:HGNC:21569] | 2,74 |
| THRSP | [ENSG00000151365](https://www.ensembl.org/id/ENSG00000151365) | thyroid hormone responsive [Source:HGNC Symbol;Acc:HGNC:11800] | -2,95 |
| TICRR | [ENSG00000140534](https://www.ensembl.org/id/ENSG00000140534) | TOPBP1 interacting checkpoint and replication regulator [Source:HGNC Symbol;Acc:HGNC:28704] | -2,00 |
| TIGD3 | [ENSG00000173825](https://www.ensembl.org/id/ENSG00000173825) | tigger transposable element derived 3 [Source:HGNC Symbol;Acc:HGNC:18334] | -2,87 |
| TIGD4 | [ENSG00000169989](https://www.ensembl.org/id/ENSG00000169989) | tigger transposable element derived 4 [Source:HGNC Symbol;Acc:HGNC:18335] | 3,55 |
| TIGIT | [ENSG00000181847](https://www.ensembl.org/id/ENSG00000181847) | T cell immunoreceptor with Ig and ITIM domains [Source:HGNC Symbol;Acc:HGNC:26838] | 4,04 |
| TIMM17A | [ENSG00000134375](https://www.ensembl.org/id/ENSG00000134375) | translocase of inner mitochondrial membrane 17A [Source:HGNC Symbol;Acc:HGNC:17315] | 1,01 |
| TIMM21 | [ENSG00000075336](https://www.ensembl.org/id/ENSG00000075336) | translocase of inner mitochondrial membrane 21 [Source:HGNC Symbol;Acc:HGNC:25010] | 1,29 |
| TIMM8B | [ENSG00000150779](https://www.ensembl.org/id/ENSG00000150779) | translocase of inner mitochondrial membrane 8 homolog B [Source:HGNC Symbol;Acc:HGNC:11818] | 1,00 |
| TIPARP-AS1 | [ENSG00000243926](https://www.ensembl.org/id/ENSG00000243926) | TIPARP antisense RNA 1 [Source:HGNC Symbol;Acc:HGNC:41028] | 2,39 |
| TLCD2 | [ENSG00000185561](https://www.ensembl.org/id/ENSG00000185561) | TLC domain containing 2 [Source:HGNC Symbol;Acc:HGNC:33522] | -1,38 |
| TLR8 | [ENSG00000101916](https://www.ensembl.org/id/ENSG00000101916) | toll like receptor 8 [Source:HGNC Symbol;Acc:HGNC:15632] | 2,60 |
| TM4SF19 | [ENSG00000145107](https://www.ensembl.org/id/ENSG00000145107) | transmembrane 4 L six family member 19 [Source:HGNC Symbol;Acc:HGNC:25167] | 7,89 |
| TM6SF2 | [ENSG00000213996](https://www.ensembl.org/id/ENSG00000213996) | transmembrane 6 superfamily member 2 [Source:HGNC Symbol;Acc:HGNC:11861] | 3,39 |
| TMC2 | [ENSG00000149488](https://www.ensembl.org/id/ENSG00000149488) | transmembrane channel like 2 [Source:HGNC Symbol;Acc:HGNC:16527] | 2,52 |
| TMEFF1 | [ENSG00000241697](https://www.ensembl.org/id/ENSG00000241697) | transmembrane protein with EGF like and two follistatin like domains 1 [Source:HGNC Symbol;Acc:HGNC:11866] | -5,63 |
| TMEM100 | [ENSG00000166292](https://www.ensembl.org/id/ENSG00000166292) | transmembrane protein 100 [Source:HGNC Symbol;Acc:HGNC:25607] | -1,74 |
| TMEM108 | [ENSG00000144868](https://www.ensembl.org/id/ENSG00000144868) | transmembrane protein 108 [Source:HGNC Symbol;Acc:HGNC:28451] | 1,79 |
| TMEM114 | [ENSG00000232258](https://www.ensembl.org/id/ENSG00000232258) | transmembrane protein 114 [Source:HGNC Symbol;Acc:HGNC:33227] | 5,58 |
| TMEM126A | [ENSG00000171202](https://www.ensembl.org/id/ENSG00000171202) | transmembrane protein 126A [Source:HGNC Symbol;Acc:HGNC:25382] | 1,03 |
| TMEM132C | [ENSG00000181234](https://www.ensembl.org/id/ENSG00000181234) | transmembrane protein 132C [Source:HGNC Symbol;Acc:HGNC:25436] | -2,24 |
| TMEM132E | [ENSG00000181291](https://www.ensembl.org/id/ENSG00000181291) | transmembrane protein 132E [Source:HGNC Symbol;Acc:HGNC:26991] | -3,45 |
| TMEM143 | [ENSG00000161558](https://www.ensembl.org/id/ENSG00000161558) | transmembrane protein 143 [Source:HGNC Symbol;Acc:HGNC:25603] | 1,98 |
| TMEM151A | [ENSG00000179292](https://www.ensembl.org/id/ENSG00000179292) | transmembrane protein 151A [Source:HGNC Symbol;Acc:HGNC:28497] | -4,13 |
| TMEM165 | [ENSG00000134851](https://www.ensembl.org/id/ENSG00000134851) | transmembrane protein 165 [Source:HGNC Symbol;Acc:HGNC:30760] | -1,01 |
| TMEM182 | [ENSG00000170417](https://www.ensembl.org/id/ENSG00000170417) | transmembrane protein 182 [Source:HGNC Symbol;Acc:HGNC:26391] | 1,82 |
| TMEM200C | [ENSG00000206432](https://www.ensembl.org/id/ENSG00000206432) | transmembrane protein 200C [Source:HGNC Symbol;Acc:HGNC:37208] | 4,04 |
| TMEM217B | [ENSG00000286105](https://www.ensembl.org/id/ENSG00000286105) | transmembrane protein 217B [Source:HGNC Symbol;Acc:HGNC:55922] | -5,75 |
| TMEM265 | [ENSG00000281991](https://www.ensembl.org/id/ENSG00000281991) | transmembrane protein 265 [Source:HGNC Symbol;Acc:HGNC:51241] | 5,61 |
| TMEM37 | [ENSG00000171227](https://www.ensembl.org/id/ENSG00000171227) | transmembrane protein 37 [Source:HGNC Symbol;Acc:HGNC:18216] | -2,43 |
| TMEM38A | [ENSG00000072954](https://www.ensembl.org/id/ENSG00000072954) | transmembrane protein 38A [Source:HGNC Symbol;Acc:HGNC:28462] | 2,24 |
| TMEM40 | [ENSG00000088726](https://www.ensembl.org/id/ENSG00000088726) | transmembrane protein 40 [Source:HGNC Symbol;Acc:HGNC:25620] | 3,33 |
| TMEM63C | [ENSG00000165548](https://www.ensembl.org/id/ENSG00000165548) | transmembrane protein 63C [Source:HGNC Symbol;Acc:HGNC:23787] | 2,91 |
| TMEM65 | [ENSG00000164983](https://www.ensembl.org/id/ENSG00000164983) | transmembrane protein 65 [Source:HGNC Symbol;Acc:HGNC:25203] | 1,43 |
| TMEM82 | [ENSG00000162460](https://www.ensembl.org/id/ENSG00000162460) | transmembrane protein 82 [Source:HGNC Symbol;Acc:HGNC:32350] | 5,10 |
| TMEM89 | [ENSG00000183396](https://www.ensembl.org/id/ENSG00000183396) | transmembrane protein 89 [Source:HGNC Symbol;Acc:HGNC:32372] | -5,27 |
| TMEM92 | [ENSG00000167105](https://www.ensembl.org/id/ENSG00000167105) | transmembrane protein 92 [Source:HGNC Symbol;Acc:HGNC:26579] | -5,49 |
| TMLHE | [ENSG00000185973](https://www.ensembl.org/id/ENSG00000185973) | trimethyllysine hydroxylase, epsilon [Source:HGNC Symbol;Acc:HGNC:18308] | 1,51 |
| TMPRSS15 | [ENSG00000154646](https://www.ensembl.org/id/ENSG00000154646) | transmembrane serine protease 15 [Source:HGNC Symbol;Acc:HGNC:9490] | 5,76 |
| TNFRSF12A | [ENSG00000006327](https://www.ensembl.org/id/ENSG00000006327) | TNF receptor superfamily member 12A [Source:HGNC Symbol;Acc:HGNC:18152] | -1,72 |
| TNFRSF17 | [ENSG00000048462](https://www.ensembl.org/id/ENSG00000048462) | TNF receptor superfamily member 17 [Source:HGNC Symbol;Acc:HGNC:11913] | 3,55 |
| TNIK | [ENSG00000154310](https://www.ensembl.org/id/ENSG00000154310) | TRAF2 and NCK interacting kinase [Source:HGNC Symbol;Acc:HGNC:30765] | 1,21 |
| TNNI3 | [ENSG00000129991](https://www.ensembl.org/id/ENSG00000129991) | troponin I3, cardiac type [Source:HGNC Symbol;Acc:HGNC:11947] | 3,85 |
| TOMM40L | [ENSG00000158882](https://www.ensembl.org/id/ENSG00000158882) | translocase of outer mitochondrial membrane 40 like [Source:HGNC Symbol;Acc:HGNC:25756] | 1,15 |
| TOX-DT | [ENSG00000167912](https://www.ensembl.org/id/ENSG00000167912) | TOX divergent transcript [Source:HGNC Symbol;Acc:HGNC:55935] | 3,56 |
| TPD52L1 | [ENSG00000111907](https://www.ensembl.org/id/ENSG00000111907) | TPD52 like 1 [Source:HGNC Symbol;Acc:HGNC:12006] | 1,67 |
| TPM3 | [ENSG00000143549](https://www.ensembl.org/id/ENSG00000143549) | tropomyosin 3 [Source:HGNC Symbol;Acc:HGNC:12012] | 2,87 |
| TRAF4 | [ENSG00000076604](https://www.ensembl.org/id/ENSG00000076604) | TNF receptor associated factor 4 [Source:HGNC Symbol;Acc:HGNC:12034] | -1,43 |
| TRARG1 | [ENSG00000184811](https://www.ensembl.org/id/ENSG00000184811) | trafficking regulator of GLUT4 (SLC2A4) 1 [Source:HGNC Symbol;Acc:HGNC:29592] | -1,80 |
| TREM1 | [ENSG00000124731](https://www.ensembl.org/id/ENSG00000124731) | triggering receptor expressed on myeloid cells 1 [Source:HGNC Symbol;Acc:HGNC:17760] | 3,06 |
| TREML1 | [ENSG00000161911](https://www.ensembl.org/id/ENSG00000161911) | triggering receptor expressed on myeloid cells like 1 [Source:HGNC Symbol;Acc:HGNC:20434] | 5,17 |
| TRHDE-AS1 | [ENSG00000236333](https://www.ensembl.org/id/ENSG00000236333) | TRHDE antisense RNA 1 [Source:HGNC Symbol;Acc:HGNC:27471] | -1,61 |
| TRIM10 | [ENSG00000204613](https://www.ensembl.org/id/ENSG00000204613) | tripartite motif containing 10 [Source:HGNC Symbol;Acc:HGNC:10072] | 5,46 |
| TRIM29 | [ENSG00000137699](https://www.ensembl.org/id/ENSG00000137699) | tripartite motif containing 29 [Source:HGNC Symbol;Acc:HGNC:17274] | -2,21 |
| TRIM47 | [ENSG00000132481](https://www.ensembl.org/id/ENSG00000132481) | tripartite motif containing 47 [Source:HGNC Symbol;Acc:HGNC:19020] | -1,05 |
| TRIM7 | [ENSG00000146054](https://www.ensembl.org/id/ENSG00000146054) | tripartite motif containing 7 [Source:HGNC Symbol;Acc:HGNC:16278] | 1,58 |
| TRIM7-AS2 | [ENSG00000250222](https://www.ensembl.org/id/ENSG00000250222) | TRIM7 antisense RNA 2 [Source:HGNC Symbol;Acc:HGNC:56031] | 1,80 |
| TRIP13 | [ENSG00000071539](https://www.ensembl.org/id/ENSG00000071539) | thyroid hormone receptor interactor 13 [Source:HGNC Symbol;Acc:HGNC:12307] | -2,79 |
| TRMT1L | [ENSG00000121486](https://www.ensembl.org/id/ENSG00000121486) | tRNA methyltransferase 1 like [Source:HGNC Symbol;Acc:HGNC:16782] | 1,18 |
| TRMT2B | [ENSG00000188917](https://www.ensembl.org/id/ENSG00000188917) | tRNA methyltransferase 2 homolog B [Source:HGNC Symbol;Acc:HGNC:25748] | 1,08 |
| TRPC5 | [ENSG00000072315](https://www.ensembl.org/id/ENSG00000072315) | transient receptor potential cation channel subfamily C member 5 [Source:HGNC Symbol;Acc:HGNC:12337] | -4,83 |
| TRUB2 | [ENSG00000167112](https://www.ensembl.org/id/ENSG00000167112) | TruB pseudouridine synthase family member 2 [Source:HGNC Symbol;Acc:HGNC:17170] | 1,04 |
| TSFM | [ENSG00000123297](https://www.ensembl.org/id/ENSG00000123297) | Ts translation elongation factor, mitochondrial [Source:HGNC Symbol;Acc:HGNC:12367] | 1,09 |
| TSGA10 | [ENSG00000135951](https://www.ensembl.org/id/ENSG00000135951) | testis specific 10 [Source:HGNC Symbol;Acc:HGNC:14927] | -1,82 |
| TSPAN19 | [ENSG00000231738](https://www.ensembl.org/id/ENSG00000231738) | tetraspanin 19 [Source:HGNC Symbol;Acc:HGNC:31886] | 5,70 |
| TSPEAR-AS1 | [ENSG00000235890](https://www.ensembl.org/id/ENSG00000235890) | TSPEAR antisense RNA 1 [Source:HGNC Symbol;Acc:HGNC:1271] | -2,96 |
| TSSK1B | [ENSG00000212122](https://www.ensembl.org/id/ENSG00000212122) | testis specific serine kinase 1B [Source:HGNC Symbol;Acc:HGNC:14968] | -5,06 |
| TSSK3 | [ENSG00000162526](https://www.ensembl.org/id/ENSG00000162526) | testis specific serine kinase 3 [Source:HGNC Symbol;Acc:HGNC:15473] | -3,46 |
| TTLL6 | [ENSG00000170703](https://www.ensembl.org/id/ENSG00000170703) | tubulin tyrosine ligase like 6 [Source:HGNC Symbol;Acc:HGNC:26664] | -4,60 |
| TTN-AS1 | [ENSG00000237298](https://www.ensembl.org/id/ENSG00000237298) | TTN antisense RNA 1 [Source:HGNC Symbol;Acc:HGNC:44124] | 2,76 |
| TTPA | [ENSG00000137561](https://www.ensembl.org/id/ENSG00000137561) | alpha tocopherol transfer protein [Source:HGNC Symbol;Acc:HGNC:12404] | -2,23 |
| TTPAL | [ENSG00000124120](https://www.ensembl.org/id/ENSG00000124120) | alpha tocopherol transfer protein like [Source:HGNC Symbol;Acc:HGNC:16114] | -1,01 |
| TTYH2 | [ENSG00000141540](https://www.ensembl.org/id/ENSG00000141540) | tweety family member 2 [Source:HGNC Symbol;Acc:HGNC:13877] | 1,21 |
| TUBA3FP | [ENSG00000284130](https://www.ensembl.org/id/ENSG00000284130) | tubulin alpha 3f pseudogene [Source:HGNC Symbol;Acc:HGNC:24067] | 3,31 |
| TUBA4A | [ENSG00000127824](https://www.ensembl.org/id/ENSG00000127824) | tubulin alpha 4a [Source:HGNC Symbol;Acc:HGNC:12407] | 1,25 |
| TUBB1 | [ENSG00000101162](https://www.ensembl.org/id/ENSG00000101162) | tubulin beta 1 class VI [Source:HGNC Symbol;Acc:HGNC:16257] | 4,65 |
| TUNAR | [ENSG00000250366](https://www.ensembl.org/id/ENSG00000250366) | TCL1 upstream neural differentiation-associated RNA [Source:HGNC Symbol;Acc:HGNC:44088] | 4,96 |
| TWF2 | [ENSG00000247596](https://www.ensembl.org/id/ENSG00000247596) | twinfilin actin binding protein 2 [Source:HGNC Symbol;Acc:HGNC:9621] | 2,25 |
| TXLNB | [ENSG00000164440](https://www.ensembl.org/id/ENSG00000164440) | taxilin beta [Source:HGNC Symbol;Acc:HGNC:21617] | 2,37 |
| TYRO3 | [ENSG00000092445](https://www.ensembl.org/id/ENSG00000092445) | TYRO3 protein tyrosine kinase [Source:HGNC Symbol;Acc:HGNC:12446] | -1,50 |
| TYRP1 | [ENSG00000107165](https://www.ensembl.org/id/ENSG00000107165) | tyrosinase related protein 1 [Source:HGNC Symbol;Acc:HGNC:12450] | 2,48 |
| UBAC1 | [ENSG00000130560](https://www.ensembl.org/id/ENSG00000130560) | UBA domain containing 1 [Source:HGNC Symbol;Acc:HGNC:30221] | 1,34 |
| UBE2D4 | [ENSG00000078967](https://www.ensembl.org/id/ENSG00000078967) | ubiquitin conjugating enzyme E2 D4 (putative) [Source:HGNC Symbol;Acc:HGNC:21647] | 1,19 |
| UBE2QL1 | [ENSG00000215218](https://www.ensembl.org/id/ENSG00000215218) | ubiquitin conjugating enzyme E2 Q family like 1 [Source:HGNC Symbol;Acc:HGNC:37269] | 1,74 |
| UBE2T | [ENSG00000077152](https://www.ensembl.org/id/ENSG00000077152) | ubiquitin conjugating enzyme E2 T [Source:HGNC Symbol;Acc:HGNC:25009] | 2,47 |
| UBL4A | [ENSG00000102178](https://www.ensembl.org/id/ENSG00000102178) | ubiquitin like 4A [Source:HGNC Symbol;Acc:HGNC:12505] | 0,95 |
| UBXN7-AS1 | [ENSG00000225822](https://www.ensembl.org/id/ENSG00000225822) | UBXN7 antisense RNA 1 [Source:HGNC Symbol;Acc:HGNC:41227] | 6,15 |
| UNC5D | [ENSG00000156687](https://www.ensembl.org/id/ENSG00000156687) | unc-5 netrin receptor D [Source:HGNC Symbol;Acc:HGNC:18634] | 4,14 |
| UPK1B | [ENSG00000114638](https://www.ensembl.org/id/ENSG00000114638) | uroplakin 1B [Source:HGNC Symbol;Acc:HGNC:12578] | -8,19 |
| UPK3A | [ENSG00000100373](https://www.ensembl.org/id/ENSG00000100373) | uroplakin 3A [Source:HGNC Symbol;Acc:HGNC:12580] | 2,59 |
| UPK3B | [ENSG00000243566](https://www.ensembl.org/id/ENSG00000243566) | uroplakin 3B [Source:HGNC Symbol;Acc:HGNC:21444] | -7,28 |
| UPK3BL2 | [ENSG00000284981](https://www.ensembl.org/id/ENSG00000284981) | uroplakin 3B like 2 [Source:HGNC Symbol;Acc:HGNC:53444] | -7,34 |
| UQCC1 | [ENSG00000101019](https://www.ensembl.org/id/ENSG00000101019) | ubiquinol-cytochrome c reductase complex assembly factor 1 [Source:HGNC Symbol;Acc:HGNC:15891] | 1,27 |
| UQCR10 | [ENSG00000184076](https://www.ensembl.org/id/ENSG00000184076) | ubiquinol-cytochrome c reductase, complex III subunit X [Source:HGNC Symbol;Acc:HGNC:30863] | 1,71 |
| UQCRC1 | [ENSG00000010256](https://www.ensembl.org/id/ENSG00000010256) | ubiquinol-cytochrome c reductase core protein 1 [Source:HGNC Symbol;Acc:HGNC:12585] | 1,57 |
| UQCRC2 | [ENSG00000140740](https://www.ensembl.org/id/ENSG00000140740) | ubiquinol-cytochrome c reductase core protein 2 [Source:HGNC Symbol;Acc:HGNC:12586] | 1,74 |
| UQCRFS1 | [ENSG00000169021](https://www.ensembl.org/id/ENSG00000169021) | ubiquinol-cytochrome c reductase, Rieske iron-sulfur polypeptide 1 [Source:HGNC Symbol;Acc:HGNC:12587] | 1,62 |
| UQCRQ | [ENSG00000164405](https://www.ensembl.org/id/ENSG00000164405) | ubiquinol-cytochrome c reductase complex III subunit VII [Source:HGNC Symbol;Acc:HGNC:29594] | 1,20 |
| UROC1 | [ENSG00000159650](https://www.ensembl.org/id/ENSG00000159650) | urocanate hydratase 1 [Source:HGNC Symbol;Acc:HGNC:26444] | -5,16 |
| UROS | [ENSG00000188690](https://www.ensembl.org/id/ENSG00000188690) | uroporphyrinogen III synthase [Source:HGNC Symbol;Acc:HGNC:12592] | 1,11 |
| USO1 | [ENSG00000138768](https://www.ensembl.org/id/ENSG00000138768) | USO1 vesicle transport factor [Source:HGNC Symbol;Acc:HGNC:30904] | 1,10 |
| USP13 | [ENSG00000058056](https://www.ensembl.org/id/ENSG00000058056) | ubiquitin specific peptidase 13 [Source:HGNC Symbol;Acc:HGNC:12611] | 2,34 |
| USP28 | [ENSG00000048028](https://www.ensembl.org/id/ENSG00000048028) | ubiquitin specific peptidase 28 [Source:HGNC Symbol;Acc:HGNC:12625] | 1,45 |
| UTP11 | [ENSG00000183520](https://www.ensembl.org/id/ENSG00000183520) | UTP11 small subunit processome component [Source:HGNC Symbol;Acc:HGNC:24329] | 1,15 |
| VAT1L | [ENSG00000171724](https://www.ensembl.org/id/ENSG00000171724) | vesicle amine transport 1 like [Source:HGNC Symbol;Acc:HGNC:29315] | -1,27 |
| VBP1 | [ENSG00000155959](https://www.ensembl.org/id/ENSG00000155959) | VHL binding protein 1 [Source:HGNC Symbol;Acc:HGNC:12662] | 1,12 |
| VDAC1 | [ENSG00000213585](https://www.ensembl.org/id/ENSG00000213585) | voltage dependent anion channel 1 [Source:HGNC Symbol;Acc:HGNC:12669] | 1,50 |
| VDAC3 | [ENSG00000078668](https://www.ensembl.org/id/ENSG00000078668) | voltage dependent anion channel 3 [Source:HGNC Symbol;Acc:HGNC:12674] | 1,65 |
| VENTX | [ENSG00000151650](https://www.ensembl.org/id/ENSG00000151650) | VENT homeobox [Source:HGNC Symbol;Acc:HGNC:13639] | 2,48 |
| VGLL2 | [ENSG00000170162](https://www.ensembl.org/id/ENSG00000170162) | vestigial like family member 2 [Source:HGNC Symbol;Acc:HGNC:20232] | 2,70 |
| VIL1 | [ENSG00000127831](https://www.ensembl.org/id/ENSG00000127831) | villin 1 [Source:HGNC Symbol;Acc:HGNC:12690] | 8,08 |
| VIPR1-AS1 | [ENSG00000232354](https://www.ensembl.org/id/ENSG00000232354) | VIPR1 antisense RNA 1 [Source:HGNC Symbol;Acc:HGNC:40610] | -2,81 |
| VN1R1 | [ENSG00000178201](https://www.ensembl.org/id/ENSG00000178201) | vomeronasal 1 receptor 1 [Source:HGNC Symbol;Acc:HGNC:13548] | 2,55 |
| VNN2 | [ENSG00000112303](https://www.ensembl.org/id/ENSG00000112303) | vanin 2 [Source:HGNC Symbol;Acc:HGNC:12706] | 4,71 |
| VSIG4 | [ENSG00000155659](https://www.ensembl.org/id/ENSG00000155659) | V-set and immunoglobulin domain containing 4 [Source:HGNC Symbol;Acc:HGNC:17032] | 1,82 |
| VSIG8 | [ENSG00000243284](https://www.ensembl.org/id/ENSG00000243284) | V-set and immunoglobulin domain containing 8 [Source:HGNC Symbol;Acc:HGNC:32063] | -3,21 |
| VSX1 | [ENSG00000100987](https://www.ensembl.org/id/ENSG00000100987) | visual system homeobox 1 [Source:HGNC Symbol;Acc:HGNC:12723] | 6,46 |
| VWA8 | [ENSG00000102763](https://www.ensembl.org/id/ENSG00000102763) | von Willebrand factor A domain containing 8 [Source:HGNC Symbol;Acc:HGNC:29071] | 1,34 |
| VWC2 | [ENSG00000188730](https://www.ensembl.org/id/ENSG00000188730) | von Willebrand factor C domain containing 2 [Source:HGNC Symbol;Acc:HGNC:30200] | 2,50 |
| VWCE | [ENSG00000167992](https://www.ensembl.org/id/ENSG00000167992) | von Willebrand factor C and EGF domains [Source:HGNC Symbol;Acc:HGNC:26487] | -1,59 |
| WASHC1 | [ENSG00000181404](https://www.ensembl.org/id/ENSG00000181404) | WASH complex subunit 1 [Source:HGNC Symbol;Acc:HGNC:24361] | -1,28 |
| WDFY3-AS2 | [ENSG00000180769](https://www.ensembl.org/id/ENSG00000180769) | WDFY3 antisense RNA 2 [Source:HGNC Symbol;Acc:HGNC:21603] | 1,23 |
| WDR11-DT | [ENSG00000227165](https://www.ensembl.org/id/ENSG00000227165) | WDR11 divergent transcript [Source:HGNC Symbol;Acc:HGNC:27437] | 4,24 |
| WDR62 | [ENSG00000075702](https://www.ensembl.org/id/ENSG00000075702) | WD repeat domain 62 [Source:HGNC Symbol;Acc:HGNC:24502] | 2,42 |
| WFIKKN2 | [ENSG00000173714](https://www.ensembl.org/id/ENSG00000173714) | WAP, follistatin/kazal, immunoglobulin, kunitz and netrin domain containing 2 [Source:HGNC Symbol;Acc:HGNC:30916] | 2,38 |
| WHRN | [ENSG00000095397](https://www.ensembl.org/id/ENSG00000095397) | whirlin [Source:HGNC Symbol;Acc:HGNC:16361] | -1,82 |
| WIPF3 | [ENSG00000122574](https://www.ensembl.org/id/ENSG00000122574) | WAS/WASL interacting protein family member 3 [Source:HGNC Symbol;Acc:HGNC:22004] | 1,66 |
| WIPI1 | [ENSG00000070540](https://www.ensembl.org/id/ENSG00000070540) | WD repeat domain, phosphoinositide interacting 1 [Source:HGNC Symbol;Acc:HGNC:25471] | 1,36 |
| WNT9B | [ENSG00000158955](https://www.ensembl.org/id/ENSG00000158955) | Wnt family member 9B [Source:HGNC Symbol;Acc:HGNC:12779] | -3,99 |
| WSB2 | [ENSG00000176871](https://www.ensembl.org/id/ENSG00000176871) | WD repeat and SOCS box containing 2 [Source:HGNC Symbol;Acc:HGNC:19222] | 1,12 |
| WSCD1 | [ENSG00000179314](https://www.ensembl.org/id/ENSG00000179314) | WSC domain containing 1 [Source:HGNC Symbol;Acc:HGNC:29060] | 2,10 |
| WT1 | [ENSG00000184937](https://www.ensembl.org/id/ENSG00000184937) | WT1 transcription factor [Source:HGNC Symbol;Acc:HGNC:12796] | -9,19 |
| WT1-AS | [ENSG00000183242](https://www.ensembl.org/id/ENSG00000183242) | WT1 antisense RNA [Source:HGNC Symbol;Acc:HGNC:18135] | -3,89 |
| WWC1 | [ENSG00000113645](https://www.ensembl.org/id/ENSG00000113645) | WW and C2 domain containing 1 [Source:HGNC Symbol;Acc:HGNC:29435] | -3,39 |
| WWP1 | [ENSG00000123124](https://www.ensembl.org/id/ENSG00000123124) | WW domain containing E3 ubiquitin protein ligase 1 [Source:HGNC Symbol;Acc:HGNC:17004] | 1,22 |
| XDH | [ENSG00000158125](https://www.ensembl.org/id/ENSG00000158125) | xanthine dehydrogenase [Source:HGNC Symbol;Acc:HGNC:12805] | 4,97 |
| XIRP2 | [ENSG00000163092](https://www.ensembl.org/id/ENSG00000163092) | xin actin binding repeat containing 2 [Source:HGNC Symbol;Acc:HGNC:14303] | 3,47 |
| XIST | [ENSG00000229807](https://www.ensembl.org/id/ENSG00000229807) | X inactive specific transcript [Source:HGNC Symbol;Acc:HGNC:12810] | -9,38 |
| XPO4 | [ENSG00000132953](https://www.ensembl.org/id/ENSG00000132953) | exportin 4 [Source:HGNC Symbol;Acc:HGNC:17796] | 3,12 |
| ZDHHC11 | [ENSG00000188818](https://www.ensembl.org/id/ENSG00000188818) | zinc finger DHHC-type containing 11 [Source:HGNC Symbol;Acc:HGNC:19158] | -1,71 |
| ZDHHC2 | [ENSG00000104219](https://www.ensembl.org/id/ENSG00000104219) | zinc finger DHHC-type palmitoyltransferase 2 [Source:HGNC Symbol;Acc:HGNC:18469] | 1,11 |
| ZDHHC22 | [ENSG00000177108](https://www.ensembl.org/id/ENSG00000177108) | zinc finger DHHC-type palmitoyltransferase 22 [Source:HGNC Symbol;Acc:HGNC:20106] | 4,36 |
| ZFR2 | [ENSG00000105278](https://www.ensembl.org/id/ENSG00000105278) | zinc finger RNA binding protein 2 [Source:HGNC Symbol;Acc:HGNC:29189] | -3,23 |
| ZMYND10 | [ENSG00000004838](https://www.ensembl.org/id/ENSG00000004838) | zinc finger MYND-type containing 10 [Source:HGNC Symbol;Acc:HGNC:19412] | 1,93 |
| ZNF106 | [ENSG00000103994](https://www.ensembl.org/id/ENSG00000103994) | zinc finger protein 106 [Source:HGNC Symbol;Acc:HGNC:12886] | 2,51 |
| ZNF286A-TBC1D26 | [ENSG00000255104](https://www.ensembl.org/id/ENSG00000255104) | ZNF286A-TBC1D26 readthrough (NMD candidate) [Source:HGNC Symbol;Acc:HGNC:55384] | -6,02 |
| ZNF30-AS1 | [ENSG00000270876](https://www.ensembl.org/id/ENSG00000270876) | ZNF30 antisense RNA 1 [Source:HGNC Symbol;Acc:HGNC:51179] | 3,07 |
| ZNF337 | [ENSG00000130684](https://www.ensembl.org/id/ENSG00000130684) | zinc finger protein 337 [Source:HGNC Symbol;Acc:HGNC:15809] | -0,92 |
| ZNF385B | [ENSG00000144331](https://www.ensembl.org/id/ENSG00000144331) | zinc finger protein 385B [Source:HGNC Symbol;Acc:HGNC:26332] | 2,32 |
| ZNF503-AS1 | [ENSG00000226051](https://www.ensembl.org/id/ENSG00000226051) | ZNF503 antisense RNA 1 [Source:HGNC Symbol;Acc:HGNC:27370] | 2,76 |
| ZNF556 | [ENSG00000172000](https://www.ensembl.org/id/ENSG00000172000) | zinc finger protein 556 [Source:HGNC Symbol;Acc:HGNC:25669] | 3,66 |
| ZNF710 | [ENSG00000140548](https://www.ensembl.org/id/ENSG00000140548) | zinc finger protein 710 [Source:HGNC Symbol;Acc:HGNC:25352] | 1,73 |
| ZNF710-AS1 | [ENSG00000259291](https://www.ensembl.org/id/ENSG00000259291) | ZNF710 antisense RNA 1 [Source:HGNC Symbol;Acc:HGNC:53141] | 1,98 |
| ZNF804A | [ENSG00000170396](https://www.ensembl.org/id/ENSG00000170396) | zinc finger protein 804A [Source:HGNC Symbol;Acc:HGNC:21711] | 2,96 |
| ZNF853 | [ENSG00000236609](https://www.ensembl.org/id/ENSG00000236609) | zinc finger protein 853 [Source:HGNC Symbol;Acc:HGNC:21767] | 1,26 |
| ZSCAN23 | [ENSG00000187987](https://www.ensembl.org/id/ENSG00000187987) | zinc finger and SCAN domain containing 23 [Source:HGNC Symbol;Acc:HGNC:21193] | 4,60 |
| ZYG11B | [ENSG00000162378](https://www.ensembl.org/id/ENSG00000162378) | zyg-11 family member B, cell cycle regulator [Source:HGNC Symbol;Acc:HGNC:25820] | 1,22 |
