# Supplementary material for: Role of inflammatory signaling pathways involving the CD40–CD40L–TRAF cascade in diabetes and hypertension—insights from animal and human studies
Source: Basic Res Cardiol. 2024 Mar 30;119(4):1–18. doi: 10.1007/s00395-024-01045-1 (PMC11319409; doi:10.1007/s00395-024-01045-1)
Supplement: Supplementary file 3 — Supplementary file3 (DOCX 218 KB) [file 395_2024_1045_MOESM3_ESM.docx]

| **Suppl. Table S4. RNA-Seq data: 1695 different expressed genes (DEG, p < 0.05) for the comparison CHD + HT + T2DM vs. CHD.** | | | |
| --- | --- | --- | --- |
| **Gene name** | **Identifier** | **Gene description** | **aHT-Dia vs. CAD - Log fold change** |
| AADAC | [ENSG00000114771](https://www.ensembl.org/id/ENSG00000114771) | arylacetamide deacetylase [Source:HGNC Symbol;Acc:HGNC:17] | -6,31 |
| ABCB11 | [ENSG00000073734](https://www.ensembl.org/id/ENSG00000073734) | ATP binding cassette subfamily B member 11 [Source:HGNC Symbol;Acc:HGNC:42] | -5,91 |
| ABCB5 | [ENSG00000004846](https://www.ensembl.org/id/ENSG00000004846) | ATP binding cassette subfamily B member 5 [Source:HGNC Symbol;Acc:HGNC:46] | -3,33 |
| ABCC12 | [ENSG00000140798](https://www.ensembl.org/id/ENSG00000140798) | ATP binding cassette subfamily C member 12 [Source:HGNC Symbol;Acc:HGNC:14640] | -5,57 |
| ABCC3 | [ENSG00000108846](https://www.ensembl.org/id/ENSG00000108846) | ATP binding cassette subfamily C member 3 [Source:HGNC Symbol;Acc:HGNC:54] | -2,13 |
| ABHD1 | [ENSG00000143994](https://www.ensembl.org/id/ENSG00000143994) | abhydrolase domain containing 1 [Source:HGNC Symbol;Acc:HGNC:17553] | -2,33 |
| ACACB | [ENSG00000076555](https://www.ensembl.org/id/ENSG00000076555) | acetyl-CoA carboxylase beta [Source:HGNC Symbol;Acc:HGNC:85] | -1,24 |
| ACHE | [ENSG00000087085](https://www.ensembl.org/id/ENSG00000087085) | acetylcholinesterase (Cartwright blood group) [Source:HGNC Symbol;Acc:HGNC:108] | -3,39 |
| ACKR4 | [ENSG00000129048](https://www.ensembl.org/id/ENSG00000129048) | atypical chemokine receptor 4 [Source:HGNC Symbol;Acc:HGNC:1611] | -1,79 |
| ACTA1 | [ENSG00000143632](https://www.ensembl.org/id/ENSG00000143632) | actin alpha 1, skeletal muscle [Source:HGNC Symbol;Acc:HGNC:129] | -6,86 |
| ACTN2 | [ENSG00000077522](https://www.ensembl.org/id/ENSG00000077522) | actinin alpha 2 [Source:HGNC Symbol;Acc:HGNC:164] | -2,92 |
| ACTN3 | [ENSG00000248746](https://www.ensembl.org/id/ENSG00000248746) | actinin alpha 3 [Source:HGNC Symbol;Acc:HGNC:165] | -8,14 |
| ADAM23 | [ENSG00000114948](https://www.ensembl.org/id/ENSG00000114948) | ADAM metallopeptidase domain 23 [Source:HGNC Symbol;Acc:HGNC:202] | -1,74 |
| ADAM8 | [ENSG00000151651](https://www.ensembl.org/id/ENSG00000151651) | ADAM metallopeptidase domain 8 [Source:HGNC Symbol;Acc:HGNC:215] | 2,38 |
| ADAMTS15 | [ENSG00000166106](https://www.ensembl.org/id/ENSG00000166106) | ADAM metallopeptidase with thrombospondin type 1 motif 15 [Source:HGNC Symbol;Acc:HGNC:16305] | 1,67 |
| ADAMTS16-DT | [ENSG00000250579](https://www.ensembl.org/id/ENSG00000250579) | ADAMTS16 divergent transcript [Source:HGNC Symbol;Acc:HGNC:55548] | -3,59 |
| ADAMTS17 | [ENSG00000140470](https://www.ensembl.org/id/ENSG00000140470) | ADAM metallopeptidase with thrombospondin type 1 motif 17 [Source:HGNC Symbol;Acc:HGNC:17109] | -2,00 |
| ADAMTS18 | [ENSG00000140873](https://www.ensembl.org/id/ENSG00000140873) | ADAM metallopeptidase with thrombospondin type 1 motif 18 [Source:HGNC Symbol;Acc:HGNC:17110] | -2,72 |
| ADAMTS7 | [ENSG00000136378](https://www.ensembl.org/id/ENSG00000136378) | ADAM metallopeptidase with thrombospondin type 1 motif 7 [Source:HGNC Symbol;Acc:HGNC:223] | -1,64 |
| ADCY2 | [ENSG00000078295](https://www.ensembl.org/id/ENSG00000078295) | adenylate cyclase 2 [Source:HGNC Symbol;Acc:HGNC:233] | -2,55 |
| ADCYAP1 | [ENSG00000141433](https://www.ensembl.org/id/ENSG00000141433) | adenylate cyclase activating polypeptide 1 [Source:HGNC Symbol;Acc:HGNC:241] | -3,43 |
| ADGRE2 | [ENSG00000127507](https://www.ensembl.org/id/ENSG00000127507) | adhesion G protein-coupled receptor E2 [Source:HGNC Symbol;Acc:HGNC:3337] | 3,45 |
| ADGRE3 | [ENSG00000131355](https://www.ensembl.org/id/ENSG00000131355) | adhesion G protein-coupled receptor E3 [Source:HGNC Symbol;Acc:HGNC:23647] | 6,56 |
| ADGRG3 | [ENSG00000182885](https://www.ensembl.org/id/ENSG00000182885) | adhesion G protein-coupled receptor G3 [Source:HGNC Symbol;Acc:HGNC:13728] | 4,65 |
| ADGRG7 | [ENSG00000144820](https://www.ensembl.org/id/ENSG00000144820) | adhesion G protein-coupled receptor G7 [Source:HGNC Symbol;Acc:HGNC:19241] | -10,79 |
| ADH1C | [ENSG00000248144](https://www.ensembl.org/id/ENSG00000248144) | alcohol dehydrogenase 1C (class I), gamma polypeptide [Source:HGNC Symbol;Acc:HGNC:251] | -1,86 |
| ADIPOQ | [ENSG00000181092](https://www.ensembl.org/id/ENSG00000181092) | adiponectin, C1Q and collagen domain containing [Source:HGNC Symbol;Acc:HGNC:13633] | -2,66 |
| ADIPOQ-AS1 | [ENSG00000226482](https://www.ensembl.org/id/ENSG00000226482) | ADIPOQ antisense RNA 1 [Source:HGNC Symbol;Acc:HGNC:40648] | -4,66 |
| ADORA1 | [ENSG00000163485](https://www.ensembl.org/id/ENSG00000163485) | adenosine A1 receptor [Source:HGNC Symbol;Acc:HGNC:262] | -4,05 |
| ADRB1 | [ENSG00000043591](https://www.ensembl.org/id/ENSG00000043591) | adrenoceptor beta 1 [Source:HGNC Symbol;Acc:HGNC:285] | -2,81 |
| AFF3 | [ENSG00000144218](https://www.ensembl.org/id/ENSG00000144218) | ALF transcription elongation factor 3 [Source:HGNC Symbol;Acc:HGNC:6473] | 1,08 |
| AGBL1 | [ENSG00000273540](https://www.ensembl.org/id/ENSG00000273540) | AGBL carboxypeptidase 1 [Source:HGNC Symbol;Acc:HGNC:26504] | -6,68 |
| AGMAT | [ENSG00000116771](https://www.ensembl.org/id/ENSG00000116771) | agmatinase [Source:HGNC Symbol;Acc:HGNC:18407] | -6,42 |
| AGPAT2 | [ENSG00000169692](https://www.ensembl.org/id/ENSG00000169692) | 1-acylglycerol-3-phosphate O-acyltransferase 2 [Source:HGNC Symbol;Acc:HGNC:325] | -1,25 |
| AHSP | [ENSG00000169877](https://www.ensembl.org/id/ENSG00000169877) | alpha hemoglobin stabilizing protein [Source:HGNC Symbol;Acc:HGNC:18075] | 3,39 |
| AKR1B15 | [ENSG00000227471](https://www.ensembl.org/id/ENSG00000227471) | aldo-keto reductase family 1 member B15 [Source:HGNC Symbol;Acc:HGNC:37281] | -4,64 |
| AKR1C1 | [ENSG00000187134](https://www.ensembl.org/id/ENSG00000187134) | aldo-keto reductase family 1 member C1 [Source:HGNC Symbol;Acc:HGNC:384] | -1,32 |
| AKR1C2 | [ENSG00000151632](https://www.ensembl.org/id/ENSG00000151632) | aldo-keto reductase family 1 member C2 [Source:HGNC Symbol;Acc:HGNC:385] | -2,06 |
| AKR1C3 | [ENSG00000196139](https://www.ensembl.org/id/ENSG00000196139) | aldo-keto reductase family 1 member C3 [Source:HGNC Symbol;Acc:HGNC:386] | -1,34 |
| ALAS2 | [ENSG00000158578](https://www.ensembl.org/id/ENSG00000158578) | 5'-aminolevulinate synthase 2 [Source:HGNC Symbol;Acc:HGNC:397] | 7,48 |
| ALDH1A2 | [ENSG00000128918](https://www.ensembl.org/id/ENSG00000128918) | aldehyde dehydrogenase 1 family member A2 [Source:HGNC Symbol;Acc:HGNC:15472] | -1,31 |
| ALDH1L1 | [ENSG00000144908](https://www.ensembl.org/id/ENSG00000144908) | aldehyde dehydrogenase 1 family member L1 [Source:HGNC Symbol;Acc:HGNC:3978] | -1,27 |
| ALKAL2 | [ENSG00000189292](https://www.ensembl.org/id/ENSG00000189292) | ALK and LTK ligand 2 [Source:HGNC Symbol;Acc:HGNC:27683] | 1,91 |
| ALOX15 | [ENSG00000161905](https://www.ensembl.org/id/ENSG00000161905) | arachidonate 15-lipoxygenase [Source:HGNC Symbol;Acc:HGNC:433] | -8,49 |
| ALOX5 | [ENSG00000012779](https://www.ensembl.org/id/ENSG00000012779) | arachidonate 5-lipoxygenase [Source:HGNC Symbol;Acc:HGNC:435] | 2,23 |
| ALOX5AP | [ENSG00000132965](https://www.ensembl.org/id/ENSG00000132965) | arachidonate 5-lipoxygenase activating protein [Source:HGNC Symbol;Acc:HGNC:436] | 2,17 |
| ALPK2 | [ENSG00000198796](https://www.ensembl.org/id/ENSG00000198796) | alpha kinase 2 [Source:HGNC Symbol;Acc:HGNC:20565] | -8,08 |
| AMER2 | [ENSG00000165566](https://www.ensembl.org/id/ENSG00000165566) | APC membrane recruitment protein 2 [Source:HGNC Symbol;Acc:HGNC:26360] | -6,15 |
| AMHR2 | [ENSG00000135409](https://www.ensembl.org/id/ENSG00000135409) | anti-Mullerian hormone receptor type 2 [Source:HGNC Symbol;Acc:HGNC:465] | -3,52 |
| AMIGO3 | [ENSG00000176020](https://www.ensembl.org/id/ENSG00000176020) | adhesion molecule with Ig like domain 3 [Source:HGNC Symbol;Acc:HGNC:24075] | -3,28 |
| AMOT | [ENSG00000126016](https://www.ensembl.org/id/ENSG00000126016) | angiomotin [Source:HGNC Symbol;Acc:HGNC:17810] | -1,50 |
| AMPD1 | [ENSG00000116748](https://www.ensembl.org/id/ENSG00000116748) | adenosine monophosphate deaminase 1 [Source:HGNC Symbol;Acc:HGNC:468] | -7,81 |
| ANGPT1 | [ENSG00000154188](https://www.ensembl.org/id/ENSG00000154188) | angiopoietin 1 [Source:HGNC Symbol;Acc:HGNC:484] | 1,13 |
| ANGPTL1 | [ENSG00000116194](https://www.ensembl.org/id/ENSG00000116194) | angiopoietin like 1 [Source:HGNC Symbol;Acc:HGNC:489] | 1,27 |
| ANGPTL7 | [ENSG00000171819](https://www.ensembl.org/id/ENSG00000171819) | angiopoietin like 7 [Source:HGNC Symbol;Acc:HGNC:24078] | -2,07 |
| ANK1 | [ENSG00000029534](https://www.ensembl.org/id/ENSG00000029534) | ankyrin 1 [Source:HGNC Symbol;Acc:HGNC:492] | -2,71 |
| ANKDD1B | [ENSG00000189045](https://www.ensembl.org/id/ENSG00000189045) | ankyrin repeat and death domain containing 1B [Source:HGNC Symbol;Acc:HGNC:32525] | -2,57 |
| ANKRD1 | [ENSG00000148677](https://www.ensembl.org/id/ENSG00000148677) | ankyrin repeat domain 1 [Source:HGNC Symbol;Acc:HGNC:15819] | -7,37 |
| ANKRD2 | [ENSG00000165887](https://www.ensembl.org/id/ENSG00000165887) | ankyrin repeat domain 2 [Source:HGNC Symbol;Acc:HGNC:495] | -8,54 |
| ANKRD23 | [ENSG00000163126](https://www.ensembl.org/id/ENSG00000163126) | ankyrin repeat domain 23 [Source:HGNC Symbol;Acc:HGNC:24470] | -6,76 |
| ANKRD44-AS1 | [ENSG00000231621](https://www.ensembl.org/id/ENSG00000231621) | ANKRD44 antisense RNA 1 [Source:HGNC Symbol;Acc:HGNC:41090] | 5,40 |
| ANO5 | [ENSG00000171714](https://www.ensembl.org/id/ENSG00000171714) | anoctamin 5 [Source:HGNC Symbol;Acc:HGNC:27337] | -1,34 |
| ANOS1 | [ENSG00000011201](https://www.ensembl.org/id/ENSG00000011201) | anosmin 1 [Source:HGNC Symbol;Acc:HGNC:6211] | -1,84 |
| ANXA13 | [ENSG00000104537](https://www.ensembl.org/id/ENSG00000104537) | annexin A13 [Source:HGNC Symbol;Acc:HGNC:536] | -6,53 |
| ANXA8 | [ENSG00000265190](https://www.ensembl.org/id/ENSG00000265190) | annexin A8 [Source:HGNC Symbol;Acc:HGNC:546] | -6,61 |
| ANXA8L1 | [ENSG00000264230](https://www.ensembl.org/id/ENSG00000264230) | annexin A8 like 1 [Source:HGNC Symbol;Acc:HGNC:23334] | -7,50 |
| AP1M2 | [ENSG00000129354](https://www.ensembl.org/id/ENSG00000129354) | adaptor related protein complex 1 subunit mu 2 [Source:HGNC Symbol;Acc:HGNC:558] | -3,03 |
| APCDD1 | [ENSG00000154856](https://www.ensembl.org/id/ENSG00000154856) | APC down-regulated 1 [Source:HGNC Symbol;Acc:HGNC:15718] | -2,06 |
| APCDD1L | [ENSG00000198768](https://www.ensembl.org/id/ENSG00000198768) | APC down-regulated 1 like [Source:HGNC Symbol;Acc:HGNC:26892] | -2,01 |
| APOBEC2 | [ENSG00000124701](https://www.ensembl.org/id/ENSG00000124701) | apolipoprotein B mRNA editing enzyme catalytic subunit 2 [Source:HGNC Symbol;Acc:HGNC:605] | -7,87 |
| APOBEC3A | [ENSG00000128383](https://www.ensembl.org/id/ENSG00000128383) | apolipoprotein B mRNA editing enzyme catalytic subunit 3A [Source:HGNC Symbol;Acc:HGNC:17343] | 4,69 |
| APOD | [ENSG00000189058](https://www.ensembl.org/id/ENSG00000189058) | apolipoprotein D [Source:HGNC Symbol;Acc:HGNC:612] | -1,44 |
| APOL6 | [ENSG00000221963](https://www.ensembl.org/id/ENSG00000221963) | apolipoprotein L6 [Source:HGNC Symbol;Acc:HGNC:14870] | -1,08 |
| AQP4 | [ENSG00000171885](https://www.ensembl.org/id/ENSG00000171885) | aquaporin 4 [Source:HGNC Symbol;Acc:HGNC:637] | -6,15 |
| AQP7 | [ENSG00000165269](https://www.ensembl.org/id/ENSG00000165269) | aquaporin 7 [Source:HGNC Symbol;Acc:HGNC:640] | -2,02 |
| AQP7B | [ENSG00000259916](https://www.ensembl.org/id/ENSG00000259916) | aquaporin 7B [Source:HGNC Symbol;Acc:HGNC:53895] | -2,95 |
| ARHGAP11B | [ENSG00000285077](https://www.ensembl.org/id/ENSG00000285077) | Rho GTPase activating protein 11B [Source:HGNC Symbol;Acc:HGNC:15782] | -3,60 |
| ARHGAP18 | [ENSG00000146376](https://www.ensembl.org/id/ENSG00000146376) | Rho GTPase activating protein 18 [Source:HGNC Symbol;Acc:HGNC:21035] | -1,53 |
| ARHGAP28-AS1 | [ENSG00000266441](https://www.ensembl.org/id/ENSG00000266441) | ARHGAP28 antisense RNA 1 [Source:HGNC Symbol;Acc:HGNC:55320] | -4,76 |
| ARHGAP29-AS1 | [ENSG00000226835](https://www.ensembl.org/id/ENSG00000226835) | ARHGAP29 antisense RNA 1 [Source:HGNC Symbol;Acc:HGNC:54311] | -3,78 |
| ARHGAP9 | [ENSG00000123329](https://www.ensembl.org/id/ENSG00000123329) | Rho GTPase activating protein 9 [Source:HGNC Symbol;Acc:HGNC:14130] | 2,01 |
| ARHGDIG | [ENSG00000242173](https://www.ensembl.org/id/ENSG00000242173) | Rho GDP dissociation inhibitor gamma [Source:HGNC Symbol;Acc:HGNC:680] | -2,20 |
| ARHGEF35 | [ENSG00000213214](https://www.ensembl.org/id/ENSG00000213214) | Rho guanine nucleotide exchange factor 35 [Source:HGNC Symbol;Acc:HGNC:33846] | -2,97 |
| ARHGEF35-AS1 | [ENSG00000244198](https://www.ensembl.org/id/ENSG00000244198) | ARHGEF35 antisense RNA 1 [Source:HGNC Symbol;Acc:HGNC:41292] | -1,67 |
| ARHGEF5 | [ENSG00000050327](https://www.ensembl.org/id/ENSG00000050327) | Rho guanine nucleotide exchange factor 5 [Source:HGNC Symbol;Acc:HGNC:13209] | -1,63 |
| ARL2-SNX15 | [ENSG00000273003](https://www.ensembl.org/id/ENSG00000273003) | ARL2-SNX15 readthrough (NMD candidate) [Source:HGNC Symbol;Acc:HGNC:49197] | 5,50 |
| ARPP21 | [ENSG00000172995](https://www.ensembl.org/id/ENSG00000172995) | cAMP regulated phosphoprotein 21 [Source:HGNC Symbol;Acc:HGNC:16968] | -5,79 |
| ARRDC5 | [ENSG00000205784](https://www.ensembl.org/id/ENSG00000205784) | arrestin domain containing 5 [Source:HGNC Symbol;Acc:HGNC:31407] | 3,69 |
| ARSL | [ENSG00000157399](https://www.ensembl.org/id/ENSG00000157399) | arylsulfatase L [Source:HGNC Symbol;Acc:HGNC:719] | -3,14 |
| ART1 | [ENSG00000129744](https://www.ensembl.org/id/ENSG00000129744) | ADP-ribosyltransferase 1 [Source:HGNC Symbol;Acc:HGNC:723] | -8,32 |
| ART5 | [ENSG00000167311](https://www.ensembl.org/id/ENSG00000167311) | ADP-ribosyltransferase 5 [Source:HGNC Symbol;Acc:HGNC:24049] | -4,05 |
| ARX | [ENSG00000004848](https://www.ensembl.org/id/ENSG00000004848) | aristaless related homeobox [Source:HGNC Symbol;Acc:HGNC:18060] | -6,99 |
| ASB10 | [ENSG00000146926](https://www.ensembl.org/id/ENSG00000146926) | ankyrin repeat and SOCS box containing 10 [Source:HGNC Symbol;Acc:HGNC:17185] | -4,75 |
| ASB11 | [ENSG00000165192](https://www.ensembl.org/id/ENSG00000165192) | ankyrin repeat and SOCS box containing 11 [Source:HGNC Symbol;Acc:HGNC:17186] | -5,07 |
| ASB12 | [ENSG00000198881](https://www.ensembl.org/id/ENSG00000198881) | ankyrin repeat and SOCS box containing 12 [Source:HGNC Symbol;Acc:HGNC:19763] | -2,97 |
| ASB14 | [ENSG00000239388](https://www.ensembl.org/id/ENSG00000239388) | ankyrin repeat and SOCS box containing 14 [Source:HGNC Symbol;Acc:HGNC:19766] | -2,01 |
| ASB15 | [ENSG00000146809](https://www.ensembl.org/id/ENSG00000146809) | ankyrin repeat and SOCS box containing 15 [Source:HGNC Symbol;Acc:HGNC:19767] | -7,04 |
| ASB18 | [ENSG00000182177](https://www.ensembl.org/id/ENSG00000182177) | ankyrin repeat and SOCS box containing 18 [Source:HGNC Symbol;Acc:HGNC:19770] | -7,93 |
| ASB4 | [ENSG00000005981](https://www.ensembl.org/id/ENSG00000005981) | ankyrin repeat and SOCS box containing 4 [Source:HGNC Symbol;Acc:HGNC:16009] | -10,25 |
| ASB5 | [ENSG00000164122](https://www.ensembl.org/id/ENSG00000164122) | ankyrin repeat and SOCS box containing 5 [Source:HGNC Symbol;Acc:HGNC:17180] | -7,63 |
| ASCL5 | [ENSG00000232237](https://www.ensembl.org/id/ENSG00000232237) | achaete-scute family bHLH transcription factor 5 [Source:HGNC Symbol;Acc:HGNC:33169] | -5,08 |
| ASPHD1 | [ENSG00000174939](https://www.ensembl.org/id/ENSG00000174939) | aspartate beta-hydroxylase domain containing 1 [Source:HGNC Symbol;Acc:HGNC:27380] | -4,60 |
| ATP1B1 | [ENSG00000143153](https://www.ensembl.org/id/ENSG00000143153) | ATPase Na+/K+ transporting subunit beta 1 [Source:HGNC Symbol;Acc:HGNC:804] | -2,72 |
| ATP1B4 | [ENSG00000101892](https://www.ensembl.org/id/ENSG00000101892) | ATPase Na+/K+ transporting family member beta 4 [Source:HGNC Symbol;Acc:HGNC:808] | -6,28 |
| ATP2A1 | [ENSG00000196296](https://www.ensembl.org/id/ENSG00000196296) | ATPase sarcoplasmic/endoplasmic reticulum Ca2+ transporting 1 [Source:HGNC Symbol;Acc:HGNC:811] | -8,35 |
| ATP2A1-AS1 | [ENSG00000260442](https://www.ensembl.org/id/ENSG00000260442) | ATP2A1 antisense RNA 1 [Source:HGNC Symbol;Acc:HGNC:51370] | -3,22 |
| ATP2B2 | [ENSG00000157087](https://www.ensembl.org/id/ENSG00000157087) | ATPase plasma membrane Ca2+ transporting 2 [Source:HGNC Symbol;Acc:HGNC:815] | -4,29 |
| ATP5MF-PTCD1 | [ENSG00000248919](https://www.ensembl.org/id/ENSG00000248919) | ATP5MF-PTCD1 readthrough [Source:HGNC Symbol;Acc:HGNC:38844] | 8,02 |
| ATRNL1 | [ENSG00000107518](https://www.ensembl.org/id/ENSG00000107518) | attractin like 1 [Source:HGNC Symbol;Acc:HGNC:29063] | -2,04 |
| ATXN2-AS | [ENSG00000258099](https://www.ensembl.org/id/ENSG00000258099) | ATXN2 antisense RNA [Source:HGNC Symbol;Acc:HGNC:51838] | -4,99 |
| AURKA | [ENSG00000087586](https://www.ensembl.org/id/ENSG00000087586) | aurora kinase A [Source:HGNC Symbol;Acc:HGNC:11393] | -2,34 |
| AVPI1 | [ENSG00000119986](https://www.ensembl.org/id/ENSG00000119986) | arginine vasopressin induced 1 [Source:HGNC Symbol;Acc:HGNC:30898] | 1,06 |
| AVPR1A | [ENSG00000166148](https://www.ensembl.org/id/ENSG00000166148) | arginine vasopressin receptor 1A [Source:HGNC Symbol;Acc:HGNC:895] | 2,36 |
| B3GNT7 | [ENSG00000156966](https://www.ensembl.org/id/ENSG00000156966) | UDP-GlcNAc:betaGal beta-1,3-N-acetylglucosaminyltransferase 7 [Source:HGNC Symbol;Acc:HGNC:18811] | -1,88 |
| B4GALT6 | [ENSG00000118276](https://www.ensembl.org/id/ENSG00000118276) | beta-1,4-galactosyltransferase 6 [Source:HGNC Symbol;Acc:HGNC:929] | -1,68 |
| BARX2 | [ENSG00000043039](https://www.ensembl.org/id/ENSG00000043039) | BARX homeobox 2 [Source:HGNC Symbol;Acc:HGNC:956] | -6,89 |
| BASP1 | [ENSG00000176788](https://www.ensembl.org/id/ENSG00000176788) | brain abundant membrane attached signal protein 1 [Source:HGNC Symbol;Acc:HGNC:957] | 1,45 |
| BCAS1 | [ENSG00000064787](https://www.ensembl.org/id/ENSG00000064787) | brain enriched myelin associated protein 1 [Source:HGNC Symbol;Acc:HGNC:974] | -2,68 |
| BCL2A1 | [ENSG00000140379](https://www.ensembl.org/id/ENSG00000140379) | BCL2 related protein A1 [Source:HGNC Symbol;Acc:HGNC:991] | 4,43 |
| BCO1 | [ENSG00000135697](https://www.ensembl.org/id/ENSG00000135697) | beta-carotene oxygenase 1 [Source:HGNC Symbol;Acc:HGNC:13815] | -4,67 |
| BDKRB1 | [ENSG00000100739](https://www.ensembl.org/id/ENSG00000100739) | bradykinin receptor B1 [Source:HGNC Symbol;Acc:HGNC:1029] | -3,80 |
| BEST3 | [ENSG00000127325](https://www.ensembl.org/id/ENSG00000127325) | bestrophin 3 [Source:HGNC Symbol;Acc:HGNC:17105] | -5,14 |
| BEST4 | [ENSG00000142959](https://www.ensembl.org/id/ENSG00000142959) | bestrophin 4 [Source:HGNC Symbol;Acc:HGNC:17106] | -2,37 |
| BICDL1 | [ENSG00000135127](https://www.ensembl.org/id/ENSG00000135127) | BICD family like cargo adaptor 1 [Source:HGNC Symbol;Acc:HGNC:28095] | -2,56 |
| BIN1 | [ENSG00000136717](https://www.ensembl.org/id/ENSG00000136717) | bridging integrator 1 [Source:HGNC Symbol;Acc:HGNC:1052] | -2,70 |
| BIRC7 | [ENSG00000101197](https://www.ensembl.org/id/ENSG00000101197) | baculoviral IAP repeat containing 7 [Source:HGNC Symbol;Acc:HGNC:13702] | -3,90 |
| BLNK | [ENSG00000095585](https://www.ensembl.org/id/ENSG00000095585) | B cell linker [Source:HGNC Symbol;Acc:HGNC:14211] | -2,17 |
| BMP3 | [ENSG00000152785](https://www.ensembl.org/id/ENSG00000152785) | bone morphogenetic protein 3 [Source:HGNC Symbol;Acc:HGNC:1070] | -3,78 |
| BMP7 | [ENSG00000101144](https://www.ensembl.org/id/ENSG00000101144) | bone morphogenetic protein 7 [Source:HGNC Symbol;Acc:HGNC:1074] | -3,60 |
| BNC1 | [ENSG00000169594](https://www.ensembl.org/id/ENSG00000169594) | basonuclin 1 [Source:HGNC Symbol;Acc:HGNC:1081] | -9,91 |
| BNIP3 | [ENSG00000176171](https://www.ensembl.org/id/ENSG00000176171) | BCL2 interacting protein 3 [Source:HGNC Symbol;Acc:HGNC:1084] | -1,41 |
| BOK | [ENSG00000176720](https://www.ensembl.org/id/ENSG00000176720) | BCL2 family apoptosis regulator BOK [Source:HGNC Symbol;Acc:HGNC:1087] | -1,70 |
| BORCS7-ASMT | [ENSG00000270316](https://www.ensembl.org/id/ENSG00000270316) | BORCS7-ASMT readthrough (NMD candidate) [Source:HGNC Symbol;Acc:HGNC:49183] | -8,77 |
| BTNL3 | [ENSG00000168903](https://www.ensembl.org/id/ENSG00000168903) | butyrophilin like 3 [Source:HGNC Symbol;Acc:HGNC:1143] | -5,10 |
| BTNL9 | [ENSG00000165810](https://www.ensembl.org/id/ENSG00000165810) | butyrophilin like 9 [Source:HGNC Symbol;Acc:HGNC:24176] | -1,49 |
| BUB1B | [ENSG00000156970](https://www.ensembl.org/id/ENSG00000156970) | BUB1 mitotic checkpoint serine/threonine kinase B [Source:HGNC Symbol;Acc:HGNC:1149] | -3,83 |
| BZW1-AS1 | [ENSG00000230408](https://www.ensembl.org/id/ENSG00000230408) | BZW1 antisense RNA 1 [Source:HGNC Symbol;Acc:HGNC:40839] | -4,74 |
| C10orf71 | [ENSG00000177354](https://www.ensembl.org/id/ENSG00000177354) | chromosome 10 open reading frame 71 [Source:HGNC Symbol;Acc:HGNC:26973] | -8,90 |
| C10orf71-AS1 | [ENSG00000236208](https://www.ensembl.org/id/ENSG00000236208) | C10orf71 antisense RNA 1 [Source:HGNC Symbol;Acc:HGNC:45007] | -7,66 |
| C10orf82 | [ENSG00000165863](https://www.ensembl.org/id/ENSG00000165863) | chromosome 10 open reading frame 82 [Source:HGNC Symbol;Acc:HGNC:28500] | -3,33 |
| C19orf33 | [ENSG00000167644](https://www.ensembl.org/id/ENSG00000167644) | chromosome 19 open reading frame 33 [Source:HGNC Symbol;Acc:HGNC:16668] | -2,52 |
| C19orf38 | [ENSG00000214212](https://www.ensembl.org/id/ENSG00000214212) | chromosome 19 open reading frame 38 [Source:HGNC Symbol;Acc:HGNC:34073] | 2,67 |
| C19orf81 | [ENSG00000235034](https://www.ensembl.org/id/ENSG00000235034) | chromosome 19 open reading frame 81 [Source:HGNC Symbol;Acc:HGNC:40041] | -5,05 |
| C1orf127 | [ENSG00000175262](https://www.ensembl.org/id/ENSG00000175262) | chromosome 1 open reading frame 127 [Source:HGNC Symbol;Acc:HGNC:26730] | -4,74 |
| C1orf220 | [ENSG00000213057](https://www.ensembl.org/id/ENSG00000213057) | chromosome 1 putative open reading frame 220 [Source:HGNC Symbol;Acc:HGNC:33805] | 2,72 |
| C1QL1 | [ENSG00000131094](https://www.ensembl.org/id/ENSG00000131094) | complement C1q like 1 [Source:HGNC Symbol;Acc:HGNC:24182] | -3,88 |
| C1QL4 | [ENSG00000186897](https://www.ensembl.org/id/ENSG00000186897) | complement C1q like 4 [Source:HGNC Symbol;Acc:HGNC:31416] | -3,67 |
| C1QTNF3-AMACR | [ENSG00000273294](https://www.ensembl.org/id/ENSG00000273294) | C1QTNF3-AMACR readthrough (NMD candidate) [Source:HGNC Symbol;Acc:HGNC:49198] | -3,17 |
| C20orf144 | [ENSG00000149609](https://www.ensembl.org/id/ENSG00000149609) | chromosome 20 open reading frame 144 [Source:HGNC Symbol;Acc:HGNC:16137] | 4,03 |
| C21orf62 | [ENSG00000205929](https://www.ensembl.org/id/ENSG00000205929) | chromosome 21 open reading frame 62 [Source:HGNC Symbol;Acc:HGNC:1305] | -3,09 |
| C22orf42 | [ENSG00000205856](https://www.ensembl.org/id/ENSG00000205856) | chromosome 22 open reading frame 42 [Source:HGNC Symbol;Acc:HGNC:27160] | -5,72 |
| C2CD4D | [ENSG00000225556](https://www.ensembl.org/id/ENSG00000225556) | C2 calcium dependent domain containing 4D [Source:HGNC Symbol;Acc:HGNC:37210] | -2,72 |
| C4orf54 | [ENSG00000248713](https://www.ensembl.org/id/ENSG00000248713) | chromosome 4 open reading frame 54 [Source:HGNC Symbol;Acc:HGNC:27741] | -8,37 |
| C5AR1 | [ENSG00000197405](https://www.ensembl.org/id/ENSG00000197405) | complement C5a receptor 1 [Source:HGNC Symbol;Acc:HGNC:1338] | 2,73 |
| C5AR2 | [ENSG00000134830](https://www.ensembl.org/id/ENSG00000134830) | complement C5a receptor 2 [Source:HGNC Symbol;Acc:HGNC:4527] | 3,48 |
| C5orf63 | [ENSG00000164241](https://www.ensembl.org/id/ENSG00000164241) | chromosome 5 open reading frame 63 [Source:HGNC Symbol;Acc:HGNC:40051] | -2,23 |
| C6orf47-AS1 | [ENSG00000227198](https://www.ensembl.org/id/ENSG00000227198) | C6orf47 antisense RNA 1 [Source:HGNC Symbol;Acc:HGNC:39767] | -5,91 |
| C7orf25 | [ENSG00000136197](https://www.ensembl.org/id/ENSG00000136197) | chromosome 7 open reading frame 25 [Source:HGNC Symbol;Acc:HGNC:21703] | 1,93 |
| CA1 | [ENSG00000133742](https://www.ensembl.org/id/ENSG00000133742) | carbonic anhydrase 1 [Source:HGNC Symbol;Acc:HGNC:1368] | 5,05 |
| CA14 | [ENSG00000118298](https://www.ensembl.org/id/ENSG00000118298) | carbonic anhydrase 14 [Source:HGNC Symbol;Acc:HGNC:1372] | -3,03 |
| CA3 | [ENSG00000164879](https://www.ensembl.org/id/ENSG00000164879) | carbonic anhydrase 3 [Source:HGNC Symbol;Acc:HGNC:1374] | -7,74 |
| CA9 | [ENSG00000107159](https://www.ensembl.org/id/ENSG00000107159) | carbonic anhydrase 9 [Source:HGNC Symbol;Acc:HGNC:1383] | -6,05 |
| CACNA1A | [ENSG00000141837](https://www.ensembl.org/id/ENSG00000141837) | calcium voltage-gated channel subunit alpha1 A [Source:HGNC Symbol;Acc:HGNC:1388] | 2,18 |
| CACNA1H | [ENSG00000196557](https://www.ensembl.org/id/ENSG00000196557) | calcium voltage-gated channel subunit alpha1 H [Source:HGNC Symbol;Acc:HGNC:1395] | 1,86 |
| CACNA1S | [ENSG00000081248](https://www.ensembl.org/id/ENSG00000081248) | calcium voltage-gated channel subunit alpha1 S [Source:HGNC Symbol;Acc:HGNC:1397] | -4,92 |
| CACNB1 | [ENSG00000067191](https://www.ensembl.org/id/ENSG00000067191) | calcium voltage-gated channel auxiliary subunit beta 1 [Source:HGNC Symbol;Acc:HGNC:1401] | -1,40 |
| CACNG1 | [ENSG00000108878](https://www.ensembl.org/id/ENSG00000108878) | calcium voltage-gated channel auxiliary subunit gamma 1 [Source:HGNC Symbol;Acc:HGNC:1405] | -6,75 |
| CACNG6 | [ENSG00000130433](https://www.ensembl.org/id/ENSG00000130433) | calcium voltage-gated channel auxiliary subunit gamma 6 [Source:HGNC Symbol;Acc:HGNC:13625] | -6,87 |
| CADM4 | [ENSG00000105767](https://www.ensembl.org/id/ENSG00000105767) | cell adhesion molecule 4 [Source:HGNC Symbol;Acc:HGNC:30825] | -1,62 |
| CALB2 | [ENSG00000172137](https://www.ensembl.org/id/ENSG00000172137) | calbindin 2 [Source:HGNC Symbol;Acc:HGNC:1435] | -3,03 |
| CALCA | [ENSG00000110680](https://www.ensembl.org/id/ENSG00000110680) | calcitonin related polypeptide alpha [Source:HGNC Symbol;Acc:HGNC:1437] | 2,37 |
| CALHM4 | [ENSG00000164451](https://www.ensembl.org/id/ENSG00000164451) | calcium homeostasis modulator family member 4 [Source:HGNC Symbol;Acc:HGNC:21094] | -3,29 |
| CALML6 | [ENSG00000169885](https://www.ensembl.org/id/ENSG00000169885) | calmodulin like 6 [Source:HGNC Symbol;Acc:HGNC:24193] | -4,69 |
| CALN1 | [ENSG00000183166](https://www.ensembl.org/id/ENSG00000183166) | calneuron 1 [Source:HGNC Symbol;Acc:HGNC:13248] | -3,39 |
| CAMK2A | [ENSG00000070808](https://www.ensembl.org/id/ENSG00000070808) | calcium/calmodulin dependent protein kinase II alpha [Source:HGNC Symbol;Acc:HGNC:1460] | -4,15 |
| CAMK2B | [ENSG00000058404](https://www.ensembl.org/id/ENSG00000058404) | calcium/calmodulin dependent protein kinase II beta [Source:HGNC Symbol;Acc:HGNC:1461] | -4,29 |
| CAPN3 | [ENSG00000092529](https://www.ensembl.org/id/ENSG00000092529) | calpain 3 [Source:HGNC Symbol;Acc:HGNC:1480] | -2,86 |
| CARD14 | [ENSG00000141527](https://www.ensembl.org/id/ENSG00000141527) | caspase recruitment domain family member 14 [Source:HGNC Symbol;Acc:HGNC:16446] | -2,19 |
| CARTPT | [ENSG00000164326](https://www.ensembl.org/id/ENSG00000164326) | CART prepropeptide [Source:HGNC Symbol;Acc:HGNC:24323] | -5,68 |
| CASQ1 | [ENSG00000143318](https://www.ensembl.org/id/ENSG00000143318) | calsequestrin 1 [Source:HGNC Symbol;Acc:HGNC:1512] | -7,06 |
| CASTOR2 | [ENSG00000274070](https://www.ensembl.org/id/ENSG00000274070) | cytosolic arginine sensor for mTORC1 subunit 2 [Source:HGNC Symbol;Acc:HGNC:37073] | -2,29 |
| CASTOR3 | [ENSG00000291122](https://www.ensembl.org/id/ENSG00000291122) | CASTOR family member 3, pseudogene [Source:HGNC Symbol;Acc:HGNC:29954] | -1,97 |
| CAV3 | [ENSG00000182533](https://www.ensembl.org/id/ENSG00000182533) | caveolin 3 [Source:HGNC Symbol;Acc:HGNC:1529] | -1,79 |
| CAVIN4 | [ENSG00000170681](https://www.ensembl.org/id/ENSG00000170681) | caveolae associated protein 4 [Source:HGNC Symbol;Acc:HGNC:33742] | -5,45 |
| CBS | [ENSG00000160200](https://www.ensembl.org/id/ENSG00000160200) | cystathionine beta-synthase [Source:HGNC Symbol;Acc:HGNC:1550] | -2,03 |
| CCDC15-DT | [ENSG00000285825](https://www.ensembl.org/id/ENSG00000285825) | CCDC15 divergent transcript [Source:HGNC Symbol;Acc:HGNC:54193] | -3,47 |
| CCDC28B | [ENSG00000160050](https://www.ensembl.org/id/ENSG00000160050) | coiled-coil domain containing 28B [Source:HGNC Symbol;Acc:HGNC:28163] | -1,52 |
| CCL20 | [ENSG00000115009](https://www.ensembl.org/id/ENSG00000115009) | C-C motif chemokine ligand 20 [Source:HGNC Symbol;Acc:HGNC:10619] | 5,13 |
| CCL24 | [ENSG00000106178](https://www.ensembl.org/id/ENSG00000106178) | C-C motif chemokine ligand 24 [Source:HGNC Symbol;Acc:HGNC:10623] | -3,03 |
| CCNA1 | [ENSG00000133101](https://www.ensembl.org/id/ENSG00000133101) | cyclin A1 [Source:HGNC Symbol;Acc:HGNC:1577] | -2,89 |
| CCR6 | [ENSG00000112486](https://www.ensembl.org/id/ENSG00000112486) | C-C motif chemokine receptor 6 [Source:HGNC Symbol;Acc:HGNC:1607] | 3,77 |
| CD177 | [ENSG00000204936](https://www.ensembl.org/id/ENSG00000204936) | CD177 molecule [Source:HGNC Symbol;Acc:HGNC:30072] | -2,67 |
| CD300A | [ENSG00000167851](https://www.ensembl.org/id/ENSG00000167851) | CD300a molecule [Source:HGNC Symbol;Acc:HGNC:19319] | 2,34 |
| CD300C | [ENSG00000167850](https://www.ensembl.org/id/ENSG00000167850) | CD300c molecule [Source:HGNC Symbol;Acc:HGNC:19320] | 4,48 |
| CD300E | [ENSG00000186407](https://www.ensembl.org/id/ENSG00000186407) | CD300e molecule [Source:HGNC Symbol;Acc:HGNC:28874] | 4,53 |
| CD300LG | [ENSG00000161649](https://www.ensembl.org/id/ENSG00000161649) | CD300 molecule like family member g [Source:HGNC Symbol;Acc:HGNC:30455] | -1,18 |
| CD53 | [ENSG00000143119](https://www.ensembl.org/id/ENSG00000143119) | CD53 molecule [Source:HGNC Symbol;Acc:HGNC:1686] | 2,06 |
| CDCP1 | [ENSG00000163814](https://www.ensembl.org/id/ENSG00000163814) | CUB domain containing protein 1 [Source:HGNC Symbol;Acc:HGNC:24357] | -2,61 |
| CDH15 | [ENSG00000129910](https://www.ensembl.org/id/ENSG00000129910) | cadherin 15 [Source:HGNC Symbol;Acc:HGNC:1754] | -5,36 |
| CDH2 | [ENSG00000170558](https://www.ensembl.org/id/ENSG00000170558) | cadherin 2 [Source:HGNC Symbol;Acc:HGNC:1759] | -1,89 |
| CDH22 | [ENSG00000149654](https://www.ensembl.org/id/ENSG00000149654) | cadherin 22 [Source:HGNC Symbol;Acc:HGNC:13251] | -3,72 |
| CDH6 | [ENSG00000113361](https://www.ensembl.org/id/ENSG00000113361) | cadherin 6 [Source:HGNC Symbol;Acc:HGNC:1765] | 1,52 |
| CDH8 | [ENSG00000150394](https://www.ensembl.org/id/ENSG00000150394) | cadherin 8 [Source:HGNC Symbol;Acc:HGNC:1767] | -3,23 |
| CDK18 | [ENSG00000117266](https://www.ensembl.org/id/ENSG00000117266) | cyclin dependent kinase 18 [Source:HGNC Symbol;Acc:HGNC:8751] | 1,04 |
| CDNF | [ENSG00000185267](https://www.ensembl.org/id/ENSG00000185267) | cerebral dopamine neurotrophic factor [Source:HGNC Symbol;Acc:HGNC:24913] | -1,89 |
| CDO1 | [ENSG00000129596](https://www.ensembl.org/id/ENSG00000129596) | cysteine dioxygenase type 1 [Source:HGNC Symbol;Acc:HGNC:1795] | -1,29 |
| CDR1 | [ENSG00000288642](https://www.ensembl.org/id/ENSG00000288642) | cerebellar degeneration related protein 1 [Source:HGNC Symbol;Acc:HGNC:1798] | -7,18 |
| CDRT7 | [ENSG00000259944](https://www.ensembl.org/id/ENSG00000259944) | CMT1A duplicated region transcript 7 [Source:HGNC Symbol;Acc:HGNC:14386] | -5,22 |
| CEACAM16 | [ENSG00000213892](https://www.ensembl.org/id/ENSG00000213892) | CEA cell adhesion molecule 16, tectorial membrane component [Source:HGNC Symbol;Acc:HGNC:31948] | -4,93 |
| CEACAM3 | [ENSG00000170956](https://www.ensembl.org/id/ENSG00000170956) | CEA cell adhesion molecule 3 [Source:HGNC Symbol;Acc:HGNC:1815] | 4,03 |
| CEACAM4 | [ENSG00000105352](https://www.ensembl.org/id/ENSG00000105352) | CEA cell adhesion molecule 4 [Source:HGNC Symbol;Acc:HGNC:1816] | 3,86 |
| CEBPA | [ENSG00000245848](https://www.ensembl.org/id/ENSG00000245848) | CCAAT enhancer binding protein alpha [Source:HGNC Symbol;Acc:HGNC:1833] | -1,63 |
| CECR2 | [ENSG00000099954](https://www.ensembl.org/id/ENSG00000099954) | CECR2 histone acetyl-lysine reader [Source:HGNC Symbol;Acc:HGNC:1840] | -3,39 |
| CEMIP2 | [ENSG00000135048](https://www.ensembl.org/id/ENSG00000135048) | cell migration inducing hyaluronidase 2 [Source:HGNC Symbol;Acc:HGNC:11869] | -1,26 |
| CENPS-CORT | [ENSG00000251503](https://www.ensembl.org/id/ENSG00000251503) | CENPS-CORT readthrough [Source:HGNC Symbol;Acc:HGNC:38843] | -4,07 |
| CEP85 | [ENSG00000130695](https://www.ensembl.org/id/ENSG00000130695) | centrosomal protein 85 [Source:HGNC Symbol;Acc:HGNC:25309] | -2,58 |
| CERS6-AS1 | [ENSG00000227617](https://www.ensembl.org/id/ENSG00000227617) | CERS6 antisense RNA 1 [Source:HGNC Symbol;Acc:HGNC:44485] | 1,85 |
| CES3 | [ENSG00000172828](https://www.ensembl.org/id/ENSG00000172828) | carboxylesterase 3 [Source:HGNC Symbol;Acc:HGNC:1865] | -3,40 |
| CFAP46 | [ENSG00000171811](https://www.ensembl.org/id/ENSG00000171811) | cilia and flagella associated protein 46 [Source:HGNC Symbol;Acc:HGNC:25247] | -5,32 |
| CFAP61 | [ENSG00000089101](https://www.ensembl.org/id/ENSG00000089101) | cilia and flagella associated protein 61 [Source:HGNC Symbol;Acc:HGNC:15872] | -5,28 |
| CFB | [ENSG00000243649](https://www.ensembl.org/id/ENSG00000243649) | complement factor B [Source:HGNC Symbol;Acc:HGNC:1037] | -1,48 |
| CFC1 | [ENSG00000136698](https://www.ensembl.org/id/ENSG00000136698) | cripto, FRL-1, cryptic family 1 [Source:HGNC Symbol;Acc:HGNC:18292] | -7,91 |
| CGB7 | [ENSG00000196337](https://www.ensembl.org/id/ENSG00000196337) | chorionic gonadotropin subunit beta 7 [Source:HGNC Symbol;Acc:HGNC:16451] | -4,26 |
| CGN | [ENSG00000143375](https://www.ensembl.org/id/ENSG00000143375) | cingulin [Source:HGNC Symbol;Acc:HGNC:17429] | -4,83 |
| CH25H | [ENSG00000138135](https://www.ensembl.org/id/ENSG00000138135) | cholesterol 25-hydroxylase [Source:HGNC Symbol;Acc:HGNC:1907] | 2,84 |
| CHIT1 | [ENSG00000133063](https://www.ensembl.org/id/ENSG00000133063) | chitinase 1 [Source:HGNC Symbol;Acc:HGNC:1936] | 3,22 |
| CHL1 | [ENSG00000134121](https://www.ensembl.org/id/ENSG00000134121) | cell adhesion molecule L1 like [Source:HGNC Symbol;Acc:HGNC:1939] | -1,99 |
| CHML | [ENSG00000203668](https://www.ensembl.org/id/ENSG00000203668) | CHM like Rab escort protein [Source:HGNC Symbol;Acc:HGNC:1941] | 0,94 |
| CHMP1B | [ENSG00000255112](https://www.ensembl.org/id/ENSG00000255112) | charged multivesicular body protein 1B [Source:HGNC Symbol;Acc:HGNC:24287] | 1,98 |
| CHRNA1 | [ENSG00000138435](https://www.ensembl.org/id/ENSG00000138435) | cholinergic receptor nicotinic alpha 1 subunit [Source:HGNC Symbol;Acc:HGNC:1955] | -4,99 |
| CHRNA4 | [ENSG00000101204](https://www.ensembl.org/id/ENSG00000101204) | cholinergic receptor nicotinic alpha 4 subunit [Source:HGNC Symbol;Acc:HGNC:1958] | -3,62 |
| CHRNA7 | [ENSG00000175344](https://www.ensembl.org/id/ENSG00000175344) | cholinergic receptor nicotinic alpha 7 subunit [Source:HGNC Symbol;Acc:HGNC:1960] | -1,90 |
| CHRNB1 | [ENSG00000170175](https://www.ensembl.org/id/ENSG00000170175) | cholinergic receptor nicotinic beta 1 subunit [Source:HGNC Symbol;Acc:HGNC:1961] | -1,56 |
| CHRND | [ENSG00000135902](https://www.ensembl.org/id/ENSG00000135902) | cholinergic receptor nicotinic delta subunit [Source:HGNC Symbol;Acc:HGNC:1965] | -11,30 |
| CHRNG | [ENSG00000196811](https://www.ensembl.org/id/ENSG00000196811) | cholinergic receptor nicotinic gamma subunit [Source:HGNC Symbol;Acc:HGNC:1967] | -6,10 |
| CHST1 | [ENSG00000175264](https://www.ensembl.org/id/ENSG00000175264) | carbohydrate sulfotransferase 1 [Source:HGNC Symbol;Acc:HGNC:1969] | -1,47 |
| CHST15 | [ENSG00000182022](https://www.ensembl.org/id/ENSG00000182022) | carbohydrate sulfotransferase 15 [Source:HGNC Symbol;Acc:HGNC:18137] | 1,05 |
| CHST5 | [ENSG00000135702](https://www.ensembl.org/id/ENSG00000135702) | carbohydrate sulfotransferase 5 [Source:HGNC Symbol;Acc:HGNC:1973] | 3,72 |
| CHTF8 | [ENSG00000168802](https://www.ensembl.org/id/ENSG00000168802) | chromosome transmission fidelity factor 8 [Source:HGNC Symbol;Acc:HGNC:24353] | 0,81 |
| CIDEA | [ENSG00000176194](https://www.ensembl.org/id/ENSG00000176194) | cell death inducing DFFA like effector a [Source:HGNC Symbol;Acc:HGNC:1976] | -2,89 |
| CIDEC | [ENSG00000187288](https://www.ensembl.org/id/ENSG00000187288) | cell death inducing DFFA like effector c [Source:HGNC Symbol;Acc:HGNC:24229] | -2,54 |
| CKM | [ENSG00000104879](https://www.ensembl.org/id/ENSG00000104879) | creatine kinase, M-type [Source:HGNC Symbol;Acc:HGNC:1994] | -9,57 |
| CKMT1A | [ENSG00000223572](https://www.ensembl.org/id/ENSG00000223572) | creatine kinase, mitochondrial 1A [Source:HGNC Symbol;Acc:HGNC:31736] | -4,29 |
| CKMT1B | [ENSG00000237289](https://www.ensembl.org/id/ENSG00000237289) | creatine kinase, mitochondrial 1B [Source:HGNC Symbol;Acc:HGNC:1995] | -4,24 |
| CLASP1-AS1 | [ENSG00000265451](https://www.ensembl.org/id/ENSG00000265451) | CLASP1 antisense RNA 1 [Source:HGNC Symbol;Acc:HGNC:55328] | -3,70 |
| CLC | [ENSG00000105205](https://www.ensembl.org/id/ENSG00000105205) | Charcot-Leyden crystal galectin [Source:HGNC Symbol;Acc:HGNC:2014] | 3,79 |
| CLCN1 | [ENSG00000188037](https://www.ensembl.org/id/ENSG00000188037) | chloride voltage-gated channel 1 [Source:HGNC Symbol;Acc:HGNC:2019] | -6,67 |
| CLCNKA | [ENSG00000186510](https://www.ensembl.org/id/ENSG00000186510) | chloride voltage-gated channel Ka [Source:HGNC Symbol;Acc:HGNC:2026] | -2,08 |
| CLDN1 | [ENSG00000163347](https://www.ensembl.org/id/ENSG00000163347) | claudin 1 [Source:HGNC Symbol;Acc:HGNC:2032] | -2,44 |
| CLDN15 | [ENSG00000106404](https://www.ensembl.org/id/ENSG00000106404) | claudin 15 [Source:HGNC Symbol;Acc:HGNC:2036] | -1,95 |
| CLDN19 | [ENSG00000164007](https://www.ensembl.org/id/ENSG00000164007) | claudin 19 [Source:HGNC Symbol;Acc:HGNC:2040] | -3,66 |
| CLEC18A | [ENSG00000157322](https://www.ensembl.org/id/ENSG00000157322) | C-type lectin domain family 18 member A [Source:HGNC Symbol;Acc:HGNC:30388] | -1,89 |
| CLEC1B | [ENSG00000165682](https://www.ensembl.org/id/ENSG00000165682) | C-type lectin domain family 1 member B [Source:HGNC Symbol;Acc:HGNC:24356] | 5,28 |
| CLEC4D | [ENSG00000166527](https://www.ensembl.org/id/ENSG00000166527) | C-type lectin domain family 4 member D [Source:HGNC Symbol;Acc:HGNC:14554] | 6,44 |
| CLEC4E | [ENSG00000166523](https://www.ensembl.org/id/ENSG00000166523) | C-type lectin domain family 4 member E [Source:HGNC Symbol;Acc:HGNC:14555] | 3,95 |
| CLEC4OP | [ENSG00000291059](https://www.ensembl.org/id/ENSG00000291059) | C-type lectin domain family 4 member O, pseudogene [Source:HGNC Symbol;Acc:HGNC:52651] | -3,55 |
| CLEC7A | [ENSG00000172243](https://www.ensembl.org/id/ENSG00000172243) | C-type lectin domain containing 7A [Source:HGNC Symbol;Acc:HGNC:14558] | 2,58 |
| CLIC3 | [ENSG00000169583](https://www.ensembl.org/id/ENSG00000169583) | chloride intracellular channel 3 [Source:HGNC Symbol;Acc:HGNC:2064] | -1,80 |
| CLIC5 | [ENSG00000112782](https://www.ensembl.org/id/ENSG00000112782) | chloride intracellular channel 5 [Source:HGNC Symbol;Acc:HGNC:13517] | -3,20 |
| CLIC6 | [ENSG00000159212](https://www.ensembl.org/id/ENSG00000159212) | chloride intracellular channel 6 [Source:HGNC Symbol;Acc:HGNC:2065] | -1,76 |
| CLMP | [ENSG00000166250](https://www.ensembl.org/id/ENSG00000166250) | CXADR like membrane protein [Source:HGNC Symbol;Acc:HGNC:24039] | -2,29 |
| CMTM2 | [ENSG00000140932](https://www.ensembl.org/id/ENSG00000140932) | CKLF like MARVEL transmembrane domain containing 2 [Source:HGNC Symbol;Acc:HGNC:19173] | 5,66 |
| CMYA5 | [ENSG00000164309](https://www.ensembl.org/id/ENSG00000164309) | cardiomyopathy associated 5 [Source:HGNC Symbol;Acc:HGNC:14305] | -7,82 |
| CNBD2 | [ENSG00000149646](https://www.ensembl.org/id/ENSG00000149646) | cyclic nucleotide binding domain containing 2 [Source:HGNC Symbol;Acc:HGNC:16145] | -2,71 |
| CNKSR1 | [ENSG00000142675](https://www.ensembl.org/id/ENSG00000142675) | connector enhancer of kinase suppressor of Ras 1 [Source:HGNC Symbol;Acc:HGNC:19700] | -2,65 |
| CNTFR-AS1 | [ENSG00000237159](https://www.ensembl.org/id/ENSG00000237159) | CNTFR antisense RNA 1 [Source:HGNC Symbol;Acc:HGNC:48712] | -3,94 |
| CNTNAP2 | [ENSG00000174469](https://www.ensembl.org/id/ENSG00000174469) | contactin associated protein 2 [Source:HGNC Symbol;Acc:HGNC:13830] | -4,71 |
| COBL | [ENSG00000106078](https://www.ensembl.org/id/ENSG00000106078) | cordon-bleu WH2 repeat protein [Source:HGNC Symbol;Acc:HGNC:22199] | -4,97 |
| COL21A1 | [ENSG00000124749](https://www.ensembl.org/id/ENSG00000124749) | collagen type XXI alpha 1 chain [Source:HGNC Symbol;Acc:HGNC:17025] | -1,28 |
| COL26A1 | [ENSG00000160963](https://www.ensembl.org/id/ENSG00000160963) | collagen type XXVI alpha 1 chain [Source:HGNC Symbol;Acc:HGNC:18038] | -3,99 |
| COL28A1 | [ENSG00000215018](https://www.ensembl.org/id/ENSG00000215018) | collagen type XXVIII alpha 1 chain [Source:HGNC Symbol;Acc:HGNC:22442] | -1,62 |
| COL2A1 | [ENSG00000139219](https://www.ensembl.org/id/ENSG00000139219) | collagen type II alpha 1 chain [Source:HGNC Symbol;Acc:HGNC:2200] | -6,63 |
| COL4A3 | [ENSG00000169031](https://www.ensembl.org/id/ENSG00000169031) | collagen type IV alpha 3 chain [Source:HGNC Symbol;Acc:HGNC:2204] | -2,38 |
| COL4A4 | [ENSG00000081052](https://www.ensembl.org/id/ENSG00000081052) | collagen type IV alpha 4 chain [Source:HGNC Symbol;Acc:HGNC:2206] | -2,38 |
| COL6A6 | [ENSG00000206384](https://www.ensembl.org/id/ENSG00000206384) | collagen type VI alpha 6 chain [Source:HGNC Symbol;Acc:HGNC:27023] | -4,35 |
| COL8A2 | [ENSG00000171812](https://www.ensembl.org/id/ENSG00000171812) | collagen type VIII alpha 2 chain [Source:HGNC Symbol;Acc:HGNC:2216] | 1,13 |
| COL9A2 | [ENSG00000049089](https://www.ensembl.org/id/ENSG00000049089) | collagen type IX alpha 2 chain [Source:HGNC Symbol;Acc:HGNC:2218] | -1,53 |
| COLEC10 | [ENSG00000184374](https://www.ensembl.org/id/ENSG00000184374) | collectin subfamily member 10 [Source:HGNC Symbol;Acc:HGNC:2220] | 3,29 |
| COLEC12 | [ENSG00000158270](https://www.ensembl.org/id/ENSG00000158270) | collectin subfamily member 12 [Source:HGNC Symbol;Acc:HGNC:16016] | -1,24 |
| COQ8A | [ENSG00000163050](https://www.ensembl.org/id/ENSG00000163050) | coenzyme Q8A [Source:HGNC Symbol;Acc:HGNC:16812] | -2,58 |
| CORO1A | [ENSG00000102879](https://www.ensembl.org/id/ENSG00000102879) | coronin 1A [Source:HGNC Symbol;Acc:HGNC:2252] | 1,88 |
| COX6A2 | [ENSG00000156885](https://www.ensembl.org/id/ENSG00000156885) | cytochrome c oxidase subunit 6A2 [Source:HGNC Symbol;Acc:HGNC:2279] | -8,22 |
| CPAMD8 | [ENSG00000160111](https://www.ensembl.org/id/ENSG00000160111) | C3 and PZP like alpha-2-macroglobulin domain containing 8 [Source:HGNC Symbol;Acc:HGNC:23228] | -1,35 |
| CPB1 | [ENSG00000153002](https://www.ensembl.org/id/ENSG00000153002) | carboxypeptidase B1 [Source:HGNC Symbol;Acc:HGNC:2299] | -3,24 |
| CPM | [ENSG00000135678](https://www.ensembl.org/id/ENSG00000135678) | carboxypeptidase M [Source:HGNC Symbol;Acc:HGNC:2311] | -1,97 |
| CPT1B | [ENSG00000205560](https://www.ensembl.org/id/ENSG00000205560) | carnitine palmitoyltransferase 1B [Source:HGNC Symbol;Acc:HGNC:2329] | -2,73 |
| CPXM1 | [ENSG00000088882](https://www.ensembl.org/id/ENSG00000088882) | carboxypeptidase X, M14 family member 1 [Source:HGNC Symbol;Acc:HGNC:15771] | -3,89 |
| CRB2 | [ENSG00000148204](https://www.ensembl.org/id/ENSG00000148204) | crumbs cell polarity complex component 2 [Source:HGNC Symbol;Acc:HGNC:18688] | -3,47 |
| CRISP3 | [ENSG00000096006](https://www.ensembl.org/id/ENSG00000096006) | cysteine rich secretory protein 3 [Source:HGNC Symbol;Acc:HGNC:16904] | 4,03 |
| CSF3R | [ENSG00000119535](https://www.ensembl.org/id/ENSG00000119535) | colony stimulating factor 3 receptor [Source:HGNC Symbol;Acc:HGNC:2439] | 4,63 |
| CSMD2 | [ENSG00000121904](https://www.ensembl.org/id/ENSG00000121904) | CUB and Sushi multiple domains 2 [Source:HGNC Symbol;Acc:HGNC:19290] | 2,80 |
| CSNK1G2-AS1 | [ENSG00000180846](https://www.ensembl.org/id/ENSG00000180846) | CSNK1G2 antisense RNA 1 [Source:HGNC Symbol;Acc:HGNC:28604] | 5,66 |
| CSRP3 | [ENSG00000129170](https://www.ensembl.org/id/ENSG00000129170) | cysteine and glycine rich protein 3 [Source:HGNC Symbol;Acc:HGNC:2472] | -9,26 |
| CTAG2 | [ENSG00000126890](https://www.ensembl.org/id/ENSG00000126890) | cancer/testis antigen 2 [Source:HGNC Symbol;Acc:HGNC:2492] | 8,88 |
| CTAGE8 | [ENSG00000289604](https://www.ensembl.org/id/ENSG00000289604) | CTAGE family member 8 [Source:HGNC Symbol;Acc:HGNC:37294] | -4,33 |
| CTAGE9 | [ENSG00000236761](https://www.ensembl.org/id/ENSG00000236761) | CTAGE family member 9 [Source:HGNC Symbol;Acc:HGNC:37275] | -5,84 |
| CTNNA2 | [ENSG00000066032](https://www.ensembl.org/id/ENSG00000066032) | catenin alpha 2 [Source:HGNC Symbol;Acc:HGNC:2510] | -6,08 |
| CTRB1 | [ENSG00000168925](https://www.ensembl.org/id/ENSG00000168925) | chymotrypsinogen B1 [Source:HGNC Symbol;Acc:HGNC:2521] | -2,99 |
| CTSG | [ENSG00000100448](https://www.ensembl.org/id/ENSG00000100448) | cathepsin G [Source:HGNC Symbol;Acc:HGNC:2532] | -2,36 |
| CTXN2-AS1 | [ENSG00000259235](https://www.ensembl.org/id/ENSG00000259235) | CTXN2 antisense RNA 1 [Source:HGNC Symbol;Acc:HGNC:55426] | -5,27 |
| CUTC | [ENSG00000119929](https://www.ensembl.org/id/ENSG00000119929) | cutC copper transporter [Source:HGNC Symbol;Acc:HGNC:24271] | -1,62 |
| CXADR | [ENSG00000154639](https://www.ensembl.org/id/ENSG00000154639) | CXADR Ig-like cell adhesion molecule [Source:HGNC Symbol;Acc:HGNC:2559] | -4,68 |
| CXCL11 | [ENSG00000169248](https://www.ensembl.org/id/ENSG00000169248) | C-X-C motif chemokine ligand 11 [Source:HGNC Symbol;Acc:HGNC:10638] | -2,91 |
| CXCL14 | [ENSG00000145824](https://www.ensembl.org/id/ENSG00000145824) | C-X-C motif chemokine ligand 14 [Source:HGNC Symbol;Acc:HGNC:10640] | -1,73 |
| CXCL5 | [ENSG00000163735](https://www.ensembl.org/id/ENSG00000163735) | C-X-C motif chemokine ligand 5 [Source:HGNC Symbol;Acc:HGNC:10642] | 2,56 |
| CXCL6 | [ENSG00000124875](https://www.ensembl.org/id/ENSG00000124875) | C-X-C motif chemokine ligand 6 [Source:HGNC Symbol;Acc:HGNC:10643] | -3,95 |
| CXCL8 | [ENSG00000169429](https://www.ensembl.org/id/ENSG00000169429) | C-X-C motif chemokine ligand 8 [Source:HGNC Symbol;Acc:HGNC:6025] | 4,11 |
| CXCL9 | [ENSG00000138755](https://www.ensembl.org/id/ENSG00000138755) | C-X-C motif chemokine ligand 9 [Source:HGNC Symbol;Acc:HGNC:7098] | -3,35 |
| CXCR1 | [ENSG00000163464](https://www.ensembl.org/id/ENSG00000163464) | C-X-C motif chemokine receptor 1 [Source:HGNC Symbol;Acc:HGNC:6026] | 5,72 |
| CXCR2 | [ENSG00000180871](https://www.ensembl.org/id/ENSG00000180871) | C-X-C motif chemokine receptor 2 [Source:HGNC Symbol;Acc:HGNC:6027] | 4,49 |
| CXCR4 | [ENSG00000121966](https://www.ensembl.org/id/ENSG00000121966) | C-X-C motif chemokine receptor 4 [Source:HGNC Symbol;Acc:HGNC:2561] | 2,86 |
| CYP11A1 | [ENSG00000140459](https://www.ensembl.org/id/ENSG00000140459) | cytochrome P450 family 11 subfamily A member 1 [Source:HGNC Symbol;Acc:HGNC:2590] | -3,34 |
| CYP26B1 | [ENSG00000003137](https://www.ensembl.org/id/ENSG00000003137) | cytochrome P450 family 26 subfamily B member 1 [Source:HGNC Symbol;Acc:HGNC:20581] | -1,50 |
| CYP2J2 | [ENSG00000134716](https://www.ensembl.org/id/ENSG00000134716) | cytochrome P450 family 2 subfamily J member 2 [Source:HGNC Symbol;Acc:HGNC:2634] | -5,66 |
| CYP3A4 | [ENSG00000160868](https://www.ensembl.org/id/ENSG00000160868) | cytochrome P450 family 3 subfamily A member 4 [Source:HGNC Symbol;Acc:HGNC:2637] | -4,95 |
| CYP4F3 | [ENSG00000186529](https://www.ensembl.org/id/ENSG00000186529) | cytochrome P450 family 4 subfamily F member 3 [Source:HGNC Symbol;Acc:HGNC:2646] | 3,75 |
| CYTIP | [ENSG00000115165](https://www.ensembl.org/id/ENSG00000115165) | cytohesin 1 interacting protein [Source:HGNC Symbol;Acc:HGNC:9506] | 2,83 |
| DAB1 | [ENSG00000173406](https://www.ensembl.org/id/ENSG00000173406) | DAB adaptor protein 1 [Source:HGNC Symbol;Acc:HGNC:2661] | 2,85 |
| DACH2 | [ENSG00000126733](https://www.ensembl.org/id/ENSG00000126733) | dachshund family transcription factor 2 [Source:HGNC Symbol;Acc:HGNC:16814] | 3,85 |
| DAND5 | [ENSG00000179284](https://www.ensembl.org/id/ENSG00000179284) | DAN domain BMP antagonist family member 5 [Source:HGNC Symbol;Acc:HGNC:26780] | 3,02 |
| DAPK2 | [ENSG00000035664](https://www.ensembl.org/id/ENSG00000035664) | death associated protein kinase 2 [Source:HGNC Symbol;Acc:HGNC:2675] | -2,23 |
| DAPL1 | [ENSG00000163331](https://www.ensembl.org/id/ENSG00000163331) | death associated protein like 1 [Source:HGNC Symbol;Acc:HGNC:21490] | -2,88 |
| DBNDD1 | [ENSG00000003249](https://www.ensembl.org/id/ENSG00000003249) | dysbindin domain containing 1 [Source:HGNC Symbol;Acc:HGNC:28455] | -3,87 |
| DCC | [ENSG00000187323](https://www.ensembl.org/id/ENSG00000187323) | DCC netrin 1 receptor [Source:HGNC Symbol;Acc:HGNC:2701] | -3,22 |
| DCDC2C | [ENSG00000214866](https://www.ensembl.org/id/ENSG00000214866) | doublecortin domain containing 2C [Source:HGNC Symbol;Acc:HGNC:32696] | -3,74 |
| DCHS2 | [ENSG00000197410](https://www.ensembl.org/id/ENSG00000197410) | dachsous cadherin-related 2 [Source:HGNC Symbol;Acc:HGNC:23111] | -3,54 |
| DDIT4L | [ENSG00000145358](https://www.ensembl.org/id/ENSG00000145358) | DNA damage inducible transcript 4 like [Source:HGNC Symbol;Acc:HGNC:30555] | -2,79 |
| DDN | [ENSG00000181418](https://www.ensembl.org/id/ENSG00000181418) | dendrin [Source:HGNC Symbol;Acc:HGNC:24458] | -6,23 |
| DDN-AS1 | [ENSG00000257913](https://www.ensembl.org/id/ENSG00000257913) | DDN and PRKAG1 antisense RNA 1 [Source:HGNC Symbol;Acc:HGNC:53464] | -2,66 |
| DDX25 | [ENSG00000109832](https://www.ensembl.org/id/ENSG00000109832) | DEAD-box helicase 25 [Source:HGNC Symbol;Acc:HGNC:18698] | -2,82 |
| DEFA1 | [ENSG00000206047](https://www.ensembl.org/id/ENSG00000206047) | defensin alpha 1 [Source:HGNC Symbol;Acc:HGNC:2761] | -8,09 |
| DEFB105A | [ENSG00000186562](https://www.ensembl.org/id/ENSG00000186562) | defensin beta 105A [Source:HGNC Symbol;Acc:HGNC:18087] | -4,94 |
| DENND2C | [ENSG00000175984](https://www.ensembl.org/id/ENSG00000175984) | DENN domain containing 2C [Source:HGNC Symbol;Acc:HGNC:24748] | -1,39 |
| DEPTOR | [ENSG00000155792](https://www.ensembl.org/id/ENSG00000155792) | DEP domain containing MTOR interacting protein [Source:HGNC Symbol;Acc:HGNC:22953] | -2,20 |
| DEPTOR-AS1 | [ENSG00000245330](https://www.ensembl.org/id/ENSG00000245330) | DEPTOR antisense RNA 1 [Source:HGNC Symbol;Acc:HGNC:55602] | -4,21 |
| DGCR6 | [ENSG00000183628](https://www.ensembl.org/id/ENSG00000183628) | DiGeorge syndrome critical region gene 6 [Source:HGNC Symbol;Acc:HGNC:2846] | -2,08 |
| DHRS7C | [ENSG00000184544](https://www.ensembl.org/id/ENSG00000184544) | dehydrogenase/reductase 7C [Source:HGNC Symbol;Acc:HGNC:32423] | -6,93 |
| DLGAP2 | [ENSG00000198010](https://www.ensembl.org/id/ENSG00000198010) | DLG associated protein 2 [Source:HGNC Symbol;Acc:HGNC:2906] | -5,81 |
| DLK1 | [ENSG00000185559](https://www.ensembl.org/id/ENSG00000185559) | delta like non-canonical Notch ligand 1 [Source:HGNC Symbol;Acc:HGNC:2907] | -6,55 |
| DLX6-AS1 | [ENSG00000231764](https://www.ensembl.org/id/ENSG00000231764) | DLX6 antisense RNA 1 [Source:HGNC Symbol;Acc:HGNC:37151] | 4,41 |
| DMKN | [ENSG00000161249](https://www.ensembl.org/id/ENSG00000161249) | dermokine [Source:HGNC Symbol;Acc:HGNC:25063] | -1,96 |
| DMP1 | [ENSG00000152592](https://www.ensembl.org/id/ENSG00000152592) | dentin matrix acidic phosphoprotein 1 [Source:HGNC Symbol;Acc:HGNC:2932] | -2,79 |
| DMRT2 | [ENSG00000173253](https://www.ensembl.org/id/ENSG00000173253) | doublesex and mab-3 related transcription factor 2 [Source:HGNC Symbol;Acc:HGNC:2935] | -2,57 |
| DNAJC22 | [ENSG00000178401](https://www.ensembl.org/id/ENSG00000178401) | DnaJ heat shock protein family (Hsp40) member C22 [Source:HGNC Symbol;Acc:HGNC:25802] | -4,29 |
| DNASE2B | [ENSG00000137976](https://www.ensembl.org/id/ENSG00000137976) | deoxyribonuclease 2 beta [Source:HGNC Symbol;Acc:HGNC:28875] | -5,24 |
| DNER | [ENSG00000187957](https://www.ensembl.org/id/ENSG00000187957) | delta/notch like EGF repeat containing [Source:HGNC Symbol;Acc:HGNC:24456] | -3,72 |
| DOK5 | [ENSG00000101134](https://www.ensembl.org/id/ENSG00000101134) | docking protein 5 [Source:HGNC Symbol;Acc:HGNC:16173] | -2,15 |
| DPP10 | [ENSG00000175497](https://www.ensembl.org/id/ENSG00000175497) | dipeptidyl peptidase like 10 [Source:HGNC Symbol;Acc:HGNC:20823] | -5,93 |
| DPT | [ENSG00000143196](https://www.ensembl.org/id/ENSG00000143196) | dermatopontin [Source:HGNC Symbol;Acc:HGNC:3011] | -1,44 |
| DPY19L2 | [ENSG00000177990](https://www.ensembl.org/id/ENSG00000177990) | dpy-19 like 2 [Source:HGNC Symbol;Acc:HGNC:19414] | 1,48 |
| DRD2 | [ENSG00000149295](https://www.ensembl.org/id/ENSG00000149295) | dopamine receptor D2 [Source:HGNC Symbol;Acc:HGNC:3023] | -2,82 |
| DRP2 | [ENSG00000102385](https://www.ensembl.org/id/ENSG00000102385) | dystrophin related protein 2 [Source:HGNC Symbol;Acc:HGNC:3032] | -1,94 |
| DSG2 | [ENSG00000046604](https://www.ensembl.org/id/ENSG00000046604) | desmoglein 2 [Source:HGNC Symbol;Acc:HGNC:3049] | -1,56 |
| DSG2-AS1 | [ENSG00000264859](https://www.ensembl.org/id/ENSG00000264859) | DSG2 antisense RNA 1 [Source:HGNC Symbol;Acc:HGNC:51311] | -4,75 |
| DUBR | [ENSG00000243701](https://www.ensembl.org/id/ENSG00000243701) | DPPA2 upstream binding RNA [Source:HGNC Symbol;Acc:HGNC:48569] | 1,15 |
| DUSP13 | [ENSG00000079393](https://www.ensembl.org/id/ENSG00000079393) | dual specificity phosphatase 13 [Source:HGNC Symbol;Acc:HGNC:19681] | -8,74 |
| DUSP29 | [ENSG00000188716](https://www.ensembl.org/id/ENSG00000188716) | dual specificity phosphatase 29 [Source:HGNC Symbol;Acc:HGNC:23481] | -10,96 |
| DUSP6 | [ENSG00000139318](https://www.ensembl.org/id/ENSG00000139318) | dual specificity phosphatase 6 [Source:HGNC Symbol;Acc:HGNC:3072] | -1,08 |
| DYNC1LI2-DT | [ENSG00000246777](https://www.ensembl.org/id/ENSG00000246777) | DYNC1LI2 divergent transcript [Source:HGNC Symbol;Acc:HGNC:52990] | -5,26 |
| DYRK1B | [ENSG00000105204](https://www.ensembl.org/id/ENSG00000105204) | dual specificity tyrosine phosphorylation regulated kinase 1B [Source:HGNC Symbol;Acc:HGNC:3092] | -1,68 |
| E2F8 | [ENSG00000129173](https://www.ensembl.org/id/ENSG00000129173) | E2F transcription factor 8 [Source:HGNC Symbol;Acc:HGNC:24727] | -4,70 |
| EDN3 | [ENSG00000124205](https://www.ensembl.org/id/ENSG00000124205) | endothelin 3 [Source:HGNC Symbol;Acc:HGNC:3178] | -3,38 |
| EEF1A2 | [ENSG00000101210](https://www.ensembl.org/id/ENSG00000101210) | eukaryotic translation elongation factor 1 alpha 2 [Source:HGNC Symbol;Acc:HGNC:3192] | -7,66 |
| EFNB3 | [ENSG00000108947](https://www.ensembl.org/id/ENSG00000108947) | ephrin B3 [Source:HGNC Symbol;Acc:HGNC:3228] | -6,26 |
| EGF | [ENSG00000138798](https://www.ensembl.org/id/ENSG00000138798) | epidermal growth factor [Source:HGNC Symbol;Acc:HGNC:3229] | -4,44 |
| EGR2 | [ENSG00000122877](https://www.ensembl.org/id/ENSG00000122877) | early growth response 2 [Source:HGNC Symbol;Acc:HGNC:3239] | -1,34 |
| EIF4A3 | [ENSG00000141543](https://www.ensembl.org/id/ENSG00000141543) | eukaryotic translation initiation factor 4A3 [Source:HGNC Symbol;Acc:HGNC:18683] | 2,00 |
| ENO3 | [ENSG00000108515](https://www.ensembl.org/id/ENSG00000108515) | enolase 3 [Source:HGNC Symbol;Acc:HGNC:3354] | -7,40 |
| ENTPD2 | [ENSG00000054179](https://www.ensembl.org/id/ENSG00000054179) | ectonucleoside triphosphate diphosphohydrolase 2 [Source:HGNC Symbol;Acc:HGNC:3364] | -2,26 |
| ENTPD6 | [ENSG00000197586](https://www.ensembl.org/id/ENSG00000197586) | ectonucleoside triphosphate diphosphohydrolase 6 [Source:HGNC Symbol;Acc:HGNC:3368] | -0,89 |
| EOLA1 | [ENSG00000197620](https://www.ensembl.org/id/ENSG00000197620) | endothelium and lymphocyte associated ASCH domain 1 [Source:HGNC Symbol;Acc:HGNC:28089] | 1,13 |
| EOLA2 | [ENSG00000197021](https://www.ensembl.org/id/ENSG00000197021) | endothelium and lymphocyte associated ASCH domain 2 [Source:HGNC Symbol;Acc:HGNC:17402] | 1,20 |
| EPB41L4B | [ENSG00000095203](https://www.ensembl.org/id/ENSG00000095203) | erythrocyte membrane protein band 4.1 like 4B [Source:HGNC Symbol;Acc:HGNC:19818] | -1,96 |
| EPN3 | [ENSG00000049283](https://www.ensembl.org/id/ENSG00000049283) | epsin 3 [Source:HGNC Symbol;Acc:HGNC:18235] | -6,33 |
| EPO | [ENSG00000130427](https://www.ensembl.org/id/ENSG00000130427) | erythropoietin [Source:HGNC Symbol;Acc:HGNC:3415] | -4,18 |
| EPPIN | [ENSG00000101448](https://www.ensembl.org/id/ENSG00000101448) | epididymal peptidase inhibitor [Source:HGNC Symbol;Acc:HGNC:15932] | -5,07 |
| EPS8L1 | [ENSG00000131037](https://www.ensembl.org/id/ENSG00000131037) | EPS8 like 1 [Source:HGNC Symbol;Acc:HGNC:21295] | -3,72 |
| ERICH5 | [ENSG00000177459](https://www.ensembl.org/id/ENSG00000177459) | glutamate rich 5 [Source:HGNC Symbol;Acc:HGNC:26823] | -5,39 |
| ESR1 | [ENSG00000091831](https://www.ensembl.org/id/ENSG00000091831) | estrogen receptor 1 [Source:HGNC Symbol;Acc:HGNC:3467] | 0,92 |
| ESRRG | [ENSG00000196482](https://www.ensembl.org/id/ENSG00000196482) | estrogen related receptor gamma [Source:HGNC Symbol;Acc:HGNC:3474] | -6,75 |
| EVPL | [ENSG00000167880](https://www.ensembl.org/id/ENSG00000167880) | envoplakin [Source:HGNC Symbol;Acc:HGNC:3503] | -3,66 |
| EXTL1 | [ENSG00000158008](https://www.ensembl.org/id/ENSG00000158008) | exostosin like glycosyltransferase 1 [Source:HGNC Symbol;Acc:HGNC:3515] | -3,94 |
| EYA1 | [ENSG00000104313](https://www.ensembl.org/id/ENSG00000104313) | EYA transcriptional coactivator and phosphatase 1 [Source:HGNC Symbol;Acc:HGNC:3519] | -2,76 |
| EYA4 | [ENSG00000112319](https://www.ensembl.org/id/ENSG00000112319) | EYA transcriptional coactivator and phosphatase 4 [Source:HGNC Symbol;Acc:HGNC:3522] | -2,02 |
| F8A3 | [ENSG00000277150](https://www.ensembl.org/id/ENSG00000277150) | coagulation factor VIII associated 3 [Source:HGNC Symbol;Acc:HGNC:31850] | -2,42 |
| FAAH | [ENSG00000117480](https://www.ensembl.org/id/ENSG00000117480) | fatty acid amide hydrolase [Source:HGNC Symbol;Acc:HGNC:3553] | -1,09 |
| FABP7 | [ENSG00000164434](https://www.ensembl.org/id/ENSG00000164434) | fatty acid binding protein 7 [Source:HGNC Symbol;Acc:HGNC:3562] | -7,00 |
| FAM106A | [ENSG00000273018](https://www.ensembl.org/id/ENSG00000273018) | family with sequence similarity 106 member A [Source:HGNC Symbol;Acc:HGNC:25682] | -1,82 |
| FAM106C | [ENSG00000288235](https://www.ensembl.org/id/ENSG00000288235) | family with sequence similarity 106 member C [Source:HGNC Symbol;Acc:HGNC:38396] | 5,31 |
| FAM153B | [ENSG00000289731](https://www.ensembl.org/id/ENSG00000289731) | family with sequence similarity 153 member B [Source:HGNC Symbol;Acc:HGNC:27323] | -3,40 |
| FAM153CP | [ENSG00000204677](https://www.ensembl.org/id/ENSG00000204677) | family with sequence similarity 153 member C, pseudogene [Source:HGNC Symbol;Acc:HGNC:33936] | -4,25 |
| FAM167A | [ENSG00000154319](https://www.ensembl.org/id/ENSG00000154319) | family with sequence similarity 167 member A [Source:HGNC Symbol;Acc:HGNC:15549] | -4,16 |
| FAM171A2 | [ENSG00000161682](https://www.ensembl.org/id/ENSG00000161682) | family with sequence similarity 171 member A2 [Source:HGNC Symbol;Acc:HGNC:30480] | -2,82 |
| FAM184B | [ENSG00000047662](https://www.ensembl.org/id/ENSG00000047662) | family with sequence similarity 184 member B [Source:HGNC Symbol;Acc:HGNC:29235] | -8,41 |
| FAM240A | [ENSG00000283473](https://www.ensembl.org/id/ENSG00000283473) | family with sequence similarity 240 member A [Source:HGNC Symbol;Acc:HGNC:52390] | -5,60 |
| FAM240B | [ENSG00000283329](https://www.ensembl.org/id/ENSG00000283329) | family with sequence similarity 240 member B [Source:HGNC Symbol;Acc:HGNC:53430] | -5,17 |
| FAM240C | [ENSG00000216921](https://www.ensembl.org/id/ENSG00000216921) | family with sequence similarity 240 member C [Source:HGNC Symbol;Acc:HGNC:54200] | -6,84 |
| FAM83B | [ENSG00000168143](https://www.ensembl.org/id/ENSG00000168143) | family with sequence similarity 83 member B [Source:HGNC Symbol;Acc:HGNC:21357] | -5,07 |
| FAM83D | [ENSG00000101447](https://www.ensembl.org/id/ENSG00000101447) | family with sequence similarity 83 member D [Source:HGNC Symbol;Acc:HGNC:16122] | 2,71 |
| FAM83E | [ENSG00000105523](https://www.ensembl.org/id/ENSG00000105523) | family with sequence similarity 83 member E [Source:HGNC Symbol;Acc:HGNC:25972] | -5,60 |
| FAM83F | [ENSG00000133477](https://www.ensembl.org/id/ENSG00000133477) | family with sequence similarity 83 member F [Source:HGNC Symbol;Acc:HGNC:25148] | -6,21 |
| FAM83H | [ENSG00000180921](https://www.ensembl.org/id/ENSG00000180921) | family with sequence similarity 83 member H [Source:HGNC Symbol;Acc:HGNC:24797] | -3,15 |
| FAM90A20P | [ENSG00000233295](https://www.ensembl.org/id/ENSG00000233295) | family with sequence similarity 90 member A20, pseudogene [Source:HGNC Symbol;Acc:HGNC:32268] | -5,51 |
| FARP1-AS1 | [ENSG00000231194](https://www.ensembl.org/id/ENSG00000231194) | FARP1 antisense RNA 1 [Source:HGNC Symbol;Acc:HGNC:40229] | -3,87 |
| FBP2 | [ENSG00000130957](https://www.ensembl.org/id/ENSG00000130957) | fructose-bisphosphatase 2 [Source:HGNC Symbol;Acc:HGNC:3607] | -7,96 |
| FBXL16 | [ENSG00000127585](https://www.ensembl.org/id/ENSG00000127585) | F-box and leucine rich repeat protein 16 [Source:HGNC Symbol;Acc:HGNC:14150] | -2,21 |
| FBXL19-AS1 | [ENSG00000260852](https://www.ensembl.org/id/ENSG00000260852) | FBXL19 antisense RNA 1 [Source:HGNC Symbol;Acc:HGNC:27557] | -1,74 |
| FBXO2 | [ENSG00000116661](https://www.ensembl.org/id/ENSG00000116661) | F-box protein 2 [Source:HGNC Symbol;Acc:HGNC:13581] | -1,99 |
| FBXO40 | [ENSG00000163833](https://www.ensembl.org/id/ENSG00000163833) | F-box protein 40 [Source:HGNC Symbol;Acc:HGNC:29816] | -5,85 |
| FCAR | [ENSG00000186431](https://www.ensembl.org/id/ENSG00000186431) | Fc alpha receptor [Source:HGNC Symbol;Acc:HGNC:3608] | 5,48 |
| FCGR3B | [ENSG00000162747](https://www.ensembl.org/id/ENSG00000162747) | Fc gamma receptor IIIb [Source:HGNC Symbol;Acc:HGNC:3620] | 4,83 |
| FCN1 | [ENSG00000085265](https://www.ensembl.org/id/ENSG00000085265) | ficolin 1 [Source:HGNC Symbol;Acc:HGNC:3623] | 3,39 |
| FCRL1 | [ENSG00000163534](https://www.ensembl.org/id/ENSG00000163534) | Fc receptor like 1 [Source:HGNC Symbol;Acc:HGNC:18509] | 6,42 |
| FCRL3 | [ENSG00000160856](https://www.ensembl.org/id/ENSG00000160856) | Fc receptor like 3 [Source:HGNC Symbol;Acc:HGNC:18506] | 3,00 |
| FEM1A | [ENSG00000141965](https://www.ensembl.org/id/ENSG00000141965) | fem-1 homolog A [Source:HGNC Symbol;Acc:HGNC:16934] | -1,85 |
| FFAR4 | [ENSG00000186188](https://www.ensembl.org/id/ENSG00000186188) | free fatty acid receptor 4 [Source:HGNC Symbol;Acc:HGNC:19061] | -2,67 |
| FGF6 | [ENSG00000111241](https://www.ensembl.org/id/ENSG00000111241) | fibroblast growth factor 6 [Source:HGNC Symbol;Acc:HGNC:3684] | -6,86 |
| FGFR4 | [ENSG00000160867](https://www.ensembl.org/id/ENSG00000160867) | fibroblast growth factor receptor 4 [Source:HGNC Symbol;Acc:HGNC:3691] | -2,82 |
| FGFRL1 | [ENSG00000127418](https://www.ensembl.org/id/ENSG00000127418) | fibroblast growth factor receptor like 1 [Source:HGNC Symbol;Acc:HGNC:3693] | -0,92 |
| FGL2 | [ENSG00000127951](https://www.ensembl.org/id/ENSG00000127951) | fibrinogen like 2 [Source:HGNC Symbol;Acc:HGNC:3696] | -1,88 |
| FHAD1 | [ENSG00000142621](https://www.ensembl.org/id/ENSG00000142621) | forkhead associated phosphopeptide binding domain 1 [Source:HGNC Symbol;Acc:HGNC:29408] | 2,38 |
| FHIP1A | [ENSG00000164142](https://www.ensembl.org/id/ENSG00000164142) | FHF complex subunit HOOK interacting protein 1A [Source:HGNC Symbol;Acc:HGNC:34237] | -2,79 |
| FHL2 | [ENSG00000115641](https://www.ensembl.org/id/ENSG00000115641) | four and a half LIM domains 2 [Source:HGNC Symbol;Acc:HGNC:3703] | 1,29 |
| FHL3 | [ENSG00000183386](https://www.ensembl.org/id/ENSG00000183386) | four and a half LIM domains 3 [Source:HGNC Symbol;Acc:HGNC:3704] | -2,15 |
| FITM1 | [ENSG00000139914](https://www.ensembl.org/id/ENSG00000139914) | fat storage inducing transmembrane protein 1 [Source:HGNC Symbol;Acc:HGNC:33714] | -6,44 |
| FLJ16779 | [ENSG00000275620](https://www.ensembl.org/id/ENSG00000275620) | uncharacterized LOC100192386 [Source:NCBI gene (formerly Entrezgene);Acc:100192386] | -3,52 |
| FMO3 | [ENSG00000007933](https://www.ensembl.org/id/ENSG00000007933) | flavin containing dimethylaniline monoxygenase 3 [Source:HGNC Symbol;Acc:HGNC:3771] | -1,53 |
| FN3KRP | [ENSG00000141560](https://www.ensembl.org/id/ENSG00000141560) | fructosamine 3 kinase related protein [Source:HGNC Symbol;Acc:HGNC:25700] | 0,87 |
| FNDC1 | [ENSG00000164694](https://www.ensembl.org/id/ENSG00000164694) | fibronectin type III domain containing 1 [Source:HGNC Symbol;Acc:HGNC:21184] | -2,67 |
| FNDC4 | [ENSG00000115226](https://www.ensembl.org/id/ENSG00000115226) | fibronectin type III domain containing 4 [Source:HGNC Symbol;Acc:HGNC:20239] | -1,22 |
| FNDC5 | [ENSG00000160097](https://www.ensembl.org/id/ENSG00000160097) | fibronectin type III domain containing 5 [Source:HGNC Symbol;Acc:HGNC:20240] | -3,12 |
| FOLR3 | [ENSG00000110203](https://www.ensembl.org/id/ENSG00000110203) | folate receptor gamma [Source:HGNC Symbol;Acc:HGNC:3795] | 6,13 |
| FOXD3-AS1 | [ENSG00000230798](https://www.ensembl.org/id/ENSG00000230798) | FOXD3 antisense RNA 1 [Source:HGNC Symbol;Acc:HGNC:40241] | -2,96 |
| FPR1 | [ENSG00000171051](https://www.ensembl.org/id/ENSG00000171051) | formyl peptide receptor 1 [Source:HGNC Symbol;Acc:HGNC:3826] | 4,59 |
| FPR2 | [ENSG00000171049](https://www.ensembl.org/id/ENSG00000171049) | formyl peptide receptor 2 [Source:HGNC Symbol;Acc:HGNC:3827] | 5,23 |
| FRAS1 | [ENSG00000138759](https://www.ensembl.org/id/ENSG00000138759) | Fraser extracellular matrix complex subunit 1 [Source:HGNC Symbol;Acc:HGNC:19185] | -2,33 |
| FREM2 | [ENSG00000150893](https://www.ensembl.org/id/ENSG00000150893) | FRAS1 related extracellular matrix 2 [Source:HGNC Symbol;Acc:HGNC:25396] | -4,91 |
| FREM3 | [ENSG00000183090](https://www.ensembl.org/id/ENSG00000183090) | FRAS1 related extracellular matrix 3 [Source:HGNC Symbol;Acc:HGNC:25172] | 5,23 |
| FRMPD1 | [ENSG00000070601](https://www.ensembl.org/id/ENSG00000070601) | FERM and PDZ domain containing 1 [Source:HGNC Symbol;Acc:HGNC:29159] | -4,45 |
| FSCN1 | [ENSG00000075618](https://www.ensembl.org/id/ENSG00000075618) | fascin actin-bundling protein 1 [Source:HGNC Symbol;Acc:HGNC:11148] | -1,03 |
| FSD2 | [ENSG00000186628](https://www.ensembl.org/id/ENSG00000186628) | fibronectin type III and SPRY domain containing 2 [Source:HGNC Symbol;Acc:HGNC:18024] | -5,11 |
| FTCD | [ENSG00000160282](https://www.ensembl.org/id/ENSG00000160282) | formimidoyltransferase cyclodeaminase [Source:HGNC Symbol;Acc:HGNC:3974] | -2,39 |
| FZD2 | [ENSG00000180340](https://www.ensembl.org/id/ENSG00000180340) | frizzled class receptor 2 [Source:HGNC Symbol;Acc:HGNC:4040] | -1,58 |
| FZD9 | [ENSG00000188763](https://www.ensembl.org/id/ENSG00000188763) | frizzled class receptor 9 [Source:HGNC Symbol;Acc:HGNC:4047] | -2,59 |
| GAB4 | [ENSG00000215568](https://www.ensembl.org/id/ENSG00000215568) | GRB2 associated binding protein family member 4 [Source:HGNC Symbol;Acc:HGNC:18325] | -5,52 |
| GADL1 | [ENSG00000144644](https://www.ensembl.org/id/ENSG00000144644) | glutamate decarboxylase like 1 [Source:HGNC Symbol;Acc:HGNC:27949] | -4,54 |
| GAL | [ENSG00000069482](https://www.ensembl.org/id/ENSG00000069482) | galanin and GMAP prepropeptide [Source:HGNC Symbol;Acc:HGNC:4114] | -3,30 |
| GALNT9 | [ENSG00000182870](https://www.ensembl.org/id/ENSG00000182870) | polypeptide N-acetylgalactosaminyltransferase 9 [Source:HGNC Symbol;Acc:HGNC:4131] | -5,94 |
| GALNTL6 | [ENSG00000174473](https://www.ensembl.org/id/ENSG00000174473) | polypeptide N-acetylgalactosaminyltransferase like 6 [Source:HGNC Symbol;Acc:HGNC:33844] | -3,81 |
| GAMT | [ENSG00000130005](https://www.ensembl.org/id/ENSG00000130005) | guanidinoacetate N-methyltransferase [Source:HGNC Symbol;Acc:HGNC:4136] | -2,06 |
| GAPT | [ENSG00000175857](https://www.ensembl.org/id/ENSG00000175857) | GRB2 binding adaptor protein, transmembrane [Source:HGNC Symbol;Acc:HGNC:26588] | 5,45 |
| GARIN4 | [ENSG00000162771](https://www.ensembl.org/id/ENSG00000162771) | golgi associated RAB2 interactor family member 4 [Source:HGNC Symbol;Acc:HGNC:26541] | -2,73 |
| GASK1A | [ENSG00000144649](https://www.ensembl.org/id/ENSG00000144649) | golgi associated kinase 1A [Source:HGNC Symbol;Acc:HGNC:24485] | 1,29 |
| GATA1 | [ENSG00000102145](https://www.ensembl.org/id/ENSG00000102145) | GATA binding protein 1 [Source:HGNC Symbol;Acc:HGNC:4170] | 3,13 |
| GATA4 | [ENSG00000136574](https://www.ensembl.org/id/ENSG00000136574) | GATA binding protein 4 [Source:HGNC Symbol;Acc:HGNC:4173] | -5,38 |
| GBX1 | [ENSG00000164900](https://www.ensembl.org/id/ENSG00000164900) | gastrulation brain homeobox 1 [Source:HGNC Symbol;Acc:HGNC:4185] | -3,16 |
| GCSAML | [ENSG00000169224](https://www.ensembl.org/id/ENSG00000169224) | germinal center associated signaling and motility like [Source:HGNC Symbol;Acc:HGNC:29583] | -3,40 |
| GDA | [ENSG00000119125](https://www.ensembl.org/id/ENSG00000119125) | guanine deaminase [Source:HGNC Symbol;Acc:HGNC:4212] | -7,22 |
| GDF5 | [ENSG00000125965](https://www.ensembl.org/id/ENSG00000125965) | growth differentiation factor 5 [Source:HGNC Symbol;Acc:HGNC:4220] | -2,65 |
| GDPD5 | [ENSG00000158555](https://www.ensembl.org/id/ENSG00000158555) | glycerophosphodiester phosphodiesterase domain containing 5 [Source:HGNC Symbol;Acc:HGNC:28804] | -1,22 |
| gene:ENSG00000173366 | [ENSG00000173366](https://www.ensembl.org/id/ENSG00000173366) | novel twinfilin, actin-binding protein, homolog 2 (Drosophila) (TWF2) and toll-like receptor 9 (TLR9) protein | 5,14 |
| gene:ENSG00000189229 | [ENSG00000189229](https://www.ensembl.org/id/ENSG00000189229) | novel transcript | -6,18 |
| gene:ENSG00000189316 | [ENSG00000189316](https://www.ensembl.org/id/ENSG00000189316) | novel transcript, antisense to ZNF273 | -4,01 |
| gene:ENSG00000198491 | [ENSG00000198491](https://www.ensembl.org/id/ENSG00000198491) | novel transcript | -4,89 |
| gene:ENSG00000203392 | [ENSG00000203392](https://www.ensembl.org/id/ENSG00000203392) | novel transcript, antisense to CSPG4 | 1,38 |
| gene:ENSG00000204117 | [ENSG00000204117](https://www.ensembl.org/id/ENSG00000204117) | novel transcript | -5,32 |
| gene:ENSG00000204422 | [ENSG00000204422](https://www.ensembl.org/id/ENSG00000204422) | novel transcript | -6,14 |
| gene:ENSG00000214970 | [ENSG00000214970](https://www.ensembl.org/id/ENSG00000214970) | novel transcript, antisense to MYH2, MYH8, MYH1 and MYH2 | -4,96 |
| gene:ENSG00000223343 | [ENSG00000223343](https://www.ensembl.org/id/ENSG00000223343) | novel transcript | -3,48 |
| gene:ENSG00000224661 | [ENSG00000224661](https://www.ensembl.org/id/ENSG00000224661) | novel transcript | -5,44 |
| gene:ENSG00000226087 | [ENSG00000226087](https://www.ensembl.org/id/ENSG00000226087) | novel transcript | -5,93 |
| gene:ENSG00000226281 | [ENSG00000226281](https://www.ensembl.org/id/ENSG00000226281) | novel transcript | -3,34 |
| gene:ENSG00000227496 | [ENSG00000227496](https://www.ensembl.org/id/ENSG00000227496) | novel transcript | -4,78 |
| gene:ENSG00000228509 | [ENSG00000228509](https://www.ensembl.org/id/ENSG00000228509) | novel transcript, antisense to NAB1 | -4,77 |
| gene:ENSG00000228643 | [ENSG00000228643](https://www.ensembl.org/id/ENSG00000228643) | novel transcript | 2,81 |
| gene:ENSG00000228843 | [ENSG00000228843](https://www.ensembl.org/id/ENSG00000228843) | novel transcript | -2,93 |
| gene:ENSG00000229227 | [ENSG00000229227](https://www.ensembl.org/id/ENSG00000229227) | novel transcript | 2,26 |
| gene:ENSG00000229425 | [ENSG00000229425](https://www.ensembl.org/id/ENSG00000229425) | novel transcript | -4,02 |
| gene:ENSG00000230289 | [ENSG00000230289](https://www.ensembl.org/id/ENSG00000230289) | novel transcript | 5,03 |
| gene:ENSG00000230534 | [ENSG00000230534](https://www.ensembl.org/id/ENSG00000230534) | novel transcript | -5,88 |
| gene:ENSG00000231193 | [ENSG00000231193](https://www.ensembl.org/id/ENSG00000231193) | novel transcript | -3,90 |
| gene:ENSG00000231236 | [ENSG00000231236](https://www.ensembl.org/id/ENSG00000231236) | novel transcript | -3,73 |
| gene:ENSG00000231252 | [ENSG00000231252](https://www.ensembl.org/id/ENSG00000231252) | novel transcript | -3,14 |
| gene:ENSG00000231412 | [ENSG00000231412](https://www.ensembl.org/id/ENSG00000231412) | novel transcript | -6,20 |
| gene:ENSG00000233005 | [ENSG00000233005](https://www.ensembl.org/id/ENSG00000233005) | novel transcript | -4,39 |
| gene:ENSG00000233478 | [ENSG00000233478](https://www.ensembl.org/id/ENSG00000233478) | novel transcript | 3,84 |
| gene:ENSG00000234147 | [ENSG00000234147](https://www.ensembl.org/id/ENSG00000234147) | novel transcript | -4,51 |
| gene:ENSG00000234292 | [ENSG00000234292](https://www.ensembl.org/id/ENSG00000234292) | novel transcript | 2,89 |
| gene:ENSG00000234418 | [ENSG00000234418](https://www.ensembl.org/id/ENSG00000234418) | novel transcript | 5,10 |
| gene:ENSG00000234477 | [ENSG00000234477](https://www.ensembl.org/id/ENSG00000234477) | novel transcript | -5,33 |
| gene:ENSG00000234699 | [ENSG00000234699](https://www.ensembl.org/id/ENSG00000234699) | novel transcript | 2,55 |
| gene:ENSG00000235007 | [ENSG00000235007](https://www.ensembl.org/id/ENSG00000235007) | novel protein | 3,59 |
| gene:ENSG00000235070 | [ENSG00000235070](https://www.ensembl.org/id/ENSG00000235070) | novel transcript | -7,04 |
| gene:ENSG00000235296 | [ENSG00000235296](https://www.ensembl.org/id/ENSG00000235296) | novel transcript | -5,17 |
| gene:ENSG00000235480 | [ENSG00000235480](https://www.ensembl.org/id/ENSG00000235480) | novel transcript | -3,17 |
| gene:ENSG00000237927 | [ENSG00000237927](https://www.ensembl.org/id/ENSG00000237927) | novel transcript | -3,14 |
| gene:ENSG00000238007 | [ENSG00000238007](https://www.ensembl.org/id/ENSG00000238007) | novel transcript | -5,76 |
| gene:ENSG00000238039 | [ENSG00000238039](https://www.ensembl.org/id/ENSG00000238039) | novel transcript | -2,22 |
| gene:ENSG00000241168 | [ENSG00000241168](https://www.ensembl.org/id/ENSG00000241168) | novel transcript | 5,14 |
| gene:ENSG00000243243 | [ENSG00000243243](https://www.ensembl.org/id/ENSG00000243243) | novel transcript | 3,27 |
| gene:ENSG00000244998 | [ENSG00000244998](https://www.ensembl.org/id/ENSG00000244998) | novel transcript, antisense to PTP4A3 | -7,71 |
| gene:ENSG00000247311 | [ENSG00000247311](https://www.ensembl.org/id/ENSG00000247311) | novel transcript | -4,98 |
| gene:ENSG00000248100 | [ENSG00000248100](https://www.ensembl.org/id/ENSG00000248100) | novel transcript | 4,91 |
| gene:ENSG00000248367 | [ENSG00000248367](https://www.ensembl.org/id/ENSG00000248367) | novel transcript | 3,50 |
| gene:ENSG00000248491 | [ENSG00000248491](https://www.ensembl.org/id/ENSG00000248491) | novel transcript | -5,36 |
| gene:ENSG00000248896 | [ENSG00000248896](https://www.ensembl.org/id/ENSG00000248896) | novel transcript, antisense to SOX7 and PINX1 | 2,70 |
| gene:ENSG00000249240 | [ENSG00000249240](https://www.ensembl.org/id/ENSG00000249240) | novel protein | 2,96 |
| gene:ENSG00000249483 | [ENSG00000249483](https://www.ensembl.org/id/ENSG00000249483) | novel transcript | -5,36 |
| gene:ENSG00000249773 | [ENSG00000249773](https://www.ensembl.org/id/ENSG00000249773) | novel zinc finger protein 713 (ZNF713) and mitochondrial ribosomal protein S17 (MRPS17) protein | -6,47 |
| gene:ENSG00000249971 | [ENSG00000249971](https://www.ensembl.org/id/ENSG00000249971) | novel transcript | -6,21 |
| gene:ENSG00000250041 | [ENSG00000250041](https://www.ensembl.org/id/ENSG00000250041) | novel transcript | -5,05 |
| gene:ENSG00000250978 | [ENSG00000250978](https://www.ensembl.org/id/ENSG00000250978) | novel transcript | -8,91 |
| gene:ENSG00000250994 | [ENSG00000250994](https://www.ensembl.org/id/ENSG00000250994) | novel transcript | -5,27 |
| gene:ENSG00000251023 | [ENSG00000251023](https://www.ensembl.org/id/ENSG00000251023) | novel transcript | 1,85 |
| gene:ENSG00000251665 | [ENSG00000251665](https://www.ensembl.org/id/ENSG00000251665) | novel transcript | -3,41 |
| gene:ENSG00000253115 | [ENSG00000253115](https://www.ensembl.org/id/ENSG00000253115) | novel transcript | -5,53 |
| gene:ENSG00000253348 | [ENSG00000253348](https://www.ensembl.org/id/ENSG00000253348) | novel transcript | -4,37 |
| gene:ENSG00000253389 | [ENSG00000253389](https://www.ensembl.org/id/ENSG00000253389) | novel transcript, antisense to ANK1 | -4,36 |
| gene:ENSG00000253985 | [ENSG00000253985](https://www.ensembl.org/id/ENSG00000253985) | novel transcript | -4,98 |
| gene:ENSG00000254288 | [ENSG00000254288](https://www.ensembl.org/id/ENSG00000254288) | novel transcript | -2,85 |
| gene:ENSG00000254343 | [ENSG00000254343](https://www.ensembl.org/id/ENSG00000254343) | novel transcript | -3,00 |
| gene:ENSG00000254485 | [ENSG00000254485](https://www.ensembl.org/id/ENSG00000254485) | novel transcript, antisense to THUMPD3 | -3,48 |
| gene:ENSG00000254653 | [ENSG00000254653](https://www.ensembl.org/id/ENSG00000254653) | novel transcript | -5,33 |
| gene:ENSG00000255126 | [ENSG00000255126](https://www.ensembl.org/id/ENSG00000255126) | novel transcript | -4,71 |
| gene:ENSG00000255202 | [ENSG00000255202](https://www.ensembl.org/id/ENSG00000255202) | novel transcript | -3,40 |
| gene:ENSG00000255339 | [ENSG00000255339](https://www.ensembl.org/id/ENSG00000255339) | NADH dehydrogenase (ubiquinone) 1 beta subcomplex, 8, 19kDa (NDUFB8) and SEC31 homolog B (S. cerevisiae) (SEC31B) readthrough | -1,87 |
| gene:ENSG00000255663 | [ENSG00000255663](https://www.ensembl.org/id/ENSG00000255663) | novel transcript, RBM7-REXO2 readthrough | -3,32 |
| gene:ENSG00000255801 | [ENSG00000255801](https://www.ensembl.org/id/ENSG00000255801) | novel transcript | 4,95 |
| gene:ENSG00000255835 | [ENSG00000255835](https://www.ensembl.org/id/ENSG00000255835) | novel protein | 5,98 |
| gene:ENSG00000256196 | [ENSG00000256196](https://www.ensembl.org/id/ENSG00000256196) | novel transcript | -4,77 |
| gene:ENSG00000256407 | [ENSG00000256407](https://www.ensembl.org/id/ENSG00000256407) | novel transcript | -5,22 |
| gene:ENSG00000256500 | [ENSG00000256500](https://www.ensembl.org/id/ENSG00000256500) | novel protein | -9,21 |
| gene:ENSG00000256928 | [ENSG00000256928](https://www.ensembl.org/id/ENSG00000256928) | novel transcript | -6,04 |
| gene:ENSG00000257322 | [ENSG00000257322](https://www.ensembl.org/id/ENSG00000257322) | novel transcript, antisense to novel protein | -4,76 |
| gene:ENSG00000257434 | [ENSG00000257434](https://www.ensembl.org/id/ENSG00000257434) | novel transcript, antisense to KCNC2 | -5,60 |
| gene:ENSG00000257764 | [ENSG00000257764](https://www.ensembl.org/id/ENSG00000257764) | novel transcript, antisense to LYZ | -5,42 |
| gene:ENSG00000257921 | [ENSG00000257921](https://www.ensembl.org/id/ENSG00000257921) | novel protein | -3,42 |
| gene:ENSG00000258231 | [ENSG00000258231](https://www.ensembl.org/id/ENSG00000258231) | novel transcript | -6,43 |
| gene:ENSG00000258365 | [ENSG00000258365](https://www.ensembl.org/id/ENSG00000258365) | novel transcript, antisense to PLXNC1 | -2,75 |
| gene:ENSG00000258504 | [ENSG00000258504](https://www.ensembl.org/id/ENSG00000258504) | novel transcript | -7,41 |
| gene:ENSG00000258525 | [ENSG00000258525](https://www.ensembl.org/id/ENSG00000258525) | novel transcript, antisense to COCH | -3,68 |
| gene:ENSG00000258660 | [ENSG00000258660](https://www.ensembl.org/id/ENSG00000258660) | novel transcript, antisense to DNAL1 | -5,98 |
| gene:ENSG00000258661 | [ENSG00000258661](https://www.ensembl.org/id/ENSG00000258661) | novel transcript, antisense to PAX9 | 3,16 |
| gene:ENSG00000258752 | [ENSG00000258752](https://www.ensembl.org/id/ENSG00000258752) | novel transcript, antisense to FOXN3 | 4,70 |
| gene:ENSG00000258757 | [ENSG00000258757](https://www.ensembl.org/id/ENSG00000258757) | novel transcript, antisense to ERO1L | -4,98 |
| gene:ENSG00000258761 | [ENSG00000258761](https://www.ensembl.org/id/ENSG00000258761) | novel transcript, antisense to SLCO3A1 | -5,49 |
| gene:ENSG00000258943 | [ENSG00000258943](https://www.ensembl.org/id/ENSG00000258943) | novel transcript | -4,72 |
| gene:ENSG00000259219 | [ENSG00000259219](https://www.ensembl.org/id/ENSG00000259219) | novel transcript, antisense to ADAMTS17 | -4,98 |
| gene:ENSG00000259359 | [ENSG00000259359](https://www.ensembl.org/id/ENSG00000259359) | novel transcript | 5,06 |
| gene:ENSG00000259408 | [ENSG00000259408](https://www.ensembl.org/id/ENSG00000259408) | novel transcript, antisense to RYR3 | 2,80 |
| gene:ENSG00000259744 | [ENSG00000259744](https://www.ensembl.org/id/ENSG00000259744) | novel transcript, sense intronic to LARP6 | 3,09 |
| gene:ENSG00000259881 | [ENSG00000259881](https://www.ensembl.org/id/ENSG00000259881) | novel transcript, antisense to CBFA2T3 | -5,81 |
| gene:ENSG00000259921 | [ENSG00000259921](https://www.ensembl.org/id/ENSG00000259921) | novel transcript, sense intronic to TTC23 | -3,69 |
| gene:ENSG00000260092 | [ENSG00000260092](https://www.ensembl.org/id/ENSG00000260092) | TMEM231-CHST5 readthrough protein | -2,46 |
| gene:ENSG00000260188 | [ENSG00000260188](https://www.ensembl.org/id/ENSG00000260188) | novel transcript, antisense to SLC22A16 | -5,76 |
| gene:ENSG00000260277 | [ENSG00000260277](https://www.ensembl.org/id/ENSG00000260277) | novel transcript, antisense to C16orf52 and VWA3A | -5,60 |
| gene:ENSG00000260398 | [ENSG00000260398](https://www.ensembl.org/id/ENSG00000260398) | novel transcript, overlapping to PKIA | -5,52 |
| gene:ENSG00000260476 | [ENSG00000260476](https://www.ensembl.org/id/ENSG00000260476) | novel transcript | -5,17 |
| gene:ENSG00000260484 | [ENSG00000260484](https://www.ensembl.org/id/ENSG00000260484) | novel transcript | 6,01 |
| gene:ENSG00000260604 | [ENSG00000260604](https://www.ensembl.org/id/ENSG00000260604) | novel transcript | -2,87 |
| gene:ENSG00000260646 | [ENSG00000260646](https://www.ensembl.org/id/ENSG00000260646) | novel transcript | -4,12 |
| gene:ENSG00000260657 | [ENSG00000260657](https://www.ensembl.org/id/ENSG00000260657) | novel transcript | -4,92 |
| gene:ENSG00000260672 | [ENSG00000260672](https://www.ensembl.org/id/ENSG00000260672) | novel transcript | 2,97 |
| gene:ENSG00000261026 | [ENSG00000261026](https://www.ensembl.org/id/ENSG00000261026) | novel transcript, overlapping to EGR3 | -2,21 |
| gene:ENSG00000261076 | [ENSG00000261076](https://www.ensembl.org/id/ENSG00000261076) | novel transcript | -5,06 |
| gene:ENSG00000261103 | [ENSG00000261103](https://www.ensembl.org/id/ENSG00000261103) | novel transcript, antisense to CDH13 | -2,54 |
| gene:ENSG00000261159 | [ENSG00000261159](https://www.ensembl.org/id/ENSG00000261159) | novel transcript | -2,56 |
| gene:ENSG00000261168 | [ENSG00000261168](https://www.ensembl.org/id/ENSG00000261168) | novel transcript, sense overlapping SEMA6C | -3,26 |
| gene:ENSG00000261211 | [ENSG00000261211](https://www.ensembl.org/id/ENSG00000261211) | novel transcript | -4,12 |
| gene:ENSG00000261253 | [ENSG00000261253](https://www.ensembl.org/id/ENSG00000261253) | novel transcript, antisense to ANKRD11 | -2,94 |
| gene:ENSG00000261433 | [ENSG00000261433](https://www.ensembl.org/id/ENSG00000261433) | novel transcript | -3,88 |
| gene:ENSG00000261434 | [ENSG00000261434](https://www.ensembl.org/id/ENSG00000261434) | novel transcript, overlapping LRRC14B | -5,45 |
| gene:ENSG00000261445 | [ENSG00000261445](https://www.ensembl.org/id/ENSG00000261445) | novel transcript | -5,22 |
| gene:ENSG00000261822 | [ENSG00000261822](https://www.ensembl.org/id/ENSG00000261822) | novel transcript, antisense to HAUS2 | -3,62 |
| gene:ENSG00000262265 | [ENSG00000262265](https://www.ensembl.org/id/ENSG00000262265) | novel transcript, sense intronic to CDC27 | 2,42 |
| gene:ENSG00000262526 | [ENSG00000262526](https://www.ensembl.org/id/ENSG00000262526) | novel protein | 5,60 |
| gene:ENSG00000262791 | [ENSG00000262791](https://www.ensembl.org/id/ENSG00000262791) | novel transcript, antisense to WDR81 | -5,52 |
| gene:ENSG00000263020 | [ENSG00000263020](https://www.ensembl.org/id/ENSG00000263020) | novel protein | -1,36 |
| gene:ENSG00000263938 | [ENSG00000263938](https://www.ensembl.org/id/ENSG00000263938) | novel transcript, sense intronic to PPM1E | -5,60 |
| gene:ENSG00000263952 | [ENSG00000263952](https://www.ensembl.org/id/ENSG00000263952) | novel transcript, antisense to PIEZO2 | -4,98 |
| gene:ENSG00000264151 | [ENSG00000264151](https://www.ensembl.org/id/ENSG00000264151) | novel transcript | -5,74 |
| gene:ENSG00000264324 | [ENSG00000264324](https://www.ensembl.org/id/ENSG00000264324) | novel protein | 3,61 |
| gene:ENSG00000264490 | [ENSG00000264490](https://www.ensembl.org/id/ENSG00000264490) | novel transcript | -4,40 |
| gene:ENSG00000264668 | [ENSG00000264668](https://www.ensembl.org/id/ENSG00000264668) | novel protein | 5,88 |
| gene:ENSG00000265399 | [ENSG00000265399](https://www.ensembl.org/id/ENSG00000265399) | novel transcript, antisense to MYOM1 | -2,70 |
| gene:ENSG00000265751 | [ENSG00000265751](https://www.ensembl.org/id/ENSG00000265751) | novel transcript, antisense to GREB1L | -6,86 |
| gene:ENSG00000266106 | [ENSG00000266106](https://www.ensembl.org/id/ENSG00000266106) | novel transcript, antisense to DNAI2 | -4,98 |
| gene:ENSG00000266495 | [ENSG00000266495](https://www.ensembl.org/id/ENSG00000266495) | novel transcript, antisense to CABLES1 | -5,35 |
| gene:ENSG00000266844 | [ENSG00000266844](https://www.ensembl.org/id/ENSG00000266844) | novel transcript, antisense to MBP | 5,60 |
| gene:ENSG00000266994 | [ENSG00000266994](https://www.ensembl.org/id/ENSG00000266994) | novel transcript | -5,19 |
| gene:ENSG00000267007 | [ENSG00000267007](https://www.ensembl.org/id/ENSG00000267007) | novel transcript, antisense to REXO1 | -6,21 |
| gene:ENSG00000267022 | [ENSG00000267022](https://www.ensembl.org/id/ENSG00000267022) | novel protein | -6,46 |
| gene:ENSG00000267042 | [ENSG00000267042](https://www.ensembl.org/id/ENSG00000267042) | novel transcript, antisense to CCR10 | -3,73 |
| gene:ENSG00000267150 | [ENSG00000267150](https://www.ensembl.org/id/ENSG00000267150) | novel transcript, antisense ZNF519 | 4,91 |
| gene:ENSG00000267174 | [ENSG00000267174](https://www.ensembl.org/id/ENSG00000267174) | novel transcript | 4,31 |
| gene:ENSG00000267364 | [ENSG00000267364](https://www.ensembl.org/id/ENSG00000267364) | novel transcript | -2,38 |
| gene:ENSG00000267423 | [ENSG00000267423](https://www.ensembl.org/id/ENSG00000267423) | novel transcript | -6,43 |
| gene:ENSG00000267436 | [ENSG00000267436](https://www.ensembl.org/id/ENSG00000267436) | novel transcript, antisense to MFSD12 | -3,86 |
| gene:ENSG00000267561 | [ENSG00000267561](https://www.ensembl.org/id/ENSG00000267561) | novel protein | -6,86 |
| gene:ENSG00000267653 | [ENSG00000267653](https://www.ensembl.org/id/ENSG00000267653) | novel transcript | -3,14 |
| gene:ENSG00000267727 | [ENSG00000267727](https://www.ensembl.org/id/ENSG00000267727) | novel transcript | -5,52 |
| gene:ENSG00000267939 | [ENSG00000267939](https://www.ensembl.org/id/ENSG00000267939) | novel transcript | -5,58 |
| gene:ENSG00000268240 | [ENSG00000268240](https://www.ensembl.org/id/ENSG00000268240) | novel transcript | 5,35 |
| gene:ENSG00000268518 | [ENSG00000268518](https://www.ensembl.org/id/ENSG00000268518) | novel transcript | -5,17 |
| gene:ENSG00000268734 | [ENSG00000268734](https://www.ensembl.org/id/ENSG00000268734) | novel transcript | 7,39 |
| gene:ENSG00000269604 | [ENSG00000269604](https://www.ensembl.org/id/ENSG00000269604) | novel transcript, antisense to FEM1A | -2,23 |
| gene:ENSG00000269693 | [ENSG00000269693](https://www.ensembl.org/id/ENSG00000269693) | novel transcript | -5,30 |
| gene:ENSG00000269921 | [ENSG00000269921](https://www.ensembl.org/id/ENSG00000269921) | novel transcript | 5,55 |
| gene:ENSG00000270031 | [ENSG00000270031](https://www.ensembl.org/id/ENSG00000270031) | novel transcript, sense intronic to STX12 | 2,99 |
| gene:ENSG00000270110 | [ENSG00000270110](https://www.ensembl.org/id/ENSG00000270110) | novel transcript, antisense to OBSCN | -4,85 |
| gene:ENSG00000270149 | [ENSG00000270149](https://www.ensembl.org/id/ENSG00000270149) | novel transcript, TSTD1 - F11R readthrough | -4,82 |
| gene:ENSG00000270696 | [ENSG00000270696](https://www.ensembl.org/id/ENSG00000270696) | novel transcript, antisense to C2orf3 | 6,40 |
| gene:ENSG00000270972 | [ENSG00000270972](https://www.ensembl.org/id/ENSG00000270972) | novel transcript | -5,23 |
| gene:ENSG00000271420 | [ENSG00000271420](https://www.ensembl.org/id/ENSG00000271420) | novel transcript | -2,90 |
| gene:ENSG00000271579 | [ENSG00000271579](https://www.ensembl.org/id/ENSG00000271579) | novel transcript | -4,53 |
| gene:ENSG00000271743 | [ENSG00000271743](https://www.ensembl.org/id/ENSG00000271743) | novel transcript | -2,60 |
| gene:ENSG00000272217 | [ENSG00000272217](https://www.ensembl.org/id/ENSG00000272217) | novel transcript | -4,14 |
| gene:ENSG00000272386 | [ENSG00000272386](https://www.ensembl.org/id/ENSG00000272386) | novel transcript, sense intronic to PRCD | -4,98 |
| gene:ENSG00000272491 | [ENSG00000272491](https://www.ensembl.org/id/ENSG00000272491) | novel transcript, antisense to SLC5A9 | -2,08 |
| gene:ENSG00000272498 | [ENSG00000272498](https://www.ensembl.org/id/ENSG00000272498) | novel transcript, antisense to RFTN1 | 4,70 |
| gene:ENSG00000272518 | [ENSG00000272518](https://www.ensembl.org/id/ENSG00000272518) | novel transcript, sense intronic to MRPS28 | -2,43 |
| gene:ENSG00000272529 | [ENSG00000272529](https://www.ensembl.org/id/ENSG00000272529) | novel transcript, antisense to RFTN1 | 4,38 |
| gene:ENSG00000272541 | [ENSG00000272541](https://www.ensembl.org/id/ENSG00000272541) | novel transcript | -2,32 |
| gene:ENSG00000272936 | [ENSG00000272936](https://www.ensembl.org/id/ENSG00000272936) | novel transcript, antisense to GNPDA2 | -5,63 |
| gene:ENSG00000272941 | [ENSG00000272941](https://www.ensembl.org/id/ENSG00000272941) | novel transcript, antisense to C7orf49 | -2,43 |
| gene:ENSG00000272967 | [ENSG00000272967](https://www.ensembl.org/id/ENSG00000272967) | novel transcript, antisense to ADPRH | -3,93 |
| gene:ENSG00000273046 | [ENSG00000273046](https://www.ensembl.org/id/ENSG00000273046) | novel transcript | 1,79 |
| gene:ENSG00000273059 | [ENSG00000273059](https://www.ensembl.org/id/ENSG00000273059) | novel transcript | 4,95 |
| gene:ENSG00000273069 | [ENSG00000273069](https://www.ensembl.org/id/ENSG00000273069) | novel transcript | -4,72 |
| gene:ENSG00000273272 | [ENSG00000273272](https://www.ensembl.org/id/ENSG00000273272) | novel transcript | 6,19 |
| gene:ENSG00000273406 | [ENSG00000273406](https://www.ensembl.org/id/ENSG00000273406) | novel transcript | -5,33 |
| gene:ENSG00000273424 | [ENSG00000273424](https://www.ensembl.org/id/ENSG00000273424) | novel transcript | -5,43 |
| gene:ENSG00000273449 | [ENSG00000273449](https://www.ensembl.org/id/ENSG00000273449) | novel transcript | 4,17 |
| gene:ENSG00000273998 | [ENSG00000273998](https://www.ensembl.org/id/ENSG00000273998) | novel transcript | -5,92 |
| gene:ENSG00000274021 | [ENSG00000274021](https://www.ensembl.org/id/ENSG00000274021) | novel transcript, antisense to DUSP6 | -2,44 |
| gene:ENSG00000274276 | [ENSG00000274276](https://www.ensembl.org/id/ENSG00000274276) | cystathionine-beta-synthase like | 7,17 |
| gene:ENSG00000274312 | [ENSG00000274312](https://www.ensembl.org/id/ENSG00000274312) | novel transcript | 2,49 |
| gene:ENSG00000274565 | [ENSG00000274565](https://www.ensembl.org/id/ENSG00000274565) | novel transcript | -1,76 |
| gene:ENSG00000274751 | [ENSG00000274751](https://www.ensembl.org/id/ENSG00000274751) | novel transcript, antisense to UBE2I | -5,59 |
| gene:ENSG00000275392 | [ENSG00000275392](https://www.ensembl.org/id/ENSG00000275392) | novel transcript, antisense to C4BPA | -4,08 |
| gene:ENSG00000275413 | [ENSG00000275413](https://www.ensembl.org/id/ENSG00000275413) | novel transcript, antisense to TTC19 | 5,18 |
| gene:ENSG00000275481 | [ENSG00000275481](https://www.ensembl.org/id/ENSG00000275481) | novel transcript | 4,06 |
| gene:ENSG00000275993 | [ENSG00000275993](https://www.ensembl.org/id/ENSG00000275993) | novel protein, similar to salt-inducible kinase 1 SIK1 | 1,76 |
| gene:ENSG00000276012 | [ENSG00000276012](https://www.ensembl.org/id/ENSG00000276012) | novel transcript, antisense to FGF14 | -3,87 |
| gene:ENSG00000276076 | [ENSG00000276076](https://www.ensembl.org/id/ENSG00000276076) | novel protein, similar to crystallin, alpha A CRYAA | 4,86 |
| gene:ENSG00000276418 | [ENSG00000276418](https://www.ensembl.org/id/ENSG00000276418) | novel protein, TPD52-MRPS28 readthrough | -5,17 |
| gene:ENSG00000276445 | [ENSG00000276445](https://www.ensembl.org/id/ENSG00000276445) | novel transcript | -3,02 |
| gene:ENSG00000276850 | [ENSG00000276850](https://www.ensembl.org/id/ENSG00000276850) | novel transcript | -8,43 |
| gene:ENSG00000276851 | [ENSG00000276851](https://www.ensembl.org/id/ENSG00000276851) | novel transcript | -3,33 |
| gene:ENSG00000277270 | [ENSG00000277270](https://www.ensembl.org/id/ENSG00000277270) | novel transcript | 4,58 |
| gene:ENSG00000277287 | [ENSG00000277287](https://www.ensembl.org/id/ENSG00000277287) | novel transcript | -3,03 |
| gene:ENSG00000277476 | [ENSG00000277476](https://www.ensembl.org/id/ENSG00000277476) | novel transcript | -1,03 |
| gene:ENSG00000277558 | [ENSG00000277558](https://www.ensembl.org/id/ENSG00000277558) | novel transcript, sense intronic to ITCH | 5,03 |
| gene:ENSG00000277831 | [ENSG00000277831](https://www.ensembl.org/id/ENSG00000277831) | novel transcript | 5,19 |
| gene:ENSG00000278383 | [ENSG00000278383](https://www.ensembl.org/id/ENSG00000278383) | novel transcript, antisense to ZNF337 | 2,90 |
| gene:ENSG00000278445 | [ENSG00000278445](https://www.ensembl.org/id/ENSG00000278445) | novel transcript | -6,19 |
| gene:ENSG00000278462 | [ENSG00000278462](https://www.ensembl.org/id/ENSG00000278462) | novel transcript, sense intronic to LRCH1 | -5,21 |
| gene:ENSG00000278464 | [ENSG00000278464](https://www.ensembl.org/id/ENSG00000278464) | novel transcript | -2,85 |
| gene:ENSG00000278532 | [ENSG00000278532](https://www.ensembl.org/id/ENSG00000278532) | novel transcript | -6,32 |
| gene:ENSG00000278920 | [ENSG00000278920](https://www.ensembl.org/id/ENSG00000278920) | novel transcript, sense overlapping SMTN | -3,91 |
| gene:ENSG00000279182 | [ENSG00000279182](https://www.ensembl.org/id/ENSG00000279182) | novel transcript, antisense to DENND6B | -3,11 |
| gene:ENSG00000279232 | [ENSG00000279232](https://www.ensembl.org/id/ENSG00000279232) | novel transcript, antisense to RGMB | -5,62 |
| gene:ENSG00000279668 | [ENSG00000279668](https://www.ensembl.org/id/ENSG00000279668) | novel transcript | -5,52 |
| gene:ENSG00000279686 | [ENSG00000279686](https://www.ensembl.org/id/ENSG00000279686) | novel transcript | -3,13 |
| gene:ENSG00000279855 | [ENSG00000279855](https://www.ensembl.org/id/ENSG00000279855) | novel transcript, antisense to protocadherin gamma cluster | -3,82 |
| gene:ENSG00000280029 | [ENSG00000280029](https://www.ensembl.org/id/ENSG00000280029) | novel transcript, antisense to PCDHB5, PCDHB6, PCDHB7, PCDHB8, PCDHB9, PCDHB10, PCDHB11, PCDHB12, PCDHB13, PCDHB14 and PCDHB16 | 5,23 |
| gene:ENSG00000280356 | [ENSG00000280356](https://www.ensembl.org/id/ENSG00000280356) | novel transcript | -4,98 |
| gene:ENSG00000280425 | [ENSG00000280425](https://www.ensembl.org/id/ENSG00000280425) | novel transcript, antisense to CDCP2 | -3,86 |
| gene:ENSG00000282033 | [ENSG00000282033](https://www.ensembl.org/id/ENSG00000282033) | novel transcript | -3,90 |
| gene:ENSG00000282572 | [ENSG00000282572](https://www.ensembl.org/id/ENSG00000282572) | family with sequence similarity 157 member D [Source:HGNC Symbol;Acc:HGNC:56252] | 5,78 |
| gene:ENSG00000283098 | [ENSG00000283098](https://www.ensembl.org/id/ENSG00000283098) | novel transcript | 2,28 |
| gene:ENSG00000283228 | [ENSG00000283228](https://www.ensembl.org/id/ENSG00000283228) | novel transcript | -7,21 |
| gene:ENSG00000283352 | [ENSG00000283352](https://www.ensembl.org/id/ENSG00000283352) | novel transcript | 2,15 |
| gene:ENSG00000283662 | [ENSG00000283662](https://www.ensembl.org/id/ENSG00000283662) | novel transcript | -7,01 |
| gene:ENSG00000283897 | [ENSG00000283897](https://www.ensembl.org/id/ENSG00000283897) | novel transcript | -3,90 |
| gene:ENSG00000284057 | [ENSG00000284057](https://www.ensembl.org/id/ENSG00000284057) | novel protein, C11orf54-MED17 readthrough | -3,15 |
| gene:ENSG00000284341 | [ENSG00000284341](https://www.ensembl.org/id/ENSG00000284341) | novel transcript | -3,87 |
| gene:ENSG00000284554 | [ENSG00000284554](https://www.ensembl.org/id/ENSG00000284554) | novel protein | -5,56 |
| gene:ENSG00000284644 | [ENSG00000284644](https://www.ensembl.org/id/ENSG00000284644) | novel transcript, antisense to OR2A1 and ARHGEF5 | -7,04 |
| gene:ENSG00000284685 | [ENSG00000284685](https://www.ensembl.org/id/ENSG00000284685) | novel transcript | 4,13 |
| gene:ENSG00000284716 | [ENSG00000284716](https://www.ensembl.org/id/ENSG00000284716) | novel transcript | -3,63 |
| gene:ENSG00000284906 | [ENSG00000284906](https://www.ensembl.org/id/ENSG00000284906) | novel protein | -2,59 |
| gene:ENSG00000284946 | [ENSG00000284946](https://www.ensembl.org/id/ENSG00000284946) | novel protein | 2,06 |
| gene:ENSG00000284969 | [ENSG00000284969](https://www.ensembl.org/id/ENSG00000284969) | novel protein | -2,08 |
| gene:ENSG00000284989 | [ENSG00000284989](https://www.ensembl.org/id/ENSG00000284989) | novel transcript | 5,30 |
| gene:ENSG00000285095 | [ENSG00000285095](https://www.ensembl.org/id/ENSG00000285095) | novel transcript | -5,62 |
| gene:ENSG00000285155 | [ENSG00000285155](https://www.ensembl.org/id/ENSG00000285155) | novel transcript | -6,92 |
| gene:ENSG00000285171 | [ENSG00000285171](https://www.ensembl.org/id/ENSG00000285171) | novel protein, IL2RG-CXorf65 readthrough | 3,48 |
| gene:ENSG00000285238 | [ENSG00000285238](https://www.ensembl.org/id/ENSG00000285238) | novel transcript | -2,86 |
| gene:ENSG00000285446 | [ENSG00000285446](https://www.ensembl.org/id/ENSG00000285446) | novel protein | -4,98 |
| gene:ENSG00000285523 | [ENSG00000285523](https://www.ensembl.org/id/ENSG00000285523) | novel transcript | 2,98 |
| gene:ENSG00000285555 | [ENSG00000285555](https://www.ensembl.org/id/ENSG00000285555) | novel transcript, antisense to CACNA1C | 4,87 |
| gene:ENSG00000285564 | [ENSG00000285564](https://www.ensembl.org/id/ENSG00000285564) | novel transcript | -5,05 |
| gene:ENSG00000285583 | [ENSG00000285583](https://www.ensembl.org/id/ENSG00000285583) | novel transcript, antisense to WDR83 | -5,24 |
| gene:ENSG00000285672 | [ENSG00000285672](https://www.ensembl.org/id/ENSG00000285672) | novel transcript | -5,45 |
| gene:ENSG00000285706 | [ENSG00000285706](https://www.ensembl.org/id/ENSG00000285706) | Novel transcript | -5,01 |
| gene:ENSG00000285747 | [ENSG00000285747](https://www.ensembl.org/id/ENSG00000285747) | novel transcript | -4,06 |
| gene:ENSG00000285849 | [ENSG00000285849](https://www.ensembl.org/id/ENSG00000285849) | novel transcript, antisense to ZNF184 | -3,64 |
| gene:ENSG00000285868 | [ENSG00000285868](https://www.ensembl.org/id/ENSG00000285868) | Novel protein | -2,71 |
| gene:ENSG00000285900 | [ENSG00000285900](https://www.ensembl.org/id/ENSG00000285900) | novel transcript, antisense to DMD | 4,90 |
| gene:ENSG00000286150 | [ENSG00000286150](https://www.ensembl.org/id/ENSG00000286150) | novel transcript, antisense to EMCN | -4,83 |
| gene:ENSG00000286231 | [ENSG00000286231](https://www.ensembl.org/id/ENSG00000286231) | novel protein | 7,26 |
| gene:ENSG00000286322 | [ENSG00000286322](https://www.ensembl.org/id/ENSG00000286322) | novel transcript | -3,74 |
| gene:ENSG00000286333 | [ENSG00000286333](https://www.ensembl.org/id/ENSG00000286333) | novel transcript, antisense to RGS6 | 4,70 |
| gene:ENSG00000286452 | [ENSG00000286452](https://www.ensembl.org/id/ENSG00000286452) | novel transcript | -3,80 |
| gene:ENSG00000286606 | [ENSG00000286606](https://www.ensembl.org/id/ENSG00000286606) | novel transcript | -3,86 |
| gene:ENSG00000286658 | [ENSG00000286658](https://www.ensembl.org/id/ENSG00000286658) | novel transcript, antisense to GRB10 | -5,79 |
| gene:ENSG00000286694 | [ENSG00000286694](https://www.ensembl.org/id/ENSG00000286694) | novel transcript | -5,55 |
| gene:ENSG00000286717 | [ENSG00000286717](https://www.ensembl.org/id/ENSG00000286717) | novel transcript, antisense to KCNJ6 | -4,03 |
| gene:ENSG00000286729 | [ENSG00000286729](https://www.ensembl.org/id/ENSG00000286729) | novel transcript, antisense to COPG1 | -2,26 |
| gene:ENSG00000286846 | [ENSG00000286846](https://www.ensembl.org/id/ENSG00000286846) | novel transcript | 4,73 |
| gene:ENSG00000286891 | [ENSG00000286891](https://www.ensembl.org/id/ENSG00000286891) | novel transcript | -3,63 |
| gene:ENSG00000286892 | [ENSG00000286892](https://www.ensembl.org/id/ENSG00000286892) | novel transcript | -5,86 |
| gene:ENSG00000286957 | [ENSG00000286957](https://www.ensembl.org/id/ENSG00000286957) | novel transcript | -3,91 |
| gene:ENSG00000287047 | [ENSG00000287047](https://www.ensembl.org/id/ENSG00000287047) | novel transcript, antisense to SORCS1 | -7,09 |
| gene:ENSG00000287201 | [ENSG00000287201](https://www.ensembl.org/id/ENSG00000287201) | novel transcript, antisense to ASAP1 | 5,04 |
| gene:ENSG00000287222 | [ENSG00000287222](https://www.ensembl.org/id/ENSG00000287222) | novel transcript, antisense to PUF60 | -1,92 |
| gene:ENSG00000287236 | [ENSG00000287236](https://www.ensembl.org/id/ENSG00000287236) | novel transcript, antisense to ANKRD54 | -2,90 |
| gene:ENSG00000287255 | [ENSG00000287255](https://www.ensembl.org/id/ENSG00000287255) | novel transcript, antisense to SLC8A1 | 2,28 |
| gene:ENSG00000287258 | [ENSG00000287258](https://www.ensembl.org/id/ENSG00000287258) | novel transcript, antisense to PKIB | -3,48 |
| gene:ENSG00000287283 | [ENSG00000287283](https://www.ensembl.org/id/ENSG00000287283) | novel transcript, antisense to LRRC7 | -5,33 |
| gene:ENSG00000287303 | [ENSG00000287303](https://www.ensembl.org/id/ENSG00000287303) | novel transcript, antisense to PDGFRL | -4,35 |
| gene:ENSG00000287340 | [ENSG00000287340](https://www.ensembl.org/id/ENSG00000287340) | novel transcript, antisense to RBFOX1 | -5,93 |
| gene:ENSG00000287483 | [ENSG00000287483](https://www.ensembl.org/id/ENSG00000287483) | novel transcript | -6,47 |
| gene:ENSG00000287490 | [ENSG00000287490](https://www.ensembl.org/id/ENSG00000287490) | novel transcript | -4,20 |
| gene:ENSG00000287534 | [ENSG00000287534](https://www.ensembl.org/id/ENSG00000287534) | novel transcript, sense intronic to RP11-231C18.3 | -3,94 |
| gene:ENSG00000287535 | [ENSG00000287535](https://www.ensembl.org/id/ENSG00000287535) | novel transcript | 7,23 |
| gene:ENSG00000287538 | [ENSG00000287538](https://www.ensembl.org/id/ENSG00000287538) | novel transcript | -5,07 |
| gene:ENSG00000287580 | [ENSG00000287580](https://www.ensembl.org/id/ENSG00000287580) | novel transcript, antisense to ZNF138 | -5,38 |
| gene:ENSG00000287647 | [ENSG00000287647](https://www.ensembl.org/id/ENSG00000287647) | novel transcript, antisense to AK5 | 2,97 |
| gene:ENSG00000287776 | [ENSG00000287776](https://www.ensembl.org/id/ENSG00000287776) | novel transcript | -5,81 |
| gene:ENSG00000287820 | [ENSG00000287820](https://www.ensembl.org/id/ENSG00000287820) | novel transcript | 4,76 |
| gene:ENSG00000287836 | [ENSG00000287836](https://www.ensembl.org/id/ENSG00000287836) | novel transcript | -5,10 |
| gene:ENSG00000287872 | [ENSG00000287872](https://www.ensembl.org/id/ENSG00000287872) | novel transcript | -3,48 |
| gene:ENSG00000288000 | [ENSG00000288000](https://www.ensembl.org/id/ENSG00000288000) | novel protein | -5,20 |
| gene:ENSG00000288473 | [ENSG00000288473](https://www.ensembl.org/id/ENSG00000288473) | novel protein | -1,90 |
| gene:ENSG00000288520 | [ENSG00000288520](https://www.ensembl.org/id/ENSG00000288520) | novel protein | 3,94 |
| gene:ENSG00000288684 | [ENSG00000288684](https://www.ensembl.org/id/ENSG00000288684) | novel protein | 8,29 |
| gene:ENSG00000288725 | [ENSG00000288725](https://www.ensembl.org/id/ENSG00000288725) | novel protein | -4,95 |
| gene:ENSG00000288754 | [ENSG00000288754](https://www.ensembl.org/id/ENSG00000288754) | novel transcript | 4,99 |
| gene:ENSG00000288779 | [ENSG00000288779](https://www.ensembl.org/id/ENSG00000288779) | novel transcript | 5,01 |
| gene:ENSG00000288794 | [ENSG00000288794](https://www.ensembl.org/id/ENSG00000288794) | novel transcript, sense intronic to LPP | 2,63 |
| gene:ENSG00000288806 | [ENSG00000288806](https://www.ensembl.org/id/ENSG00000288806) | novel transcript, antisense to MYLK | 2,25 |
| gene:ENSG00000288832 | [ENSG00000288832](https://www.ensembl.org/id/ENSG00000288832) | novel transcript, antisense to CNKSR2 | -3,37 |
| gene:ENSG00000288842 | [ENSG00000288842](https://www.ensembl.org/id/ENSG00000288842) | novel transcript | 4,23 |
| gene:ENSG00000288856 | [ENSG00000288856](https://www.ensembl.org/id/ENSG00000288856) | novel transcript, antisense to BCOR | 2,41 |
| gene:ENSG00000288882 | [ENSG00000288882](https://www.ensembl.org/id/ENSG00000288882) | novel transcript | 6,65 |
| gene:ENSG00000288894 | [ENSG00000288894](https://www.ensembl.org/id/ENSG00000288894) | novel protein | -6,21 |
| gene:ENSG00000288916 | [ENSG00000288916](https://www.ensembl.org/id/ENSG00000288916) | novel transcript | -4,95 |
| gene:ENSG00000288980 | [ENSG00000288980](https://www.ensembl.org/id/ENSG00000288980) | novel transcript | -6,73 |
| gene:ENSG00000288984 | [ENSG00000288984](https://www.ensembl.org/id/ENSG00000288984) | novel transcript, antisense to ITFG2 | 1,45 |
| gene:ENSG00000289001 | [ENSG00000289001](https://www.ensembl.org/id/ENSG00000289001) | novel transcript, antisense to SETD4 | -4,03 |
| gene:ENSG00000289080 | [ENSG00000289080](https://www.ensembl.org/id/ENSG00000289080) | novel transcript | 5,75 |
| gene:ENSG00000289085 | [ENSG00000289085](https://www.ensembl.org/id/ENSG00000289085) | novel transcript, sense intronic to CPEB4 | 5,53 |
| gene:ENSG00000289111 | [ENSG00000289111](https://www.ensembl.org/id/ENSG00000289111) | novel transcript | -4,71 |
| gene:ENSG00000289143 | [ENSG00000289143](https://www.ensembl.org/id/ENSG00000289143) | novel transcript | 5,67 |
| gene:ENSG00000289198 | [ENSG00000289198](https://www.ensembl.org/id/ENSG00000289198) | novel transcript | -4,07 |
| gene:ENSG00000289254 | [ENSG00000289254](https://www.ensembl.org/id/ENSG00000289254) | novel transcript, antisense to NMRK2 | -3,74 |
| gene:ENSG00000289258 | [ENSG00000289258](https://www.ensembl.org/id/ENSG00000289258) | novel protein | -2,78 |
| gene:ENSG00000289276 | [ENSG00000289276](https://www.ensembl.org/id/ENSG00000289276) | novel transcript, sense intronic to EFHC1and PAQR8 | -5,52 |
| gene:ENSG00000289299 | [ENSG00000289299](https://www.ensembl.org/id/ENSG00000289299) | novel transcript | -4,66 |
| gene:ENSG00000289302 | [ENSG00000289302](https://www.ensembl.org/id/ENSG00000289302) | novel transcript | -2,80 |
| gene:ENSG00000289365 | [ENSG00000289365](https://www.ensembl.org/id/ENSG00000289365) | novel transcript | -3,57 |
| gene:ENSG00000289366 | [ENSG00000289366](https://www.ensembl.org/id/ENSG00000289366) | novel transcript | 5,30 |
| gene:ENSG00000289386 | [ENSG00000289386](https://www.ensembl.org/id/ENSG00000289386) | novel transcript | -4,56 |
| gene:ENSG00000289397 | [ENSG00000289397](https://www.ensembl.org/id/ENSG00000289397) | Novel transcript | -4,08 |
| gene:ENSG00000289415 | [ENSG00000289415](https://www.ensembl.org/id/ENSG00000289415) | novel transcript, antisense to CES4A | -2,99 |
| gene:ENSG00000289438 | [ENSG00000289438](https://www.ensembl.org/id/ENSG00000289438) | novel transcript, antisense to DGKI | -5,43 |
| gene:ENSG00000289454 | [ENSG00000289454](https://www.ensembl.org/id/ENSG00000289454) | novel transcript | -6,03 |
| gene:ENSG00000289476 | [ENSG00000289476](https://www.ensembl.org/id/ENSG00000289476) | novel transcript | -3,68 |
| gene:ENSG00000289488 | [ENSG00000289488](https://www.ensembl.org/id/ENSG00000289488) | novel transcript, sense intronic to PKP4 | 5,12 |
| gene:ENSG00000289527 | [ENSG00000289527](https://www.ensembl.org/id/ENSG00000289527) | novel transcript, antisense to PRKG1 | -3,59 |
| gene:ENSG00000289609 | [ENSG00000289609](https://www.ensembl.org/id/ENSG00000289609) | novel transcript | -5,17 |
| gene:ENSG00000289615 | [ENSG00000289615](https://www.ensembl.org/id/ENSG00000289615) | novel transcript, antisense to CYP26B1 | -1,61 |
| gene:ENSG00000289621 | [ENSG00000289621](https://www.ensembl.org/id/ENSG00000289621) | novel transcript | 3,82 |
| gene:ENSG00000289626 | [ENSG00000289626](https://www.ensembl.org/id/ENSG00000289626) | novel transcript, sense intronic to ZFP36L1 | -2,42 |
| gene:ENSG00000289747 | [ENSG00000289747](https://www.ensembl.org/id/ENSG00000289747) | novel protein | 5,34 |
| gene:ENSG00000289768 | [ENSG00000289768](https://www.ensembl.org/id/ENSG00000289768) | novel protein similar to Fc fragment of IgG, low affinity III, receptor (CD16a) proteins | 6,16 |
| gene:ENSG00000289810 | [ENSG00000289810](https://www.ensembl.org/id/ENSG00000289810) | novel transcript | -2,80 |
| gene:ENSG00000289874 | [ENSG00000289874](https://www.ensembl.org/id/ENSG00000289874) | novel transcript | -3,52 |
| gene:ENSG00000289980 | [ENSG00000289980](https://www.ensembl.org/id/ENSG00000289980) | novel transcript | -5,42 |
| gene:ENSG00000289989 | [ENSG00000289989](https://www.ensembl.org/id/ENSG00000289989) | novel transcript, antisense to RHOBTB1 | -4,42 |
| gene:ENSG00000290007 | [ENSG00000290007](https://www.ensembl.org/id/ENSG00000290007) | novel transcript | -3,53 |
| gene:ENSG00000290025 | [ENSG00000290025](https://www.ensembl.org/id/ENSG00000290025) | novel transcript | -4,33 |
| gene:ENSG00000290043 | [ENSG00000290043](https://www.ensembl.org/id/ENSG00000290043) | novel transcript | -3,39 |
| gene:ENSG00000290044 | [ENSG00000290044](https://www.ensembl.org/id/ENSG00000290044) | novel transcript | -4,84 |
| gene:ENSG00000290242 | [ENSG00000290242](https://www.ensembl.org/id/ENSG00000290242) | novel transcript | -6,17 |
| gene:ENSG00000290563 | [ENSG00000290563](https://www.ensembl.org/id/ENSG00000290563) | novel transcript | 5,45 |
| gene:ENSG00000290573 | [ENSG00000290573](https://www.ensembl.org/id/ENSG00000290573) | novel transcript | -3,54 |
| gene:ENSG00000290579 | [ENSG00000290579](https://www.ensembl.org/id/ENSG00000290579) | novel transcript | -4,55 |
| gene:ENSG00000290585 | [ENSG00000290585](https://www.ensembl.org/id/ENSG00000290585) | novel transcript | 3,47 |
| gene:ENSG00000290594 | [ENSG00000290594](https://www.ensembl.org/id/ENSG00000290594) | novel transcript | 2,77 |
| gene:ENSG00000290659 | [ENSG00000290659](https://www.ensembl.org/id/ENSG00000290659) | novel transcript | -3,22 |
| gene:ENSG00000290690 | [ENSG00000290690](https://www.ensembl.org/id/ENSG00000290690) | novel transcript | 2,15 |
| gene:ENSG00000290769 | [ENSG00000290769](https://www.ensembl.org/id/ENSG00000290769) | novel transcript | -3,39 |
| gene:ENSG00000290786 | [ENSG00000290786](https://www.ensembl.org/id/ENSG00000290786) | novel transcript | 3,15 |
| gene:ENSG00000290816 | [ENSG00000290816](https://www.ensembl.org/id/ENSG00000290816) | novel transcript | -5,26 |
| gene:ENSG00000290858 | [ENSG00000290858](https://www.ensembl.org/id/ENSG00000290858) | novel transcript | -5,42 |
| gene:ENSG00000290896 | [ENSG00000290896](https://www.ensembl.org/id/ENSG00000290896) | novel transcript | 3,80 |
| gene:ENSG00000290980 | [ENSG00000290980](https://www.ensembl.org/id/ENSG00000290980) | novel transcript | -8,77 |
| gene:ENSG00000290989 | [ENSG00000290989](https://www.ensembl.org/id/ENSG00000290989) | novel transcript | 1,20 |
| gene:ENSG00000291048 | [ENSG00000291048](https://www.ensembl.org/id/ENSG00000291048) | novel transcript | 3,72 |
| gene:ENSG00000291073 | [ENSG00000291073](https://www.ensembl.org/id/ENSG00000291073) | novel transcript | 1,04 |
| gene:ENSG00000291144 | [ENSG00000291144](https://www.ensembl.org/id/ENSG00000291144) | novel transcript | 4,09 |
| gene:ENSG00000291182 | [ENSG00000291182](https://www.ensembl.org/id/ENSG00000291182) | novel transcript | 4,87 |
| gene:ENSG00000291198 | [ENSG00000291198](https://www.ensembl.org/id/ENSG00000291198) | novel transcript | -6,39 |
| gene:ENSG00000291233 | [ENSG00000291233](https://www.ensembl.org/id/ENSG00000291233) | novel transcript | -2,80 |
| gene:ENSG00000291234 | [ENSG00000291234](https://www.ensembl.org/id/ENSG00000291234) | novel transcript | 3,26 |
| gene:ENSG00000291236 | [ENSG00000291236](https://www.ensembl.org/id/ENSG00000291236) | novel transcript | 4,93 |
| gene:ENSG00000291250 | [ENSG00000291250](https://www.ensembl.org/id/ENSG00000291250) | novel transcript | -4,72 |
| gene:ENSG00000291261 | [ENSG00000291261](https://www.ensembl.org/id/ENSG00000291261) | novel transcript | -4,81 |
| GFPT2 | [ENSG00000131459](https://www.ensembl.org/id/ENSG00000131459) | glutamine-fructose-6-phosphate transaminase 2 [Source:HGNC Symbol;Acc:HGNC:4242] | -1,23 |
| GJA4 | [ENSG00000187513](https://www.ensembl.org/id/ENSG00000187513) | gap junction protein alpha 4 [Source:HGNC Symbol;Acc:HGNC:4278] | 1,10 |
| GJB5 | [ENSG00000189280](https://www.ensembl.org/id/ENSG00000189280) | gap junction protein beta 5 [Source:HGNC Symbol;Acc:HGNC:4287] | 3,54 |
| GJC3 | [ENSG00000176402](https://www.ensembl.org/id/ENSG00000176402) | gap junction protein gamma 3 [Source:HGNC Symbol;Acc:HGNC:17495] | -4,87 |
| GLDN | [ENSG00000186417](https://www.ensembl.org/id/ENSG00000186417) | gliomedin [Source:HGNC Symbol;Acc:HGNC:29514] | 1,12 |
| GLRX | [ENSG00000173221](https://www.ensembl.org/id/ENSG00000173221) | glutaredoxin [Source:HGNC Symbol;Acc:HGNC:4330] | -1,41 |
| GLYAT | [ENSG00000149124](https://www.ensembl.org/id/ENSG00000149124) | glycine-N-acyltransferase [Source:HGNC Symbol;Acc:HGNC:13734] | -2,78 |
| GLYATL1 | [ENSG00000166840](https://www.ensembl.org/id/ENSG00000166840) | glycine-N-acyltransferase like 1 [Source:HGNC Symbol;Acc:HGNC:30519] | -5,64 |
| GLYATL2 | [ENSG00000156689](https://www.ensembl.org/id/ENSG00000156689) | glycine-N-acyltransferase like 2 [Source:HGNC Symbol;Acc:HGNC:24178] | -2,78 |
| GNAT1 | [ENSG00000114349](https://www.ensembl.org/id/ENSG00000114349) | G protein subunit alpha transducin 1 [Source:HGNC Symbol;Acc:HGNC:4393] | -3,53 |
| GNLY | [ENSG00000115523](https://www.ensembl.org/id/ENSG00000115523) | granulysin [Source:HGNC Symbol;Acc:HGNC:4414] | 2,67 |
| GOLGA8H | [ENSG00000261794](https://www.ensembl.org/id/ENSG00000261794) | golgin A8 family member H [Source:HGNC Symbol;Acc:HGNC:37443] | -2,52 |
| GOLGA8K | [ENSG00000249931](https://www.ensembl.org/id/ENSG00000249931) | golgin A8 family member K [Source:HGNC Symbol;Acc:HGNC:38652] | -2,90 |
| GOLGA8S | [ENSG00000261739](https://www.ensembl.org/id/ENSG00000261739) | golgin A8 family member S [Source:HGNC Symbol;Acc:HGNC:44409] | -3,04 |
| GPAM | [ENSG00000119927](https://www.ensembl.org/id/ENSG00000119927) | glycerol-3-phosphate acyltransferase, mitochondrial [Source:HGNC Symbol;Acc:HGNC:24865] | -1,93 |
| GPD1 | [ENSG00000167588](https://www.ensembl.org/id/ENSG00000167588) | glycerol-3-phosphate dehydrogenase 1 [Source:HGNC Symbol;Acc:HGNC:4455] | -2,96 |
| GPM6B | [ENSG00000046653](https://www.ensembl.org/id/ENSG00000046653) | glycoprotein M6B [Source:HGNC Symbol;Acc:HGNC:4461] | -1,62 |
| GPR34 | [ENSG00000171659](https://www.ensembl.org/id/ENSG00000171659) | G protein-coupled receptor 34 [Source:HGNC Symbol;Acc:HGNC:4490] | -1,46 |
| GPR37L1 | [ENSG00000170075](https://www.ensembl.org/id/ENSG00000170075) | G protein-coupled receptor 37 like 1 [Source:HGNC Symbol;Acc:HGNC:14923] | -2,07 |
| GPR39 | [ENSG00000183840](https://www.ensembl.org/id/ENSG00000183840) | G protein-coupled receptor 39 [Source:HGNC Symbol;Acc:HGNC:4496] | -5,42 |
| GPSM1 | [ENSG00000160360](https://www.ensembl.org/id/ENSG00000160360) | G protein signaling modulator 1 [Source:HGNC Symbol;Acc:HGNC:17858] | -1,25 |
| GPT | [ENSG00000167701](https://www.ensembl.org/id/ENSG00000167701) | glutamic--pyruvic transaminase [Source:HGNC Symbol;Acc:HGNC:4552] | -2,88 |
| GPT2 | [ENSG00000166123](https://www.ensembl.org/id/ENSG00000166123) | glutamic--pyruvic transaminase 2 [Source:HGNC Symbol;Acc:HGNC:18062] | -3,57 |
| GRAP2 | [ENSG00000100351](https://www.ensembl.org/id/ENSG00000100351) | GRB2 related adaptor protein 2 [Source:HGNC Symbol;Acc:HGNC:4563] | 2,06 |
| GRB7 | [ENSG00000141738](https://www.ensembl.org/id/ENSG00000141738) | growth factor receptor bound protein 7 [Source:HGNC Symbol;Acc:HGNC:4567] | -2,23 |
| GRIA2 | [ENSG00000120251](https://www.ensembl.org/id/ENSG00000120251) | glutamate ionotropic receptor AMPA type subunit 2 [Source:HGNC Symbol;Acc:HGNC:4572] | -1,90 |
| GRIK1 | [ENSG00000171189](https://www.ensembl.org/id/ENSG00000171189) | glutamate ionotropic receptor kainate type subunit 1 [Source:HGNC Symbol;Acc:HGNC:4579] | -3,73 |
| GRK3 | [ENSG00000100077](https://www.ensembl.org/id/ENSG00000100077) | G protein-coupled receptor kinase 3 [Source:HGNC Symbol;Acc:HGNC:290] | -1,40 |
| GSDMC | [ENSG00000147697](https://www.ensembl.org/id/ENSG00000147697) | gasdermin C [Source:HGNC Symbol;Acc:HGNC:7151] | -7,05 |
| GXYLT2 | [ENSG00000172986](https://www.ensembl.org/id/ENSG00000172986) | glucoside xylosyltransferase 2 [Source:HGNC Symbol;Acc:HGNC:33383] | -1,48 |
| GYG2 | [ENSG00000056998](https://www.ensembl.org/id/ENSG00000056998) | glycogenin 2 [Source:HGNC Symbol;Acc:HGNC:4700] | -1,59 |
| GZF1 | [ENSG00000125812](https://www.ensembl.org/id/ENSG00000125812) | GDNF inducible zinc finger protein 1 [Source:HGNC Symbol;Acc:HGNC:15808] | 1,35 |
| GZMB | [ENSG00000100453](https://www.ensembl.org/id/ENSG00000100453) | granzyme B [Source:HGNC Symbol;Acc:HGNC:4709] | 2,90 |
| H19 | [ENSG00000130600](https://www.ensembl.org/id/ENSG00000130600) | H19 imprinted maternally expressed transcript [Source:HGNC Symbol;Acc:HGNC:4713] | -2,02 |
| HACD4 | [ENSG00000188921](https://www.ensembl.org/id/ENSG00000188921) | 3-hydroxyacyl-CoA dehydratase 4 [Source:HGNC Symbol;Acc:HGNC:20920] | 1,36 |
| HAP1 | [ENSG00000173805](https://www.ensembl.org/id/ENSG00000173805) | huntingtin associated protein 1 [Source:HGNC Symbol;Acc:HGNC:4812] | -3,93 |
| HAPLN2 | [ENSG00000132702](https://www.ensembl.org/id/ENSG00000132702) | hyaluronan and proteoglycan link protein 2 [Source:HGNC Symbol;Acc:HGNC:17410] | 2,02 |
| HAPLN4 | [ENSG00000187664](https://www.ensembl.org/id/ENSG00000187664) | hyaluronan and proteoglycan link protein 4 [Source:HGNC Symbol;Acc:HGNC:31357] | -4,87 |
| HBB | [ENSG00000244734](https://www.ensembl.org/id/ENSG00000244734) | hemoglobin subunit beta [Source:HGNC Symbol;Acc:HGNC:4827] | 3,55 |
| HBD | [ENSG00000223609](https://www.ensembl.org/id/ENSG00000223609) | hemoglobin subunit delta [Source:HGNC Symbol;Acc:HGNC:4829] | 4,72 |
| HBG2 | [ENSG00000196565](https://www.ensembl.org/id/ENSG00000196565) | hemoglobin subunit gamma 2 [Source:HGNC Symbol;Acc:HGNC:4832] | 11,10 |
| HBM | [ENSG00000206177](https://www.ensembl.org/id/ENSG00000206177) | hemoglobin subunit mu [Source:HGNC Symbol;Acc:HGNC:4826] | 3,92 |
| HBQ1 | [ENSG00000086506](https://www.ensembl.org/id/ENSG00000086506) | hemoglobin subunit theta 1 [Source:HGNC Symbol;Acc:HGNC:4833] | 4,20 |
| HCAR3 | [ENSG00000255398](https://www.ensembl.org/id/ENSG00000255398) | hydroxycarboxylic acid receptor 3 [Source:HGNC Symbol;Acc:HGNC:16824] | 3,03 |
| HCN1 | [ENSG00000164588](https://www.ensembl.org/id/ENSG00000164588) | hyperpolarization activated cyclic nucleotide gated potassium channel 1 [Source:HGNC Symbol;Acc:HGNC:4845] | -2,99 |
| HDAC10 | [ENSG00000100429](https://www.ensembl.org/id/ENSG00000100429) | histone deacetylase 10 [Source:HGNC Symbol;Acc:HGNC:18128] | -0,94 |
| HECW1 | [ENSG00000002746](https://www.ensembl.org/id/ENSG00000002746) | HECT, C2 and WW domain containing E3 ubiquitin protein ligase 1 [Source:HGNC Symbol;Acc:HGNC:22195] | -3,90 |
| HEPN1 | [ENSG00000221932](https://www.ensembl.org/id/ENSG00000221932) | hepatocellular carcinoma, down-regulated 1 [Source:HGNC Symbol;Acc:HGNC:34400] | -3,81 |
| HHATL | [ENSG00000010282](https://www.ensembl.org/id/ENSG00000010282) | hedgehog acyltransferase like [Source:HGNC Symbol;Acc:HGNC:13242] | -5,23 |
| HIP1R | [ENSG00000130787](https://www.ensembl.org/id/ENSG00000130787) | huntingtin interacting protein 1 related [Source:HGNC Symbol;Acc:HGNC:18415] | -1,04 |
| HJV | [ENSG00000168509](https://www.ensembl.org/id/ENSG00000168509) | hemojuvelin BMP co-receptor [Source:HGNC Symbol;Acc:HGNC:4887] | -8,12 |
| HK2 | [ENSG00000159399](https://www.ensembl.org/id/ENSG00000159399) | hexokinase 2 [Source:HGNC Symbol;Acc:HGNC:4923] | -1,76 |
| HK2-DT | [ENSG00000272711](https://www.ensembl.org/id/ENSG00000272711) | HK2 divergent transcript [Source:HGNC Symbol;Acc:HGNC:55204] | -2,12 |
| HK3 | [ENSG00000160883](https://www.ensembl.org/id/ENSG00000160883) | hexokinase 3 [Source:HGNC Symbol;Acc:HGNC:4925] | 2,51 |
| HLA-C | [ENSG00000204525](https://www.ensembl.org/id/ENSG00000204525) | major histocompatibility complex, class I, C [Source:HGNC Symbol;Acc:HGNC:4933] | 1,70 |
| HLA-DQA2 | [ENSG00000237541](https://www.ensembl.org/id/ENSG00000237541) | major histocompatibility complex, class II, DQ alpha 2 [Source:HGNC Symbol;Acc:HGNC:4943] | 3,43 |
| HLX-AS1 | [ENSG00000257551](https://www.ensembl.org/id/ENSG00000257551) | HLX antisense RNA 1 [Source:HGNC Symbol;Acc:HGNC:42509] | 3,17 |
| HMCN1 | [ENSG00000143341](https://www.ensembl.org/id/ENSG00000143341) | hemicentin 1 [Source:HGNC Symbol;Acc:HGNC:19194] | -1,87 |
| HMGA1 | [ENSG00000137309](https://www.ensembl.org/id/ENSG00000137309) | high mobility group AT-hook 1 [Source:HGNC Symbol;Acc:HGNC:5010] | -1,32 |
| HMGB1P1 | [ENSG00000124097](https://www.ensembl.org/id/ENSG00000124097) | high mobility group box 1 pseudogene 1 [Source:HGNC Symbol;Acc:HGNC:4993] | -3,53 |
| HMGCS2 | [ENSG00000134240](https://www.ensembl.org/id/ENSG00000134240) | 3-hydroxy-3-methylglutaryl-CoA synthase 2 [Source:HGNC Symbol;Acc:HGNC:5008] | -9,21 |
| HMX1 | [ENSG00000215612](https://www.ensembl.org/id/ENSG00000215612) | H6 family homeobox 1 [Source:HGNC Symbol;Acc:HGNC:5017] | -5,24 |
| HOOK1 | [ENSG00000134709](https://www.ensembl.org/id/ENSG00000134709) | hook microtubule tethering protein 1 [Source:HGNC Symbol;Acc:HGNC:19884] | -2,81 |
| HOOK2 | [ENSG00000095066](https://www.ensembl.org/id/ENSG00000095066) | hook microtubule tethering protein 2 [Source:HGNC Symbol;Acc:HGNC:19885] | -1,23 |
| HOXA5 | [ENSG00000106004](https://www.ensembl.org/id/ENSG00000106004) | homeobox A5 [Source:HGNC Symbol;Acc:HGNC:5106] | 1,11 |
| HOXB-AS3 | [ENSG00000233101](https://www.ensembl.org/id/ENSG00000233101) | HOXB cluster antisense RNA 3 [Source:HGNC Symbol;Acc:HGNC:40283] | -3,34 |
| HP | [ENSG00000257017](https://www.ensembl.org/id/ENSG00000257017) | haptoglobin [Source:HGNC Symbol;Acc:HGNC:5141] | -6,47 |
| HPN | [ENSG00000105707](https://www.ensembl.org/id/ENSG00000105707) | hepsin [Source:HGNC Symbol;Acc:HGNC:5155] | -2,45 |
| HRH1 | [ENSG00000196639](https://www.ensembl.org/id/ENSG00000196639) | histamine receptor H1 [Source:HGNC Symbol;Acc:HGNC:5182] | -1,28 |
| HS6ST2 | [ENSG00000171004](https://www.ensembl.org/id/ENSG00000171004) | heparan sulfate 6-O-sulfotransferase 2 [Source:HGNC Symbol;Acc:HGNC:19133] | -3,84 |
| HSFX2 | [ENSG00000268738](https://www.ensembl.org/id/ENSG00000268738) | heat shock transcription factor family, X-linked 2 [Source:HGNC Symbol;Acc:HGNC:32701] | -8,25 |
| HSFX4 | [ENSG00000283463](https://www.ensembl.org/id/ENSG00000283463) | heat shock transcription factor family, X-linked member 4 [Source:HGNC Symbol;Acc:HGNC:52398] | 8,92 |
| HSH2D | [ENSG00000196684](https://www.ensembl.org/id/ENSG00000196684) | hematopoietic SH2 domain containing [Source:HGNC Symbol;Acc:HGNC:24920] | 3,07 |
| HYAL4 | [ENSG00000106302](https://www.ensembl.org/id/ENSG00000106302) | hyaluronidase 4 [Source:HGNC Symbol;Acc:HGNC:5323] | -4,37 |
| ID1 | [ENSG00000125968](https://www.ensembl.org/id/ENSG00000125968) | inhibitor of DNA binding 1 [Source:HGNC Symbol;Acc:HGNC:5360] | 1,18 |
| IDI2 | [ENSG00000148377](https://www.ensembl.org/id/ENSG00000148377) | isopentenyl-diphosphate delta isomerase 2 [Source:HGNC Symbol;Acc:HGNC:23487] | -12,12 |
| IDO1 | [ENSG00000131203](https://www.ensembl.org/id/ENSG00000131203) | indoleamine 2,3-dioxygenase 1 [Source:HGNC Symbol;Acc:HGNC:6059] | -3,46 |
| IER5L-AS1 | [ENSG00000204055](https://www.ensembl.org/id/ENSG00000204055) | IER5L antisense RNA 1 [Source:HGNC Symbol;Acc:HGNC:55825] | -2,65 |
| IFIT1B | [ENSG00000204010](https://www.ensembl.org/id/ENSG00000204010) | interferon induced protein with tetratricopeptide repeats 1B [Source:HGNC Symbol;Acc:HGNC:23442] | 3,95 |
| IFIT3 | [ENSG00000119917](https://www.ensembl.org/id/ENSG00000119917) | interferon induced protein with tetratricopeptide repeats 3 [Source:HGNC Symbol;Acc:HGNC:5411] | -1,38 |
| IFNG-AS1 | [ENSG00000255733](https://www.ensembl.org/id/ENSG00000255733) | IFNG antisense RNA 1 [Source:HGNC Symbol;Acc:HGNC:43910] | 2,82 |
| IGDCC4 | [ENSG00000103742](https://www.ensembl.org/id/ENSG00000103742) | immunoglobulin superfamily DCC subclass member 4 [Source:HGNC Symbol;Acc:HGNC:13770] | -2,30 |
| IGFN1 | [ENSG00000163395](https://www.ensembl.org/id/ENSG00000163395) | immunoglobulin like and fibronectin type III domain containing 1 [Source:HGNC Symbol;Acc:HGNC:24607] | -7,69 |
| IGSF9 | [ENSG00000085552](https://www.ensembl.org/id/ENSG00000085552) | immunoglobulin superfamily member 9 [Source:HGNC Symbol;Acc:HGNC:18132] | -4,69 |
| IL17D | [ENSG00000172458](https://www.ensembl.org/id/ENSG00000172458) | interleukin 17D [Source:HGNC Symbol;Acc:HGNC:5984] | -2,65 |
| IL18 | [ENSG00000150782](https://www.ensembl.org/id/ENSG00000150782) | interleukin 18 [Source:HGNC Symbol;Acc:HGNC:5986] | -2,10 |
| IL18RAP | [ENSG00000115607](https://www.ensembl.org/id/ENSG00000115607) | interleukin 18 receptor accessory protein [Source:HGNC Symbol;Acc:HGNC:5989] | 2,41 |
| IL1B | [ENSG00000125538](https://www.ensembl.org/id/ENSG00000125538) | interleukin 1 beta [Source:HGNC Symbol;Acc:HGNC:5992] | 2,68 |
| IL1R2 | [ENSG00000115590](https://www.ensembl.org/id/ENSG00000115590) | interleukin 1 receptor type 2 [Source:HGNC Symbol;Acc:HGNC:5994] | 4,19 |
| IL1RN | [ENSG00000136689](https://www.ensembl.org/id/ENSG00000136689) | interleukin 1 receptor antagonist [Source:HGNC Symbol;Acc:HGNC:6000] | 2,81 |
| IL21R | [ENSG00000103522](https://www.ensembl.org/id/ENSG00000103522) | interleukin 21 receptor [Source:HGNC Symbol;Acc:HGNC:6006] | 2,80 |
| IL31RA | [ENSG00000164509](https://www.ensembl.org/id/ENSG00000164509) | interleukin 31 receptor A [Source:HGNC Symbol;Acc:HGNC:18969] | -6,17 |
| IL32 | [ENSG00000008517](https://www.ensembl.org/id/ENSG00000008517) | interleukin 32 [Source:HGNC Symbol;Acc:HGNC:16830] | -1,94 |
| IL6ST-DT | [ENSG00000227908](https://www.ensembl.org/id/ENSG00000227908) | IL6ST divergent transcript [Source:HGNC Symbol;Acc:HGNC:55804] | -2,55 |
| IL9R | [ENSG00000124334](https://www.ensembl.org/id/ENSG00000124334) | interleukin 9 receptor [Source:HGNC Symbol;Acc:HGNC:6030] | -3,35 |
| ILDR2 | [ENSG00000143195](https://www.ensembl.org/id/ENSG00000143195) | immunoglobulin like domain containing receptor 2 [Source:HGNC Symbol;Acc:HGNC:18131] | -2,39 |
| INHBB | [ENSG00000163083](https://www.ensembl.org/id/ENSG00000163083) | inhibin subunit beta B [Source:HGNC Symbol;Acc:HGNC:6067] | -1,14 |
| INHBC | [ENSG00000175189](https://www.ensembl.org/id/ENSG00000175189) | inhibin subunit beta C [Source:HGNC Symbol;Acc:HGNC:6068] | -3,70 |
| IQANK1 | [ENSG00000203499](https://www.ensembl.org/id/ENSG00000203499) | IQ motif and ankyrin repeat containing 1 [Source:HGNC Symbol;Acc:HGNC:49576] | -3,74 |
| IQSEC3-AS1 | [ENSG00000256540](https://www.ensembl.org/id/ENSG00000256540) | IQSEC3 antisense RNA 1 [Source:HGNC Symbol;Acc:HGNC:56002] | -3,65 |
| IRX6 | [ENSG00000159387](https://www.ensembl.org/id/ENSG00000159387) | iroquois homeobox 6 [Source:HGNC Symbol;Acc:HGNC:14675] | -2,64 |
| ISM2 | [ENSG00000100593](https://www.ensembl.org/id/ENSG00000100593) | isthmin 2 [Source:HGNC Symbol;Acc:HGNC:23176] | -5,83 |
| ITGA2-AS1 | [ENSG00000249899](https://www.ensembl.org/id/ENSG00000249899) | ITGA2 antisense RNA 1 [Source:HGNC Symbol;Acc:HGNC:40306] | 4,50 |
| ITGA2B | [ENSG00000005961](https://www.ensembl.org/id/ENSG00000005961) | integrin subunit alpha 2b [Source:HGNC Symbol;Acc:HGNC:6138] | 2,76 |
| ITGAX | [ENSG00000140678](https://www.ensembl.org/id/ENSG00000140678) | integrin subunit alpha X [Source:HGNC Symbol;Acc:HGNC:6152] | 3,45 |
| ITGB6 | [ENSG00000115221](https://www.ensembl.org/id/ENSG00000115221) | integrin subunit beta 6 [Source:HGNC Symbol;Acc:HGNC:6161] | -11,05 |
| ITIH1 | [ENSG00000055957](https://www.ensembl.org/id/ENSG00000055957) | inter-alpha-trypsin inhibitor heavy chain 1 [Source:HGNC Symbol;Acc:HGNC:6166] | 3,33 |
| ITIH3 | [ENSG00000162267](https://www.ensembl.org/id/ENSG00000162267) | inter-alpha-trypsin inhibitor heavy chain 3 [Source:HGNC Symbol;Acc:HGNC:6168] | 2,38 |
| ITLN2 | [ENSG00000158764](https://www.ensembl.org/id/ENSG00000158764) | intelectin 2 [Source:HGNC Symbol;Acc:HGNC:20599] | -2,54 |
| JAML | [ENSG00000160593](https://www.ensembl.org/id/ENSG00000160593) | junction adhesion molecule like [Source:HGNC Symbol;Acc:HGNC:19084] | 2,69 |
| JPH1 | [ENSG00000104369](https://www.ensembl.org/id/ENSG00000104369) | junctophilin 1 [Source:HGNC Symbol;Acc:HGNC:14201] | -5,40 |
| JPH3 | [ENSG00000154118](https://www.ensembl.org/id/ENSG00000154118) | junctophilin 3 [Source:HGNC Symbol;Acc:HGNC:14203] | -2,50 |
| JSRP1 | [ENSG00000167476](https://www.ensembl.org/id/ENSG00000167476) | junctional sarcoplasmic reticulum protein 1 [Source:HGNC Symbol;Acc:HGNC:24963] | -7,17 |
| KANK4 | [ENSG00000132854](https://www.ensembl.org/id/ENSG00000132854) | KN motif and ankyrin repeat domains 4 [Source:HGNC Symbol;Acc:HGNC:27263] | -3,17 |
| KCNA7 | [ENSG00000104848](https://www.ensembl.org/id/ENSG00000104848) | potassium voltage-gated channel subfamily A member 7 [Source:HGNC Symbol;Acc:HGNC:6226] | -10,89 |
| KCNC2 | [ENSG00000166006](https://www.ensembl.org/id/ENSG00000166006) | potassium voltage-gated channel subfamily C member 2 [Source:HGNC Symbol;Acc:HGNC:6234] | -6,05 |
| KCNE4 | [ENSG00000152049](https://www.ensembl.org/id/ENSG00000152049) | potassium voltage-gated channel subfamily E regulatory subunit 4 [Source:HGNC Symbol;Acc:HGNC:6244] | 1,22 |
| KCNE5 | [ENSG00000176076](https://www.ensembl.org/id/ENSG00000176076) | potassium voltage-gated channel subfamily E regulatory subunit 5 [Source:HGNC Symbol;Acc:HGNC:6241] | -4,39 |
| KCNG1 | [ENSG00000026559](https://www.ensembl.org/id/ENSG00000026559) | potassium voltage-gated channel modifier subfamily G member 1 [Source:HGNC Symbol;Acc:HGNC:6248] | -4,03 |
| KCNH5 | [ENSG00000140015](https://www.ensembl.org/id/ENSG00000140015) | potassium voltage-gated channel subfamily H member 5 [Source:HGNC Symbol;Acc:HGNC:6254] | -6,10 |
| KCNIP2 | [ENSG00000120049](https://www.ensembl.org/id/ENSG00000120049) | potassium voltage-gated channel interacting protein 2 [Source:HGNC Symbol;Acc:HGNC:15522] | -2,94 |
| KCNJ1 | [ENSG00000151704](https://www.ensembl.org/id/ENSG00000151704) | potassium inwardly rectifying channel subfamily J member 1 [Source:HGNC Symbol;Acc:HGNC:6255] | -2,45 |
| KCNJ11 | [ENSG00000187486](https://www.ensembl.org/id/ENSG00000187486) | potassium inwardly rectifying channel subfamily J member 11 [Source:HGNC Symbol;Acc:HGNC:6257] | -2,10 |
| KCNJ12 | [ENSG00000184185](https://www.ensembl.org/id/ENSG00000184185) | potassium inwardly rectifying channel subfamily J member 12 [Source:HGNC Symbol;Acc:HGNC:6258] | -2,20 |
| KCNJ15 | [ENSG00000157551](https://www.ensembl.org/id/ENSG00000157551) | potassium inwardly rectifying channel subfamily J member 15 [Source:HGNC Symbol;Acc:HGNC:6261] | 2,60 |
| KCNJ8 | [ENSG00000121361](https://www.ensembl.org/id/ENSG00000121361) | potassium inwardly rectifying channel subfamily J member 8 [Source:HGNC Symbol;Acc:HGNC:6269] | 1,23 |
| KCNK6 | [ENSG00000099337](https://www.ensembl.org/id/ENSG00000099337) | potassium two pore domain channel subfamily K member 6 [Source:HGNC Symbol;Acc:HGNC:6281] | -1,56 |
| KCP | [ENSG00000135253](https://www.ensembl.org/id/ENSG00000135253) | kielin cysteine rich BMP regulator [Source:HGNC Symbol;Acc:HGNC:17585] | -1,65 |
| KCTD16 | [ENSG00000183775](https://www.ensembl.org/id/ENSG00000183775) | potassium channel tetramerization domain containing 16 [Source:HGNC Symbol;Acc:HGNC:29244] | -2,12 |
| KHDC1L | [ENSG00000256980](https://www.ensembl.org/id/ENSG00000256980) | KH domain containing 1 like [Source:HGNC Symbol;Acc:HGNC:37274] | -5,91 |
| KIAA1549 | [ENSG00000122778](https://www.ensembl.org/id/ENSG00000122778) | KIAA1549 [Source:HGNC Symbol;Acc:HGNC:22219] | -2,19 |
| KIAA1671-AS1 | [ENSG00000203280](https://www.ensembl.org/id/ENSG00000203280) | KIAA1671 antisense RNA 1 [Source:HGNC Symbol;Acc:HGNC:41238] | -3,42 |
| KIAA1755 | [ENSG00000149633](https://www.ensembl.org/id/ENSG00000149633) | KIAA1755 [Source:HGNC Symbol;Acc:HGNC:29372] | -1,66 |
| KIF1A | [ENSG00000130294](https://www.ensembl.org/id/ENSG00000130294) | kinesin family member 1A [Source:HGNC Symbol;Acc:HGNC:888] | -3,93 |
| KIF25-AS1 | [ENSG00000229921](https://www.ensembl.org/id/ENSG00000229921) | KIF25 antisense RNA 1 [Source:HGNC Symbol;Acc:HGNC:20953] | -5,45 |
| KIR3DL2 | [ENSG00000240403](https://www.ensembl.org/id/ENSG00000240403) | killer cell immunoglobulin like receptor, three Ig domains and long cytoplasmic tail 2 [Source:HGNC Symbol;Acc:HGNC:6339] | 5,24 |
| KLB | [ENSG00000134962](https://www.ensembl.org/id/ENSG00000134962) | klotho beta [Source:HGNC Symbol;Acc:HGNC:15527] | -2,75 |
| KLC3 | [ENSG00000104892](https://www.ensembl.org/id/ENSG00000104892) | kinesin light chain 3 [Source:HGNC Symbol;Acc:HGNC:20717] | 5,14 |
| KLC4-AS1 | [ENSG00000272170](https://www.ensembl.org/id/ENSG00000272170) | KLC4 antisense RNA 1 [Source:HGNC Symbol;Acc:HGNC:55228] | -3,87 |
| KLF14 | [ENSG00000266265](https://www.ensembl.org/id/ENSG00000266265) | KLF transcription factor 14 [Source:HGNC Symbol;Acc:HGNC:23025] | -2,97 |
| KLF17 | [ENSG00000171872](https://www.ensembl.org/id/ENSG00000171872) | KLF transcription factor 17 [Source:HGNC Symbol;Acc:HGNC:18830] | -3,79 |
| KLF5 | [ENSG00000102554](https://www.ensembl.org/id/ENSG00000102554) | KLF transcription factor 5 [Source:HGNC Symbol;Acc:HGNC:6349] | -1,83 |
| KLHL31 | [ENSG00000124743](https://www.ensembl.org/id/ENSG00000124743) | kelch like family member 31 [Source:HGNC Symbol;Acc:HGNC:21353] | -6,98 |
| KLHL33 | [ENSG00000185271](https://www.ensembl.org/id/ENSG00000185271) | kelch like family member 33 [Source:HGNC Symbol;Acc:HGNC:31952] | -3,52 |
| KLHL34 | [ENSG00000185915](https://www.ensembl.org/id/ENSG00000185915) | kelch like family member 34 [Source:HGNC Symbol;Acc:HGNC:26634] | -7,25 |
| KLHL40 | [ENSG00000157119](https://www.ensembl.org/id/ENSG00000157119) | kelch like family member 40 [Source:HGNC Symbol;Acc:HGNC:30372] | -6,49 |
| KLHL41 | [ENSG00000239474](https://www.ensembl.org/id/ENSG00000239474) | kelch like family member 41 [Source:HGNC Symbol;Acc:HGNC:16905] | -7,99 |
| KLK10 | [ENSG00000129451](https://www.ensembl.org/id/ENSG00000129451) | kallikrein related peptidase 10 [Source:HGNC Symbol;Acc:HGNC:6358] | -5,05 |
| KLK11 | [ENSG00000167757](https://www.ensembl.org/id/ENSG00000167757) | kallikrein related peptidase 11 [Source:HGNC Symbol;Acc:HGNC:6359] | -7,33 |
| KLK13 | [ENSG00000167759](https://www.ensembl.org/id/ENSG00000167759) | kallikrein related peptidase 13 [Source:HGNC Symbol;Acc:HGNC:6361] | -4,95 |
| KLK5 | [ENSG00000167754](https://www.ensembl.org/id/ENSG00000167754) | kallikrein related peptidase 5 [Source:HGNC Symbol;Acc:HGNC:6366] | -4,82 |
| KLK6 | [ENSG00000167755](https://www.ensembl.org/id/ENSG00000167755) | kallikrein related peptidase 6 [Source:HGNC Symbol;Acc:HGNC:6367] | -5,22 |
| KLK8 | [ENSG00000129455](https://www.ensembl.org/id/ENSG00000129455) | kallikrein related peptidase 8 [Source:HGNC Symbol;Acc:HGNC:6369] | -6,34 |
| KMT2E-AS1 | [ENSG00000239569](https://www.ensembl.org/id/ENSG00000239569) | KMT2E antisense RNA 1 [Source:HGNC Symbol;Acc:HGNC:40845] | -1,39 |
| KRT1 | [ENSG00000167768](https://www.ensembl.org/id/ENSG00000167768) | keratin 1 [Source:HGNC Symbol;Acc:HGNC:6412] | 6,04 |
| KRT13 | [ENSG00000171401](https://www.ensembl.org/id/ENSG00000171401) | keratin 13 [Source:HGNC Symbol;Acc:HGNC:6415] | -4,91 |
| KRT14 | [ENSG00000186847](https://www.ensembl.org/id/ENSG00000186847) | keratin 14 [Source:HGNC Symbol;Acc:HGNC:6416] | -2,92 |
| KRT15 | [ENSG00000171346](https://www.ensembl.org/id/ENSG00000171346) | keratin 15 [Source:HGNC Symbol;Acc:HGNC:6421] | -5,93 |
| KRT18 | [ENSG00000111057](https://www.ensembl.org/id/ENSG00000111057) | keratin 18 [Source:HGNC Symbol;Acc:HGNC:6430] | -2,11 |
| KRT19 | [ENSG00000171345](https://www.ensembl.org/id/ENSG00000171345) | keratin 19 [Source:HGNC Symbol;Acc:HGNC:6436] | -4,10 |
| KRT23 | [ENSG00000108244](https://www.ensembl.org/id/ENSG00000108244) | keratin 23 [Source:HGNC Symbol;Acc:HGNC:6438] | 5,22 |
| KRT7 | [ENSG00000135480](https://www.ensembl.org/id/ENSG00000135480) | keratin 7 [Source:HGNC Symbol;Acc:HGNC:6445] | -3,12 |
| KRT8 | [ENSG00000170421](https://www.ensembl.org/id/ENSG00000170421) | keratin 8 [Source:HGNC Symbol;Acc:HGNC:6446] | -2,60 |
| KRTAP19-3 | [ENSG00000244025](https://www.ensembl.org/id/ENSG00000244025) | keratin associated protein 19-3 [Source:HGNC Symbol;Acc:HGNC:18938] | -4,95 |
| KRTAP5-10 | [ENSG00000204572](https://www.ensembl.org/id/ENSG00000204572) | keratin associated protein 5-10 [Source:HGNC Symbol;Acc:HGNC:23605] | 3,58 |
| KRTAP5-8 | [ENSG00000241233](https://www.ensembl.org/id/ENSG00000241233) | keratin associated protein 5-8 [Source:HGNC Symbol;Acc:HGNC:23603] | -3,35 |
| LAD1 | [ENSG00000159166](https://www.ensembl.org/id/ENSG00000159166) | ladinin 1 [Source:HGNC Symbol;Acc:HGNC:6472] | -6,86 |
| LAG3 | [ENSG00000089692](https://www.ensembl.org/id/ENSG00000089692) | lymphocyte activating 3 [Source:HGNC Symbol;Acc:HGNC:6476] | -1,79 |
| LAMB1 | [ENSG00000091136](https://www.ensembl.org/id/ENSG00000091136) | laminin subunit beta 1 [Source:HGNC Symbol;Acc:HGNC:6486] | -1,37 |
| LAMC3 | [ENSG00000050555](https://www.ensembl.org/id/ENSG00000050555) | laminin subunit gamma 3 [Source:HGNC Symbol;Acc:HGNC:6494] | -1,66 |
| LANCL1-AS1 | [ENSG00000234281](https://www.ensembl.org/id/ENSG00000234281) | LANCL1 antisense RNA 1 [Source:HGNC Symbol;Acc:HGNC:50727] | -7,36 |
| LBR | [ENSG00000143815](https://www.ensembl.org/id/ENSG00000143815) | lamin B receptor [Source:HGNC Symbol;Acc:HGNC:6518] | 1,10 |
| LBX1 | [ENSG00000138136](https://www.ensembl.org/id/ENSG00000138136) | ladybird homeobox 1 [Source:HGNC Symbol;Acc:HGNC:16960] | -7,33 |
| LBX1-AS1 | [ENSG00000227128](https://www.ensembl.org/id/ENSG00000227128) | LBX1 antisense RNA 1 [Source:HGNC Symbol;Acc:HGNC:48678] | -7,88 |
| LCN10 | [ENSG00000187922](https://www.ensembl.org/id/ENSG00000187922) | lipocalin 10 [Source:HGNC Symbol;Acc:HGNC:20892] | -1,94 |
| LCN12 | [ENSG00000184925](https://www.ensembl.org/id/ENSG00000184925) | lipocalin 12 [Source:HGNC Symbol;Acc:HGNC:28733] | -3,14 |
| LCN2 | [ENSG00000148346](https://www.ensembl.org/id/ENSG00000148346) | lipocalin 2 [Source:HGNC Symbol;Acc:HGNC:6526] | 3,33 |
| LCN8 | [ENSG00000204001](https://www.ensembl.org/id/ENSG00000204001) | lipocalin 8 [Source:HGNC Symbol;Acc:HGNC:27038] | -6,86 |
| LCNL1 | [ENSG00000214402](https://www.ensembl.org/id/ENSG00000214402) | lipocalin like 1 [Source:HGNC Symbol;Acc:HGNC:34436] | -2,00 |
| LCP1 | [ENSG00000136167](https://www.ensembl.org/id/ENSG00000136167) | lymphocyte cytosolic protein 1 [Source:HGNC Symbol;Acc:HGNC:6528] | 2,45 |
| LGALS2 | [ENSG00000100079](https://www.ensembl.org/id/ENSG00000100079) | galectin 2 [Source:HGNC Symbol;Acc:HGNC:6562] | -3,24 |
| LHX2 | [ENSG00000106689](https://www.ensembl.org/id/ENSG00000106689) | LIM homeobox 2 [Source:HGNC Symbol;Acc:HGNC:6594] | -4,90 |
| LIF-AS2 | [ENSG00000268812](https://www.ensembl.org/id/ENSG00000268812) | LIF antisense RNA 2 [Source:HGNC Symbol;Acc:HGNC:28298] | -4,75 |
| LILRA1 | [ENSG00000104974](https://www.ensembl.org/id/ENSG00000104974) | leukocyte immunoglobulin like receptor A1 [Source:HGNC Symbol;Acc:HGNC:6602] | 3,22 |
| LILRA2 | [ENSG00000239998](https://www.ensembl.org/id/ENSG00000239998) | leukocyte immunoglobulin like receptor A2 [Source:HGNC Symbol;Acc:HGNC:6603] | 3,43 |
| LILRA5 | [ENSG00000187116](https://www.ensembl.org/id/ENSG00000187116) | leukocyte immunoglobulin like receptor A5 [Source:HGNC Symbol;Acc:HGNC:16309] | 4,60 |
| LILRB3 | [ENSG00000204577](https://www.ensembl.org/id/ENSG00000204577) | leukocyte immunoglobulin like receptor B3 [Source:HGNC Symbol;Acc:HGNC:6607] | 2,73 |
| LINC00243 | [ENSG00000214894](https://www.ensembl.org/id/ENSG00000214894) | long intergenic non-protein coding RNA 243 [Source:HGNC Symbol;Acc:HGNC:30956] | 5,40 |
| LINC00545 | [ENSG00000236094](https://www.ensembl.org/id/ENSG00000236094) | long intergenic non-protein coding RNA 545 [Source:HGNC Symbol;Acc:HGNC:43680] | 5,41 |
| LINC00570 | [ENSG00000224177](https://www.ensembl.org/id/ENSG00000224177) | long intergenic non-protein coding RNA 570 [Source:HGNC Symbol;Acc:HGNC:43717] | 2,32 |
| LINC00645 | [ENSG00000258548](https://www.ensembl.org/id/ENSG00000258548) | long intergenic non-protein coding RNA 645 [Source:HGNC Symbol;Acc:HGNC:44299] | -3,99 |
| LINC00664 | [ENSG00000268658](https://www.ensembl.org/id/ENSG00000268658) | long intergenic non-protein coding RNA 664 [Source:HGNC Symbol;Acc:HGNC:44319] | 2,86 |
| LINC00880 | [ENSG00000243629](https://www.ensembl.org/id/ENSG00000243629) | long intergenic non-protein coding RNA 880 [Source:HGNC Symbol;Acc:HGNC:27948] | -5,16 |
| LINC00900 | [ENSG00000246100](https://www.ensembl.org/id/ENSG00000246100) | long intergenic non-protein coding RNA 900 [Source:HGNC Symbol;Acc:HGNC:27444] | -3,10 |
| LINC00942 | [ENSG00000249628](https://www.ensembl.org/id/ENSG00000249628) | long intergenic non-protein coding RNA 942 [Source:HGNC Symbol;Acc:HGNC:48636] | -3,54 |
| LINC01016 | [ENSG00000249346](https://www.ensembl.org/id/ENSG00000249346) | long intergenic non-protein coding RNA 1016 [Source:HGNC Symbol;Acc:HGNC:48991] | 3,06 |
| LINC01121 | [ENSG00000205054](https://www.ensembl.org/id/ENSG00000205054) | long intergenic non-protein coding RNA 1121 [Source:HGNC Symbol;Acc:HGNC:49266] | -3,67 |
| LINC01124 | [ENSG00000222033](https://www.ensembl.org/id/ENSG00000222033) | long intergenic non-protein coding RNA 1124 [Source:HGNC Symbol;Acc:HGNC:49270] | 2,64 |
| LINC01140 | [ENSG00000267272](https://www.ensembl.org/id/ENSG00000267272) | long intergenic non-protein coding RNA 1140 [Source:HGNC Symbol;Acc:HGNC:27922] | -1,94 |
| LINC01168 | [ENSG00000240707](https://www.ensembl.org/id/ENSG00000240707) | long intergenic non-protein coding RNA 1168 [Source:HGNC Symbol;Acc:HGNC:49537] | -4,64 |
| LINC01230 | [ENSG00000281769](https://www.ensembl.org/id/ENSG00000281769) | long intergenic non-protein coding RNA 1230 [Source:HGNC Symbol;Acc:HGNC:49686] | -3,86 |
| LINC01297 | [ENSG00000274827](https://www.ensembl.org/id/ENSG00000274827) | long intergenic non-protein coding RNA 1297 [Source:HGNC Symbol;Acc:HGNC:50443] | -5,22 |
| LINC01322 | [ENSG00000244128](https://www.ensembl.org/id/ENSG00000244128) | long intergenic non-protein coding RNA 1322 [Source:HGNC Symbol;Acc:HGNC:50528] | -5,54 |
| LINC01366 | [ENSG00000235172](https://www.ensembl.org/id/ENSG00000235172) | long intergenic non-protein coding RNA 1366 [Source:HGNC Symbol;Acc:HGNC:27416] | 2,79 |
| LINC01405 | [ENSG00000185847](https://www.ensembl.org/id/ENSG00000185847) | long intergenic non-protein coding RNA 1405 [Source:HGNC Symbol;Acc:HGNC:50688] | -8,69 |
| LINC01411 | [ENSG00000249306](https://www.ensembl.org/id/ENSG00000249306) | long intergenic non-protein coding RNA 1411 [Source:HGNC Symbol;Acc:HGNC:50703] | -5,23 |
| LINC01465 | [ENSG00000221949](https://www.ensembl.org/id/ENSG00000221949) | long intergenic non-protein coding RNA 1465 [Source:HGNC Symbol;Acc:HGNC:26364] | -1,86 |
| LINC01484 | [ENSG00000253686](https://www.ensembl.org/id/ENSG00000253686) | long intergenic non-protein coding RNA 1484 [Source:HGNC Symbol;Acc:HGNC:51136] | -3,59 |
| LINC01489 | [ENSG00000255727](https://www.ensembl.org/id/ENSG00000255727) | long intergenic non-protein coding RNA 1489 [Source:HGNC Symbol;Acc:HGNC:51145] | -6,03 |
| LINC01497 | [ENSG00000237560](https://www.ensembl.org/id/ENSG00000237560) | long intergenic non-protein coding RNA 1497 [Source:HGNC Symbol;Acc:HGNC:51163] | -3,28 |
| LINC01563 | [ENSG00000236819](https://www.ensembl.org/id/ENSG00000236819) | long intergenic non-protein coding RNA 1563 [Source:HGNC Symbol;Acc:HGNC:51343] | -8,24 |
| LINC01597 | [ENSG00000205611](https://www.ensembl.org/id/ENSG00000205611) | long intergenic non-protein coding RNA 1597 [Source:HGNC Symbol;Acc:HGNC:51594] | 2,24 |
| LINC01634 | [ENSG00000235295](https://www.ensembl.org/id/ENSG00000235295) | long intergenic non-protein coding RNA 1634 [Source:HGNC Symbol;Acc:HGNC:52421] | -5,54 |
| LINC01637 | [ENSG00000237476](https://www.ensembl.org/id/ENSG00000237476) | long intergenic non-protein coding RNA 1637 [Source:HGNC Symbol;Acc:HGNC:52424] | -3,91 |
| LINC01801 | [ENSG00000267767](https://www.ensembl.org/id/ENSG00000267767) | long intergenic non-protein coding RNA 1801 [Source:HGNC Symbol;Acc:HGNC:52592] | -3,44 |
| LINC01854 | [ENSG00000204460](https://www.ensembl.org/id/ENSG00000204460) | long intergenic non-protein coding RNA 1854 [Source:HGNC Symbol;Acc:HGNC:52670] | -8,47 |
| LINC01936 | [ENSG00000235997](https://www.ensembl.org/id/ENSG00000235997) | long intergenic non-protein coding RNA 1936 [Source:HGNC Symbol;Acc:HGNC:52759] | 2,13 |
| LINC02006 | [ENSG00000238755](https://www.ensembl.org/id/ENSG00000238755) | long intergenic non-protein coding RNA 2006 [Source:HGNC Symbol;Acc:HGNC:52842] | -6,20 |
| LINC02120 | [ENSG00000248279](https://www.ensembl.org/id/ENSG00000248279) | long intergenic non-protein coding RNA 2120 [Source:HGNC Symbol;Acc:HGNC:27971] | -5,52 |
| LINC02268 | [ENSG00000248174](https://www.ensembl.org/id/ENSG00000248174) | long intergenic non-protein coding RNA 2268 [Source:HGNC Symbol;Acc:HGNC:53183] | -5,91 |
| LINC02308 | [ENSG00000258675](https://www.ensembl.org/id/ENSG00000258675) | long intergenic non-protein coding RNA 2308 [Source:HGNC Symbol;Acc:HGNC:53227] | -3,96 |
| LINC02328 | [ENSG00000258733](https://www.ensembl.org/id/ENSG00000258733) | long intergenic non-protein coding RNA 2328 [Source:HGNC Symbol;Acc:HGNC:53248] | -2,16 |
| LINC02405 | [ENSG00000249345](https://www.ensembl.org/id/ENSG00000249345) | long intergenic non-protein coding RNA 2405 [Source:HGNC Symbol;Acc:HGNC:53333] | -3,46 |
| LINC02418 | [ENSG00000214039](https://www.ensembl.org/id/ENSG00000214039) | long intergenic non-protein coding RNA 2418 [Source:HGNC Symbol;Acc:HGNC:53348] | -5,34 |
| LINC02541 | [ENSG00000230943](https://www.ensembl.org/id/ENSG00000230943) | long intergenic non-protein coding RNA 2541 [Source:HGNC Symbol;Acc:HGNC:53574] | -5,66 |
| LINC02542 | [ENSG00000226453](https://www.ensembl.org/id/ENSG00000226453) | long intergenic non-protein coding RNA 2542 [Source:HGNC Symbol;Acc:HGNC:53576] | -4,31 |
| LINC02682 | [ENSG00000254661](https://www.ensembl.org/id/ENSG00000254661) | long intergenic non-protein coding RNA 2682 [Source:HGNC Symbol;Acc:HGNC:54177] | 3,12 |
| LINC02721 | [ENSG00000255133](https://www.ensembl.org/id/ENSG00000255133) | long intergenic non-protein coding RNA 2721 [Source:HGNC Symbol;Acc:HGNC:54238] | -5,50 |
| LINC02747 | [ENSG00000255774](https://www.ensembl.org/id/ENSG00000255774) | long intergenic non-protein coding RNA 2747 [Source:HGNC Symbol;Acc:HGNC:54266] | 4,99 |
| LINC02772 | [ENSG00000271736](https://www.ensembl.org/id/ENSG00000271736) | long intergenic non-protein coding RNA 2772 [Source:HGNC Symbol;Acc:HGNC:54292] | 5,35 |
| LINC02798 | [ENSG00000227082](https://www.ensembl.org/id/ENSG00000227082) | long intergenic non-protein coding RNA 2798 [Source:HGNC Symbol;Acc:HGNC:54323] | -2,11 |
| LINC02899 | [ENSG00000248874](https://www.ensembl.org/id/ENSG00000248874) | long intergenic non-protein coding RNA 2899 [Source:HGNC Symbol;Acc:HGNC:26630] | 3,09 |
| LINGO1 | [ENSG00000169783](https://www.ensembl.org/id/ENSG00000169783) | leucine rich repeat and Ig domain containing 1 [Source:HGNC Symbol;Acc:HGNC:21205] | 2,48 |
| LINGO4 | [ENSG00000213171](https://www.ensembl.org/id/ENSG00000213171) | leucine rich repeat and Ig domain containing 4 [Source:HGNC Symbol;Acc:HGNC:31814] | -8,51 |
| LINP1 | [ENSG00000223784](https://www.ensembl.org/id/ENSG00000223784) | lncRNA in non-homologous end joining pathway 1 [Source:HGNC Symbol;Acc:HGNC:53170] | -8,79 |
| LIPC | [ENSG00000166035](https://www.ensembl.org/id/ENSG00000166035) | lipase C, hepatic type [Source:HGNC Symbol;Acc:HGNC:6619] | -2,47 |
| LIPE | [ENSG00000079435](https://www.ensembl.org/id/ENSG00000079435) | lipase E, hormone sensitive type [Source:HGNC Symbol;Acc:HGNC:6621] | -3,08 |
| LIPN | [ENSG00000204020](https://www.ensembl.org/id/ENSG00000204020) | lipase family member N [Source:HGNC Symbol;Acc:HGNC:23452] | 5,10 |
| LLPH-DT | [ENSG00000239335](https://www.ensembl.org/id/ENSG00000239335) | LLPH divergent transcript [Source:HGNC Symbol;Acc:HGNC:50493] | -5,16 |
| LMNTD2-AS1 | [ENSG00000254815](https://www.ensembl.org/id/ENSG00000254815) | LMNTD2 antisense RNA 1 [Source:HGNC Symbol;Acc:HGNC:41204] | -2,47 |
| LMO1 | [ENSG00000166407](https://www.ensembl.org/id/ENSG00000166407) | LIM domain only 1 [Source:HGNC Symbol;Acc:HGNC:6641] | -3,96 |
| LMOD2 | [ENSG00000170807](https://www.ensembl.org/id/ENSG00000170807) | leiomodin 2 [Source:HGNC Symbol;Acc:HGNC:6648] | -8,08 |
| LMOD3 | [ENSG00000163380](https://www.ensembl.org/id/ENSG00000163380) | leiomodin 3 [Source:HGNC Symbol;Acc:HGNC:6649] | -4,82 |
| LMX1B | [ENSG00000136944](https://www.ensembl.org/id/ENSG00000136944) | LIM homeobox transcription factor 1 beta [Source:HGNC Symbol;Acc:HGNC:6654] | -2,26 |
| LPL | [ENSG00000175445](https://www.ensembl.org/id/ENSG00000175445) | lipoprotein lipase [Source:HGNC Symbol;Acc:HGNC:6677] | -2,11 |
| LRAT | [ENSG00000121207](https://www.ensembl.org/id/ENSG00000121207) | lecithin retinol acyltransferase [Source:HGNC Symbol;Acc:HGNC:6685] | -3,33 |
| LRP1B | [ENSG00000168702](https://www.ensembl.org/id/ENSG00000168702) | LDL receptor related protein 1B [Source:HGNC Symbol;Acc:HGNC:6693] | -3,37 |
| LRP2 | [ENSG00000081479](https://www.ensembl.org/id/ENSG00000081479) | LDL receptor related protein 2 [Source:HGNC Symbol;Acc:HGNC:6694] | -9,53 |
| LRRC10B | [ENSG00000204950](https://www.ensembl.org/id/ENSG00000204950) | leucine rich repeat containing 10B [Source:HGNC Symbol;Acc:HGNC:37215] | 2,50 |
| LRRC14B | [ENSG00000185028](https://www.ensembl.org/id/ENSG00000185028) | leucine rich repeat containing 14B [Source:HGNC Symbol;Acc:HGNC:37268] | -9,92 |
| LRRC2 | [ENSG00000163827](https://www.ensembl.org/id/ENSG00000163827) | leucine rich repeat containing 2 [Source:HGNC Symbol;Acc:HGNC:14676] | -4,53 |
| LRRC20 | [ENSG00000172731](https://www.ensembl.org/id/ENSG00000172731) | leucine rich repeat containing 20 [Source:HGNC Symbol;Acc:HGNC:23421] | -2,58 |
| LRRC30 | [ENSG00000206422](https://www.ensembl.org/id/ENSG00000206422) | leucine rich repeat containing 30 [Source:HGNC Symbol;Acc:HGNC:30219] | -6,05 |
| LRRC37A11P | [ENSG00000290925](https://www.ensembl.org/id/ENSG00000290925) | leucine rich repeat containing 37 member A11, pseudogene [Source:HGNC Symbol;Acc:HGNC:43815] | -3,55 |
| LRRC38 | [ENSG00000162494](https://www.ensembl.org/id/ENSG00000162494) | leucine rich repeat containing 38 [Source:HGNC Symbol;Acc:HGNC:27005] | -5,35 |
| LRRC39 | [ENSG00000122477](https://www.ensembl.org/id/ENSG00000122477) | leucine rich repeat containing 39 [Source:HGNC Symbol;Acc:HGNC:28228] | -4,98 |
| LRRC3B-AS1 | [ENSG00000225386](https://www.ensembl.org/id/ENSG00000225386) | LRRC3B antisense RNA 1 [Source:HGNC Symbol;Acc:HGNC:55847] | -5,87 |
| LRRC4B | [ENSG00000131409](https://www.ensembl.org/id/ENSG00000131409) | leucine rich repeat containing 4B [Source:HGNC Symbol;Acc:HGNC:25042] | 1,58 |
| LRRN1 | [ENSG00000175928](https://www.ensembl.org/id/ENSG00000175928) | leucine rich repeat neuronal 1 [Source:HGNC Symbol;Acc:HGNC:20980] | -2,37 |
| LRRN4 | [ENSG00000125872](https://www.ensembl.org/id/ENSG00000125872) | leucine rich repeat neuronal 4 [Source:HGNC Symbol;Acc:HGNC:16208] | -5,49 |
| LRRTM4 | [ENSG00000176204](https://www.ensembl.org/id/ENSG00000176204) | leucine rich repeat transmembrane neuronal 4 [Source:HGNC Symbol;Acc:HGNC:19411] | -3,61 |
| LSAMP-AS1 | [ENSG00000240922](https://www.ensembl.org/id/ENSG00000240922) | LSAMP antisense RNA 1 [Source:HGNC Symbol;Acc:HGNC:40350] | -5,66 |
| LSMEM1 | [ENSG00000181016](https://www.ensembl.org/id/ENSG00000181016) | leucine rich single-pass membrane protein 1 [Source:HGNC Symbol;Acc:HGNC:22036] | -2,39 |
| LSP1P5_1 | [ENSG00000288905](https://www.ensembl.org/id/ENSG00000288905) | LSP1 pseudogene 5 [Source:NCBI gene (formerly Entrezgene);Acc:645166] | -1,64 |
| LST1 | [ENSG00000204482](https://www.ensembl.org/id/ENSG00000204482) | leukocyte specific transcript 1 [Source:HGNC Symbol;Acc:HGNC:14189] | 2,74 |
| LTB | [ENSG00000227507](https://www.ensembl.org/id/ENSG00000227507) | lymphotoxin beta [Source:HGNC Symbol;Acc:HGNC:6711] | 2,51 |
| LVRN | [ENSG00000172901](https://www.ensembl.org/id/ENSG00000172901) | laeverin [Source:HGNC Symbol;Acc:HGNC:26904] | -1,74 |
| LYPD6 | [ENSG00000187123](https://www.ensembl.org/id/ENSG00000187123) | LY6/PLAUR domain containing 6 [Source:HGNC Symbol;Acc:HGNC:28751] | -2,48 |
| LYZ | [ENSG00000090382](https://www.ensembl.org/id/ENSG00000090382) | lysozyme [Source:HGNC Symbol;Acc:HGNC:6740] | 2,49 |
| MAF | [ENSG00000178573](https://www.ensembl.org/id/ENSG00000178573) | MAF bZIP transcription factor [Source:HGNC Symbol;Acc:HGNC:6776] | -1,38 |
| MAL2 | [ENSG00000147676](https://www.ensembl.org/id/ENSG00000147676) | mal, T cell differentiation protein 2 [Source:HGNC Symbol;Acc:HGNC:13634] | -2,66 |
| MAP3K5-AS2 | [ENSG00000286646](https://www.ensembl.org/id/ENSG00000286646) | MAP3K5 antisense RNA 2 [Source:HGNC Symbol;Acc:HGNC:56125] | -3,88 |
| MAP4K1-AS1 | [ENSG00000267291](https://www.ensembl.org/id/ENSG00000267291) | MAP4K1 antisense RNA 1 [Source:HGNC Symbol;Acc:HGNC:55302] | -5,93 |
| MAPK10 | [ENSG00000109339](https://www.ensembl.org/id/ENSG00000109339) | mitogen-activated protein kinase 10 [Source:HGNC Symbol;Acc:HGNC:6872] | -1,18 |
| MAPK12 | [ENSG00000188130](https://www.ensembl.org/id/ENSG00000188130) | mitogen-activated protein kinase 12 [Source:HGNC Symbol;Acc:HGNC:6874] | -2,34 |
| MAPK4 | [ENSG00000141639](https://www.ensembl.org/id/ENSG00000141639) | mitogen-activated protein kinase 4 [Source:HGNC Symbol;Acc:HGNC:6878] | 1,56 |
| MAPT | [ENSG00000186868](https://www.ensembl.org/id/ENSG00000186868) | microtubule associated protein tau [Source:HGNC Symbol;Acc:HGNC:6893] | -1,88 |
| MARCHF10 | [ENSG00000173838](https://www.ensembl.org/id/ENSG00000173838) | membrane associated ring-CH-type finger 10 [Source:HGNC Symbol;Acc:HGNC:26655] | -4,92 |
| MAT1A | [ENSG00000151224](https://www.ensembl.org/id/ENSG00000151224) | methionine adenosyltransferase 1A [Source:HGNC Symbol;Acc:HGNC:6903] | 5,21 |
| MATN4 | [ENSG00000124159](https://www.ensembl.org/id/ENSG00000124159) | matrilin 4 [Source:HGNC Symbol;Acc:HGNC:6910] | -2,40 |
| MB | [ENSG00000198125](https://www.ensembl.org/id/ENSG00000198125) | myoglobin [Source:HGNC Symbol;Acc:HGNC:6915] | -9,44 |
| MCEMP1 | [ENSG00000183019](https://www.ensembl.org/id/ENSG00000183019) | mast cell expressed membrane protein 1 [Source:HGNC Symbol;Acc:HGNC:27291] | 6,16 |
| MCOLN3 | [ENSG00000055732](https://www.ensembl.org/id/ENSG00000055732) | mucolipin TRP cation channel 3 [Source:HGNC Symbol;Acc:HGNC:13358] | -2,77 |
| MDFI | [ENSG00000112559](https://www.ensembl.org/id/ENSG00000112559) | MyoD family inhibitor [Source:HGNC Symbol;Acc:HGNC:6967] | -2,88 |
| MEFV | [ENSG00000103313](https://www.ensembl.org/id/ENSG00000103313) | MEFV innate immuity regulator, pyrin [Source:HGNC Symbol;Acc:HGNC:6998] | 3,85 |
| MEG3 | [ENSG00000214548](https://www.ensembl.org/id/ENSG00000214548) | maternally expressed 3 [Source:HGNC Symbol;Acc:HGNC:14575] | -2,02 |
| MESP1 | [ENSG00000166823](https://www.ensembl.org/id/ENSG00000166823) | mesoderm posterior bHLH transcription factor 1 [Source:HGNC Symbol;Acc:HGNC:29658] | -2,48 |
| Metazoa_SRP_123 | [ENSG00000274860](https://www.ensembl.org/id/ENSG00000274860) | Metazoan signal recognition particle RNA [Source:RFAM;Acc:RF00017] | -4,98 |
| METTL7B | [ENSG00000170439](https://www.ensembl.org/id/ENSG00000170439) | methyltransferase like 7B [Source:HGNC Symbol;Acc:HGNC:28276] | -2,85 |
| MGAM | [ENSG00000257335](https://www.ensembl.org/id/ENSG00000257335) | maltase-glucoamylase [Source:HGNC Symbol;Acc:HGNC:7043] | 2,85 |
| MGARP | [ENSG00000137463](https://www.ensembl.org/id/ENSG00000137463) | mitochondria localized glutamic acid rich protein [Source:HGNC Symbol;Acc:HGNC:29969] | -2,14 |
| MGST1 | [ENSG00000008394](https://www.ensembl.org/id/ENSG00000008394) | microsomal glutathione S-transferase 1 [Source:HGNC Symbol;Acc:HGNC:7061] | -2,06 |
| MID1 | [ENSG00000101871](https://www.ensembl.org/id/ENSG00000101871) | midline 1 [Source:HGNC Symbol;Acc:HGNC:7095] | -1,54 |
| MIR1-1HG | [ENSG00000174407](https://www.ensembl.org/id/ENSG00000174407) | MIR1-1 host gene [Source:HGNC Symbol;Acc:HGNC:16159] | -7,87 |
| MIR133A1HG | [ENSG00000265142](https://www.ensembl.org/id/ENSG00000265142) | MIR133A1 host gene [Source:HGNC Symbol;Acc:HGNC:49594] | -3,63 |
| MIR223HG | [ENSG00000274536](https://www.ensembl.org/id/ENSG00000274536) | MIR223 host gene [Source:HGNC Symbol;Acc:HGNC:54520] | 5,85 |
| MIR3945HG | [ENSG00000251230](https://www.ensembl.org/id/ENSG00000251230) | MIR3945 host gene [Source:HGNC Symbol;Acc:HGNC:52002] | 5,95 |
| MIR4300HG | [ENSG00000245832](https://www.ensembl.org/id/ENSG00000245832) | MIR4300 host gene [Source:HGNC Symbol;Acc:HGNC:52003] | -5,42 |
| MLIP | [ENSG00000146147](https://www.ensembl.org/id/ENSG00000146147) | muscular LMNA interacting protein [Source:HGNC Symbol;Acc:HGNC:21355] | -3,97 |
| MLXIPL | [ENSG00000009950](https://www.ensembl.org/id/ENSG00000009950) | MLX interacting protein like [Source:HGNC Symbol;Acc:HGNC:12744] | -3,33 |
| MMP13 | [ENSG00000137745](https://www.ensembl.org/id/ENSG00000137745) | matrix metallopeptidase 13 [Source:HGNC Symbol;Acc:HGNC:7159] | -4,98 |
| MMP25 | [ENSG00000008516](https://www.ensembl.org/id/ENSG00000008516) | matrix metallopeptidase 25 [Source:HGNC Symbol;Acc:HGNC:14246] | 3,74 |
| MMP9 | [ENSG00000100985](https://www.ensembl.org/id/ENSG00000100985) | matrix metallopeptidase 9 [Source:HGNC Symbol;Acc:HGNC:7176] | 2,88 |
| MND1 | [ENSG00000121211](https://www.ensembl.org/id/ENSG00000121211) | meiotic nuclear divisions 1 [Source:HGNC Symbol;Acc:HGNC:24839] | 2,18 |
| MNDA | [ENSG00000163563](https://www.ensembl.org/id/ENSG00000163563) | myeloid cell nuclear differentiation antigen [Source:HGNC Symbol;Acc:HGNC:7183] | 2,90 |
| MPHOSPH10P1 | [ENSG00000291103](https://www.ensembl.org/id/ENSG00000291103) | MPHOSPH10 pseudogene 1 [Source:NCBI gene (formerly Entrezgene);Acc:643802] | 3,05 |
| MPO | [ENSG00000005381](https://www.ensembl.org/id/ENSG00000005381) | myeloperoxidase [Source:HGNC Symbol;Acc:HGNC:7218] | -2,76 |
| MPZ | [ENSG00000158887](https://www.ensembl.org/id/ENSG00000158887) | myelin protein zero [Source:HGNC Symbol;Acc:HGNC:7225] | -2,24 |
| MRC2 | [ENSG00000011028](https://www.ensembl.org/id/ENSG00000011028) | mannose receptor C type 2 [Source:HGNC Symbol;Acc:HGNC:16875] | -1,46 |
| MRLN | [ENSG00000227877](https://www.ensembl.org/id/ENSG00000227877) | myoregulin [Source:HGNC Symbol;Acc:HGNC:48649] | -4,80 |
| MS4A2 | [ENSG00000149534](https://www.ensembl.org/id/ENSG00000149534) | membrane spanning 4-domains A2 [Source:HGNC Symbol;Acc:HGNC:7316] | -2,73 |
| MS4A8 | [ENSG00000166959](https://www.ensembl.org/id/ENSG00000166959) | membrane spanning 4-domains A8 [Source:HGNC Symbol;Acc:HGNC:13380] | -5,03 |
| MSLN | [ENSG00000102854](https://www.ensembl.org/id/ENSG00000102854) | mesothelin [Source:HGNC Symbol;Acc:HGNC:7371] | -8,03 |
| MSS51 | [ENSG00000166343](https://www.ensembl.org/id/ENSG00000166343) | MSS51 mitochondrial translational activator [Source:HGNC Symbol;Acc:HGNC:21000] | -3,97 |
| MST1R | [ENSG00000164078](https://www.ensembl.org/id/ENSG00000164078) | macrophage stimulating 1 receptor [Source:HGNC Symbol;Acc:HGNC:7381] | -1,72 |
| MSTN | [ENSG00000138379](https://www.ensembl.org/id/ENSG00000138379) | myostatin [Source:HGNC Symbol;Acc:HGNC:4223] | -2,86 |
| MT-ATP8 | [ENSG00000228253](https://www.ensembl.org/id/ENSG00000228253) | mitochondrially encoded ATP synthase membrane subunit 8 [Source:HGNC Symbol;Acc:HGNC:7415] | -2,33 |
| MT-CYB | [ENSG00000198727](https://www.ensembl.org/id/ENSG00000198727) | mitochondrially encoded cytochrome b [Source:HGNC Symbol;Acc:HGNC:7427] | -1,93 |
| MT-ND1 | [ENSG00000198888](https://www.ensembl.org/id/ENSG00000198888) | mitochondrially encoded NADH:ubiquinone oxidoreductase core subunit 1 [Source:HGNC Symbol;Acc:HGNC:7455] | -2,01 |
| MT-ND4L | [ENSG00000212907](https://www.ensembl.org/id/ENSG00000212907) | mitochondrially encoded NADH:ubiquinone oxidoreductase core subunit 4L [Source:HGNC Symbol;Acc:HGNC:7460] | -2,20 |
| MT-ND5 | [ENSG00000198786](https://www.ensembl.org/id/ENSG00000198786) | mitochondrially encoded NADH:ubiquinone oxidoreductase core subunit 5 [Source:HGNC Symbol;Acc:HGNC:7461] | -2,13 |
| MTUS1-DT | [ENSG00000253944](https://www.ensembl.org/id/ENSG00000253944) | MTUS1 divergent transcript [Source:HGNC Symbol;Acc:HGNC:55525] | -3,58 |
| MTUS2 | [ENSG00000132938](https://www.ensembl.org/id/ENSG00000132938) | microtubule associated scaffold protein 2 [Source:HGNC Symbol;Acc:HGNC:20595] | -3,01 |
| MUC13 | [ENSG00000173702](https://www.ensembl.org/id/ENSG00000173702) | mucin 13, cell surface associated [Source:HGNC Symbol;Acc:HGNC:7511] | -5,77 |
| MUC16 | [ENSG00000181143](https://www.ensembl.org/id/ENSG00000181143) | mucin 16, cell surface associated [Source:HGNC Symbol;Acc:HGNC:15582] | -10,40 |
| MYADML2 | [ENSG00000185105](https://www.ensembl.org/id/ENSG00000185105) | myeloid associated differentiation marker like 2 [Source:HGNC Symbol;Acc:HGNC:34548] | -11,96 |
| MYBPC1 | [ENSG00000196091](https://www.ensembl.org/id/ENSG00000196091) | myosin binding protein C1 [Source:HGNC Symbol;Acc:HGNC:7549] | -9,21 |
| MYBPC2 | [ENSG00000086967](https://www.ensembl.org/id/ENSG00000086967) | myosin binding protein C2 [Source:HGNC Symbol;Acc:HGNC:7550] | -8,40 |
| MYBPH | [ENSG00000133055](https://www.ensembl.org/id/ENSG00000133055) | myosin binding protein H [Source:HGNC Symbol;Acc:HGNC:7552] | -3,47 |
| MYCL-AS1 | [ENSG00000236546](https://www.ensembl.org/id/ENSG00000236546) | MYCL antisense RNA 1 [Source:HGNC Symbol;Acc:HGNC:40386] | -3,89 |
| MYF5 | [ENSG00000111049](https://www.ensembl.org/id/ENSG00000111049) | myogenic factor 5 [Source:HGNC Symbol;Acc:HGNC:7565] | -6,75 |
| MYF6 | [ENSG00000111046](https://www.ensembl.org/id/ENSG00000111046) | myogenic factor 6 [Source:HGNC Symbol;Acc:HGNC:7566] | -5,96 |
| MYH1 | [ENSG00000109061](https://www.ensembl.org/id/ENSG00000109061) | myosin heavy chain 1 [Source:HGNC Symbol;Acc:HGNC:7567] | -8,25 |
| MYH2 | [ENSG00000125414](https://www.ensembl.org/id/ENSG00000125414) | myosin heavy chain 2 [Source:HGNC Symbol;Acc:HGNC:7572] | -8,25 |
| MYH3 | [ENSG00000109063](https://www.ensembl.org/id/ENSG00000109063) | myosin heavy chain 3 [Source:HGNC Symbol;Acc:HGNC:7573] | -1,92 |
| MYH4 | [ENSG00000264424](https://www.ensembl.org/id/ENSG00000264424) | myosin heavy chain 4 [Source:HGNC Symbol;Acc:HGNC:7574] | -5,99 |
| MYH6 | [ENSG00000197616](https://www.ensembl.org/id/ENSG00000197616) | myosin heavy chain 6 [Source:HGNC Symbol;Acc:HGNC:7576] | -9,14 |
| MYH7 | [ENSG00000092054](https://www.ensembl.org/id/ENSG00000092054) | myosin heavy chain 7 [Source:HGNC Symbol;Acc:HGNC:7577] | -12,46 |
| MYH7B | [ENSG00000078814](https://www.ensembl.org/id/ENSG00000078814) | myosin heavy chain 7B [Source:HGNC Symbol;Acc:HGNC:15906] | -4,44 |
| MYHAS | [ENSG00000272975](https://www.ensembl.org/id/ENSG00000272975) | myosin heavy chain gene cluster antisense RNA [Source:HGNC Symbol;Acc:HGNC:50609] | -9,14 |
| MYL1 | [ENSG00000168530](https://www.ensembl.org/id/ENSG00000168530) | myosin light chain 1 [Source:HGNC Symbol;Acc:HGNC:7582] | -8,43 |
| MYL10 | [ENSG00000106436](https://www.ensembl.org/id/ENSG00000106436) | myosin light chain 10 [Source:HGNC Symbol;Acc:HGNC:29825] | -11,52 |
| MYL11 | [ENSG00000180209](https://www.ensembl.org/id/ENSG00000180209) | myosin light chain 11 [Source:HGNC Symbol;Acc:HGNC:29824] | -6,96 |
| MYL2 | [ENSG00000111245](https://www.ensembl.org/id/ENSG00000111245) | myosin light chain 2 [Source:HGNC Symbol;Acc:HGNC:7583] | -10,67 |
| MYL3 | [ENSG00000160808](https://www.ensembl.org/id/ENSG00000160808) | myosin light chain 3 [Source:HGNC Symbol;Acc:HGNC:7584] | -6,18 |
| MYL6B | [ENSG00000196465](https://www.ensembl.org/id/ENSG00000196465) | myosin light chain 6B [Source:HGNC Symbol;Acc:HGNC:29823] | -2,90 |
| MYLK2 | [ENSG00000101306](https://www.ensembl.org/id/ENSG00000101306) | myosin light chain kinase 2 [Source:HGNC Symbol;Acc:HGNC:16243] | -6,73 |
| MYLK3 | [ENSG00000140795](https://www.ensembl.org/id/ENSG00000140795) | myosin light chain kinase 3 [Source:HGNC Symbol;Acc:HGNC:29826] | -4,04 |
| MYMK | [ENSG00000187616](https://www.ensembl.org/id/ENSG00000187616) | myomaker, myoblast fusion factor [Source:HGNC Symbol;Acc:HGNC:33778] | -4,74 |
| MYMX | [ENSG00000262179](https://www.ensembl.org/id/ENSG00000262179) | myomixer, myoblast fusion factor [Source:HGNC Symbol;Acc:HGNC:52391] | -3,26 |
| MYO18B | [ENSG00000133454](https://www.ensembl.org/id/ENSG00000133454) | myosin XVIIIB [Source:HGNC Symbol;Acc:HGNC:18150] | -5,18 |
| MYO1F | [ENSG00000142347](https://www.ensembl.org/id/ENSG00000142347) | myosin IF [Source:HGNC Symbol;Acc:HGNC:7600] | 2,26 |
| MYO5B | [ENSG00000167306](https://www.ensembl.org/id/ENSG00000167306) | myosin VB [Source:HGNC Symbol;Acc:HGNC:7603] | -2,50 |
| MYO7A | [ENSG00000137474](https://www.ensembl.org/id/ENSG00000137474) | myosin VIIA [Source:HGNC Symbol;Acc:HGNC:7606] | -1,51 |
| MYOD1 | [ENSG00000129152](https://www.ensembl.org/id/ENSG00000129152) | myogenic differentiation 1 [Source:HGNC Symbol;Acc:HGNC:7611] | -6,18 |
| MYOG | [ENSG00000122180](https://www.ensembl.org/id/ENSG00000122180) | myogenin [Source:HGNC Symbol;Acc:HGNC:7612] | -3,86 |
| MYOM2 | [ENSG00000036448](https://www.ensembl.org/id/ENSG00000036448) | myomesin 2 [Source:HGNC Symbol;Acc:HGNC:7614] | -4,39 |
| MYOM3 | [ENSG00000142661](https://www.ensembl.org/id/ENSG00000142661) | myomesin 3 [Source:HGNC Symbol;Acc:HGNC:26679] | -6,96 |
| MYOT | [ENSG00000120729](https://www.ensembl.org/id/ENSG00000120729) | myotilin [Source:HGNC Symbol;Acc:HGNC:12399] | -7,88 |
| MYOZ1 | [ENSG00000177791](https://www.ensembl.org/id/ENSG00000177791) | myozenin 1 [Source:HGNC Symbol;Acc:HGNC:13752] | -3,26 |
| MYOZ2 | [ENSG00000172399](https://www.ensembl.org/id/ENSG00000172399) | myozenin 2 [Source:HGNC Symbol;Acc:HGNC:1330] | -3,72 |
| MYOZ3 | [ENSG00000164591](https://www.ensembl.org/id/ENSG00000164591) | myozenin 3 [Source:HGNC Symbol;Acc:HGNC:18565] | -3,79 |
| MYPN | [ENSG00000138347](https://www.ensembl.org/id/ENSG00000138347) | myopalladin [Source:HGNC Symbol;Acc:HGNC:23246] | -8,35 |
| MYRF | [ENSG00000124920](https://www.ensembl.org/id/ENSG00000124920) | myelin regulatory factor [Source:HGNC Symbol;Acc:HGNC:1181] | -2,95 |
| MYRIP | [ENSG00000170011](https://www.ensembl.org/id/ENSG00000170011) | myosin VIIA and Rab interacting protein [Source:HGNC Symbol;Acc:HGNC:19156] | 1,05 |
| NANOS1 | [ENSG00000188613](https://www.ensembl.org/id/ENSG00000188613) | nanos C2HC-type zinc finger 1 [Source:HGNC Symbol;Acc:HGNC:23044] | -1,70 |
| NAT8L | [ENSG00000185818](https://www.ensembl.org/id/ENSG00000185818) | N-acetyltransferase 8 like [Source:HGNC Symbol;Acc:HGNC:26742] | -2,69 |
| NAV1 | [ENSG00000134369](https://www.ensembl.org/id/ENSG00000134369) | neuron navigator 1 [Source:HGNC Symbol;Acc:HGNC:15989] | -1,07 |
| NAV2-AS1 | [ENSG00000254894](https://www.ensembl.org/id/ENSG00000254894) | NAV2 antisense RNA 1 [Source:HGNC Symbol;Acc:HGNC:40744] | -6,14 |
| NAV2-AS2 | [ENSG00000254453](https://www.ensembl.org/id/ENSG00000254453) | NAV2 antisense RNA 2 [Source:HGNC Symbol;Acc:HGNC:40743] | 3,30 |
| NCAM1 | [ENSG00000149294](https://www.ensembl.org/id/ENSG00000149294) | neural cell adhesion molecule 1 [Source:HGNC Symbol;Acc:HGNC:7656] | -2,04 |
| NCF1 | [ENSG00000158517](https://www.ensembl.org/id/ENSG00000158517) | neutrophil cytosolic factor 1 [Source:HGNC Symbol;Acc:HGNC:7660] | 2,50 |
| NCMAP | [ENSG00000184454](https://www.ensembl.org/id/ENSG00000184454) | non-compact myelin associated protein [Source:HGNC Symbol;Acc:HGNC:29332] | -4,40 |
| NCR1 | [ENSG00000189430](https://www.ensembl.org/id/ENSG00000189430) | natural cytotoxicity triggering receptor 1 [Source:HGNC Symbol;Acc:HGNC:6731] | 5,10 |
| NCR3 | [ENSG00000204475](https://www.ensembl.org/id/ENSG00000204475) | natural cytotoxicity triggering receptor 3 [Source:HGNC Symbol;Acc:HGNC:19077] | 5,97 |
| NEB | [ENSG00000183091](https://www.ensembl.org/id/ENSG00000183091) | nebulin [Source:HGNC Symbol;Acc:HGNC:7720] | -8,17 |
| NEDD4L | [ENSG00000049759](https://www.ensembl.org/id/ENSG00000049759) | NEDD4 like E3 ubiquitin protein ligase [Source:HGNC Symbol;Acc:HGNC:7728] | -2,32 |
| NEFH | [ENSG00000100285](https://www.ensembl.org/id/ENSG00000100285) | neurofilament heavy chain [Source:HGNC Symbol;Acc:HGNC:7737] | -2,15 |
| NEFM | [ENSG00000104722](https://www.ensembl.org/id/ENSG00000104722) | neurofilament medium chain [Source:HGNC Symbol;Acc:HGNC:7734] | -3,62 |
| NEURL1 | [ENSG00000107954](https://www.ensembl.org/id/ENSG00000107954) | neuralized E3 ubiquitin protein ligase 1 [Source:HGNC Symbol;Acc:HGNC:7761] | -4,97 |
| NEURL3 | [ENSG00000163121](https://www.ensembl.org/id/ENSG00000163121) | neuralized E3 ubiquitin protein ligase 3 [Source:HGNC Symbol;Acc:HGNC:25162] | -6,07 |
| NFAM1 | [ENSG00000235568](https://www.ensembl.org/id/ENSG00000235568) | NFAT activating protein with ITAM motif 1 [Source:HGNC Symbol;Acc:HGNC:29872] | 3,91 |
| NFASC | [ENSG00000163531](https://www.ensembl.org/id/ENSG00000163531) | neurofascin [Source:HGNC Symbol;Acc:HGNC:29866] | 1,52 |
| NFE2 | [ENSG00000123405](https://www.ensembl.org/id/ENSG00000123405) | nuclear factor, erythroid 2 [Source:HGNC Symbol;Acc:HGNC:7780] | 3,01 |
| NGFR | [ENSG00000064300](https://www.ensembl.org/id/ENSG00000064300) | nerve growth factor receptor [Source:HGNC Symbol;Acc:HGNC:7809] | -1,03 |
| NIPAL4-DT | [ENSG00000251405](https://www.ensembl.org/id/ENSG00000251405) | NIPAL4 divergent transcript [Source:HGNC Symbol;Acc:HGNC:55542] | -4,46 |
| NKAIN1 | [ENSG00000084628](https://www.ensembl.org/id/ENSG00000084628) | sodium/potassium transporting ATPase interacting 1 [Source:HGNC Symbol;Acc:HGNC:25743] | -5,75 |
| NKAIN4 | [ENSG00000101198](https://www.ensembl.org/id/ENSG00000101198) | sodium/potassium transporting ATPase interacting 4 [Source:HGNC Symbol;Acc:HGNC:16191] | -6,30 |
| NKG7 | [ENSG00000105374](https://www.ensembl.org/id/ENSG00000105374) | natural killer cell granule protein 7 [Source:HGNC Symbol;Acc:HGNC:7830] | 2,58 |
| NLRP12 | [ENSG00000142405](https://www.ensembl.org/id/ENSG00000142405) | NLR family pyrin domain containing 12 [Source:HGNC Symbol;Acc:HGNC:22938] | 3,08 |
| NLRP2B | [ENSG00000215174](https://www.ensembl.org/id/ENSG00000215174) | NLR family pyrin domain containing 2B [Source:HGNC Symbol;Acc:HGNC:29887] | 5,34 |
| NOMO3 | [ENSG00000103226](https://www.ensembl.org/id/ENSG00000103226) | NODAL modulator 3 [Source:HGNC Symbol;Acc:HGNC:25242] | 1,19 |
| NOS1 | [ENSG00000089250](https://www.ensembl.org/id/ENSG00000089250) | nitric oxide synthase 1 [Source:HGNC Symbol;Acc:HGNC:7872] | -4,36 |
| NOTUM | [ENSG00000185269](https://www.ensembl.org/id/ENSG00000185269) | notum, palmitoleoyl-protein carboxylesterase [Source:HGNC Symbol;Acc:HGNC:27106] | -4,08 |
| NOX4 | [ENSG00000086991](https://www.ensembl.org/id/ENSG00000086991) | NADPH oxidase 4 [Source:HGNC Symbol;Acc:HGNC:7891] | -1,36 |
| NOX5_2 | [ENSG00000255346](https://www.ensembl.org/id/ENSG00000255346) | NADPH oxidase 5 [Source:HGNC Symbol;Acc:HGNC:14874] | 3,47 |
| NPAS2 | [ENSG00000170485](https://www.ensembl.org/id/ENSG00000170485) | neuronal PAS domain protein 2 [Source:HGNC Symbol;Acc:HGNC:7895] | -1,43 |
| NPFFR1 | [ENSG00000148734](https://www.ensembl.org/id/ENSG00000148734) | neuropeptide FF receptor 1 [Source:HGNC Symbol;Acc:HGNC:17425] | -7,15 |
| NPFFR2 | [ENSG00000056291](https://www.ensembl.org/id/ENSG00000056291) | neuropeptide FF receptor 2 [Source:HGNC Symbol;Acc:HGNC:4525] | -3,61 |
| NPIPA8 | [ENSG00000214940](https://www.ensembl.org/id/ENSG00000214940) | nuclear pore complex interacting protein family member A8 [Source:HGNC Symbol;Acc:HGNC:41983] | 10,69 |
| NPTX1 | [ENSG00000171246](https://www.ensembl.org/id/ENSG00000171246) | neuronal pentraxin 1 [Source:HGNC Symbol;Acc:HGNC:7952] | 1,73 |
| NPW | [ENSG00000183971](https://www.ensembl.org/id/ENSG00000183971) | neuropeptide W [Source:HGNC Symbol;Acc:HGNC:30509] | -2,89 |
| NQO1-DT | [ENSG00000262136](https://www.ensembl.org/id/ENSG00000262136) | NQO1 divergent transcript [Source:HGNC Symbol;Acc:HGNC:55344] | 5,54 |
| NR4A2 | [ENSG00000153234](https://www.ensembl.org/id/ENSG00000153234) | nuclear receptor subfamily 4 group A member 2 [Source:HGNC Symbol;Acc:HGNC:7981] | 1,75 |
| NRAP | [ENSG00000197893](https://www.ensembl.org/id/ENSG00000197893) | nebulin related anchoring protein [Source:HGNC Symbol;Acc:HGNC:7988] | -9,67 |
| NRG4 | [ENSG00000169752](https://www.ensembl.org/id/ENSG00000169752) | neuregulin 4 [Source:HGNC Symbol;Acc:HGNC:29862] | -2,96 |
| NRIP2 | [ENSG00000053702](https://www.ensembl.org/id/ENSG00000053702) | nuclear receptor interacting protein 2 [Source:HGNC Symbol;Acc:HGNC:23078] | 1,83 |
| NT5C1A | [ENSG00000116981](https://www.ensembl.org/id/ENSG00000116981) | 5'-nucleotidase, cytosolic IA [Source:HGNC Symbol;Acc:HGNC:17819] | -2,00 |
| NTF4 | [ENSG00000225950](https://www.ensembl.org/id/ENSG00000225950) | neurotrophin 4 [Source:HGNC Symbol;Acc:HGNC:8024] | -5,97 |
| NTNG1 | [ENSG00000162631](https://www.ensembl.org/id/ENSG00000162631) | netrin G1 [Source:HGNC Symbol;Acc:HGNC:23319] | -2,73 |
| NUDT8 | [ENSG00000167799](https://www.ensembl.org/id/ENSG00000167799) | nudix hydrolase 8 [Source:HGNC Symbol;Acc:HGNC:8055] | -1,60 |
| NUTM2A | [ENSG00000184923](https://www.ensembl.org/id/ENSG00000184923) | NUT family member 2A [Source:HGNC Symbol;Acc:HGNC:23438] | -2,86 |
| OBSCN | [ENSG00000154358](https://www.ensembl.org/id/ENSG00000154358) | obscurin, cytoskeletal calmodulin and titin-interacting RhoGEF [Source:HGNC Symbol;Acc:HGNC:15719] | -4,59 |
| ODAM | [ENSG00000109205](https://www.ensembl.org/id/ENSG00000109205) | odontogenic, ameloblast associated [Source:HGNC Symbol;Acc:HGNC:26043] | -3,81 |
| ODF2-AS1 | [ENSG00000225951](https://www.ensembl.org/id/ENSG00000225951) | ODF2 antisense RNA 1 [Source:HGNC Symbol;Acc:HGNC:49461] | -5,24 |
| ODF3L2 | [ENSG00000181781](https://www.ensembl.org/id/ENSG00000181781) | outer dense fiber of sperm tails 3 like 2 [Source:HGNC Symbol;Acc:HGNC:26841] | -3,28 |
| OGDHL | [ENSG00000197444](https://www.ensembl.org/id/ENSG00000197444) | oxoglutarate dehydrogenase L [Source:HGNC Symbol;Acc:HGNC:25590] | -6,33 |
| OIT3 | [ENSG00000138315](https://www.ensembl.org/id/ENSG00000138315) | oncoprotein induced transcript 3 [Source:HGNC Symbol;Acc:HGNC:29953] | -3,52 |
| OLFM2 | [ENSG00000105088](https://www.ensembl.org/id/ENSG00000105088) | olfactomedin 2 [Source:HGNC Symbol;Acc:HGNC:17189] | -1,90 |
| OLFM4 | [ENSG00000102837](https://www.ensembl.org/id/ENSG00000102837) | olfactomedin 4 [Source:HGNC Symbol;Acc:HGNC:17190] | -2,06 |
| OPCML | [ENSG00000183715](https://www.ensembl.org/id/ENSG00000183715) | opioid binding protein/cell adhesion molecule like [Source:HGNC Symbol;Acc:HGNC:8143] | 2,71 |
| OPLAH | [ENSG00000178814](https://www.ensembl.org/id/ENSG00000178814) | 5-oxoprolinase, ATP-hydrolysing [Source:HGNC Symbol;Acc:HGNC:8149] | -2,17 |
| OPRD1 | [ENSG00000116329](https://www.ensembl.org/id/ENSG00000116329) | opioid receptor delta 1 [Source:HGNC Symbol;Acc:HGNC:8153] | -2,88 |
| OR10Z1 | [ENSG00000198967](https://www.ensembl.org/id/ENSG00000198967) | olfactory receptor family 10 subfamily Z member 1 [Source:HGNC Symbol;Acc:HGNC:14996] | -7,02 |
| OR2A1-AS1 | [ENSG00000244479](https://www.ensembl.org/id/ENSG00000244479) | OR2A1 antisense RNA 1 [Source:HGNC Symbol;Acc:HGNC:49168] | -2,20 |
| OR2A4 | [ENSG00000180658](https://www.ensembl.org/id/ENSG00000180658) | olfactory receptor family 2 subfamily A member 4 [Source:HGNC Symbol;Acc:HGNC:14729] | -4,04 |
| OR2W3 | [ENSG00000238243](https://www.ensembl.org/id/ENSG00000238243) | olfactory receptor family 2 subfamily W member 3 [Source:HGNC Symbol;Acc:HGNC:15021] | 3,89 |
| OR51E2 | [ENSG00000167332](https://www.ensembl.org/id/ENSG00000167332) | olfactory receptor family 51 subfamily E member 2 [Source:HGNC Symbol;Acc:HGNC:15195] | 2,28 |
| OR7E47P | [ENSG00000290965](https://www.ensembl.org/id/ENSG00000290965) | olfactory receptor family 7 subfamily E member 47 pseudogene [Source:NCBI gene (formerly Entrezgene);Acc:26628] | -4,69 |
| ORM1 | [ENSG00000229314](https://www.ensembl.org/id/ENSG00000229314) | orosomucoid 1 [Source:HGNC Symbol;Acc:HGNC:8498] | 3,60 |
| OSCAR | [ENSG00000170909](https://www.ensembl.org/id/ENSG00000170909) | osteoclast associated Ig-like receptor [Source:HGNC Symbol;Acc:HGNC:29960] | 3,47 |
| OSM | [ENSG00000099985](https://www.ensembl.org/id/ENSG00000099985) | oncostatin M [Source:HGNC Symbol;Acc:HGNC:8506] | 5,18 |
| OSTN | [ENSG00000188729](https://www.ensembl.org/id/ENSG00000188729) | osteocrin [Source:HGNC Symbol;Acc:HGNC:29961] | -3,41 |
| OVCH1-AS1 | [ENSG00000257599](https://www.ensembl.org/id/ENSG00000257599) | OVCH1 antisense RNA 1 [Source:HGNC Symbol;Acc:HGNC:44484] | 3,17 |
| P2RX1 | [ENSG00000108405](https://www.ensembl.org/id/ENSG00000108405) | purinergic receptor P2X 1 [Source:HGNC Symbol;Acc:HGNC:8533] | 3,46 |
| P2RX3 | [ENSG00000109991](https://www.ensembl.org/id/ENSG00000109991) | purinergic receptor P2X 3 [Source:HGNC Symbol;Acc:HGNC:8534] | -5,87 |
| P2RX6 | [ENSG00000099957](https://www.ensembl.org/id/ENSG00000099957) | purinergic receptor P2X 6 [Source:HGNC Symbol;Acc:HGNC:8538] | -2,89 |
| P2RY6 | [ENSG00000171631](https://www.ensembl.org/id/ENSG00000171631) | pyrimidinergic receptor P2Y6 [Source:HGNC Symbol;Acc:HGNC:8543] | 1,59 |
| PADI4 | [ENSG00000159339](https://www.ensembl.org/id/ENSG00000159339) | peptidyl arginine deiminase 4 [Source:HGNC Symbol;Acc:HGNC:18368] | 6,38 |
| PAQR9 | [ENSG00000188582](https://www.ensembl.org/id/ENSG00000188582) | progestin and adipoQ receptor family member 9 [Source:HGNC Symbol;Acc:HGNC:30131] | -3,41 |
| PARVB | [ENSG00000188677](https://www.ensembl.org/id/ENSG00000188677) | parvin beta [Source:HGNC Symbol;Acc:HGNC:14653] | -1,92 |
| PAX7 | [ENSG00000009709](https://www.ensembl.org/id/ENSG00000009709) | paired box 7 [Source:HGNC Symbol;Acc:HGNC:8621] | -6,84 |
| PAX8-AS1 | [ENSG00000189223](https://www.ensembl.org/id/ENSG00000189223) | PAX8 antisense RNA 1 [Source:HGNC Symbol;Acc:HGNC:49271] | 1,25 |
| PAX9 | [ENSG00000198807](https://www.ensembl.org/id/ENSG00000198807) | paired box 9 [Source:HGNC Symbol;Acc:HGNC:8623] | 2,11 |
| PBX3-DT | [ENSG00000229582](https://www.ensembl.org/id/ENSG00000229582) | PBX3 divergent transcript [Source:HGNC Symbol;Acc:HGNC:24638] | -4,48 |
| PC | [ENSG00000173599](https://www.ensembl.org/id/ENSG00000173599) | pyruvate carboxylase [Source:HGNC Symbol;Acc:HGNC:8636] | -1,93 |
| PCAT7 | [ENSG00000231806](https://www.ensembl.org/id/ENSG00000231806) | prostate cancer associated transcript 7 [Source:HGNC Symbol;Acc:HGNC:48824] | -4,92 |
| PCDHA6 | [ENSG00000081842](https://www.ensembl.org/id/ENSG00000081842) | protocadherin alpha 6 [Source:HGNC Symbol;Acc:HGNC:8672] | 4,45 |
| PCDHA8 | [ENSG00000204962](https://www.ensembl.org/id/ENSG00000204962) | protocadherin alpha 8 [Source:HGNC Symbol;Acc:HGNC:8674] | -5,40 |
| PCDHA9 | [ENSG00000204961](https://www.ensembl.org/id/ENSG00000204961) | protocadherin alpha 9 [Source:HGNC Symbol;Acc:HGNC:8675] | 4,87 |
| PCDHAC2 | [ENSG00000243232](https://www.ensembl.org/id/ENSG00000243232) | protocadherin alpha subfamily C, 2 [Source:HGNC Symbol;Acc:HGNC:8677] | -2,43 |
| PCDHB1-AS1 | [ENSG00000279047](https://www.ensembl.org/id/ENSG00000279047) | PCDHB1 antisense RNA 1 [Source:HGNC Symbol;Acc:HGNC:56111] | -3,89 |
| PCF11-AS1 | [ENSG00000269939](https://www.ensembl.org/id/ENSG00000269939) | PCF11 antisense RNA 1 [Source:HGNC Symbol;Acc:HGNC:52263] | -5,65 |
| PCP4 | [ENSG00000183036](https://www.ensembl.org/id/ENSG00000183036) | Purkinje cell protein 4 [Source:HGNC Symbol;Acc:HGNC:8742] | 2,47 |
| PCSK2 | [ENSG00000125851](https://www.ensembl.org/id/ENSG00000125851) | proprotein convertase subtilisin/kexin type 2 [Source:HGNC Symbol;Acc:HGNC:8744] | -2,82 |
| PDE11A | [ENSG00000128655](https://www.ensembl.org/id/ENSG00000128655) | phosphodiesterase 11A [Source:HGNC Symbol;Acc:HGNC:8773] | -3,38 |
| PDE1C | [ENSG00000154678](https://www.ensembl.org/id/ENSG00000154678) | phosphodiesterase 1C [Source:HGNC Symbol;Acc:HGNC:8776] | 1,95 |
| PDE3B | [ENSG00000152270](https://www.ensembl.org/id/ENSG00000152270) | phosphodiesterase 3B [Source:HGNC Symbol;Acc:HGNC:8779] | -2,74 |
| PDE4A | [ENSG00000065989](https://www.ensembl.org/id/ENSG00000065989) | phosphodiesterase 4A [Source:HGNC Symbol;Acc:HGNC:8780] | -1,08 |
| PDE4B | [ENSG00000184588](https://www.ensembl.org/id/ENSG00000184588) | phosphodiesterase 4B [Source:HGNC Symbol;Acc:HGNC:8781] | 1,63 |
| PDE7A-DT | [ENSG00000272192](https://www.ensembl.org/id/ENSG00000272192) | PDE7A divergent transcript [Source:HGNC Symbol;Acc:HGNC:55223] | -3,44 |
| PDGFD | [ENSG00000170962](https://www.ensembl.org/id/ENSG00000170962) | platelet derived growth factor D [Source:HGNC Symbol;Acc:HGNC:30620] | -1,54 |
| PDGFRA | [ENSG00000134853](https://www.ensembl.org/id/ENSG00000134853) | platelet derived growth factor receptor alpha [Source:HGNC Symbol;Acc:HGNC:8803] | -1,42 |
| PDZD3 | [ENSG00000172367](https://www.ensembl.org/id/ENSG00000172367) | PDZ domain containing 3 [Source:HGNC Symbol;Acc:HGNC:19891] | -3,67 |
| PDZRN4 | [ENSG00000165966](https://www.ensembl.org/id/ENSG00000165966) | PDZ domain containing ring finger 4 [Source:HGNC Symbol;Acc:HGNC:30552] | 1,20 |
| PELATON | [ENSG00000224397](https://www.ensembl.org/id/ENSG00000224397) | plaque enriched lncRNA in atherosclerotic and inflammatory bowel macrophage regulation [Source:HGNC Symbol;Acc:HGNC:50328] | 3,64 |
| PENK | [ENSG00000181195](https://www.ensembl.org/id/ENSG00000181195) | proenkephalin [Source:HGNC Symbol;Acc:HGNC:8831] | -2,73 |
| PERM1 | [ENSG00000187642](https://www.ensembl.org/id/ENSG00000187642) | PPARGC1 and ESRR induced regulator, muscle 1 [Source:HGNC Symbol;Acc:HGNC:28208] | -3,28 |
| PEX5L | [ENSG00000114757](https://www.ensembl.org/id/ENSG00000114757) | peroxisomal biogenesis factor 5 like [Source:HGNC Symbol;Acc:HGNC:30024] | -2,93 |
| PF4 | [ENSG00000163737](https://www.ensembl.org/id/ENSG00000163737) | platelet factor 4 [Source:HGNC Symbol;Acc:HGNC:8861] | 3,63 |
| PFKFB1 | [ENSG00000158571](https://www.ensembl.org/id/ENSG00000158571) | 6-phosphofructo-2-kinase/fructose-2,6-biphosphatase 1 [Source:HGNC Symbol;Acc:HGNC:8872] | -4,36 |
| PGA4 | [ENSG00000229183](https://www.ensembl.org/id/ENSG00000229183) | pepsinogen A4 [Source:HGNC Symbol;Acc:HGNC:8886] | -4,12 |
| PGAM2 | [ENSG00000164708](https://www.ensembl.org/id/ENSG00000164708) | phosphoglycerate mutase 2 [Source:HGNC Symbol;Acc:HGNC:8889] | -2,45 |
| PGAP4 | [ENSG00000165152](https://www.ensembl.org/id/ENSG00000165152) | post-GPI attachment to proteins GalNAc transferase 4 [Source:HGNC Symbol;Acc:HGNC:28180] | 1,06 |
| PGBD5 | [ENSG00000177614](https://www.ensembl.org/id/ENSG00000177614) | piggyBac transposable element derived 5 [Source:HGNC Symbol;Acc:HGNC:19405] | -2,77 |
| PGF | [ENSG00000119630](https://www.ensembl.org/id/ENSG00000119630) | placental growth factor [Source:HGNC Symbol;Acc:HGNC:8893] | 1,05 |
| PGLYRP1 | [ENSG00000008438](https://www.ensembl.org/id/ENSG00000008438) | peptidoglycan recognition protein 1 [Source:HGNC Symbol;Acc:HGNC:8904] | 6,74 |
| PHACTR3 | [ENSG00000087495](https://www.ensembl.org/id/ENSG00000087495) | phosphatase and actin regulator 3 [Source:HGNC Symbol;Acc:HGNC:15833] | -2,83 |
| PHKA1 | [ENSG00000067177](https://www.ensembl.org/id/ENSG00000067177) | phosphorylase kinase regulatory subunit alpha 1 [Source:HGNC Symbol;Acc:HGNC:8925] | -1,26 |
| PHKG1 | [ENSG00000164776](https://www.ensembl.org/id/ENSG00000164776) | phosphorylase kinase catalytic subunit gamma 1 [Source:HGNC Symbol;Acc:HGNC:8930] | -1,43 |
| PHLDB1 | [ENSG00000019144](https://www.ensembl.org/id/ENSG00000019144) | pleckstrin homology like domain family B member 1 [Source:HGNC Symbol;Acc:HGNC:23697] | -0,99 |
| PI3 | [ENSG00000124102](https://www.ensembl.org/id/ENSG00000124102) | peptidase inhibitor 3 [Source:HGNC Symbol;Acc:HGNC:8947] | 3,55 |
| PIANP | [ENSG00000139200](https://www.ensembl.org/id/ENSG00000139200) | PILR alpha associated neural protein [Source:HGNC Symbol;Acc:HGNC:25338] | -2,35 |
| PIEZO2 | [ENSG00000154864](https://www.ensembl.org/id/ENSG00000154864) | piezo type mechanosensitive ion channel component 2 [Source:HGNC Symbol;Acc:HGNC:26270] | -2,26 |
| PIGA | [ENSG00000165195](https://www.ensembl.org/id/ENSG00000165195) | phosphatidylinositol glycan anchor biosynthesis class A [Source:HGNC Symbol;Acc:HGNC:8957] | 1,54 |
| PIMREG | [ENSG00000129195](https://www.ensembl.org/id/ENSG00000129195) | PICALM interacting mitotic regulator [Source:HGNC Symbol;Acc:HGNC:25483] | -3,60 |
| PITX2 | [ENSG00000164093](https://www.ensembl.org/id/ENSG00000164093) | paired like homeodomain 2 [Source:HGNC Symbol;Acc:HGNC:9005] | -6,09 |
| PITX3 | [ENSG00000107859](https://www.ensembl.org/id/ENSG00000107859) | paired like homeodomain 3 [Source:HGNC Symbol;Acc:HGNC:9006] | -3,88 |
| PKD2L1 | [ENSG00000107593](https://www.ensembl.org/id/ENSG00000107593) | polycystin 2 like 1, transient receptor potential cation channel [Source:HGNC Symbol;Acc:HGNC:9011] | -5,43 |
| PKD2L2-DT | [ENSG00000250159](https://www.ensembl.org/id/ENSG00000250159) | PKD2L2 divergent transcript [Source:HGNC Symbol;Acc:HGNC:55557] | -3,31 |
| PKHD1L1 | [ENSG00000205038](https://www.ensembl.org/id/ENSG00000205038) | PKHD1 like 1 [Source:HGNC Symbol;Acc:HGNC:20313] | -2,65 |
| PKIA | [ENSG00000171033](https://www.ensembl.org/id/ENSG00000171033) | cAMP-dependent protein kinase inhibitor alpha [Source:HGNC Symbol;Acc:HGNC:9017] | -5,22 |
| PKP2 | [ENSG00000057294](https://www.ensembl.org/id/ENSG00000057294) | plakophilin 2 [Source:HGNC Symbol;Acc:HGNC:9024] | -3,47 |
| PLA2G4F | [ENSG00000168907](https://www.ensembl.org/id/ENSG00000168907) | phospholipase A2 group IVF [Source:HGNC Symbol;Acc:HGNC:27396] | -5,28 |
| PLAAT1 | [ENSG00000127252](https://www.ensembl.org/id/ENSG00000127252) | phospholipase A and acyltransferase 1 [Source:HGNC Symbol;Acc:HGNC:14922] | -4,28 |
| PLCD4 | [ENSG00000115556](https://www.ensembl.org/id/ENSG00000115556) | phospholipase C delta 4 [Source:HGNC Symbol;Acc:HGNC:9062] | -2,65 |
| PLCE1 | [ENSG00000138193](https://www.ensembl.org/id/ENSG00000138193) | phospholipase C epsilon 1 [Source:HGNC Symbol;Acc:HGNC:17175] | 1,15 |
| PLEK2 | [ENSG00000100558](https://www.ensembl.org/id/ENSG00000100558) | pleckstrin 2 [Source:HGNC Symbol;Acc:HGNC:19238] | 3,47 |
| PLIN1 | [ENSG00000166819](https://www.ensembl.org/id/ENSG00000166819) | perilipin 1 [Source:HGNC Symbol;Acc:HGNC:9076] | -2,60 |
| PLIN4 | [ENSG00000167676](https://www.ensembl.org/id/ENSG00000167676) | perilipin 4 [Source:HGNC Symbol;Acc:HGNC:29393] | -2,54 |
| PLIN5 | [ENSG00000214456](https://www.ensembl.org/id/ENSG00000214456) | perilipin 5 [Source:HGNC Symbol;Acc:HGNC:33196] | -3,12 |
| PLP1 | [ENSG00000123560](https://www.ensembl.org/id/ENSG00000123560) | proteolipid protein 1 [Source:HGNC Symbol;Acc:HGNC:9086] | -1,75 |
| PLXNA4 | [ENSG00000221866](https://www.ensembl.org/id/ENSG00000221866) | plexin A4 [Source:HGNC Symbol;Acc:HGNC:9102] | -1,60 |
| PNCK | [ENSG00000130822](https://www.ensembl.org/id/ENSG00000130822) | pregnancy up-regulated nonubiquitous CaM kinase [Source:HGNC Symbol;Acc:HGNC:13415] | 1,76 |
| PNMA1 | [ENSG00000176903](https://www.ensembl.org/id/ENSG00000176903) | PNMA family member 1 [Source:HGNC Symbol;Acc:HGNC:9158] | 0,96 |
| PNMA6A | [ENSG00000235961](https://www.ensembl.org/id/ENSG00000235961) | PNMA family member 6A [Source:HGNC Symbol;Acc:HGNC:28248] | -1,74 |
| POPDC2 | [ENSG00000121577](https://www.ensembl.org/id/ENSG00000121577) | popeye domain containing 2 [Source:HGNC Symbol;Acc:HGNC:17648] | -2,07 |
| POPDC3 | [ENSG00000132429](https://www.ensembl.org/id/ENSG00000132429) | popeye domain containing 3 [Source:HGNC Symbol;Acc:HGNC:17649] | -5,13 |
| POTEH-AS1 | [ENSG00000236666](https://www.ensembl.org/id/ENSG00000236666) | POTEH antisense RNA 1 [Source:HGNC Symbol;Acc:HGNC:40058] | -5,10 |
| POTEM | [ENSG00000222036](https://www.ensembl.org/id/ENSG00000222036) | POTE ankyrin domain family member M [Source:HGNC Symbol;Acc:HGNC:37096] | -4,89 |
| POU2AF2 | [ENSG00000150750](https://www.ensembl.org/id/ENSG00000150750) | POU class 2 homeobox associating factor 2 [Source:HGNC Symbol;Acc:HGNC:30527] | -6,20 |
| PPBP | [ENSG00000163736](https://www.ensembl.org/id/ENSG00000163736) | pro-platelet basic protein [Source:HGNC Symbol;Acc:HGNC:9240] | 4,47 |
| PPDPFL | [ENSG00000168333](https://www.ensembl.org/id/ENSG00000168333) | pancreatic progenitor cell differentiation and proliferation factor like [Source:HGNC Symbol;Acc:HGNC:31745] | -9,34 |
| PPFIA4 | [ENSG00000143847](https://www.ensembl.org/id/ENSG00000143847) | PTPRF interacting protein alpha 4 [Source:HGNC Symbol;Acc:HGNC:9248] | -1,63 |
| PPL | [ENSG00000118898](https://www.ensembl.org/id/ENSG00000118898) | periplakin [Source:HGNC Symbol;Acc:HGNC:9273] | -1,18 |
| PPM1J | [ENSG00000155367](https://www.ensembl.org/id/ENSG00000155367) | protein phosphatase, Mg2+/Mn2+ dependent 1J [Source:HGNC Symbol;Acc:HGNC:20785] | -1,35 |
| PPP1R14C | [ENSG00000198729](https://www.ensembl.org/id/ENSG00000198729) | protein phosphatase 1 regulatory inhibitor subunit 14C [Source:HGNC Symbol;Acc:HGNC:14952] | -3,48 |
| PPP1R1A | [ENSG00000135447](https://www.ensembl.org/id/ENSG00000135447) | protein phosphatase 1 regulatory inhibitor subunit 1A [Source:HGNC Symbol;Acc:HGNC:9286] | -2,19 |
| PPP1R1B | [ENSG00000131771](https://www.ensembl.org/id/ENSG00000131771) | protein phosphatase 1 regulatory inhibitor subunit 1B [Source:HGNC Symbol;Acc:HGNC:9287] | -4,24 |
| PPP1R27 | [ENSG00000182676](https://www.ensembl.org/id/ENSG00000182676) | protein phosphatase 1 regulatory subunit 27 [Source:HGNC Symbol;Acc:HGNC:16813] | -7,87 |
| PPP1R3A | [ENSG00000154415](https://www.ensembl.org/id/ENSG00000154415) | protein phosphatase 1 regulatory subunit 3A [Source:HGNC Symbol;Acc:HGNC:9291] | -8,61 |
| PPP1R42 | [ENSG00000178125](https://www.ensembl.org/id/ENSG00000178125) | protein phosphatase 1 regulatory subunit 42 [Source:HGNC Symbol;Acc:HGNC:33732] | -4,93 |
| PPP2R3B | [ENSG00000167393](https://www.ensembl.org/id/ENSG00000167393) | protein phosphatase 2 regulatory subunit B''beta [Source:HGNC Symbol;Acc:HGNC:13417] | -1,65 |
| PPP4R4 | [ENSG00000119698](https://www.ensembl.org/id/ENSG00000119698) | protein phosphatase 4 regulatory subunit 4 [Source:HGNC Symbol;Acc:HGNC:23788] | 4,97 |
| PRAG1 | [ENSG00000275342](https://www.ensembl.org/id/ENSG00000275342) | PEAK1 related, kinase-activating pseudokinase 1 [Source:HGNC Symbol;Acc:HGNC:25438] | -1,96 |
| PRAP1 | [ENSG00000165828](https://www.ensembl.org/id/ENSG00000165828) | proline rich acidic protein 1 [Source:HGNC Symbol;Acc:HGNC:23304] | -2,95 |
| PRKAG3 | [ENSG00000115592](https://www.ensembl.org/id/ENSG00000115592) | protein kinase AMP-activated non-catalytic subunit gamma 3 [Source:HGNC Symbol;Acc:HGNC:9387] | -4,44 |
| PRKAR2B | [ENSG00000005249](https://www.ensembl.org/id/ENSG00000005249) | protein kinase cAMP-dependent type II regulatory subunit beta [Source:HGNC Symbol;Acc:HGNC:9392] | -1,70 |
| PRKCA | [ENSG00000154229](https://www.ensembl.org/id/ENSG00000154229) | protein kinase C alpha [Source:HGNC Symbol;Acc:HGNC:9393] | 1,31 |
| PRKCQ | [ENSG00000065675](https://www.ensembl.org/id/ENSG00000065675) | protein kinase C theta [Source:HGNC Symbol;Acc:HGNC:9410] | -3,88 |
| PRKCQ-AS1 | [ENSG00000237943](https://www.ensembl.org/id/ENSG00000237943) | PRKCQ antisense RNA 1 [Source:HGNC Symbol;Acc:HGNC:44689] | -2,97 |
| PRKG2 | [ENSG00000138669](https://www.ensembl.org/id/ENSG00000138669) | protein kinase cGMP-dependent 2 [Source:HGNC Symbol;Acc:HGNC:9416] | -2,84 |
| PRMT9 | [ENSG00000164169](https://www.ensembl.org/id/ENSG00000164169) | protein arginine methyltransferase 9 [Source:HGNC Symbol;Acc:HGNC:25099] | 1,26 |
| PROB1 | [ENSG00000228672](https://www.ensembl.org/id/ENSG00000228672) | proline rich basic protein 1 [Source:HGNC Symbol;Acc:HGNC:41906] | -2,10 |
| PROK2 | [ENSG00000163421](https://www.ensembl.org/id/ENSG00000163421) | prokineticin 2 [Source:HGNC Symbol;Acc:HGNC:18455] | 5,80 |
| PRR15 | [ENSG00000176532](https://www.ensembl.org/id/ENSG00000176532) | proline rich 15 [Source:HGNC Symbol;Acc:HGNC:22310] | -2,66 |
| PRR15-DT | [ENSG00000223813](https://www.ensembl.org/id/ENSG00000223813) | PRR15 divergent transcript [Source:HGNC Symbol;Acc:HGNC:55866] | -6,96 |
| PRR32 | [ENSG00000183631](https://www.ensembl.org/id/ENSG00000183631) | proline rich 32 [Source:HGNC Symbol;Acc:HGNC:34498] | -8,69 |
| PRR5 | [ENSG00000186654](https://www.ensembl.org/id/ENSG00000186654) | proline rich 5 [Source:HGNC Symbol;Acc:HGNC:31682] | -1,71 |
| PRR9 | [ENSG00000203783](https://www.ensembl.org/id/ENSG00000203783) | proline rich 9 [Source:HGNC Symbol;Acc:HGNC:32057] | -4,83 |
| PRSS56 | [ENSG00000237412](https://www.ensembl.org/id/ENSG00000237412) | serine protease 56 [Source:HGNC Symbol;Acc:HGNC:39433] | -8,66 |
| PRTN3 | [ENSG00000196415](https://www.ensembl.org/id/ENSG00000196415) | proteinase 3 [Source:HGNC Symbol;Acc:HGNC:9495] | -4,06 |
| PRXL2A | [ENSG00000122378](https://www.ensembl.org/id/ENSG00000122378) | peroxiredoxin like 2A [Source:HGNC Symbol;Acc:HGNC:28651] | -1,28 |
| PTCRA | [ENSG00000171611](https://www.ensembl.org/id/ENSG00000171611) | pre T cell antigen receptor alpha [Source:HGNC Symbol;Acc:HGNC:21290] | 7,04 |
| PTGDR2 | [ENSG00000183134](https://www.ensembl.org/id/ENSG00000183134) | prostaglandin D2 receptor 2 [Source:HGNC Symbol;Acc:HGNC:4502] | -3,19 |
| PTPRC | [ENSG00000081237](https://www.ensembl.org/id/ENSG00000081237) | protein tyrosine phosphatase receptor type C [Source:HGNC Symbol;Acc:HGNC:9666] | 2,17 |
| PTPRQ | [ENSG00000139304](https://www.ensembl.org/id/ENSG00000139304) | protein tyrosine phosphatase receptor type Q [Source:HGNC Symbol;Acc:HGNC:9679] | -2,87 |
| PVALB | [ENSG00000100362](https://www.ensembl.org/id/ENSG00000100362) | parvalbumin [Source:HGNC Symbol;Acc:HGNC:9704] | -5,28 |
| PVRIG | [ENSG00000213413](https://www.ensembl.org/id/ENSG00000213413) | PVR related immunoglobulin domain containing [Source:HGNC Symbol;Acc:HGNC:32190] | 3,71 |
| PWWP3B | [ENSG00000157502](https://www.ensembl.org/id/ENSG00000157502) | PWWP domain containing 3B [Source:HGNC Symbol;Acc:HGNC:26583] | -2,87 |
| PYGM | [ENSG00000068976](https://www.ensembl.org/id/ENSG00000068976) | glycogen phosphorylase, muscle associated [Source:HGNC Symbol;Acc:HGNC:9726] | -3,09 |
| QRFPR | [ENSG00000186867](https://www.ensembl.org/id/ENSG00000186867) | pyroglutamylated RFamide peptide receptor [Source:HGNC Symbol;Acc:HGNC:15565] | 5,80 |
| RAB27B | [ENSG00000041353](https://www.ensembl.org/id/ENSG00000041353) | RAB27B, member RAS oncogene family [Source:HGNC Symbol;Acc:HGNC:9767] | -2,58 |
| RAB3A | [ENSG00000105649](https://www.ensembl.org/id/ENSG00000105649) | RAB3A, member RAS oncogene family [Source:HGNC Symbol;Acc:HGNC:9777] | 1,65 |
| RAB40AL | [ENSG00000102128](https://www.ensembl.org/id/ENSG00000102128) | RAB40A like [Source:HGNC Symbol;Acc:HGNC:25410] | -6,46 |
| RANBP3-DT | [ENSG00000266983](https://www.ensembl.org/id/ENSG00000266983) | RANBP3 divergent transcript [Source:HGNC Symbol;Acc:HGNC:55312] | 4,48 |
| RAP1GAP | [ENSG00000076864](https://www.ensembl.org/id/ENSG00000076864) | RAP1 GTPase activating protein [Source:HGNC Symbol;Acc:HGNC:9858] | 2,20 |
| RAPSN | [ENSG00000165917](https://www.ensembl.org/id/ENSG00000165917) | receptor associated protein of the synapse [Source:HGNC Symbol;Acc:HGNC:9863] | -5,17 |
| RARRES1 | [ENSG00000118849](https://www.ensembl.org/id/ENSG00000118849) | retinoic acid receptor responder 1 [Source:HGNC Symbol;Acc:HGNC:9867] | -1,88 |
| RASD2 | [ENSG00000100302](https://www.ensembl.org/id/ENSG00000100302) | RASD family member 2 [Source:HGNC Symbol;Acc:HGNC:18229] | -2,79 |
| RASL10B | [ENSG00000270885](https://www.ensembl.org/id/ENSG00000270885) | RAS like family 10 member B [Source:HGNC Symbol;Acc:HGNC:30295] | -2,12 |
| RASSF6 | [ENSG00000169435](https://www.ensembl.org/id/ENSG00000169435) | Ras association domain family member 6 [Source:HGNC Symbol;Acc:HGNC:20796] | -5,44 |
| RBFOX1 | [ENSG00000078328](https://www.ensembl.org/id/ENSG00000078328) | RNA binding fox-1 homolog 1 [Source:HGNC Symbol;Acc:HGNC:18222] | -8,53 |
| RBKS | [ENSG00000171174](https://www.ensembl.org/id/ENSG00000171174) | ribokinase [Source:HGNC Symbol;Acc:HGNC:30325] | 1,41 |
| RBP4 | [ENSG00000138207](https://www.ensembl.org/id/ENSG00000138207) | retinol binding protein 4 [Source:HGNC Symbol;Acc:HGNC:9922] | -2,53 |
| RBPMS2 | [ENSG00000166831](https://www.ensembl.org/id/ENSG00000166831) | RNA binding protein, mRNA processing factor 2 [Source:HGNC Symbol;Acc:HGNC:19098] | 1,53 |
| REC8 | [ENSG00000100918](https://www.ensembl.org/id/ENSG00000100918) | REC8 meiotic recombination protein [Source:HGNC Symbol;Acc:HGNC:16879] | -1,51 |
| REEP2 | [ENSG00000132563](https://www.ensembl.org/id/ENSG00000132563) | receptor accessory protein 2 [Source:HGNC Symbol;Acc:HGNC:17975] | -1,28 |
| RENBP | [ENSG00000102032](https://www.ensembl.org/id/ENSG00000102032) | renin binding protein [Source:HGNC Symbol;Acc:HGNC:9959] | 1,26 |
| RERGL | [ENSG00000111404](https://www.ensembl.org/id/ENSG00000111404) | RERG like [Source:HGNC Symbol;Acc:HGNC:26213] | 1,58 |
| RETREG1 | [ENSG00000154153](https://www.ensembl.org/id/ENSG00000154153) | reticulophagy regulator 1 [Source:HGNC Symbol;Acc:HGNC:25964] | -2,58 |
| RFLNA | [ENSG00000178882](https://www.ensembl.org/id/ENSG00000178882) | refilin A [Source:HGNC Symbol;Acc:HGNC:27051] | -2,06 |
| RFX8 | [ENSG00000196460](https://www.ensembl.org/id/ENSG00000196460) | regulatory factor X8 [Source:HGNC Symbol;Acc:HGNC:37253] | -2,70 |
| RGL4 | [ENSG00000159496](https://www.ensembl.org/id/ENSG00000159496) | ral guanine nucleotide dissociation stimulator like 4 [Source:HGNC Symbol;Acc:HGNC:31911] | 4,56 |
| RGPD4 | [ENSG00000196862](https://www.ensembl.org/id/ENSG00000196862) | RANBP2 like and GRIP domain containing 4 [Source:HGNC Symbol;Acc:HGNC:32417] | -2,11 |
| RGS11 | [ENSG00000076344](https://www.ensembl.org/id/ENSG00000076344) | regulator of G protein signaling 11 [Source:HGNC Symbol;Acc:HGNC:9993] | -1,13 |
| RGS16 | [ENSG00000143333](https://www.ensembl.org/id/ENSG00000143333) | regulator of G protein signaling 16 [Source:HGNC Symbol;Acc:HGNC:9997] | 1,50 |
| RGS18 | [ENSG00000150681](https://www.ensembl.org/id/ENSG00000150681) | regulator of G protein signaling 18 [Source:HGNC Symbol;Acc:HGNC:14261] | 4,46 |
| RGS19 | [ENSG00000171700](https://www.ensembl.org/id/ENSG00000171700) | regulator of G protein signaling 19 [Source:HGNC Symbol;Acc:HGNC:13735] | 1,59 |
| RGS2 | [ENSG00000116741](https://www.ensembl.org/id/ENSG00000116741) | regulator of G protein signaling 2 [Source:HGNC Symbol;Acc:HGNC:9998] | 1,80 |
| RGS22 | [ENSG00000132554](https://www.ensembl.org/id/ENSG00000132554) | regulator of G protein signaling 22 [Source:HGNC Symbol;Acc:HGNC:24499] | -1,87 |
| RGS4 | [ENSG00000117152](https://www.ensembl.org/id/ENSG00000117152) | regulator of G protein signaling 4 [Source:HGNC Symbol;Acc:HGNC:10000] | -1,70 |
| RGS6 | [ENSG00000182732](https://www.ensembl.org/id/ENSG00000182732) | regulator of G protein signaling 6 [Source:HGNC Symbol;Acc:HGNC:10002] | 1,80 |
| RGS9BP | [ENSG00000186326](https://www.ensembl.org/id/ENSG00000186326) | regulator of G protein signaling 9 binding protein [Source:HGNC Symbol;Acc:HGNC:30304] | -5,49 |
| RHD | [ENSG00000187010](https://www.ensembl.org/id/ENSG00000187010) | Rh blood group D antigen [Source:HGNC Symbol;Acc:HGNC:10009] | 4,02 |
| RHOV | [ENSG00000104140](https://www.ensembl.org/id/ENSG00000104140) | ras homolog family member V [Source:HGNC Symbol;Acc:HGNC:18313] | -3,42 |
| RHPN2 | [ENSG00000131941](https://www.ensembl.org/id/ENSG00000131941) | rhophilin Rho GTPase binding protein 2 [Source:HGNC Symbol;Acc:HGNC:19974] | -2,18 |
| RNF128 | [ENSG00000133135](https://www.ensembl.org/id/ENSG00000133135) | ring finger protein 128 [Source:HGNC Symbol;Acc:HGNC:21153] | -7,69 |
| RNF144B | [ENSG00000137393](https://www.ensembl.org/id/ENSG00000137393) | ring finger protein 144B [Source:HGNC Symbol;Acc:HGNC:21578] | -1,52 |
| RNF152 | [ENSG00000176641](https://www.ensembl.org/id/ENSG00000176641) | ring finger protein 152 [Source:HGNC Symbol;Acc:HGNC:26811] | 1,04 |
| RNF157 | [ENSG00000141576](https://www.ensembl.org/id/ENSG00000141576) | ring finger protein 157 [Source:HGNC Symbol;Acc:HGNC:29402] | -2,73 |
| ROPN1L | [ENSG00000145491](https://www.ensembl.org/id/ENSG00000145491) | rhophilin associated tail protein 1 like [Source:HGNC Symbol;Acc:HGNC:24060] | 3,10 |
| RORC | [ENSG00000143365](https://www.ensembl.org/id/ENSG00000143365) | RAR related orphan receptor C [Source:HGNC Symbol;Acc:HGNC:10260] | -5,81 |
| RPH3AL-AS2 | [ENSG00000262294](https://www.ensembl.org/id/ENSG00000262294) | RPH3AL antisense RNA 2 [Source:HGNC Symbol;Acc:HGNC:56089] | -4,84 |
| RPL3L | [ENSG00000140986](https://www.ensembl.org/id/ENSG00000140986) | ribosomal protein L3 like [Source:HGNC Symbol;Acc:HGNC:10351] | -8,61 |
| RPP38-DT | [ENSG00000176236](https://www.ensembl.org/id/ENSG00000176236) | RPP38 divergent transcript [Source:HGNC Symbol;Acc:HGNC:28582] | -2,91 |
| RPRM | [ENSG00000177519](https://www.ensembl.org/id/ENSG00000177519) | reprimo, TP53 dependent G2 arrest mediator homolog [Source:HGNC Symbol;Acc:HGNC:24201] | -2,70 |
| RPRML | [ENSG00000179673](https://www.ensembl.org/id/ENSG00000179673) | reprimo like [Source:HGNC Symbol;Acc:HGNC:32422] | -2,55 |
| RSAD2 | [ENSG00000134321](https://www.ensembl.org/id/ENSG00000134321) | radical S-adenosyl methionine domain containing 2 [Source:HGNC Symbol;Acc:HGNC:30908] | -1,42 |
| RSPH6A | [ENSG00000104941](https://www.ensembl.org/id/ENSG00000104941) | radial spoke head 6 homolog A [Source:HGNC Symbol;Acc:HGNC:14241] | -5,16 |
| RSPO1 | [ENSG00000169218](https://www.ensembl.org/id/ENSG00000169218) | R-spondin 1 [Source:HGNC Symbol;Acc:HGNC:21679] | -4,11 |
| RTN2 | [ENSG00000125744](https://www.ensembl.org/id/ENSG00000125744) | reticulon 2 [Source:HGNC Symbol;Acc:HGNC:10468] | -2,57 |
| RTP2 | [ENSG00000198471](https://www.ensembl.org/id/ENSG00000198471) | receptor transporter protein 2 [Source:HGNC Symbol;Acc:HGNC:32486] | -5,99 |
| RUBCNL | [ENSG00000102445](https://www.ensembl.org/id/ENSG00000102445) | rubicon like autophagy enhancer [Source:HGNC Symbol;Acc:HGNC:20420] | 3,09 |
| RYR1 | [ENSG00000196218](https://www.ensembl.org/id/ENSG00000196218) | ryanodine receptor 1 [Source:HGNC Symbol;Acc:HGNC:10483] | -6,50 |
| S100A1 | [ENSG00000160678](https://www.ensembl.org/id/ENSG00000160678) | S100 calcium binding protein A1 [Source:HGNC Symbol;Acc:HGNC:10486] | -5,74 |
| S100A12 | [ENSG00000163221](https://www.ensembl.org/id/ENSG00000163221) | S100 calcium binding protein A12 [Source:HGNC Symbol;Acc:HGNC:10489] | 4,19 |
| S100A8 | [ENSG00000143546](https://www.ensembl.org/id/ENSG00000143546) | S100 calcium binding protein A8 [Source:HGNC Symbol;Acc:HGNC:10498] | 4,89 |
| S100A9 | [ENSG00000163220](https://www.ensembl.org/id/ENSG00000163220) | S100 calcium binding protein A9 [Source:HGNC Symbol;Acc:HGNC:10499] | 4,70 |
| S100B | [ENSG00000160307](https://www.ensembl.org/id/ENSG00000160307) | S100 calcium binding protein B [Source:HGNC Symbol;Acc:HGNC:10500] | -1,83 |
| S100P | [ENSG00000163993](https://www.ensembl.org/id/ENSG00000163993) | S100 calcium binding protein P [Source:HGNC Symbol;Acc:HGNC:10504] | 5,90 |
| S1PR5 | [ENSG00000180739](https://www.ensembl.org/id/ENSG00000180739) | sphingosine-1-phosphate receptor 5 [Source:HGNC Symbol;Acc:HGNC:14299] | 2,70 |
| SAA1 | [ENSG00000173432](https://www.ensembl.org/id/ENSG00000173432) | serum amyloid A1 [Source:HGNC Symbol;Acc:HGNC:10513] | -2,08 |
| SAA2 | [ENSG00000134339](https://www.ensembl.org/id/ENSG00000134339) | serum amyloid A2 [Source:HGNC Symbol;Acc:HGNC:10514] | -3,44 |
| SAMMSON | [ENSG00000240405](https://www.ensembl.org/id/ENSG00000240405) | survival associated mitochondrial melanoma specific oncogenic non-coding RNA [Source:HGNC Symbol;Acc:HGNC:49644] | -2,52 |
| SAMSN1 | [ENSG00000155307](https://www.ensembl.org/id/ENSG00000155307) | SAM domain, SH3 domain and nuclear localization signals 1 [Source:HGNC Symbol;Acc:HGNC:10528] | 3,11 |
| SBK1 | [ENSG00000188322](https://www.ensembl.org/id/ENSG00000188322) | SH3 domain binding kinase 1 [Source:HGNC Symbol;Acc:HGNC:17699] | -2,62 |
| SBK2 | [ENSG00000187550](https://www.ensembl.org/id/ENSG00000187550) | SH3 domain binding kinase family member 2 [Source:HGNC Symbol;Acc:HGNC:34416] | -7,13 |
| SBK3 | [ENSG00000231274](https://www.ensembl.org/id/ENSG00000231274) | SH3 domain binding kinase family member 3 [Source:HGNC Symbol;Acc:HGNC:44121] | -2,86 |
| SCARNA10 | [ENSG00000239002](https://www.ensembl.org/id/ENSG00000239002) | small Cajal body-specific RNA 10 [Source:HGNC Symbol;Acc:HGNC:32567] | -3,57 |
| SCEL | [ENSG00000136155](https://www.ensembl.org/id/ENSG00000136155) | sciellin [Source:HGNC Symbol;Acc:HGNC:10573] | -4,11 |
| SCG5 | [ENSG00000166922](https://www.ensembl.org/id/ENSG00000166922) | secretogranin V [Source:HGNC Symbol;Acc:HGNC:10816] | -2,85 |
| SCIRT | [ENSG00000237686](https://www.ensembl.org/id/ENSG00000237686) | stem cell inhibitory RNA transcript [Source:HGNC Symbol;Acc:HGNC:55341] | -3,93 |
| SCML4 | [ENSG00000146285](https://www.ensembl.org/id/ENSG00000146285) | Scm polycomb group protein like 4 [Source:HGNC Symbol;Acc:HGNC:21397] | 3,27 |
| SCN1B | [ENSG00000105711](https://www.ensembl.org/id/ENSG00000105711) | sodium voltage-gated channel beta subunit 1 [Source:HGNC Symbol;Acc:HGNC:10586] | -2,24 |
| SCN2B | [ENSG00000149575](https://www.ensembl.org/id/ENSG00000149575) | sodium voltage-gated channel beta subunit 2 [Source:HGNC Symbol;Acc:HGNC:10589] | -2,39 |
| SCN3B | [ENSG00000166257](https://www.ensembl.org/id/ENSG00000166257) | sodium voltage-gated channel beta subunit 3 [Source:HGNC Symbol;Acc:HGNC:20665] | -2,46 |
| SCN4A | [ENSG00000007314](https://www.ensembl.org/id/ENSG00000007314) | sodium voltage-gated channel alpha subunit 4 [Source:HGNC Symbol;Acc:HGNC:10591] | -2,91 |
| SCN7A | [ENSG00000136546](https://www.ensembl.org/id/ENSG00000136546) | sodium voltage-gated channel alpha subunit 7 [Source:HGNC Symbol;Acc:HGNC:10594] | -2,12 |
| SCTR-AS1 | [ENSG00000231013](https://www.ensembl.org/id/ENSG00000231013) | SCTR antisense RNA 1 [Source:HGNC Symbol;Acc:HGNC:40516] | -3,22 |
| SEC16B | [ENSG00000120341](https://www.ensembl.org/id/ENSG00000120341) | SEC16 homolog B, endoplasmic reticulum export factor [Source:HGNC Symbol;Acc:HGNC:30301] | -2,01 |
| SELL | [ENSG00000188404](https://www.ensembl.org/id/ENSG00000188404) | selectin L [Source:HGNC Symbol;Acc:HGNC:10720] | 2,40 |
| SEMA6C | [ENSG00000143434](https://www.ensembl.org/id/ENSG00000143434) | semaphorin 6C [Source:HGNC Symbol;Acc:HGNC:10740] | -2,03 |
| SERPINA1 | [ENSG00000197249](https://www.ensembl.org/id/ENSG00000197249) | serpin family A member 1 [Source:HGNC Symbol;Acc:HGNC:8941] | 4,40 |
| SERPINA5 | [ENSG00000188488](https://www.ensembl.org/id/ENSG00000188488) | serpin family A member 5 [Source:HGNC Symbol;Acc:HGNC:8723] | -2,17 |
| SERPINE3 | [ENSG00000253309](https://www.ensembl.org/id/ENSG00000253309) | serpin family E member 3 [Source:HGNC Symbol;Acc:HGNC:24774] | -5,13 |
| SERPINI2 | [ENSG00000114204](https://www.ensembl.org/id/ENSG00000114204) | serpin family I member 2 [Source:HGNC Symbol;Acc:HGNC:8945] | -5,09 |
| SERTM1 | [ENSG00000180440](https://www.ensembl.org/id/ENSG00000180440) | serine rich and transmembrane domain containing 1 [Source:HGNC Symbol;Acc:HGNC:33792] | -5,04 |
| SEZ6L | [ENSG00000100095](https://www.ensembl.org/id/ENSG00000100095) | seizure related 6 homolog like [Source:HGNC Symbol;Acc:HGNC:10763] | -6,43 |
| SEZ6L2 | [ENSG00000174938](https://www.ensembl.org/id/ENSG00000174938) | seizure related 6 homolog like 2 [Source:HGNC Symbol;Acc:HGNC:30844] | -2,74 |
| SFRP1 | [ENSG00000104332](https://www.ensembl.org/id/ENSG00000104332) | secreted frizzled related protein 1 [Source:HGNC Symbol;Acc:HGNC:10776] | -1,69 |
| SFRP2 | [ENSG00000145423](https://www.ensembl.org/id/ENSG00000145423) | secreted frizzled related protein 2 [Source:HGNC Symbol;Acc:HGNC:10777] | -1,31 |
| SFRP5 | [ENSG00000120057](https://www.ensembl.org/id/ENSG00000120057) | secreted frizzled related protein 5 [Source:HGNC Symbol;Acc:HGNC:10779] | -2,84 |
| SFTPD | [ENSG00000133661](https://www.ensembl.org/id/ENSG00000133661) | surfactant protein D [Source:HGNC Symbol;Acc:HGNC:10803] | 1,74 |
| SGCG | [ENSG00000102683](https://www.ensembl.org/id/ENSG00000102683) | sarcoglycan gamma [Source:HGNC Symbol;Acc:HGNC:10809] | -2,83 |
| SGK2 | [ENSG00000101049](https://www.ensembl.org/id/ENSG00000101049) | serum/glucocorticoid regulated kinase 2 [Source:HGNC Symbol;Acc:HGNC:13900] | -1,89 |
| SH3GL3 | [ENSG00000140600](https://www.ensembl.org/id/ENSG00000140600) | SH3 domain containing GRB2 like 3, endophilin A3 [Source:HGNC Symbol;Acc:HGNC:10832] | -3,09 |
| SH3RF2 | [ENSG00000156463](https://www.ensembl.org/id/ENSG00000156463) | SH3 domain containing ring finger 2 [Source:HGNC Symbol;Acc:HGNC:26299] | -1,40 |
| SHBG | [ENSG00000129214](https://www.ensembl.org/id/ENSG00000129214) | sex hormone binding globulin [Source:HGNC Symbol;Acc:HGNC:10839] | -2,63 |
| SHISA4 | [ENSG00000198892](https://www.ensembl.org/id/ENSG00000198892) | shisa family member 4 [Source:HGNC Symbol;Acc:HGNC:27139] | -1,39 |
| SHOC1 | [ENSG00000165181](https://www.ensembl.org/id/ENSG00000165181) | shortage in chiasmata 1 [Source:HGNC Symbol;Acc:HGNC:26535] | 4,77 |
| SHTN1 | [ENSG00000187164](https://www.ensembl.org/id/ENSG00000187164) | shootin 1 [Source:HGNC Symbol;Acc:HGNC:29319] | -1,53 |
| SIGLEC10 | [ENSG00000142512](https://www.ensembl.org/id/ENSG00000142512) | sialic acid binding Ig like lectin 10 [Source:HGNC Symbol;Acc:HGNC:15620] | 5,00 |
| SIGLEC5_1 | [ENSG00000268500](https://www.ensembl.org/id/ENSG00000268500) | sialic acid binding Ig like lectin 5 [Source:HGNC Symbol;Acc:HGNC:10874] | 3,15 |
| SIGLEC7 | [ENSG00000168995](https://www.ensembl.org/id/ENSG00000168995) | sialic acid binding Ig like lectin 7 [Source:HGNC Symbol;Acc:HGNC:10876] | 6,25 |
| SILC1 | [ENSG00000232044](https://www.ensembl.org/id/ENSG00000232044) | sciatic injury induced lincRNA upregulator of SOX11 [Source:HGNC Symbol;Acc:HGNC:26403] | -5,60 |
| SIM1 | [ENSG00000112246](https://www.ensembl.org/id/ENSG00000112246) | SIM bHLH transcription factor 1 [Source:HGNC Symbol;Acc:HGNC:10882] | -3,74 |
| SIRPB1 | [ENSG00000101307](https://www.ensembl.org/id/ENSG00000101307) | signal regulatory protein beta 1 [Source:HGNC Symbol;Acc:HGNC:15928] | 4,68 |
| SIX1 | [ENSG00000126778](https://www.ensembl.org/id/ENSG00000126778) | SIX homeobox 1 [Source:HGNC Symbol;Acc:HGNC:10887] | -2,17 |
| SIX4 | [ENSG00000100625](https://www.ensembl.org/id/ENSG00000100625) | SIX homeobox 4 [Source:HGNC Symbol;Acc:HGNC:10890] | -2,01 |
| SLA | [ENSG00000155926](https://www.ensembl.org/id/ENSG00000155926) | Src like adaptor [Source:HGNC Symbol;Acc:HGNC:10902] | 2,21 |
| SLA2 | [ENSG00000101082](https://www.ensembl.org/id/ENSG00000101082) | Src like adaptor 2 [Source:HGNC Symbol;Acc:HGNC:17329] | 2,71 |
| SLC11A1 | [ENSG00000018280](https://www.ensembl.org/id/ENSG00000018280) | solute carrier family 11 member 1 [Source:HGNC Symbol;Acc:HGNC:10907] | 4,02 |
| SLC16A10 | [ENSG00000112394](https://www.ensembl.org/id/ENSG00000112394) | solute carrier family 16 member 10 [Source:HGNC Symbol;Acc:HGNC:17027] | -2,56 |
| SLC16A3 | [ENSG00000141526](https://www.ensembl.org/id/ENSG00000141526) | solute carrier family 16 member 3 [Source:HGNC Symbol;Acc:HGNC:10924] | 1,76 |
| SLC18A2-AS1 | [ENSG00000225936](https://www.ensembl.org/id/ENSG00000225936) | SLC18A2 antisense RNA 1 [Source:HGNC Symbol;Acc:HGNC:55843] | 5,95 |
| SLC1A3 | [ENSG00000079215](https://www.ensembl.org/id/ENSG00000079215) | solute carrier family 1 member 3 [Source:HGNC Symbol;Acc:HGNC:10941] | -1,73 |
| SLC24A4 | [ENSG00000140090](https://www.ensembl.org/id/ENSG00000140090) | solute carrier family 24 member 4 [Source:HGNC Symbol;Acc:HGNC:10978] | -2,04 |
| SLC25A10 | [ENSG00000183048](https://www.ensembl.org/id/ENSG00000183048) | solute carrier family 25 member 10 [Source:HGNC Symbol;Acc:HGNC:10980] | -2,06 |
| SLC25A34 | [ENSG00000162461](https://www.ensembl.org/id/ENSG00000162461) | solute carrier family 25 member 34 [Source:HGNC Symbol;Acc:HGNC:27653] | -1,42 |
| SLC26A9 | [ENSG00000174502](https://www.ensembl.org/id/ENSG00000174502) | solute carrier family 26 member 9 [Source:HGNC Symbol;Acc:HGNC:14469] | -2,80 |
| SLC28A3 | [ENSG00000197506](https://www.ensembl.org/id/ENSG00000197506) | solute carrier family 28 member 3 [Source:HGNC Symbol;Acc:HGNC:16484] | -4,80 |
| SLC29A2 | [ENSG00000174669](https://www.ensembl.org/id/ENSG00000174669) | solute carrier family 29 member 2 [Source:HGNC Symbol;Acc:HGNC:11004] | -2,51 |
| SLC29A4 | [ENSG00000164638](https://www.ensembl.org/id/ENSG00000164638) | solute carrier family 29 member 4 [Source:HGNC Symbol;Acc:HGNC:23097] | -2,87 |
| SLC2A5 | [ENSG00000142583](https://www.ensembl.org/id/ENSG00000142583) | solute carrier family 2 member 5 [Source:HGNC Symbol;Acc:HGNC:11010] | -3,55 |
| SLC36A2 | [ENSG00000186335](https://www.ensembl.org/id/ENSG00000186335) | solute carrier family 36 member 2 [Source:HGNC Symbol;Acc:HGNC:18762] | -8,24 |
| SLC36A3 | [ENSG00000186334](https://www.ensembl.org/id/ENSG00000186334) | solute carrier family 36 member 3 [Source:HGNC Symbol;Acc:HGNC:19659] | -4,80 |
| SLC38A1 | [ENSG00000111371](https://www.ensembl.org/id/ENSG00000111371) | solute carrier family 38 member 1 [Source:HGNC Symbol;Acc:HGNC:13447] | 1,28 |
| SLC39A8 | [ENSG00000138821](https://www.ensembl.org/id/ENSG00000138821) | solute carrier family 39 member 8 [Source:HGNC Symbol;Acc:HGNC:20862] | -1,63 |
| SLC45A3 | [ENSG00000158715](https://www.ensembl.org/id/ENSG00000158715) | solute carrier family 45 member 3 [Source:HGNC Symbol;Acc:HGNC:8642] | -2,09 |
| SLC4A1 | [ENSG00000004939](https://www.ensembl.org/id/ENSG00000004939) | solute carrier family 4 member 1 (Diego blood group) [Source:HGNC Symbol;Acc:HGNC:11027] | 4,27 |
| SLC4A11 | [ENSG00000088836](https://www.ensembl.org/id/ENSG00000088836) | solute carrier family 4 member 11 [Source:HGNC Symbol;Acc:HGNC:16438] | -3,22 |
| SLC6A1 | [ENSG00000157103](https://www.ensembl.org/id/ENSG00000157103) | solute carrier family 6 member 1 [Source:HGNC Symbol;Acc:HGNC:11042] | -1,78 |
| SLC6A10P | [ENSG00000290985](https://www.ensembl.org/id/ENSG00000290985) | solute carrier family 6 member 10, pseudogene [Source:NCBI gene (formerly Entrezgene);Acc:386757] | -7,09 |
| SLC6A13 | [ENSG00000010379](https://www.ensembl.org/id/ENSG00000010379) | solute carrier family 6 member 13 [Source:HGNC Symbol;Acc:HGNC:11046] | -3,09 |
| SLC6A14 | [ENSG00000268104](https://www.ensembl.org/id/ENSG00000268104) | solute carrier family 6 member 14 [Source:HGNC Symbol;Acc:HGNC:11047] | -5,44 |
| SLC6A17 | [ENSG00000197106](https://www.ensembl.org/id/ENSG00000197106) | solute carrier family 6 member 17 [Source:HGNC Symbol;Acc:HGNC:31399] | 2,44 |
| SLC8A3 | [ENSG00000100678](https://www.ensembl.org/id/ENSG00000100678) | solute carrier family 8 member A3 [Source:HGNC Symbol;Acc:HGNC:11070] | -5,72 |
| SLCO6A1 | [ENSG00000205359](https://www.ensembl.org/id/ENSG00000205359) | solute carrier organic anion transporter family member 6A1 [Source:HGNC Symbol;Acc:HGNC:23613] | -5,22 |
| SLFN14 | [ENSG00000236320](https://www.ensembl.org/id/ENSG00000236320) | schlafen family member 14 [Source:HGNC Symbol;Acc:HGNC:32689] | 5,78 |
| SLN | [ENSG00000170290](https://www.ensembl.org/id/ENSG00000170290) | sarcolipin [Source:HGNC Symbol;Acc:HGNC:11089] | -7,41 |
| SLPI | [ENSG00000124107](https://www.ensembl.org/id/ENSG00000124107) | secretory leukocyte peptidase inhibitor [Source:HGNC Symbol;Acc:HGNC:11092] | -1,93 |
| SMCO1 | [ENSG00000214097](https://www.ensembl.org/id/ENSG00000214097) | single-pass membrane protein with coiled-coil domains 1 [Source:HGNC Symbol;Acc:HGNC:27407] | -11,24 |
| SMCO2 | [ENSG00000165935](https://www.ensembl.org/id/ENSG00000165935) | single-pass membrane protein with coiled-coil domains 2 [Source:HGNC Symbol;Acc:HGNC:34448] | -5,69 |
| SMOC1 | [ENSG00000198732](https://www.ensembl.org/id/ENSG00000198732) | SPARC related modular calcium binding 1 [Source:HGNC Symbol;Acc:HGNC:20318] | 1,12 |
| SMPD3 | [ENSG00000103056](https://www.ensembl.org/id/ENSG00000103056) | sphingomyelin phosphodiesterase 3 [Source:HGNC Symbol;Acc:HGNC:14240] | -2,16 |
| SMPX | [ENSG00000091482](https://www.ensembl.org/id/ENSG00000091482) | small muscle protein X-linked [Source:HGNC Symbol;Acc:HGNC:11122] | -8,29 |
| SMTNL1 | [ENSG00000214872](https://www.ensembl.org/id/ENSG00000214872) | smoothelin like 1 [Source:HGNC Symbol;Acc:HGNC:32394] | -8,99 |
| SMTNL2 | [ENSG00000188176](https://www.ensembl.org/id/ENSG00000188176) | smoothelin like 2 [Source:HGNC Symbol;Acc:HGNC:24764] | -6,11 |
| SMYD1 | [ENSG00000115593](https://www.ensembl.org/id/ENSG00000115593) | SET and MYND domain containing 1 [Source:HGNC Symbol;Acc:HGNC:20986] | -8,55 |
| SNCA | [ENSG00000145335](https://www.ensembl.org/id/ENSG00000145335) | synuclein alpha [Source:HGNC Symbol;Acc:HGNC:11138] | 1,85 |
| SNCG | [ENSG00000173267](https://www.ensembl.org/id/ENSG00000173267) | synuclein gamma [Source:HGNC Symbol;Acc:HGNC:11141] | 1,48 |
| SNED1 | [ENSG00000162804](https://www.ensembl.org/id/ENSG00000162804) | sushi, nidogen and EGF like domains 1 [Source:HGNC Symbol;Acc:HGNC:24696] | -0,86 |
| SNHG7 | [ENSG00000233016](https://www.ensembl.org/id/ENSG00000233016) | small nucleolar RNA host gene 7 [Source:HGNC Symbol;Acc:HGNC:28254] | -1,04 |
| SORD2P | [ENSG00000290387](https://www.ensembl.org/id/ENSG00000290387) | sorbitol dehydrogenase 2, pseudogene [Source:NCBI gene (formerly Entrezgene);Acc:653381] | 5,55 |
| SOX4 | [ENSG00000124766](https://www.ensembl.org/id/ENSG00000124766) | SRY-box transcription factor 4 [Source:HGNC Symbol;Acc:HGNC:11200] | -0,93 |
| SOX8 | [ENSG00000005513](https://www.ensembl.org/id/ENSG00000005513) | SRY-box transcription factor 8 [Source:HGNC Symbol;Acc:HGNC:11203] | -1,55 |
| SOX9-AS1 | [ENSG00000234899](https://www.ensembl.org/id/ENSG00000234899) | SOX9 antisense RNA 1 [Source:HGNC Symbol;Acc:HGNC:49321] | -3,87 |
| SP6 | [ENSG00000189120](https://www.ensembl.org/id/ENSG00000189120) | Sp6 transcription factor [Source:HGNC Symbol;Acc:HGNC:14530] | -2,83 |
| SPATA41 | [ENSG00000189419](https://www.ensembl.org/id/ENSG00000189419) | spermatogenesis associated 41 [Source:HGNC Symbol;Acc:HGNC:48613] | -1,85 |
| SPATA46 | [ENSG00000171722](https://www.ensembl.org/id/ENSG00000171722) | spermatogenesis associated 46 [Source:HGNC Symbol;Acc:HGNC:27648] | 4,96 |
| SPATC1L | [ENSG00000160284](https://www.ensembl.org/id/ENSG00000160284) | spermatogenesis and centriole associated 1 like [Source:HGNC Symbol;Acc:HGNC:1298] | -1,68 |
| SPINT2 | [ENSG00000167642](https://www.ensembl.org/id/ENSG00000167642) | serine peptidase inhibitor, Kunitz type 2 [Source:HGNC Symbol;Acc:HGNC:11247] | -2,34 |
| SPIRE2 | [ENSG00000204991](https://www.ensembl.org/id/ENSG00000204991) | spire type actin nucleation factor 2 [Source:HGNC Symbol;Acc:HGNC:30623] | -2,50 |
| SPN | [ENSG00000197471](https://www.ensembl.org/id/ENSG00000197471) | sialophorin [Source:HGNC Symbol;Acc:HGNC:11249] | 2,42 |
| SPON1 | [ENSG00000262655](https://www.ensembl.org/id/ENSG00000262655) | spondin 1 [Source:HGNC Symbol;Acc:HGNC:11252] | -1,75 |
| SPRR2F | [ENSG00000244094](https://www.ensembl.org/id/ENSG00000244094) | small proline rich protein 2F [Source:HGNC Symbol;Acc:HGNC:11266] | -3,60 |
| SPTB | [ENSG00000070182](https://www.ensembl.org/id/ENSG00000070182) | spectrin beta, erythrocytic [Source:HGNC Symbol;Acc:HGNC:11274] | -2,09 |
| SPX | [ENSG00000134548](https://www.ensembl.org/id/ENSG00000134548) | spexin hormone [Source:HGNC Symbol;Acc:HGNC:28139] | -3,42 |
| SRGN | [ENSG00000122862](https://www.ensembl.org/id/ENSG00000122862) | serglycin [Source:HGNC Symbol;Acc:HGNC:9361] | 1,85 |
| SRL | [ENSG00000185739](https://www.ensembl.org/id/ENSG00000185739) | sarcalumenin [Source:HGNC Symbol;Acc:HGNC:11295] | -2,26 |
| SSTR2 | [ENSG00000180616](https://www.ensembl.org/id/ENSG00000180616) | somatostatin receptor 2 [Source:HGNC Symbol;Acc:HGNC:11331] | 1,81 |
| ST3GAL3-AS1 | [ENSG00000229444](https://www.ensembl.org/id/ENSG00000229444) | ST3GAL3 antisense RNA 1 [Source:HGNC Symbol;Acc:HGNC:40529] | -10,50 |
| ST8SIA2 | [ENSG00000140557](https://www.ensembl.org/id/ENSG00000140557) | ST8 alpha-N-acetyl-neuraminide alpha-2,8-sialyltransferase 2 [Source:HGNC Symbol;Acc:HGNC:10870] | -4,09 |
| STAC3 | [ENSG00000185482](https://www.ensembl.org/id/ENSG00000185482) | SH3 and cysteine rich domain 3 [Source:HGNC Symbol;Acc:HGNC:28423] | -6,58 |
| STARD4 | [ENSG00000164211](https://www.ensembl.org/id/ENSG00000164211) | StAR related lipid transfer domain containing 4 [Source:HGNC Symbol;Acc:HGNC:18058] | 1,24 |
| STC1 | [ENSG00000159167](https://www.ensembl.org/id/ENSG00000159167) | stanniocalcin 1 [Source:HGNC Symbol;Acc:HGNC:11373] | -1,45 |
| STK32B | [ENSG00000152953](https://www.ensembl.org/id/ENSG00000152953) | serine/threonine kinase 32B [Source:HGNC Symbol;Acc:HGNC:14217] | -1,96 |
| STK33 | [ENSG00000130413](https://www.ensembl.org/id/ENSG00000130413) | serine/threonine kinase 33 [Source:HGNC Symbol;Acc:HGNC:14568] | -1,81 |
| STRIP2 | [ENSG00000128578](https://www.ensembl.org/id/ENSG00000128578) | striatin interacting protein 2 [Source:HGNC Symbol;Acc:HGNC:22209] | -4,02 |
| STRIT1 | [ENSG00000240045](https://www.ensembl.org/id/ENSG00000240045) | small transmembrane regulator of ion transport 1 [Source:HGNC Symbol;Acc:HGNC:52297] | -8,97 |
| STUM | [ENSG00000203685](https://www.ensembl.org/id/ENSG00000203685) | stum, mechanosensory transduction mediator homolog [Source:HGNC Symbol;Acc:HGNC:30491] | 1,62 |
| STYXL2 | [ENSG00000198842](https://www.ensembl.org/id/ENSG00000198842) | serine/threonine/tyrosine interacting like 2 [Source:HGNC Symbol;Acc:HGNC:25034] | -4,06 |
| SULT1A4 | [ENSG00000213648](https://www.ensembl.org/id/ENSG00000213648) | sulfotransferase family 1A member 4 [Source:HGNC Symbol;Acc:HGNC:30004] | -2,96 |
| SUSD2 | [ENSG00000099994](https://www.ensembl.org/id/ENSG00000099994) | sushi domain containing 2 [Source:HGNC Symbol;Acc:HGNC:30667] | 1,66 |
| SUSD4 | [ENSG00000143502](https://www.ensembl.org/id/ENSG00000143502) | sushi domain containing 4 [Source:HGNC Symbol;Acc:HGNC:25470] | -1,76 |
| SVOP | [ENSG00000166111](https://www.ensembl.org/id/ENSG00000166111) | SV2 related protein [Source:HGNC Symbol;Acc:HGNC:25417] | -6,10 |
| SYNPO2L | [ENSG00000166317](https://www.ensembl.org/id/ENSG00000166317) | synaptopodin 2 like [Source:HGNC Symbol;Acc:HGNC:23532] | -6,06 |
| SYPL2 | [ENSG00000143028](https://www.ensembl.org/id/ENSG00000143028) | synaptophysin like 2 [Source:HGNC Symbol;Acc:HGNC:27638] | -1,68 |
| SYT4 | [ENSG00000132872](https://www.ensembl.org/id/ENSG00000132872) | synaptotagmin 4 [Source:HGNC Symbol;Acc:HGNC:11512] | -4,82 |
| SYTL2 | [ENSG00000137501](https://www.ensembl.org/id/ENSG00000137501) | synaptotagmin like 2 [Source:HGNC Symbol;Acc:HGNC:15585] | 1,28 |
| TAFA5 | [ENSG00000219438](https://www.ensembl.org/id/ENSG00000219438) | TAFA chemokine like family member 5 [Source:HGNC Symbol;Acc:HGNC:21592] | -2,52 |
| TAL2 | [ENSG00000186051](https://www.ensembl.org/id/ENSG00000186051) | TAL bHLH transcription factor 2 [Source:HGNC Symbol;Acc:HGNC:11557] | -4,35 |
| TASL | [ENSG00000120280](https://www.ensembl.org/id/ENSG00000120280) | TLR adaptor interacting with endolysosomal SLC15A4 [Source:HGNC Symbol;Acc:HGNC:25667] | 5,13 |
| TBC1D3D | [ENSG00000274419](https://www.ensembl.org/id/ENSG00000274419) | TBC1 domain family member 3D [Source:HGNC Symbol;Acc:HGNC:28944] | -8,24 |
| TBC1D3K | [ENSG00000273513](https://www.ensembl.org/id/ENSG00000273513) | TBC1 domain family member 3K [Source:HGNC Symbol;Acc:HGNC:51245] | 6,07 |
| TBX15 | [ENSG00000092607](https://www.ensembl.org/id/ENSG00000092607) | T-box transcription factor 15 [Source:HGNC Symbol;Acc:HGNC:11594] | -3,11 |
| TBX21 | [ENSG00000073861](https://www.ensembl.org/id/ENSG00000073861) | T-box transcription factor 21 [Source:HGNC Symbol;Acc:HGNC:11599] | 2,75 |
| TCAP | [ENSG00000173991](https://www.ensembl.org/id/ENSG00000173991) | titin-cap [Source:HGNC Symbol;Acc:HGNC:11610] | -7,96 |
| TCP11X2 | [ENSG00000215029](https://www.ensembl.org/id/ENSG00000215029) | t-complex 11 family, X-linked 2 [Source:HGNC Symbol;Acc:HGNC:48335] | -4,98 |
| TECRL | [ENSG00000205678](https://www.ensembl.org/id/ENSG00000205678) | trans-2,3-enoyl-CoA reductase like [Source:HGNC Symbol;Acc:HGNC:27365] | -11,04 |
| TENM3-AS1 | [ENSG00000177822](https://www.ensembl.org/id/ENSG00000177822) | TENM3 antisense RNA 1 [Source:HGNC Symbol;Acc:HGNC:28076] | -2,75 |
| TERLR1 | [ENSG00000249201](https://www.ensembl.org/id/ENSG00000249201) | TERT regulating lncRNA 1 [Source:HGNC Symbol;Acc:HGNC:52952] | -3,41 |
| TESMIN | [ENSG00000132749](https://www.ensembl.org/id/ENSG00000132749) | testis expressed metallothionein like protein [Source:HGNC Symbol;Acc:HGNC:7446] | -2,75 |
| TEX45 | [ENSG00000198723](https://www.ensembl.org/id/ENSG00000198723) | testis expressed 45 [Source:HGNC Symbol;Acc:HGNC:24745] | 4,89 |
| TF | [ENSG00000091513](https://www.ensembl.org/id/ENSG00000091513) | transferrin [Source:HGNC Symbol;Acc:HGNC:11740] | -1,75 |
| TFAP2A-AS2 | [ENSG00000285278](https://www.ensembl.org/id/ENSG00000285278) | TFAP2A antisense RNA 2 [Source:NCBI gene (formerly Entrezgene);Acc:109729173] | -2,88 |
| TFPI2 | [ENSG00000105825](https://www.ensembl.org/id/ENSG00000105825) | tissue factor pathway inhibitor 2 [Source:HGNC Symbol;Acc:HGNC:11761] | -1,43 |
| TGFBI | [ENSG00000120708](https://www.ensembl.org/id/ENSG00000120708) | transforming growth factor beta induced [Source:HGNC Symbol;Acc:HGNC:11771] | 1,52 |
| TGM1 | [ENSG00000092295](https://www.ensembl.org/id/ENSG00000092295) | transglutaminase 1 [Source:HGNC Symbol;Acc:HGNC:11777] | -2,34 |
| THRSP | [ENSG00000151365](https://www.ensembl.org/id/ENSG00000151365) | thyroid hormone responsive [Source:HGNC Symbol;Acc:HGNC:11800] | -2,27 |
| TIGIT | [ENSG00000181847](https://www.ensembl.org/id/ENSG00000181847) | T cell immunoreceptor with Ig and ITIM domains [Source:HGNC Symbol;Acc:HGNC:26838] | 3,05 |
| TLCD3B | [ENSG00000149926](https://www.ensembl.org/id/ENSG00000149926) | TLC domain containing 3B [Source:HGNC Symbol;Acc:HGNC:25295] | -5,30 |
| TLE1-DT | [ENSG00000233926](https://www.ensembl.org/id/ENSG00000233926) | TLE1 divergent transcript [Source:HGNC Symbol;Acc:HGNC:55701] | -3,15 |
| TLL2 | [ENSG00000095587](https://www.ensembl.org/id/ENSG00000095587) | tolloid like 2 [Source:HGNC Symbol;Acc:HGNC:11844] | -3,80 |
| TLN2 | [ENSG00000171914](https://www.ensembl.org/id/ENSG00000171914) | talin 2 [Source:HGNC Symbol;Acc:HGNC:15447] | -0,91 |
| TM6SF1 | [ENSG00000136404](https://www.ensembl.org/id/ENSG00000136404) | transmembrane 6 superfamily member 1 [Source:HGNC Symbol;Acc:HGNC:11860] | -1,96 |
| TM7SF2 | [ENSG00000149809](https://www.ensembl.org/id/ENSG00000149809) | transmembrane 7 superfamily member 2 [Source:HGNC Symbol;Acc:HGNC:11863] | -1,74 |
| TMC1 | [ENSG00000165091](https://www.ensembl.org/id/ENSG00000165091) | transmembrane channel like 1 [Source:HGNC Symbol;Acc:HGNC:16513] | -4,11 |
| TMEFF1 | [ENSG00000241697](https://www.ensembl.org/id/ENSG00000241697) | transmembrane protein with EGF like and two follistatin like domains 1 [Source:HGNC Symbol;Acc:HGNC:11866] | -6,34 |
| TMEM132B | [ENSG00000139364](https://www.ensembl.org/id/ENSG00000139364) | transmembrane protein 132B [Source:HGNC Symbol;Acc:HGNC:29397] | -2,35 |
| TMEM151A | [ENSG00000179292](https://www.ensembl.org/id/ENSG00000179292) | transmembrane protein 151A [Source:HGNC Symbol;Acc:HGNC:28497] | -3,82 |
| TMEM171 | [ENSG00000157111](https://www.ensembl.org/id/ENSG00000157111) | transmembrane protein 171 [Source:HGNC Symbol;Acc:HGNC:27031] | -2,77 |
| TMEM181 | [ENSG00000146433](https://www.ensembl.org/id/ENSG00000146433) | transmembrane protein 181 [Source:HGNC Symbol;Acc:HGNC:20958] | 1,04 |
| TMEM182 | [ENSG00000170417](https://www.ensembl.org/id/ENSG00000170417) | transmembrane protein 182 [Source:HGNC Symbol;Acc:HGNC:26391] | -2,44 |
| TMEM233 | [ENSG00000224982](https://www.ensembl.org/id/ENSG00000224982) | transmembrane protein 233 [Source:HGNC Symbol;Acc:HGNC:37219] | -3,33 |
| TMEM26 | [ENSG00000196932](https://www.ensembl.org/id/ENSG00000196932) | transmembrane protein 26 [Source:HGNC Symbol;Acc:HGNC:28550] | -3,04 |
| TMEM266 | [ENSG00000169758](https://www.ensembl.org/id/ENSG00000169758) | transmembrane protein 266 [Source:HGNC Symbol;Acc:HGNC:26763] | -2,73 |
| TMEM35A | [ENSG00000126950](https://www.ensembl.org/id/ENSG00000126950) | transmembrane protein 35A [Source:HGNC Symbol;Acc:HGNC:25864] | 1,21 |
| TMEM37 | [ENSG00000171227](https://www.ensembl.org/id/ENSG00000171227) | transmembrane protein 37 [Source:HGNC Symbol;Acc:HGNC:18216] | -1,87 |
| TMEM38A | [ENSG00000072954](https://www.ensembl.org/id/ENSG00000072954) | transmembrane protein 38A [Source:HGNC Symbol;Acc:HGNC:28462] | -2,90 |
| TMEM52 | [ENSG00000178821](https://www.ensembl.org/id/ENSG00000178821) | transmembrane protein 52 [Source:HGNC Symbol;Acc:HGNC:27916] | -5,47 |
| TMEM61 | [ENSG00000143001](https://www.ensembl.org/id/ENSG00000143001) | transmembrane protein 61 [Source:HGNC Symbol;Acc:HGNC:27296] | 2,28 |
| TMEM71 | [ENSG00000165071](https://www.ensembl.org/id/ENSG00000165071) | transmembrane protein 71 [Source:HGNC Symbol;Acc:HGNC:26572] | 2,11 |
| TMOD4 | [ENSG00000163157](https://www.ensembl.org/id/ENSG00000163157) | tropomodulin 4 [Source:HGNC Symbol;Acc:HGNC:11874] | -7,28 |
| TMPRSS3 | [ENSG00000160183](https://www.ensembl.org/id/ENSG00000160183) | transmembrane serine protease 3 [Source:HGNC Symbol;Acc:HGNC:11877] | -5,53 |
| TMSB15A | [ENSG00000158164](https://www.ensembl.org/id/ENSG00000158164) | thymosin beta 15A [Source:HGNC Symbol;Acc:HGNC:30744] | 1,97 |
| TNC | [ENSG00000041982](https://www.ensembl.org/id/ENSG00000041982) | tenascin C [Source:HGNC Symbol;Acc:HGNC:5318] | 2,12 |
| TNFRSF10C | [ENSG00000173535](https://www.ensembl.org/id/ENSG00000173535) | TNF receptor superfamily member 10c [Source:HGNC Symbol;Acc:HGNC:11906] | 2,53 |
| TNFRSF21 | [ENSG00000146072](https://www.ensembl.org/id/ENSG00000146072) | TNF receptor superfamily member 21 [Source:HGNC Symbol;Acc:HGNC:13469] | -2,06 |
| TNFSF14 | [ENSG00000125735](https://www.ensembl.org/id/ENSG00000125735) | TNF superfamily member 14 [Source:HGNC Symbol;Acc:HGNC:11930] | -2,01 |
| TNNC1 | [ENSG00000114854](https://www.ensembl.org/id/ENSG00000114854) | troponin C1, slow skeletal and cardiac type [Source:HGNC Symbol;Acc:HGNC:11943] | -7,23 |
| TNNC2 | [ENSG00000101470](https://www.ensembl.org/id/ENSG00000101470) | troponin C2, fast skeletal type [Source:HGNC Symbol;Acc:HGNC:11944] | -6,70 |
| TNNI1 | [ENSG00000159173](https://www.ensembl.org/id/ENSG00000159173) | troponin I1, slow skeletal type [Source:HGNC Symbol;Acc:HGNC:11945] | -11,84 |
| TNNI2 | [ENSG00000130598](https://www.ensembl.org/id/ENSG00000130598) | troponin I2, fast skeletal type [Source:HGNC Symbol;Acc:HGNC:11946] | -6,38 |
| TNNI3 | [ENSG00000129991](https://www.ensembl.org/id/ENSG00000129991) | troponin I3, cardiac type [Source:HGNC Symbol;Acc:HGNC:11947] | -8,55 |
| TNNT1 | [ENSG00000105048](https://www.ensembl.org/id/ENSG00000105048) | troponin T1, slow skeletal type [Source:HGNC Symbol;Acc:HGNC:11948] | -10,55 |
| TNNT2 | [ENSG00000118194](https://www.ensembl.org/id/ENSG00000118194) | troponin T2, cardiac type [Source:HGNC Symbol;Acc:HGNC:11949] | -3,75 |
| TNNT3 | [ENSG00000130595](https://www.ensembl.org/id/ENSG00000130595) | troponin T3, fast skeletal type [Source:HGNC Symbol;Acc:HGNC:11950] | -4,99 |
| TNP1 | [ENSG00000118245](https://www.ensembl.org/id/ENSG00000118245) | transition protein 1 [Source:HGNC Symbol;Acc:HGNC:11951] | 5,28 |
| TOB1 | [ENSG00000141232](https://www.ensembl.org/id/ENSG00000141232) | transducer of ERBB2, 1 [Source:HGNC Symbol;Acc:HGNC:11979] | 1,31 |
| TOX2 | [ENSG00000124191](https://www.ensembl.org/id/ENSG00000124191) | TOX high mobility group box family member 2 [Source:HGNC Symbol;Acc:HGNC:16095] | -2,82 |
| TP63 | [ENSG00000073282](https://www.ensembl.org/id/ENSG00000073282) | tumor protein p63 [Source:HGNC Symbol;Acc:HGNC:15979] | -2,37 |
| TPBG | [ENSG00000146242](https://www.ensembl.org/id/ENSG00000146242) | trophoblast glycoprotein [Source:HGNC Symbol;Acc:HGNC:12004] | -1,02 |
| TPH1 | [ENSG00000129167](https://www.ensembl.org/id/ENSG00000129167) | tryptophan hydroxylase 1 [Source:HGNC Symbol;Acc:HGNC:12008] | -3,31 |
| TPM3 | [ENSG00000143549](https://www.ensembl.org/id/ENSG00000143549) | tropomyosin 3 [Source:HGNC Symbol;Acc:HGNC:12012] | -3,40 |
| TPPP2 | [ENSG00000179636](https://www.ensembl.org/id/ENSG00000179636) | tubulin polymerization promoting protein family member 2 [Source:HGNC Symbol;Acc:HGNC:19293] | -3,79 |
| TPSAB1 | [ENSG00000172236](https://www.ensembl.org/id/ENSG00000172236) | tryptase alpha/beta 1 [Source:HGNC Symbol;Acc:HGNC:12019] | -2,08 |
| TPSB2 | [ENSG00000197253](https://www.ensembl.org/id/ENSG00000197253) | tryptase beta 2 [Source:HGNC Symbol;Acc:HGNC:14120] | -1,86 |
| TRDN | [ENSG00000186439](https://www.ensembl.org/id/ENSG00000186439) | triadin [Source:HGNC Symbol;Acc:HGNC:12261] | -4,07 |
| TREH | [ENSG00000118094](https://www.ensembl.org/id/ENSG00000118094) | trehalase [Source:HGNC Symbol;Acc:HGNC:12266] | -2,32 |
| TREM1 | [ENSG00000124731](https://www.ensembl.org/id/ENSG00000124731) | triggering receptor expressed on myeloid cells 1 [Source:HGNC Symbol;Acc:HGNC:17760] | 4,18 |
| TREML1 | [ENSG00000161911](https://www.ensembl.org/id/ENSG00000161911) | triggering receptor expressed on myeloid cells like 1 [Source:HGNC Symbol;Acc:HGNC:20434] | 6,32 |
| TREML2 | [ENSG00000112195](https://www.ensembl.org/id/ENSG00000112195) | triggering receptor expressed on myeloid cells like 2 [Source:HGNC Symbol;Acc:HGNC:21092] | 4,04 |
| TRIM29 | [ENSG00000137699](https://www.ensembl.org/id/ENSG00000137699) | tripartite motif containing 29 [Source:HGNC Symbol;Acc:HGNC:17274] | -3,13 |
| TRIM54 | [ENSG00000138100](https://www.ensembl.org/id/ENSG00000138100) | tripartite motif containing 54 [Source:HGNC Symbol;Acc:HGNC:16008] | -6,31 |
| TRIM55 | [ENSG00000147573](https://www.ensembl.org/id/ENSG00000147573) | tripartite motif containing 55 [Source:HGNC Symbol;Acc:HGNC:14215] | -3,90 |
| TRIM58 | [ENSG00000162722](https://www.ensembl.org/id/ENSG00000162722) | tripartite motif containing 58 [Source:HGNC Symbol;Acc:HGNC:24150] | 4,16 |
| TRIM63 | [ENSG00000158022](https://www.ensembl.org/id/ENSG00000158022) | tripartite motif containing 63 [Source:HGNC Symbol;Acc:HGNC:16007] | -10,95 |
| TRIM7 | [ENSG00000146054](https://www.ensembl.org/id/ENSG00000146054) | tripartite motif containing 7 [Source:HGNC Symbol;Acc:HGNC:16278] | -2,22 |
| TRIM7-AS1 | [ENSG00000250900](https://www.ensembl.org/id/ENSG00000250900) | TRIM7 antisense RNA 1 [Source:HGNC Symbol;Acc:HGNC:40764] | -3,27 |
| TRIM72 | [ENSG00000177238](https://www.ensembl.org/id/ENSG00000177238) | tripartite motif containing 72 [Source:HGNC Symbol;Acc:HGNC:32671] | -6,10 |
| TRMT9B | [ENSG00000250305](https://www.ensembl.org/id/ENSG00000250305) | tRNA methyltransferase 9B (putative) [Source:HGNC Symbol;Acc:HGNC:26725] | -1,63 |
| TRPV1 | [ENSG00000196689](https://www.ensembl.org/id/ENSG00000196689) | transient receptor potential cation channel subfamily V member 1 [Source:HGNC Symbol;Acc:HGNC:12716] | 1,55 |
| TSPAN12 | [ENSG00000106025](https://www.ensembl.org/id/ENSG00000106025) | tetraspanin 12 [Source:HGNC Symbol;Acc:HGNC:21641] | 1,02 |
| TSPEAR-AS1 | [ENSG00000235890](https://www.ensembl.org/id/ENSG00000235890) | TSPEAR antisense RNA 1 [Source:HGNC Symbol;Acc:HGNC:1271] | -2,52 |
| TSSK1B | [ENSG00000212122](https://www.ensembl.org/id/ENSG00000212122) | testis specific serine kinase 1B [Source:HGNC Symbol;Acc:HGNC:14968] | -5,76 |
| TTLL6 | [ENSG00000170703](https://www.ensembl.org/id/ENSG00000170703) | tubulin tyrosine ligase like 6 [Source:HGNC Symbol;Acc:HGNC:26664] | -3,51 |
| TTN | [ENSG00000155657](https://www.ensembl.org/id/ENSG00000155657) | titin [Source:HGNC Symbol;Acc:HGNC:12403] | -7,84 |
| TTN-AS1 | [ENSG00000237298](https://www.ensembl.org/id/ENSG00000237298) | TTN antisense RNA 1 [Source:HGNC Symbol;Acc:HGNC:44124] | -2,47 |
| TUBA3D | [ENSG00000075886](https://www.ensembl.org/id/ENSG00000075886) | tubulin alpha 3d [Source:HGNC Symbol;Acc:HGNC:24071] | -4,73 |
| TUBA8 | [ENSG00000183785](https://www.ensembl.org/id/ENSG00000183785) | tubulin alpha 8 [Source:HGNC Symbol;Acc:HGNC:12410] | -2,51 |
| TUBB4A | [ENSG00000104833](https://www.ensembl.org/id/ENSG00000104833) | tubulin beta 4A class IVa [Source:HGNC Symbol;Acc:HGNC:20774] | -2,96 |
| TVP23C-CDRT4 | [ENSG00000259024](https://www.ensembl.org/id/ENSG00000259024) | TVP23C-CDRT4 readthrough [Source:HGNC Symbol;Acc:HGNC:42961] | 2,29 |
| TWIST2 | [ENSG00000233608](https://www.ensembl.org/id/ENSG00000233608) | twist family bHLH transcription factor 2 [Source:HGNC Symbol;Acc:HGNC:20670] | -1,54 |
| TXLNB | [ENSG00000164440](https://www.ensembl.org/id/ENSG00000164440) | taxilin beta [Source:HGNC Symbol;Acc:HGNC:21617] | -6,21 |
| UBE2F-SCLY | [ENSG00000258984](https://www.ensembl.org/id/ENSG00000258984) | UBE2F-SCLY readthrough (NMD candidate) [Source:HGNC Symbol;Acc:HGNC:48339] | 3,27 |
| UBXN10 | [ENSG00000162543](https://www.ensembl.org/id/ENSG00000162543) | UBX domain protein 10 [Source:HGNC Symbol;Acc:HGNC:26354] | -2,57 |
| UCP2 | [ENSG00000175567](https://www.ensembl.org/id/ENSG00000175567) | uncoupling protein 2 [Source:HGNC Symbol;Acc:HGNC:12518] | -1,80 |
| UCP3 | [ENSG00000175564](https://www.ensembl.org/id/ENSG00000175564) | uncoupling protein 3 [Source:HGNC Symbol;Acc:HGNC:12519] | -4,00 |
| UGT3A1 | [ENSG00000145626](https://www.ensembl.org/id/ENSG00000145626) | UDP glycosyltransferase family 3 member A1 [Source:HGNC Symbol;Acc:HGNC:26625] | -4,17 |
| ULK4P1 | [ENSG00000291023](https://www.ensembl.org/id/ENSG00000291023) | ULK4 pseudogene 1 [Source:NCBI gene (formerly Entrezgene);Acc:89838] | -2,62 |
| UMODL1 | [ENSG00000177398](https://www.ensembl.org/id/ENSG00000177398) | uromodulin like 1 [Source:HGNC Symbol;Acc:HGNC:12560] | -3,65 |
| UNC45B | [ENSG00000141161](https://www.ensembl.org/id/ENSG00000141161) | unc-45 myosin chaperone B [Source:HGNC Symbol;Acc:HGNC:14304] | -6,72 |
| UPK1B | [ENSG00000114638](https://www.ensembl.org/id/ENSG00000114638) | uroplakin 1B [Source:HGNC Symbol;Acc:HGNC:12578] | -6,10 |
| UPK3A | [ENSG00000100373](https://www.ensembl.org/id/ENSG00000100373) | uroplakin 3A [Source:HGNC Symbol;Acc:HGNC:12580] | -2,23 |
| UPK3B | [ENSG00000243566](https://www.ensembl.org/id/ENSG00000243566) | uroplakin 3B [Source:HGNC Symbol;Acc:HGNC:21444] | -7,47 |
| UPK3BL2 | [ENSG00000284981](https://www.ensembl.org/id/ENSG00000284981) | uroplakin 3B like 2 [Source:HGNC Symbol;Acc:HGNC:53444] | -8,05 |
| UROC1 | [ENSG00000159650](https://www.ensembl.org/id/ENSG00000159650) | urocanate hydratase 1 [Source:HGNC Symbol;Acc:HGNC:26444] | -4,31 |
| USH1C | [ENSG00000006611](https://www.ensembl.org/id/ENSG00000006611) | USH1 protein network component harmonin [Source:HGNC Symbol;Acc:HGNC:12597] | 5,04 |
| USP13 | [ENSG00000058056](https://www.ensembl.org/id/ENSG00000058056) | ubiquitin specific peptidase 13 [Source:HGNC Symbol;Acc:HGNC:12611] | -1,87 |
| VCX3B | [ENSG00000205642](https://www.ensembl.org/id/ENSG00000205642) | variable charge X-linked 3B [Source:HGNC Symbol;Acc:HGNC:31838] | -3,49 |
| VGLL2 | [ENSG00000170162](https://www.ensembl.org/id/ENSG00000170162) | vestigial like family member 2 [Source:HGNC Symbol;Acc:HGNC:20232] | -10,01 |
| VIL1 | [ENSG00000127831](https://www.ensembl.org/id/ENSG00000127831) | villin 1 [Source:HGNC Symbol;Acc:HGNC:12690] | 7,44 |
| VMO1 | [ENSG00000182853](https://www.ensembl.org/id/ENSG00000182853) | vitelline membrane outer layer 1 homolog [Source:HGNC Symbol;Acc:HGNC:30387] | -2,33 |
| VNN2 | [ENSG00000112303](https://www.ensembl.org/id/ENSG00000112303) | vanin 2 [Source:HGNC Symbol;Acc:HGNC:12706] | 4,82 |
| VSIG10L2 | [ENSG00000283703](https://www.ensembl.org/id/ENSG00000283703) | V-set and immunoglobulin domain containing 10 like 2 [Source:HGNC Symbol;Acc:HGNC:27879] | -4,48 |
| VSIG8 | [ENSG00000243284](https://www.ensembl.org/id/ENSG00000243284) | V-set and immunoglobulin domain containing 8 [Source:HGNC Symbol;Acc:HGNC:32063] | -2,99 |
| WASHC1 | [ENSG00000181404](https://www.ensembl.org/id/ENSG00000181404) | WASH complex subunit 1 [Source:HGNC Symbol;Acc:HGNC:24361] | -1,01 |
| WDR62 | [ENSG00000075702](https://www.ensembl.org/id/ENSG00000075702) | WD repeat domain 62 [Source:HGNC Symbol;Acc:HGNC:24502] | -3,29 |
| WDR97 | [ENSG00000179698](https://www.ensembl.org/id/ENSG00000179698) | WD repeat domain 97 [Source:HGNC Symbol;Acc:HGNC:26959] | -1,52 |
| WFDC1 | [ENSG00000103175](https://www.ensembl.org/id/ENSG00000103175) | WAP four-disulfide core domain 1 [Source:HGNC Symbol;Acc:HGNC:15466] | 2,03 |
| WFIKKN1 | [ENSG00000127578](https://www.ensembl.org/id/ENSG00000127578) | WAP, follistatin/kazal, immunoglobulin, kunitz and netrin domain containing 1 [Source:HGNC Symbol;Acc:HGNC:30912] | -1,82 |
| WSCD2 | [ENSG00000075035](https://www.ensembl.org/id/ENSG00000075035) | WSC domain containing 2 [Source:HGNC Symbol;Acc:HGNC:29117] | 1,25 |
| WT1 | [ENSG00000184937](https://www.ensembl.org/id/ENSG00000184937) | WT1 transcription factor [Source:HGNC Symbol;Acc:HGNC:12796] | -9,91 |
| WT1-AS | [ENSG00000183242](https://www.ensembl.org/id/ENSG00000183242) | WT1 antisense RNA [Source:HGNC Symbol;Acc:HGNC:18135] | -4,04 |
| XIRP1 | [ENSG00000168334](https://www.ensembl.org/id/ENSG00000168334) | xin actin binding repeat containing 1 [Source:HGNC Symbol;Acc:HGNC:14301] | -3,98 |
| XIRP2 | [ENSG00000163092](https://www.ensembl.org/id/ENSG00000163092) | xin actin binding repeat containing 2 [Source:HGNC Symbol;Acc:HGNC:14303] | -9,46 |
| XK | [ENSG00000047597](https://www.ensembl.org/id/ENSG00000047597) | X-linked Kx blood group antigen, Kell and VPS13A binding protein [Source:HGNC Symbol;Acc:HGNC:12811] | 1,38 |
| YIF1B | [ENSG00000167645](https://www.ensembl.org/id/ENSG00000167645) | Yip1 interacting factor homolog B, membrane trafficking protein [Source:HGNC Symbol;Acc:HGNC:30511] | -1,05 |
| YIPF7 | [ENSG00000177752](https://www.ensembl.org/id/ENSG00000177752) | Yip1 domain family member 7 [Source:HGNC Symbol;Acc:HGNC:26825] | -4,66 |
| ZIC1 | [ENSG00000152977](https://www.ensembl.org/id/ENSG00000152977) | Zic family member 1 [Source:HGNC Symbol;Acc:HGNC:12872] | -6,23 |
| ZIC3 | [ENSG00000156925](https://www.ensembl.org/id/ENSG00000156925) | Zic family member 3 [Source:HGNC Symbol;Acc:HGNC:12874] | -5,10 |
| ZIC4 | [ENSG00000174963](https://www.ensembl.org/id/ENSG00000174963) | Zic family member 4 [Source:HGNC Symbol;Acc:HGNC:20393] | -4,30 |
| ZNF106 | [ENSG00000103994](https://www.ensembl.org/id/ENSG00000103994) | zinc finger protein 106 [Source:HGNC Symbol;Acc:HGNC:12886] | -2,03 |
| ZNF141 | [ENSG00000131127](https://www.ensembl.org/id/ENSG00000131127) | zinc finger protein 141 [Source:HGNC Symbol;Acc:HGNC:12926] | 1,61 |
| ZNF331 | [ENSG00000130844](https://www.ensembl.org/id/ENSG00000130844) | zinc finger protein 331 [Source:HGNC Symbol;Acc:HGNC:15489] | 2,05 |
| ZNF350-AS1 | [ENSG00000269235](https://www.ensembl.org/id/ENSG00000269235) | ZNF350 antisense RNA 1 [Source:HGNC Symbol;Acc:HGNC:48598] | 2,00 |
| ZNF385A | [ENSG00000161642](https://www.ensembl.org/id/ENSG00000161642) | zinc finger protein 385A [Source:HGNC Symbol;Acc:HGNC:17521] | -1,22 |
| ZNF556 | [ENSG00000172000](https://www.ensembl.org/id/ENSG00000172000) | zinc finger protein 556 [Source:HGNC Symbol;Acc:HGNC:25669] | -3,48 |
| ZNF774 | [ENSG00000196391](https://www.ensembl.org/id/ENSG00000196391) | zinc finger protein 774 [Source:HGNC Symbol;Acc:HGNC:33108] | 1,37 |
| ZNF804A | [ENSG00000170396](https://www.ensembl.org/id/ENSG00000170396) | zinc finger protein 804A [Source:HGNC Symbol;Acc:HGNC:21711] | 4,40 |
| ZNF98 | [ENSG00000197360](https://www.ensembl.org/id/ENSG00000197360) | zinc finger protein 98 [Source:HGNC Symbol;Acc:HGNC:13174] | 4,96 |
| ZSCAN23 | [ENSG00000187987](https://www.ensembl.org/id/ENSG00000187987) | zinc finger and SCAN domain containing 23 [Source:HGNC Symbol;Acc:HGNC:21193] | 3,53 |
