# Supplementary material for: Role of inflammatory signaling pathways involving the CD40–CD40L–TRAF cascade in diabetes and hypertension—insights from animal and human studies
Source: Basic Res Cardiol. 2024 Mar 30;119(4):1–18. doi: 10.1007/s00395-024-01045-1 (PMC11319409; doi:10.1007/s00395-024-01045-1)
Supplement: Supplementary file 4 — Supplementary file4 (DOCX 400 KB) [file 395_2024_1045_MOESM4_ESM.docx]

| **Suppl. Table S5. RNA-Seq data: 2928 different expressed genes (DEG, p < 0.05) for the comparison CHD + HT + T2DM vs. CHD + HT.** | | | |
| --- | --- | --- | --- |
| **Gene name** | **Identifier** | **Gene description** | **CHD + HT + T2DM vs. CHD + HD - Log fold change** |
| A1BG-AS1 | [ENSG00000268895](https://www.ensembl.org/id/ENSG00000268895) | A1BG antisense RNA 1 [Source:HGNC Symbol;Acc:HGNC:37133] | 1,48 |
| A3GALT2 | [ENSG00000184389](https://www.ensembl.org/id/ENSG00000184389) | alpha 1,3-galactosyltransferase 2 [Source:HGNC Symbol;Acc:HGNC:30005] | 4,78 |
| AAMDC | [ENSG00000087884](https://www.ensembl.org/id/ENSG00000087884) | adipogenesis associated Mth938 domain containing [Source:HGNC Symbol;Acc:HGNC:30205] | -1,61 |
| AARD | [ENSG00000205002](https://www.ensembl.org/id/ENSG00000205002) | alanine and arginine rich domain containing protein [Source:HGNC Symbol;Acc:HGNC:33842] | -5,61 |
| ABCB4 | [ENSG00000005471](https://www.ensembl.org/id/ENSG00000005471) | ATP binding cassette subfamily B member 4 [Source:HGNC Symbol;Acc:HGNC:45] | -1,81 |
| ABCB5 | [ENSG00000004846](https://www.ensembl.org/id/ENSG00000004846) | ATP binding cassette subfamily B member 5 [Source:HGNC Symbol;Acc:HGNC:46] | -2,70 |
| ABCB7 | [ENSG00000131269](https://www.ensembl.org/id/ENSG00000131269) | ATP binding cassette subfamily B member 7 [Source:HGNC Symbol;Acc:HGNC:48] | -1,00 |
| ABCC12 | [ENSG00000140798](https://www.ensembl.org/id/ENSG00000140798) | ATP binding cassette subfamily C member 12 [Source:HGNC Symbol;Acc:HGNC:14640] | -7,90 |
| ABCD2 | [ENSG00000173208](https://www.ensembl.org/id/ENSG00000173208) | ATP binding cassette subfamily D member 2 [Source:HGNC Symbol;Acc:HGNC:66] | 1,69 |
| ABCG1 | [ENSG00000160179](https://www.ensembl.org/id/ENSG00000160179) | ATP binding cassette subfamily G member 1 [Source:HGNC Symbol;Acc:HGNC:73] | -1,57 |
| ABCG8 | [ENSG00000143921](https://www.ensembl.org/id/ENSG00000143921) | ATP binding cassette subfamily G member 8 [Source:HGNC Symbol;Acc:HGNC:13887] | -3,51 |
| ABHD1 | [ENSG00000143994](https://www.ensembl.org/id/ENSG00000143994) | abhydrolase domain containing 1 [Source:HGNC Symbol;Acc:HGNC:17553] | -2,93 |
| ABHD16B | [ENSG00000183260](https://www.ensembl.org/id/ENSG00000183260) | abhydrolase domain containing 16B [Source:HGNC Symbol;Acc:HGNC:16128] | 5,31 |
| ABLIM1 | [ENSG00000099204](https://www.ensembl.org/id/ENSG00000099204) | actin binding LIM protein 1 [Source:HGNC Symbol;Acc:HGNC:78] | -2,18 |
| ABLIM2 | [ENSG00000163995](https://www.ensembl.org/id/ENSG00000163995) | actin binding LIM protein family member 2 [Source:HGNC Symbol;Acc:HGNC:19195] | -2,89 |
| ABLIM3 | [ENSG00000173210](https://www.ensembl.org/id/ENSG00000173210) | actin binding LIM protein family member 3 [Source:HGNC Symbol;Acc:HGNC:29132] | -1,47 |
| ABRA | [ENSG00000174429](https://www.ensembl.org/id/ENSG00000174429) | actin binding Rho activating protein [Source:HGNC Symbol;Acc:HGNC:30655] | -2,29 |
| ACAA2 | [ENSG00000167315](https://www.ensembl.org/id/ENSG00000167315) | acetyl-CoA acyltransferase 2 [Source:HGNC Symbol;Acc:HGNC:83] | -2,10 |
| ACAD8 | [ENSG00000151498](https://www.ensembl.org/id/ENSG00000151498) | acyl-CoA dehydrogenase family member 8 [Source:HGNC Symbol;Acc:HGNC:87] | -1,00 |
| ACADM | [ENSG00000117054](https://www.ensembl.org/id/ENSG00000117054) | acyl-CoA dehydrogenase medium chain [Source:HGNC Symbol;Acc:HGNC:89] | -2,34 |
| ACADS | [ENSG00000122971](https://www.ensembl.org/id/ENSG00000122971) | acyl-CoA dehydrogenase short chain [Source:HGNC Symbol;Acc:HGNC:90] | -2,22 |
| ACADSB | [ENSG00000196177](https://www.ensembl.org/id/ENSG00000196177) | acyl-CoA dehydrogenase short/branched chain [Source:HGNC Symbol;Acc:HGNC:91] | -1,76 |
| ACADVL | [ENSG00000072778](https://www.ensembl.org/id/ENSG00000072778) | acyl-CoA dehydrogenase very long chain [Source:HGNC Symbol;Acc:HGNC:92] | -1,95 |
| ACAT1 | [ENSG00000075239](https://www.ensembl.org/id/ENSG00000075239) | acetyl-CoA acetyltransferase 1 [Source:HGNC Symbol;Acc:HGNC:93] | -2,45 |
| ACBD5 | [ENSG00000107897](https://www.ensembl.org/id/ENSG00000107897) | acyl-CoA binding domain containing 5 [Source:HGNC Symbol;Acc:HGNC:23338] | -1,00 |
| ACHE | [ENSG00000087085](https://www.ensembl.org/id/ENSG00000087085) | acetylcholinesterase (Cartwright blood group) [Source:HGNC Symbol;Acc:HGNC:108] | -5,91 |
| ACKR3 | [ENSG00000144476](https://www.ensembl.org/id/ENSG00000144476) | atypical chemokine receptor 3 [Source:HGNC Symbol;Acc:HGNC:23692] | 1,97 |
| ACO2 | [ENSG00000100412](https://www.ensembl.org/id/ENSG00000100412) | aconitase 2 [Source:HGNC Symbol;Acc:HGNC:118] | -2,39 |
| ACOT11 | [ENSG00000162390](https://www.ensembl.org/id/ENSG00000162390) | acyl-CoA thioesterase 11 [Source:HGNC Symbol;Acc:HGNC:18156] | -1,71 |
| ACRBP | [ENSG00000111644](https://www.ensembl.org/id/ENSG00000111644) | acrosin binding protein [Source:HGNC Symbol;Acc:HGNC:17195] | -2,85 |
| ACSF2 | [ENSG00000167107](https://www.ensembl.org/id/ENSG00000167107) | acyl-CoA synthetase family member 2 [Source:HGNC Symbol;Acc:HGNC:26101] | -1,09 |
| ACSL1 | [ENSG00000151726](https://www.ensembl.org/id/ENSG00000151726) | acyl-CoA synthetase long chain family member 1 [Source:HGNC Symbol;Acc:HGNC:3569] | -2,15 |
| ACSL6 | [ENSG00000164398](https://www.ensembl.org/id/ENSG00000164398) | acyl-CoA synthetase long chain family member 6 [Source:HGNC Symbol;Acc:HGNC:16496] | -2,01 |
| ACSS1 | [ENSG00000154930](https://www.ensembl.org/id/ENSG00000154930) | acyl-CoA synthetase short chain family member 1 [Source:HGNC Symbol;Acc:HGNC:16091] | -1,79 |
| ACSS2 | [ENSG00000131069](https://www.ensembl.org/id/ENSG00000131069) | acyl-CoA synthetase short chain family member 2 [Source:HGNC Symbol;Acc:HGNC:15814] | -1,31 |
| ACTA1 | [ENSG00000143632](https://www.ensembl.org/id/ENSG00000143632) | actin alpha 1, skeletal muscle [Source:HGNC Symbol;Acc:HGNC:129] | -8,74 |
| ACTL7B | [ENSG00000148156](https://www.ensembl.org/id/ENSG00000148156) | actin like 7B [Source:HGNC Symbol;Acc:HGNC:162] | -4,71 |
| ACTN2 | [ENSG00000077522](https://www.ensembl.org/id/ENSG00000077522) | actinin alpha 2 [Source:HGNC Symbol;Acc:HGNC:164] | -5,83 |
| ACTN3 | [ENSG00000248746](https://www.ensembl.org/id/ENSG00000248746) | actinin alpha 3 [Source:HGNC Symbol;Acc:HGNC:165] | -10,19 |
| ACVR1C | [ENSG00000123612](https://www.ensembl.org/id/ENSG00000123612) | activin A receptor type 1C [Source:HGNC Symbol;Acc:HGNC:18123] | 2,02 |
| ACVR2B-AS1 | [ENSG00000229589](https://www.ensembl.org/id/ENSG00000229589) | ACVR2B antisense RNA 1 [Source:HGNC Symbol;Acc:HGNC:44161] | -2,59 |
| ACY1 | [ENSG00000243989](https://www.ensembl.org/id/ENSG00000243989) | aminoacylase 1 [Source:HGNC Symbol;Acc:HGNC:177] | -1,80 |
| ACY3 | [ENSG00000132744](https://www.ensembl.org/id/ENSG00000132744) | aminoacylase 3 [Source:HGNC Symbol;Acc:HGNC:24104] | -4,86 |
| ACYP2 | [ENSG00000170634](https://www.ensembl.org/id/ENSG00000170634) | acylphosphatase 2 [Source:HGNC Symbol;Acc:HGNC:180] | -2,64 |
| ADAM19 | [ENSG00000135074](https://www.ensembl.org/id/ENSG00000135074) | ADAM metallopeptidase domain 19 [Source:HGNC Symbol;Acc:HGNC:197] | 1,19 |
| ADAM20P1 | [ENSG00000290783](https://www.ensembl.org/id/ENSG00000290783) | ADAM metallopeptidase domain 20 pseudogene 1 [Source:HGNC Symbol;Acc:HGNC:20102] | 2,14 |
| ADAM23 | [ENSG00000114948](https://www.ensembl.org/id/ENSG00000114948) | ADAM metallopeptidase domain 23 [Source:HGNC Symbol;Acc:HGNC:202] | -2,44 |
| ADAM28 | [ENSG00000042980](https://www.ensembl.org/id/ENSG00000042980) | ADAM metallopeptidase domain 28 [Source:HGNC Symbol;Acc:HGNC:206] | -2,84 |
| ADAMTS15 | [ENSG00000166106](https://www.ensembl.org/id/ENSG00000166106) | ADAM metallopeptidase with thrombospondin type 1 motif 15 [Source:HGNC Symbol;Acc:HGNC:16305] | 2,40 |
| ADAMTS19 | [ENSG00000145808](https://www.ensembl.org/id/ENSG00000145808) | ADAM metallopeptidase with thrombospondin type 1 motif 19 [Source:HGNC Symbol;Acc:HGNC:17111] | -4,22 |
| ADAMTS4 | [ENSG00000158859](https://www.ensembl.org/id/ENSG00000158859) | ADAM metallopeptidase with thrombospondin type 1 motif 4 [Source:HGNC Symbol;Acc:HGNC:220] | 2,29 |
| ADAMTS9-AS1 | [ENSG00000241158](https://www.ensembl.org/id/ENSG00000241158) | ADAMTS9 antisense RNA 1 [Source:HGNC Symbol;Acc:HGNC:40625] | 1,67 |
| ADAMTSL5 | [ENSG00000185761](https://www.ensembl.org/id/ENSG00000185761) | ADAMTS like 5 [Source:HGNC Symbol;Acc:HGNC:27912] | -1,93 |
| ADAP2 | [ENSG00000184060](https://www.ensembl.org/id/ENSG00000184060) | ArfGAP with dual PH domains 2 [Source:HGNC Symbol;Acc:HGNC:16487] | -2,06 |
| ADCY2 | [ENSG00000078295](https://www.ensembl.org/id/ENSG00000078295) | adenylate cyclase 2 [Source:HGNC Symbol;Acc:HGNC:233] | -5,06 |
| ADCY3 | [ENSG00000138031](https://www.ensembl.org/id/ENSG00000138031) | adenylate cyclase 3 [Source:HGNC Symbol;Acc:HGNC:234] | 0,88 |
| ADGRF4 | [ENSG00000153294](https://www.ensembl.org/id/ENSG00000153294) | adhesion G protein-coupled receptor F4 [Source:HGNC Symbol;Acc:HGNC:19011] | -4,32 |
| ADGRL3 | [ENSG00000150471](https://www.ensembl.org/id/ENSG00000150471) | adhesion G protein-coupled receptor L3 [Source:HGNC Symbol;Acc:HGNC:20974] | 1,60 |
| ADH1C | [ENSG00000248144](https://www.ensembl.org/id/ENSG00000248144) | alcohol dehydrogenase 1C (class I), gamma polypeptide [Source:HGNC Symbol;Acc:HGNC:251] | -1,82 |
| ADHFE1 | [ENSG00000147576](https://www.ensembl.org/id/ENSG00000147576) | alcohol dehydrogenase iron containing 1 [Source:HGNC Symbol;Acc:HGNC:16354] | -1,61 |
| ADIRF-AS1 | [ENSG00000272734](https://www.ensembl.org/id/ENSG00000272734) | ADIRF antisense RNA 1 [Source:HGNC Symbol;Acc:HGNC:45127] | 0,99 |
| ADORA1 | [ENSG00000163485](https://www.ensembl.org/id/ENSG00000163485) | adenosine A1 receptor [Source:HGNC Symbol;Acc:HGNC:262] | -4,70 |
| ADPRHL1 | [ENSG00000153531](https://www.ensembl.org/id/ENSG00000153531) | ADP-ribosylhydrolase like 1 [Source:HGNC Symbol;Acc:HGNC:21303] | -4,44 |
| ADRA1B | [ENSG00000170214](https://www.ensembl.org/id/ENSG00000170214) | adrenoceptor alpha 1B [Source:HGNC Symbol;Acc:HGNC:278] | 1,46 |
| ADSL | [ENSG00000239900](https://www.ensembl.org/id/ENSG00000239900) | adenylosuccinate lyase [Source:HGNC Symbol;Acc:HGNC:291] | -1,98 |
| ADSS1 | [ENSG00000185100](https://www.ensembl.org/id/ENSG00000185100) | adenylosuccinate synthase 1 [Source:HGNC Symbol;Acc:HGNC:20093] | -3,38 |
| AFAP1L1 | [ENSG00000157510](https://www.ensembl.org/id/ENSG00000157510) | actin filament associated protein 1 like 1 [Source:HGNC Symbol;Acc:HGNC:26714] | -1,36 |
| AFF3 | [ENSG00000144218](https://www.ensembl.org/id/ENSG00000144218) | ALF transcription elongation factor 3 [Source:HGNC Symbol;Acc:HGNC:6473] | 1,40 |
| AFG3L2 | [ENSG00000141385](https://www.ensembl.org/id/ENSG00000141385) | AFG3 like matrix AAA peptidase subunit 2 [Source:HGNC Symbol;Acc:HGNC:315] | -1,82 |
| AGAP2 | [ENSG00000135439](https://www.ensembl.org/id/ENSG00000135439) | ArfGAP with GTPase domain, ankyrin repeat and PH domain 2 [Source:HGNC Symbol;Acc:HGNC:16921] | -2,42 |
| AGBL1 | [ENSG00000273540](https://www.ensembl.org/id/ENSG00000273540) | AGBL carboxypeptidase 1 [Source:HGNC Symbol;Acc:HGNC:26504] | -9,95 |
| AGK-DT | [ENSG00000261570](https://www.ensembl.org/id/ENSG00000261570) | AGK divergent transcript [Source:HGNC Symbol;Acc:HGNC:55356] | -3,48 |
| AGL | [ENSG00000162688](https://www.ensembl.org/id/ENSG00000162688) | amylo-alpha-1, 6-glucosidase, 4-alpha-glucanotransferase [Source:HGNC Symbol;Acc:HGNC:321] | -2,98 |
| AGMAT | [ENSG00000116771](https://www.ensembl.org/id/ENSG00000116771) | agmatinase [Source:HGNC Symbol;Acc:HGNC:18407] | -7,25 |
| AGPAT3 | [ENSG00000160216](https://www.ensembl.org/id/ENSG00000160216) | 1-acylglycerol-3-phosphate O-acyltransferase 3 [Source:HGNC Symbol;Acc:HGNC:326] | -1,12 |
| AGTR2 | [ENSG00000180772](https://www.ensembl.org/id/ENSG00000180772) | angiotensin II receptor type 2 [Source:HGNC Symbol;Acc:HGNC:338] | -4,00 |
| AHCY | [ENSG00000101444](https://www.ensembl.org/id/ENSG00000101444) | adenosylhomocysteinase [Source:HGNC Symbol;Acc:HGNC:343] | -0,82 |
| AHCYL2 | [ENSG00000158467](https://www.ensembl.org/id/ENSG00000158467) | adenosylhomocysteinase like 2 [Source:HGNC Symbol;Acc:HGNC:22204] | -1,61 |
| AHSP | [ENSG00000169877](https://www.ensembl.org/id/ENSG00000169877) | alpha hemoglobin stabilizing protein [Source:HGNC Symbol;Acc:HGNC:18075] | 5,10 |
| AIF1L | [ENSG00000126878](https://www.ensembl.org/id/ENSG00000126878) | allograft inflammatory factor 1 like [Source:HGNC Symbol;Acc:HGNC:28904] | 1,59 |
| AIFM1 | [ENSG00000156709](https://www.ensembl.org/id/ENSG00000156709) | apoptosis inducing factor mitochondria associated 1 [Source:HGNC Symbol;Acc:HGNC:8768] | -1,23 |
| AIMP2 | [ENSG00000106305](https://www.ensembl.org/id/ENSG00000106305) | aminoacyl tRNA synthetase complex interacting multifunctional protein 2 [Source:HGNC Symbol;Acc:HGNC:20609] | -1,84 |
| AJUBA | [ENSG00000129474](https://www.ensembl.org/id/ENSG00000129474) | ajuba LIM protein [Source:HGNC Symbol;Acc:HGNC:20250] | 1,12 |
| AK1 | [ENSG00000106992](https://www.ensembl.org/id/ENSG00000106992) | adenylate kinase 1 [Source:HGNC Symbol;Acc:HGNC:361] | -2,22 |
| AK3 | [ENSG00000147853](https://www.ensembl.org/id/ENSG00000147853) | adenylate kinase 3 [Source:HGNC Symbol;Acc:HGNC:17376] | -1,22 |
| AK4 | [ENSG00000162433](https://www.ensembl.org/id/ENSG00000162433) | adenylate kinase 4 [Source:HGNC Symbol;Acc:HGNC:363] | 1,05 |
| AK5 | [ENSG00000154027](https://www.ensembl.org/id/ENSG00000154027) | adenylate kinase 5 [Source:HGNC Symbol;Acc:HGNC:365] | 2,36 |
| AKAP12 | [ENSG00000131016](https://www.ensembl.org/id/ENSG00000131016) | A-kinase anchoring protein 12 [Source:HGNC Symbol;Acc:HGNC:370] | 1,50 |
| AKR1B1 | [ENSG00000085662](https://www.ensembl.org/id/ENSG00000085662) | aldo-keto reductase family 1 member B [Source:HGNC Symbol;Acc:HGNC:381] | -2,68 |
| AKR1B10 | [ENSG00000198074](https://www.ensembl.org/id/ENSG00000198074) | aldo-keto reductase family 1 member B10 [Source:HGNC Symbol;Acc:HGNC:382] | -2,78 |
| AKR1B15 | [ENSG00000227471](https://www.ensembl.org/id/ENSG00000227471) | aldo-keto reductase family 1 member B15 [Source:HGNC Symbol;Acc:HGNC:37281] | -5,52 |
| ALAS1 | [ENSG00000023330](https://www.ensembl.org/id/ENSG00000023330) | 5'-aminolevulinate synthase 1 [Source:HGNC Symbol;Acc:HGNC:396] | -1,21 |
| ALAS2 | [ENSG00000158578](https://www.ensembl.org/id/ENSG00000158578) | 5'-aminolevulinate synthase 2 [Source:HGNC Symbol;Acc:HGNC:397] | 6,86 |
| ALDH1B1 | [ENSG00000137124](https://www.ensembl.org/id/ENSG00000137124) | aldehyde dehydrogenase 1 family member B1 [Source:HGNC Symbol;Acc:HGNC:407] | 1,59 |
| ALDH1L1 | [ENSG00000144908](https://www.ensembl.org/id/ENSG00000144908) | aldehyde dehydrogenase 1 family member L1 [Source:HGNC Symbol;Acc:HGNC:3978] | -2,05 |
| ALDH1L1-AS2 | [ENSG00000246022](https://www.ensembl.org/id/ENSG00000246022) | ALDH1L1 antisense RNA 2 [Source:HGNC Symbol;Acc:HGNC:42446] | -3,90 |
| ALDH4A1 | [ENSG00000159423](https://www.ensembl.org/id/ENSG00000159423) | aldehyde dehydrogenase 4 family member A1 [Source:HGNC Symbol;Acc:HGNC:406] | -1,26 |
| ALDH5A1 | [ENSG00000112294](https://www.ensembl.org/id/ENSG00000112294) | aldehyde dehydrogenase 5 family member A1 [Source:HGNC Symbol;Acc:HGNC:408] | -3,14 |
| ALDH6A1 | [ENSG00000119711](https://www.ensembl.org/id/ENSG00000119711) | aldehyde dehydrogenase 6 family member A1 [Source:HGNC Symbol;Acc:HGNC:7179] | -1,79 |
| ALDH9A1 | [ENSG00000143149](https://www.ensembl.org/id/ENSG00000143149) | aldehyde dehydrogenase 9 family member A1 [Source:HGNC Symbol;Acc:HGNC:412] | -0,95 |
| ALDOA | [ENSG00000149925](https://www.ensembl.org/id/ENSG00000149925) | aldolase, fructose-bisphosphate A [Source:HGNC Symbol;Acc:HGNC:414] | -2,39 |
| ALKAL2 | [ENSG00000189292](https://www.ensembl.org/id/ENSG00000189292) | ALK and LTK ligand 2 [Source:HGNC Symbol;Acc:HGNC:27683] | 1,77 |
| ALKBH5 | [ENSG00000091542](https://www.ensembl.org/id/ENSG00000091542) | alkB homolog 5, RNA demethylase [Source:HGNC Symbol;Acc:HGNC:25996] | -1,13 |
| ALPK2 | [ENSG00000198796](https://www.ensembl.org/id/ENSG00000198796) | alpha kinase 2 [Source:HGNC Symbol;Acc:HGNC:20565] | -9,55 |
| ALPK3 | [ENSG00000136383](https://www.ensembl.org/id/ENSG00000136383) | alpha kinase 3 [Source:HGNC Symbol;Acc:HGNC:17574] | -1,92 |
| ALX1 | [ENSG00000180318](https://www.ensembl.org/id/ENSG00000180318) | ALX homeobox 1 [Source:HGNC Symbol;Acc:HGNC:1494] | 3,57 |
| AMACR | [ENSG00000242110](https://www.ensembl.org/id/ENSG00000242110) | alpha-methylacyl-CoA racemase [Source:HGNC Symbol;Acc:HGNC:451] | -1,27 |
| AMHR2 | [ENSG00000135409](https://www.ensembl.org/id/ENSG00000135409) | anti-Mullerian hormone receptor type 2 [Source:HGNC Symbol;Acc:HGNC:465] | -4,10 |
| AMIGO2 | [ENSG00000139211](https://www.ensembl.org/id/ENSG00000139211) | adhesion molecule with Ig like domain 2 [Source:HGNC Symbol;Acc:HGNC:24073] | 1,92 |
| AMOT | [ENSG00000126016](https://www.ensembl.org/id/ENSG00000126016) | angiomotin [Source:HGNC Symbol;Acc:HGNC:17810] | -2,25 |
| AMOTL2 | [ENSG00000114019](https://www.ensembl.org/id/ENSG00000114019) | angiomotin like 2 [Source:HGNC Symbol;Acc:HGNC:17812] | 1,29 |
| AMPD1 | [ENSG00000116748](https://www.ensembl.org/id/ENSG00000116748) | adenosine monophosphate deaminase 1 [Source:HGNC Symbol;Acc:HGNC:468] | -10,33 |
| AMPD3 | [ENSG00000133805](https://www.ensembl.org/id/ENSG00000133805) | adenosine monophosphate deaminase 3 [Source:HGNC Symbol;Acc:HGNC:470] | -3,86 |
| ANGPT2 | [ENSG00000091879](https://www.ensembl.org/id/ENSG00000091879) | angiopoietin 2 [Source:HGNC Symbol;Acc:HGNC:485] | 1,18 |
| ANGPT4 | [ENSG00000101280](https://www.ensembl.org/id/ENSG00000101280) | angiopoietin 4 [Source:HGNC Symbol;Acc:HGNC:487] | 1,87 |
| ANGPTL3 | [ENSG00000132855](https://www.ensembl.org/id/ENSG00000132855) | angiopoietin like 3 [Source:HGNC Symbol;Acc:HGNC:491] | 3,28 |
| ANGPTL4 | [ENSG00000167772](https://www.ensembl.org/id/ENSG00000167772) | angiopoietin like 4 [Source:HGNC Symbol;Acc:HGNC:16039] | 2,19 |
| ANGPTL7 | [ENSG00000171819](https://www.ensembl.org/id/ENSG00000171819) | angiopoietin like 7 [Source:HGNC Symbol;Acc:HGNC:24078] | -2,39 |
| ANK1 | [ENSG00000029534](https://www.ensembl.org/id/ENSG00000029534) | ankyrin 1 [Source:HGNC Symbol;Acc:HGNC:492] | -4,62 |
| ANK3 | [ENSG00000151150](https://www.ensembl.org/id/ENSG00000151150) | ankyrin 3 [Source:HGNC Symbol;Acc:HGNC:494] | -2,64 |
| ANKHD1-DT | [ENSG00000249637](https://www.ensembl.org/id/ENSG00000249637) | ANKHD1 divergent transcript [Source:HGNC Symbol;Acc:HGNC:55564] | -2,02 |
| ANKRD1 | [ENSG00000148677](https://www.ensembl.org/id/ENSG00000148677) | ankyrin repeat domain 1 [Source:HGNC Symbol;Acc:HGNC:15819] | -8,14 |
| ANKRD18B | [ENSG00000230453](https://www.ensembl.org/id/ENSG00000230453) | ankyrin repeat domain 18B [Source:HGNC Symbol;Acc:HGNC:23644] | -6,67 |
| ANKRD2 | [ENSG00000165887](https://www.ensembl.org/id/ENSG00000165887) | ankyrin repeat domain 2 [Source:HGNC Symbol;Acc:HGNC:495] | -12,23 |
| ANKRD20A5P | [ENSG00000290972](https://www.ensembl.org/id/ENSG00000290972) | ankyrin repeat domain 20 family member A5, pseudogene [Source:NCBI gene (formerly Entrezgene);Acc:440482] | 5,13 |
| ANKRD23 | [ENSG00000163126](https://www.ensembl.org/id/ENSG00000163126) | ankyrin repeat domain 23 [Source:HGNC Symbol;Acc:HGNC:24470] | -8,92 |
| ANKRD39 | [ENSG00000213337](https://www.ensembl.org/id/ENSG00000213337) | ankyrin repeat domain 39 [Source:HGNC Symbol;Acc:HGNC:28640] | -2,58 |
| ANKRD9 | [ENSG00000156381](https://www.ensembl.org/id/ENSG00000156381) | ankyrin repeat domain 9 [Source:HGNC Symbol;Acc:HGNC:20096] | -1,96 |
| ANLN | [ENSG00000011426](https://www.ensembl.org/id/ENSG00000011426) | anillin, actin binding protein [Source:HGNC Symbol;Acc:HGNC:14082] | 1,96 |
| ANO5 | [ENSG00000171714](https://www.ensembl.org/id/ENSG00000171714) | anoctamin 5 [Source:HGNC Symbol;Acc:HGNC:27337] | -2,74 |
| ANOS1 | [ENSG00000011201](https://www.ensembl.org/id/ENSG00000011201) | anosmin 1 [Source:HGNC Symbol;Acc:HGNC:6211] | -1,82 |
| AP1S2 | [ENSG00000182287](https://www.ensembl.org/id/ENSG00000182287) | adaptor related protein complex 1 subunit sigma 2 [Source:HGNC Symbol;Acc:HGNC:560] | -1,64 |
| AP5S1 | [ENSG00000125843](https://www.ensembl.org/id/ENSG00000125843) | adaptor related protein complex 5 subunit sigma 1 [Source:HGNC Symbol;Acc:HGNC:15875] | -1,45 |
| APEH | [ENSG00000164062](https://www.ensembl.org/id/ENSG00000164062) | acylaminoacyl-peptide hydrolase [Source:HGNC Symbol;Acc:HGNC:586] | -1,13 |
| APOBEC2 | [ENSG00000124701](https://www.ensembl.org/id/ENSG00000124701) | apolipoprotein B mRNA editing enzyme catalytic subunit 2 [Source:HGNC Symbol;Acc:HGNC:605] | -9,89 |
| APOBEC3H | [ENSG00000100298](https://www.ensembl.org/id/ENSG00000100298) | apolipoprotein B mRNA editing enzyme catalytic subunit 3H [Source:HGNC Symbol;Acc:HGNC:24100] | 5,30 |
| APOC1 | [ENSG00000130208](https://www.ensembl.org/id/ENSG00000130208) | apolipoprotein C1 [Source:HGNC Symbol;Acc:HGNC:607] | -3,13 |
| APOD | [ENSG00000189058](https://www.ensembl.org/id/ENSG00000189058) | apolipoprotein D [Source:HGNC Symbol;Acc:HGNC:612] | -1,44 |
| APOL5 | [ENSG00000128313](https://www.ensembl.org/id/ENSG00000128313) | apolipoprotein L5 [Source:HGNC Symbol;Acc:HGNC:14869] | -3,50 |
| APOLD1 | [ENSG00000178878](https://www.ensembl.org/id/ENSG00000178878) | apolipoprotein L domain containing 1 [Source:HGNC Symbol;Acc:HGNC:25268] | 1,27 |
| APOO | [ENSG00000184831](https://www.ensembl.org/id/ENSG00000184831) | apolipoprotein O [Source:HGNC Symbol;Acc:HGNC:28727] | -1,99 |
| APP-DT | [ENSG00000273492](https://www.ensembl.org/id/ENSG00000273492) | APP divergent transcript [Source:HGNC Symbol;Acc:HGNC:55075] | -3,58 |
| AQP4 | [ENSG00000171885](https://www.ensembl.org/id/ENSG00000171885) | aquaporin 4 [Source:HGNC Symbol;Acc:HGNC:637] | -9,64 |
| AQP7 | [ENSG00000165269](https://www.ensembl.org/id/ENSG00000165269) | aquaporin 7 [Source:HGNC Symbol;Acc:HGNC:640] | -2,00 |
| ARF4-AS1 | [ENSG00000272146](https://www.ensembl.org/id/ENSG00000272146) | ARF4 antisense RNA 1 [Source:HGNC Symbol;Acc:HGNC:51593] | -2,46 |
| ARFGEF3 | [ENSG00000112379](https://www.ensembl.org/id/ENSG00000112379) | ARFGEF family member 3 [Source:HGNC Symbol;Acc:HGNC:21213] | -2,81 |
| ARGFX | [ENSG00000186103](https://www.ensembl.org/id/ENSG00000186103) | arginine-fifty homeobox [Source:HGNC Symbol;Acc:HGNC:30146] | -3,72 |
| ARHGAP1 | [ENSG00000175220](https://www.ensembl.org/id/ENSG00000175220) | Rho GTPase activating protein 1 [Source:HGNC Symbol;Acc:HGNC:673] | 1,11 |
| ARHGAP18 | [ENSG00000146376](https://www.ensembl.org/id/ENSG00000146376) | Rho GTPase activating protein 18 [Source:HGNC Symbol;Acc:HGNC:21035] | -1,72 |
| ARHGAP22 | [ENSG00000128805](https://www.ensembl.org/id/ENSG00000128805) | Rho GTPase activating protein 22 [Source:HGNC Symbol;Acc:HGNC:30320] | -1,44 |
| ARHGAP28-AS1 | [ENSG00000266441](https://www.ensembl.org/id/ENSG00000266441) | ARHGAP28 antisense RNA 1 [Source:HGNC Symbol;Acc:HGNC:55320] | -5,63 |
| ARHGAP36 | [ENSG00000147256](https://www.ensembl.org/id/ENSG00000147256) | Rho GTPase activating protein 36 [Source:HGNC Symbol;Acc:HGNC:26388] | -4,43 |
| ARHGEF35 | [ENSG00000213214](https://www.ensembl.org/id/ENSG00000213214) | Rho guanine nucleotide exchange factor 35 [Source:HGNC Symbol;Acc:HGNC:33846] | -2,39 |
| ARHGEF4 | [ENSG00000136002](https://www.ensembl.org/id/ENSG00000136002) | Rho guanine nucleotide exchange factor 4 [Source:HGNC Symbol;Acc:HGNC:684] | -1,65 |
| ARHGEF9 | [ENSG00000131089](https://www.ensembl.org/id/ENSG00000131089) | Cdc42 guanine nucleotide exchange factor 9 [Source:HGNC Symbol;Acc:HGNC:14561] | 1,04 |
| ARPP21 | [ENSG00000172995](https://www.ensembl.org/id/ENSG00000172995) | cAMP regulated phosphoprotein 21 [Source:HGNC Symbol;Acc:HGNC:16968] | -7,01 |
| ARSJ | [ENSG00000180801](https://www.ensembl.org/id/ENSG00000180801) | arylsulfatase family member J [Source:HGNC Symbol;Acc:HGNC:26286] | 2,29 |
| ART1 | [ENSG00000129744](https://www.ensembl.org/id/ENSG00000129744) | ADP-ribosyltransferase 1 [Source:HGNC Symbol;Acc:HGNC:723] | -10,83 |
| ART3 | [ENSG00000156219](https://www.ensembl.org/id/ENSG00000156219) | ADP-ribosyltransferase 3 (inactive) [Source:HGNC Symbol;Acc:HGNC:725] | -3,06 |
| ART5 | [ENSG00000167311](https://www.ensembl.org/id/ENSG00000167311) | ADP-ribosyltransferase 5 [Source:HGNC Symbol;Acc:HGNC:24049] | -6,71 |
| ARX | [ENSG00000004848](https://www.ensembl.org/id/ENSG00000004848) | aristaless related homeobox [Source:HGNC Symbol;Acc:HGNC:18060] | -9,03 |
| ASAH1-AS1 | [ENSG00000245281](https://www.ensembl.org/id/ENSG00000245281) | ASAH1 antisense RNA 1 [Source:HGNC Symbol;Acc:HGNC:55603] | -1,95 |
| ASAP3 | [ENSG00000088280](https://www.ensembl.org/id/ENSG00000088280) | ArfGAP with SH3 domain, ankyrin repeat and PH domain 3 [Source:HGNC Symbol;Acc:HGNC:14987] | -1,14 |
| ASB10 | [ENSG00000146926](https://www.ensembl.org/id/ENSG00000146926) | ankyrin repeat and SOCS box containing 10 [Source:HGNC Symbol;Acc:HGNC:17185] | -7,14 |
| ASB11 | [ENSG00000165192](https://www.ensembl.org/id/ENSG00000165192) | ankyrin repeat and SOCS box containing 11 [Source:HGNC Symbol;Acc:HGNC:17186] | -7,71 |
| ASB12 | [ENSG00000198881](https://www.ensembl.org/id/ENSG00000198881) | ankyrin repeat and SOCS box containing 12 [Source:HGNC Symbol;Acc:HGNC:19763] | -4,78 |
| ASB14 | [ENSG00000239388](https://www.ensembl.org/id/ENSG00000239388) | ankyrin repeat and SOCS box containing 14 [Source:HGNC Symbol;Acc:HGNC:19766] | -3,86 |
| ASB15 | [ENSG00000146809](https://www.ensembl.org/id/ENSG00000146809) | ankyrin repeat and SOCS box containing 15 [Source:HGNC Symbol;Acc:HGNC:19767] | -9,13 |
| ASB16 | [ENSG00000161664](https://www.ensembl.org/id/ENSG00000161664) | ankyrin repeat and SOCS box containing 16 [Source:HGNC Symbol;Acc:HGNC:19768] | -3,34 |
| ASB16-AS1 | [ENSG00000267080](https://www.ensembl.org/id/ENSG00000267080) | ASB16 antisense RNA 1 [Source:HGNC Symbol;Acc:HGNC:25442] | -1,61 |
| ASB18 | [ENSG00000182177](https://www.ensembl.org/id/ENSG00000182177) | ankyrin repeat and SOCS box containing 18 [Source:HGNC Symbol;Acc:HGNC:19770] | -10,66 |
| ASB4 | [ENSG00000005981](https://www.ensembl.org/id/ENSG00000005981) | ankyrin repeat and SOCS box containing 4 [Source:HGNC Symbol;Acc:HGNC:16009] | -11,56 |
| ASB5 | [ENSG00000164122](https://www.ensembl.org/id/ENSG00000164122) | ankyrin repeat and SOCS box containing 5 [Source:HGNC Symbol;Acc:HGNC:17180] | -9,76 |
| ASB8 | [ENSG00000177981](https://www.ensembl.org/id/ENSG00000177981) | ankyrin repeat and SOCS box containing 8 [Source:HGNC Symbol;Acc:HGNC:17183] | -2,08 |
| ASCL5 | [ENSG00000232237](https://www.ensembl.org/id/ENSG00000232237) | achaete-scute family bHLH transcription factor 5 [Source:HGNC Symbol;Acc:HGNC:33169] | -5,17 |
| ASIP | [ENSG00000101440](https://www.ensembl.org/id/ENSG00000101440) | agouti signaling protein [Source:HGNC Symbol;Acc:HGNC:745] | -4,80 |
| ASNS | [ENSG00000070669](https://www.ensembl.org/id/ENSG00000070669) | asparagine synthetase (glutamine-hydrolyzing) [Source:HGNC Symbol;Acc:HGNC:753] | 1,30 |
| ATG4D | [ENSG00000130734](https://www.ensembl.org/id/ENSG00000130734) | autophagy related 4D cysteine peptidase [Source:HGNC Symbol;Acc:HGNC:20789] | -1,17 |
| ATG9A | [ENSG00000198925](https://www.ensembl.org/id/ENSG00000198925) | autophagy related 9A [Source:HGNC Symbol;Acc:HGNC:22408] | -1,15 |
| ATOH8 | [ENSG00000168874](https://www.ensembl.org/id/ENSG00000168874) | atonal bHLH transcription factor 8 [Source:HGNC Symbol;Acc:HGNC:24126] | 1,10 |
| ATP12A | [ENSG00000075673](https://www.ensembl.org/id/ENSG00000075673) | ATPase H+/K+ transporting non-gastric alpha2 subunit [Source:HGNC Symbol;Acc:HGNC:13816] | 3,11 |
| ATP1A2 | [ENSG00000018625](https://www.ensembl.org/id/ENSG00000018625) | ATPase Na+/K+ transporting subunit alpha 2 [Source:HGNC Symbol;Acc:HGNC:800] | -1,65 |
| ATP1B1 | [ENSG00000143153](https://www.ensembl.org/id/ENSG00000143153) | ATPase Na+/K+ transporting subunit beta 1 [Source:HGNC Symbol;Acc:HGNC:804] | -4,69 |
| ATP1B3 | [ENSG00000069849](https://www.ensembl.org/id/ENSG00000069849) | ATPase Na+/K+ transporting subunit beta 3 [Source:HGNC Symbol;Acc:HGNC:806] | 1,45 |
| ATP1B4 | [ENSG00000101892](https://www.ensembl.org/id/ENSG00000101892) | ATPase Na+/K+ transporting family member beta 4 [Source:HGNC Symbol;Acc:HGNC:808] | -9,14 |
| ATP2A1 | [ENSG00000196296](https://www.ensembl.org/id/ENSG00000196296) | ATPase sarcoplasmic/endoplasmic reticulum Ca2+ transporting 1 [Source:HGNC Symbol;Acc:HGNC:811] | -10,30 |
| ATP2A1-AS1 | [ENSG00000260442](https://www.ensembl.org/id/ENSG00000260442) | ATP2A1 antisense RNA 1 [Source:HGNC Symbol;Acc:HGNC:51370] | -3,85 |
| ATP2A2 | [ENSG00000174437](https://www.ensembl.org/id/ENSG00000174437) | ATPase sarcoplasmic/endoplasmic reticulum Ca2+ transporting 2 [Source:HGNC Symbol;Acc:HGNC:812] | -3,59 |
| ATP2B2 | [ENSG00000157087](https://www.ensembl.org/id/ENSG00000157087) | ATPase plasma membrane Ca2+ transporting 2 [Source:HGNC Symbol;Acc:HGNC:815] | -5,37 |
| ATP5F1A | [ENSG00000152234](https://www.ensembl.org/id/ENSG00000152234) | ATP synthase F1 subunit alpha [Source:HGNC Symbol;Acc:HGNC:823] | -1,57 |
| ATP5F1B | [ENSG00000110955](https://www.ensembl.org/id/ENSG00000110955) | ATP synthase F1 subunit beta [Source:HGNC Symbol;Acc:HGNC:830] | -2,22 |
| ATP5F1C | [ENSG00000165629](https://www.ensembl.org/id/ENSG00000165629) | ATP synthase F1 subunit gamma [Source:HGNC Symbol;Acc:HGNC:833] | -1,19 |
| ATP5F1D | [ENSG00000099624](https://www.ensembl.org/id/ENSG00000099624) | ATP synthase F1 subunit delta [Source:HGNC Symbol;Acc:HGNC:837] | -1,89 |
| ATP5IF1 | [ENSG00000130770](https://www.ensembl.org/id/ENSG00000130770) | ATP synthase inhibitory factor subunit 1 [Source:HGNC Symbol;Acc:HGNC:871] | -1,79 |
| ATP5MC1 | [ENSG00000159199](https://www.ensembl.org/id/ENSG00000159199) | ATP synthase membrane subunit c locus 1 [Source:HGNC Symbol;Acc:HGNC:841] | -1,71 |
| ATP5MC3 | [ENSG00000154518](https://www.ensembl.org/id/ENSG00000154518) | ATP synthase membrane subunit c locus 3 [Source:HGNC Symbol;Acc:HGNC:843] | -1,64 |
| ATP5MF | [ENSG00000241468](https://www.ensembl.org/id/ENSG00000241468) | ATP synthase membrane subunit f [Source:HGNC Symbol;Acc:HGNC:848] | -1,31 |
| ATP5MJ | [ENSG00000156411](https://www.ensembl.org/id/ENSG00000156411) | ATP synthase membrane subunit j [Source:HGNC Symbol;Acc:HGNC:1188] | -1,20 |
| ATP5PB | [ENSG00000116459](https://www.ensembl.org/id/ENSG00000116459) | ATP synthase peripheral stalk-membrane subunit b [Source:HGNC Symbol;Acc:HGNC:840] | -1,25 |
| ATP5PD | [ENSG00000167863](https://www.ensembl.org/id/ENSG00000167863) | ATP synthase peripheral stalk subunit d [Source:HGNC Symbol;Acc:HGNC:845] | -1,28 |
| ATP5PF | [ENSG00000154723](https://www.ensembl.org/id/ENSG00000154723) | ATP synthase peripheral stalk subunit F6 [Source:HGNC Symbol;Acc:HGNC:847] | -1,20 |
| ATP5PO | [ENSG00000241837](https://www.ensembl.org/id/ENSG00000241837) | ATP synthase peripheral stalk subunit OSCP [Source:HGNC Symbol;Acc:HGNC:850] | -1,13 |
| ATP8A1 | [ENSG00000124406](https://www.ensembl.org/id/ENSG00000124406) | ATPase phospholipid transporting 8A1 [Source:HGNC Symbol;Acc:HGNC:13531] | -1,86 |
| ATP8A2 | [ENSG00000132932](https://www.ensembl.org/id/ENSG00000132932) | ATPase phospholipid transporting 8A2 [Source:HGNC Symbol;Acc:HGNC:13533] | -3,18 |
| ATPAF1 | [ENSG00000123472](https://www.ensembl.org/id/ENSG00000123472) | ATP synthase mitochondrial F1 complex assembly factor 1 [Source:HGNC Symbol;Acc:HGNC:18803] | -1,46 |
| ATPAF2 | [ENSG00000171953](https://www.ensembl.org/id/ENSG00000171953) | ATP synthase mitochondrial F1 complex assembly factor 2 [Source:HGNC Symbol;Acc:HGNC:18802] | -1,35 |
| ATRNL1 | [ENSG00000107518](https://www.ensembl.org/id/ENSG00000107518) | attractin like 1 [Source:HGNC Symbol;Acc:HGNC:29063] | -2,28 |
| ATXN1-AS1 | [ENSG00000229931](https://www.ensembl.org/id/ENSG00000229931) | ATXN1 antisense RNA 1 [Source:HGNC Symbol;Acc:HGNC:40515] | 1,82 |
| AUH | [ENSG00000148090](https://www.ensembl.org/id/ENSG00000148090) | AU RNA binding methylglutaconyl-CoA hydratase [Source:HGNC Symbol;Acc:HGNC:890] | -1,41 |
| AVPI1 | [ENSG00000119986](https://www.ensembl.org/id/ENSG00000119986) | arginine vasopressin induced 1 [Source:HGNC Symbol;Acc:HGNC:30898] | 1,13 |
| AVPR1A | [ENSG00000166148](https://www.ensembl.org/id/ENSG00000166148) | arginine vasopressin receptor 1A [Source:HGNC Symbol;Acc:HGNC:895] | 2,12 |
| B3GALT1 | [ENSG00000172318](https://www.ensembl.org/id/ENSG00000172318) | beta-1,3-galactosyltransferase 1 [Source:HGNC Symbol;Acc:HGNC:916] | -2,17 |
| B3GNT4 | [ENSG00000176383](https://www.ensembl.org/id/ENSG00000176383) | UDP-GlcNAc:betaGal beta-1,3-N-acetylglucosaminyltransferase 4 [Source:HGNC Symbol;Acc:HGNC:15683] | 2,25 |
| B3GNT5 | [ENSG00000176597](https://www.ensembl.org/id/ENSG00000176597) | UDP-GlcNAc:betaGal beta-1,3-N-acetylglucosaminyltransferase 5 [Source:HGNC Symbol;Acc:HGNC:15684] | -2,20 |
| BACH1-IT2 | [ENSG00000228817](https://www.ensembl.org/id/ENSG00000228817) | BACH1 intronic transcript 2 [Source:HGNC Symbol;Acc:HGNC:40007] | 4,02 |
| BASP1 | [ENSG00000176788](https://www.ensembl.org/id/ENSG00000176788) | brain abundant membrane attached signal protein 1 [Source:HGNC Symbol;Acc:HGNC:957] | 1,18 |
| BCAT2 | [ENSG00000105552](https://www.ensembl.org/id/ENSG00000105552) | branched chain amino acid transaminase 2 [Source:HGNC Symbol;Acc:HGNC:977] | -0,87 |
| BCKDHA | [ENSG00000248098](https://www.ensembl.org/id/ENSG00000248098) | branched chain keto acid dehydrogenase E1 subunit alpha [Source:HGNC Symbol;Acc:HGNC:986] | -1,88 |
| BCL2L13 | [ENSG00000099968](https://www.ensembl.org/id/ENSG00000099968) | BCL2 like 13 [Source:HGNC Symbol;Acc:HGNC:17164] | -1,62 |
| BDH1 | [ENSG00000161267](https://www.ensembl.org/id/ENSG00000161267) | 3-hydroxybutyrate dehydrogenase 1 [Source:HGNC Symbol;Acc:HGNC:1027] | -4,27 |
| BEST3 | [ENSG00000127325](https://www.ensembl.org/id/ENSG00000127325) | bestrophin 3 [Source:HGNC Symbol;Acc:HGNC:17105] | -8,01 |
| BEST4 | [ENSG00000142959](https://www.ensembl.org/id/ENSG00000142959) | bestrophin 4 [Source:HGNC Symbol;Acc:HGNC:17106] | -2,40 |
| BEX2 | [ENSG00000133134](https://www.ensembl.org/id/ENSG00000133134) | brain expressed X-linked 2 [Source:HGNC Symbol;Acc:HGNC:30933] | -1,63 |
| BICD1 | [ENSG00000151746](https://www.ensembl.org/id/ENSG00000151746) | BICD cargo adaptor 1 [Source:HGNC Symbol;Acc:HGNC:1049] | -1,38 |
| BID | [ENSG00000015475](https://www.ensembl.org/id/ENSG00000015475) | BH3 interacting domain death agonist [Source:HGNC Symbol;Acc:HGNC:1050] | -1,35 |
| BIN1 | [ENSG00000136717](https://www.ensembl.org/id/ENSG00000136717) | bridging integrator 1 [Source:HGNC Symbol;Acc:HGNC:1052] | -4,86 |
| BIVM-ERCC5 | [ENSG00000270181](https://www.ensembl.org/id/ENSG00000270181) | BIVM-ERCC5 readthrough [Source:HGNC Symbol;Acc:HGNC:43690] | -7,14 |
| BLCAP | [ENSG00000166619](https://www.ensembl.org/id/ENSG00000166619) | BLCAP apoptosis inducing factor [Source:HGNC Symbol;Acc:HGNC:1055] | -2,27 |
| BLNK | [ENSG00000095585](https://www.ensembl.org/id/ENSG00000095585) | B cell linker [Source:HGNC Symbol;Acc:HGNC:14211] | -2,43 |
| BLOC1S2 | [ENSG00000196072](https://www.ensembl.org/id/ENSG00000196072) | biogenesis of lysosomal organelles complex 1 subunit 2 [Source:HGNC Symbol;Acc:HGNC:20984] | -1,29 |
| BMP2 | [ENSG00000125845](https://www.ensembl.org/id/ENSG00000125845) | bone morphogenetic protein 2 [Source:HGNC Symbol;Acc:HGNC:1069] | 1,72 |
| BMP3 | [ENSG00000152785](https://www.ensembl.org/id/ENSG00000152785) | bone morphogenetic protein 3 [Source:HGNC Symbol;Acc:HGNC:1070] | -2,43 |
| BMS1P1 | [ENSG00000291069](https://www.ensembl.org/id/ENSG00000291069) | BMS1 pseudogene 1 [Source:NCBI gene (formerly Entrezgene);Acc:399761] | 1,44 |
| BNC1 | [ENSG00000169594](https://www.ensembl.org/id/ENSG00000169594) | basonuclin 1 [Source:HGNC Symbol;Acc:HGNC:1081] | -5,06 |
| BNIP3 | [ENSG00000176171](https://www.ensembl.org/id/ENSG00000176171) | BCL2 interacting protein 3 [Source:HGNC Symbol;Acc:HGNC:1084] | -2,53 |
| BNIPL | [ENSG00000163141](https://www.ensembl.org/id/ENSG00000163141) | BCL2 interacting protein like [Source:HGNC Symbol;Acc:HGNC:16976] | -2,43 |
| BOLA3 | [ENSG00000163170](https://www.ensembl.org/id/ENSG00000163170) | bolA family member 3 [Source:HGNC Symbol;Acc:HGNC:24415] | -1,49 |
| BOLL | [ENSG00000152430](https://www.ensembl.org/id/ENSG00000152430) | boule homolog, RNA binding protein [Source:HGNC Symbol;Acc:HGNC:14273] | -4,46 |
| BOP1 | [ENSG00000261236](https://www.ensembl.org/id/ENSG00000261236) | BOP1 ribosomal biogenesis factor [Source:HGNC Symbol;Acc:HGNC:15519] | -0,92 |
| BORCS7-ASMT | [ENSG00000270316](https://www.ensembl.org/id/ENSG00000270316) | BORCS7-ASMT readthrough (NMD candidate) [Source:HGNC Symbol;Acc:HGNC:49183] | -8,22 |
| BRCA2 | [ENSG00000139618](https://www.ensembl.org/id/ENSG00000139618) | BRCA2 DNA repair associated [Source:HGNC Symbol;Acc:HGNC:1101] | 2,23 |
| BRINP1 | [ENSG00000078725](https://www.ensembl.org/id/ENSG00000078725) | BMP/retinoic acid inducible neural specific 1 [Source:HGNC Symbol;Acc:HGNC:2687] | -2,62 |
| BTBD1 | [ENSG00000064726](https://www.ensembl.org/id/ENSG00000064726) | BTB domain containing 1 [Source:HGNC Symbol;Acc:HGNC:1120] | -2,64 |
| BTNL3 | [ENSG00000168903](https://www.ensembl.org/id/ENSG00000168903) | butyrophilin like 3 [Source:HGNC Symbol;Acc:HGNC:1143] | -5,43 |
| BZW2 | [ENSG00000136261](https://www.ensembl.org/id/ENSG00000136261) | basic leucine zipper and W2 domains 2 [Source:HGNC Symbol;Acc:HGNC:18808] | -3,10 |
| C10orf67 | [ENSG00000179133](https://www.ensembl.org/id/ENSG00000179133) | chromosome 10 open reading frame 67 [Source:HGNC Symbol;Acc:HGNC:28716] | -4,61 |
| C10orf71 | [ENSG00000177354](https://www.ensembl.org/id/ENSG00000177354) | chromosome 10 open reading frame 71 [Source:HGNC Symbol;Acc:HGNC:26973] | -11,66 |
| C10orf71-AS1 | [ENSG00000236208](https://www.ensembl.org/id/ENSG00000236208) | C10orf71 antisense RNA 1 [Source:HGNC Symbol;Acc:HGNC:45007] | -9,47 |
| C11orf24 | [ENSG00000171067](https://www.ensembl.org/id/ENSG00000171067) | chromosome 11 open reading frame 24 [Source:HGNC Symbol;Acc:HGNC:1174] | 0,87 |
| C11orf52 | [ENSG00000149300](https://www.ensembl.org/id/ENSG00000149300) | chromosome 11 open reading frame 52 [Source:HGNC Symbol;Acc:HGNC:30531] | -2,93 |
| C11orf65 | [ENSG00000166323](https://www.ensembl.org/id/ENSG00000166323) | chromosome 11 open reading frame 65 [Source:HGNC Symbol;Acc:HGNC:28519] | -2,45 |
| C12orf73 | [ENSG00000204954](https://www.ensembl.org/id/ENSG00000204954) | chromosome 12 open reading frame 73 [Source:HGNC Symbol;Acc:HGNC:34450] | -1,53 |
| C15orf39 | [ENSG00000167173](https://www.ensembl.org/id/ENSG00000167173) | chromosome 15 open reading frame 39 [Source:HGNC Symbol;Acc:HGNC:24497] | 1,59 |
| C15orf61 | [ENSG00000189227](https://www.ensembl.org/id/ENSG00000189227) | chromosome 15 open reading frame 61 [Source:HGNC Symbol;Acc:HGNC:34453] | -2,25 |
| C19orf47 | [ENSG00000160392](https://www.ensembl.org/id/ENSG00000160392) | chromosome 19 open reading frame 47 [Source:HGNC Symbol;Acc:HGNC:26723] | -1,58 |
| C19orf81 | [ENSG00000235034](https://www.ensembl.org/id/ENSG00000235034) | chromosome 19 open reading frame 81 [Source:HGNC Symbol;Acc:HGNC:40041] | -5,87 |
| C1orf105 | [ENSG00000180999](https://www.ensembl.org/id/ENSG00000180999) | chromosome 1 open reading frame 105 [Source:HGNC Symbol;Acc:HGNC:29591] | -6,54 |
| C1orf127 | [ENSG00000175262](https://www.ensembl.org/id/ENSG00000175262) | chromosome 1 open reading frame 127 [Source:HGNC Symbol;Acc:HGNC:26730] | -5,77 |
| C1orf21 | [ENSG00000116667](https://www.ensembl.org/id/ENSG00000116667) | chromosome 1 open reading frame 21 [Source:HGNC Symbol;Acc:HGNC:15494] | -2,31 |
| C1orf21-DT | [ENSG00000271387](https://www.ensembl.org/id/ENSG00000271387) | C1orf21 divergent transcript [Source:HGNC Symbol;Acc:HGNC:55249] | -2,81 |
| C1orf43 | [ENSG00000143612](https://www.ensembl.org/id/ENSG00000143612) | chromosome 1 open reading frame 43 [Source:HGNC Symbol;Acc:HGNC:29876] | -1,19 |
| C1orf87 | [ENSG00000162598](https://www.ensembl.org/id/ENSG00000162598) | chromosome 1 open reading frame 87 [Source:HGNC Symbol;Acc:HGNC:28547] | -5,31 |
| C1QB | [ENSG00000173369](https://www.ensembl.org/id/ENSG00000173369) | complement C1q B chain [Source:HGNC Symbol;Acc:HGNC:1242] | -1,50 |
| C1QBP | [ENSG00000108561](https://www.ensembl.org/id/ENSG00000108561) | complement C1q binding protein [Source:HGNC Symbol;Acc:HGNC:1243] | -0,97 |
| C1QC | [ENSG00000159189](https://www.ensembl.org/id/ENSG00000159189) | complement C1q C chain [Source:HGNC Symbol;Acc:HGNC:1245] | -1,67 |
| C1QL1 | [ENSG00000131094](https://www.ensembl.org/id/ENSG00000131094) | complement C1q like 1 [Source:HGNC Symbol;Acc:HGNC:24182] | -3,12 |
| C1QTNF1 | [ENSG00000173918](https://www.ensembl.org/id/ENSG00000173918) | C1q and TNF related 1 [Source:HGNC Symbol;Acc:HGNC:14324] | 1,81 |
| C1QTNF3-AMACR | [ENSG00000273294](https://www.ensembl.org/id/ENSG00000273294) | C1QTNF3-AMACR readthrough (NMD candidate) [Source:HGNC Symbol;Acc:HGNC:49198] | -3,23 |
| C1QTNF7 | [ENSG00000163145](https://www.ensembl.org/id/ENSG00000163145) | C1q and TNF related 7 [Source:HGNC Symbol;Acc:HGNC:14342] | 1,25 |
| C1QTNF9 | [ENSG00000240654](https://www.ensembl.org/id/ENSG00000240654) | C1q and TNF related 9 [Source:HGNC Symbol;Acc:HGNC:28732] | -2,26 |
| C22orf31 | [ENSG00000100249](https://www.ensembl.org/id/ENSG00000100249) | chromosome 22 open reading frame 31 [Source:HGNC Symbol;Acc:HGNC:26931] | -5,42 |
| C2orf74 | [ENSG00000237651](https://www.ensembl.org/id/ENSG00000237651) | chromosome 2 open reading frame 74 [Source:HGNC Symbol;Acc:HGNC:34439] | -1,02 |
| C2orf88 | [ENSG00000187699](https://www.ensembl.org/id/ENSG00000187699) | chromosome 2 open reading frame 88 [Source:HGNC Symbol;Acc:HGNC:28191] | -2,06 |
| C3orf70 | [ENSG00000187068](https://www.ensembl.org/id/ENSG00000187068) | chromosome 3 open reading frame 70 [Source:HGNC Symbol;Acc:HGNC:33731] | 1,47 |
| C4A | [ENSG00000244731](https://www.ensembl.org/id/ENSG00000244731) | complement C4A (Rodgers blood group) [Source:HGNC Symbol;Acc:HGNC:1323] | 1,66 |
| C4B | [ENSG00000224389](https://www.ensembl.org/id/ENSG00000224389) | complement C4B (Chido blood group) [Source:HGNC Symbol;Acc:HGNC:1324] | 1,44 |
| C4orf54 | [ENSG00000248713](https://www.ensembl.org/id/ENSG00000248713) | chromosome 4 open reading frame 54 [Source:HGNC Symbol;Acc:HGNC:27741] | -9,38 |
| C5orf24 | [ENSG00000181904](https://www.ensembl.org/id/ENSG00000181904) | chromosome 5 open reading frame 24 [Source:HGNC Symbol;Acc:HGNC:26746] | 1,06 |
| C5orf63 | [ENSG00000164241](https://www.ensembl.org/id/ENSG00000164241) | chromosome 5 open reading frame 63 [Source:HGNC Symbol;Acc:HGNC:40051] | -3,02 |
| C6orf136 | [ENSG00000204564](https://www.ensembl.org/id/ENSG00000204564) | chromosome 6 open reading frame 136 [Source:HGNC Symbol;Acc:HGNC:21301] | -1,85 |
| C8G | [ENSG00000176919](https://www.ensembl.org/id/ENSG00000176919) | complement C8 gamma chain [Source:HGNC Symbol;Acc:HGNC:1354] | -2,68 |
| C8orf34 | [ENSG00000165084](https://www.ensembl.org/id/ENSG00000165084) | chromosome 8 open reading frame 34 [Source:HGNC Symbol;Acc:HGNC:30905] | -1,79 |
| CA1 | [ENSG00000133742](https://www.ensembl.org/id/ENSG00000133742) | carbonic anhydrase 1 [Source:HGNC Symbol;Acc:HGNC:1368] | 2,69 |
| CA14 | [ENSG00000118298](https://www.ensembl.org/id/ENSG00000118298) | carbonic anhydrase 14 [Source:HGNC Symbol;Acc:HGNC:1372] | -4,35 |
| CA2 | [ENSG00000104267](https://www.ensembl.org/id/ENSG00000104267) | carbonic anhydrase 2 [Source:HGNC Symbol;Acc:HGNC:1373] | -3,57 |
| CA3 | [ENSG00000164879](https://www.ensembl.org/id/ENSG00000164879) | carbonic anhydrase 3 [Source:HGNC Symbol;Acc:HGNC:1374] | -9,98 |
| CA3-AS1 | [ENSG00000253549](https://www.ensembl.org/id/ENSG00000253549) | CA3 antisense RNA 1 [Source:HGNC Symbol;Acc:HGNC:51657] | -3,49 |
| CA7 | [ENSG00000168748](https://www.ensembl.org/id/ENSG00000168748) | carbonic anhydrase 7 [Source:HGNC Symbol;Acc:HGNC:1381] | -5,67 |
| CACNA1A | [ENSG00000141837](https://www.ensembl.org/id/ENSG00000141837) | calcium voltage-gated channel subunit alpha1 A [Source:HGNC Symbol;Acc:HGNC:1388] | 2,23 |
| CACNA1D | [ENSG00000157388](https://www.ensembl.org/id/ENSG00000157388) | calcium voltage-gated channel subunit alpha1 D [Source:HGNC Symbol;Acc:HGNC:1391] | -3,19 |
| CACNA1F | [ENSG00000102001](https://www.ensembl.org/id/ENSG00000102001) | calcium voltage-gated channel subunit alpha1 F [Source:HGNC Symbol;Acc:HGNC:1393] | 2,48 |
| CACNA1H | [ENSG00000196557](https://www.ensembl.org/id/ENSG00000196557) | calcium voltage-gated channel subunit alpha1 H [Source:HGNC Symbol;Acc:HGNC:1395] | 2,38 |
| CACNA1S | [ENSG00000081248](https://www.ensembl.org/id/ENSG00000081248) | calcium voltage-gated channel subunit alpha1 S [Source:HGNC Symbol;Acc:HGNC:1397] | -8,10 |
| CACNA2D1 | [ENSG00000153956](https://www.ensembl.org/id/ENSG00000153956) | calcium voltage-gated channel auxiliary subunit alpha2delta 1 [Source:HGNC Symbol;Acc:HGNC:1399] | -1,73 |
| CACNA2D3 | [ENSG00000157445](https://www.ensembl.org/id/ENSG00000157445) | calcium voltage-gated channel auxiliary subunit alpha2delta 3 [Source:HGNC Symbol;Acc:HGNC:15460] | -2,08 |
| CACNB1 | [ENSG00000067191](https://www.ensembl.org/id/ENSG00000067191) | calcium voltage-gated channel auxiliary subunit beta 1 [Source:HGNC Symbol;Acc:HGNC:1401] | -2,55 |
| CACNG1 | [ENSG00000108878](https://www.ensembl.org/id/ENSG00000108878) | calcium voltage-gated channel auxiliary subunit gamma 1 [Source:HGNC Symbol;Acc:HGNC:1405] | -8,59 |
| CACNG6 | [ENSG00000130433](https://www.ensembl.org/id/ENSG00000130433) | calcium voltage-gated channel auxiliary subunit gamma 6 [Source:HGNC Symbol;Acc:HGNC:13625] | -9,00 |
| CADM2 | [ENSG00000175161](https://www.ensembl.org/id/ENSG00000175161) | cell adhesion molecule 2 [Source:HGNC Symbol;Acc:HGNC:29849] | -2,98 |
| CADM4 | [ENSG00000105767](https://www.ensembl.org/id/ENSG00000105767) | cell adhesion molecule 4 [Source:HGNC Symbol;Acc:HGNC:30825] | -1,97 |
| CALCA | [ENSG00000110680](https://www.ensembl.org/id/ENSG00000110680) | calcitonin related polypeptide alpha [Source:HGNC Symbol;Acc:HGNC:1437] | 2,84 |
| CALCR | [ENSG00000004948](https://www.ensembl.org/id/ENSG00000004948) | calcitonin receptor [Source:HGNC Symbol;Acc:HGNC:1440] | -3,43 |
| CALHM5 | [ENSG00000178033](https://www.ensembl.org/id/ENSG00000178033) | calcium homeostasis modulator family member 5 [Source:HGNC Symbol;Acc:HGNC:21568] | 1,26 |
| CALHM6 | [ENSG00000188820](https://www.ensembl.org/id/ENSG00000188820) | calcium homeostasis modulator family member 6 [Source:HGNC Symbol;Acc:HGNC:33391] | -1,97 |
| CALML6 | [ENSG00000169885](https://www.ensembl.org/id/ENSG00000169885) | calmodulin like 6 [Source:HGNC Symbol;Acc:HGNC:24193] | -5,66 |
| CAMK1G | [ENSG00000008118](https://www.ensembl.org/id/ENSG00000008118) | calcium/calmodulin dependent protein kinase IG [Source:HGNC Symbol;Acc:HGNC:14585] | -4,57 |
| CAMK2A | [ENSG00000070808](https://www.ensembl.org/id/ENSG00000070808) | calcium/calmodulin dependent protein kinase II alpha [Source:HGNC Symbol;Acc:HGNC:1460] | -6,35 |
| CAMK2B | [ENSG00000058404](https://www.ensembl.org/id/ENSG00000058404) | calcium/calmodulin dependent protein kinase II beta [Source:HGNC Symbol;Acc:HGNC:1461] | -6,40 |
| CAND2 | [ENSG00000144712](https://www.ensembl.org/id/ENSG00000144712) | cullin associated and neddylation dissociated 2 (putative) [Source:HGNC Symbol;Acc:HGNC:30689] | -1,70 |
| CAP2 | [ENSG00000112186](https://www.ensembl.org/id/ENSG00000112186) | cyclase associated actin cytoskeleton regulatory protein 2 [Source:HGNC Symbol;Acc:HGNC:20039] | -1,74 |
| CAPN1-AS1 | [ENSG00000254614](https://www.ensembl.org/id/ENSG00000254614) | CAPN1 antisense RNA 1 [Source:HGNC Symbol;Acc:HGNC:56068] | -2,09 |
| CAPN3 | [ENSG00000092529](https://www.ensembl.org/id/ENSG00000092529) | calpain 3 [Source:HGNC Symbol;Acc:HGNC:1480] | -4,68 |
| CARMN | [ENSG00000249669](https://www.ensembl.org/id/ENSG00000249669) | cardiac mesoderm enhancer-associated non-coding RNA [Source:HGNC Symbol;Acc:HGNC:42872] | 1,98 |
| CARTPT | [ENSG00000164326](https://www.ensembl.org/id/ENSG00000164326) | CART prepropeptide [Source:HGNC Symbol;Acc:HGNC:24323] | -7,17 |
| CASKIN1 | [ENSG00000167971](https://www.ensembl.org/id/ENSG00000167971) | CASK interacting protein 1 [Source:HGNC Symbol;Acc:HGNC:20879] | -3,29 |
| CASQ1 | [ENSG00000143318](https://www.ensembl.org/id/ENSG00000143318) | calsequestrin 1 [Source:HGNC Symbol;Acc:HGNC:1512] | -8,98 |
| CASTOR2 | [ENSG00000274070](https://www.ensembl.org/id/ENSG00000274070) | cytosolic arginine sensor for mTORC1 subunit 2 [Source:HGNC Symbol;Acc:HGNC:37073] | -3,15 |
| CASTOR3 | [ENSG00000291122](https://www.ensembl.org/id/ENSG00000291122) | CASTOR family member 3, pseudogene [Source:HGNC Symbol;Acc:HGNC:29954] | -3,36 |
| CAV2 | [ENSG00000105971](https://www.ensembl.org/id/ENSG00000105971) | caveolin 2 [Source:HGNC Symbol;Acc:HGNC:1528] | 1,12 |
| CAV3 | [ENSG00000182533](https://www.ensembl.org/id/ENSG00000182533) | caveolin 3 [Source:HGNC Symbol;Acc:HGNC:1529] | -3,93 |
| CAVIN4 | [ENSG00000170681](https://www.ensembl.org/id/ENSG00000170681) | caveolae associated protein 4 [Source:HGNC Symbol;Acc:HGNC:33742] | -7,63 |
| CBARP | [ENSG00000099625](https://www.ensembl.org/id/ENSG00000099625) | CACN subunit beta associated regulatory protein [Source:HGNC Symbol;Acc:HGNC:28617] | 1,76 |
| CBL | [ENSG00000110395](https://www.ensembl.org/id/ENSG00000110395) | Cbl proto-oncogene [Source:HGNC Symbol;Acc:HGNC:1541] | 1,07 |
| CBLN4 | [ENSG00000054803](https://www.ensembl.org/id/ENSG00000054803) | cerebellin 4 precursor [Source:HGNC Symbol;Acc:HGNC:16231] | -3,86 |
| CBS | [ENSG00000160200](https://www.ensembl.org/id/ENSG00000160200) | cystathionine beta-synthase [Source:HGNC Symbol;Acc:HGNC:1550] | -1,65 |
| CBY3 | [ENSG00000204659](https://www.ensembl.org/id/ENSG00000204659) | chibby family member 3 [Source:HGNC Symbol;Acc:HGNC:33278] | -5,31 |
| CCDC106 | [ENSG00000173581](https://www.ensembl.org/id/ENSG00000173581) | coiled-coil domain containing 106 [Source:HGNC Symbol;Acc:HGNC:30181] | -1,01 |
| CCDC107 | [ENSG00000159884](https://www.ensembl.org/id/ENSG00000159884) | coiled-coil domain containing 107 [Source:HGNC Symbol;Acc:HGNC:28465] | 1,05 |
| CCDC136 | [ENSG00000128596](https://www.ensembl.org/id/ENSG00000128596) | coiled-coil domain containing 136 [Source:HGNC Symbol;Acc:HGNC:22225] | -1,92 |
| CCDC140 | [ENSG00000163081](https://www.ensembl.org/id/ENSG00000163081) | CCDC140 long non-coding RNA [Source:HGNC Symbol;Acc:HGNC:26514] | -5,63 |
| CCDC141 | [ENSG00000163492](https://www.ensembl.org/id/ENSG00000163492) | coiled-coil domain containing 141 [Source:HGNC Symbol;Acc:HGNC:26821] | -4,80 |
| CCDC15-DT | [ENSG00000285825](https://www.ensembl.org/id/ENSG00000285825) | CCDC15 divergent transcript [Source:HGNC Symbol;Acc:HGNC:54193] | -4,23 |
| CCDC190 | [ENSG00000185860](https://www.ensembl.org/id/ENSG00000185860) | coiled-coil domain containing 190 [Source:HGNC Symbol;Acc:HGNC:28736] | 1,81 |
| CCDC28B | [ENSG00000160050](https://www.ensembl.org/id/ENSG00000160050) | coiled-coil domain containing 28B [Source:HGNC Symbol;Acc:HGNC:28163] | -2,64 |
| CCDC3 | [ENSG00000151468](https://www.ensembl.org/id/ENSG00000151468) | coiled-coil domain containing 3 [Source:HGNC Symbol;Acc:HGNC:23813] | 1,65 |
| CCDC43 | [ENSG00000180329](https://www.ensembl.org/id/ENSG00000180329) | coiled-coil domain containing 43 [Source:HGNC Symbol;Acc:HGNC:26472] | -1,90 |
| CCDC81 | [ENSG00000149201](https://www.ensembl.org/id/ENSG00000149201) | coiled-coil domain containing 81 [Source:HGNC Symbol;Acc:HGNC:26281] | 2,63 |
| CCL21 | [ENSG00000137077](https://www.ensembl.org/id/ENSG00000137077) | C-C motif chemokine ligand 21 [Source:HGNC Symbol;Acc:HGNC:10620] | 2,04 |
| CCL25 | [ENSG00000131142](https://www.ensembl.org/id/ENSG00000131142) | C-C motif chemokine ligand 25 [Source:HGNC Symbol;Acc:HGNC:10624] | -6,04 |
| CCL8 | [ENSG00000108700](https://www.ensembl.org/id/ENSG00000108700) | C-C motif chemokine ligand 8 [Source:HGNC Symbol;Acc:HGNC:10635] | 1,85 |
| CCNB3 | [ENSG00000147082](https://www.ensembl.org/id/ENSG00000147082) | cyclin B3 [Source:HGNC Symbol;Acc:HGNC:18709] | -3,30 |
| CCR3 | [ENSG00000183625](https://www.ensembl.org/id/ENSG00000183625) | C-C motif chemokine receptor 3 [Source:HGNC Symbol;Acc:HGNC:1604] | -4,83 |
| CCR5AS | [ENSG00000223552](https://www.ensembl.org/id/ENSG00000223552) | CCR5 antisense RNA [Source:HGNC Symbol;Acc:HGNC:54398] | -2,86 |
| CD300LG | [ENSG00000161649](https://www.ensembl.org/id/ENSG00000161649) | CD300 molecule like family member g [Source:HGNC Symbol;Acc:HGNC:30455] | -1,25 |
| CD36 | [ENSG00000135218](https://www.ensembl.org/id/ENSG00000135218) | CD36 molecule [Source:HGNC Symbol;Acc:HGNC:1663] | -1,49 |
| CD38 | [ENSG00000004468](https://www.ensembl.org/id/ENSG00000004468) | CD38 molecule [Source:HGNC Symbol;Acc:HGNC:1667] | -2,94 |
| CDC20-DT | [ENSG00000234694](https://www.ensembl.org/id/ENSG00000234694) | CDC20 divergent transcript [Source:HGNC Symbol;Acc:HGNC:55681] | -2,53 |
| CDCP1 | [ENSG00000163814](https://www.ensembl.org/id/ENSG00000163814) | CUB domain containing protein 1 [Source:HGNC Symbol;Acc:HGNC:24357] | -2,56 |
| CDH15 | [ENSG00000129910](https://www.ensembl.org/id/ENSG00000129910) | cadherin 15 [Source:HGNC Symbol;Acc:HGNC:1754] | -7,53 |
| CDH2 | [ENSG00000170558](https://www.ensembl.org/id/ENSG00000170558) | cadherin 2 [Source:HGNC Symbol;Acc:HGNC:1759] | -2,05 |
| CDH6 | [ENSG00000113361](https://www.ensembl.org/id/ENSG00000113361) | cadherin 6 [Source:HGNC Symbol;Acc:HGNC:1765] | 1,91 |
| CDH8 | [ENSG00000150394](https://www.ensembl.org/id/ENSG00000150394) | cadherin 8 [Source:HGNC Symbol;Acc:HGNC:1767] | -2,57 |
| CDHR5 | [ENSG00000099834](https://www.ensembl.org/id/ENSG00000099834) | cadherin related family member 5 [Source:HGNC Symbol;Acc:HGNC:7521] | 2,66 |
| CDK14 | [ENSG00000058091](https://www.ensembl.org/id/ENSG00000058091) | cyclin dependent kinase 14 [Source:HGNC Symbol;Acc:HGNC:8883] | 1,37 |
| CDK16 | [ENSG00000102225](https://www.ensembl.org/id/ENSG00000102225) | cyclin dependent kinase 16 [Source:HGNC Symbol;Acc:HGNC:8749] | -1,58 |
| CDK18 | [ENSG00000117266](https://www.ensembl.org/id/ENSG00000117266) | cyclin dependent kinase 18 [Source:HGNC Symbol;Acc:HGNC:8751] | 1,03 |
| CDKN1C | [ENSG00000129757](https://www.ensembl.org/id/ENSG00000129757) | cyclin dependent kinase inhibitor 1C [Source:HGNC Symbol;Acc:HGNC:1786] | -1,09 |
| CDNF | [ENSG00000185267](https://www.ensembl.org/id/ENSG00000185267) | cerebral dopamine neurotrophic factor [Source:HGNC Symbol;Acc:HGNC:24913] | -4,22 |
| CDR2L | [ENSG00000109089](https://www.ensembl.org/id/ENSG00000109089) | cerebellar degeneration related protein 2 like [Source:HGNC Symbol;Acc:HGNC:29999] | 1,05 |
| CEACAM16 | [ENSG00000213892](https://www.ensembl.org/id/ENSG00000213892) | CEA cell adhesion molecule 16, tectorial membrane component [Source:HGNC Symbol;Acc:HGNC:31948] | -5,23 |
| CEBPB-AS1 | [ENSG00000277449](https://www.ensembl.org/id/ENSG00000277449) | CEBPB antisense RNA 1 [Source:HGNC Symbol;Acc:HGNC:51226] | -1,26 |
| CECR2 | [ENSG00000099954](https://www.ensembl.org/id/ENSG00000099954) | CECR2 histone acetyl-lysine reader [Source:HGNC Symbol;Acc:HGNC:1840] | -5,13 |
| CELF5 | [ENSG00000161082](https://www.ensembl.org/id/ENSG00000161082) | CUGBP Elav-like family member 5 [Source:HGNC Symbol;Acc:HGNC:14058] | -4,10 |
| CEP85 | [ENSG00000130695](https://www.ensembl.org/id/ENSG00000130695) | centrosomal protein 85 [Source:HGNC Symbol;Acc:HGNC:25309] | -4,40 |
| CEROX1 | [ENSG00000260807](https://www.ensembl.org/id/ENSG00000260807) | cytoplasmic endogenous regulator of oxidative phosphorylation 1 [Source:HGNC Symbol;Acc:HGNC:53928] | -1,60 |
| CERS1 | [ENSG00000223802](https://www.ensembl.org/id/ENSG00000223802) | ceramide synthase 1 [Source:HGNC Symbol;Acc:HGNC:14253] | -3,25 |
| CES3 | [ENSG00000172828](https://www.ensembl.org/id/ENSG00000172828) | carboxylesterase 3 [Source:HGNC Symbol;Acc:HGNC:1865] | -4,18 |
| CFAP206 | [ENSG00000272514](https://www.ensembl.org/id/ENSG00000272514) | cilia and flagella associated protein 206 [Source:HGNC Symbol;Acc:HGNC:21405] | -3,25 |
| CFAP221 | [ENSG00000163075](https://www.ensembl.org/id/ENSG00000163075) | cilia and flagella associated protein 221 [Source:HGNC Symbol;Acc:HGNC:33720] | 4,45 |
| CFAP46 | [ENSG00000171811](https://www.ensembl.org/id/ENSG00000171811) | cilia and flagella associated protein 46 [Source:HGNC Symbol;Acc:HGNC:25247] | -6,57 |
| CFAP54 | [ENSG00000188596](https://www.ensembl.org/id/ENSG00000188596) | cilia and flagella associated protein 54 [Source:HGNC Symbol;Acc:HGNC:26456] | 5,92 |
| CFAP61 | [ENSG00000089101](https://www.ensembl.org/id/ENSG00000089101) | cilia and flagella associated protein 61 [Source:HGNC Symbol;Acc:HGNC:15872] | -8,95 |
| CFL2 | [ENSG00000165410](https://www.ensembl.org/id/ENSG00000165410) | cofilin 2 [Source:HGNC Symbol;Acc:HGNC:1875] | -2,15 |
| CGB7 | [ENSG00000196337](https://www.ensembl.org/id/ENSG00000196337) | chorionic gonadotropin subunit beta 7 [Source:HGNC Symbol;Acc:HGNC:16451] | -4,31 |
| CGREF1 | [ENSG00000138028](https://www.ensembl.org/id/ENSG00000138028) | cell growth regulator with EF-hand domain 1 [Source:HGNC Symbol;Acc:HGNC:16962] | -1,75 |
| CH25H | [ENSG00000138135](https://www.ensembl.org/id/ENSG00000138135) | cholesterol 25-hydroxylase [Source:HGNC Symbol;Acc:HGNC:1907] | 2,41 |
| CHAD | [ENSG00000136457](https://www.ensembl.org/id/ENSG00000136457) | chondroadherin [Source:HGNC Symbol;Acc:HGNC:1909] | -3,15 |
| CHAF1B | [ENSG00000159259](https://www.ensembl.org/id/ENSG00000159259) | chromatin assembly factor 1 subunit B [Source:HGNC Symbol;Acc:HGNC:1911] | -1,87 |
| CHCHD10 | [ENSG00000250479](https://www.ensembl.org/id/ENSG00000250479) | coiled-coil-helix-coiled-coil-helix domain containing 10 [Source:HGNC Symbol;Acc:HGNC:15559] | -2,86 |
| CHCHD3 | [ENSG00000106554](https://www.ensembl.org/id/ENSG00000106554) | coiled-coil-helix-coiled-coil-helix domain containing 3 [Source:HGNC Symbol;Acc:HGNC:21906] | -1,70 |
| CHDH | [ENSG00000016391](https://www.ensembl.org/id/ENSG00000016391) | choline dehydrogenase [Source:HGNC Symbol;Acc:HGNC:24288] | -2,50 |
| CHEK1 | [ENSG00000149554](https://www.ensembl.org/id/ENSG00000149554) | checkpoint kinase 1 [Source:HGNC Symbol;Acc:HGNC:1925] | 1,70 |
| CHKB-CPT1B | [ENSG00000254413](https://www.ensembl.org/id/ENSG00000254413) | CHKB-CPT1B readthrough (NMD candidate) [Source:HGNC Symbol;Acc:HGNC:41998] | -1,80 |
| CHML | [ENSG00000203668](https://www.ensembl.org/id/ENSG00000203668) | CHM like Rab escort protein [Source:HGNC Symbol;Acc:HGNC:1941] | 0,93 |
| CHMP1B | [ENSG00000255112](https://www.ensembl.org/id/ENSG00000255112) | charged multivesicular body protein 1B [Source:HGNC Symbol;Acc:HGNC:24287] | 1,32 |
| CHN1 | [ENSG00000128656](https://www.ensembl.org/id/ENSG00000128656) | chimerin 1 [Source:HGNC Symbol;Acc:HGNC:1943] | 1,14 |
| CHRDL2 | [ENSG00000054938](https://www.ensembl.org/id/ENSG00000054938) | chordin like 2 [Source:HGNC Symbol;Acc:HGNC:24168] | -4,11 |
| CHRNA1 | [ENSG00000138435](https://www.ensembl.org/id/ENSG00000138435) | cholinergic receptor nicotinic alpha 1 subunit [Source:HGNC Symbol;Acc:HGNC:1955] | -5,77 |
| CHRNA10 | [ENSG00000129749](https://www.ensembl.org/id/ENSG00000129749) | cholinergic receptor nicotinic alpha 10 subunit [Source:HGNC Symbol;Acc:HGNC:13800] | -2,94 |
| CHRNA3 | [ENSG00000080644](https://www.ensembl.org/id/ENSG00000080644) | cholinergic receptor nicotinic alpha 3 subunit [Source:HGNC Symbol;Acc:HGNC:1957] | -3,72 |
| CHRNA7 | [ENSG00000175344](https://www.ensembl.org/id/ENSG00000175344) | cholinergic receptor nicotinic alpha 7 subunit [Source:HGNC Symbol;Acc:HGNC:1960] | -2,55 |
| CHRNB1 | [ENSG00000170175](https://www.ensembl.org/id/ENSG00000170175) | cholinergic receptor nicotinic beta 1 subunit [Source:HGNC Symbol;Acc:HGNC:1961] | -3,25 |
| CHRND | [ENSG00000135902](https://www.ensembl.org/id/ENSG00000135902) | cholinergic receptor nicotinic delta subunit [Source:HGNC Symbol;Acc:HGNC:1965] | -11,99 |
| CHRNG | [ENSG00000196811](https://www.ensembl.org/id/ENSG00000196811) | cholinergic receptor nicotinic gamma subunit [Source:HGNC Symbol;Acc:HGNC:1967] | -6,22 |
| CHST1 | [ENSG00000175264](https://www.ensembl.org/id/ENSG00000175264) | carbohydrate sulfotransferase 1 [Source:HGNC Symbol;Acc:HGNC:1969] | -1,72 |
| CHST15 | [ENSG00000182022](https://www.ensembl.org/id/ENSG00000182022) | carbohydrate sulfotransferase 15 [Source:HGNC Symbol;Acc:HGNC:18137] | 1,29 |
| CIAO2A | [ENSG00000166797](https://www.ensembl.org/id/ENSG00000166797) | cytosolic iron-sulfur assembly component 2A [Source:HGNC Symbol;Acc:HGNC:26235] | -1,61 |
| CILP2 | [ENSG00000160161](https://www.ensembl.org/id/ENSG00000160161) | cartilage intermediate layer protein 2 [Source:HGNC Symbol;Acc:HGNC:24213] | -2,46 |
| CIPC | [ENSG00000198894](https://www.ensembl.org/id/ENSG00000198894) | CLOCK interacting pacemaker [Source:HGNC Symbol;Acc:HGNC:20365] | -1,30 |
| CISD1 | [ENSG00000122873](https://www.ensembl.org/id/ENSG00000122873) | CDGSH iron sulfur domain 1 [Source:HGNC Symbol;Acc:HGNC:30880] | -1,77 |
| CKM | [ENSG00000104879](https://www.ensembl.org/id/ENSG00000104879) | creatine kinase, M-type [Source:HGNC Symbol;Acc:HGNC:1994] | -11,84 |
| CKMT2 | [ENSG00000131730](https://www.ensembl.org/id/ENSG00000131730) | creatine kinase, mitochondrial 2 [Source:HGNC Symbol;Acc:HGNC:1996] | -3,71 |
| CLCN1 | [ENSG00000188037](https://www.ensembl.org/id/ENSG00000188037) | chloride voltage-gated channel 1 [Source:HGNC Symbol;Acc:HGNC:2019] | -8,77 |
| CLCN4 | [ENSG00000073464](https://www.ensembl.org/id/ENSG00000073464) | chloride voltage-gated channel 4 [Source:HGNC Symbol;Acc:HGNC:2022] | -2,68 |
| CLDN19 | [ENSG00000164007](https://www.ensembl.org/id/ENSG00000164007) | claudin 19 [Source:HGNC Symbol;Acc:HGNC:2040] | -3,01 |
| CLEC10A | [ENSG00000132514](https://www.ensembl.org/id/ENSG00000132514) | C-type lectin domain containing 10A [Source:HGNC Symbol;Acc:HGNC:16916] | -1,93 |
| CLEC2L | [ENSG00000236279](https://www.ensembl.org/id/ENSG00000236279) | C-type lectin domain family 2 member L [Source:HGNC Symbol;Acc:HGNC:21969] | -5,40 |
| CLEC4F | [ENSG00000152672](https://www.ensembl.org/id/ENSG00000152672) | C-type lectin domain family 4 member F [Source:HGNC Symbol;Acc:HGNC:25357] | -2,43 |
| CLEC9A | [ENSG00000197992](https://www.ensembl.org/id/ENSG00000197992) | C-type lectin domain containing 9A [Source:HGNC Symbol;Acc:HGNC:26705] | -3,84 |
| CLIC5 | [ENSG00000112782](https://www.ensembl.org/id/ENSG00000112782) | chloride intracellular channel 5 [Source:HGNC Symbol;Acc:HGNC:13517] | -5,24 |
| CLMN | [ENSG00000165959](https://www.ensembl.org/id/ENSG00000165959) | calmin [Source:HGNC Symbol;Acc:HGNC:19972] | 1,24 |
| CLPX | [ENSG00000166855](https://www.ensembl.org/id/ENSG00000166855) | caseinolytic mitochondrial matrix peptidase chaperone subunit X [Source:HGNC Symbol;Acc:HGNC:2088] | -1,15 |
| CLTCL1 | [ENSG00000070371](https://www.ensembl.org/id/ENSG00000070371) | clathrin heavy chain like 1 [Source:HGNC Symbol;Acc:HGNC:2093] | -2,82 |
| CLUH | [ENSG00000132361](https://www.ensembl.org/id/ENSG00000132361) | clustered mitochondria homolog [Source:HGNC Symbol;Acc:HGNC:29094] | -1,35 |
| CMBL | [ENSG00000164237](https://www.ensembl.org/id/ENSG00000164237) | carboxymethylenebutenolidase homolog [Source:HGNC Symbol;Acc:HGNC:25090] | -1,82 |
| CMYA5 | [ENSG00000164309](https://www.ensembl.org/id/ENSG00000164309) | cardiomyopathy associated 5 [Source:HGNC Symbol;Acc:HGNC:14305] | -10,32 |
| CNBD2 | [ENSG00000149646](https://www.ensembl.org/id/ENSG00000149646) | cyclic nucleotide binding domain containing 2 [Source:HGNC Symbol;Acc:HGNC:16145] | -3,50 |
| CNKSR1 | [ENSG00000142675](https://www.ensembl.org/id/ENSG00000142675) | connector enhancer of kinase suppressor of Ras 1 [Source:HGNC Symbol;Acc:HGNC:19700] | -4,09 |
| CNMD | [ENSG00000136110](https://www.ensembl.org/id/ENSG00000136110) | chondromodulin [Source:HGNC Symbol;Acc:HGNC:17005] | -6,17 |
| CNNM2 | [ENSG00000148842](https://www.ensembl.org/id/ENSG00000148842) | cyclin and CBS domain divalent metal cation transport mediator 2 [Source:HGNC Symbol;Acc:HGNC:103] | 1,67 |
| CNNM3 | [ENSG00000168763](https://www.ensembl.org/id/ENSG00000168763) | cyclin and CBS domain divalent metal cation transport mediator 3 [Source:HGNC Symbol;Acc:HGNC:104] | -1,25 |
| CNNM4 | [ENSG00000158158](https://www.ensembl.org/id/ENSG00000158158) | cyclin and CBS domain divalent metal cation transport mediator 4 [Source:HGNC Symbol;Acc:HGNC:105] | -1,82 |
| CNTFR-AS1 | [ENSG00000237159](https://www.ensembl.org/id/ENSG00000237159) | CNTFR antisense RNA 1 [Source:HGNC Symbol;Acc:HGNC:48712] | -4,30 |
| COA6 | [ENSG00000168275](https://www.ensembl.org/id/ENSG00000168275) | cytochrome c oxidase assembly factor 6 [Source:HGNC Symbol;Acc:HGNC:18025] | -1,46 |
| COA8 | [ENSG00000256053](https://www.ensembl.org/id/ENSG00000256053) | cytochrome c oxidase assembly factor 8 [Source:HGNC Symbol;Acc:HGNC:20492] | -1,23 |
| COBL | [ENSG00000106078](https://www.ensembl.org/id/ENSG00000106078) | cordon-bleu WH2 repeat protein [Source:HGNC Symbol;Acc:HGNC:22199] | -6,82 |
| COBLL1 | [ENSG00000082438](https://www.ensembl.org/id/ENSG00000082438) | cordon-bleu WH2 repeat protein like 1 [Source:HGNC Symbol;Acc:HGNC:23571] | 1,06 |
| COL11A1 | [ENSG00000060718](https://www.ensembl.org/id/ENSG00000060718) | collagen type XI alpha 1 chain [Source:HGNC Symbol;Acc:HGNC:2186] | -3,17 |
| COL13A1 | [ENSG00000197467](https://www.ensembl.org/id/ENSG00000197467) | collagen type XIII alpha 1 chain [Source:HGNC Symbol;Acc:HGNC:2190] | 1,52 |
| COL18A1-AS1 | [ENSG00000183535](https://www.ensembl.org/id/ENSG00000183535) | COL18A1 antisense RNA 1 [Source:HGNC Symbol;Acc:HGNC:23132] | 2,08 |
| COL21A1 | [ENSG00000124749](https://www.ensembl.org/id/ENSG00000124749) | collagen type XXI alpha 1 chain [Source:HGNC Symbol;Acc:HGNC:17025] | -1,05 |
| COL22A1 | [ENSG00000169436](https://www.ensembl.org/id/ENSG00000169436) | collagen type XXII alpha 1 chain [Source:HGNC Symbol;Acc:HGNC:22989] | -7,44 |
| COL4A3 | [ENSG00000169031](https://www.ensembl.org/id/ENSG00000169031) | collagen type IV alpha 3 chain [Source:HGNC Symbol;Acc:HGNC:2204] | -2,43 |
| COL4A4 | [ENSG00000081052](https://www.ensembl.org/id/ENSG00000081052) | collagen type IV alpha 4 chain [Source:HGNC Symbol;Acc:HGNC:2206] | -2,19 |
| COL8A2 | [ENSG00000171812](https://www.ensembl.org/id/ENSG00000171812) | collagen type VIII alpha 2 chain [Source:HGNC Symbol;Acc:HGNC:2216] | 1,32 |
| COLGALT2 | [ENSG00000198756](https://www.ensembl.org/id/ENSG00000198756) | collagen beta(1-O)galactosyltransferase 2 [Source:HGNC Symbol;Acc:HGNC:16790] | -1,96 |
| COLQ | [ENSG00000206561](https://www.ensembl.org/id/ENSG00000206561) | collagen like tail subunit of asymmetric acetylcholinesterase [Source:HGNC Symbol;Acc:HGNC:2226] | -1,97 |
| COMMD3-BMI1 | [ENSG00000269897](https://www.ensembl.org/id/ENSG00000269897) | COMMD3-BMI1 readthrough [Source:HGNC Symbol;Acc:HGNC:48326] | -7,82 |
| COMMD9 | [ENSG00000110442](https://www.ensembl.org/id/ENSG00000110442) | COMM domain containing 9 [Source:HGNC Symbol;Acc:HGNC:25014] | -1,47 |
| COMTD1 | [ENSG00000165644](https://www.ensembl.org/id/ENSG00000165644) | catechol-O-methyltransferase domain containing 1 [Source:HGNC Symbol;Acc:HGNC:26309] | -1,02 |
| COQ10A | [ENSG00000135469](https://www.ensembl.org/id/ENSG00000135469) | coenzyme Q10A [Source:HGNC Symbol;Acc:HGNC:26515] | -3,69 |
| COQ2 | [ENSG00000173085](https://www.ensembl.org/id/ENSG00000173085) | coenzyme Q2, polyprenyltransferase [Source:HGNC Symbol;Acc:HGNC:25223] | -2,08 |
| COQ3 | [ENSG00000132423](https://www.ensembl.org/id/ENSG00000132423) | coenzyme Q3, methyltransferase [Source:HGNC Symbol;Acc:HGNC:18175] | -1,65 |
| COQ5 | [ENSG00000110871](https://www.ensembl.org/id/ENSG00000110871) | coenzyme Q5, methyltransferase [Source:HGNC Symbol;Acc:HGNC:28722] | -1,78 |
| COQ6 | [ENSG00000119723](https://www.ensembl.org/id/ENSG00000119723) | coenzyme Q6, monooxygenase [Source:HGNC Symbol;Acc:HGNC:20233] | -1,02 |
| COQ7-DT | [ENSG00000261465](https://www.ensembl.org/id/ENSG00000261465) | COQ7 divergent transcript [Source:HGNC Symbol;Acc:HGNC:55362] | -5,40 |
| COQ8A | [ENSG00000163050](https://www.ensembl.org/id/ENSG00000163050) | coenzyme Q8A [Source:HGNC Symbol;Acc:HGNC:16812] | -4,79 |
| COQ9 | [ENSG00000088682](https://www.ensembl.org/id/ENSG00000088682) | coenzyme Q9 [Source:HGNC Symbol;Acc:HGNC:25302] | -2,35 |
| CORO6 | [ENSG00000167549](https://www.ensembl.org/id/ENSG00000167549) | coronin 6 [Source:HGNC Symbol;Acc:HGNC:21356] | -3,02 |
| COX10 | [ENSG00000006695](https://www.ensembl.org/id/ENSG00000006695) | cytochrome c oxidase assembly factor heme A:farnesyltransferase COX10 [Source:HGNC Symbol;Acc:HGNC:2260] | -1,91 |
| COX15 | [ENSG00000014919](https://www.ensembl.org/id/ENSG00000014919) | cytochrome c oxidase assembly homolog COX15 [Source:HGNC Symbol;Acc:HGNC:2263] | -1,05 |
| COX17 | [ENSG00000138495](https://www.ensembl.org/id/ENSG00000138495) | cytochrome c oxidase copper chaperone COX17 [Source:HGNC Symbol;Acc:HGNC:2264] | -1,59 |
| COX5A | [ENSG00000178741](https://www.ensembl.org/id/ENSG00000178741) | cytochrome c oxidase subunit 5A [Source:HGNC Symbol;Acc:HGNC:2267] | -2,87 |
| COX5B | [ENSG00000135940](https://www.ensembl.org/id/ENSG00000135940) | cytochrome c oxidase subunit 5B [Source:HGNC Symbol;Acc:HGNC:2269] | -1,75 |
| COX6A2 | [ENSG00000156885](https://www.ensembl.org/id/ENSG00000156885) | cytochrome c oxidase subunit 6A2 [Source:HGNC Symbol;Acc:HGNC:2279] | -10,68 |
| COX6B1 | [ENSG00000126267](https://www.ensembl.org/id/ENSG00000126267) | cytochrome c oxidase subunit 6B1 [Source:HGNC Symbol;Acc:HGNC:2280] | -1,47 |
| COX7A1 | [ENSG00000161281](https://www.ensembl.org/id/ENSG00000161281) | cytochrome c oxidase subunit 7A1 [Source:HGNC Symbol;Acc:HGNC:2287] | -2,23 |
| COX7B | [ENSG00000131174](https://www.ensembl.org/id/ENSG00000131174) | cytochrome c oxidase subunit 7B [Source:HGNC Symbol;Acc:HGNC:2291] | -1,83 |
| COX8A | [ENSG00000176340](https://www.ensembl.org/id/ENSG00000176340) | cytochrome c oxidase subunit 8A [Source:HGNC Symbol;Acc:HGNC:2294] | -1,47 |
| CPA3 | [ENSG00000163751](https://www.ensembl.org/id/ENSG00000163751) | carboxypeptidase A3 [Source:HGNC Symbol;Acc:HGNC:2298] | -1,91 |
| CPA5 | [ENSG00000158525](https://www.ensembl.org/id/ENSG00000158525) | carboxypeptidase A5 [Source:HGNC Symbol;Acc:HGNC:15722] | -3,44 |
| CPEB2 | [ENSG00000137449](https://www.ensembl.org/id/ENSG00000137449) | cytoplasmic polyadenylation element binding protein 2 [Source:HGNC Symbol;Acc:HGNC:21745] | 1,13 |
| CPEB3 | [ENSG00000107864](https://www.ensembl.org/id/ENSG00000107864) | cytoplasmic polyadenylation element binding protein 3 [Source:HGNC Symbol;Acc:HGNC:21746] | -1,79 |
| CPED1 | [ENSG00000106034](https://www.ensembl.org/id/ENSG00000106034) | cadherin like and PC-esterase domain containing 1 [Source:HGNC Symbol;Acc:HGNC:26159] | 1,14 |
| CPT1A | [ENSG00000110090](https://www.ensembl.org/id/ENSG00000110090) | carnitine palmitoyltransferase 1A [Source:HGNC Symbol;Acc:HGNC:2328] | 1,11 |
| CPT1B | [ENSG00000205560](https://www.ensembl.org/id/ENSG00000205560) | carnitine palmitoyltransferase 1B [Source:HGNC Symbol;Acc:HGNC:2329] | -5,28 |
| CPTP | [ENSG00000224051](https://www.ensembl.org/id/ENSG00000224051) | ceramide-1-phosphate transfer protein [Source:HGNC Symbol;Acc:HGNC:28116] | -1,66 |
| CPVL | [ENSG00000106066](https://www.ensembl.org/id/ENSG00000106066) | carboxypeptidase vitellogenic like [Source:HGNC Symbol;Acc:HGNC:14399] | -1,87 |
| CPXM1 | [ENSG00000088882](https://www.ensembl.org/id/ENSG00000088882) | carboxypeptidase X, M14 family member 1 [Source:HGNC Symbol;Acc:HGNC:15771] | -2,52 |
| CRAT | [ENSG00000095321](https://www.ensembl.org/id/ENSG00000095321) | carnitine O-acetyltransferase [Source:HGNC Symbol;Acc:HGNC:2342] | -2,37 |
| CREG2 | [ENSG00000175874](https://www.ensembl.org/id/ENSG00000175874) | cellular repressor of E1A stimulated genes 2 [Source:HGNC Symbol;Acc:HGNC:14272] | -6,01 |
| CRHR2 | [ENSG00000106113](https://www.ensembl.org/id/ENSG00000106113) | corticotropin releasing hormone receptor 2 [Source:HGNC Symbol;Acc:HGNC:2358] | -2,58 |
| CRISP3 | [ENSG00000096006](https://www.ensembl.org/id/ENSG00000096006) | cysteine rich secretory protein 3 [Source:HGNC Symbol;Acc:HGNC:16904] | 5,09 |
| CRTC3 | [ENSG00000140577](https://www.ensembl.org/id/ENSG00000140577) | CREB regulated transcription coactivator 3 [Source:HGNC Symbol;Acc:HGNC:26148] | 1,08 |
| CRX | [ENSG00000105392](https://www.ensembl.org/id/ENSG00000105392) | cone-rod homeobox [Source:HGNC Symbol;Acc:HGNC:2383] | -3,50 |
| CRYAB | [ENSG00000109846](https://www.ensembl.org/id/ENSG00000109846) | crystallin alpha B [Source:HGNC Symbol;Acc:HGNC:2389] | -2,26 |
| CRYBB3 | [ENSG00000100053](https://www.ensembl.org/id/ENSG00000100053) | crystallin beta B3 [Source:HGNC Symbol;Acc:HGNC:2400] | 5,91 |
| CRYBG2 | [ENSG00000176092](https://www.ensembl.org/id/ENSG00000176092) | crystallin beta-gamma domain containing 2 [Source:HGNC Symbol;Acc:HGNC:17295] | -6,85 |
| CS | [ENSG00000062485](https://www.ensembl.org/id/ENSG00000062485) | citrate synthase [Source:HGNC Symbol;Acc:HGNC:2422] | -1,67 |
| CSN2 | [ENSG00000135222](https://www.ensembl.org/id/ENSG00000135222) | casein beta [Source:HGNC Symbol;Acc:HGNC:2447] | -6,39 |
| CSNK1G2-AS1 | [ENSG00000180846](https://www.ensembl.org/id/ENSG00000180846) | CSNK1G2 antisense RNA 1 [Source:HGNC Symbol;Acc:HGNC:28604] | 5,20 |
| CSRP3 | [ENSG00000129170](https://www.ensembl.org/id/ENSG00000129170) | cysteine and glycine rich protein 3 [Source:HGNC Symbol;Acc:HGNC:2472] | -12,46 |
| CT69 | [ENSG00000231971](https://www.ensembl.org/id/ENSG00000231971) | cancer/testis associated transcript 69 [Source:HGNC Symbol;Acc:HGNC:37196] | -5,63 |
| CTAG2 | [ENSG00000126890](https://www.ensembl.org/id/ENSG00000126890) | cancer/testis antigen 2 [Source:HGNC Symbol;Acc:HGNC:2492] | 8,35 |
| CTAGE8 | [ENSG00000289604](https://www.ensembl.org/id/ENSG00000289604) | CTAGE family member 8 [Source:HGNC Symbol;Acc:HGNC:37294] | -3,81 |
| CTDNEP1 | [ENSG00000175826](https://www.ensembl.org/id/ENSG00000175826) | CTD nuclear envelope phosphatase 1 [Source:HGNC Symbol;Acc:HGNC:19085] | -1,89 |
| CTNNA3 | [ENSG00000183230](https://www.ensembl.org/id/ENSG00000183230) | catenin alpha 3 [Source:HGNC Symbol;Acc:HGNC:2511] | -1,57 |
| CTRB1 | [ENSG00000168925](https://www.ensembl.org/id/ENSG00000168925) | chymotrypsinogen B1 [Source:HGNC Symbol;Acc:HGNC:2521] | -3,30 |
| CTSLP2 | [ENSG00000290874](https://www.ensembl.org/id/ENSG00000290874) | cathepsin L pseudogene 2 [Source:NCBI gene (formerly Entrezgene);Acc:1517] | -4,73 |
| CTXND2 | [ENSG00000283324](https://www.ensembl.org/id/ENSG00000283324) | cortexin domain containing 2 [Source:HGNC Symbol;Acc:HGNC:53440] | -5,08 |
| CUL4A | [ENSG00000139842](https://www.ensembl.org/id/ENSG00000139842) | cullin 4A [Source:HGNC Symbol;Acc:HGNC:2554] | -1,43 |
| CUL5 | [ENSG00000166266](https://www.ensembl.org/id/ENSG00000166266) | cullin 5 [Source:HGNC Symbol;Acc:HGNC:2556] | -1,18 |
| CUTC | [ENSG00000119929](https://www.ensembl.org/id/ENSG00000119929) | cutC copper transporter [Source:HGNC Symbol;Acc:HGNC:24271] | -3,70 |
| CUX2 | [ENSG00000111249](https://www.ensembl.org/id/ENSG00000111249) | cut like homeobox 2 [Source:HGNC Symbol;Acc:HGNC:19347] | -4,88 |
| CX3CR1 | [ENSG00000168329](https://www.ensembl.org/id/ENSG00000168329) | C-X3-C motif chemokine receptor 1 [Source:HGNC Symbol;Acc:HGNC:2558] | -2,91 |
| CXCL11 | [ENSG00000169248](https://www.ensembl.org/id/ENSG00000169248) | C-X-C motif chemokine ligand 11 [Source:HGNC Symbol;Acc:HGNC:10638] | -3,24 |
| CXCL9 | [ENSG00000138755](https://www.ensembl.org/id/ENSG00000138755) | C-X-C motif chemokine ligand 9 [Source:HGNC Symbol;Acc:HGNC:7098] | -3,68 |
| CXCR6 | [ENSG00000172215](https://www.ensembl.org/id/ENSG00000172215) | C-X-C motif chemokine receptor 6 [Source:HGNC Symbol;Acc:HGNC:16647] | -2,56 |
| CYB5R1 | [ENSG00000159348](https://www.ensembl.org/id/ENSG00000159348) | cytochrome b5 reductase 1 [Source:HGNC Symbol;Acc:HGNC:13397] | -2,93 |
| CYC1 | [ENSG00000179091](https://www.ensembl.org/id/ENSG00000179091) | cytochrome c1 [Source:HGNC Symbol;Acc:HGNC:2579] | -2,02 |
| CYFIP2 | [ENSG00000055163](https://www.ensembl.org/id/ENSG00000055163) | cytoplasmic FMR1 interacting protein 2 [Source:HGNC Symbol;Acc:HGNC:13760] | 1,71 |
| CYP11A1 | [ENSG00000140459](https://www.ensembl.org/id/ENSG00000140459) | cytochrome P450 family 11 subfamily A member 1 [Source:HGNC Symbol;Acc:HGNC:2590] | -3,75 |
| CYP2J2 | [ENSG00000134716](https://www.ensembl.org/id/ENSG00000134716) | cytochrome P450 family 2 subfamily J member 2 [Source:HGNC Symbol;Acc:HGNC:2634] | -8,10 |
| CYP2U1-AS1 | [ENSG00000245293](https://www.ensembl.org/id/ENSG00000245293) | CYP2U1 and SGMS2 antisense RNA 1 [Source:HGNC Symbol;Acc:HGNC:54817] | -2,05 |
| CYP4B1 | [ENSG00000142973](https://www.ensembl.org/id/ENSG00000142973) | cytochrome P450 family 4 subfamily B member 1 [Source:HGNC Symbol;Acc:HGNC:2644] | -1,97 |
| CYP4F26P | [ENSG00000226562](https://www.ensembl.org/id/ENSG00000226562) | cytochrome P450 family 4 subfamily F member 26, pseudogene [Source:HGNC Symbol;Acc:HGNC:39948] | -3,32 |
| CYP4F29P | [ENSG00000290541](https://www.ensembl.org/id/ENSG00000290541) | cytochrome P450 family 4 subfamily F member 29, pseudogene [Source:NCBI gene (formerly Entrezgene);Acc:54055] | 3,79 |
| CYP51A1 | [ENSG00000001630](https://www.ensembl.org/id/ENSG00000001630) | cytochrome P450 family 51 subfamily A member 1 [Source:HGNC Symbol;Acc:HGNC:2649] | 0,96 |
| DAB1 | [ENSG00000173406](https://www.ensembl.org/id/ENSG00000173406) | DAB adaptor protein 1 [Source:HGNC Symbol;Acc:HGNC:2661] | 2,07 |
| DANCR | [ENSG00000226950](https://www.ensembl.org/id/ENSG00000226950) | differentiation antagonizing non-protein coding RNA [Source:HGNC Symbol;Acc:HGNC:28964] | -1,09 |
| DAPK2 | [ENSG00000035664](https://www.ensembl.org/id/ENSG00000035664) | death associated protein kinase 2 [Source:HGNC Symbol;Acc:HGNC:2675] | -3,85 |
| DAPL1 | [ENSG00000163331](https://www.ensembl.org/id/ENSG00000163331) | death associated protein like 1 [Source:HGNC Symbol;Acc:HGNC:21490] | -2,55 |
| DBI | [ENSG00000155368](https://www.ensembl.org/id/ENSG00000155368) | diazepam binding inhibitor, acyl-CoA binding protein [Source:HGNC Symbol;Acc:HGNC:2690] | -1,22 |
| DBNDD1 | [ENSG00000003249](https://www.ensembl.org/id/ENSG00000003249) | dysbindin domain containing 1 [Source:HGNC Symbol;Acc:HGNC:28455] | -4,23 |
| DBT | [ENSG00000137992](https://www.ensembl.org/id/ENSG00000137992) | dihydrolipoamide branched chain transacylase E2 [Source:HGNC Symbol;Acc:HGNC:2698] | -1,02 |
| DCAF11 | [ENSG00000100897](https://www.ensembl.org/id/ENSG00000100897) | DDB1 and CUL4 associated factor 11 [Source:HGNC Symbol;Acc:HGNC:20258] | -1,05 |
| DCAF6 | [ENSG00000143164](https://www.ensembl.org/id/ENSG00000143164) | DDB1 and CUL4 associated factor 6 [Source:HGNC Symbol;Acc:HGNC:30002] | -3,02 |
| DCDC2C | [ENSG00000214866](https://www.ensembl.org/id/ENSG00000214866) | doublecortin domain containing 2C [Source:HGNC Symbol;Acc:HGNC:32696] | -3,10 |
| DCHS2 | [ENSG00000197410](https://www.ensembl.org/id/ENSG00000197410) | dachsous cadherin-related 2 [Source:HGNC Symbol;Acc:HGNC:23111] | -3,27 |
| DCLK3 | [ENSG00000163673](https://www.ensembl.org/id/ENSG00000163673) | doublecortin like kinase 3 [Source:HGNC Symbol;Acc:HGNC:19005] | -2,95 |
| DCUN1D2 | [ENSG00000150401](https://www.ensembl.org/id/ENSG00000150401) | defective in cullin neddylation 1 domain containing 2 [Source:HGNC Symbol;Acc:HGNC:20328] | -2,50 |
| DCXR | [ENSG00000169738](https://www.ensembl.org/id/ENSG00000169738) | dicarbonyl and L-xylulose reductase [Source:HGNC Symbol;Acc:HGNC:18985] | -1,70 |
| DCXR-DT | [ENSG00000264569](https://www.ensembl.org/id/ENSG00000264569) | DCXR divergent transcript [Source:HGNC Symbol;Acc:HGNC:54083] | -3,75 |
| DDI2 | [ENSG00000197312](https://www.ensembl.org/id/ENSG00000197312) | DNA damage inducible 1 homolog 2 [Source:HGNC Symbol;Acc:HGNC:24578] | -1,01 |
| DDIT4L | [ENSG00000145358](https://www.ensembl.org/id/ENSG00000145358) | DNA damage inducible transcript 4 like [Source:HGNC Symbol;Acc:HGNC:30555] | -3,44 |
| DDN | [ENSG00000181418](https://www.ensembl.org/id/ENSG00000181418) | dendrin [Source:HGNC Symbol;Acc:HGNC:24458] | -9,16 |
| DDN-AS1 | [ENSG00000257913](https://www.ensembl.org/id/ENSG00000257913) | DDN and PRKAG1 antisense RNA 1 [Source:HGNC Symbol;Acc:HGNC:53464] | -4,22 |
| DDX11L16_1 | [ENSG00000290824](https://www.ensembl.org/id/ENSG00000290824) | DEAD/H-box helicase 11 like 16 (pseudogene) [Source:NCBI gene (formerly Entrezgene);Acc:727856] | -3,63 |
| DDX11L16_2 | [ENSG00000290355](https://www.ensembl.org/id/ENSG00000290355) | DEAD/H-box helicase 11 like 16 (pseudogene) [Source:NCBI gene (formerly Entrezgene);Acc:727856] | -5,48 |
| DECR1 | [ENSG00000104325](https://www.ensembl.org/id/ENSG00000104325) | 2,4-dienoyl-CoA reductase 1 [Source:HGNC Symbol;Acc:HGNC:2753] | -1,65 |
| DEGS1 | [ENSG00000143753](https://www.ensembl.org/id/ENSG00000143753) | delta 4-desaturase, sphingolipid 1 [Source:HGNC Symbol;Acc:HGNC:13709] | 0,92 |
| DENND2C | [ENSG00000175984](https://www.ensembl.org/id/ENSG00000175984) | DENN domain containing 2C [Source:HGNC Symbol;Acc:HGNC:24748] | -1,98 |
| DENND4B | [ENSG00000198837](https://www.ensembl.org/id/ENSG00000198837) | DENN domain containing 4B [Source:HGNC Symbol;Acc:HGNC:29044] | -1,07 |
| DEPDC7 | [ENSG00000121690](https://www.ensembl.org/id/ENSG00000121690) | DEP domain containing 7 [Source:HGNC Symbol;Acc:HGNC:29899] | -3,37 |
| DEPTOR | [ENSG00000155792](https://www.ensembl.org/id/ENSG00000155792) | DEP domain containing MTOR interacting protein [Source:HGNC Symbol;Acc:HGNC:22953] | -3,74 |
| DEPTOR-AS1 | [ENSG00000245330](https://www.ensembl.org/id/ENSG00000245330) | DEPTOR antisense RNA 1 [Source:HGNC Symbol;Acc:HGNC:55602] | -5,43 |
| DES | [ENSG00000175084](https://www.ensembl.org/id/ENSG00000175084) | desmin [Source:HGNC Symbol;Acc:HGNC:2770] | -2,49 |
| DEXI | [ENSG00000182108](https://www.ensembl.org/id/ENSG00000182108) | Dexi homolog [Source:HGNC Symbol;Acc:HGNC:13267] | -0,90 |
| DGCR6 | [ENSG00000183628](https://www.ensembl.org/id/ENSG00000183628) | DiGeorge syndrome critical region gene 6 [Source:HGNC Symbol;Acc:HGNC:2846] | -4,33 |
| DGKB | [ENSG00000136267](https://www.ensembl.org/id/ENSG00000136267) | diacylglycerol kinase beta [Source:HGNC Symbol;Acc:HGNC:2850] | -3,81 |
| DGKK | [ENSG00000274588](https://www.ensembl.org/id/ENSG00000274588) | diacylglycerol kinase kappa [Source:HGNC Symbol;Acc:HGNC:32395] | -5,40 |
| DGLUCY | [ENSG00000133943](https://www.ensembl.org/id/ENSG00000133943) | D-glutamate cyclase [Source:HGNC Symbol;Acc:HGNC:20498] | -2,00 |
| DHCR24 | [ENSG00000116133](https://www.ensembl.org/id/ENSG00000116133) | 24-dehydrocholesterol reductase [Source:HGNC Symbol;Acc:HGNC:2859] | -1,07 |
| DHRS7C | [ENSG00000184544](https://www.ensembl.org/id/ENSG00000184544) | dehydrogenase/reductase 7C [Source:HGNC Symbol;Acc:HGNC:32423] | -9,24 |
| DHTKD1 | [ENSG00000181192](https://www.ensembl.org/id/ENSG00000181192) | dehydrogenase E1 and transketolase domain containing 1 [Source:HGNC Symbol;Acc:HGNC:23537] | -1,50 |
| DIO3 | [ENSG00000197406](https://www.ensembl.org/id/ENSG00000197406) | iodothyronine deiodinase 3 [Source:HGNC Symbol;Acc:HGNC:2885] | -2,03 |
| DIRAS1 | [ENSG00000176490](https://www.ensembl.org/id/ENSG00000176490) | DIRAS family GTPase 1 [Source:HGNC Symbol;Acc:HGNC:19127] | -4,08 |
| DIS3L | [ENSG00000166938](https://www.ensembl.org/id/ENSG00000166938) | DIS3 like exosome 3'-5' exoribonuclease [Source:HGNC Symbol;Acc:HGNC:28698] | -1,24 |
| DKK2 | [ENSG00000155011](https://www.ensembl.org/id/ENSG00000155011) | dickkopf WNT signaling pathway inhibitor 2 [Source:HGNC Symbol;Acc:HGNC:2892] | -2,88 |
| DLAT | [ENSG00000150768](https://www.ensembl.org/id/ENSG00000150768) | dihydrolipoamide S-acetyltransferase [Source:HGNC Symbol;Acc:HGNC:2896] | -1,77 |
| DLD | [ENSG00000091140](https://www.ensembl.org/id/ENSG00000091140) | dihydrolipoamide dehydrogenase [Source:HGNC Symbol;Acc:HGNC:2898] | -1,80 |
| DLK1 | [ENSG00000185559](https://www.ensembl.org/id/ENSG00000185559) | delta like non-canonical Notch ligand 1 [Source:HGNC Symbol;Acc:HGNC:2907] | -9,13 |
| DLX6-AS1 | [ENSG00000231764](https://www.ensembl.org/id/ENSG00000231764) | DLX6 antisense RNA 1 [Source:HGNC Symbol;Acc:HGNC:37151] | 2,98 |
| DMAC2L | [ENSG00000125375](https://www.ensembl.org/id/ENSG00000125375) | distal membrane arm assembly component 2 like [Source:HGNC Symbol;Acc:HGNC:18799] | -1,63 |
| DMGDH | [ENSG00000132837](https://www.ensembl.org/id/ENSG00000132837) | dimethylglycine dehydrogenase [Source:HGNC Symbol;Acc:HGNC:24475] | -1,66 |
| DMRTC1B | [ENSG00000184911](https://www.ensembl.org/id/ENSG00000184911) | DMRT like family C1B [Source:HGNC Symbol;Acc:HGNC:31686] | -6,68 |
| DNAH14 | [ENSG00000185842](https://www.ensembl.org/id/ENSG00000185842) | dynein axonemal heavy chain 14 [Source:HGNC Symbol;Acc:HGNC:2945] | 2,69 |
| DNAH3 | [ENSG00000158486](https://www.ensembl.org/id/ENSG00000158486) | dynein axonemal heavy chain 3 [Source:HGNC Symbol;Acc:HGNC:2949] | -3,42 |
| DNAH8-AS1 | [ENSG00000231150](https://www.ensembl.org/id/ENSG00000231150) | DNAH8 antisense RNA 1 [Source:HGNC Symbol;Acc:HGNC:40188] | -5,47 |
| DNAJA3 | [ENSG00000103423](https://www.ensembl.org/id/ENSG00000103423) | DnaJ heat shock protein family (Hsp40) member A3 [Source:HGNC Symbol;Acc:HGNC:11808] | -1,14 |
| DNAJA4 | [ENSG00000140403](https://www.ensembl.org/id/ENSG00000140403) | DnaJ heat shock protein family (Hsp40) member A4 [Source:HGNC Symbol;Acc:HGNC:14885] | -2,53 |
| DNAJC12 | [ENSG00000108176](https://www.ensembl.org/id/ENSG00000108176) | DnaJ heat shock protein family (Hsp40) member C12 [Source:HGNC Symbol;Acc:HGNC:28908] | -2,46 |
| DNAJC19 | [ENSG00000205981](https://www.ensembl.org/id/ENSG00000205981) | DnaJ heat shock protein family (Hsp40) member C19 [Source:HGNC Symbol;Acc:HGNC:30528] | -1,05 |
| DNAJC4 | [ENSG00000110011](https://www.ensembl.org/id/ENSG00000110011) | DnaJ heat shock protein family (Hsp40) member C4 [Source:HGNC Symbol;Acc:HGNC:5271] | -0,90 |
| DNAJC5B | [ENSG00000147570](https://www.ensembl.org/id/ENSG00000147570) | DnaJ heat shock protein family (Hsp40) member C5 beta [Source:HGNC Symbol;Acc:HGNC:24138] | -3,69 |
| DNASE1L1 | [ENSG00000013563](https://www.ensembl.org/id/ENSG00000013563) | deoxyribonuclease 1 like 1 [Source:HGNC Symbol;Acc:HGNC:2957] | -2,64 |
| DNASE2B | [ENSG00000137976](https://www.ensembl.org/id/ENSG00000137976) | deoxyribonuclease 2 beta [Source:HGNC Symbol;Acc:HGNC:28875] | -6,15 |
| DND1 | [ENSG00000256453](https://www.ensembl.org/id/ENSG00000256453) | DND microRNA-mediated repression inhibitor 1 [Source:HGNC Symbol;Acc:HGNC:23799] | 4,98 |
| DNER | [ENSG00000187957](https://www.ensembl.org/id/ENSG00000187957) | delta/notch like EGF repeat containing [Source:HGNC Symbol;Acc:HGNC:24456] | -5,48 |
| DNM3OS | [ENSG00000230630](https://www.ensembl.org/id/ENSG00000230630) | DNM3 opposite strand/antisense RNA [Source:HGNC Symbol;Acc:HGNC:41228] | 1,34 |
| DNPH1 | [ENSG00000112667](https://www.ensembl.org/id/ENSG00000112667) | 2'-deoxynucleoside 5'-phosphate N-hydrolase 1 [Source:HGNC Symbol;Acc:HGNC:21218] | -1,16 |
| DOCK11 | [ENSG00000147251](https://www.ensembl.org/id/ENSG00000147251) | dedicator of cytokinesis 11 [Source:HGNC Symbol;Acc:HGNC:23483] | 0,95 |
| DOK5 | [ENSG00000101134](https://www.ensembl.org/id/ENSG00000101134) | docking protein 5 [Source:HGNC Symbol;Acc:HGNC:16173] | -3,54 |
| DOK7 | [ENSG00000175920](https://www.ensembl.org/id/ENSG00000175920) | docking protein 7 [Source:HGNC Symbol;Acc:HGNC:26594] | -1,81 |
| DPF3 | [ENSG00000205683](https://www.ensembl.org/id/ENSG00000205683) | double PHD fingers 3 [Source:HGNC Symbol;Acc:HGNC:17427] | -1,32 |
| DPH1-AS1 | [ENSG00000263050](https://www.ensembl.org/id/ENSG00000263050) | DPH1 antisense RNA 1 [Source:HGNC Symbol;Acc:HGNC:56040] | 5,31 |
| DPH2 | [ENSG00000132768](https://www.ensembl.org/id/ENSG00000132768) | diphthamide biosynthesis 2 [Source:HGNC Symbol;Acc:HGNC:3004] | -1,01 |
| DPY19L2 | [ENSG00000177990](https://www.ensembl.org/id/ENSG00000177990) | dpy-19 like 2 [Source:HGNC Symbol;Acc:HGNC:19414] | 1,32 |
| DPYSL5 | [ENSG00000157851](https://www.ensembl.org/id/ENSG00000157851) | dihydropyrimidinase like 5 [Source:HGNC Symbol;Acc:HGNC:20637] | -5,70 |
| DRD2 | [ENSG00000149295](https://www.ensembl.org/id/ENSG00000149295) | dopamine receptor D2 [Source:HGNC Symbol;Acc:HGNC:3023] | -2,55 |
| DRP2 | [ENSG00000102385](https://www.ensembl.org/id/ENSG00000102385) | dystrophin related protein 2 [Source:HGNC Symbol;Acc:HGNC:3032] | -1,58 |
| DSC3 | [ENSG00000134762](https://www.ensembl.org/id/ENSG00000134762) | desmocollin 3 [Source:HGNC Symbol;Acc:HGNC:3037] | 2,47 |
| DSCR8 | [ENSG00000198054](https://www.ensembl.org/id/ENSG00000198054) | Down syndrome critical region 8 [Source:HGNC Symbol;Acc:HGNC:16707] | -7,43 |
| DUSP10 | [ENSG00000143507](https://www.ensembl.org/id/ENSG00000143507) | dual specificity phosphatase 10 [Source:HGNC Symbol;Acc:HGNC:3065] | -1,73 |
| DUSP13 | [ENSG00000079393](https://www.ensembl.org/id/ENSG00000079393) | dual specificity phosphatase 13 [Source:HGNC Symbol;Acc:HGNC:19681] | -10,86 |
| DUSP26 | [ENSG00000133878](https://www.ensembl.org/id/ENSG00000133878) | dual specificity phosphatase 26 [Source:HGNC Symbol;Acc:HGNC:28161] | -2,43 |
| DUSP28 | [ENSG00000188542](https://www.ensembl.org/id/ENSG00000188542) | dual specificity phosphatase 28 [Source:HGNC Symbol;Acc:HGNC:33237] | -1,72 |
| DUSP29 | [ENSG00000188716](https://www.ensembl.org/id/ENSG00000188716) | dual specificity phosphatase 29 [Source:HGNC Symbol;Acc:HGNC:23481] | -12,90 |
| DUSP4 | [ENSG00000120875](https://www.ensembl.org/id/ENSG00000120875) | dual specificity phosphatase 4 [Source:HGNC Symbol;Acc:HGNC:3070] | -1,39 |
| DUSP8 | [ENSG00000184545](https://www.ensembl.org/id/ENSG00000184545) | dual specificity phosphatase 8 [Source:HGNC Symbol;Acc:HGNC:3074] | 1,30 |
| DVL1 | [ENSG00000107404](https://www.ensembl.org/id/ENSG00000107404) | dishevelled segment polarity protein 1 [Source:HGNC Symbol;Acc:HGNC:3084] | -1,98 |
| DYRK1B | [ENSG00000105204](https://www.ensembl.org/id/ENSG00000105204) | dual specificity tyrosine phosphorylation regulated kinase 1B [Source:HGNC Symbol;Acc:HGNC:3092] | -2,93 |
| E2F8 | [ENSG00000129173](https://www.ensembl.org/id/ENSG00000129173) | E2F transcription factor 8 [Source:HGNC Symbol;Acc:HGNC:24727] | -6,77 |
| EARS2 | [ENSG00000103356](https://www.ensembl.org/id/ENSG00000103356) | glutamyl-tRNA synthetase 2, mitochondrial [Source:HGNC Symbol;Acc:HGNC:29419] | -1,23 |
| EBF1 | [ENSG00000164330](https://www.ensembl.org/id/ENSG00000164330) | EBF transcription factor 1 [Source:HGNC Symbol;Acc:HGNC:3126] | 0,95 |
| ECE2 | [ENSG00000145194](https://www.ensembl.org/id/ENSG00000145194) | endothelin converting enzyme 2 [Source:HGNC Symbol;Acc:HGNC:13275] | -4,91 |
| ECH1 | [ENSG00000104823](https://www.ensembl.org/id/ENSG00000104823) | enoyl-CoA hydratase 1 [Source:HGNC Symbol;Acc:HGNC:3149] | -2,02 |
| ECHDC3 | [ENSG00000134463](https://www.ensembl.org/id/ENSG00000134463) | enoyl-CoA hydratase domain containing 3 [Source:HGNC Symbol;Acc:HGNC:23489] | -2,00 |
| ECI1 | [ENSG00000167969](https://www.ensembl.org/id/ENSG00000167969) | enoyl-CoA delta isomerase 1 [Source:HGNC Symbol;Acc:HGNC:2703] | -1,38 |
| ECI2 | [ENSG00000198721](https://www.ensembl.org/id/ENSG00000198721) | enoyl-CoA delta isomerase 2 [Source:HGNC Symbol;Acc:HGNC:14601] | -1,69 |
| ECSIT | [ENSG00000130159](https://www.ensembl.org/id/ENSG00000130159) | ECSIT signaling integrator [Source:HGNC Symbol;Acc:HGNC:29548] | -1,38 |
| EDN3 | [ENSG00000124205](https://www.ensembl.org/id/ENSG00000124205) | endothelin 3 [Source:HGNC Symbol;Acc:HGNC:3178] | -3,48 |
| EDNRA | [ENSG00000151617](https://www.ensembl.org/id/ENSG00000151617) | endothelin receptor type A [Source:HGNC Symbol;Acc:HGNC:3179] | 1,44 |
| EDNRB | [ENSG00000136160](https://www.ensembl.org/id/ENSG00000136160) | endothelin receptor type B [Source:HGNC Symbol;Acc:HGNC:3180] | 1,54 |
| EDRF1-AS1 | [ENSG00000236991](https://www.ensembl.org/id/ENSG00000236991) | EDRF1 antisense RNA 1 [Source:HGNC Symbol;Acc:HGNC:49501] | 5,08 |
| EEF1A2 | [ENSG00000101210](https://www.ensembl.org/id/ENSG00000101210) | eukaryotic translation elongation factor 1 alpha 2 [Source:HGNC Symbol;Acc:HGNC:3192] | -10,10 |
| EEF1B2 | [ENSG00000114942](https://www.ensembl.org/id/ENSG00000114942) | eukaryotic translation elongation factor 1 beta 2 [Source:HGNC Symbol;Acc:HGNC:3208] | -1,27 |
| EFCAB2 | [ENSG00000203666](https://www.ensembl.org/id/ENSG00000203666) | EF-hand calcium binding domain 2 [Source:HGNC Symbol;Acc:HGNC:28166] | -1,60 |
| EFNB3 | [ENSG00000108947](https://www.ensembl.org/id/ENSG00000108947) | ephrin B3 [Source:HGNC Symbol;Acc:HGNC:3228] | -3,46 |
| EFR3B | [ENSG00000084710](https://www.ensembl.org/id/ENSG00000084710) | EFR3 homolog B [Source:HGNC Symbol;Acc:HGNC:29155] | -3,09 |
| EGF | [ENSG00000138798](https://www.ensembl.org/id/ENSG00000138798) | epidermal growth factor [Source:HGNC Symbol;Acc:HGNC:3229] | -5,87 |
| EGLN1 | [ENSG00000135766](https://www.ensembl.org/id/ENSG00000135766) | egl-9 family hypoxia inducible factor 1 [Source:HGNC Symbol;Acc:HGNC:1232] | -1,65 |
| EHD1 | [ENSG00000110047](https://www.ensembl.org/id/ENSG00000110047) | EH domain containing 1 [Source:HGNC Symbol;Acc:HGNC:3242] | 1,18 |
| EHD4-AS1 | [ENSG00000259883](https://www.ensembl.org/id/ENSG00000259883) | EHD4 antisense RNA 1 [Source:HGNC Symbol;Acc:HGNC:51418] | -4,01 |
| EIF1AY | [ENSG00000198692](https://www.ensembl.org/id/ENSG00000198692) | eukaryotic translation initiation factor 1A Y-linked [Source:HGNC Symbol;Acc:HGNC:3252] | -2,11 |
| EIF2B3 | [ENSG00000070785](https://www.ensembl.org/id/ENSG00000070785) | eukaryotic translation initiation factor 2B subunit gamma [Source:HGNC Symbol;Acc:HGNC:3259] | -1,18 |
| EIF3J | [ENSG00000104131](https://www.ensembl.org/id/ENSG00000104131) | eukaryotic translation initiation factor 3 subunit J [Source:HGNC Symbol;Acc:HGNC:3270] | -1,03 |
| EIF4A3 | [ENSG00000141543](https://www.ensembl.org/id/ENSG00000141543) | eukaryotic translation initiation factor 4A3 [Source:HGNC Symbol;Acc:HGNC:18683] | 1,61 |
| ELAVL4 | [ENSG00000162374](https://www.ensembl.org/id/ENSG00000162374) | ELAV like RNA binding protein 4 [Source:HGNC Symbol;Acc:HGNC:3315] | -5,72 |
| ELMOD1 | [ENSG00000110675](https://www.ensembl.org/id/ENSG00000110675) | ELMO domain containing 1 [Source:HGNC Symbol;Acc:HGNC:25334] | -3,78 |
| EMC6 | [ENSG00000127774](https://www.ensembl.org/id/ENSG00000127774) | ER membrane protein complex subunit 6 [Source:HGNC Symbol;Acc:HGNC:28430] | -1,71 |
| EMC9 | [ENSG00000100908](https://www.ensembl.org/id/ENSG00000100908) | ER membrane protein complex subunit 9 [Source:HGNC Symbol;Acc:HGNC:20273] | -1,24 |
| EML6 | [ENSG00000214595](https://www.ensembl.org/id/ENSG00000214595) | EMAP like 6 [Source:HGNC Symbol;Acc:HGNC:35412] | -2,36 |
| EN1 | [ENSG00000163064](https://www.ensembl.org/id/ENSG00000163064) | engrailed homeobox 1 [Source:HGNC Symbol;Acc:HGNC:3342] | -2,73 |
| ENC1 | [ENSG00000171617](https://www.ensembl.org/id/ENSG00000171617) | ectodermal-neural cortex 1 [Source:HGNC Symbol;Acc:HGNC:3345] | -2,13 |
| ENDOG | [ENSG00000167136](https://www.ensembl.org/id/ENSG00000167136) | endonuclease G [Source:HGNC Symbol;Acc:HGNC:3346] | -2,63 |
| ENO3 | [ENSG00000108515](https://www.ensembl.org/id/ENSG00000108515) | enolase 3 [Source:HGNC Symbol;Acc:HGNC:3354] | -9,40 |
| ENTPD1 | [ENSG00000138185](https://www.ensembl.org/id/ENSG00000138185) | ectonucleoside triphosphate diphosphohydrolase 1 [Source:HGNC Symbol;Acc:HGNC:3363] | 1,19 |
| EPB41L1-AS1 | [ENSG00000232406](https://www.ensembl.org/id/ENSG00000232406) | EPB41L1 antisense RNA 1 [Source:HGNC Symbol;Acc:HGNC:55728] | -3,23 |
| EPB42 | [ENSG00000166947](https://www.ensembl.org/id/ENSG00000166947) | erythrocyte membrane protein band 4.2 [Source:HGNC Symbol;Acc:HGNC:3381] | 3,32 |
| EPHA6 | [ENSG00000080224](https://www.ensembl.org/id/ENSG00000080224) | EPH receptor A6 [Source:HGNC Symbol;Acc:HGNC:19296] | 2,52 |
| EPHX3 | [ENSG00000105131](https://www.ensembl.org/id/ENSG00000105131) | epoxide hydrolase 3 [Source:HGNC Symbol;Acc:HGNC:23760] | 1,94 |
| EPM2A | [ENSG00000112425](https://www.ensembl.org/id/ENSG00000112425) | EPM2A glucan phosphatase, laforin [Source:HGNC Symbol;Acc:HGNC:3413] | -1,49 |
| ERBB3 | [ENSG00000065361](https://www.ensembl.org/id/ENSG00000065361) | erb-b2 receptor tyrosine kinase 3 [Source:HGNC Symbol;Acc:HGNC:3431] | -2,04 |
| ERICH5 | [ENSG00000177459](https://www.ensembl.org/id/ENSG00000177459) | glutamate rich 5 [Source:HGNC Symbol;Acc:HGNC:26823] | -5,11 |
| ESM1 | [ENSG00000164283](https://www.ensembl.org/id/ENSG00000164283) | endothelial cell specific molecule 1 [Source:HGNC Symbol;Acc:HGNC:3466] | 2,36 |
| ESPNL | [ENSG00000144488](https://www.ensembl.org/id/ENSG00000144488) | espin like [Source:HGNC Symbol;Acc:HGNC:27937] | -2,43 |
| ESR1 | [ENSG00000091831](https://www.ensembl.org/id/ENSG00000091831) | estrogen receptor 1 [Source:HGNC Symbol;Acc:HGNC:3467] | 0,95 |
| ESRRA | [ENSG00000173153](https://www.ensembl.org/id/ENSG00000173153) | estrogen related receptor alpha [Source:HGNC Symbol;Acc:HGNC:3471] | -2,63 |
| ESRRB | [ENSG00000119715](https://www.ensembl.org/id/ENSG00000119715) | estrogen related receptor beta [Source:HGNC Symbol;Acc:HGNC:3473] | -3,78 |
| ESRRG | [ENSG00000196482](https://www.ensembl.org/id/ENSG00000196482) | estrogen related receptor gamma [Source:HGNC Symbol;Acc:HGNC:3474] | -8,69 |
| ETFA | [ENSG00000140374](https://www.ensembl.org/id/ENSG00000140374) | electron transfer flavoprotein subunit alpha [Source:HGNC Symbol;Acc:HGNC:3481] | -1,76 |
| ETFB | [ENSG00000105379](https://www.ensembl.org/id/ENSG00000105379) | electron transfer flavoprotein subunit beta [Source:HGNC Symbol;Acc:HGNC:3482] | -1,18 |
| ETFDH | [ENSG00000171503](https://www.ensembl.org/id/ENSG00000171503) | electron transfer flavoprotein dehydrogenase [Source:HGNC Symbol;Acc:HGNC:3483] | -1,79 |
| ETFRF1 | [ENSG00000205707](https://www.ensembl.org/id/ENSG00000205707) | electron transfer flavoprotein regulatory factor 1 [Source:HGNC Symbol;Acc:HGNC:27052] | -0,89 |
| ETV4 | [ENSG00000175832](https://www.ensembl.org/id/ENSG00000175832) | ETS variant transcription factor 4 [Source:HGNC Symbol;Acc:HGNC:3493] | -3,23 |
| ETV5 | [ENSG00000244405](https://www.ensembl.org/id/ENSG00000244405) | ETS variant transcription factor 5 [Source:HGNC Symbol;Acc:HGNC:3494] | -1,54 |
| ETV6 | [ENSG00000139083](https://www.ensembl.org/id/ENSG00000139083) | ETS variant transcription factor 6 [Source:HGNC Symbol;Acc:HGNC:3495] | 1,28 |
| EXOC3-AS1 | [ENSG00000221990](https://www.ensembl.org/id/ENSG00000221990) | EXOC3 antisense RNA 1 [Source:HGNC Symbol;Acc:HGNC:25175] | -1,42 |
| EXOC6 | [ENSG00000138190](https://www.ensembl.org/id/ENSG00000138190) | exocyst complex component 6 [Source:HGNC Symbol;Acc:HGNC:23196] | -1,39 |
| EXOSC7 | [ENSG00000075914](https://www.ensembl.org/id/ENSG00000075914) | exosome component 7 [Source:HGNC Symbol;Acc:HGNC:28112] | -1,75 |
| EXTL1 | [ENSG00000158008](https://www.ensembl.org/id/ENSG00000158008) | exostosin like glycosyltransferase 1 [Source:HGNC Symbol;Acc:HGNC:3515] | -6,83 |
| EYA1 | [ENSG00000104313](https://www.ensembl.org/id/ENSG00000104313) | EYA transcriptional coactivator and phosphatase 1 [Source:HGNC Symbol;Acc:HGNC:3519] | -3,93 |
| EYA4 | [ENSG00000112319](https://www.ensembl.org/id/ENSG00000112319) | EYA transcriptional coactivator and phosphatase 4 [Source:HGNC Symbol;Acc:HGNC:3522] | -3,45 |
| F2RL2 | [ENSG00000164220](https://www.ensembl.org/id/ENSG00000164220) | coagulation factor II thrombin receptor like 2 [Source:HGNC Symbol;Acc:HGNC:3539] | -4,15 |
| F8 | [ENSG00000185010](https://www.ensembl.org/id/ENSG00000185010) | coagulation factor VIII [Source:HGNC Symbol;Acc:HGNC:3546] | -1,43 |
| F8A3 | [ENSG00000277150](https://www.ensembl.org/id/ENSG00000277150) | coagulation factor VIII associated 3 [Source:HGNC Symbol;Acc:HGNC:31850] | -2,94 |
| FABP3 | [ENSG00000121769](https://www.ensembl.org/id/ENSG00000121769) | fatty acid binding protein 3 [Source:HGNC Symbol;Acc:HGNC:3557] | -2,04 |
| FABP7 | [ENSG00000164434](https://www.ensembl.org/id/ENSG00000164434) | fatty acid binding protein 7 [Source:HGNC Symbol;Acc:HGNC:3562] | -10,29 |
| FADS1 | [ENSG00000149485](https://www.ensembl.org/id/ENSG00000149485) | fatty acid desaturase 1 [Source:HGNC Symbol;Acc:HGNC:3574] | 1,26 |
| FADS2 | [ENSG00000134824](https://www.ensembl.org/id/ENSG00000134824) | fatty acid desaturase 2 [Source:HGNC Symbol;Acc:HGNC:3575] | 0,96 |
| FAM117A | [ENSG00000121104](https://www.ensembl.org/id/ENSG00000121104) | family with sequence similarity 117 member A [Source:HGNC Symbol;Acc:HGNC:24179] | 1,01 |
| FAM135B | [ENSG00000147724](https://www.ensembl.org/id/ENSG00000147724) | family with sequence similarity 135 member B [Source:HGNC Symbol;Acc:HGNC:28029] | -4,93 |
| FAM151A | [ENSG00000162391](https://www.ensembl.org/id/ENSG00000162391) | family with sequence similarity 151 member A [Source:HGNC Symbol;Acc:HGNC:25032] | -5,03 |
| FAM166B | [ENSG00000215187](https://www.ensembl.org/id/ENSG00000215187) | family with sequence similarity 166 member B [Source:HGNC Symbol;Acc:HGNC:34242] | -2,70 |
| FAM177B | [ENSG00000197520](https://www.ensembl.org/id/ENSG00000197520) | family with sequence similarity 177 member B [Source:HGNC Symbol;Acc:HGNC:34395] | -5,89 |
| FAM184B | [ENSG00000047662](https://www.ensembl.org/id/ENSG00000047662) | family with sequence similarity 184 member B [Source:HGNC Symbol;Acc:HGNC:29235] | -9,05 |
| FAM210A | [ENSG00000177150](https://www.ensembl.org/id/ENSG00000177150) | family with sequence similarity 210 member A [Source:HGNC Symbol;Acc:HGNC:28346] | -1,56 |
| FAM220A | [ENSG00000178397](https://www.ensembl.org/id/ENSG00000178397) | family with sequence similarity 220 member A [Source:HGNC Symbol;Acc:HGNC:22422] | -2,12 |
| FAM222A-AS1 | [ENSG00000255650](https://www.ensembl.org/id/ENSG00000255650) | FAM222A antisense RNA 1 [Source:HGNC Symbol;Acc:HGNC:28223] | -4,84 |
| FAM225B | [ENSG00000225684](https://www.ensembl.org/id/ENSG00000225684) | family with sequence similarity 225 member B [Source:HGNC Symbol;Acc:HGNC:21865] | -2,43 |
| FAM226B | [ENSG00000269911](https://www.ensembl.org/id/ENSG00000269911) | family with sequence similarity 226 member B [Source:HGNC Symbol;Acc:HGNC:31964] | -2,57 |
| FAM238C | [ENSG00000290706](https://www.ensembl.org/id/ENSG00000290706) | family with sequence similarity 238 member C [Source:HGNC Symbol;Acc:HGNC:24672] | -3,00 |
| FAM240C | [ENSG00000216921](https://www.ensembl.org/id/ENSG00000216921) | family with sequence similarity 240 member C [Source:HGNC Symbol;Acc:HGNC:54200] | -12,20 |
| FAM47E-STBD1 | [ENSG00000272414](https://www.ensembl.org/id/ENSG00000272414) | FAM47E-STBD1 readthrough [Source:HGNC Symbol;Acc:HGNC:44667] | -3,11 |
| FAM50B | [ENSG00000145945](https://www.ensembl.org/id/ENSG00000145945) | family with sequence similarity 50 member B [Source:HGNC Symbol;Acc:HGNC:18789] | -1,12 |
| FAM53B | [ENSG00000189319](https://www.ensembl.org/id/ENSG00000189319) | family with sequence similarity 53 member B [Source:HGNC Symbol;Acc:HGNC:28968] | -1,10 |
| FAM78A | [ENSG00000126882](https://www.ensembl.org/id/ENSG00000126882) | family with sequence similarity 78 member A [Source:HGNC Symbol;Acc:HGNC:25465] | -3,71 |
| FAM78B | [ENSG00000188859](https://www.ensembl.org/id/ENSG00000188859) | family with sequence similarity 78 member B [Source:HGNC Symbol;Acc:HGNC:13495] | -1,79 |
| FAM83B | [ENSG00000168143](https://www.ensembl.org/id/ENSG00000168143) | family with sequence similarity 83 member B [Source:HGNC Symbol;Acc:HGNC:21357] | -8,20 |
| FAM83D | [ENSG00000101447](https://www.ensembl.org/id/ENSG00000101447) | family with sequence similarity 83 member D [Source:HGNC Symbol;Acc:HGNC:16122] | 3,02 |
| FAM83E | [ENSG00000105523](https://www.ensembl.org/id/ENSG00000105523) | family with sequence similarity 83 member E [Source:HGNC Symbol;Acc:HGNC:25972] | -6,16 |
| FAM83F | [ENSG00000133477](https://www.ensembl.org/id/ENSG00000133477) | family with sequence similarity 83 member F [Source:HGNC Symbol;Acc:HGNC:25148] | -6,03 |
| FAM85B | [ENSG00000253893](https://www.ensembl.org/id/ENSG00000253893) | family with sequence similarity 85 member B [Source:HGNC Symbol;Acc:HGNC:32160] | -4,86 |
| FAM88E | [ENSG00000237357](https://www.ensembl.org/id/ENSG00000237357) | family with sequence similarity 88 member E [Source:HGNC Symbol;Acc:HGNC:56161] | 2,41 |
| FANCC | [ENSG00000158169](https://www.ensembl.org/id/ENSG00000158169) | FA complementation group C [Source:HGNC Symbol;Acc:HGNC:3584] | 1,34 |
| FARP1 | [ENSG00000152767](https://www.ensembl.org/id/ENSG00000152767) | FERM, ARH/RhoGEF and pleckstrin domain protein 1 [Source:HGNC Symbol;Acc:HGNC:3591] | 1,06 |
| FAS | [ENSG00000026103](https://www.ensembl.org/id/ENSG00000026103) | Fas cell surface death receptor [Source:HGNC Symbol;Acc:HGNC:11920] | 0,98 |
| FASTK | [ENSG00000164896](https://www.ensembl.org/id/ENSG00000164896) | Fas activated serine/threonine kinase [Source:HGNC Symbol;Acc:HGNC:24676] | -1,51 |
| FAT1 | [ENSG00000083857](https://www.ensembl.org/id/ENSG00000083857) | FAT atypical cadherin 1 [Source:HGNC Symbol;Acc:HGNC:3595] | 1,54 |
| FATE1 | [ENSG00000147378](https://www.ensembl.org/id/ENSG00000147378) | fetal and adult testis expressed 1 [Source:HGNC Symbol;Acc:HGNC:24683] | -3,25 |
| FBP2 | [ENSG00000130957](https://www.ensembl.org/id/ENSG00000130957) | fructose-bisphosphatase 2 [Source:HGNC Symbol;Acc:HGNC:3607] | -9,08 |
| FBXL19-AS1 | [ENSG00000260852](https://www.ensembl.org/id/ENSG00000260852) | FBXL19 antisense RNA 1 [Source:HGNC Symbol;Acc:HGNC:27557] | -2,05 |
| FBXO16 | [ENSG00000214050](https://www.ensembl.org/id/ENSG00000214050) | F-box protein 16 [Source:HGNC Symbol;Acc:HGNC:13618] | 1,83 |
| FBXO2 | [ENSG00000116661](https://www.ensembl.org/id/ENSG00000116661) | F-box protein 2 [Source:HGNC Symbol;Acc:HGNC:13581] | -1,78 |
| FBXO40 | [ENSG00000163833](https://www.ensembl.org/id/ENSG00000163833) | F-box protein 40 [Source:HGNC Symbol;Acc:HGNC:29816] | -9,12 |
| FBXO6 | [ENSG00000116663](https://www.ensembl.org/id/ENSG00000116663) | F-box protein 6 [Source:HGNC Symbol;Acc:HGNC:13585] | -1,56 |
| FCGBP | [ENSG00000275395](https://www.ensembl.org/id/ENSG00000275395) | Fc gamma binding protein [Source:HGNC Symbol;Acc:HGNC:13572] | -4,09 |
| FCGR1A | [ENSG00000150337](https://www.ensembl.org/id/ENSG00000150337) | Fc gamma receptor Ia [Source:HGNC Symbol;Acc:HGNC:3613] | -2,33 |
| FDX1 | [ENSG00000137714](https://www.ensembl.org/id/ENSG00000137714) | ferredoxin 1 [Source:HGNC Symbol;Acc:HGNC:3638] | -1,20 |
| FECH | [ENSG00000066926](https://www.ensembl.org/id/ENSG00000066926) | ferrochelatase [Source:HGNC Symbol;Acc:HGNC:3647] | -1,15 |
| FEM1A | [ENSG00000141965](https://www.ensembl.org/id/ENSG00000141965) | fem-1 homolog A [Source:HGNC Symbol;Acc:HGNC:16934] | -4,15 |
| FER1L5 | [ENSG00000249715](https://www.ensembl.org/id/ENSG00000249715) | fer-1 like family member 5 [Source:HGNC Symbol;Acc:HGNC:19044] | -3,59 |
| FEZ2 | [ENSG00000171055](https://www.ensembl.org/id/ENSG00000171055) | fasciculation and elongation protein zeta 2 [Source:HGNC Symbol;Acc:HGNC:3660] | -2,90 |
| FEZF1-AS1 | [ENSG00000230316](https://www.ensembl.org/id/ENSG00000230316) | FEZF1 antisense RNA 1 [Source:HGNC Symbol;Acc:HGNC:41001] | -5,01 |
| FGF21 | [ENSG00000105550](https://www.ensembl.org/id/ENSG00000105550) | fibroblast growth factor 21 [Source:HGNC Symbol;Acc:HGNC:3678] | -4,93 |
| FGF5 | [ENSG00000138675](https://www.ensembl.org/id/ENSG00000138675) | fibroblast growth factor 5 [Source:HGNC Symbol;Acc:HGNC:3683] | -3,97 |
| FGF6 | [ENSG00000111241](https://www.ensembl.org/id/ENSG00000111241) | fibroblast growth factor 6 [Source:HGNC Symbol;Acc:HGNC:3684] | -8,63 |
| FGFR2 | [ENSG00000066468](https://www.ensembl.org/id/ENSG00000066468) | fibroblast growth factor receptor 2 [Source:HGNC Symbol;Acc:HGNC:3689] | 2,52 |
| FGFR4 | [ENSG00000160867](https://www.ensembl.org/id/ENSG00000160867) | fibroblast growth factor receptor 4 [Source:HGNC Symbol;Acc:HGNC:3691] | -2,63 |
| FGGY | [ENSG00000172456](https://www.ensembl.org/id/ENSG00000172456) | FGGY carbohydrate kinase domain containing [Source:HGNC Symbol;Acc:HGNC:25610] | -1,46 |
| FH | [ENSG00000091483](https://www.ensembl.org/id/ENSG00000091483) | fumarate hydratase [Source:HGNC Symbol;Acc:HGNC:3700] | -1,51 |
| FHAD1-AS1 | [ENSG00000233485](https://www.ensembl.org/id/ENSG00000233485) | FHAD1 antisense RNA 1 [Source:HGNC Symbol;Acc:HGNC:41241] | 4,07 |
| FHIP1A | [ENSG00000164142](https://www.ensembl.org/id/ENSG00000164142) | FHF complex subunit HOOK interacting protein 1A [Source:HGNC Symbol;Acc:HGNC:34237] | -4,96 |
| FHL1 | [ENSG00000022267](https://www.ensembl.org/id/ENSG00000022267) | four and a half LIM domains 1 [Source:HGNC Symbol;Acc:HGNC:3702] | -2,05 |
| FHL3 | [ENSG00000183386](https://www.ensembl.org/id/ENSG00000183386) | four and a half LIM domains 3 [Source:HGNC Symbol;Acc:HGNC:3704] | -4,37 |
| FHOD1 | [ENSG00000135723](https://www.ensembl.org/id/ENSG00000135723) | formin homology 2 domain containing 1 [Source:HGNC Symbol;Acc:HGNC:17905] | -2,50 |
| FIGNL2-DT | [ENSG00000259887](https://www.ensembl.org/id/ENSG00000259887) | FIGNL2 divergent transcript [Source:HGNC Symbol;Acc:HGNC:53299] | -4,68 |
| FITM1 | [ENSG00000139914](https://www.ensembl.org/id/ENSG00000139914) | fat storage inducing transmembrane protein 1 [Source:HGNC Symbol;Acc:HGNC:33714] | -9,25 |
| FKBP3 | [ENSG00000100442](https://www.ensembl.org/id/ENSG00000100442) | FKBP prolyl isomerase 3 [Source:HGNC Symbol;Acc:HGNC:3719] | -2,64 |
| FKBP5 | [ENSG00000096060](https://www.ensembl.org/id/ENSG00000096060) | FKBP prolyl isomerase 5 [Source:HGNC Symbol;Acc:HGNC:3721] | -1,47 |
| FLNB-AS1 | [ENSG00000244161](https://www.ensembl.org/id/ENSG00000244161) | FLNB antisense RNA 1 [Source:HGNC Symbol;Acc:HGNC:40239] | 2,55 |
| FMC1 | [ENSG00000164898](https://www.ensembl.org/id/ENSG00000164898) | formation of mitochondrial complex V assembly factor 1 homolog [Source:HGNC Symbol;Acc:HGNC:26946] | -1,44 |
| FMN1 | [ENSG00000248905](https://www.ensembl.org/id/ENSG00000248905) | formin 1 [Source:HGNC Symbol;Acc:HGNC:3768] | -2,48 |
| FNBP1L | [ENSG00000137942](https://www.ensembl.org/id/ENSG00000137942) | formin binding protein 1 like [Source:HGNC Symbol;Acc:HGNC:20851] | -1,35 |
| FNDC1 | [ENSG00000164694](https://www.ensembl.org/id/ENSG00000164694) | fibronectin type III domain containing 1 [Source:HGNC Symbol;Acc:HGNC:21184] | -1,96 |
| FNDC5 | [ENSG00000160097](https://www.ensembl.org/id/ENSG00000160097) | fibronectin type III domain containing 5 [Source:HGNC Symbol;Acc:HGNC:20240] | -4,66 |
| FOXC1 | [ENSG00000054598](https://www.ensembl.org/id/ENSG00000054598) | forkhead box C1 [Source:HGNC Symbol;Acc:HGNC:3800] | 1,54 |
| FOXC2 | [ENSG00000176692](https://www.ensembl.org/id/ENSG00000176692) | forkhead box C2 [Source:HGNC Symbol;Acc:HGNC:3801] | 1,48 |
| FOXCUT | [ENSG00000280916](https://www.ensembl.org/id/ENSG00000280916) | FOXC1 upstream transcript [Source:HGNC Symbol;Acc:HGNC:50650] | 1,66 |
| FOXD3-AS1 | [ENSG00000230798](https://www.ensembl.org/id/ENSG00000230798) | FOXD3 antisense RNA 1 [Source:HGNC Symbol;Acc:HGNC:40241] | -3,87 |
| FOXD4L1 | [ENSG00000184492](https://www.ensembl.org/id/ENSG00000184492) | forkhead box D4 like 1 [Source:HGNC Symbol;Acc:HGNC:18521] | -3,60 |
| FOXE3 | [ENSG00000186790](https://www.ensembl.org/id/ENSG00000186790) | forkhead box E3 [Source:HGNC Symbol;Acc:HGNC:3808] | -5,21 |
| FOXL1 | [ENSG00000176678](https://www.ensembl.org/id/ENSG00000176678) | forkhead box L1 [Source:HGNC Symbol;Acc:HGNC:3817] | 1,47 |
| FOXO4 | [ENSG00000184481](https://www.ensembl.org/id/ENSG00000184481) | forkhead box O4 [Source:HGNC Symbol;Acc:HGNC:7139] | -1,40 |
| FOXP1-IT1 | [ENSG00000242094](https://www.ensembl.org/id/ENSG00000242094) | FOXP1 intronic transcript 1 [Source:HGNC Symbol;Acc:HGNC:41335] | 4,79 |
| FOXQ1 | [ENSG00000164379](https://www.ensembl.org/id/ENSG00000164379) | forkhead box Q1 [Source:HGNC Symbol;Acc:HGNC:20951] | -5,15 |
| FOXS1 | [ENSG00000179772](https://www.ensembl.org/id/ENSG00000179772) | forkhead box S1 [Source:HGNC Symbol;Acc:HGNC:3735] | 1,98 |
| FRAS1 | [ENSG00000138759](https://www.ensembl.org/id/ENSG00000138759) | Fraser extracellular matrix complex subunit 1 [Source:HGNC Symbol;Acc:HGNC:19185] | -3,36 |
| FRAT1 | [ENSG00000165879](https://www.ensembl.org/id/ENSG00000165879) | FRAT regulator of WNT signaling pathway 1 [Source:HGNC Symbol;Acc:HGNC:3944] | -1,56 |
| FRAT2 | [ENSG00000181274](https://www.ensembl.org/id/ENSG00000181274) | FRAT regulator of WNT signaling pathway 2 [Source:HGNC Symbol;Acc:HGNC:16048] | -1,40 |
| FREM1 | [ENSG00000164946](https://www.ensembl.org/id/ENSG00000164946) | FRAS1 related extracellular matrix 1 [Source:HGNC Symbol;Acc:HGNC:23399] | 1,40 |
| FREM2 | [ENSG00000150893](https://www.ensembl.org/id/ENSG00000150893) | FRAS1 related extracellular matrix 2 [Source:HGNC Symbol;Acc:HGNC:25396] | -6,85 |
| FRK | [ENSG00000111816](https://www.ensembl.org/id/ENSG00000111816) | fyn related Src family tyrosine kinase [Source:HGNC Symbol;Acc:HGNC:3955] | 1,22 |
| FRMD3 | [ENSG00000172159](https://www.ensembl.org/id/ENSG00000172159) | FERM domain containing 3 [Source:HGNC Symbol;Acc:HGNC:24125] | -2,36 |
| FRMPD1 | [ENSG00000070601](https://www.ensembl.org/id/ENSG00000070601) | FERM and PDZ domain containing 1 [Source:HGNC Symbol;Acc:HGNC:29159] | -5,04 |
| FRY-AS1 | [ENSG00000237637](https://www.ensembl.org/id/ENSG00000237637) | FRY antisense RNA 1 [Source:HGNC Symbol;Acc:HGNC:39725] | -3,32 |
| FSD1L | [ENSG00000106701](https://www.ensembl.org/id/ENSG00000106701) | fibronectin type III and SPRY domain containing 1 like [Source:HGNC Symbol;Acc:HGNC:13753] | -2,60 |
| FSD2 | [ENSG00000186628](https://www.ensembl.org/id/ENSG00000186628) | fibronectin type III and SPRY domain containing 2 [Source:HGNC Symbol;Acc:HGNC:18024] | -7,38 |
| FTCDNL1 | [ENSG00000226124](https://www.ensembl.org/id/ENSG00000226124) | formiminotransferase cyclodeaminase N-terminal like [Source:HGNC Symbol;Acc:HGNC:48661] | -4,79 |
| FUNDC2 | [ENSG00000165775](https://www.ensembl.org/id/ENSG00000165775) | FUN14 domain containing 2 [Source:HGNC Symbol;Acc:HGNC:24925] | -1,32 |
| FXN | [ENSG00000165060](https://www.ensembl.org/id/ENSG00000165060) | frataxin [Source:HGNC Symbol;Acc:HGNC:3951] | -1,40 |
| FXR1 | [ENSG00000114416](https://www.ensembl.org/id/ENSG00000114416) | FMR1 autosomal homolog 1 [Source:HGNC Symbol;Acc:HGNC:4023] | -2,29 |
| FXR2 | [ENSG00000129245](https://www.ensembl.org/id/ENSG00000129245) | FMR1 autosomal homolog 2 [Source:HGNC Symbol;Acc:HGNC:4024] | -1,08 |
| FXYD4 | [ENSG00000150201](https://www.ensembl.org/id/ENSG00000150201) | FXYD domain containing ion transport regulator 4 [Source:HGNC Symbol;Acc:HGNC:4028] | -7,82 |
| FYCO1 | [ENSG00000163820](https://www.ensembl.org/id/ENSG00000163820) | FYVE and coiled-coil domain autophagy adaptor 1 [Source:HGNC Symbol;Acc:HGNC:14673] | -1,75 |
| FZD1 | [ENSG00000157240](https://www.ensembl.org/id/ENSG00000157240) | frizzled class receptor 1 [Source:HGNC Symbol;Acc:HGNC:4038] | 1,28 |
| FZD3 | [ENSG00000104290](https://www.ensembl.org/id/ENSG00000104290) | frizzled class receptor 3 [Source:HGNC Symbol;Acc:HGNC:4041] | 1,66 |
| FZD9 | [ENSG00000188763](https://www.ensembl.org/id/ENSG00000188763) | frizzled class receptor 9 [Source:HGNC Symbol;Acc:HGNC:4047] | -4,69 |
| GABBR2 | [ENSG00000136928](https://www.ensembl.org/id/ENSG00000136928) | gamma-aminobutyric acid type B receptor subunit 2 [Source:HGNC Symbol;Acc:HGNC:4507] | -4,25 |
| GABRB2 | [ENSG00000145864](https://www.ensembl.org/id/ENSG00000145864) | gamma-aminobutyric acid type A receptor subunit beta2 [Source:HGNC Symbol;Acc:HGNC:4082] | -3,91 |
| GADD45G | [ENSG00000130222](https://www.ensembl.org/id/ENSG00000130222) | growth arrest and DNA damage inducible gamma [Source:HGNC Symbol;Acc:HGNC:4097] | -1,26 |
| GADL1 | [ENSG00000144644](https://www.ensembl.org/id/ENSG00000144644) | glutamate decarboxylase like 1 [Source:HGNC Symbol;Acc:HGNC:27949] | -6,63 |
| GALNTL6 | [ENSG00000174473](https://www.ensembl.org/id/ENSG00000174473) | polypeptide N-acetylgalactosaminyltransferase like 6 [Source:HGNC Symbol;Acc:HGNC:33844] | -4,11 |
| GALR3 | [ENSG00000128310](https://www.ensembl.org/id/ENSG00000128310) | galanin receptor 3 [Source:HGNC Symbol;Acc:HGNC:4134] | -5,17 |
| GAMT | [ENSG00000130005](https://www.ensembl.org/id/ENSG00000130005) | guanidinoacetate N-methyltransferase [Source:HGNC Symbol;Acc:HGNC:4136] | -3,41 |
| GAPDH | [ENSG00000111640](https://www.ensembl.org/id/ENSG00000111640) | glyceraldehyde-3-phosphate dehydrogenase [Source:HGNC Symbol;Acc:HGNC:4141] | -1,79 |
| GAREM2 | [ENSG00000157833](https://www.ensembl.org/id/ENSG00000157833) | GRB2 associated regulator of MAPK1 subtype 2 [Source:HGNC Symbol;Acc:HGNC:27172] | -2,00 |
| GARIN5A | [ENSG00000142530](https://www.ensembl.org/id/ENSG00000142530) | golgi associated RAB2 interactor 5A [Source:HGNC Symbol;Acc:HGNC:25107] | -3,79 |
| GAS2 | [ENSG00000148935](https://www.ensembl.org/id/ENSG00000148935) | growth arrest specific 2 [Source:HGNC Symbol;Acc:HGNC:4167] | -2,40 |
| GAS2L2 | [ENSG00000270765](https://www.ensembl.org/id/ENSG00000270765) | growth arrest specific 2 like 2 [Source:HGNC Symbol;Acc:HGNC:24846] | -2,64 |
| GATB | [ENSG00000059691](https://www.ensembl.org/id/ENSG00000059691) | glutamyl-tRNA amidotransferase subunit B [Source:HGNC Symbol;Acc:HGNC:8849] | -1,56 |
| GATD3 | [ENSG00000160221](https://www.ensembl.org/id/ENSG00000160221) | glutamine amidotransferase class 1 domain containing 3 [Source:HGNC Symbol;Acc:HGNC:1273] | -2,24 |
| GBX1 | [ENSG00000164900](https://www.ensembl.org/id/ENSG00000164900) | gastrulation brain homeobox 1 [Source:HGNC Symbol;Acc:HGNC:4185] | -4,01 |
| GCAT | [ENSG00000100116](https://www.ensembl.org/id/ENSG00000100116) | glycine C-acetyltransferase [Source:HGNC Symbol;Acc:HGNC:4188] | -1,73 |
| GCNT2 | [ENSG00000111846](https://www.ensembl.org/id/ENSG00000111846) | glucosaminyl (N-acetyl) transferase 2 (I blood group) [Source:HGNC Symbol;Acc:HGNC:4204] | -2,32 |
| GCOM1 | [ENSG00000137878](https://www.ensembl.org/id/ENSG00000137878) | GCOM1, MYZAP-POLR2M combined locus [Source:HGNC Symbol;Acc:HGNC:26424] | -2,00 |
| GCSH | [ENSG00000140905](https://www.ensembl.org/id/ENSG00000140905) | glycine cleavage system protein H [Source:HGNC Symbol;Acc:HGNC:4208] | -1,21 |
| GDA | [ENSG00000119125](https://www.ensembl.org/id/ENSG00000119125) | guanine deaminase [Source:HGNC Symbol;Acc:HGNC:4212] | -8,05 |
| GDF1 | [ENSG00000130283](https://www.ensembl.org/id/ENSG00000130283) | growth differentiation factor 1 [Source:HGNC Symbol;Acc:HGNC:4214] | -3,56 |
| gene:ENSG00000093100 | [ENSG00000093100](https://www.ensembl.org/id/ENSG00000093100) | novel transcript | 0,92 |
| gene:ENSG00000189316 | [ENSG00000189316](https://www.ensembl.org/id/ENSG00000189316) | novel transcript, antisense to ZNF273 | -5,17 |
| gene:ENSG00000197813 | [ENSG00000197813](https://www.ensembl.org/id/ENSG00000197813) | novel transcript | 2,72 |
| gene:ENSG00000203392 | [ENSG00000203392](https://www.ensembl.org/id/ENSG00000203392) | novel transcript, antisense to CSPG4 | 2,25 |
| gene:ENSG00000205414 | [ENSG00000205414](https://www.ensembl.org/id/ENSG00000205414) | novel transcript, antisense to NKD1 | 3,28 |
| gene:ENSG00000206549 | [ENSG00000206549](https://www.ensembl.org/id/ENSG00000206549) | novel protein identical to PRSS50 | -1,98 |
| gene:ENSG00000214970 | [ENSG00000214970](https://www.ensembl.org/id/ENSG00000214970) | novel transcript, antisense to MYH2, MYH8, MYH1 and MYH2 | -7,89 |
| gene:ENSG00000221857 | [ENSG00000221857](https://www.ensembl.org/id/ENSG00000221857) | novel transcript | -5,30 |
| gene:ENSG00000223774 | [ENSG00000223774](https://www.ensembl.org/id/ENSG00000223774) | novel transcript | -6,39 |
| gene:ENSG00000223837 | [ENSG00000223837](https://www.ensembl.org/id/ENSG00000223837) | novel transcript | -3,79 |
| gene:ENSG00000223930 | [ENSG00000223930](https://www.ensembl.org/id/ENSG00000223930) | novel transcript, antisense to KCNMB2 | -4,23 |
| gene:ENSG00000224361 | [ENSG00000224361](https://www.ensembl.org/id/ENSG00000224361) | novel transcript, antisense to KLHL29 | -5,02 |
| gene:ENSG00000224661 | [ENSG00000224661](https://www.ensembl.org/id/ENSG00000224661) | novel transcript | -7,11 |
| gene:ENSG00000224819 | [ENSG00000224819](https://www.ensembl.org/id/ENSG00000224819) | novel transcript | -4,84 |
| gene:ENSG00000224945 | [ENSG00000224945](https://www.ensembl.org/id/ENSG00000224945) | novel transcript | 3,33 |
| gene:ENSG00000225028 | [ENSG00000225028](https://www.ensembl.org/id/ENSG00000225028) | novel transcript | 1,84 |
| gene:ENSG00000225325 | [ENSG00000225325](https://www.ensembl.org/id/ENSG00000225325) | novel transcript | -5,07 |
| gene:ENSG00000225393 | [ENSG00000225393](https://www.ensembl.org/id/ENSG00000225393) | novel transcript | -5,28 |
| gene:ENSG00000225794 | [ENSG00000225794](https://www.ensembl.org/id/ENSG00000225794) | novel transcript | -4,57 |
| gene:ENSG00000226087 | [ENSG00000226087](https://www.ensembl.org/id/ENSG00000226087) | novel transcript | -8,85 |
| gene:ENSG00000226622 | [ENSG00000226622](https://www.ensembl.org/id/ENSG00000226622) | novel transcript | -3,06 |
| gene:ENSG00000226900 | [ENSG00000226900](https://www.ensembl.org/id/ENSG00000226900) | novel transcript | -3,88 |
| gene:ENSG00000227017 | [ENSG00000227017](https://www.ensembl.org/id/ENSG00000227017) | novel transcript | -6,23 |
| gene:ENSG00000227107 | [ENSG00000227107](https://www.ensembl.org/id/ENSG00000227107) | novel transcript | -3,82 |
| gene:ENSG00000227496 | [ENSG00000227496](https://www.ensembl.org/id/ENSG00000227496) | novel transcript | -6,84 |
| gene:ENSG00000227706 | [ENSG00000227706](https://www.ensembl.org/id/ENSG00000227706) | novel transcript | -6,51 |
| gene:ENSG00000228318 | [ENSG00000228318](https://www.ensembl.org/id/ENSG00000228318) | novel transcript | -4,46 |
| gene:ENSG00000228392 | [ENSG00000228392](https://www.ensembl.org/id/ENSG00000228392) | novel transcript | -6,02 |
| gene:ENSG00000228692 | [ENSG00000228692](https://www.ensembl.org/id/ENSG00000228692) | novel transcript | -3,94 |
| gene:ENSG00000228714 | [ENSG00000228714](https://www.ensembl.org/id/ENSG00000228714) | novel transcript, antisense to DEC1 | -5,07 |
| gene:ENSG00000229191 | [ENSG00000229191](https://www.ensembl.org/id/ENSG00000229191) | novel transcript, antisense KIF21B | -4,85 |
| gene:ENSG00000229227 | [ENSG00000229227](https://www.ensembl.org/id/ENSG00000229227) | novel transcript | 2,94 |
| gene:ENSG00000229425 | [ENSG00000229425](https://www.ensembl.org/id/ENSG00000229425) | novel transcript | -3,91 |
| gene:ENSG00000229727 | [ENSG00000229727](https://www.ensembl.org/id/ENSG00000229727) | novel transcript | -4,10 |
| gene:ENSG00000230303 | [ENSG00000230303](https://www.ensembl.org/id/ENSG00000230303) | novel transcript | -3,01 |
| gene:ENSG00000230385 | [ENSG00000230385](https://www.ensembl.org/id/ENSG00000230385) | novel transcript | -5,38 |
| gene:ENSG00000230393 | [ENSG00000230393](https://www.ensembl.org/id/ENSG00000230393) | novel transcript | 2,63 |
| gene:ENSG00000230423 | [ENSG00000230423](https://www.ensembl.org/id/ENSG00000230423) | novel transcript | 3,26 |
| gene:ENSG00000230732 | [ENSG00000230732](https://www.ensembl.org/id/ENSG00000230732) | novel transcript | 2,62 |
| gene:ENSG00000231536 | [ENSG00000231536](https://www.ensembl.org/id/ENSG00000231536) | novel transcript | -6,77 |
| gene:ENSG00000231811 | [ENSG00000231811](https://www.ensembl.org/id/ENSG00000231811) | novel transcript | -3,88 |
| gene:ENSG00000232058 | [ENSG00000232058](https://www.ensembl.org/id/ENSG00000232058) | novel transcript | -6,58 |
| gene:ENSG00000232682 | [ENSG00000232682](https://www.ensembl.org/id/ENSG00000232682) | novel transcript, antisense to ANK3 | -6,05 |
| gene:ENSG00000232748 | [ENSG00000232748](https://www.ensembl.org/id/ENSG00000232748) | novel transcript, antisense to ZNF668 | 2,55 |
| gene:ENSG00000233271 | [ENSG00000233271](https://www.ensembl.org/id/ENSG00000233271) | novel transcript | -3,63 |
| gene:ENSG00000233397 | [ENSG00000233397](https://www.ensembl.org/id/ENSG00000233397) | novel transcript | -5,63 |
| gene:ENSG00000233539 | [ENSG00000233539](https://www.ensembl.org/id/ENSG00000233539) | novel transcript | -4,76 |
| gene:ENSG00000233547 | [ENSG00000233547](https://www.ensembl.org/id/ENSG00000233547) | novel transcript | 4,71 |
| gene:ENSG00000234132 | [ENSG00000234132](https://www.ensembl.org/id/ENSG00000234132) | novel transcript, antisense CACNA1S | -5,22 |
| gene:ENSG00000234139 | [ENSG00000234139](https://www.ensembl.org/id/ENSG00000234139) | novel transcript | -3,22 |
| gene:ENSG00000234147 | [ENSG00000234147](https://www.ensembl.org/id/ENSG00000234147) | novel transcript | -3,45 |
| gene:ENSG00000234740 | [ENSG00000234740](https://www.ensembl.org/id/ENSG00000234740) | novel transcript | -4,16 |
| gene:ENSG00000234929 | [ENSG00000234929](https://www.ensembl.org/id/ENSG00000234929) | novel transcript | -5,54 |
| gene:ENSG00000235027 | [ENSG00000235027](https://www.ensembl.org/id/ENSG00000235027) | PRC2 and DDX5 associated lncRNA [Source:HGNC Symbol;Acc:HGNC:40168] | -3,58 |
| gene:ENSG00000235070 | [ENSG00000235070](https://www.ensembl.org/id/ENSG00000235070) | novel transcript | -7,88 |
| gene:ENSG00000235151 | [ENSG00000235151](https://www.ensembl.org/id/ENSG00000235151) | novel transcript | -2,94 |
| gene:ENSG00000235296 | [ENSG00000235296](https://www.ensembl.org/id/ENSG00000235296) | novel transcript | -6,13 |
| gene:ENSG00000235772 | [ENSG00000235772](https://www.ensembl.org/id/ENSG00000235772) | novel transcript | -3,58 |
| gene:ENSG00000235862 | [ENSG00000235862](https://www.ensembl.org/id/ENSG00000235862) | novel transcript | -3,63 |
| gene:ENSG00000235994 | [ENSG00000235994](https://www.ensembl.org/id/ENSG00000235994) | novel transcript | -5,42 |
| gene:ENSG00000236494 | [ENSG00000236494](https://www.ensembl.org/id/ENSG00000236494) | novel transcript | -3,05 |
| gene:ENSG00000236842 | [ENSG00000236842](https://www.ensembl.org/id/ENSG00000236842) | novel transcript, antisense to C10orf11 | -5,63 |
| gene:ENSG00000236883 | [ENSG00000236883](https://www.ensembl.org/id/ENSG00000236883) | novel transcript | -5,83 |
| gene:ENSG00000236936 | [ENSG00000236936](https://www.ensembl.org/id/ENSG00000236936) | novel transcript | -3,98 |
| gene:ENSG00000237720 | [ENSG00000237720](https://www.ensembl.org/id/ENSG00000237720) | novel transcript | -5,53 |
| gene:ENSG00000238102 | [ENSG00000238102](https://www.ensembl.org/id/ENSG00000238102) | novel transcript | -5,34 |
| gene:ENSG00000238140 | [ENSG00000238140](https://www.ensembl.org/id/ENSG00000238140) | novel transcript | -4,75 |
| gene:ENSG00000239381 | [ENSG00000239381](https://www.ensembl.org/id/ENSG00000239381) | novel transcript | -7,15 |
| gene:ENSG00000239922 | [ENSG00000239922](https://www.ensembl.org/id/ENSG00000239922) | novel transcript | -5,90 |
| gene:ENSG00000241679 | [ENSG00000241679](https://www.ensembl.org/id/ENSG00000241679) | novel transcript | -6,19 |
| gene:ENSG00000242539 | [ENSG00000242539](https://www.ensembl.org/id/ENSG00000242539) | novel transcript | -4,83 |
| gene:ENSG00000243107 | [ENSG00000243107](https://www.ensembl.org/id/ENSG00000243107) | novel transcript | 3,35 |
| gene:ENSG00000243179 | [ENSG00000243179](https://www.ensembl.org/id/ENSG00000243179) | novel transcript | -5,07 |
| gene:ENSG00000243230 | [ENSG00000243230](https://www.ensembl.org/id/ENSG00000243230) | novel transcript | -6,79 |
| gene:ENSG00000243243 | [ENSG00000243243](https://www.ensembl.org/id/ENSG00000243243) | novel transcript | 3,57 |
| gene:ENSG00000243762 | [ENSG00000243762](https://www.ensembl.org/id/ENSG00000243762) | novel transcript, antisense to DGCR8 | -7,22 |
| gene:ENSG00000243944 | [ENSG00000243944](https://www.ensembl.org/id/ENSG00000243944) | novel transcript, antisense to PFN2 | -4,87 |
| gene:ENSG00000244998 | [ENSG00000244998](https://www.ensembl.org/id/ENSG00000244998) | novel transcript, antisense to PTP4A3 | -8,65 |
| gene:ENSG00000246465 | [ENSG00000246465](https://www.ensembl.org/id/ENSG00000246465) | novel transcript LOC100506705 | -6,50 |
| gene:ENSG00000247765 | [ENSG00000247765](https://www.ensembl.org/id/ENSG00000247765) | novel transcript | 2,28 |
| gene:ENSG00000248206 | [ENSG00000248206](https://www.ensembl.org/id/ENSG00000248206) | novel transcript | -4,48 |
| gene:ENSG00000248738 | [ENSG00000248738](https://www.ensembl.org/id/ENSG00000248738) | novel transcript | -3,32 |
| gene:ENSG00000248751 | [ENSG00000248751](https://www.ensembl.org/id/ENSG00000248751) | novel protein | -2,65 |
| gene:ENSG00000248898 | [ENSG00000248898](https://www.ensembl.org/id/ENSG00000248898) | PELO antisense RNA 1 [Source:HGNC Symbol;Acc:HGNC:56263] | 5,35 |
| gene:ENSG00000248964 | [ENSG00000248964](https://www.ensembl.org/id/ENSG00000248964) | novel transcript | -6,13 |
| gene:ENSG00000249209 | [ENSG00000249209](https://www.ensembl.org/id/ENSG00000249209) | novel protein similar to ATP synthase delta (OSCP) subunit domain | -7,02 |
| gene:ENSG00000249593 | [ENSG00000249593](https://www.ensembl.org/id/ENSG00000249593) | novel transcript | -3,62 |
| gene:ENSG00000249773 | [ENSG00000249773](https://www.ensembl.org/id/ENSG00000249773) | novel zinc finger protein 713 (ZNF713) and mitochondrial ribosomal protein S17 (MRPS17) protein | -5,39 |
| gene:ENSG00000250041 | [ENSG00000250041](https://www.ensembl.org/id/ENSG00000250041) | novel transcript | -6,86 |
| gene:ENSG00000250046 | [ENSG00000250046](https://www.ensembl.org/id/ENSG00000250046) | novel transcript | -5,31 |
| gene:ENSG00000250060 | [ENSG00000250060](https://www.ensembl.org/id/ENSG00000250060) | novel transcript | -5,22 |
| gene:ENSG00000250348 | [ENSG00000250348](https://www.ensembl.org/id/ENSG00000250348) | novel transcript | -6,02 |
| gene:ENSG00000250424 | [ENSG00000250424](https://www.ensembl.org/id/ENSG00000250424) | novel protein, MINDY4 and AQP1 readthrough | 5,54 |
| gene:ENSG00000250511 | [ENSG00000250511](https://www.ensembl.org/id/ENSG00000250511) | novel transcript, antisense to ENPEP | -6,22 |
| gene:ENSG00000250615 | [ENSG00000250615](https://www.ensembl.org/id/ENSG00000250615) | novel transcript | -4,78 |
| gene:ENSG00000250697 | [ENSG00000250697](https://www.ensembl.org/id/ENSG00000250697) | novel transcript | -3,32 |
| gene:ENSG00000250978 | [ENSG00000250978](https://www.ensembl.org/id/ENSG00000250978) | novel transcript | -9,78 |
| gene:ENSG00000250994 | [ENSG00000250994](https://www.ensembl.org/id/ENSG00000250994) | novel transcript | -6,89 |
| gene:ENSG00000251076 | [ENSG00000251076](https://www.ensembl.org/id/ENSG00000251076) | novel transcript | -5,91 |
| gene:ENSG00000251081 | [ENSG00000251081](https://www.ensembl.org/id/ENSG00000251081) | novel transcript | -5,98 |
| gene:ENSG00000251244 | [ENSG00000251244](https://www.ensembl.org/id/ENSG00000251244) | novel transcript | 3,25 |
| gene:ENSG00000251257 | [ENSG00000251257](https://www.ensembl.org/id/ENSG00000251257) | novel transcript | -4,18 |
| gene:ENSG00000251615 | [ENSG00000251615](https://www.ensembl.org/id/ENSG00000251615) | novel transcript | 1,24 |
| gene:ENSG00000251665 | [ENSG00000251665](https://www.ensembl.org/id/ENSG00000251665) | novel transcript | -4,51 |
| gene:ENSG00000253115 | [ENSG00000253115](https://www.ensembl.org/id/ENSG00000253115) | novel transcript | -7,70 |
| gene:ENSG00000253123 | [ENSG00000253123](https://www.ensembl.org/id/ENSG00000253123) | novel transcript | 1,93 |
| gene:ENSG00000253227 | [ENSG00000253227](https://www.ensembl.org/id/ENSG00000253227) | novel transcript | -7,21 |
| gene:ENSG00000253389 | [ENSG00000253389](https://www.ensembl.org/id/ENSG00000253389) | novel transcript, antisense to ANK1 | -5,43 |
| gene:ENSG00000253395 | [ENSG00000253395](https://www.ensembl.org/id/ENSG00000253395) | long intergenic non-protein coding RNA 3044 [Source:HGNC Symbol;Acc:HGNC:56250] | -5,07 |
| gene:ENSG00000253515 | [ENSG00000253515](https://www.ensembl.org/id/ENSG00000253515) | novel transcript | -4,85 |
| gene:ENSG00000253844 | [ENSG00000253844](https://www.ensembl.org/id/ENSG00000253844) | novel transcript | -5,95 |
| gene:ENSG00000254054 | [ENSG00000254054](https://www.ensembl.org/id/ENSG00000254054) | novel transcript | -6,33 |
| gene:ENSG00000254343 | [ENSG00000254343](https://www.ensembl.org/id/ENSG00000254343) | novel transcript | -3,49 |
| gene:ENSG00000254485 | [ENSG00000254485](https://www.ensembl.org/id/ENSG00000254485) | novel transcript, antisense to THUMPD3 | -3,43 |
| gene:ENSG00000254519 | [ENSG00000254519](https://www.ensembl.org/id/ENSG00000254519) | novel transcript | -5,16 |
| gene:ENSG00000254692 | [ENSG00000254692](https://www.ensembl.org/id/ENSG00000254692) | novel protein | 7,50 |
| gene:ENSG00000254833 | [ENSG00000254833](https://www.ensembl.org/id/ENSG00000254833) | novel transcript | -5,39 |
| gene:ENSG00000255130 | [ENSG00000255130](https://www.ensembl.org/id/ENSG00000255130) | novel transcript | -5,07 |
| gene:ENSG00000255246 | [ENSG00000255246](https://www.ensembl.org/id/ENSG00000255246) | novel transcript | -6,18 |
| gene:ENSG00000255292 | [ENSG00000255292](https://www.ensembl.org/id/ENSG00000255292) | novel transcript | -4,62 |
| gene:ENSG00000255372 | [ENSG00000255372](https://www.ensembl.org/id/ENSG00000255372) | novel transcript | -5,15 |
| gene:ENSG00000255432 | [ENSG00000255432](https://www.ensembl.org/id/ENSG00000255432) | novel protein | -5,07 |
| gene:ENSG00000255455 | [ENSG00000255455](https://www.ensembl.org/id/ENSG00000255455) | novel transcript | -5,38 |
| gene:ENSG00000255663 | [ENSG00000255663](https://www.ensembl.org/id/ENSG00000255663) | novel transcript, RBM7-REXO2 readthrough | -3,37 |
| gene:ENSG00000255835 | [ENSG00000255835](https://www.ensembl.org/id/ENSG00000255835) | novel protein | 5,47 |
| gene:ENSG00000256349 | [ENSG00000256349](https://www.ensembl.org/id/ENSG00000256349) | novel protein | 4,37 |
| gene:ENSG00000256407 | [ENSG00000256407](https://www.ensembl.org/id/ENSG00000256407) | novel transcript | -5,23 |
| gene:ENSG00000256500 | [ENSG00000256500](https://www.ensembl.org/id/ENSG00000256500) | novel protein | -11,58 |
| gene:ENSG00000256646 | [ENSG00000256646](https://www.ensembl.org/id/ENSG00000256646) | novel PSMA2 and C7orf25 readthrough | -1,09 |
| gene:ENSG00000256861 | [ENSG00000256861](https://www.ensembl.org/id/ENSG00000256861) | novel protein | -5,68 |
| gene:ENSG00000256928 | [ENSG00000256928](https://www.ensembl.org/id/ENSG00000256928) | novel transcript | -5,89 |
| gene:ENSG00000257042 | [ENSG00000257042](https://www.ensembl.org/id/ENSG00000257042) | novel transcript, antisense to PTHLH | -4,18 |
| gene:ENSG00000257060 | [ENSG00000257060](https://www.ensembl.org/id/ENSG00000257060) | novel transcript | -3,13 |
| gene:ENSG00000257277 | [ENSG00000257277](https://www.ensembl.org/id/ENSG00000257277) | novel transcript, antisense to ARHGAP15 | 4,61 |
| gene:ENSG00000257279 | [ENSG00000257279](https://www.ensembl.org/id/ENSG00000257279) | novel transcript | 4,77 |
| gene:ENSG00000257322 | [ENSG00000257322](https://www.ensembl.org/id/ENSG00000257322) | novel transcript, antisense to novel protein | -4,93 |
| gene:ENSG00000257386 | [ENSG00000257386](https://www.ensembl.org/id/ENSG00000257386) | novel transcript, antisense to ATXN7L3B | -5,38 |
| gene:ENSG00000257390 | [ENSG00000257390](https://www.ensembl.org/id/ENSG00000257390) | novel protein | 5,20 |
| gene:ENSG00000258084 | [ENSG00000258084](https://www.ensembl.org/id/ENSG00000258084) | novel transcript | -6,02 |
| gene:ENSG00000258231 | [ENSG00000258231](https://www.ensembl.org/id/ENSG00000258231) | novel transcript | -8,64 |
| gene:ENSG00000258471 | [ENSG00000258471](https://www.ensembl.org/id/ENSG00000258471) | novel transcript, antisense to SLC39A2 | -3,85 |
| gene:ENSG00000258603 | [ENSG00000258603](https://www.ensembl.org/id/ENSG00000258603) | novel transcript, antisense to ACOT6 | -3,22 |
| gene:ENSG00000258604 | [ENSG00000258604](https://www.ensembl.org/id/ENSG00000258604) | novel transcript | -5,56 |
| gene:ENSG00000258660 | [ENSG00000258660](https://www.ensembl.org/id/ENSG00000258660) | novel transcript, antisense to DNAL1 | -5,71 |
| gene:ENSG00000258661 | [ENSG00000258661](https://www.ensembl.org/id/ENSG00000258661) | novel transcript, antisense to PAX9 | 5,07 |
| gene:ENSG00000258695 | [ENSG00000258695](https://www.ensembl.org/id/ENSG00000258695) | novel transcript, antisense to HEATR4 | -6,32 |
| gene:ENSG00000258857 | [ENSG00000258857](https://www.ensembl.org/id/ENSG00000258857) | novel transcript | -4,68 |
| gene:ENSG00000259033 | [ENSG00000259033](https://www.ensembl.org/id/ENSG00000259033) | novel transcript | -3,20 |
| gene:ENSG00000259132 | [ENSG00000259132](https://www.ensembl.org/id/ENSG00000259132) | novel protein | 6,03 |
| gene:ENSG00000259198 | [ENSG00000259198](https://www.ensembl.org/id/ENSG00000259198) | novel transcript, antisense to PLCB2 | -5,38 |
| gene:ENSG00000259219 | [ENSG00000259219](https://www.ensembl.org/id/ENSG00000259219) | novel transcript, antisense to ADAMTS17 | -5,10 |
| gene:ENSG00000259362 | [ENSG00000259362](https://www.ensembl.org/id/ENSG00000259362) | novel transcript | -3,52 |
| gene:ENSG00000259553 | [ENSG00000259553](https://www.ensembl.org/id/ENSG00000259553) | novel transcript | -5,40 |
| gene:ENSG00000259560 | [ENSG00000259560](https://www.ensembl.org/id/ENSG00000259560) | novel transcript | -6,87 |
| gene:ENSG00000259616 | [ENSG00000259616](https://www.ensembl.org/id/ENSG00000259616) | novel transcript | -2,96 |
| gene:ENSG00000259668 | [ENSG00000259668](https://www.ensembl.org/id/ENSG00000259668) | novel transcript, antisense to DMXL2 | 2,64 |
| gene:ENSG00000259675 | [ENSG00000259675](https://www.ensembl.org/id/ENSG00000259675) | novel transcript | -3,39 |
| gene:ENSG00000259744 | [ENSG00000259744](https://www.ensembl.org/id/ENSG00000259744) | novel transcript, sense intronic to LARP6 | 3,38 |
| gene:ENSG00000259828 | [ENSG00000259828](https://www.ensembl.org/id/ENSG00000259828) | novel transcript | -2,31 |
| gene:ENSG00000259881 | [ENSG00000259881](https://www.ensembl.org/id/ENSG00000259881) | novel transcript, antisense to CBFA2T3 | -7,81 |
| gene:ENSG00000259921 | [ENSG00000259921](https://www.ensembl.org/id/ENSG00000259921) | novel transcript, sense intronic to TTC23 | -4,04 |
| gene:ENSG00000260029 | [ENSG00000260029](https://www.ensembl.org/id/ENSG00000260029) | novel transcript, antisense to NKD1 | 5,01 |
| gene:ENSG00000260100 | [ENSG00000260100](https://www.ensembl.org/id/ENSG00000260100) | novel transcript | -3,48 |
| gene:ENSG00000260118 | [ENSG00000260118](https://www.ensembl.org/id/ENSG00000260118) | novel transcript | 2,05 |
| gene:ENSG00000260132 | [ENSG00000260132](https://www.ensembl.org/id/ENSG00000260132) | novel transcript, antisense to UNKL | -4,72 |
| gene:ENSG00000260269 | [ENSG00000260269](https://www.ensembl.org/id/ENSG00000260269) | novel transcript | -5,32 |
| gene:ENSG00000260398 | [ENSG00000260398](https://www.ensembl.org/id/ENSG00000260398) | novel transcript, overlapping to PKIA | -7,73 |
| gene:ENSG00000260401 | [ENSG00000260401](https://www.ensembl.org/id/ENSG00000260401) | novel transcript, overlapping to P2RY2 | -2,15 |
| gene:ENSG00000260487 | [ENSG00000260487](https://www.ensembl.org/id/ENSG00000260487) | novel transcript, antisense to ITGAL | -3,21 |
| gene:ENSG00000260517 | [ENSG00000260517](https://www.ensembl.org/id/ENSG00000260517) | novel transcript | -2,44 |
| gene:ENSG00000260536 | [ENSG00000260536](https://www.ensembl.org/id/ENSG00000260536) | novel transcript, antisense to MAPRE1 | -4,68 |
| gene:ENSG00000260542 | [ENSG00000260542](https://www.ensembl.org/id/ENSG00000260542) | novel transcript | -7,54 |
| gene:ENSG00000260578 | [ENSG00000260578](https://www.ensembl.org/id/ENSG00000260578) | novel transcript | 2,83 |
| gene:ENSG00000260581 | [ENSG00000260581](https://www.ensembl.org/id/ENSG00000260581) | novel transcript, antisense to SPARC | -3,72 |
| gene:ENSG00000260604 | [ENSG00000260604](https://www.ensembl.org/id/ENSG00000260604) | novel transcript | -3,00 |
| gene:ENSG00000260971 | [ENSG00000260971](https://www.ensembl.org/id/ENSG00000260971) | novel transcript | -3,89 |
| gene:ENSG00000261037 | [ENSG00000261037](https://www.ensembl.org/id/ENSG00000261037) | novel transcript | 4,78 |
| gene:ENSG00000261055 | [ENSG00000261055](https://www.ensembl.org/id/ENSG00000261055) | novel transcript | -6,62 |
| gene:ENSG00000261076 | [ENSG00000261076](https://www.ensembl.org/id/ENSG00000261076) | novel transcript | -4,87 |
| gene:ENSG00000261147 | [ENSG00000261147](https://www.ensembl.org/id/ENSG00000261147) | novel protein | -5,05 |
| gene:ENSG00000261168 | [ENSG00000261168](https://www.ensembl.org/id/ENSG00000261168) | novel transcript, sense overlapping SEMA6C | -6,07 |
| gene:ENSG00000261211 | [ENSG00000261211](https://www.ensembl.org/id/ENSG00000261211) | novel transcript | -4,27 |
| gene:ENSG00000261226 | [ENSG00000261226](https://www.ensembl.org/id/ENSG00000261226) | novel transcript | -3,34 |
| gene:ENSG00000261253 | [ENSG00000261253](https://www.ensembl.org/id/ENSG00000261253) | novel transcript, antisense to ANKRD11 | -4,07 |
| gene:ENSG00000261292 | [ENSG00000261292](https://www.ensembl.org/id/ENSG00000261292) | novel transcript | -3,33 |
| gene:ENSG00000261298 | [ENSG00000261298](https://www.ensembl.org/id/ENSG00000261298) | novel transcript | -6,80 |
| gene:ENSG00000261332 | [ENSG00000261332](https://www.ensembl.org/id/ENSG00000261332) | novel transcript, antisense to ITGAL | -5,64 |
| gene:ENSG00000261434 | [ENSG00000261434](https://www.ensembl.org/id/ENSG00000261434) | novel transcript, overlapping LRRC14B | -10,31 |
| gene:ENSG00000261476 | [ENSG00000261476](https://www.ensembl.org/id/ENSG00000261476) | novel transcript | -3,44 |
| gene:ENSG00000261625 | [ENSG00000261625](https://www.ensembl.org/id/ENSG00000261625) | novel transcript, overlapping to MRGPRF | 1,87 |
| gene:ENSG00000261655 | [ENSG00000261655](https://www.ensembl.org/id/ENSG00000261655) | novel transcript, overlapping to GPR20 | 1,71 |
| gene:ENSG00000262410 | [ENSG00000262410](https://www.ensembl.org/id/ENSG00000262410) | novel transcript, sense intronic FN3K | 2,24 |
| gene:ENSG00000262420 | [ENSG00000262420](https://www.ensembl.org/id/ENSG00000262420) | novel transcript, antisense to TXNDC11 | -3,12 |
| gene:ENSG00000262526 | [ENSG00000262526](https://www.ensembl.org/id/ENSG00000262526) | novel protein | 5,08 |
| gene:ENSG00000262633 | [ENSG00000262633](https://www.ensembl.org/id/ENSG00000262633) | novel protein | -5,61 |
| gene:ENSG00000262681 | [ENSG00000262681](https://www.ensembl.org/id/ENSG00000262681) | novel transcript | -4,86 |
| gene:ENSG00000262967 | [ENSG00000262967](https://www.ensembl.org/id/ENSG00000262967) | novel transcript, antisense to ANKRD40 | 5,48 |
| gene:ENSG00000263154 | [ENSG00000263154](https://www.ensembl.org/id/ENSG00000263154) | novel transcript | -5,07 |
| gene:ENSG00000263370 | [ENSG00000263370](https://www.ensembl.org/id/ENSG00000263370) | novel transcript, antisense to SSH2 | -6,59 |
| gene:ENSG00000263489 | [ENSG00000263489](https://www.ensembl.org/id/ENSG00000263489) | novel transcript | -5,36 |
| gene:ENSG00000263603 | [ENSG00000263603](https://www.ensembl.org/id/ENSG00000263603) | novel transcript | -3,86 |
| gene:ENSG00000263859 | [ENSG00000263859](https://www.ensembl.org/id/ENSG00000263859) | novel transcript | -4,76 |
| gene:ENSG00000264007 | [ENSG00000264007](https://www.ensembl.org/id/ENSG00000264007) | novel transcript | -3,50 |
| gene:ENSG00000264116 | [ENSG00000264116](https://www.ensembl.org/id/ENSG00000264116) | novel transcript, antisense to SMIM21 | -5,34 |
| gene:ENSG00000264151 | [ENSG00000264151](https://www.ensembl.org/id/ENSG00000264151) | novel transcript | -8,30 |
| gene:ENSG00000264490 | [ENSG00000264490](https://www.ensembl.org/id/ENSG00000264490) | novel transcript | -6,40 |
| gene:ENSG00000264578 | [ENSG00000264578](https://www.ensembl.org/id/ENSG00000264578) | novel transcript, antisense to AGPAT6 | -5,94 |
| gene:ENSG00000265511 | [ENSG00000265511](https://www.ensembl.org/id/ENSG00000265511) | novel transcript | -3,47 |
| gene:ENSG00000265618 | [ENSG00000265618](https://www.ensembl.org/id/ENSG00000265618) | novel transcript, antisense to TMEM199 | -8,62 |
| gene:ENSG00000265751 | [ENSG00000265751](https://www.ensembl.org/id/ENSG00000265751) | novel transcript, antisense to GREB1L | -8,98 |
| gene:ENSG00000265967 | [ENSG00000265967](https://www.ensembl.org/id/ENSG00000265967) | novel transcript, antisense to ornithine decarboxylase antizyme 2 OAZ2 | -5,05 |
| gene:ENSG00000266602 | [ENSG00000266602](https://www.ensembl.org/id/ENSG00000266602) | novel transcript | -5,63 |
| gene:ENSG00000266718 | [ENSG00000266718](https://www.ensembl.org/id/ENSG00000266718) | novel transcript, antisense MYO1D | -2,35 |
| gene:ENSG00000266844 | [ENSG00000266844](https://www.ensembl.org/id/ENSG00000266844) | novel transcript, antisense to MBP | 5,10 |
| gene:ENSG00000266923 | [ENSG00000266923](https://www.ensembl.org/id/ENSG00000266923) | novel transcript, antisense to CLLU1OS | -3,86 |
| gene:ENSG00000266997 | [ENSG00000266997](https://www.ensembl.org/id/ENSG00000266997) | novel protein | -7,87 |
| gene:ENSG00000267007 | [ENSG00000267007](https://www.ensembl.org/id/ENSG00000267007) | novel transcript, antisense to REXO1 | -6,22 |
| gene:ENSG00000267069 | [ENSG00000267069](https://www.ensembl.org/id/ENSG00000267069) | novel transcript | 2,20 |
| gene:ENSG00000267179 | [ENSG00000267179](https://www.ensembl.org/id/ENSG00000267179) | novel protein | -3,96 |
| gene:ENSG00000267197 | [ENSG00000267197](https://www.ensembl.org/id/ENSG00000267197) | novel transcript, sense intronic with KEAP1 | -5,62 |
| gene:ENSG00000267257 | [ENSG00000267257](https://www.ensembl.org/id/ENSG00000267257) | novel transcript, antisense to ALPK2 | -3,96 |
| gene:ENSG00000267260 | [ENSG00000267260](https://www.ensembl.org/id/ENSG00000267260) | novel transcript | 2,82 |
| gene:ENSG00000267262 | [ENSG00000267262](https://www.ensembl.org/id/ENSG00000267262) | novel transcript, antisense to RFX2 | 5,13 |
| gene:ENSG00000267360 | [ENSG00000267360](https://www.ensembl.org/id/ENSG00000267360) | novel protein | -7,54 |
| gene:ENSG00000267364 | [ENSG00000267364](https://www.ensembl.org/id/ENSG00000267364) | novel transcript | -3,06 |
| gene:ENSG00000267396 | [ENSG00000267396](https://www.ensembl.org/id/ENSG00000267396) | novel transcript, antisense to NEDD4L | -5,64 |
| gene:ENSG00000267417 | [ENSG00000267417](https://www.ensembl.org/id/ENSG00000267417) | novel transcript, antisense to NFIX | -6,48 |
| gene:ENSG00000267423 | [ENSG00000267423](https://www.ensembl.org/id/ENSG00000267423) | novel transcript | -8,00 |
| gene:ENSG00000267665 | [ENSG00000267665](https://www.ensembl.org/id/ENSG00000267665) | novel transcript | -4,82 |
| gene:ENSG00000267677 | [ENSG00000267677](https://www.ensembl.org/id/ENSG00000267677) | novel transcript | -4,71 |
| gene:ENSG00000267784 | [ENSG00000267784](https://www.ensembl.org/id/ENSG00000267784) | novel transcript, antisense to titin | -2,87 |
| gene:ENSG00000268199 | [ENSG00000268199](https://www.ensembl.org/id/ENSG00000268199) | novel transcript, antisense to ELL | -5,63 |
| gene:ENSG00000268205 | [ENSG00000268205](https://www.ensembl.org/id/ENSG00000268205) | novel transcript | 1,10 |
| gene:ENSG00000268401 | [ENSG00000268401](https://www.ensembl.org/id/ENSG00000268401) | novel transcript | 2,95 |
| gene:ENSG00000268460 | [ENSG00000268460](https://www.ensembl.org/id/ENSG00000268460) | novel transcript | -3,02 |
| gene:ENSG00000268518 | [ENSG00000268518](https://www.ensembl.org/id/ENSG00000268518) | novel transcript | -5,82 |
| gene:ENSG00000268536 | [ENSG00000268536](https://www.ensembl.org/id/ENSG00000268536) | novel transcript | -3,73 |
| gene:ENSG00000268643 | [ENSG00000268643](https://www.ensembl.org/id/ENSG00000268643) | novel protein | -3,07 |
| gene:ENSG00000269091 | [ENSG00000269091](https://www.ensembl.org/id/ENSG00000269091) | novel transcript, antisense to ZNF473 | 4,78 |
| gene:ENSG00000269271 | [ENSG00000269271](https://www.ensembl.org/id/ENSG00000269271) | novel transcript | -5,07 |
| gene:ENSG00000269289 | [ENSG00000269289](https://www.ensembl.org/id/ENSG00000269289) | novel transcript, antisense to ZNF726 | -5,38 |
| gene:ENSG00000269349 | [ENSG00000269349](https://www.ensembl.org/id/ENSG00000269349) | novel transcript, antisense to ZNF578 | -5,07 |
| gene:ENSG00000269427 | [ENSG00000269427](https://www.ensembl.org/id/ENSG00000269427) | novel transcript, antisense C19orf42 | 5,83 |
| gene:ENSG00000269446 | [ENSG00000269446](https://www.ensembl.org/id/ENSG00000269446) | novel transcript | -5,87 |
| gene:ENSG00000269560 | [ENSG00000269560](https://www.ensembl.org/id/ENSG00000269560) | novel transcript, sense intronic to ZNF564 | -6,46 |
| gene:ENSG00000269604 | [ENSG00000269604](https://www.ensembl.org/id/ENSG00000269604) | novel transcript, antisense to FEM1A | -4,43 |
| gene:ENSG00000269693 | [ENSG00000269693](https://www.ensembl.org/id/ENSG00000269693) | novel transcript | -6,87 |
| gene:ENSG00000269711 | [ENSG00000269711](https://www.ensembl.org/id/ENSG00000269711) | novel protein | -9,72 |
| gene:ENSG00000269846 | [ENSG00000269846](https://www.ensembl.org/id/ENSG00000269846) | novel transcript, antisense to RBL1 | -5,11 |
| gene:ENSG00000269931 | [ENSG00000269931](https://www.ensembl.org/id/ENSG00000269931) | novel transcript | -4,68 |
| gene:ENSG00000270031 | [ENSG00000270031](https://www.ensembl.org/id/ENSG00000270031) | novel transcript, sense intronic to STX12 | 3,29 |
| gene:ENSG00000270100 | [ENSG00000270100](https://www.ensembl.org/id/ENSG00000270100) | novel transcript | 2,55 |
| gene:ENSG00000270110 | [ENSG00000270110](https://www.ensembl.org/id/ENSG00000270110) | novel transcript, antisense to OBSCN | -6,40 |
| gene:ENSG00000270696 | [ENSG00000270696](https://www.ensembl.org/id/ENSG00000270696) | novel transcript, antisense to C2orf3 | 5,90 |
| gene:ENSG00000270777 | [ENSG00000270777](https://www.ensembl.org/id/ENSG00000270777) | novel transcript | -3,56 |
| gene:ENSG00000271384 | [ENSG00000271384](https://www.ensembl.org/id/ENSG00000271384) | novel transcript | -5,50 |
| gene:ENSG00000271420 | [ENSG00000271420](https://www.ensembl.org/id/ENSG00000271420) | novel transcript | -4,28 |
| gene:ENSG00000271522 | [ENSG00000271522](https://www.ensembl.org/id/ENSG00000271522) | novel transcript | -5,53 |
| gene:ENSG00000271533 | [ENSG00000271533](https://www.ensembl.org/id/ENSG00000271533) | novel transcript, sense intronic FTX | 1,32 |
| gene:ENSG00000271553 | [ENSG00000271553](https://www.ensembl.org/id/ENSG00000271553) | novel transcript | 2,53 |
| gene:ENSG00000271579 | [ENSG00000271579](https://www.ensembl.org/id/ENSG00000271579) | novel transcript | -4,78 |
| gene:ENSG00000271787 | [ENSG00000271787](https://www.ensembl.org/id/ENSG00000271787) | novel transcript, antisense to KLF11 | 1,67 |
| gene:ENSG00000271821 | [ENSG00000271821](https://www.ensembl.org/id/ENSG00000271821) | novel transcript | -4,68 |
| gene:ENSG00000271937 | [ENSG00000271937](https://www.ensembl.org/id/ENSG00000271937) | novel transcript, antisense to TCAIM | -3,75 |
| gene:ENSG00000271992 | [ENSG00000271992](https://www.ensembl.org/id/ENSG00000271992) | novel transcript | -2,49 |
| gene:ENSG00000272078 | [ENSG00000272078](https://www.ensembl.org/id/ENSG00000272078) | novel transcript, antisense to CASZ1 | 1,78 |
| gene:ENSG00000272108 | [ENSG00000272108](https://www.ensembl.org/id/ENSG00000272108) | novel transcript | 2,75 |
| gene:ENSG00000272234 | [ENSG00000272234](https://www.ensembl.org/id/ENSG00000272234) | novel transcript, antisense to SEPP1 | -6,58 |
| gene:ENSG00000272341 | [ENSG00000272341](https://www.ensembl.org/id/ENSG00000272341) | novel transcript | 1,64 |
| gene:ENSG00000272360 | [ENSG00000272360](https://www.ensembl.org/id/ENSG00000272360) | novel transcript | -3,03 |
| gene:ENSG00000272384 | [ENSG00000272384](https://www.ensembl.org/id/ENSG00000272384) | novel transcript | -2,27 |
| gene:ENSG00000272510 | [ENSG00000272510](https://www.ensembl.org/id/ENSG00000272510) | novel transcript, antisense to DNAJC16 | -5,39 |
| gene:ENSG00000272529 | [ENSG00000272529](https://www.ensembl.org/id/ENSG00000272529) | novel transcript, antisense to RFTN1 | 2,70 |
| gene:ENSG00000272566 | [ENSG00000272566](https://www.ensembl.org/id/ENSG00000272566) | novel transcript | -3,26 |
| gene:ENSG00000272622 | [ENSG00000272622](https://www.ensembl.org/id/ENSG00000272622) | novel transcript | -2,60 |
| gene:ENSG00000272631 | [ENSG00000272631](https://www.ensembl.org/id/ENSG00000272631) | novel transcript, antisense to BMPR1A | 2,00 |
| gene:ENSG00000272777 | [ENSG00000272777](https://www.ensembl.org/id/ENSG00000272777) | novel transcript | 2,00 |
| gene:ENSG00000272789 | [ENSG00000272789](https://www.ensembl.org/id/ENSG00000272789) | novel transcript, antisense to MYO7B | 1,98 |
| gene:ENSG00000272829 | [ENSG00000272829](https://www.ensembl.org/id/ENSG00000272829) | novel transcript | -4,26 |
| gene:ENSG00000272871 | [ENSG00000272871](https://www.ensembl.org/id/ENSG00000272871) | novel transcript | 2,82 |
| gene:ENSG00000272885 | [ENSG00000272885](https://www.ensembl.org/id/ENSG00000272885) | novel transcript | -3,61 |
| gene:ENSG00000272936 | [ENSG00000272936](https://www.ensembl.org/id/ENSG00000272936) | novel transcript, antisense to GNPDA2 | -5,80 |
| gene:ENSG00000273026 | [ENSG00000273026](https://www.ensembl.org/id/ENSG00000273026) | novel transcript, antisense to SLC39A1 | 3,18 |
| gene:ENSG00000273090 | [ENSG00000273090](https://www.ensembl.org/id/ENSG00000273090) | novel transcript | -4,87 |
| gene:ENSG00000273139 | [ENSG00000273139](https://www.ensembl.org/id/ENSG00000273139) | novel transcript | -3,23 |
| gene:ENSG00000273145 | [ENSG00000273145](https://www.ensembl.org/id/ENSG00000273145) | novel transcript | -1,81 |
| gene:ENSG00000273155 | [ENSG00000273155](https://www.ensembl.org/id/ENSG00000273155) | novel LIPT1-MRPL30 readthrough | -3,11 |
| gene:ENSG00000273259 | [ENSG00000273259](https://www.ensembl.org/id/ENSG00000273259) | novel protein | -7,53 |
| gene:ENSG00000273355 | [ENSG00000273355](https://www.ensembl.org/id/ENSG00000273355) | novel transcript | -2,17 |
| gene:ENSG00000273387 | [ENSG00000273387](https://www.ensembl.org/id/ENSG00000273387) | novel transcript, antisense to SMTN | 2,72 |
| gene:ENSG00000273391 | [ENSG00000273391](https://www.ensembl.org/id/ENSG00000273391) | novel transcript, antisense to LUC7L2 | -2,85 |
| gene:ENSG00000273424 | [ENSG00000273424](https://www.ensembl.org/id/ENSG00000273424) | novel transcript | -5,03 |
| gene:ENSG00000273487 | [ENSG00000273487](https://www.ensembl.org/id/ENSG00000273487) | novel transcript | -2,41 |
| gene:ENSG00000273796 | [ENSG00000273796](https://www.ensembl.org/id/ENSG00000273796) | novel transcript | 1,67 |
| gene:ENSG00000273908 | [ENSG00000273908](https://www.ensembl.org/id/ENSG00000273908) |  | -5,71 |
| gene:ENSG00000274204 | [ENSG00000274204](https://www.ensembl.org/id/ENSG00000274204) | novel transcript, sense intronic to EFNB2 | -3,27 |
| gene:ENSG00000274825 | [ENSG00000274825](https://www.ensembl.org/id/ENSG00000274825) | novel transcript | 5,05 |
| gene:ENSG00000274944 | [ENSG00000274944](https://www.ensembl.org/id/ENSG00000274944) | novel protein | 6,10 |
| gene:ENSG00000275178 | [ENSG00000275178](https://www.ensembl.org/id/ENSG00000275178) | novel transcript, antisense to MBP | -5,01 |
| gene:ENSG00000275180 | [ENSG00000275180](https://www.ensembl.org/id/ENSG00000275180) | novel transcript | 2,86 |
| gene:ENSG00000275426 | [ENSG00000275426](https://www.ensembl.org/id/ENSG00000275426) | novel transcript | 2,67 |
| gene:ENSG00000275720 | [ENSG00000275720](https://www.ensembl.org/id/ENSG00000275720) | novel transcript | -3,81 |
| gene:ENSG00000275993 | [ENSG00000275993](https://www.ensembl.org/id/ENSG00000275993) | novel protein, similar to salt-inducible kinase 1 SIK1 | 1,78 |
| gene:ENSG00000275995 | [ENSG00000275995](https://www.ensembl.org/id/ENSG00000275995) | novel transcript | -5,38 |
| gene:ENSG00000276012 | [ENSG00000276012](https://www.ensembl.org/id/ENSG00000276012) | novel transcript, antisense to FGF14 | -3,57 |
| gene:ENSG00000276071 | [ENSG00000276071](https://www.ensembl.org/id/ENSG00000276071) | novel transcript | 3,72 |
| gene:ENSG00000276445 | [ENSG00000276445](https://www.ensembl.org/id/ENSG00000276445) | novel transcript | -3,23 |
| gene:ENSG00000276772 | [ENSG00000276772](https://www.ensembl.org/id/ENSG00000276772) | novel transcript | -3,21 |
| gene:ENSG00000276791 | [ENSG00000276791](https://www.ensembl.org/id/ENSG00000276791) | novel transcript | -2,70 |
| gene:ENSG00000277135 | [ENSG00000277135](https://www.ensembl.org/id/ENSG00000277135) | novel transcript | 3,69 |
| gene:ENSG00000277287 | [ENSG00000277287](https://www.ensembl.org/id/ENSG00000277287) | novel transcript | -2,58 |
| gene:ENSG00000277299 | [ENSG00000277299](https://www.ensembl.org/id/ENSG00000277299) | novel transcript, antisense to GIT2 | 4,78 |
| gene:ENSG00000277351 | [ENSG00000277351](https://www.ensembl.org/id/ENSG00000277351) | novel transcript, antisense to SLC51B | 2,41 |
| gene:ENSG00000277534 | [ENSG00000277534](https://www.ensembl.org/id/ENSG00000277534) | novel transcript, sense intronic to KCTD1 | 2,40 |
| gene:ENSG00000277543 | [ENSG00000277543](https://www.ensembl.org/id/ENSG00000277543) | novel transcript, antisense to COX6A2 | -7,24 |
| gene:ENSG00000277797 | [ENSG00000277797](https://www.ensembl.org/id/ENSG00000277797) | novel transcript | -5,21 |
| gene:ENSG00000278022 | [ENSG00000278022](https://www.ensembl.org/id/ENSG00000278022) | novel transcript, sense intronic to IGF1R | 3,48 |
| gene:ENSG00000278254 | [ENSG00000278254](https://www.ensembl.org/id/ENSG00000278254) | novel transcript | -6,44 |
| gene:ENSG00000278291 | [ENSG00000278291](https://www.ensembl.org/id/ENSG00000278291) | novel transcript, antisense to IL17D | -4,31 |
| gene:ENSG00000278434 | [ENSG00000278434](https://www.ensembl.org/id/ENSG00000278434) | novel transcript, sense intronic to CDIP1 | 3,01 |
| gene:ENSG00000278445 | [ENSG00000278445](https://www.ensembl.org/id/ENSG00000278445) | novel transcript | -7,50 |
| gene:ENSG00000278903 | [ENSG00000278903](https://www.ensembl.org/id/ENSG00000278903) | novel transcript | -2,68 |
| gene:ENSG00000278946 | [ENSG00000278946](https://www.ensembl.org/id/ENSG00000278946) | novel transcript, antisense to PCDHA10 | -4,00 |
| gene:ENSG00000279668 | [ENSG00000279668](https://www.ensembl.org/id/ENSG00000279668) | novel transcript | -4,39 |
| gene:ENSG00000279833 | [ENSG00000279833](https://www.ensembl.org/id/ENSG00000279833) | novel transcript, antisense to CBX7 | 1,87 |
| gene:ENSG00000280145 | [ENSG00000280145](https://www.ensembl.org/id/ENSG00000280145) | novel transcript | -2,70 |
| gene:ENSG00000280341 | [ENSG00000280341](https://www.ensembl.org/id/ENSG00000280341) | novel transcript | -6,46 |
| gene:ENSG00000280445 | [ENSG00000280445](https://www.ensembl.org/id/ENSG00000280445) | novel transcript | -5,38 |
| gene:ENSG00000281856 | [ENSG00000281856](https://www.ensembl.org/id/ENSG00000281856) | novel transcript | 3,52 |
| gene:ENSG00000282033 | [ENSG00000282033](https://www.ensembl.org/id/ENSG00000282033) | novel transcript | -5,76 |
| gene:ENSG00000282386 | [ENSG00000282386](https://www.ensembl.org/id/ENSG00000282386) | novel transcript, antisense to SLC39A1 | -2,18 |
| gene:ENSG00000282907 | [ENSG00000282907](https://www.ensembl.org/id/ENSG00000282907) | novel transcript | -4,66 |
| gene:ENSG00000283064 | [ENSG00000283064](https://www.ensembl.org/id/ENSG00000283064) | novel transcript, antisense to HIST1H2BD | -2,87 |
| gene:ENSG00000283098 | [ENSG00000283098](https://www.ensembl.org/id/ENSG00000283098) | novel transcript | 2,59 |
| gene:ENSG00000283189 | [ENSG00000283189](https://www.ensembl.org/id/ENSG00000283189) | novel protein | 1,10 |
| gene:ENSG00000283213 | [ENSG00000283213](https://www.ensembl.org/id/ENSG00000283213) | novel transcript | 2,66 |
| gene:ENSG00000283228 | [ENSG00000283228](https://www.ensembl.org/id/ENSG00000283228) | novel transcript | -8,50 |
| gene:ENSG00000283294 | [ENSG00000283294](https://www.ensembl.org/id/ENSG00000283294) | novel transcript | -2,98 |
| gene:ENSG00000283403 | [ENSG00000283403](https://www.ensembl.org/id/ENSG00000283403) | novel transcript | -5,06 |
| gene:ENSG00000283633 | [ENSG00000283633](https://www.ensembl.org/id/ENSG00000283633) | novel transcript | -2,33 |
| gene:ENSG00000283662 | [ENSG00000283662](https://www.ensembl.org/id/ENSG00000283662) | novel transcript | -9,72 |
| gene:ENSG00000283809 | [ENSG00000283809](https://www.ensembl.org/id/ENSG00000283809) | novel protein | -4,02 |
| gene:ENSG00000283828 | [ENSG00000283828](https://www.ensembl.org/id/ENSG00000283828) | novel transcript, antisense to F10 | -3,13 |
| gene:ENSG00000283897 | [ENSG00000283897](https://www.ensembl.org/id/ENSG00000283897) | novel transcript | -3,83 |
| gene:ENSG00000284052 | [ENSG00000284052](https://www.ensembl.org/id/ENSG00000284052) | novel transcript | 2,16 |
| gene:ENSG00000284292 | [ENSG00000284292](https://www.ensembl.org/id/ENSG00000284292) | novel protein, ARPC1A and ARPC1B readthrough | 6,24 |
| gene:ENSG00000284644 | [ENSG00000284644](https://www.ensembl.org/id/ENSG00000284644) | novel transcript, antisense to OR2A1 and ARHGEF5 | -6,98 |
| gene:ENSG00000284672 | [ENSG00000284672](https://www.ensembl.org/id/ENSG00000284672) | novel transcript | -2,29 |
| gene:ENSG00000284720 | [ENSG00000284720](https://www.ensembl.org/id/ENSG00000284720) | novel transcript | -5,38 |
| gene:ENSG00000284931 | [ENSG00000284931](https://www.ensembl.org/id/ENSG00000284931) | novel protein | 3,20 |
| gene:ENSG00000284952 | [ENSG00000284952](https://www.ensembl.org/id/ENSG00000284952) | novel transcript | -2,65 |
| gene:ENSG00000284968 | [ENSG00000284968](https://www.ensembl.org/id/ENSG00000284968) | Novel transcript, antisense to AFF1 | -1,55 |
| gene:ENSG00000284977 | [ENSG00000284977](https://www.ensembl.org/id/ENSG00000284977) | novel transcript | -2,21 |
| gene:ENSG00000285043 | [ENSG00000285043](https://www.ensembl.org/id/ENSG00000285043) | novel protein | -1,68 |
| gene:ENSG00000285079 | [ENSG00000285079](https://www.ensembl.org/id/ENSG00000285079) | novel transcript | -6,04 |
| gene:ENSG00000285082 | [ENSG00000285082](https://www.ensembl.org/id/ENSG00000285082) | novel protein | -2,66 |
| gene:ENSG00000285155 | [ENSG00000285155](https://www.ensembl.org/id/ENSG00000285155) | novel transcript | -8,85 |
| gene:ENSG00000285238 | [ENSG00000285238](https://www.ensembl.org/id/ENSG00000285238) | novel transcript | 6,09 |
| gene:ENSG00000285530 | [ENSG00000285530](https://www.ensembl.org/id/ENSG00000285530) | novel transcript, antisense to DBT | -4,37 |
| gene:ENSG00000285547 | [ENSG00000285547](https://www.ensembl.org/id/ENSG00000285547) | novel protein | 5,07 |
| gene:ENSG00000285563 | [ENSG00000285563](https://www.ensembl.org/id/ENSG00000285563) | novel transcript | -7,36 |
| gene:ENSG00000285564 | [ENSG00000285564](https://www.ensembl.org/id/ENSG00000285564) | novel transcript | -6,24 |
| gene:ENSG00000285610 | [ENSG00000285610](https://www.ensembl.org/id/ENSG00000285610) | novel transcript, antisense to PACRG | -5,47 |
| gene:ENSG00000285658 | [ENSG00000285658](https://www.ensembl.org/id/ENSG00000285658) | novel transcript, antisense to AMBRA1 | -7,06 |
| gene:ENSG00000285681 | [ENSG00000285681](https://www.ensembl.org/id/ENSG00000285681) | novel transcript, antisense to MC4R | -5,63 |
| gene:ENSG00000285713 | [ENSG00000285713](https://www.ensembl.org/id/ENSG00000285713) | novel transcript | -3,95 |
| gene:ENSG00000285750 | [ENSG00000285750](https://www.ensembl.org/id/ENSG00000285750) | novel transcript | -4,32 |
| gene:ENSG00000285920 | [ENSG00000285920](https://www.ensembl.org/id/ENSG00000285920) | novel protein | -7,77 |
| gene:ENSG00000285942 | [ENSG00000285942](https://www.ensembl.org/id/ENSG00000285942) | novel protein | -2,42 |
| gene:ENSG00000285987 | [ENSG00000285987](https://www.ensembl.org/id/ENSG00000285987) | Novel transcript, antisense to SLC28A3 | -6,46 |
| gene:ENSG00000286084 | [ENSG00000286084](https://www.ensembl.org/id/ENSG00000286084) | novel transcript | -3,94 |
| gene:ENSG00000286164 | [ENSG00000286164](https://www.ensembl.org/id/ENSG00000286164) | novel transcript, antisense to OXCT1 | -3,76 |
| gene:ENSG00000286185 | [ENSG00000286185](https://www.ensembl.org/id/ENSG00000286185) | novel protein, identical to neuroblastoma breakpoint family, member 19 NBPF19 | 2,76 |
| gene:ENSG00000286194 | [ENSG00000286194](https://www.ensembl.org/id/ENSG00000286194) | Novel transcript | -5,89 |
| gene:ENSG00000286231 | [ENSG00000286231](https://www.ensembl.org/id/ENSG00000286231) | novel protein | 2,86 |
| gene:ENSG00000286235 | [ENSG00000286235](https://www.ensembl.org/id/ENSG00000286235) | novel protein | -3,41 |
| gene:ENSG00000286248 | [ENSG00000286248](https://www.ensembl.org/id/ENSG00000286248) | novel transcript, antisense to P2RX7 | 3,12 |
| gene:ENSG00000286257 | [ENSG00000286257](https://www.ensembl.org/id/ENSG00000286257) | novel transcript | -6,78 |
| gene:ENSG00000286272 | [ENSG00000286272](https://www.ensembl.org/id/ENSG00000286272) | novel transcript | -5,69 |
| gene:ENSG00000286289 | [ENSG00000286289](https://www.ensembl.org/id/ENSG00000286289) | novel transcript | 1,55 |
| gene:ENSG00000286322 | [ENSG00000286322](https://www.ensembl.org/id/ENSG00000286322) | novel transcript | -4,86 |
| gene:ENSG00000286326 | [ENSG00000286326](https://www.ensembl.org/id/ENSG00000286326) | novel transcript, antisense to CRYBB1 | -4,18 |
| gene:ENSG00000286378 | [ENSG00000286378](https://www.ensembl.org/id/ENSG00000286378) | novel transcript | -3,37 |
| gene:ENSG00000286415 | [ENSG00000286415](https://www.ensembl.org/id/ENSG00000286415) | novel transcript | -3,77 |
| gene:ENSG00000286445 | [ENSG00000286445](https://www.ensembl.org/id/ENSG00000286445) | novel transcript | -4,46 |
| gene:ENSG00000286452 | [ENSG00000286452](https://www.ensembl.org/id/ENSG00000286452) | novel transcript | -5,86 |
| gene:ENSG00000286563 | [ENSG00000286563](https://www.ensembl.org/id/ENSG00000286563) | novel transcript | -5,71 |
| gene:ENSG00000286584 | [ENSG00000286584](https://www.ensembl.org/id/ENSG00000286584) | novel transcript, antisense to COX17 | -2,79 |
| gene:ENSG00000286618 | [ENSG00000286618](https://www.ensembl.org/id/ENSG00000286618) | novel transcript, antisense to PKD2and SPP1 | -5,47 |
| gene:ENSG00000286658 | [ENSG00000286658](https://www.ensembl.org/id/ENSG00000286658) | novel transcript, antisense to GRB10 | -9,53 |
| gene:ENSG00000286690 | [ENSG00000286690](https://www.ensembl.org/id/ENSG00000286690) | novel transcript, antisense to SGCD | -5,10 |
| gene:ENSG00000286694 | [ENSG00000286694](https://www.ensembl.org/id/ENSG00000286694) | novel transcript | -5,03 |
| gene:ENSG00000286713 | [ENSG00000286713](https://www.ensembl.org/id/ENSG00000286713) | novel transcript | -3,74 |
| gene:ENSG00000286773 | [ENSG00000286773](https://www.ensembl.org/id/ENSG00000286773) | novel transcript, sense intronic to OBSCN | -4,66 |
| gene:ENSG00000286788 | [ENSG00000286788](https://www.ensembl.org/id/ENSG00000286788) | novel transcript, antisense to LPO | -5,65 |
| gene:ENSG00000286881 | [ENSG00000286881](https://www.ensembl.org/id/ENSG00000286881) | novel transcript, sense intronic to MLLT10 | -5,22 |
| gene:ENSG00000286907 | [ENSG00000286907](https://www.ensembl.org/id/ENSG00000286907) | novel transcript | -3,79 |
| gene:ENSG00000286952 | [ENSG00000286952](https://www.ensembl.org/id/ENSG00000286952) | novel transcript, antisense to DNAH12 | -2,83 |
| gene:ENSG00000286957 | [ENSG00000286957](https://www.ensembl.org/id/ENSG00000286957) | novel transcript | -5,98 |
| gene:ENSG00000286964 | [ENSG00000286964](https://www.ensembl.org/id/ENSG00000286964) | novel transcript | 1,54 |
| gene:ENSG00000286986 | [ENSG00000286986](https://www.ensembl.org/id/ENSG00000286986) | novel transcript | -6,23 |
| gene:ENSG00000287000 | [ENSG00000287000](https://www.ensembl.org/id/ENSG00000287000) | novel transcript | -4,68 |
| gene:ENSG00000287023 | [ENSG00000287023](https://www.ensembl.org/id/ENSG00000287023) | novel transcript | -5,26 |
| gene:ENSG00000287026 | [ENSG00000287026](https://www.ensembl.org/id/ENSG00000287026) | novel transcript | -6,98 |
| gene:ENSG00000287047 | [ENSG00000287047](https://www.ensembl.org/id/ENSG00000287047) | novel transcript, antisense to SORCS1 | -8,24 |
| gene:ENSG00000287048 | [ENSG00000287048](https://www.ensembl.org/id/ENSG00000287048) | novel transcript | -7,88 |
| gene:ENSG00000287075 | [ENSG00000287075](https://www.ensembl.org/id/ENSG00000287075) | novel transcript | 2,96 |
| gene:ENSG00000287125 | [ENSG00000287125](https://www.ensembl.org/id/ENSG00000287125) | novel transcript, antisense to C16orf74 | -5,38 |
| gene:ENSG00000287130 | [ENSG00000287130](https://www.ensembl.org/id/ENSG00000287130) | novel transcript | -5,81 |
| gene:ENSG00000287170 | [ENSG00000287170](https://www.ensembl.org/id/ENSG00000287170) | novel transcript, antisense to PPP1R12C | -3,23 |
| gene:ENSG00000287234 | [ENSG00000287234](https://www.ensembl.org/id/ENSG00000287234) | novel transcript | -5,19 |
| gene:ENSG00000287237 | [ENSG00000287237](https://www.ensembl.org/id/ENSG00000287237) | novel transcript, antisense to PLXNC1 | -6,86 |
| gene:ENSG00000287272 | [ENSG00000287272](https://www.ensembl.org/id/ENSG00000287272) | novel transcript | -5,70 |
| gene:ENSG00000287292 | [ENSG00000287292](https://www.ensembl.org/id/ENSG00000287292) | novel transcript | -3,07 |
| gene:ENSG00000287307 | [ENSG00000287307](https://www.ensembl.org/id/ENSG00000287307) | novel transcript | -5,63 |
| gene:ENSG00000287309 | [ENSG00000287309](https://www.ensembl.org/id/ENSG00000287309) | novel transcript | -7,16 |
| gene:ENSG00000287340 | [ENSG00000287340](https://www.ensembl.org/id/ENSG00000287340) | novel transcript, antisense to RBFOX1 | -7,77 |
| gene:ENSG00000287380 | [ENSG00000287380](https://www.ensembl.org/id/ENSG00000287380) | novel transcript | -4,68 |
| gene:ENSG00000287381 | [ENSG00000287381](https://www.ensembl.org/id/ENSG00000287381) | novel transcript, antisense to MAPK4 | -4,06 |
| gene:ENSG00000287385 | [ENSG00000287385](https://www.ensembl.org/id/ENSG00000287385) | novel transcript, antisense to RAD51B | 4,91 |
| gene:ENSG00000287401 | [ENSG00000287401](https://www.ensembl.org/id/ENSG00000287401) | novel transcript, antisense to STBD1and FAM47E | -3,76 |
| gene:ENSG00000287483 | [ENSG00000287483](https://www.ensembl.org/id/ENSG00000287483) | novel transcript | -7,64 |
| gene:ENSG00000287502 | [ENSG00000287502](https://www.ensembl.org/id/ENSG00000287502) | novel transcript | -4,09 |
| gene:ENSG00000287553 | [ENSG00000287553](https://www.ensembl.org/id/ENSG00000287553) | novel transcript, antisense to DPH1 | 2,13 |
| gene:ENSG00000287580 | [ENSG00000287580](https://www.ensembl.org/id/ENSG00000287580) | novel transcript, antisense to ZNF138 | -7,22 |
| gene:ENSG00000287655 | [ENSG00000287655](https://www.ensembl.org/id/ENSG00000287655) | novel transcript, antisense to SHTN1 | -6,58 |
| gene:ENSG00000287692 | [ENSG00000287692](https://www.ensembl.org/id/ENSG00000287692) | novel transcript | -3,43 |
| gene:ENSG00000287715 | [ENSG00000287715](https://www.ensembl.org/id/ENSG00000287715) | novel transcript | -3,53 |
| gene:ENSG00000287729 | [ENSG00000287729](https://www.ensembl.org/id/ENSG00000287729) | novel transcript | -4,98 |
| gene:ENSG00000287792 | [ENSG00000287792](https://www.ensembl.org/id/ENSG00000287792) | novel transcript | -4,86 |
| gene:ENSG00000287891 | [ENSG00000287891](https://www.ensembl.org/id/ENSG00000287891) | novel transcript | -6,89 |
| gene:ENSG00000287910 | [ENSG00000287910](https://www.ensembl.org/id/ENSG00000287910) | novel transcript | 2,28 |
| gene:ENSG00000287922 | [ENSG00000287922](https://www.ensembl.org/id/ENSG00000287922) | novel transcript | 2,63 |
| gene:ENSG00000287927 | [ENSG00000287927](https://www.ensembl.org/id/ENSG00000287927) | novel transcript | -5,67 |
| gene:ENSG00000287958 | [ENSG00000287958](https://www.ensembl.org/id/ENSG00000287958) | novel transcript | -5,30 |
| gene:ENSG00000288009 | [ENSG00000288009](https://www.ensembl.org/id/ENSG00000288009) | novel transcript | -2,10 |
| gene:ENSG00000288067 | [ENSG00000288067](https://www.ensembl.org/id/ENSG00000288067) | novel transcript | -5,30 |
| gene:ENSG00000288068 | [ENSG00000288068](https://www.ensembl.org/id/ENSG00000288068) | novel transcript, sense intronic to NOX5 | -5,48 |
| gene:ENSG00000288162 | [ENSG00000288162](https://www.ensembl.org/id/ENSG00000288162) | novel transcript | -5,30 |
| gene:ENSG00000288531 | [ENSG00000288531](https://www.ensembl.org/id/ENSG00000288531) | novel transcript | -3,15 |
| gene:ENSG00000288636 | [ENSG00000288636](https://www.ensembl.org/id/ENSG00000288636) | novel protein | -6,33 |
| gene:ENSG00000288640 | [ENSG00000288640](https://www.ensembl.org/id/ENSG00000288640) | novel protein | -1,19 |
| gene:ENSG00000288670 | [ENSG00000288670](https://www.ensembl.org/id/ENSG00000288670) | novel transcript | -2,20 |
| gene:ENSG00000288698 | [ENSG00000288698](https://www.ensembl.org/id/ENSG00000288698) | novel protein | -4,70 |
| gene:ENSG00000288703 | [ENSG00000288703](https://www.ensembl.org/id/ENSG00000288703) | novel transcript | -5,19 |
| gene:ENSG00000288704 | [ENSG00000288704](https://www.ensembl.org/id/ENSG00000288704) | novel transcript, antisense to MIDN | -3,48 |
| gene:ENSG00000288727 | [ENSG00000288727](https://www.ensembl.org/id/ENSG00000288727) | novel transcript, antisense to NUP214 | -4,68 |
| gene:ENSG00000288758 | [ENSG00000288758](https://www.ensembl.org/id/ENSG00000288758) | novel transcript, antisense to FSTL4 | -3,74 |
| gene:ENSG00000288777 | [ENSG00000288777](https://www.ensembl.org/id/ENSG00000288777) | novel transcript, sense intronic to ANKRD54and MYH9 | -7,99 |
| gene:ENSG00000288790 | [ENSG00000288790](https://www.ensembl.org/id/ENSG00000288790) | novel transcript | -3,30 |
| gene:ENSG00000288809 | [ENSG00000288809](https://www.ensembl.org/id/ENSG00000288809) | novel transcript, antisense to EED | -2,73 |
| gene:ENSG00000288832 | [ENSG00000288832](https://www.ensembl.org/id/ENSG00000288832) | novel transcript, antisense to CNKSR2 | -5,48 |
| gene:ENSG00000288849 | [ENSG00000288849](https://www.ensembl.org/id/ENSG00000288849) | novel transcript | -2,02 |
| gene:ENSG00000288856 | [ENSG00000288856](https://www.ensembl.org/id/ENSG00000288856) | novel transcript, antisense to BCOR | 2,34 |
| gene:ENSG00000288862 | [ENSG00000288862](https://www.ensembl.org/id/ENSG00000288862) | novel transcript | -4,69 |
| gene:ENSG00000288912 | [ENSG00000288912](https://www.ensembl.org/id/ENSG00000288912) | novel transcript | -5,76 |
| gene:ENSG00000288938 | [ENSG00000288938](https://www.ensembl.org/id/ENSG00000288938) | novel transcript | -5,24 |
| gene:ENSG00000288960 | [ENSG00000288960](https://www.ensembl.org/id/ENSG00000288960) | novel transcript | -6,02 |
| gene:ENSG00000288970 | [ENSG00000288970](https://www.ensembl.org/id/ENSG00000288970) | novel transcript | -4,57 |
| gene:ENSG00000288980 | [ENSG00000288980](https://www.ensembl.org/id/ENSG00000288980) | novel transcript | -9,21 |
| gene:ENSG00000288984 | [ENSG00000288984](https://www.ensembl.org/id/ENSG00000288984) | novel transcript, antisense to ITFG2 | 1,50 |
| gene:ENSG00000289027 | [ENSG00000289027](https://www.ensembl.org/id/ENSG00000289027) | novel protein | -2,48 |
| gene:ENSG00000289062 | [ENSG00000289062](https://www.ensembl.org/id/ENSG00000289062) | novel transcript, antisense to IVL | -6,99 |
| gene:ENSG00000289065 | [ENSG00000289065](https://www.ensembl.org/id/ENSG00000289065) | novel transcript, antisense to ACYP2 | -2,09 |
| gene:ENSG00000289076 | [ENSG00000289076](https://www.ensembl.org/id/ENSG00000289076) | novel transcript | -4,82 |
| gene:ENSG00000289080 | [ENSG00000289080](https://www.ensembl.org/id/ENSG00000289080) | novel transcript | 3,74 |
| gene:ENSG00000289088 | [ENSG00000289088](https://www.ensembl.org/id/ENSG00000289088) | novel transcript | 2,61 |
| gene:ENSG00000289096 | [ENSG00000289096](https://www.ensembl.org/id/ENSG00000289096) | novel transcript, antisense to PITX2 | -5,77 |
| gene:ENSG00000289097 | [ENSG00000289097](https://www.ensembl.org/id/ENSG00000289097) | novel transcript | -4,07 |
| gene:ENSG00000289120 | [ENSG00000289120](https://www.ensembl.org/id/ENSG00000289120) | novel transcript | -5,19 |
| gene:ENSG00000289134 | [ENSG00000289134](https://www.ensembl.org/id/ENSG00000289134) | novel transcript, sense intronic to POPDC2 | -2,95 |
| gene:ENSG00000289135 | [ENSG00000289135](https://www.ensembl.org/id/ENSG00000289135) | novel transcript | -1,67 |
| gene:ENSG00000289146 | [ENSG00000289146](https://www.ensembl.org/id/ENSG00000289146) | novel transcript | -4,96 |
| gene:ENSG00000289164 | [ENSG00000289164](https://www.ensembl.org/id/ENSG00000289164) | novel transcript, sense intronic to RANBP10 | -2,77 |
| gene:ENSG00000289183 | [ENSG00000289183](https://www.ensembl.org/id/ENSG00000289183) | novel transcript | -2,39 |
| gene:ENSG00000289187 | [ENSG00000289187](https://www.ensembl.org/id/ENSG00000289187) | novel transcript, sense intronic to ZNF536 | -5,63 |
| gene:ENSG00000289251 | [ENSG00000289251](https://www.ensembl.org/id/ENSG00000289251) | novel transcript | 3,36 |
| gene:ENSG00000289254 | [ENSG00000289254](https://www.ensembl.org/id/ENSG00000289254) | novel transcript, antisense to NMRK2 | -3,83 |
| gene:ENSG00000289302 | [ENSG00000289302](https://www.ensembl.org/id/ENSG00000289302) | novel transcript | -2,61 |
| gene:ENSG00000289317 | [ENSG00000289317](https://www.ensembl.org/id/ENSG00000289317) | novel transcript | -3,34 |
| gene:ENSG00000289345 | [ENSG00000289345](https://www.ensembl.org/id/ENSG00000289345) | novel transcript, antisense to ASB15 | -5,33 |
| gene:ENSG00000289351 | [ENSG00000289351](https://www.ensembl.org/id/ENSG00000289351) | novel transcript | -3,44 |
| gene:ENSG00000289376 | [ENSG00000289376](https://www.ensembl.org/id/ENSG00000289376) | novel transcript, sense overlapping FRK | 3,20 |
| gene:ENSG00000289379 | [ENSG00000289379](https://www.ensembl.org/id/ENSG00000289379) | novel transcript | -5,84 |
| gene:ENSG00000289386 | [ENSG00000289386](https://www.ensembl.org/id/ENSG00000289386) | novel transcript | -5,26 |
| gene:ENSG00000289422 | [ENSG00000289422](https://www.ensembl.org/id/ENSG00000289422) | novel transcript | -5,54 |
| gene:ENSG00000289423 | [ENSG00000289423](https://www.ensembl.org/id/ENSG00000289423) | novel transcript | 4,61 |
| gene:ENSG00000289463 | [ENSG00000289463](https://www.ensembl.org/id/ENSG00000289463) | novel transcript, sense intronic to ZNF638 | 3,32 |
| gene:ENSG00000289469 | [ENSG00000289469](https://www.ensembl.org/id/ENSG00000289469) | novel transcript | -3,33 |
| gene:ENSG00000289511 | [ENSG00000289511](https://www.ensembl.org/id/ENSG00000289511) | novel transcript | 2,49 |
| gene:ENSG00000289515 | [ENSG00000289515](https://www.ensembl.org/id/ENSG00000289515) | novel transcript | -2,54 |
| gene:ENSG00000289526 | [ENSG00000289526](https://www.ensembl.org/id/ENSG00000289526) | novel transcript | -4,18 |
| gene:ENSG00000289528 | [ENSG00000289528](https://www.ensembl.org/id/ENSG00000289528) | novel transcript, sense intronic to MLLT10 | -3,57 |
| gene:ENSG00000289550 | [ENSG00000289550](https://www.ensembl.org/id/ENSG00000289550) | novel transcript | 4,95 |
| gene:ENSG00000289571 | [ENSG00000289571](https://www.ensembl.org/id/ENSG00000289571) | novel transcript | -6,35 |
| gene:ENSG00000289579 | [ENSG00000289579](https://www.ensembl.org/id/ENSG00000289579) | novel transcript, sense intronic to PTRF | -2,83 |
| gene:ENSG00000289587 | [ENSG00000289587](https://www.ensembl.org/id/ENSG00000289587) | novel transcript, antisense to PRKCA | -3,45 |
| gene:ENSG00000289594 | [ENSG00000289594](https://www.ensembl.org/id/ENSG00000289594) | novel transcript, antisense to TMTC4 | -4,71 |
| gene:ENSG00000289609 | [ENSG00000289609](https://www.ensembl.org/id/ENSG00000289609) | novel transcript | -7,20 |
| gene:ENSG00000289757 | [ENSG00000289757](https://www.ensembl.org/id/ENSG00000289757) | novel transcript | -3,57 |
| gene:ENSG00000289810 | [ENSG00000289810](https://www.ensembl.org/id/ENSG00000289810) | novel transcript | -4,60 |
| gene:ENSG00000289950 | [ENSG00000289950](https://www.ensembl.org/id/ENSG00000289950) | novel transcript | -4,52 |
| gene:ENSG00000289973 | [ENSG00000289973](https://www.ensembl.org/id/ENSG00000289973) | novel transcript, sense intronic to MYH6 | -7,25 |
| gene:ENSG00000289989 | [ENSG00000289989](https://www.ensembl.org/id/ENSG00000289989) | novel transcript, antisense to RHOBTB1 | -5,00 |
| gene:ENSG00000290025 | [ENSG00000290025](https://www.ensembl.org/id/ENSG00000290025) | novel transcript | -5,76 |
| gene:ENSG00000290029 | [ENSG00000290029](https://www.ensembl.org/id/ENSG00000290029) | novel transcript | -4,79 |
| gene:ENSG00000290034 | [ENSG00000290034](https://www.ensembl.org/id/ENSG00000290034) | novel transcript, antisense to TREM2 | -4,11 |
| gene:ENSG00000290037 | [ENSG00000290037](https://www.ensembl.org/id/ENSG00000290037) | novel transcript, antisense to ACTA1 | -2,76 |
| gene:ENSG00000290101 | [ENSG00000290101](https://www.ensembl.org/id/ENSG00000290101) | novel transcript | -3,32 |
| gene:ENSG00000290114 | [ENSG00000290114](https://www.ensembl.org/id/ENSG00000290114) | novel transcript, antisense to FIGNL1 | -2,16 |
| gene:ENSG00000290315 | [ENSG00000290315](https://www.ensembl.org/id/ENSG00000290315) | novel protein | 2,92 |
| gene:ENSG00000290405 | [ENSG00000290405](https://www.ensembl.org/id/ENSG00000290405) | novel transcript | -4,68 |
| gene:ENSG00000290443 | [ENSG00000290443](https://www.ensembl.org/id/ENSG00000290443) | novel transcript | -5,43 |
| gene:ENSG00000290537 | [ENSG00000290537](https://www.ensembl.org/id/ENSG00000290537) | novel transcript | 1,79 |
| gene:ENSG00000290573 | [ENSG00000290573](https://www.ensembl.org/id/ENSG00000290573) | novel transcript | -4,39 |
| gene:ENSG00000290655 | [ENSG00000290655](https://www.ensembl.org/id/ENSG00000290655) | novel transcript | -8,19 |
| gene:ENSG00000290656 | [ENSG00000290656](https://www.ensembl.org/id/ENSG00000290656) | novel transcript | -5,19 |
| gene:ENSG00000290659 | [ENSG00000290659](https://www.ensembl.org/id/ENSG00000290659) | novel transcript | -3,89 |
| gene:ENSG00000290685 | [ENSG00000290685](https://www.ensembl.org/id/ENSG00000290685) | novel transcript | -6,06 |
| gene:ENSG00000290689 | [ENSG00000290689](https://www.ensembl.org/id/ENSG00000290689) | novel transcript | -2,19 |
| gene:ENSG00000290799 | [ENSG00000290799](https://www.ensembl.org/id/ENSG00000290799) | novel transcript | -4,49 |
| gene:ENSG00000290816 | [ENSG00000290816](https://www.ensembl.org/id/ENSG00000290816) | novel transcript | -3,58 |
| gene:ENSG00000290839 | [ENSG00000290839](https://www.ensembl.org/id/ENSG00000290839) | novel transcript | -2,72 |
| gene:ENSG00000290884 | [ENSG00000290884](https://www.ensembl.org/id/ENSG00000290884) | novel transcript | -5,38 |
| gene:ENSG00000290916 | [ENSG00000290916](https://www.ensembl.org/id/ENSG00000290916) | novel transcript | -2,92 |
| gene:ENSG00000290931 | [ENSG00000290931](https://www.ensembl.org/id/ENSG00000290931) | novel transcript | -2,02 |
| gene:ENSG00000290980 | [ENSG00000290980](https://www.ensembl.org/id/ENSG00000290980) | novel transcript | -10,52 |
| gene:ENSG00000290989 | [ENSG00000290989](https://www.ensembl.org/id/ENSG00000290989) | novel transcript | 1,02 |
| gene:ENSG00000290990 | [ENSG00000290990](https://www.ensembl.org/id/ENSG00000290990) | novel transcript | -3,64 |
| gene:ENSG00000290999 | [ENSG00000290999](https://www.ensembl.org/id/ENSG00000290999) | novel transcript | -1,44 |
| gene:ENSG00000291018 | [ENSG00000291018](https://www.ensembl.org/id/ENSG00000291018) | novel transcript | -3,49 |
| gene:ENSG00000291034 | [ENSG00000291034](https://www.ensembl.org/id/ENSG00000291034) | novel transcript | -4,08 |
| gene:ENSG00000291061 | [ENSG00000291061](https://www.ensembl.org/id/ENSG00000291061) | novel transcript | 5,22 |
| gene:ENSG00000291157 | [ENSG00000291157](https://www.ensembl.org/id/ENSG00000291157) | novel transcript | 3,89 |
| gene:ENSG00000291168 | [ENSG00000291168](https://www.ensembl.org/id/ENSG00000291168) | novel transcript | 3,18 |
| gene:ENSG00000291184 | [ENSG00000291184](https://www.ensembl.org/id/ENSG00000291184) | novel transcript | -4,98 |
| gene:ENSG00000291198 | [ENSG00000291198](https://www.ensembl.org/id/ENSG00000291198) | novel transcript | -5,22 |
| gene:ENSG00000291209 | [ENSG00000291209](https://www.ensembl.org/id/ENSG00000291209) | novel transcript | -5,84 |
| gene:ENSG00000291210 | [ENSG00000291210](https://www.ensembl.org/id/ENSG00000291210) | novel transcript | -6,23 |
| gene:ENSG00000291211 | [ENSG00000291211](https://www.ensembl.org/id/ENSG00000291211) | novel transcript | 2,01 |
| gene:ENSG00000291230 | [ENSG00000291230](https://www.ensembl.org/id/ENSG00000291230) | novel transcript | -2,85 |
| gene:ENSG00000291233 | [ENSG00000291233](https://www.ensembl.org/id/ENSG00000291233) | novel transcript | -4,92 |
| gene:ENSG00000291250 | [ENSG00000291250](https://www.ensembl.org/id/ENSG00000291250) | novel transcript | -4,68 |
| gene:ENSG00000291260 | [ENSG00000291260](https://www.ensembl.org/id/ENSG00000291260) | novel transcript | -5,69 |
| gene:ENSG00000291286 | [ENSG00000291286](https://www.ensembl.org/id/ENSG00000291286) | novel transcript | -4,68 |
| GFM1 | [ENSG00000168827](https://www.ensembl.org/id/ENSG00000168827) | G elongation factor mitochondrial 1 [Source:HGNC Symbol;Acc:HGNC:13780] | -1,07 |
| GHR | [ENSG00000112964](https://www.ensembl.org/id/ENSG00000112964) | growth hormone receptor [Source:HGNC Symbol;Acc:HGNC:4263] | -1,16 |
| GHRLOS | [ENSG00000240288](https://www.ensembl.org/id/ENSG00000240288) | ghrelin opposite strand/antisense RNA [Source:HGNC Symbol;Acc:HGNC:33885] | 1,69 |
| GINS1 | [ENSG00000101003](https://www.ensembl.org/id/ENSG00000101003) | GINS complex subunit 1 [Source:HGNC Symbol;Acc:HGNC:28980] | 2,27 |
| GINS2 | [ENSG00000131153](https://www.ensembl.org/id/ENSG00000131153) | GINS complex subunit 2 [Source:HGNC Symbol;Acc:HGNC:24575] | -1,81 |
| GIPC3 | [ENSG00000179855](https://www.ensembl.org/id/ENSG00000179855) | GIPC PDZ domain containing family member 3 [Source:HGNC Symbol;Acc:HGNC:18183] | 1,97 |
| GJA4 | [ENSG00000187513](https://www.ensembl.org/id/ENSG00000187513) | gap junction protein alpha 4 [Source:HGNC Symbol;Acc:HGNC:4278] | 1,62 |
| GJB2 | [ENSG00000165474](https://www.ensembl.org/id/ENSG00000165474) | gap junction protein beta 2 [Source:HGNC Symbol;Acc:HGNC:4284] | 2,45 |
| GJC3 | [ENSG00000176402](https://www.ensembl.org/id/ENSG00000176402) | gap junction protein gamma 3 [Source:HGNC Symbol;Acc:HGNC:17495] | -3,48 |
| GJD2 | [ENSG00000159248](https://www.ensembl.org/id/ENSG00000159248) | gap junction protein delta 2 [Source:HGNC Symbol;Acc:HGNC:19154] | -6,09 |
| GJD2-DT | [ENSG00000250007](https://www.ensembl.org/id/ENSG00000250007) | GJD2 divergent transcript [Source:HGNC Symbol;Acc:HGNC:55560] | 1,85 |
| GJD4 | [ENSG00000177291](https://www.ensembl.org/id/ENSG00000177291) | gap junction protein delta 4 [Source:HGNC Symbol;Acc:HGNC:23296] | -3,42 |
| GKAP1 | [ENSG00000165113](https://www.ensembl.org/id/ENSG00000165113) | G kinase anchoring protein 1 [Source:HGNC Symbol;Acc:HGNC:17496] | -1,20 |
| GLDC | [ENSG00000178445](https://www.ensembl.org/id/ENSG00000178445) | glycine decarboxylase [Source:HGNC Symbol;Acc:HGNC:4313] | -3,00 |
| GLDN | [ENSG00000186417](https://www.ensembl.org/id/ENSG00000186417) | gliomedin [Source:HGNC Symbol;Acc:HGNC:29514] | 2,02 |
| GLT1D1 | [ENSG00000151948](https://www.ensembl.org/id/ENSG00000151948) | glycosyltransferase 1 domain containing 1 [Source:HGNC Symbol;Acc:HGNC:26483] | -2,56 |
| GMPR | [ENSG00000137198](https://www.ensembl.org/id/ENSG00000137198) | guanosine monophosphate reductase [Source:HGNC Symbol;Acc:HGNC:4376] | -2,05 |
| GNAI1 | [ENSG00000127955](https://www.ensembl.org/id/ENSG00000127955) | G protein subunit alpha i1 [Source:HGNC Symbol;Acc:HGNC:4384] | 0,90 |
| GNB1-DT | [ENSG00000231050](https://www.ensembl.org/id/ENSG00000231050) | GNB1 divergent transcript [Source:HGNC Symbol;Acc:HGNC:55772] | -2,93 |
| GNE | [ENSG00000159921](https://www.ensembl.org/id/ENSG00000159921) | glucosamine (UDP-N-acetyl)-2-epimerase/N-acetylmannosamine kinase [Source:HGNC Symbol;Acc:HGNC:23657] | 0,85 |
| GNG5 | [ENSG00000174021](https://www.ensembl.org/id/ENSG00000174021) | G protein subunit gamma 5 [Source:HGNC Symbol;Acc:HGNC:4408] | -0,95 |
| GNPAT | [ENSG00000116906](https://www.ensembl.org/id/ENSG00000116906) | glyceronephosphate O-acyltransferase [Source:HGNC Symbol;Acc:HGNC:4416] | -1,23 |
| GOLGA4 | [ENSG00000144674](https://www.ensembl.org/id/ENSG00000144674) | golgin A4 [Source:HGNC Symbol;Acc:HGNC:4427] | -1,24 |
| GOLGA8S | [ENSG00000261739](https://www.ensembl.org/id/ENSG00000261739) | golgin A8 family member S [Source:HGNC Symbol;Acc:HGNC:44409] | -3,28 |
| GOT1 | [ENSG00000120053](https://www.ensembl.org/id/ENSG00000120053) | glutamic-oxaloacetic transaminase 1 [Source:HGNC Symbol;Acc:HGNC:4432] | -4,10 |
| GOT2 | [ENSG00000125166](https://www.ensembl.org/id/ENSG00000125166) | glutamic-oxaloacetic transaminase 2 [Source:HGNC Symbol;Acc:HGNC:4433] | -3,42 |
| GPA33 | [ENSG00000143167](https://www.ensembl.org/id/ENSG00000143167) | glycoprotein A33 [Source:HGNC Symbol;Acc:HGNC:4445] | -5,01 |
| GPAT3 | [ENSG00000138678](https://www.ensembl.org/id/ENSG00000138678) | glycerol-3-phosphate acyltransferase 3 [Source:HGNC Symbol;Acc:HGNC:28157] | -2,88 |
| GPC1-AS1 | [ENSG00000218416](https://www.ensembl.org/id/ENSG00000218416) | GPC1 antisense RNA 1 [Source:HGNC Symbol;Acc:HGNC:56102] | 3,82 |
| GPC6 | [ENSG00000183098](https://www.ensembl.org/id/ENSG00000183098) | glypican 6 [Source:HGNC Symbol;Acc:HGNC:4454] | 1,30 |
| GPD1 | [ENSG00000167588](https://www.ensembl.org/id/ENSG00000167588) | glycerol-3-phosphate dehydrogenase 1 [Source:HGNC Symbol;Acc:HGNC:4455] | -2,44 |
| GPER1 | [ENSG00000164850](https://www.ensembl.org/id/ENSG00000164850) | G protein-coupled estrogen receptor 1 [Source:HGNC Symbol;Acc:HGNC:4485] | 1,25 |
| GPR12 | [ENSG00000132975](https://www.ensembl.org/id/ENSG00000132975) | G protein-coupled receptor 12 [Source:HGNC Symbol;Acc:HGNC:4466] | -2,89 |
| GPR157 | [ENSG00000180758](https://www.ensembl.org/id/ENSG00000180758) | G protein-coupled receptor 157 [Source:HGNC Symbol;Acc:HGNC:23687] | -2,63 |
| GPR179 | [ENSG00000277399](https://www.ensembl.org/id/ENSG00000277399) | G protein-coupled receptor 179 [Source:HGNC Symbol;Acc:HGNC:31371] | -2,31 |
| GPR182 | [ENSG00000166856](https://www.ensembl.org/id/ENSG00000166856) | G protein-coupled receptor 182 [Source:HGNC Symbol;Acc:HGNC:13708] | 2,83 |
| GPR20 | [ENSG00000204882](https://www.ensembl.org/id/ENSG00000204882) | G protein-coupled receptor 20 [Source:HGNC Symbol;Acc:HGNC:4475] | 2,55 |
| GPR26 | [ENSG00000154478](https://www.ensembl.org/id/ENSG00000154478) | G protein-coupled receptor 26 [Source:HGNC Symbol;Acc:HGNC:4481] | 2,56 |
| GPR3 | [ENSG00000181773](https://www.ensembl.org/id/ENSG00000181773) | G protein-coupled receptor 3 [Source:HGNC Symbol;Acc:HGNC:4484] | 2,27 |
| GPR6 | [ENSG00000146360](https://www.ensembl.org/id/ENSG00000146360) | G protein-coupled receptor 6 [Source:HGNC Symbol;Acc:HGNC:4515] | -5,38 |
| GPR61 | [ENSG00000156097](https://www.ensembl.org/id/ENSG00000156097) | G protein-coupled receptor 61 [Source:HGNC Symbol;Acc:HGNC:13300] | -2,94 |
| GPRC5B | [ENSG00000167191](https://www.ensembl.org/id/ENSG00000167191) | G protein-coupled receptor class C group 5 member B [Source:HGNC Symbol;Acc:HGNC:13308] | -2,09 |
| GPSM2 | [ENSG00000121957](https://www.ensembl.org/id/ENSG00000121957) | G protein signaling modulator 2 [Source:HGNC Symbol;Acc:HGNC:29501] | -1,68 |
| GPT | [ENSG00000167701](https://www.ensembl.org/id/ENSG00000167701) | glutamic--pyruvic transaminase [Source:HGNC Symbol;Acc:HGNC:4552] | -5,32 |
| GPT2 | [ENSG00000166123](https://www.ensembl.org/id/ENSG00000166123) | glutamic--pyruvic transaminase 2 [Source:HGNC Symbol;Acc:HGNC:18062] | -6,17 |
| GRAMD1C | [ENSG00000178075](https://www.ensembl.org/id/ENSG00000178075) | GRAM domain containing 1C [Source:HGNC Symbol;Acc:HGNC:25252] | -1,88 |
| GREB1 | [ENSG00000196208](https://www.ensembl.org/id/ENSG00000196208) | growth regulating estrogen receptor binding 1 [Source:HGNC Symbol;Acc:HGNC:24885] | -2,77 |
| GRIA1 | [ENSG00000155511](https://www.ensembl.org/id/ENSG00000155511) | glutamate ionotropic receptor AMPA type subunit 1 [Source:HGNC Symbol;Acc:HGNC:4571] | -2,41 |
| GRIA3 | [ENSG00000125675](https://www.ensembl.org/id/ENSG00000125675) | glutamate ionotropic receptor AMPA type subunit 3 [Source:HGNC Symbol;Acc:HGNC:4573] | 1,51 |
| GRIP1 | [ENSG00000155974](https://www.ensembl.org/id/ENSG00000155974) | glutamate receptor interacting protein 1 [Source:HGNC Symbol;Acc:HGNC:18708] | -2,41 |
| GRIP2 | [ENSG00000144596](https://www.ensembl.org/id/ENSG00000144596) | glutamate receptor interacting protein 2 [Source:HGNC Symbol;Acc:HGNC:23841] | -2,66 |
| GRK7 | [ENSG00000114124](https://www.ensembl.org/id/ENSG00000114124) | G protein-coupled receptor kinase 7 [Source:HGNC Symbol;Acc:HGNC:17031] | -2,89 |
| GRSF1 | [ENSG00000132463](https://www.ensembl.org/id/ENSG00000132463) | G-rich RNA sequence binding factor 1 [Source:HGNC Symbol;Acc:HGNC:4610] | -1,15 |
| GRTP1-AS1 | [ENSG00000225083](https://www.ensembl.org/id/ENSG00000225083) | GRTP1 antisense RNA 1 [Source:HGNC Symbol;Acc:HGNC:39917] | -4,20 |
| GSDMC | [ENSG00000147697](https://www.ensembl.org/id/ENSG00000147697) | gasdermin C [Source:HGNC Symbol;Acc:HGNC:7151] | -8,06 |
| GSR | [ENSG00000104687](https://www.ensembl.org/id/ENSG00000104687) | glutathione-disulfide reductase [Source:HGNC Symbol;Acc:HGNC:4623] | -1,01 |
| GSTZ1 | [ENSG00000100577](https://www.ensembl.org/id/ENSG00000100577) | glutathione S-transferase zeta 1 [Source:HGNC Symbol;Acc:HGNC:4643] | -1,75 |
| GTF2IRD1 | [ENSG00000006704](https://www.ensembl.org/id/ENSG00000006704) | GTF2I repeat domain containing 1 [Source:HGNC Symbol;Acc:HGNC:4661] | -1,57 |
| GTF2IRD1P1 | [ENSG00000291206](https://www.ensembl.org/id/ENSG00000291206) | GTF2I repeat domain containing 1 pseudogene 1 [Source:HGNC Symbol;Acc:HGNC:44136] | -4,72 |
| GTF3A | [ENSG00000122034](https://www.ensembl.org/id/ENSG00000122034) | general transcription factor IIIA [Source:HGNC Symbol;Acc:HGNC:4662] | -1,45 |
| GTF3C6 | [ENSG00000155115](https://www.ensembl.org/id/ENSG00000155115) | general transcription factor IIIC subunit 6 [Source:HGNC Symbol;Acc:HGNC:20872] | -1,36 |
| GTSF1 | [ENSG00000170627](https://www.ensembl.org/id/ENSG00000170627) | gametocyte specific factor 1 [Source:HGNC Symbol;Acc:HGNC:26565] | -4,35 |
| GUCY1B1 | [ENSG00000061918](https://www.ensembl.org/id/ENSG00000061918) | guanylate cyclase 1 soluble subunit beta 1 [Source:HGNC Symbol;Acc:HGNC:4687] | 1,33 |
| GUF1 | [ENSG00000151806](https://www.ensembl.org/id/ENSG00000151806) | GTP binding elongation factor GUF1 [Source:HGNC Symbol;Acc:HGNC:25799] | -1,80 |
| GYG1 | [ENSG00000163754](https://www.ensembl.org/id/ENSG00000163754) | glycogenin 1 [Source:HGNC Symbol;Acc:HGNC:4699] | -1,57 |
| GYS1 | [ENSG00000104812](https://www.ensembl.org/id/ENSG00000104812) | glycogen synthase 1 [Source:HGNC Symbol;Acc:HGNC:4706] | -2,45 |
| H19 | [ENSG00000130600](https://www.ensembl.org/id/ENSG00000130600) | H19 imprinted maternally expressed transcript [Source:HGNC Symbol;Acc:HGNC:4713] | -3,48 |
| H2AC21 | [ENSG00000184270](https://www.ensembl.org/id/ENSG00000184270) | H2A clustered histone 21 [Source:HGNC Symbol;Acc:HGNC:20508] | -4,13 |
| HADH | [ENSG00000138796](https://www.ensembl.org/id/ENSG00000138796) | hydroxyacyl-CoA dehydrogenase [Source:HGNC Symbol;Acc:HGNC:4799] | -1,21 |
| HADHA | [ENSG00000084754](https://www.ensembl.org/id/ENSG00000084754) | hydroxyacyl-CoA dehydrogenase trifunctional multienzyme complex subunit alpha [Source:HGNC Symbol;Acc:HGNC:4801] | -1,45 |
| HADHB | [ENSG00000138029](https://www.ensembl.org/id/ENSG00000138029) | hydroxyacyl-CoA dehydrogenase trifunctional multienzyme complex subunit beta [Source:HGNC Symbol;Acc:HGNC:4803] | -2,90 |
| HAGH | [ENSG00000063854](https://www.ensembl.org/id/ENSG00000063854) | hydroxyacylglutathione hydrolase [Source:HGNC Symbol;Acc:HGNC:4805] | -1,20 |
| HAP1 | [ENSG00000173805](https://www.ensembl.org/id/ENSG00000173805) | huntingtin associated protein 1 [Source:HGNC Symbol;Acc:HGNC:4812] | -3,43 |
| HAPLN2 | [ENSG00000132702](https://www.ensembl.org/id/ENSG00000132702) | hyaluronan and proteoglycan link protein 2 [Source:HGNC Symbol;Acc:HGNC:17410] | 1,60 |
| HAS1 | [ENSG00000105509](https://www.ensembl.org/id/ENSG00000105509) | hyaluronan synthase 1 [Source:HGNC Symbol;Acc:HGNC:4818] | 2,38 |
| HASPIN | [ENSG00000177602](https://www.ensembl.org/id/ENSG00000177602) | histone H3 associated protein kinase [Source:HGNC Symbol;Acc:HGNC:19682] | -3,23 |
| HBD | [ENSG00000223609](https://www.ensembl.org/id/ENSG00000223609) | hemoglobin subunit delta [Source:HGNC Symbol;Acc:HGNC:4829] | 4,85 |
| HBG1 | [ENSG00000213934](https://www.ensembl.org/id/ENSG00000213934) | hemoglobin subunit gamma 1 [Source:HGNC Symbol;Acc:HGNC:4831] | -6,75 |
| HBG2 | [ENSG00000196565](https://www.ensembl.org/id/ENSG00000196565) | hemoglobin subunit gamma 2 [Source:HGNC Symbol;Acc:HGNC:4832] | 4,36 |
| HBM | [ENSG00000206177](https://www.ensembl.org/id/ENSG00000206177) | hemoglobin subunit mu [Source:HGNC Symbol;Acc:HGNC:4826] | 3,91 |
| HBS1L | [ENSG00000112339](https://www.ensembl.org/id/ENSG00000112339) | HBS1 like translational GTPase [Source:HGNC Symbol;Acc:HGNC:4834] | -1,25 |
| HCCS | [ENSG00000004961](https://www.ensembl.org/id/ENSG00000004961) | holocytochrome c synthase [Source:HGNC Symbol;Acc:HGNC:4837] | -1,14 |
| HCN1 | [ENSG00000164588](https://www.ensembl.org/id/ENSG00000164588) | hyperpolarization activated cyclic nucleotide gated potassium channel 1 [Source:HGNC Symbol;Acc:HGNC:4845] | -4,03 |
| HDDC2 | [ENSG00000111906](https://www.ensembl.org/id/ENSG00000111906) | HD domain containing 2 [Source:HGNC Symbol;Acc:HGNC:21078] | -1,09 |
| HDHD5 | [ENSG00000069998](https://www.ensembl.org/id/ENSG00000069998) | haloacid dehalogenase like hydrolase domain containing 5 [Source:HGNC Symbol;Acc:HGNC:1843] | -1,42 |
| HECW1 | [ENSG00000002746](https://www.ensembl.org/id/ENSG00000002746) | HECT, C2 and WW domain containing E3 ubiquitin protein ligase 1 [Source:HGNC Symbol;Acc:HGNC:22195] | -5,70 |
| HES4 | [ENSG00000188290](https://www.ensembl.org/id/ENSG00000188290) | hes family bHLH transcription factor 4 [Source:HGNC Symbol;Acc:HGNC:24149] | 1,40 |
| HEY2 | [ENSG00000135547](https://www.ensembl.org/id/ENSG00000135547) | hes related family bHLH transcription factor with YRPW motif 2 [Source:HGNC Symbol;Acc:HGNC:4881] | 1,24 |
| HHATL | [ENSG00000010282](https://www.ensembl.org/id/ENSG00000010282) | hedgehog acyltransferase like [Source:HGNC Symbol;Acc:HGNC:13242] | -8,46 |
| HIBADH | [ENSG00000106049](https://www.ensembl.org/id/ENSG00000106049) | 3-hydroxyisobutyrate dehydrogenase [Source:HGNC Symbol;Acc:HGNC:4907] | -1,45 |
| HIBCH | [ENSG00000198130](https://www.ensembl.org/id/ENSG00000198130) | 3-hydroxyisobutyryl-CoA hydrolase [Source:HGNC Symbol;Acc:HGNC:4908] | -1,07 |
| HIGD1B | [ENSG00000131097](https://www.ensembl.org/id/ENSG00000131097) | HIG1 hypoxia inducible domain family member 1B [Source:HGNC Symbol;Acc:HGNC:24318] | -3,85 |
| HIGD2A | [ENSG00000146066](https://www.ensembl.org/id/ENSG00000146066) | HIG1 hypoxia inducible domain family member 2A [Source:HGNC Symbol;Acc:HGNC:28311] | -1,79 |
| HINT3 | [ENSG00000111911](https://www.ensembl.org/id/ENSG00000111911) | histidine triad nucleotide binding protein 3 [Source:HGNC Symbol;Acc:HGNC:18468] | -1,23 |
| HIP1 | [ENSG00000127946](https://www.ensembl.org/id/ENSG00000127946) | huntingtin interacting protein 1 [Source:HGNC Symbol;Acc:HGNC:4913] | 0,99 |
| HISLA | [ENSG00000258867](https://www.ensembl.org/id/ENSG00000258867) | HIF1A stabilizing long noncoding RNA [Source:HGNC Symbol;Acc:HGNC:49467] | -4,49 |
| HJV | [ENSG00000168509](https://www.ensembl.org/id/ENSG00000168509) | hemojuvelin BMP co-receptor [Source:HGNC Symbol;Acc:HGNC:4887] | -9,88 |
| HLA-C | [ENSG00000204525](https://www.ensembl.org/id/ENSG00000204525) | major histocompatibility complex, class I, C [Source:HGNC Symbol;Acc:HGNC:4933] | 2,90 |
| HLA-DPA1 | [ENSG00000231389](https://www.ensembl.org/id/ENSG00000231389) | major histocompatibility complex, class II, DP alpha 1 [Source:HGNC Symbol;Acc:HGNC:4938] | -1,49 |
| HLA-DRA | [ENSG00000204287](https://www.ensembl.org/id/ENSG00000204287) | major histocompatibility complex, class II, DR alpha [Source:HGNC Symbol;Acc:HGNC:4947] | -1,54 |
| HLA-DRB1 | [ENSG00000196126](https://www.ensembl.org/id/ENSG00000196126) | major histocompatibility complex, class II, DR beta 1 [Source:HGNC Symbol;Acc:HGNC:4948] | 3,05 |
| HLA-DRB5 | [ENSG00000198502](https://www.ensembl.org/id/ENSG00000198502) | major histocompatibility complex, class II, DR beta 5 [Source:HGNC Symbol;Acc:HGNC:4953] | 8,53 |
| HMBS | [ENSG00000256269](https://www.ensembl.org/id/ENSG00000256269) | hydroxymethylbilane synthase [Source:HGNC Symbol;Acc:HGNC:4982] | -1,45 |
| HMGA1 | [ENSG00000137309](https://www.ensembl.org/id/ENSG00000137309) | high mobility group AT-hook 1 [Source:HGNC Symbol;Acc:HGNC:5010] | -1,72 |
| HMGCS2 | [ENSG00000134240](https://www.ensembl.org/id/ENSG00000134240) | 3-hydroxy-3-methylglutaryl-CoA synthase 2 [Source:HGNC Symbol;Acc:HGNC:5008] | -10,26 |
| HMHB1 | [ENSG00000158497](https://www.ensembl.org/id/ENSG00000158497) | histocompatibility minor HB-1 [Source:HGNC Symbol;Acc:HGNC:29677] | -4,89 |
| HMX1 | [ENSG00000215612](https://www.ensembl.org/id/ENSG00000215612) | H6 family homeobox 1 [Source:HGNC Symbol;Acc:HGNC:5017] | -6,52 |
| HOMER2 | [ENSG00000103942](https://www.ensembl.org/id/ENSG00000103942) | homer scaffold protein 2 [Source:HGNC Symbol;Acc:HGNC:17513] | -4,71 |
| HOMER3 | [ENSG00000051128](https://www.ensembl.org/id/ENSG00000051128) | homer scaffold protein 3 [Source:HGNC Symbol;Acc:HGNC:17514] | -2,14 |
| HOMER3-AS1 | [ENSG00000269019](https://www.ensembl.org/id/ENSG00000269019) | HOMER3 antisense RNA 1 [Source:HGNC Symbol;Acc:HGNC:53775] | -4,32 |
| HOOK2 | [ENSG00000095066](https://www.ensembl.org/id/ENSG00000095066) | hook microtubule tethering protein 2 [Source:HGNC Symbol;Acc:HGNC:19885] | -1,05 |
| HOXB8 | [ENSG00000120068](https://www.ensembl.org/id/ENSG00000120068) | homeobox B8 [Source:HGNC Symbol;Acc:HGNC:5119] | -2,74 |
| HOXC10 | [ENSG00000180818](https://www.ensembl.org/id/ENSG00000180818) | homeobox C10 [Source:HGNC Symbol;Acc:HGNC:5122] | -2,49 |
| HPN | [ENSG00000105707](https://www.ensembl.org/id/ENSG00000105707) | hepsin [Source:HGNC Symbol;Acc:HGNC:5155] | -4,69 |
| HR | [ENSG00000168453](https://www.ensembl.org/id/ENSG00000168453) | HR lysine demethylase and nuclear receptor corepressor [Source:HGNC Symbol;Acc:HGNC:5172] | 1,74 |
| HRC | [ENSG00000130528](https://www.ensembl.org/id/ENSG00000130528) | histidine rich calcium binding protein [Source:HGNC Symbol;Acc:HGNC:5178] | -2,30 |
| HRCT1 | [ENSG00000196196](https://www.ensembl.org/id/ENSG00000196196) | histidine rich carboxyl terminus 1 [Source:HGNC Symbol;Acc:HGNC:33872] | 1,68 |
| HRG-AS1 | [ENSG00000197099](https://www.ensembl.org/id/ENSG00000197099) | HRG and FETUB antisense RNA 1 [Source:HGNC Symbol;Acc:HGNC:55915] | -6,02 |
| HRH2 | [ENSG00000113749](https://www.ensembl.org/id/ENSG00000113749) | histamine receptor H2 [Source:HGNC Symbol;Acc:HGNC:5183] | 1,81 |
| HS1BP3 | [ENSG00000118960](https://www.ensembl.org/id/ENSG00000118960) | HCLS1 binding protein 3 [Source:HGNC Symbol;Acc:HGNC:24979] | 0,93 |
| HS3ST5 | [ENSG00000249853](https://www.ensembl.org/id/ENSG00000249853) | heparan sulfate-glucosamine 3-sulfotransferase 5 [Source:HGNC Symbol;Acc:HGNC:19419] | -3,44 |
| HS6ST1 | [ENSG00000136720](https://www.ensembl.org/id/ENSG00000136720) | heparan sulfate 6-O-sulfotransferase 1 [Source:HGNC Symbol;Acc:HGNC:5201] | -1,06 |
| HS6ST2 | [ENSG00000171004](https://www.ensembl.org/id/ENSG00000171004) | heparan sulfate 6-O-sulfotransferase 2 [Source:HGNC Symbol;Acc:HGNC:19133] | -5,22 |
| HSBP1L1 | [ENSG00000226742](https://www.ensembl.org/id/ENSG00000226742) | heat shock factor binding protein 1 like 1 [Source:HGNC Symbol;Acc:HGNC:37243] | -1,67 |
| HSD17B2 | [ENSG00000086696](https://www.ensembl.org/id/ENSG00000086696) | hydroxysteroid 17-beta dehydrogenase 2 [Source:HGNC Symbol;Acc:HGNC:5211] | -3,35 |
| HSD17B6 | [ENSG00000025423](https://www.ensembl.org/id/ENSG00000025423) | hydroxysteroid 17-beta dehydrogenase 6 [Source:HGNC Symbol;Acc:HGNC:23316] | 3,45 |
| HSD17B8 | [ENSG00000204228](https://www.ensembl.org/id/ENSG00000204228) | hydroxysteroid 17-beta dehydrogenase 8 [Source:HGNC Symbol;Acc:HGNC:3554] | -1,34 |
| HSDL2 | [ENSG00000119471](https://www.ensembl.org/id/ENSG00000119471) | hydroxysteroid dehydrogenase like 2 [Source:HGNC Symbol;Acc:HGNC:18572] | -1,60 |
| HSFX1 | [ENSG00000171116](https://www.ensembl.org/id/ENSG00000171116) | heat shock transcription factor family, X-linked 1 [Source:HGNC Symbol;Acc:HGNC:29603] | 8,49 |
| HSFX2 | [ENSG00000268738](https://www.ensembl.org/id/ENSG00000268738) | heat shock transcription factor family, X-linked 2 [Source:HGNC Symbol;Acc:HGNC:32701] | -8,19 |
| HSFX4 | [ENSG00000283463](https://www.ensembl.org/id/ENSG00000283463) | heat shock transcription factor family, X-linked member 4 [Source:HGNC Symbol;Acc:HGNC:52398] | 4,62 |
| HSPA2 | [ENSG00000126803](https://www.ensembl.org/id/ENSG00000126803) | heat shock protein family A (Hsp70) member 2 [Source:HGNC Symbol;Acc:HGNC:5235] | -1,77 |
| HSPA4L | [ENSG00000164070](https://www.ensembl.org/id/ENSG00000164070) | heat shock protein family A (Hsp70) member 4 like [Source:HGNC Symbol;Acc:HGNC:17041] | 1,27 |
| HSPB2 | [ENSG00000170276](https://www.ensembl.org/id/ENSG00000170276) | heat shock protein family B (small) member 2 [Source:HGNC Symbol;Acc:HGNC:5247] | -1,22 |
| HSPB3 | [ENSG00000169271](https://www.ensembl.org/id/ENSG00000169271) | heat shock protein family B (small) member 3 [Source:HGNC Symbol;Acc:HGNC:5248] | -3,77 |
| HSPB6 | [ENSG00000004776](https://www.ensembl.org/id/ENSG00000004776) | heat shock protein family B (small) member 6 [Source:HGNC Symbol;Acc:HGNC:26511] | -2,33 |
| HTATSF1 | [ENSG00000102241](https://www.ensembl.org/id/ENSG00000102241) | HIV-1 Tat specific factor 1 [Source:HGNC Symbol;Acc:HGNC:5276] | -1,55 |
| HTN3 | [ENSG00000205649](https://www.ensembl.org/id/ENSG00000205649) | histatin 3 [Source:HGNC Symbol;Acc:HGNC:5284] | -7,71 |
| HTR2A | [ENSG00000102468](https://www.ensembl.org/id/ENSG00000102468) | 5-hydroxytryptamine receptor 2A [Source:HGNC Symbol;Acc:HGNC:5293] | 1,76 |
| HTR7 | [ENSG00000148680](https://www.ensembl.org/id/ENSG00000148680) | 5-hydroxytryptamine receptor 7 [Source:HGNC Symbol;Acc:HGNC:5302] | -2,20 |
| HYAL4 | [ENSG00000106302](https://www.ensembl.org/id/ENSG00000106302) | hyaluronidase 4 [Source:HGNC Symbol;Acc:HGNC:5323] | -4,79 |
| IBA57 | [ENSG00000181873](https://www.ensembl.org/id/ENSG00000181873) | iron-sulfur cluster assembly factor IBA57 [Source:HGNC Symbol;Acc:HGNC:27302] | -1,51 |
| IBSP | [ENSG00000029559](https://www.ensembl.org/id/ENSG00000029559) | integrin binding sialoprotein [Source:HGNC Symbol;Acc:HGNC:5341] | -5,33 |
| IBTK | [ENSG00000005700](https://www.ensembl.org/id/ENSG00000005700) | inhibitor of Bruton tyrosine kinase [Source:HGNC Symbol;Acc:HGNC:17853] | -1,01 |
| ID1 | [ENSG00000125968](https://www.ensembl.org/id/ENSG00000125968) | inhibitor of DNA binding 1 [Source:HGNC Symbol;Acc:HGNC:5360] | 1,80 |
| ID2 | [ENSG00000115738](https://www.ensembl.org/id/ENSG00000115738) | inhibitor of DNA binding 2 [Source:HGNC Symbol;Acc:HGNC:5361] | 2,03 |
| ID4 | [ENSG00000172201](https://www.ensembl.org/id/ENSG00000172201) | inhibitor of DNA binding 4 [Source:HGNC Symbol;Acc:HGNC:5363] | 1,68 |
| IDH2 | [ENSG00000182054](https://www.ensembl.org/id/ENSG00000182054) | isocitrate dehydrogenase (NADP(+)) 2 [Source:HGNC Symbol;Acc:HGNC:5383] | -3,89 |
| IDI2 | [ENSG00000148377](https://www.ensembl.org/id/ENSG00000148377) | isopentenyl-diphosphate delta isomerase 2 [Source:HGNC Symbol;Acc:HGNC:23487] | -14,08 |
| IDI2-AS1 | [ENSG00000232656](https://www.ensembl.org/id/ENSG00000232656) | IDI2 antisense RNA 1 [Source:HGNC Symbol;Acc:HGNC:30885] | -2,31 |
| IDO1 | [ENSG00000131203](https://www.ensembl.org/id/ENSG00000131203) | indoleamine 2,3-dioxygenase 1 [Source:HGNC Symbol;Acc:HGNC:6059] | -5,30 |
| IFFO2 | [ENSG00000169991](https://www.ensembl.org/id/ENSG00000169991) | intermediate filament family orphan 2 [Source:HGNC Symbol;Acc:HGNC:27006] | 1,24 |
| IFIT1 | [ENSG00000185745](https://www.ensembl.org/id/ENSG00000185745) | interferon induced protein with tetratricopeptide repeats 1 [Source:HGNC Symbol;Acc:HGNC:5407] | -1,41 |
| IFNG-AS1 | [ENSG00000255733](https://www.ensembl.org/id/ENSG00000255733) | IFNG antisense RNA 1 [Source:HGNC Symbol;Acc:HGNC:43910] | 2,69 |
| IFNLR1 | [ENSG00000185436](https://www.ensembl.org/id/ENSG00000185436) | interferon lambda receptor 1 [Source:HGNC Symbol;Acc:HGNC:18584] | -3,25 |
| IGDCC4 | [ENSG00000103742](https://www.ensembl.org/id/ENSG00000103742) | immunoglobulin superfamily DCC subclass member 4 [Source:HGNC Symbol;Acc:HGNC:13770] | -3,64 |
| IGF2BP1 | [ENSG00000159217](https://www.ensembl.org/id/ENSG00000159217) | insulin like growth factor 2 mRNA binding protein 1 [Source:HGNC Symbol;Acc:HGNC:28866] | -6,27 |
| IGFBPL1 | [ENSG00000137142](https://www.ensembl.org/id/ENSG00000137142) | insulin like growth factor binding protein like 1 [Source:HGNC Symbol;Acc:HGNC:20081] | 2,13 |
| IGFL2-AS1 | [ENSG00000268621](https://www.ensembl.org/id/ENSG00000268621) | IGFL2 antisense RNA 1 [Source:HGNC Symbol;Acc:HGNC:52559] | -6,33 |
| IGFN1 | [ENSG00000163395](https://www.ensembl.org/id/ENSG00000163395) | immunoglobulin like and fibronectin type III domain containing 1 [Source:HGNC Symbol;Acc:HGNC:24607] | -9,81 |
| IGSF1 | [ENSG00000147255](https://www.ensembl.org/id/ENSG00000147255) | immunoglobulin superfamily member 1 [Source:HGNC Symbol;Acc:HGNC:5948] | -3,98 |
| IGSF11 | [ENSG00000144847](https://www.ensembl.org/id/ENSG00000144847) | immunoglobulin superfamily member 11 [Source:HGNC Symbol;Acc:HGNC:16669] | -4,78 |
| IGSF21 | [ENSG00000117154](https://www.ensembl.org/id/ENSG00000117154) | immunoglobin superfamily member 21 [Source:HGNC Symbol;Acc:HGNC:28246] | -2,23 |
| IL12RB2 | [ENSG00000081985](https://www.ensembl.org/id/ENSG00000081985) | interleukin 12 receptor subunit beta 2 [Source:HGNC Symbol;Acc:HGNC:5972] | -2,81 |
| IL17D | [ENSG00000172458](https://www.ensembl.org/id/ENSG00000172458) | interleukin 17D [Source:HGNC Symbol;Acc:HGNC:5984] | -4,57 |
| IL17RE | [ENSG00000163701](https://www.ensembl.org/id/ENSG00000163701) | interleukin 17 receptor E [Source:HGNC Symbol;Acc:HGNC:18439] | 1,26 |
| IL1RAPL1 | [ENSG00000169306](https://www.ensembl.org/id/ENSG00000169306) | interleukin 1 receptor accessory protein like 1 [Source:HGNC Symbol;Acc:HGNC:5996] | -6,59 |
| IL20RA | [ENSG00000016402](https://www.ensembl.org/id/ENSG00000016402) | interleukin 20 receptor subunit alpha [Source:HGNC Symbol;Acc:HGNC:6003] | -3,03 |
| IL24 | [ENSG00000162892](https://www.ensembl.org/id/ENSG00000162892) | interleukin 24 [Source:HGNC Symbol;Acc:HGNC:11346] | -3,24 |
| IL31RA | [ENSG00000164509](https://www.ensembl.org/id/ENSG00000164509) | interleukin 31 receptor A [Source:HGNC Symbol;Acc:HGNC:18969] | -6,08 |
| IL32 | [ENSG00000008517](https://www.ensembl.org/id/ENSG00000008517) | interleukin 32 [Source:HGNC Symbol;Acc:HGNC:16830] | -2,47 |
| ILDR2 | [ENSG00000143195](https://www.ensembl.org/id/ENSG00000143195) | immunoglobulin like domain containing receptor 2 [Source:HGNC Symbol;Acc:HGNC:18131] | -3,56 |
| IMPA2 | [ENSG00000141401](https://www.ensembl.org/id/ENSG00000141401) | inositol monophosphatase 2 [Source:HGNC Symbol;Acc:HGNC:6051] | -2,28 |
| INAFM2 | [ENSG00000259330](https://www.ensembl.org/id/ENSG00000259330) | InaF motif containing 2 [Source:HGNC Symbol;Acc:HGNC:35165] | 1,33 |
| INHBA | [ENSG00000122641](https://www.ensembl.org/id/ENSG00000122641) | inhibin subunit beta A [Source:HGNC Symbol;Acc:HGNC:6066] | 2,19 |
| INHBA-AS1 | [ENSG00000224116](https://www.ensembl.org/id/ENSG00000224116) | INHBA antisense RNA 1 [Source:HGNC Symbol;Acc:HGNC:40303] | 2,16 |
| INHBE | [ENSG00000139269](https://www.ensembl.org/id/ENSG00000139269) | inhibin subunit beta E [Source:HGNC Symbol;Acc:HGNC:24029] | -3,57 |
| INPP5B | [ENSG00000204084](https://www.ensembl.org/id/ENSG00000204084) | inositol polyphosphate-5-phosphatase B [Source:HGNC Symbol;Acc:HGNC:6077] | 0,93 |
| INSC | [ENSG00000188487](https://www.ensembl.org/id/ENSG00000188487) | INSC spindle orientation adaptor protein [Source:HGNC Symbol;Acc:HGNC:33116] | 2,52 |
| INSL6 | [ENSG00000120210](https://www.ensembl.org/id/ENSG00000120210) | insulin like 6 [Source:HGNC Symbol;Acc:HGNC:6089] | 3,95 |
| IP6K3 | [ENSG00000161896](https://www.ensembl.org/id/ENSG00000161896) | inositol hexakisphosphate kinase 3 [Source:HGNC Symbol;Acc:HGNC:17269] | -3,58 |
| IPO13 | [ENSG00000117408](https://www.ensembl.org/id/ENSG00000117408) | importin 13 [Source:HGNC Symbol;Acc:HGNC:16853] | -1,72 |
| IQCH-AS1 | [ENSG00000259673](https://www.ensembl.org/id/ENSG00000259673) | IQCH antisense RNA 1 [Source:HGNC Symbol;Acc:HGNC:44104] | -1,41 |
| IQCJ-SCHIP1 | [ENSG00000283154](https://www.ensembl.org/id/ENSG00000283154) | IQCJ-SCHIP1 readthrough [Source:HGNC Symbol;Acc:HGNC:38842] | 1,71 |
| IQSEC3-AS1 | [ENSG00000256540](https://www.ensembl.org/id/ENSG00000256540) | IQSEC3 antisense RNA 1 [Source:HGNC Symbol;Acc:HGNC:56002] | -4,02 |
| IRAIN | [ENSG00000259424](https://www.ensembl.org/id/ENSG00000259424) | IGF1R antisense imprinted non-protein coding RNA [Source:HGNC Symbol;Acc:HGNC:50365] | 2,97 |
| IRF2BPL | [ENSG00000119669](https://www.ensembl.org/id/ENSG00000119669) | interferon regulatory factor 2 binding protein like [Source:HGNC Symbol;Acc:HGNC:14282] | 0,93 |
| IRX4-AS1 | [ENSG00000249116](https://www.ensembl.org/id/ENSG00000249116) | IRX4 antisense RNA 1 [Source:HGNC Symbol;Acc:HGNC:40305] | -7,70 |
| IRX6 | [ENSG00000159387](https://www.ensembl.org/id/ENSG00000159387) | iroquois homeobox 6 [Source:HGNC Symbol;Acc:HGNC:14675] | -2,90 |
| ISCA2 | [ENSG00000165898](https://www.ensembl.org/id/ENSG00000165898) | iron-sulfur cluster assembly 2 [Source:HGNC Symbol;Acc:HGNC:19857] | -1,91 |
| ISG20L2 | [ENSG00000143319](https://www.ensembl.org/id/ENSG00000143319) | interferon stimulated exonuclease gene 20 like 2 [Source:HGNC Symbol;Acc:HGNC:25745] | 1,05 |
| ISOC2 | [ENSG00000063241](https://www.ensembl.org/id/ENSG00000063241) | isochorismatase domain containing 2 [Source:HGNC Symbol;Acc:HGNC:26278] | -1,23 |
| ITGB1BP2 | [ENSG00000147166](https://www.ensembl.org/id/ENSG00000147166) | integrin subunit beta 1 binding protein 2 [Source:HGNC Symbol;Acc:HGNC:6154] | -1,21 |
| ITGB3 | [ENSG00000259207](https://www.ensembl.org/id/ENSG00000259207) | integrin subunit beta 3 [Source:HGNC Symbol;Acc:HGNC:6156] | 1,32 |
| ITGB6 | [ENSG00000115221](https://www.ensembl.org/id/ENSG00000115221) | integrin subunit beta 6 [Source:HGNC Symbol;Acc:HGNC:6161] | -13,26 |
| ITIH1 | [ENSG00000055957](https://www.ensembl.org/id/ENSG00000055957) | inter-alpha-trypsin inhibitor heavy chain 1 [Source:HGNC Symbol;Acc:HGNC:6166] | 2,22 |
| ITIH3 | [ENSG00000162267](https://www.ensembl.org/id/ENSG00000162267) | inter-alpha-trypsin inhibitor heavy chain 3 [Source:HGNC Symbol;Acc:HGNC:6168] | 2,52 |
| ITIH5 | [ENSG00000123243](https://www.ensembl.org/id/ENSG00000123243) | inter-alpha-trypsin inhibitor heavy chain 5 [Source:HGNC Symbol;Acc:HGNC:21449] | 1,69 |
| IZUMO3 | [ENSG00000205442](https://www.ensembl.org/id/ENSG00000205442) | IZUMO family member 3 [Source:HGNC Symbol;Acc:HGNC:31421] | -6,73 |
| JAG1 | [ENSG00000101384](https://www.ensembl.org/id/ENSG00000101384) | jagged canonical Notch ligand 1 [Source:HGNC Symbol;Acc:HGNC:6188] | 1,39 |
| JPH1 | [ENSG00000104369](https://www.ensembl.org/id/ENSG00000104369) | junctophilin 1 [Source:HGNC Symbol;Acc:HGNC:14201] | -7,43 |
| JPT1 | [ENSG00000189159](https://www.ensembl.org/id/ENSG00000189159) | Jupiter microtubule associated homolog 1 [Source:HGNC Symbol;Acc:HGNC:14569] | -2,09 |
| JSRP1 | [ENSG00000167476](https://www.ensembl.org/id/ENSG00000167476) | junctional sarcoplasmic reticulum protein 1 [Source:HGNC Symbol;Acc:HGNC:24963] | -9,42 |
| JUP | [ENSG00000173801](https://www.ensembl.org/id/ENSG00000173801) | junction plakoglobin [Source:HGNC Symbol;Acc:HGNC:6207] | -1,33 |
| KANSL1L-AS1 | [ENSG00000229127](https://www.ensembl.org/id/ENSG00000229127) | KANSL1L antisense RNA 1 [Source:HGNC Symbol;Acc:HGNC:41139] | 2,10 |
| KAZN | [ENSG00000189337](https://www.ensembl.org/id/ENSG00000189337) | kazrin, periplakin interacting protein [Source:HGNC Symbol;Acc:HGNC:29173] | 1,49 |
| KBTBD12 | [ENSG00000187715](https://www.ensembl.org/id/ENSG00000187715) | kelch repeat and BTB domain containing 12 [Source:HGNC Symbol;Acc:HGNC:25731] | -2,52 |
| KBTBD13 | [ENSG00000234438](https://www.ensembl.org/id/ENSG00000234438) | kelch repeat and BTB domain containing 13 [Source:HGNC Symbol;Acc:HGNC:37227] | -2,69 |
| KCNA2 | [ENSG00000177301](https://www.ensembl.org/id/ENSG00000177301) | potassium voltage-gated channel subfamily A member 2 [Source:HGNC Symbol;Acc:HGNC:6220] | 1,83 |
| KCNA5 | [ENSG00000130037](https://www.ensembl.org/id/ENSG00000130037) | potassium voltage-gated channel subfamily A member 5 [Source:HGNC Symbol;Acc:HGNC:6224] | 1,75 |
| KCNA7 | [ENSG00000104848](https://www.ensembl.org/id/ENSG00000104848) | potassium voltage-gated channel subfamily A member 7 [Source:HGNC Symbol;Acc:HGNC:6226] | -13,05 |
| KCNAB1 | [ENSG00000169282](https://www.ensembl.org/id/ENSG00000169282) | potassium voltage-gated channel subfamily A regulatory beta subunit 1 [Source:HGNC Symbol;Acc:HGNC:6228] | 1,40 |
| KCNB1 | [ENSG00000158445](https://www.ensembl.org/id/ENSG00000158445) | potassium voltage-gated channel subfamily B member 1 [Source:HGNC Symbol;Acc:HGNC:6231] | -1,74 |
| KCNC1 | [ENSG00000129159](https://www.ensembl.org/id/ENSG00000129159) | potassium voltage-gated channel subfamily C member 1 [Source:HGNC Symbol;Acc:HGNC:6233] | -3,17 |
| KCNE4 | [ENSG00000152049](https://www.ensembl.org/id/ENSG00000152049) | potassium voltage-gated channel subfamily E regulatory subunit 4 [Source:HGNC Symbol;Acc:HGNC:6244] | 1,70 |
| KCNE5 | [ENSG00000176076](https://www.ensembl.org/id/ENSG00000176076) | potassium voltage-gated channel subfamily E regulatory subunit 5 [Source:HGNC Symbol;Acc:HGNC:6241] | -4,80 |
| KCNF1 | [ENSG00000162975](https://www.ensembl.org/id/ENSG00000162975) | potassium voltage-gated channel modifier subfamily F member 1 [Source:HGNC Symbol;Acc:HGNC:6246] | -6,58 |
| KCNH2 | [ENSG00000055118](https://www.ensembl.org/id/ENSG00000055118) | potassium voltage-gated channel subfamily H member 2 [Source:HGNC Symbol;Acc:HGNC:6251] | 2,61 |
| KCNJ11 | [ENSG00000187486](https://www.ensembl.org/id/ENSG00000187486) | potassium inwardly rectifying channel subfamily J member 11 [Source:HGNC Symbol;Acc:HGNC:6257] | -4,19 |
| KCNJ12 | [ENSG00000184185](https://www.ensembl.org/id/ENSG00000184185) | potassium inwardly rectifying channel subfamily J member 12 [Source:HGNC Symbol;Acc:HGNC:6258] | -3,51 |
| KCNJ2 | [ENSG00000123700](https://www.ensembl.org/id/ENSG00000123700) | potassium inwardly rectifying channel subfamily J member 2 [Source:HGNC Symbol;Acc:HGNC:6263] | -2,43 |
| KCNJ2-AS1 | [ENSG00000267365](https://www.ensembl.org/id/ENSG00000267365) | KCNJ2 antisense RNA 1 [Source:HGNC Symbol;Acc:HGNC:43720] | -2,37 |
| KCNJ3 | [ENSG00000162989](https://www.ensembl.org/id/ENSG00000162989) | potassium inwardly rectifying channel subfamily J member 3 [Source:HGNC Symbol;Acc:HGNC:6264] | 1,39 |
| KCNJ8 | [ENSG00000121361](https://www.ensembl.org/id/ENSG00000121361) | potassium inwardly rectifying channel subfamily J member 8 [Source:HGNC Symbol;Acc:HGNC:6269] | 1,12 |
| KCNK12 | [ENSG00000184261](https://www.ensembl.org/id/ENSG00000184261) | potassium two pore domain channel subfamily K member 12 [Source:HGNC Symbol;Acc:HGNC:6274] | -2,05 |
| KCNK3 | [ENSG00000171303](https://www.ensembl.org/id/ENSG00000171303) | potassium two pore domain channel subfamily K member 3 [Source:HGNC Symbol;Acc:HGNC:6278] | 1,39 |
| KCNN2 | [ENSG00000080709](https://www.ensembl.org/id/ENSG00000080709) | potassium calcium-activated channel subfamily N member 2 [Source:HGNC Symbol;Acc:HGNC:6291] | -2,40 |
| KCNQ2 | [ENSG00000075043](https://www.ensembl.org/id/ENSG00000075043) | potassium voltage-gated channel subfamily Q member 2 [Source:HGNC Symbol;Acc:HGNC:6296] | -5,49 |
| KCNQ4 | [ENSG00000117013](https://www.ensembl.org/id/ENSG00000117013) | potassium voltage-gated channel subfamily Q member 4 [Source:HGNC Symbol;Acc:HGNC:6298] | 1,01 |
| KCNQ5 | [ENSG00000185760](https://www.ensembl.org/id/ENSG00000185760) | potassium voltage-gated channel subfamily Q member 5 [Source:HGNC Symbol;Acc:HGNC:6299] | -2,19 |
| KCNS3 | [ENSG00000170745](https://www.ensembl.org/id/ENSG00000170745) | potassium voltage-gated channel modifier subfamily S member 3 [Source:HGNC Symbol;Acc:HGNC:6302] | -2,01 |
| KCTD15 | [ENSG00000153885](https://www.ensembl.org/id/ENSG00000153885) | potassium channel tetramerization domain containing 15 [Source:HGNC Symbol;Acc:HGNC:23297] | 0,94 |
| KDM2B-DT | [ENSG00000256742](https://www.ensembl.org/id/ENSG00000256742) | KDM2B divergent transcript [Source:HGNC Symbol;Acc:HGNC:53287] | -5,20 |
| KDM4E | [ENSG00000235268](https://www.ensembl.org/id/ENSG00000235268) | lysine demethylase 4E [Source:HGNC Symbol;Acc:HGNC:37098] | -4,06 |
| KDSR-DT | [ENSG00000267390](https://www.ensembl.org/id/ENSG00000267390) | KDSR divergent transcript [Source:HGNC Symbol;Acc:HGNC:55299] | -1,58 |
| KEAP1 | [ENSG00000079999](https://www.ensembl.org/id/ENSG00000079999) | kelch like ECH associated protein 1 [Source:HGNC Symbol;Acc:HGNC:23177] | -1,17 |
| KERA | [ENSG00000139330](https://www.ensembl.org/id/ENSG00000139330) | keratocan [Source:HGNC Symbol;Acc:HGNC:6309] | -4,41 |
| KHDRBS2 | [ENSG00000112232](https://www.ensembl.org/id/ENSG00000112232) | KH RNA binding domain containing, signal transduction associated 2 [Source:HGNC Symbol;Acc:HGNC:18114] | -2,88 |
| KIAA0408 | [ENSG00000189367](https://www.ensembl.org/id/ENSG00000189367) | KIAA0408 [Source:HGNC Symbol;Acc:HGNC:21636] | -2,59 |
| KIAA1217 | [ENSG00000120549](https://www.ensembl.org/id/ENSG00000120549) | KIAA1217 [Source:HGNC Symbol;Acc:HGNC:25428] | -2,21 |
| KIAA1549 | [ENSG00000122778](https://www.ensembl.org/id/ENSG00000122778) | KIAA1549 [Source:HGNC Symbol;Acc:HGNC:22219] | -2,67 |
| KIAA1671 | [ENSG00000197077](https://www.ensembl.org/id/ENSG00000197077) | KIAA1671 [Source:HGNC Symbol;Acc:HGNC:29345] | 1,49 |
| KIAA1755 | [ENSG00000149633](https://www.ensembl.org/id/ENSG00000149633) | KIAA1755 [Source:HGNC Symbol;Acc:HGNC:29372] | -1,43 |
| KIF1A | [ENSG00000130294](https://www.ensembl.org/id/ENSG00000130294) | kinesin family member 1A [Source:HGNC Symbol;Acc:HGNC:888] | -4,85 |
| KIF1B | [ENSG00000054523](https://www.ensembl.org/id/ENSG00000054523) | kinesin family member 1B [Source:HGNC Symbol;Acc:HGNC:16636] | -2,10 |
| KIF1C | [ENSG00000129250](https://www.ensembl.org/id/ENSG00000129250) | kinesin family member 1C [Source:HGNC Symbol;Acc:HGNC:6317] | -2,00 |
| KIF23-AS1 | [ENSG00000259426](https://www.ensembl.org/id/ENSG00000259426) | KIF23 and PAQR5 antisense RNA 1 [Source:HGNC Symbol;Acc:HGNC:27075] | 1,87 |
| KIF24 | [ENSG00000186638](https://www.ensembl.org/id/ENSG00000186638) | kinesin family member 24 [Source:HGNC Symbol;Acc:HGNC:19916] | -3,57 |
| KLF11 | [ENSG00000172059](https://www.ensembl.org/id/ENSG00000172059) | KLF transcription factor 11 [Source:HGNC Symbol;Acc:HGNC:11811] | 0,88 |
| KLF14 | [ENSG00000266265](https://www.ensembl.org/id/ENSG00000266265) | KLF transcription factor 14 [Source:HGNC Symbol;Acc:HGNC:23025] | -3,67 |
| KLF15 | [ENSG00000163884](https://www.ensembl.org/id/ENSG00000163884) | KLF transcription factor 15 [Source:HGNC Symbol;Acc:HGNC:14536] | -1,06 |
| KLF5 | [ENSG00000102554](https://www.ensembl.org/id/ENSG00000102554) | KLF transcription factor 5 [Source:HGNC Symbol;Acc:HGNC:6349] | -1,29 |
| KLF8 | [ENSG00000102349](https://www.ensembl.org/id/ENSG00000102349) | KLF transcription factor 8 [Source:HGNC Symbol;Acc:HGNC:6351] | 1,08 |
| KLHDC3 | [ENSG00000124702](https://www.ensembl.org/id/ENSG00000124702) | kelch domain containing 3 [Source:HGNC Symbol;Acc:HGNC:20704] | -1,07 |
| KLHDC7B | [ENSG00000130487](https://www.ensembl.org/id/ENSG00000130487) | kelch domain containing 7B [Source:HGNC Symbol;Acc:HGNC:25145] | -4,05 |
| KLHDC8B | [ENSG00000185909](https://www.ensembl.org/id/ENSG00000185909) | kelch domain containing 8B [Source:HGNC Symbol;Acc:HGNC:28557] | -0,99 |
| KLHL30 | [ENSG00000168427](https://www.ensembl.org/id/ENSG00000168427) | kelch like family member 30 [Source:HGNC Symbol;Acc:HGNC:24770] | -1,43 |
| KLHL31 | [ENSG00000124743](https://www.ensembl.org/id/ENSG00000124743) | kelch like family member 31 [Source:HGNC Symbol;Acc:HGNC:21353] | -8,66 |
| KLHL33 | [ENSG00000185271](https://www.ensembl.org/id/ENSG00000185271) | kelch like family member 33 [Source:HGNC Symbol;Acc:HGNC:31952] | -5,45 |
| KLHL34 | [ENSG00000185915](https://www.ensembl.org/id/ENSG00000185915) | kelch like family member 34 [Source:HGNC Symbol;Acc:HGNC:26634] | -10,57 |
| KLHL38 | [ENSG00000175946](https://www.ensembl.org/id/ENSG00000175946) | kelch like family member 38 [Source:HGNC Symbol;Acc:HGNC:34435] | -3,85 |
| KLHL40 | [ENSG00000157119](https://www.ensembl.org/id/ENSG00000157119) | kelch like family member 40 [Source:HGNC Symbol;Acc:HGNC:30372] | -9,83 |
| KLHL41 | [ENSG00000239474](https://www.ensembl.org/id/ENSG00000239474) | kelch like family member 41 [Source:HGNC Symbol;Acc:HGNC:16905] | -10,60 |
| KLHL42 | [ENSG00000087448](https://www.ensembl.org/id/ENSG00000087448) | kelch like family member 42 [Source:HGNC Symbol;Acc:HGNC:29252] | 1,11 |
| KMT2E-AS1 | [ENSG00000239569](https://www.ensembl.org/id/ENSG00000239569) | KMT2E antisense RNA 1 [Source:HGNC Symbol;Acc:HGNC:40845] | -1,59 |
| KNSTRN | [ENSG00000128944](https://www.ensembl.org/id/ENSG00000128944) | kinetochore localized astrin (SPAG5) binding protein [Source:HGNC Symbol;Acc:HGNC:30767] | -1,42 |
| KPNA3 | [ENSG00000102753](https://www.ensembl.org/id/ENSG00000102753) | karyopherin subunit alpha 3 [Source:HGNC Symbol;Acc:HGNC:6396] | -1,45 |
| KPNA4 | [ENSG00000186432](https://www.ensembl.org/id/ENSG00000186432) | karyopherin subunit alpha 4 [Source:HGNC Symbol;Acc:HGNC:6397] | -1,42 |
| KREMEN1 | [ENSG00000183762](https://www.ensembl.org/id/ENSG00000183762) | kringle containing transmembrane protein 1 [Source:HGNC Symbol;Acc:HGNC:17550] | -2,50 |
| KREMEN2 | [ENSG00000131650](https://www.ensembl.org/id/ENSG00000131650) | kringle containing transmembrane protein 2 [Source:HGNC Symbol;Acc:HGNC:18797] | -5,18 |
| KRT19 | [ENSG00000171345](https://www.ensembl.org/id/ENSG00000171345) | keratin 19 [Source:HGNC Symbol;Acc:HGNC:6436] | -1,64 |
| KRT31 | [ENSG00000094796](https://www.ensembl.org/id/ENSG00000094796) | keratin 31 [Source:HGNC Symbol;Acc:HGNC:6448] | -7,24 |
| KRT32 | [ENSG00000108759](https://www.ensembl.org/id/ENSG00000108759) | keratin 32 [Source:HGNC Symbol;Acc:HGNC:6449] | -4,96 |
| KRT7 | [ENSG00000135480](https://www.ensembl.org/id/ENSG00000135480) | keratin 7 [Source:HGNC Symbol;Acc:HGNC:6445] | -2,20 |
| KRT87P | [ENSG00000290966](https://www.ensembl.org/id/ENSG00000290966) | keratin 87, pseudogene [Source:NCBI gene (formerly Entrezgene);Acc:85349] | -4,95 |
| KRTAP27-1 | [ENSG00000206107](https://www.ensembl.org/id/ENSG00000206107) | keratin associated protein 27-1 [Source:HGNC Symbol;Acc:HGNC:33864] | -6,69 |
| KY | [ENSG00000174611](https://www.ensembl.org/id/ENSG00000174611) | kyphoscoliosis peptidase [Source:HGNC Symbol;Acc:HGNC:26576] | -1,86 |
| L2HGDH | [ENSG00000087299](https://www.ensembl.org/id/ENSG00000087299) | L-2-hydroxyglutarate dehydrogenase [Source:HGNC Symbol;Acc:HGNC:20499] | -1,67 |
| LACTB | [ENSG00000103642](https://www.ensembl.org/id/ENSG00000103642) | lactamase beta [Source:HGNC Symbol;Acc:HGNC:16468] | -1,50 |
| LAD1 | [ENSG00000159166](https://www.ensembl.org/id/ENSG00000159166) | ladinin 1 [Source:HGNC Symbol;Acc:HGNC:6472] | -9,27 |
| LAMA1 | [ENSG00000101680](https://www.ensembl.org/id/ENSG00000101680) | laminin subunit alpha 1 [Source:HGNC Symbol;Acc:HGNC:6481] | 3,18 |
| LAMA2 | [ENSG00000196569](https://www.ensembl.org/id/ENSG00000196569) | laminin subunit alpha 2 [Source:HGNC Symbol;Acc:HGNC:6482] | -2,07 |
| LAMTOR5 | [ENSG00000134248](https://www.ensembl.org/id/ENSG00000134248) | late endosomal/lysosomal adaptor, MAPK and MTOR activator 5 [Source:HGNC Symbol;Acc:HGNC:17955] | -0,97 |
| LANCL1-AS1 | [ENSG00000234281](https://www.ensembl.org/id/ENSG00000234281) | LANCL1 antisense RNA 1 [Source:HGNC Symbol;Acc:HGNC:50727] | -8,90 |
| LAPTM4B | [ENSG00000104341](https://www.ensembl.org/id/ENSG00000104341) | lysosomal protein transmembrane 4 beta [Source:HGNC Symbol;Acc:HGNC:13646] | -1,86 |
| LBP | [ENSG00000129988](https://www.ensembl.org/id/ENSG00000129988) | lipopolysaccharide binding protein [Source:HGNC Symbol;Acc:HGNC:6517] | -4,58 |
| LBR | [ENSG00000143815](https://www.ensembl.org/id/ENSG00000143815) | lamin B receptor [Source:HGNC Symbol;Acc:HGNC:6518] | 0,93 |
| LBX1 | [ENSG00000138136](https://www.ensembl.org/id/ENSG00000138136) | ladybird homeobox 1 [Source:HGNC Symbol;Acc:HGNC:16960] | -8,88 |
| LBX1-AS1 | [ENSG00000227128](https://www.ensembl.org/id/ENSG00000227128) | LBX1 antisense RNA 1 [Source:HGNC Symbol;Acc:HGNC:48678] | -9,13 |
| LBX2 | [ENSG00000179528](https://www.ensembl.org/id/ENSG00000179528) | ladybird homeobox 2 [Source:HGNC Symbol;Acc:HGNC:15525] | -3,15 |
| LCN8 | [ENSG00000204001](https://www.ensembl.org/id/ENSG00000204001) | lipocalin 8 [Source:HGNC Symbol;Acc:HGNC:27038] | -7,82 |
| LDB3 | [ENSG00000122367](https://www.ensembl.org/id/ENSG00000122367) | LIM domain binding 3 [Source:HGNC Symbol;Acc:HGNC:15710] | -3,33 |
| LDHB | [ENSG00000111716](https://www.ensembl.org/id/ENSG00000111716) | lactate dehydrogenase B [Source:HGNC Symbol;Acc:HGNC:6541] | -1,36 |
| LDHC | [ENSG00000166796](https://www.ensembl.org/id/ENSG00000166796) | lactate dehydrogenase C [Source:HGNC Symbol;Acc:HGNC:6544] | -4,21 |
| LDHD | [ENSG00000166816](https://www.ensembl.org/id/ENSG00000166816) | lactate dehydrogenase D [Source:HGNC Symbol;Acc:HGNC:19708] | -3,74 |
| LDLR | [ENSG00000130164](https://www.ensembl.org/id/ENSG00000130164) | low density lipoprotein receptor [Source:HGNC Symbol;Acc:HGNC:6547] | 1,72 |
| LDLRAD2 | [ENSG00000187942](https://www.ensembl.org/id/ENSG00000187942) | low density lipoprotein receptor class A domain containing 2 [Source:HGNC Symbol;Acc:HGNC:32071] | 1,28 |
| LGALS17A | [ENSG00000291086](https://www.ensembl.org/id/ENSG00000291086) | galectin 14 pseudogene [Source:NCBI gene (formerly Entrezgene);Acc:400696] | -3,71 |
| LGALSL | [ENSG00000119862](https://www.ensembl.org/id/ENSG00000119862) | galectin like [Source:HGNC Symbol;Acc:HGNC:25012] | -1,27 |
| LGR5 | [ENSG00000139292](https://www.ensembl.org/id/ENSG00000139292) | leucine rich repeat containing G protein-coupled receptor 5 [Source:HGNC Symbol;Acc:HGNC:4504] | -3,58 |
| LHFPL5 | [ENSG00000197753](https://www.ensembl.org/id/ENSG00000197753) | LHFPL tetraspan subfamily member 5 [Source:HGNC Symbol;Acc:HGNC:21253] | 2,36 |
| LHX3 | [ENSG00000107187](https://www.ensembl.org/id/ENSG00000107187) | LIM homeobox 3 [Source:HGNC Symbol;Acc:HGNC:6595] | -5,84 |
| LIAS | [ENSG00000121897](https://www.ensembl.org/id/ENSG00000121897) | lipoic acid synthetase [Source:HGNC Symbol;Acc:HGNC:16429] | -1,39 |
| LIF | [ENSG00000128342](https://www.ensembl.org/id/ENSG00000128342) | LIF interleukin 6 family cytokine [Source:HGNC Symbol;Acc:HGNC:6596] | 1,89 |
| LIMCH1 | [ENSG00000064042](https://www.ensembl.org/id/ENSG00000064042) | LIM and calponin homology domains 1 [Source:HGNC Symbol;Acc:HGNC:29191] | -1,27 |
| LIMK1 | [ENSG00000106683](https://www.ensembl.org/id/ENSG00000106683) | LIM domain kinase 1 [Source:HGNC Symbol;Acc:HGNC:6613] | -1,21 |
| LINC00032 | [ENSG00000291187](https://www.ensembl.org/id/ENSG00000291187) | long intergenic non-protein coding RNA 32 [Source:HGNC Symbol;Acc:HGNC:16506] | -4,73 |
| LINC00189 | [ENSG00000215533](https://www.ensembl.org/id/ENSG00000215533) | long intergenic non-protein coding RNA 189 [Source:HGNC Symbol;Acc:HGNC:18461] | -4,24 |
| LINC00205 | [ENSG00000223768](https://www.ensembl.org/id/ENSG00000223768) | long intergenic non-protein coding RNA 205 [Source:HGNC Symbol;Acc:HGNC:16420] | 1,18 |
| LINC00294 | [ENSG00000280798](https://www.ensembl.org/id/ENSG00000280798) | long intergenic non-protein coding RNA 294 [Source:HGNC Symbol;Acc:HGNC:27456] | 0,97 |
| LINC00343 | [ENSG00000226620](https://www.ensembl.org/id/ENSG00000226620) | long intergenic non-protein coding RNA 343 [Source:HGNC Symbol;Acc:HGNC:42500] | -3,39 |
| LINC00365 | [ENSG00000224511](https://www.ensembl.org/id/ENSG00000224511) | long intergenic non-protein coding RNA 365 [Source:HGNC Symbol;Acc:HGNC:42687] | -4,41 |
| LINC00402 | [ENSG00000235532](https://www.ensembl.org/id/ENSG00000235532) | long intergenic non-protein coding RNA 402 [Source:HGNC Symbol;Acc:HGNC:42732] | -3,54 |
| LINC00472 | [ENSG00000233237](https://www.ensembl.org/id/ENSG00000233237) | long intergenic non-protein coding RNA 472 [Source:HGNC Symbol;Acc:HGNC:21380] | 1,29 |
| LINC00486_1 | [ENSG00000230876](https://www.ensembl.org/id/ENSG00000230876) | long intergenic non-protein coding RNA 486 [Source:HGNC Symbol;Acc:HGNC:42946] | -4,91 |
| LINC00501 | [ENSG00000203645](https://www.ensembl.org/id/ENSG00000203645) | long intergenic non-protein coding RNA 501 [Source:HGNC Symbol;Acc:HGNC:43439] | -5,44 |
| LINC00570 | [ENSG00000224177](https://www.ensembl.org/id/ENSG00000224177) | long intergenic non-protein coding RNA 570 [Source:HGNC Symbol;Acc:HGNC:43717] | 2,28 |
| LINC00581 | [ENSG00000280989](https://www.ensembl.org/id/ENSG00000280989) | long intergenic non-protein coding RNA 581 [Source:HGNC Symbol;Acc:HGNC:43840] | -5,83 |
| LINC00645 | [ENSG00000258548](https://www.ensembl.org/id/ENSG00000258548) | long intergenic non-protein coding RNA 645 [Source:HGNC Symbol;Acc:HGNC:44299] | -4,31 |
| LINC00649 | [ENSG00000237945](https://www.ensembl.org/id/ENSG00000237945) | long intergenic non-protein coding RNA 649 [Source:HGNC Symbol;Acc:HGNC:44305] | -2,05 |
| LINC00652 | [ENSG00000179935](https://www.ensembl.org/id/ENSG00000179935) | long intergenic non-protein coding RNA 652 [Source:HGNC Symbol;Acc:HGNC:25003] | -5,07 |
| LINC00671 | [ENSG00000213373](https://www.ensembl.org/id/ENSG00000213373) | long intergenic non-protein coding RNA 671 [Source:HGNC Symbol;Acc:HGNC:44339] | 2,85 |
| LINC00672 | [ENSG00000263874](https://www.ensembl.org/id/ENSG00000263874) | long intergenic non-protein coding RNA 672 [Source:HGNC Symbol;Acc:HGNC:44353] | 1,42 |
| LINC00836 | [ENSG00000280809](https://www.ensembl.org/id/ENSG00000280809) | long intergenic non-protein coding RNA 836 [Source:HGNC Symbol;Acc:HGNC:44915] | -6,72 |
| LINC00888 | [ENSG00000291106](https://www.ensembl.org/id/ENSG00000291106) | long intergenic non-protein coding RNA 888 [Source:HGNC Symbol;Acc:HGNC:48575] | -2,10 |
| LINC00900 | [ENSG00000246100](https://www.ensembl.org/id/ENSG00000246100) | long intergenic non-protein coding RNA 900 [Source:HGNC Symbol;Acc:HGNC:27444] | -3,98 |
| LINC00944 | [ENSG00000256128](https://www.ensembl.org/id/ENSG00000256128) | long intergenic non-protein coding RNA 944 [Source:HGNC Symbol;Acc:HGNC:48640] | -5,95 |
| LINC01016 | [ENSG00000249346](https://www.ensembl.org/id/ENSG00000249346) | long intergenic non-protein coding RNA 1016 [Source:HGNC Symbol;Acc:HGNC:48991] | 4,38 |
| LINC01036 | [ENSG00000230426](https://www.ensembl.org/id/ENSG00000230426) | long intergenic non-protein coding RNA 1036 [Source:HGNC Symbol;Acc:HGNC:49024] | -3,60 |
| LINC01063 | [ENSG00000232065](https://www.ensembl.org/id/ENSG00000232065) | long intergenic non-protein coding RNA 1063 [Source:HGNC Symbol;Acc:HGNC:49092] | -4,88 |
| LINC01091 | [ENSG00000249464](https://www.ensembl.org/id/ENSG00000249464) | long intergenic non-protein coding RNA 1091 [Source:HGNC Symbol;Acc:HGNC:27721] | -3,15 |
| LINC01094 | [ENSG00000251442](https://www.ensembl.org/id/ENSG00000251442) | long intergenic non-protein coding RNA 1094 [Source:HGNC Symbol;Acc:HGNC:49219] | -2,32 |
| LINC01115_2 | [ENSG00000237667](https://www.ensembl.org/id/ENSG00000237667) | long intergenic non-protein coding RNA 1115 [Source:HGNC Symbol;Acc:HGNC:49258] | -4,92 |
| LINC01121 | [ENSG00000205054](https://www.ensembl.org/id/ENSG00000205054) | long intergenic non-protein coding RNA 1121 [Source:HGNC Symbol;Acc:HGNC:49266] | -3,80 |
| LINC01134 | [ENSG00000236423](https://www.ensembl.org/id/ENSG00000236423) | long intergenic non-protein coding RNA 1134 [Source:HGNC Symbol;Acc:HGNC:49449] | -4,84 |
| LINC01151 | [ENSG00000253819](https://www.ensembl.org/id/ENSG00000253819) | long intergenic non-protein coding RNA 1151 [Source:HGNC Symbol;Acc:HGNC:49471] | -3,32 |
| LINC01152 | [ENSG00000256124](https://www.ensembl.org/id/ENSG00000256124) | long intergenic non-protein coding RNA 1152 [Source:HGNC Symbol;Acc:HGNC:16752] | -5,74 |
| LINC01229 | [ENSG00000260876](https://www.ensembl.org/id/ENSG00000260876) | long intergenic non-protein coding RNA 1229 [Source:HGNC Symbol;Acc:HGNC:49682] | 2,65 |
| LINC01271 | [ENSG00000233077](https://www.ensembl.org/id/ENSG00000233077) | long intergenic non-protein coding RNA 1271 [Source:HGNC Symbol;Acc:HGNC:50327] | -3,52 |
| LINC01273 | [ENSG00000231742](https://www.ensembl.org/id/ENSG00000231742) | long intergenic non-protein coding RNA 1273 [Source:HGNC Symbol;Acc:HGNC:50329] | -1,96 |
| LINC01304 | [ENSG00000237401](https://www.ensembl.org/id/ENSG00000237401) | long intergenic non-protein coding RNA 1304 [Source:HGNC Symbol;Acc:HGNC:50472] | -6,02 |
| LINC01322 | [ENSG00000244128](https://www.ensembl.org/id/ENSG00000244128) | long intergenic non-protein coding RNA 1322 [Source:HGNC Symbol;Acc:HGNC:50528] | -6,15 |
| LINC01355 | [ENSG00000261326](https://www.ensembl.org/id/ENSG00000261326) | long intergenic non-protein coding RNA 1355 [Source:HGNC Symbol;Acc:HGNC:50584] | 1,65 |
| LINC01405 | [ENSG00000185847](https://www.ensembl.org/id/ENSG00000185847) | long intergenic non-protein coding RNA 1405 [Source:HGNC Symbol;Acc:HGNC:50688] | -11,79 |
| LINC01489 | [ENSG00000255727](https://www.ensembl.org/id/ENSG00000255727) | long intergenic non-protein coding RNA 1489 [Source:HGNC Symbol;Acc:HGNC:51145] | -7,69 |
| LINC01563 | [ENSG00000236819](https://www.ensembl.org/id/ENSG00000236819) | long intergenic non-protein coding RNA 1563 [Source:HGNC Symbol;Acc:HGNC:51343] | -8,10 |
| LINC01572 | [ENSG00000261008](https://www.ensembl.org/id/ENSG00000261008) | long intergenic non-protein coding RNA 1572 [Source:HGNC Symbol;Acc:HGNC:51385] | -3,00 |
| LINC01579 | [ENSG00000258754](https://www.ensembl.org/id/ENSG00000258754) | long intergenic non-protein coding RNA 1579 [Source:HGNC Symbol;Acc:HGNC:27519] | -5,93 |
| LINC01588 | [ENSG00000214900](https://www.ensembl.org/id/ENSG00000214900) | long intergenic non-protein coding RNA 1588 [Source:HGNC Symbol;Acc:HGNC:27503] | -2,01 |
| LINC01592 | [ENSG00000253658](https://www.ensembl.org/id/ENSG00000253658) | long intergenic non-protein coding RNA 1592 [Source:HGNC Symbol;Acc:HGNC:51557] | 3,27 |
| LINC01634 | [ENSG00000235295](https://www.ensembl.org/id/ENSG00000235295) | long intergenic non-protein coding RNA 1634 [Source:HGNC Symbol;Acc:HGNC:52421] | -7,55 |
| LINC01635 | [ENSG00000228397](https://www.ensembl.org/id/ENSG00000228397) | long intergenic non-protein coding RNA 1635 [Source:HGNC Symbol;Acc:HGNC:52422] | -3,73 |
| LINC01637 | [ENSG00000237476](https://www.ensembl.org/id/ENSG00000237476) | long intergenic non-protein coding RNA 1637 [Source:HGNC Symbol;Acc:HGNC:52424] | -3,49 |
| LINC01687 | [ENSG00000233215](https://www.ensembl.org/id/ENSG00000233215) | long intergenic non-protein coding RNA 1687 [Source:HGNC Symbol;Acc:HGNC:52474] | -6,18 |
| LINC01762 | [ENSG00000233154](https://www.ensembl.org/id/ENSG00000233154) | long intergenic non-protein coding RNA 1762 [Source:HGNC Symbol;Acc:HGNC:52552] | -5,59 |
| LINC01816 | [ENSG00000231327](https://www.ensembl.org/id/ENSG00000231327) | long intergenic non-protein coding RNA 1816 [Source:HGNC Symbol;Acc:HGNC:52621] | -2,60 |
| LINC01854 | [ENSG00000204460](https://www.ensembl.org/id/ENSG00000204460) | long intergenic non-protein coding RNA 1854 [Source:HGNC Symbol;Acc:HGNC:52670] | -11,43 |
| LINC01856 | [ENSG00000237574](https://www.ensembl.org/id/ENSG00000237574) | long intergenic non-protein coding RNA 1856 [Source:HGNC Symbol;Acc:HGNC:52672] | -5,38 |
| LINC01894 | [ENSG00000264345](https://www.ensembl.org/id/ENSG00000264345) | long intergenic non-protein coding RNA 1894 [Source:HGNC Symbol;Acc:HGNC:52713] | -4,70 |
| LINC01954 | [ENSG00000271952](https://www.ensembl.org/id/ENSG00000271952) | long intergenic non-protein coding RNA 1954 [Source:HGNC Symbol;Acc:HGNC:52779] | -7,22 |
| LINC01963 | [ENSG00000260804](https://www.ensembl.org/id/ENSG00000260804) | long intergenic non-protein coding RNA 1963 [Source:HGNC Symbol;Acc:HGNC:25283] | -1,34 |
| LINC01973 | [ENSG00000204283](https://www.ensembl.org/id/ENSG00000204283) | long intergenic non-protein coding RNA 1973 [Source:HGNC Symbol;Acc:HGNC:52800] | -3,93 |
| LINC02004 | [ENSG00000240006](https://www.ensembl.org/id/ENSG00000240006) | long intergenic non-protein coding RNA 2004 [Source:HGNC Symbol;Acc:HGNC:52838] | 4,92 |
| LINC02008 | [ENSG00000239440](https://www.ensembl.org/id/ENSG00000239440) | long intergenic non-protein coding RNA 2008 [Source:HGNC Symbol;Acc:HGNC:52844] | -4,59 |
| LINC02145 | [ENSG00000250490](https://www.ensembl.org/id/ENSG00000250490) | long intergenic non-protein coding RNA 2145 [Source:HGNC Symbol;Acc:HGNC:53005] | 2,27 |
| LINC02160 | [ENSG00000251443](https://www.ensembl.org/id/ENSG00000251443) | long intergenic non-protein coding RNA 2160 [Source:HGNC Symbol;Acc:HGNC:53021] | -4,89 |
| LINC02166 | [ENSG00000260259](https://www.ensembl.org/id/ENSG00000260259) | long intergenic non-protein coding RNA 2166 [Source:HGNC Symbol;Acc:HGNC:53027] | -5,84 |
| LINC02254 | [ENSG00000259664](https://www.ensembl.org/id/ENSG00000259664) | long intergenic non-protein coding RNA 2254 [Source:HGNC Symbol;Acc:HGNC:53152] | -3,43 |
| LINC02279 | [ENSG00000258933](https://www.ensembl.org/id/ENSG00000258933) | long intergenic non-protein coding RNA 2279 [Source:HGNC Symbol;Acc:HGNC:53195] | -6,31 |
| LINC02284 | [ENSG00000259719](https://www.ensembl.org/id/ENSG00000259719) | long intergenic non-protein coding RNA 2284 [Source:HGNC Symbol;Acc:HGNC:53201] | -2,86 |
| LINC02288 | [ENSG00000246548](https://www.ensembl.org/id/ENSG00000246548) | long intergenic non-protein coding RNA 2288 [Source:HGNC Symbol;Acc:HGNC:27505] | -3,12 |
| LINC02308 | [ENSG00000258675](https://www.ensembl.org/id/ENSG00000258675) | long intergenic non-protein coding RNA 2308 [Source:HGNC Symbol;Acc:HGNC:53227] | -6,26 |
| LINC02363 | [ENSG00000180712](https://www.ensembl.org/id/ENSG00000180712) | long intergenic non-protein coding RNA 2363 [Source:HGNC Symbol;Acc:HGNC:53286] | -4,22 |
| LINC02384 | [ENSG00000251301](https://www.ensembl.org/id/ENSG00000251301) | long intergenic non-protein coding RNA 2384 [Source:HGNC Symbol;Acc:HGNC:53308] | 2,34 |
| LINC02388 | [ENSG00000257259](https://www.ensembl.org/id/ENSG00000257259) | long intergenic non-protein coding RNA 2388 [Source:HGNC Symbol;Acc:HGNC:53315] | -3,68 |
| LINC02432 | [ENSG00000248810](https://www.ensembl.org/id/ENSG00000248810) | long intergenic non-protein coding RNA 2432 [Source:HGNC Symbol;Acc:HGNC:53363] | -2,97 |
| LINC02457 | [ENSG00000258018](https://www.ensembl.org/id/ENSG00000258018) | long intergenic non-protein coding RNA 2457 [Source:HGNC Symbol;Acc:HGNC:53393] | -6,02 |
| LINC02478 | [ENSG00000285373](https://www.ensembl.org/id/ENSG00000285373) | long intergenic non-protein coding RNA 2478 [Source:HGNC Symbol;Acc:HGNC:53446] | -3,76 |
| LINC02481 | [ENSG00000246526](https://www.ensembl.org/id/ENSG00000246526) | long intergenic non-protein coding RNA 2481 [Source:HGNC Symbol;Acc:HGNC:27958] | -3,52 |
| LINC02502 | [ENSG00000250392](https://www.ensembl.org/id/ENSG00000250392) | long intergenic non-protein coding RNA 2502 [Source:HGNC Symbol;Acc:HGNC:53491] | -7,41 |
| LINC02515 | [ENSG00000250620](https://www.ensembl.org/id/ENSG00000250620) | long intergenic non-protein coding RNA 2515 [Source:HGNC Symbol;Acc:HGNC:53504] | 3,31 |
| LINC02517 | [ENSG00000249145](https://www.ensembl.org/id/ENSG00000249145) | long intergenic non-protein coding RNA 2517 [Source:HGNC Symbol;Acc:HGNC:53506] | -5,60 |
| LINC02541 | [ENSG00000230943](https://www.ensembl.org/id/ENSG00000230943) | long intergenic non-protein coding RNA 2541 [Source:HGNC Symbol;Acc:HGNC:53574] | -7,43 |
| LINC02553 | [ENSG00000255039](https://www.ensembl.org/id/ENSG00000255039) | long intergenic non-protein coding RNA 2553 [Source:HGNC Symbol;Acc:HGNC:53588] | -5,81 |
| LINC02574 | [ENSG00000233975](https://www.ensembl.org/id/ENSG00000233975) | long intergenic non-protein coding RNA 2574 [Source:HGNC Symbol;Acc:HGNC:53746] | -3,38 |
| LINC02593 | [ENSG00000223764](https://www.ensembl.org/id/ENSG00000223764) | long intergenic non-protein coding RNA 2593 [Source:HGNC Symbol;Acc:HGNC:53933] | 1,53 |
| LINC02688 | [ENSG00000254872](https://www.ensembl.org/id/ENSG00000254872) | long intergenic non-protein coding RNA 2688 [Source:HGNC Symbol;Acc:HGNC:54184] | 3,10 |
| LINC02721 | [ENSG00000255133](https://www.ensembl.org/id/ENSG00000255133) | long intergenic non-protein coding RNA 2721 [Source:HGNC Symbol;Acc:HGNC:54238] | -6,67 |
| LINC02783 | [ENSG00000204362](https://www.ensembl.org/id/ENSG00000204362) | long intergenic non-protein coding RNA 2783 [Source:HGNC Symbol;Acc:HGNC:54303] | -7,22 |
| LINC02817 | [ENSG00000234754](https://www.ensembl.org/id/ENSG00000234754) | long intergenic non-protein coding RNA 2817 [Source:HGNC Symbol;Acc:HGNC:32042] | 3,15 |
| LINC02871 | [ENSG00000125899](https://www.ensembl.org/id/ENSG00000125899) | long intergenic non-protein coding RNA 2871 [Source:HGNC Symbol;Acc:HGNC:16180] | -4,72 |
| LINC02899 | [ENSG00000248874](https://www.ensembl.org/id/ENSG00000248874) | long intergenic non-protein coding RNA 2899 [Source:HGNC Symbol;Acc:HGNC:26630] | 6,93 |
| LINC02909 | [ENSG00000226397](https://www.ensembl.org/id/ENSG00000226397) | long intergenic non-protein coding RNA 2909 [Source:HGNC Symbol;Acc:HGNC:27282] | -3,23 |
| LINC02916 | [ENSG00000230555](https://www.ensembl.org/id/ENSG00000230555) | long intergenic non-protein coding RNA 2916 [Source:HGNC Symbol;Acc:HGNC:55574] | -3,03 |
| LINC02926 | [ENSG00000230310](https://www.ensembl.org/id/ENSG00000230310) | long intergenic non-protein coding RNA 2926 [Source:HGNC Symbol;Acc:HGNC:55776] | -6,27 |
| LINC03007 | [ENSG00000223561](https://www.ensembl.org/id/ENSG00000223561) | long intergenic non-protein coding RNA 3007 [Source:HGNC Symbol;Acc:HGNC:56132] | -2,20 |
| LINC03016 | [ENSG00000230825](https://www.ensembl.org/id/ENSG00000230825) | long intergenic non-protein coding RNA 3016 [Source:HGNC Symbol;Acc:HGNC:56145] | -2,69 |
| LINC03020 | [ENSG00000253967](https://www.ensembl.org/id/ENSG00000253967) | long intergenic non-protein coding RNA 3020 [Source:HGNC Symbol;Acc:HGNC:56148] | -5,00 |
| LINGO1 | [ENSG00000169783](https://www.ensembl.org/id/ENSG00000169783) | leucine rich repeat and Ig domain containing 1 [Source:HGNC Symbol;Acc:HGNC:21205] | 3,16 |
| LINGO4 | [ENSG00000213171](https://www.ensembl.org/id/ENSG00000213171) | leucine rich repeat and Ig domain containing 4 [Source:HGNC Symbol;Acc:HGNC:31814] | -10,29 |
| LINP1 | [ENSG00000223784](https://www.ensembl.org/id/ENSG00000223784) | lncRNA in non-homologous end joining pathway 1 [Source:HGNC Symbol;Acc:HGNC:53170] | -10,57 |
| LIPJ | [ENSG00000204022](https://www.ensembl.org/id/ENSG00000204022) | lipase family member J [Source:HGNC Symbol;Acc:HGNC:21773] | -3,74 |
| LMO1 | [ENSG00000166407](https://www.ensembl.org/id/ENSG00000166407) | LIM domain only 1 [Source:HGNC Symbol;Acc:HGNC:6641] | -5,66 |
| LMO7 | [ENSG00000136153](https://www.ensembl.org/id/ENSG00000136153) | LIM domain 7 [Source:HGNC Symbol;Acc:HGNC:6646] | -1,71 |
| LMOD2 | [ENSG00000170807](https://www.ensembl.org/id/ENSG00000170807) | leiomodin 2 [Source:HGNC Symbol;Acc:HGNC:6648] | -10,34 |
| LMOD3 | [ENSG00000163380](https://www.ensembl.org/id/ENSG00000163380) | leiomodin 3 [Source:HGNC Symbol;Acc:HGNC:6649] | -7,28 |
| LPAR4 | [ENSG00000147145](https://www.ensembl.org/id/ENSG00000147145) | lysophosphatidic acid receptor 4 [Source:HGNC Symbol;Acc:HGNC:4478] | -2,27 |
| LPIN1 | [ENSG00000134324](https://www.ensembl.org/id/ENSG00000134324) | lipin 1 [Source:HGNC Symbol;Acc:HGNC:13345] | -1,31 |
| LPL | [ENSG00000175445](https://www.ensembl.org/id/ENSG00000175445) | lipoprotein lipase [Source:HGNC Symbol;Acc:HGNC:6677] | -2,25 |
| LRCH2 | [ENSG00000130224](https://www.ensembl.org/id/ENSG00000130224) | leucine rich repeats and calponin homology domain containing 2 [Source:HGNC Symbol;Acc:HGNC:29292] | 1,12 |
| LRP11 | [ENSG00000120256](https://www.ensembl.org/id/ENSG00000120256) | LDL receptor related protein 11 [Source:HGNC Symbol;Acc:HGNC:16936] | 1,07 |
| LRP1B | [ENSG00000168702](https://www.ensembl.org/id/ENSG00000168702) | LDL receptor related protein 1B [Source:HGNC Symbol;Acc:HGNC:6693] | -5,99 |
| LRP4 | [ENSG00000134569](https://www.ensembl.org/id/ENSG00000134569) | LDL receptor related protein 4 [Source:HGNC Symbol;Acc:HGNC:6696] | -1,73 |
| LRRC10B | [ENSG00000204950](https://www.ensembl.org/id/ENSG00000204950) | leucine rich repeat containing 10B [Source:HGNC Symbol;Acc:HGNC:37215] | 2,12 |
| LRRC14B | [ENSG00000185028](https://www.ensembl.org/id/ENSG00000185028) | leucine rich repeat containing 14B [Source:HGNC Symbol;Acc:HGNC:37268] | -13,56 |
| LRRC2 | [ENSG00000163827](https://www.ensembl.org/id/ENSG00000163827) | leucine rich repeat containing 2 [Source:HGNC Symbol;Acc:HGNC:14676] | -6,99 |
| LRRC2-AS1 | [ENSG00000268324](https://www.ensembl.org/id/ENSG00000268324) | LRRC2 antisense RNA 1 [Source:HGNC Symbol;Acc:HGNC:15571] | -5,30 |
| LRRC20 | [ENSG00000172731](https://www.ensembl.org/id/ENSG00000172731) | leucine rich repeat containing 20 [Source:HGNC Symbol;Acc:HGNC:23421] | -4,58 |
| LRRC25 | [ENSG00000175489](https://www.ensembl.org/id/ENSG00000175489) | leucine rich repeat containing 25 [Source:HGNC Symbol;Acc:HGNC:29806] | -2,82 |
| LRRC30 | [ENSG00000206422](https://www.ensembl.org/id/ENSG00000206422) | leucine rich repeat containing 30 [Source:HGNC Symbol;Acc:HGNC:30219] | -7,38 |
| LRRC37A11P | [ENSG00000290925](https://www.ensembl.org/id/ENSG00000290925) | leucine rich repeat containing 37 member A11, pseudogene [Source:HGNC Symbol;Acc:HGNC:43815] | -4,06 |
| LRRC38 | [ENSG00000162494](https://www.ensembl.org/id/ENSG00000162494) | leucine rich repeat containing 38 [Source:HGNC Symbol;Acc:HGNC:27005] | -5,68 |
| LRRC39 | [ENSG00000122477](https://www.ensembl.org/id/ENSG00000122477) | leucine rich repeat containing 39 [Source:HGNC Symbol;Acc:HGNC:28228] | -7,09 |
| LRRC4B | [ENSG00000131409](https://www.ensembl.org/id/ENSG00000131409) | leucine rich repeat containing 4B [Source:HGNC Symbol;Acc:HGNC:25042] | 1,54 |
| LRRC4C | [ENSG00000148948](https://www.ensembl.org/id/ENSG00000148948) | leucine rich repeat containing 4C [Source:HGNC Symbol;Acc:HGNC:29317] | -1,77 |
| LRRC52 | [ENSG00000162763](https://www.ensembl.org/id/ENSG00000162763) | leucine rich repeat containing 52 [Source:HGNC Symbol;Acc:HGNC:32156] | -6,14 |
| LRRC74B | [ENSG00000187905](https://www.ensembl.org/id/ENSG00000187905) | leucine rich repeat containing 74B [Source:HGNC Symbol;Acc:HGNC:34301] | -3,75 |
| LRRN1 | [ENSG00000175928](https://www.ensembl.org/id/ENSG00000175928) | leucine rich repeat neuronal 1 [Source:HGNC Symbol;Acc:HGNC:20980] | -3,45 |
| LRRTM3 | [ENSG00000198739](https://www.ensembl.org/id/ENSG00000198739) | leucine rich repeat transmembrane neuronal 3 [Source:HGNC Symbol;Acc:HGNC:19410] | -4,87 |
| LRRTM4 | [ENSG00000176204](https://www.ensembl.org/id/ENSG00000176204) | leucine rich repeat transmembrane neuronal 4 [Source:HGNC Symbol;Acc:HGNC:19411] | -3,86 |
| LSAMP | [ENSG00000185565](https://www.ensembl.org/id/ENSG00000185565) | limbic system associated membrane protein [Source:HGNC Symbol;Acc:HGNC:6705] | -2,33 |
| LSM10 | [ENSG00000181817](https://www.ensembl.org/id/ENSG00000181817) | LSM10, U7 small nuclear RNA associated [Source:HGNC Symbol;Acc:HGNC:17562] | -1,08 |
| LSM4 | [ENSG00000130520](https://www.ensembl.org/id/ENSG00000130520) | LSM4 homolog, U6 small nuclear RNA and mRNA degradation associated [Source:HGNC Symbol;Acc:HGNC:17259] | -1,03 |
| LSMEM1 | [ENSG00000181016](https://www.ensembl.org/id/ENSG00000181016) | leucine rich single-pass membrane protein 1 [Source:HGNC Symbol;Acc:HGNC:22036] | -3,72 |
| LY75-CD302 | [ENSG00000248672](https://www.ensembl.org/id/ENSG00000248672) | LY75-CD302 readthrough [Source:HGNC Symbol;Acc:HGNC:38828] | -6,30 |
| LYPD6B | [ENSG00000150556](https://www.ensembl.org/id/ENSG00000150556) | LY6/PLAUR domain containing 6B [Source:HGNC Symbol;Acc:HGNC:27018] | -5,11 |
| LYRM7 | [ENSG00000186687](https://www.ensembl.org/id/ENSG00000186687) | LYR motif containing 7 [Source:HGNC Symbol;Acc:HGNC:28072] | -1,30 |
| MACROD1 | [ENSG00000133315](https://www.ensembl.org/id/ENSG00000133315) | mono-ADP ribosylhydrolase 1 [Source:HGNC Symbol;Acc:HGNC:29598] | -2,92 |
| MAF | [ENSG00000178573](https://www.ensembl.org/id/ENSG00000178573) | MAF bZIP transcription factor [Source:HGNC Symbol;Acc:HGNC:6776] | -2,08 |
| MAFA | [ENSG00000182759](https://www.ensembl.org/id/ENSG00000182759) | MAF bZIP transcription factor A [Source:HGNC Symbol;Acc:HGNC:23145] | -2,50 |
| MAG | [ENSG00000105695](https://www.ensembl.org/id/ENSG00000105695) | myelin associated glycoprotein [Source:HGNC Symbol;Acc:HGNC:6783] | -3,89 |
| MAGEA8-AS1 | [ENSG00000230899](https://www.ensembl.org/id/ENSG00000230899) | MAGEA8 antisense RNA 1 [Source:HGNC Symbol;Acc:HGNC:45093] | -6,18 |
| MAGI2 | [ENSG00000187391](https://www.ensembl.org/id/ENSG00000187391) | membrane associated guanylate kinase, WW and PDZ domain containing 2 [Source:HGNC Symbol;Acc:HGNC:18957] | 1,21 |
| MALT1-AS1 | [ENSG00000267226](https://www.ensembl.org/id/ENSG00000267226) | MALT1 antisense RNA 1 [Source:HGNC Symbol;Acc:HGNC:55306] | -3,28 |
| MAMSTR | [ENSG00000176909](https://www.ensembl.org/id/ENSG00000176909) | MEF2 activating motif and SAP domain containing transcriptional regulator [Source:HGNC Symbol;Acc:HGNC:26689] | -3,24 |
| MAP10 | [ENSG00000212916](https://www.ensembl.org/id/ENSG00000212916) | microtubule associated protein 10 [Source:HGNC Symbol;Acc:HGNC:29265] | -2,39 |
| MAP2K1 | [ENSG00000169032](https://www.ensembl.org/id/ENSG00000169032) | mitogen-activated protein kinase kinase 1 [Source:HGNC Symbol;Acc:HGNC:6840] | -1,19 |
| MAP2K2 | [ENSG00000126934](https://www.ensembl.org/id/ENSG00000126934) | mitogen-activated protein kinase kinase 2 [Source:HGNC Symbol;Acc:HGNC:6842] | -1,11 |
| MAP2K6 | [ENSG00000108984](https://www.ensembl.org/id/ENSG00000108984) | mitogen-activated protein kinase kinase 6 [Source:HGNC Symbol;Acc:HGNC:6846] | -2,34 |
| MAP3K19 | [ENSG00000176601](https://www.ensembl.org/id/ENSG00000176601) | mitogen-activated protein kinase kinase kinase 19 [Source:HGNC Symbol;Acc:HGNC:26249] | -3,50 |
| MAP3K20 | [ENSG00000091436](https://www.ensembl.org/id/ENSG00000091436) | mitogen-activated protein kinase kinase kinase 20 [Source:HGNC Symbol;Acc:HGNC:17797] | -1,43 |
| MAP3K5-AS2 | [ENSG00000286646](https://www.ensembl.org/id/ENSG00000286646) | MAP3K5 antisense RNA 2 [Source:HGNC Symbol;Acc:HGNC:56125] | -3,06 |
| MAP3K7CL | [ENSG00000156265](https://www.ensembl.org/id/ENSG00000156265) | MAP3K7 C-terminal like [Source:HGNC Symbol;Acc:HGNC:16457] | -1,83 |
| MAP3K9-DT | [ENSG00000259153](https://www.ensembl.org/id/ENSG00000259153) | MAP3K9 divergent transcript [Source:HGNC Symbol;Acc:HGNC:53189] | -3,74 |
| MAP4 | [ENSG00000047849](https://www.ensembl.org/id/ENSG00000047849) | microtubule associated protein 4 [Source:HGNC Symbol;Acc:HGNC:6862] | -1,70 |
| MAP4K1-AS1 | [ENSG00000267291](https://www.ensembl.org/id/ENSG00000267291) | MAP4K1 antisense RNA 1 [Source:HGNC Symbol;Acc:HGNC:55302] | -5,11 |
| MAP6D1 | [ENSG00000180834](https://www.ensembl.org/id/ENSG00000180834) | MAP6 domain containing 1 [Source:HGNC Symbol;Acc:HGNC:25753] | -2,43 |
| MAPK10 | [ENSG00000109339](https://www.ensembl.org/id/ENSG00000109339) | mitogen-activated protein kinase 10 [Source:HGNC Symbol;Acc:HGNC:6872] | -1,62 |
| MAPK12 | [ENSG00000188130](https://www.ensembl.org/id/ENSG00000188130) | mitogen-activated protein kinase 12 [Source:HGNC Symbol;Acc:HGNC:6874] | -4,51 |
| MAPK4 | [ENSG00000141639](https://www.ensembl.org/id/ENSG00000141639) | mitogen-activated protein kinase 4 [Source:HGNC Symbol;Acc:HGNC:6878] | 1,63 |
| MAPKAPK3 | [ENSG00000114738](https://www.ensembl.org/id/ENSG00000114738) | MAPK activated protein kinase 3 [Source:HGNC Symbol;Acc:HGNC:6888] | -2,18 |
| MAPRE3 | [ENSG00000084764](https://www.ensembl.org/id/ENSG00000084764) | microtubule associated protein RP/EB family member 3 [Source:HGNC Symbol;Acc:HGNC:6892] | -1,57 |
| MAPT | [ENSG00000186868](https://www.ensembl.org/id/ENSG00000186868) | microtubule associated protein tau [Source:HGNC Symbol;Acc:HGNC:6893] | -3,73 |
| MAPT-AS1 | [ENSG00000264589](https://www.ensembl.org/id/ENSG00000264589) | MAPT antisense RNA 1 [Source:HGNC Symbol;Acc:HGNC:43738] | -5,29 |
| MAPT-IT1 | [ENSG00000279685](https://www.ensembl.org/id/ENSG00000279685) | MAPT intronic transcript 1 [Source:HGNC Symbol;Acc:HGNC:43741] | -3,34 |
| MARK1 | [ENSG00000116141](https://www.ensembl.org/id/ENSG00000116141) | microtubule affinity regulating kinase 1 [Source:HGNC Symbol;Acc:HGNC:6896] | 1,35 |
| MARVELD1 | [ENSG00000155254](https://www.ensembl.org/id/ENSG00000155254) | MARVEL domain containing 1 [Source:HGNC Symbol;Acc:HGNC:28674] | 1,28 |
| MAST2 | [ENSG00000086015](https://www.ensembl.org/id/ENSG00000086015) | microtubule associated serine/threonine kinase 2 [Source:HGNC Symbol;Acc:HGNC:19035] | -1,01 |
| MB | [ENSG00000198125](https://www.ensembl.org/id/ENSG00000198125) | myoglobin [Source:HGNC Symbol;Acc:HGNC:6915] | -11,76 |
| MBP | [ENSG00000197971](https://www.ensembl.org/id/ENSG00000197971) | myelin basic protein [Source:HGNC Symbol;Acc:HGNC:6925] | -2,05 |
| MBTPS1-DT | [ENSG00000260018](https://www.ensembl.org/id/ENSG00000260018) | MBTPS1 divergent transcript [Source:HGNC Symbol;Acc:HGNC:55401] | -2,89 |
| MCCC1 | [ENSG00000078070](https://www.ensembl.org/id/ENSG00000078070) | methylcrotonyl-CoA carboxylase subunit 1 [Source:HGNC Symbol;Acc:HGNC:6936] | -0,98 |
| MCCC2 | [ENSG00000131844](https://www.ensembl.org/id/ENSG00000131844) | methylcrotonyl-CoA carboxylase subunit 2 [Source:HGNC Symbol;Acc:HGNC:6937] | -1,37 |
| MCF2L2 | [ENSG00000053524](https://www.ensembl.org/id/ENSG00000053524) | MCF.2 cell line derived transforming sequence-like 2 [Source:HGNC Symbol;Acc:HGNC:30319] | -3,93 |
| MCRIP2 | [ENSG00000172366](https://www.ensembl.org/id/ENSG00000172366) | MAPK regulated corepressor interacting protein 2 [Source:HGNC Symbol;Acc:HGNC:14142] | -2,65 |
| MCU | [ENSG00000156026](https://www.ensembl.org/id/ENSG00000156026) | mitochondrial calcium uniporter [Source:HGNC Symbol;Acc:HGNC:23526] | -1,33 |
| MDH1 | [ENSG00000014641](https://www.ensembl.org/id/ENSG00000014641) | malate dehydrogenase 1 [Source:HGNC Symbol;Acc:HGNC:6970] | -2,09 |
| MDH2 | [ENSG00000146701](https://www.ensembl.org/id/ENSG00000146701) | malate dehydrogenase 2 [Source:HGNC Symbol;Acc:HGNC:6971] | -1,98 |
| ME1 | [ENSG00000065833](https://www.ensembl.org/id/ENSG00000065833) | malic enzyme 1 [Source:HGNC Symbol;Acc:HGNC:6983] | -1,01 |
| ME2 | [ENSG00000082212](https://www.ensembl.org/id/ENSG00000082212) | malic enzyme 2 [Source:HGNC Symbol;Acc:HGNC:6984] | -1,18 |
| MECR | [ENSG00000116353](https://www.ensembl.org/id/ENSG00000116353) | mitochondrial trans-2-enoyl-CoA reductase [Source:HGNC Symbol;Acc:HGNC:19691] | -1,32 |
| MED24 | [ENSG00000008838](https://www.ensembl.org/id/ENSG00000008838) | mediator complex subunit 24 [Source:HGNC Symbol;Acc:HGNC:22963] | -1,15 |
| MEF2C | [ENSG00000081189](https://www.ensembl.org/id/ENSG00000081189) | myocyte enhancer factor 2C [Source:HGNC Symbol;Acc:HGNC:6996] | -2,26 |
| MEIOC | [ENSG00000180336](https://www.ensembl.org/id/ENSG00000180336) | meiosis specific with coiled-coil domain [Source:HGNC Symbol;Acc:HGNC:26670] | -2,30 |
| MEMO1 | [ENSG00000162959](https://www.ensembl.org/id/ENSG00000162959) | mediator of cell motility 1 [Source:HGNC Symbol;Acc:HGNC:14014] | -1,07 |
| MEP1B | [ENSG00000141434](https://www.ensembl.org/id/ENSG00000141434) | meprin A subunit beta [Source:HGNC Symbol;Acc:HGNC:7020] | -4,45 |
| MESP1 | [ENSG00000166823](https://www.ensembl.org/id/ENSG00000166823) | mesoderm posterior bHLH transcription factor 1 [Source:HGNC Symbol;Acc:HGNC:29658] | -2,90 |
| Metazoa_SRP_142 | [ENSG00000276002](https://www.ensembl.org/id/ENSG00000276002) | Metazoan signal recognition particle RNA [Source:RFAM;Acc:RF00017] | -6,33 |
| Metazoa_SRP_157 | [ENSG00000273866](https://www.ensembl.org/id/ENSG00000273866) | Metazoan signal recognition particle RNA [Source:RFAM;Acc:RF00017] | -3,93 |
| METTL21C | [ENSG00000139780](https://www.ensembl.org/id/ENSG00000139780) | methyltransferase 21C, AARS1 lysine [Source:HGNC Symbol;Acc:HGNC:33717] | -12,98 |
| METTL7B | [ENSG00000170439](https://www.ensembl.org/id/ENSG00000170439) | methyltransferase like 7B [Source:HGNC Symbol;Acc:HGNC:28276] | -3,24 |
| MFHAS1 | [ENSG00000147324](https://www.ensembl.org/id/ENSG00000147324) | multifunctional ROCO family signaling regulator 1 [Source:HGNC Symbol;Acc:HGNC:16982] | 1,23 |
| MFN2 | [ENSG00000116688](https://www.ensembl.org/id/ENSG00000116688) | mitofusin 2 [Source:HGNC Symbol;Acc:HGNC:16877] | -1,89 |
| MFSD3 | [ENSG00000167700](https://www.ensembl.org/id/ENSG00000167700) | major facilitator superfamily domain containing 3 [Source:HGNC Symbol;Acc:HGNC:25157] | -2,43 |
| MGARP | [ENSG00000137463](https://www.ensembl.org/id/ENSG00000137463) | mitochondria localized glutamic acid rich protein [Source:HGNC Symbol;Acc:HGNC:29969] | -2,34 |
| MIA | [ENSG00000261857](https://www.ensembl.org/id/ENSG00000261857) | MIA SH3 domain containing [Source:HGNC Symbol;Acc:HGNC:7076] | -2,67 |
| MIA2 | [ENSG00000150527](https://www.ensembl.org/id/ENSG00000150527) | MIA SH3 domain ER export factor 2 [Source:HGNC Symbol;Acc:HGNC:18432] | -1,05 |
| MICU2 | [ENSG00000165487](https://www.ensembl.org/id/ENSG00000165487) | mitochondrial calcium uptake 2 [Source:HGNC Symbol;Acc:HGNC:31830] | -1,14 |
| MID1IP1-AS1 | [ENSG00000238123](https://www.ensembl.org/id/ENSG00000238123) | MID1IP1 antisense RNA 1 [Source:HGNC Symbol;Acc:HGNC:40932] | -4,57 |
| MIDEAS-AS1 | [ENSG00000259065](https://www.ensembl.org/id/ENSG00000259065) | MIDEAS antisense RNA 1 [Source:HGNC Symbol;Acc:HGNC:56194] | -2,05 |
| MILR1 | [ENSG00000271605](https://www.ensembl.org/id/ENSG00000271605) | mast cell immunoglobulin like receptor 1 [Source:HGNC Symbol;Acc:HGNC:27570] | -1,93 |
| MIOS | [ENSG00000164654](https://www.ensembl.org/id/ENSG00000164654) | meiosis regulator for oocyte development [Source:HGNC Symbol;Acc:HGNC:21905] | -1,17 |
| MIR1-1HG | [ENSG00000174407](https://www.ensembl.org/id/ENSG00000174407) | MIR1-1 host gene [Source:HGNC Symbol;Acc:HGNC:16159] | -8,69 |
| MIR122HG | [ENSG00000267391](https://www.ensembl.org/id/ENSG00000267391) | MIR122 host gene [Source:HGNC Symbol;Acc:HGNC:53821] | -5,47 |
| MIR133A1HG | [ENSG00000265142](https://www.ensembl.org/id/ENSG00000265142) | MIR133A1 host gene [Source:HGNC Symbol;Acc:HGNC:49594] | -5,09 |
| MIR17HG | [ENSG00000215417](https://www.ensembl.org/id/ENSG00000215417) | miR-17-92a-1 cluster host gene [Source:HGNC Symbol;Acc:HGNC:23564] | 2,10 |
| MIR193BHG | [ENSG00000262454](https://www.ensembl.org/id/ENSG00000262454) | MIR193b-365a host gene [Source:HGNC Symbol;Acc:HGNC:51945] | -1,45 |
| MIR29B2CHG | [ENSG00000203709](https://www.ensembl.org/id/ENSG00000203709) | MIR29B2 and MIR29C host gene [Source:HGNC Symbol;Acc:HGNC:32018] | 1,04 |
| MIR3147HG | [ENSG00000260653](https://www.ensembl.org/id/ENSG00000260653) | MIR3147 host gene [Source:HGNC Symbol;Acc:HGNC:56144] | -3,77 |
| MIR762HG | [ENSG00000260083](https://www.ensembl.org/id/ENSG00000260083) | MIR762 host gene [Source:HGNC Symbol;Acc:HGNC:51386] | -1,82 |
| MKNK2 | [ENSG00000099875](https://www.ensembl.org/id/ENSG00000099875) | MAPK interacting serine/threonine kinase 2 [Source:HGNC Symbol;Acc:HGNC:7111] | -1,34 |
| MKRN2OS | [ENSG00000225526](https://www.ensembl.org/id/ENSG00000225526) | MKRN2 opposite strand [Source:HGNC Symbol;Acc:HGNC:40375] | -5,60 |
| MKX | [ENSG00000150051](https://www.ensembl.org/id/ENSG00000150051) | mohawk homeobox [Source:HGNC Symbol;Acc:HGNC:23729] | 1,49 |
| MLIP | [ENSG00000146147](https://www.ensembl.org/id/ENSG00000146147) | muscular LMNA interacting protein [Source:HGNC Symbol;Acc:HGNC:21355] | -5,98 |
| MLLT11 | [ENSG00000213190](https://www.ensembl.org/id/ENSG00000213190) | MLLT11 transcription factor 7 cofactor [Source:HGNC Symbol;Acc:HGNC:16997] | -2,95 |
| MLXIPL | [ENSG00000009950](https://www.ensembl.org/id/ENSG00000009950) | MLX interacting protein like [Source:HGNC Symbol;Acc:HGNC:12744] | -2,54 |
| MMACHC | [ENSG00000132763](https://www.ensembl.org/id/ENSG00000132763) | metabolism of cobalamin associated C [Source:HGNC Symbol;Acc:HGNC:24525] | -1,60 |
| MMP3 | [ENSG00000149968](https://www.ensembl.org/id/ENSG00000149968) | matrix metallopeptidase 3 [Source:HGNC Symbol;Acc:HGNC:7173] | -4,15 |
| MNT | [ENSG00000070444](https://www.ensembl.org/id/ENSG00000070444) | MAX network transcriptional repressor [Source:HGNC Symbol;Acc:HGNC:7188] | 1,03 |
| MPC1 | [ENSG00000060762](https://www.ensembl.org/id/ENSG00000060762) | mitochondrial pyruvate carrier 1 [Source:HGNC Symbol;Acc:HGNC:21606] | -1,13 |
| MPC2 | [ENSG00000143158](https://www.ensembl.org/id/ENSG00000143158) | mitochondrial pyruvate carrier 2 [Source:HGNC Symbol;Acc:HGNC:24515] | -1,69 |
| MPO | [ENSG00000005381](https://www.ensembl.org/id/ENSG00000005381) | myeloperoxidase [Source:HGNC Symbol;Acc:HGNC:7218] | -3,46 |
| MPP7 | [ENSG00000150054](https://www.ensembl.org/id/ENSG00000150054) | MAGUK p55 scaffold protein 7 [Source:HGNC Symbol;Acc:HGNC:26542] | 0,94 |
| MPPED2 | [ENSG00000066382](https://www.ensembl.org/id/ENSG00000066382) | metallophosphoesterase domain containing 2 [Source:HGNC Symbol;Acc:HGNC:1180] | -1,20 |
| MPST | [ENSG00000128309](https://www.ensembl.org/id/ENSG00000128309) | mercaptopyruvate sulfurtransferase [Source:HGNC Symbol;Acc:HGNC:7223] | -1,05 |
| MPZ | [ENSG00000158887](https://www.ensembl.org/id/ENSG00000158887) | myelin protein zero [Source:HGNC Symbol;Acc:HGNC:7225] | -2,70 |
| MRGPRF | [ENSG00000172935](https://www.ensembl.org/id/ENSG00000172935) | MAS related GPR family member F [Source:HGNC Symbol;Acc:HGNC:24828] | 1,68 |
| MRLN | [ENSG00000227877](https://www.ensembl.org/id/ENSG00000227877) | myoregulin [Source:HGNC Symbol;Acc:HGNC:48649] | -6,40 |
| MRPL1 | [ENSG00000169288](https://www.ensembl.org/id/ENSG00000169288) | mitochondrial ribosomal protein L1 [Source:HGNC Symbol;Acc:HGNC:14275] | -0,97 |
| MRPL12 | [ENSG00000262814](https://www.ensembl.org/id/ENSG00000262814) | mitochondrial ribosomal protein L12 [Source:HGNC Symbol;Acc:HGNC:10378] | -1,78 |
| MRPL14 | [ENSG00000180992](https://www.ensembl.org/id/ENSG00000180992) | mitochondrial ribosomal protein L14 [Source:HGNC Symbol;Acc:HGNC:14279] | -2,40 |
| MRPL15 | [ENSG00000137547](https://www.ensembl.org/id/ENSG00000137547) | mitochondrial ribosomal protein L15 [Source:HGNC Symbol;Acc:HGNC:14054] | -2,13 |
| MRPL2 | [ENSG00000112651](https://www.ensembl.org/id/ENSG00000112651) | mitochondrial ribosomal protein L2 [Source:HGNC Symbol;Acc:HGNC:14056] | -0,99 |
| MRPL33 | [ENSG00000243147](https://www.ensembl.org/id/ENSG00000243147) | mitochondrial ribosomal protein L33 [Source:HGNC Symbol;Acc:HGNC:14487] | -1,15 |
| MRPL34 | [ENSG00000130312](https://www.ensembl.org/id/ENSG00000130312) | mitochondrial ribosomal protein L34 [Source:HGNC Symbol;Acc:HGNC:14488] | -0,93 |
| MRPL36 | [ENSG00000171421](https://www.ensembl.org/id/ENSG00000171421) | mitochondrial ribosomal protein L36 [Source:HGNC Symbol;Acc:HGNC:14490] | -1,13 |
| MRPL37 | [ENSG00000116221](https://www.ensembl.org/id/ENSG00000116221) | mitochondrial ribosomal protein L37 [Source:HGNC Symbol;Acc:HGNC:14034] | -1,34 |
| MRPL38 | [ENSG00000204316](https://www.ensembl.org/id/ENSG00000204316) | mitochondrial ribosomal protein L38 [Source:HGNC Symbol;Acc:HGNC:14033] | -0,96 |
| MRPL40 | [ENSG00000185608](https://www.ensembl.org/id/ENSG00000185608) | mitochondrial ribosomal protein L40 [Source:HGNC Symbol;Acc:HGNC:14491] | -1,25 |
| MRPL41 | [ENSG00000182154](https://www.ensembl.org/id/ENSG00000182154) | mitochondrial ribosomal protein L41 [Source:HGNC Symbol;Acc:HGNC:14492] | -1,76 |
| MRPL44 | [ENSG00000135900](https://www.ensembl.org/id/ENSG00000135900) | mitochondrial ribosomal protein L44 [Source:HGNC Symbol;Acc:HGNC:16650] | -1,36 |
| MRPL46 | [ENSG00000259494](https://www.ensembl.org/id/ENSG00000259494) | mitochondrial ribosomal protein L46 [Source:HGNC Symbol;Acc:HGNC:1192] | -1,25 |
| MRPL51 | [ENSG00000111639](https://www.ensembl.org/id/ENSG00000111639) | mitochondrial ribosomal protein L51 [Source:HGNC Symbol;Acc:HGNC:14044] | -1,00 |
| MRPL57 | [ENSG00000173141](https://www.ensembl.org/id/ENSG00000173141) | mitochondrial ribosomal protein L57 [Source:HGNC Symbol;Acc:HGNC:14514] | -1,28 |
| MRPS15 | [ENSG00000116898](https://www.ensembl.org/id/ENSG00000116898) | mitochondrial ribosomal protein S15 [Source:HGNC Symbol;Acc:HGNC:14504] | -1,17 |
| MRPS16 | [ENSG00000182180](https://www.ensembl.org/id/ENSG00000182180) | mitochondrial ribosomal protein S16 [Source:HGNC Symbol;Acc:HGNC:14048] | -1,06 |
| MRPS18B | [ENSG00000204568](https://www.ensembl.org/id/ENSG00000204568) | mitochondrial ribosomal protein S18B [Source:HGNC Symbol;Acc:HGNC:14516] | -1,48 |
| MRPS24 | [ENSG00000062582](https://www.ensembl.org/id/ENSG00000062582) | mitochondrial ribosomal protein S24 [Source:HGNC Symbol;Acc:HGNC:14510] | -1,26 |
| MRPS28 | [ENSG00000147586](https://www.ensembl.org/id/ENSG00000147586) | mitochondrial ribosomal protein S28 [Source:HGNC Symbol;Acc:HGNC:14513] | -1,70 |
| MRPS30 | [ENSG00000112996](https://www.ensembl.org/id/ENSG00000112996) | mitochondrial ribosomal protein S30 [Source:HGNC Symbol;Acc:HGNC:8769] | -1,16 |
| MRPS33 | [ENSG00000090263](https://www.ensembl.org/id/ENSG00000090263) | mitochondrial ribosomal protein S33 [Source:HGNC Symbol;Acc:HGNC:16634] | -1,61 |
| MRPS7 | [ENSG00000125445](https://www.ensembl.org/id/ENSG00000125445) | mitochondrial ribosomal protein S7 [Source:HGNC Symbol;Acc:HGNC:14499] | -1,57 |
| MS4A6A | [ENSG00000110077](https://www.ensembl.org/id/ENSG00000110077) | membrane spanning 4-domains A6A [Source:HGNC Symbol;Acc:HGNC:13375] | -1,74 |
| MS4A8 | [ENSG00000166959](https://www.ensembl.org/id/ENSG00000166959) | membrane spanning 4-domains A8 [Source:HGNC Symbol;Acc:HGNC:13380] | -6,21 |
| MSI2 | [ENSG00000153944](https://www.ensembl.org/id/ENSG00000153944) | musashi RNA binding protein 2 [Source:HGNC Symbol;Acc:HGNC:18585] | -1,42 |
| MSR1 | [ENSG00000038945](https://www.ensembl.org/id/ENSG00000038945) | macrophage scavenger receptor 1 [Source:HGNC Symbol;Acc:HGNC:7376] | -2,35 |
| MSS51 | [ENSG00000166343](https://www.ensembl.org/id/ENSG00000166343) | MSS51 mitochondrial translational activator [Source:HGNC Symbol;Acc:HGNC:21000] | -5,95 |
| MSTN | [ENSG00000138379](https://www.ensembl.org/id/ENSG00000138379) | myostatin [Source:HGNC Symbol;Acc:HGNC:4223] | -2,59 |
| MT-ATP6 | [ENSG00000198899](https://www.ensembl.org/id/ENSG00000198899) | mitochondrially encoded ATP synthase membrane subunit 6 [Source:HGNC Symbol;Acc:HGNC:7414] | -2,88 |
| MT-ATP8 | [ENSG00000228253](https://www.ensembl.org/id/ENSG00000228253) | mitochondrially encoded ATP synthase membrane subunit 8 [Source:HGNC Symbol;Acc:HGNC:7415] | -3,51 |
| MT-CO1 | [ENSG00000198804](https://www.ensembl.org/id/ENSG00000198804) | mitochondrially encoded cytochrome c oxidase I [Source:HGNC Symbol;Acc:HGNC:7419] | -2,99 |
| MT-CO2 | [ENSG00000198712](https://www.ensembl.org/id/ENSG00000198712) | mitochondrially encoded cytochrome c oxidase II [Source:HGNC Symbol;Acc:HGNC:7421] | -2,73 |
| MT-CO3 | [ENSG00000198938](https://www.ensembl.org/id/ENSG00000198938) | mitochondrially encoded cytochrome c oxidase III [Source:HGNC Symbol;Acc:HGNC:7422] | -2,71 |
| MT-CYB | [ENSG00000198727](https://www.ensembl.org/id/ENSG00000198727) | mitochondrially encoded cytochrome b [Source:HGNC Symbol;Acc:HGNC:7427] | -3,08 |
| MT-ND1 | [ENSG00000198888](https://www.ensembl.org/id/ENSG00000198888) | mitochondrially encoded NADH:ubiquinone oxidoreductase core subunit 1 [Source:HGNC Symbol;Acc:HGNC:7455] | -3,30 |
| MT-ND2 | [ENSG00000198763](https://www.ensembl.org/id/ENSG00000198763) | mitochondrially encoded NADH:ubiquinone oxidoreductase core subunit 2 [Source:HGNC Symbol;Acc:HGNC:7456] | -2,99 |
| MT-ND3 | [ENSG00000198840](https://www.ensembl.org/id/ENSG00000198840) | mitochondrially encoded NADH:ubiquinone oxidoreductase core subunit 3 [Source:HGNC Symbol;Acc:HGNC:7458] | -3,02 |
| MT-ND4 | [ENSG00000198886](https://www.ensembl.org/id/ENSG00000198886) | mitochondrially encoded NADH:ubiquinone oxidoreductase core subunit 4 [Source:HGNC Symbol;Acc:HGNC:7459] | -2,93 |
| MT-ND4L | [ENSG00000212907](https://www.ensembl.org/id/ENSG00000212907) | mitochondrially encoded NADH:ubiquinone oxidoreductase core subunit 4L [Source:HGNC Symbol;Acc:HGNC:7460] | -3,45 |
| MT-ND5 | [ENSG00000198786](https://www.ensembl.org/id/ENSG00000198786) | mitochondrially encoded NADH:ubiquinone oxidoreductase core subunit 5 [Source:HGNC Symbol;Acc:HGNC:7461] | -3,17 |
| MT-ND6 | [ENSG00000198695](https://www.ensembl.org/id/ENSG00000198695) | mitochondrially encoded NADH:ubiquinone oxidoreductase core subunit 6 [Source:HGNC Symbol;Acc:HGNC:7462] | -2,67 |
| MT-RNR1 | [ENSG00000211459](https://www.ensembl.org/id/ENSG00000211459) | mitochondrially encoded 12S rRNA [Source:HGNC Symbol;Acc:HGNC:7470] | -2,95 |
| MT-RNR2 | [ENSG00000210082](https://www.ensembl.org/id/ENSG00000210082) | mitochondrially encoded 16S rRNA [Source:HGNC Symbol;Acc:HGNC:7471] | -2,96 |
| MT1X | [ENSG00000187193](https://www.ensembl.org/id/ENSG00000187193) | metallothionein 1X [Source:HGNC Symbol;Acc:HGNC:7405] | -1,47 |
| MT3 | [ENSG00000087250](https://www.ensembl.org/id/ENSG00000087250) | metallothionein 3 [Source:HGNC Symbol;Acc:HGNC:7408] | -4,23 |
| MTCH2 | [ENSG00000109919](https://www.ensembl.org/id/ENSG00000109919) | mitochondrial carrier 2 [Source:HGNC Symbol;Acc:HGNC:17587] | -1,01 |
| MTIF2 | [ENSG00000085760](https://www.ensembl.org/id/ENSG00000085760) | mitochondrial translational initiation factor 2 [Source:HGNC Symbol;Acc:HGNC:7441] | -1,46 |
| MTLN | [ENSG00000175701](https://www.ensembl.org/id/ENSG00000175701) | mitoregulin [Source:HGNC Symbol;Acc:HGNC:27339] | -2,67 |
| MTMR14 | [ENSG00000163719](https://www.ensembl.org/id/ENSG00000163719) | myotubularin related protein 14 [Source:HGNC Symbol;Acc:HGNC:26190] | -0,92 |
| MTUS2 | [ENSG00000132938](https://www.ensembl.org/id/ENSG00000132938) | microtubule associated scaffold protein 2 [Source:HGNC Symbol;Acc:HGNC:20595] | -2,41 |
| MUC1 | [ENSG00000185499](https://www.ensembl.org/id/ENSG00000185499) | mucin 1, cell surface associated [Source:HGNC Symbol;Acc:HGNC:7508] | 1,65 |
| MUC13 | [ENSG00000173702](https://www.ensembl.org/id/ENSG00000173702) | mucin 13, cell surface associated [Source:HGNC Symbol;Acc:HGNC:7511] | -4,98 |
| MUC22 | [ENSG00000261272](https://www.ensembl.org/id/ENSG00000261272) | mucin 22 [Source:HGNC Symbol;Acc:HGNC:39755] | -4,57 |
| MYADML2 | [ENSG00000185105](https://www.ensembl.org/id/ENSG00000185105) | myeloid associated differentiation marker like 2 [Source:HGNC Symbol;Acc:HGNC:34548] | -13,99 |
| MYB | [ENSG00000118513](https://www.ensembl.org/id/ENSG00000118513) | MYB proto-oncogene, transcription factor [Source:HGNC Symbol;Acc:HGNC:7545] | -3,20 |
| MYBL1 | [ENSG00000185697](https://www.ensembl.org/id/ENSG00000185697) | MYB proto-oncogene like 1 [Source:HGNC Symbol;Acc:HGNC:7547] | 1,21 |
| MYBPC1 | [ENSG00000196091](https://www.ensembl.org/id/ENSG00000196091) | myosin binding protein C1 [Source:HGNC Symbol;Acc:HGNC:7549] | -11,81 |
| MYBPC2 | [ENSG00000086967](https://www.ensembl.org/id/ENSG00000086967) | myosin binding protein C2 [Source:HGNC Symbol;Acc:HGNC:7550] | -9,64 |
| MYBPH | [ENSG00000133055](https://www.ensembl.org/id/ENSG00000133055) | myosin binding protein H [Source:HGNC Symbol;Acc:HGNC:7552] | -5,04 |
| MYF5 | [ENSG00000111049](https://www.ensembl.org/id/ENSG00000111049) | myogenic factor 5 [Source:HGNC Symbol;Acc:HGNC:7565] | -6,80 |
| MYF6 | [ENSG00000111046](https://www.ensembl.org/id/ENSG00000111046) | myogenic factor 6 [Source:HGNC Symbol;Acc:HGNC:7566] | -7,95 |
| MYH1 | [ENSG00000109061](https://www.ensembl.org/id/ENSG00000109061) | myosin heavy chain 1 [Source:HGNC Symbol;Acc:HGNC:7567] | -7,60 |
| MYH14 | [ENSG00000105357](https://www.ensembl.org/id/ENSG00000105357) | myosin heavy chain 14 [Source:HGNC Symbol;Acc:HGNC:23212] | -3,33 |
| MYH15 | [ENSG00000144821](https://www.ensembl.org/id/ENSG00000144821) | myosin heavy chain 15 [Source:HGNC Symbol;Acc:HGNC:31073] | -3,99 |
| MYH2 | [ENSG00000125414](https://www.ensembl.org/id/ENSG00000125414) | myosin heavy chain 2 [Source:HGNC Symbol;Acc:HGNC:7572] | -10,22 |
| MYH3 | [ENSG00000109063](https://www.ensembl.org/id/ENSG00000109063) | myosin heavy chain 3 [Source:HGNC Symbol;Acc:HGNC:7573] | -4,19 |
| MYH4 | [ENSG00000264424](https://www.ensembl.org/id/ENSG00000264424) | myosin heavy chain 4 [Source:HGNC Symbol;Acc:HGNC:7574] | -5,58 |
| MYH6 | [ENSG00000197616](https://www.ensembl.org/id/ENSG00000197616) | myosin heavy chain 6 [Source:HGNC Symbol;Acc:HGNC:7576] | -11,69 |
| MYH7 | [ENSG00000092054](https://www.ensembl.org/id/ENSG00000092054) | myosin heavy chain 7 [Source:HGNC Symbol;Acc:HGNC:7577] | -15,29 |
| MYH7B | [ENSG00000078814](https://www.ensembl.org/id/ENSG00000078814) | myosin heavy chain 7B [Source:HGNC Symbol;Acc:HGNC:15906] | -6,65 |
| MYHAS | [ENSG00000272975](https://www.ensembl.org/id/ENSG00000272975) | myosin heavy chain gene cluster antisense RNA [Source:HGNC Symbol;Acc:HGNC:50609] | -10,07 |
| MYL1 | [ENSG00000168530](https://www.ensembl.org/id/ENSG00000168530) | myosin light chain 1 [Source:HGNC Symbol;Acc:HGNC:7582] | -9,55 |
| MYL10 | [ENSG00000106436](https://www.ensembl.org/id/ENSG00000106436) | myosin light chain 10 [Source:HGNC Symbol;Acc:HGNC:29825] | -8,73 |
| MYL11 | [ENSG00000180209](https://www.ensembl.org/id/ENSG00000180209) | myosin light chain 11 [Source:HGNC Symbol;Acc:HGNC:29824] | -8,19 |
| MYL12A | [ENSG00000101608](https://www.ensembl.org/id/ENSG00000101608) | myosin light chain 12A [Source:HGNC Symbol;Acc:HGNC:16701] | -2,72 |
| MYL2 | [ENSG00000111245](https://www.ensembl.org/id/ENSG00000111245) | myosin light chain 2 [Source:HGNC Symbol;Acc:HGNC:7583] | -13,66 |
| MYL3 | [ENSG00000160808](https://www.ensembl.org/id/ENSG00000160808) | myosin light chain 3 [Source:HGNC Symbol;Acc:HGNC:7584] | -8,96 |
| MYL4 | [ENSG00000198336](https://www.ensembl.org/id/ENSG00000198336) | myosin light chain 4 [Source:HGNC Symbol;Acc:HGNC:7585] | 2,52 |
| MYL6B | [ENSG00000196465](https://www.ensembl.org/id/ENSG00000196465) | myosin light chain 6B [Source:HGNC Symbol;Acc:HGNC:29823] | -6,86 |
| MYLK2 | [ENSG00000101306](https://www.ensembl.org/id/ENSG00000101306) | myosin light chain kinase 2 [Source:HGNC Symbol;Acc:HGNC:16243] | -8,19 |
| MYLK3 | [ENSG00000140795](https://www.ensembl.org/id/ENSG00000140795) | myosin light chain kinase 3 [Source:HGNC Symbol;Acc:HGNC:29826] | -6,86 |
| MYO18A | [ENSG00000196535](https://www.ensembl.org/id/ENSG00000196535) | myosin XVIIIA [Source:HGNC Symbol;Acc:HGNC:31104] | -1,78 |
| MYO18B | [ENSG00000133454](https://www.ensembl.org/id/ENSG00000133454) | myosin XVIIIB [Source:HGNC Symbol;Acc:HGNC:18150] | -7,29 |
| MYO1E | [ENSG00000157483](https://www.ensembl.org/id/ENSG00000157483) | myosin IE [Source:HGNC Symbol;Acc:HGNC:7599] | 1,20 |
| MYO3A | [ENSG00000095777](https://www.ensembl.org/id/ENSG00000095777) | myosin IIIA [Source:HGNC Symbol;Acc:HGNC:7601] | 3,04 |
| MYOC | [ENSG00000034971](https://www.ensembl.org/id/ENSG00000034971) | myocilin [Source:HGNC Symbol;Acc:HGNC:7610] | -1,28 |
| MYOD1 | [ENSG00000129152](https://www.ensembl.org/id/ENSG00000129152) | myogenic differentiation 1 [Source:HGNC Symbol;Acc:HGNC:7611] | -8,80 |
| MYOG | [ENSG00000122180](https://www.ensembl.org/id/ENSG00000122180) | myogenin [Source:HGNC Symbol;Acc:HGNC:7612] | -6,73 |
| MYOM1 | [ENSG00000101605](https://www.ensembl.org/id/ENSG00000101605) | myomesin 1 [Source:HGNC Symbol;Acc:HGNC:7613] | -3,90 |
| MYOM2 | [ENSG00000036448](https://www.ensembl.org/id/ENSG00000036448) | myomesin 2 [Source:HGNC Symbol;Acc:HGNC:7614] | -7,84 |
| MYOM3 | [ENSG00000142661](https://www.ensembl.org/id/ENSG00000142661) | myomesin 3 [Source:HGNC Symbol;Acc:HGNC:26679] | -9,58 |
| MYORG | [ENSG00000164976](https://www.ensembl.org/id/ENSG00000164976) | myogenesis regulating glycosidase (putative) [Source:HGNC Symbol;Acc:HGNC:19918] | -2,27 |
| MYOT | [ENSG00000120729](https://www.ensembl.org/id/ENSG00000120729) | myotilin [Source:HGNC Symbol;Acc:HGNC:12399] | -10,65 |
| MYOZ1 | [ENSG00000177791](https://www.ensembl.org/id/ENSG00000177791) | myozenin 1 [Source:HGNC Symbol;Acc:HGNC:13752] | -5,23 |
| MYOZ2 | [ENSG00000172399](https://www.ensembl.org/id/ENSG00000172399) | myozenin 2 [Source:HGNC Symbol;Acc:HGNC:1330] | -6,92 |
| MYOZ3 | [ENSG00000164591](https://www.ensembl.org/id/ENSG00000164591) | myozenin 3 [Source:HGNC Symbol;Acc:HGNC:18565] | -6,20 |
| MYPN | [ENSG00000138347](https://www.ensembl.org/id/ENSG00000138347) | myopalladin [Source:HGNC Symbol;Acc:HGNC:23246] | -10,45 |
| MYRF | [ENSG00000124920](https://www.ensembl.org/id/ENSG00000124920) | myelin regulatory factor [Source:HGNC Symbol;Acc:HGNC:1181] | 2,37 |
| MYRIP | [ENSG00000170011](https://www.ensembl.org/id/ENSG00000170011) | myosin VIIA and Rab interacting protein [Source:HGNC Symbol;Acc:HGNC:19156] | 1,29 |
| NAA50 | [ENSG00000121579](https://www.ensembl.org/id/ENSG00000121579) | N-alpha-acetyltransferase 50, NatE catalytic subunit [Source:HGNC Symbol;Acc:HGNC:29533] | -1,48 |
| NACAD | [ENSG00000136274](https://www.ensembl.org/id/ENSG00000136274) | NAC alpha domain containing [Source:HGNC Symbol;Acc:HGNC:22196] | 1,15 |
| NACC2 | [ENSG00000148411](https://www.ensembl.org/id/ENSG00000148411) | NACC family member 2 [Source:HGNC Symbol;Acc:HGNC:23846] | 1,06 |
| NAIP | [ENSG00000249437](https://www.ensembl.org/id/ENSG00000249437) | NLR family apoptosis inhibitory protein [Source:HGNC Symbol;Acc:HGNC:7634] | 1,13 |
| NALF1 | [ENSG00000204442](https://www.ensembl.org/id/ENSG00000204442) | NALCN channel auxiliary factor 1 [Source:HGNC Symbol;Acc:HGNC:33877] | 2,01 |
| NANOS2 | [ENSG00000188425](https://www.ensembl.org/id/ENSG00000188425) | nanos C2HC-type zinc finger 2 [Source:HGNC Symbol;Acc:HGNC:23292] | -7,29 |
| NAP1L3 | [ENSG00000186310](https://www.ensembl.org/id/ENSG00000186310) | nucleosome assembly protein 1 like 3 [Source:HGNC Symbol;Acc:HGNC:7639] | 1,22 |
| NAP1L5 | [ENSG00000177432](https://www.ensembl.org/id/ENSG00000177432) | nucleosome assembly protein 1 like 5 [Source:HGNC Symbol;Acc:HGNC:19968] | 1,17 |
| NARF-IT1 | [ENSG00000266236](https://www.ensembl.org/id/ENSG00000266236) | NARF intronic transcript 1 [Source:HGNC Symbol;Acc:HGNC:43651] | 3,00 |
| NARS2 | [ENSG00000137513](https://www.ensembl.org/id/ENSG00000137513) | asparaginyl-tRNA synthetase 2, mitochondrial [Source:HGNC Symbol;Acc:HGNC:26274] | -1,30 |
| NATD1 | [ENSG00000274180](https://www.ensembl.org/id/ENSG00000274180) | N-acetyltransferase domain containing 1 [Source:HGNC Symbol;Acc:HGNC:30770] | -1,50 |
| NBEAL1 | [ENSG00000144426](https://www.ensembl.org/id/ENSG00000144426) | neurobeachin like 1 [Source:HGNC Symbol;Acc:HGNC:20681] | 1,15 |
| NCALD | [ENSG00000104490](https://www.ensembl.org/id/ENSG00000104490) | neurocalcin delta [Source:HGNC Symbol;Acc:HGNC:7655] | 1,16 |
| NCAM1 | [ENSG00000149294](https://www.ensembl.org/id/ENSG00000149294) | neural cell adhesion molecule 1 [Source:HGNC Symbol;Acc:HGNC:7656] | -3,76 |
| NCBP2L | [ENSG00000170935](https://www.ensembl.org/id/ENSG00000170935) | nuclear cap binding protein subunit 2 like [Source:HGNC Symbol;Acc:HGNC:31795] | -4,80 |
| NCMAP | [ENSG00000184454](https://www.ensembl.org/id/ENSG00000184454) | non-compact myelin associated protein [Source:HGNC Symbol;Acc:HGNC:29332] | -4,70 |
| NDRG2 | [ENSG00000165795](https://www.ensembl.org/id/ENSG00000165795) | NDRG family member 2 [Source:HGNC Symbol;Acc:HGNC:14460] | -2,67 |
| NDUFA1 | [ENSG00000125356](https://www.ensembl.org/id/ENSG00000125356) | NADH:ubiquinone oxidoreductase subunit A1 [Source:HGNC Symbol;Acc:HGNC:7683] | -1,52 |
| NDUFA10 | [ENSG00000130414](https://www.ensembl.org/id/ENSG00000130414) | NADH:ubiquinone oxidoreductase subunit A10 [Source:HGNC Symbol;Acc:HGNC:7684] | -1,25 |
| NDUFA12 | [ENSG00000184752](https://www.ensembl.org/id/ENSG00000184752) | NADH:ubiquinone oxidoreductase subunit A12 [Source:HGNC Symbol;Acc:HGNC:23987] | -1,28 |
| NDUFA2 | [ENSG00000131495](https://www.ensembl.org/id/ENSG00000131495) | NADH:ubiquinone oxidoreductase subunit A2 [Source:HGNC Symbol;Acc:HGNC:7685] | -1,09 |
| NDUFA3 | [ENSG00000170906](https://www.ensembl.org/id/ENSG00000170906) | NADH:ubiquinone oxidoreductase subunit A3 [Source:HGNC Symbol;Acc:HGNC:7686] | -1,69 |
| NDUFA5 | [ENSG00000128609](https://www.ensembl.org/id/ENSG00000128609) | NADH:ubiquinone oxidoreductase subunit A5 [Source:HGNC Symbol;Acc:HGNC:7688] | -1,22 |
| NDUFA6 | [ENSG00000184983](https://www.ensembl.org/id/ENSG00000184983) | NADH:ubiquinone oxidoreductase subunit A6 [Source:HGNC Symbol;Acc:HGNC:7690] | -1,13 |
| NDUFA7 | [ENSG00000267855](https://www.ensembl.org/id/ENSG00000267855) | NADH:ubiquinone oxidoreductase subunit A7 [Source:HGNC Symbol;Acc:HGNC:7691] | -1,99 |
| NDUFA8 | [ENSG00000119421](https://www.ensembl.org/id/ENSG00000119421) | NADH:ubiquinone oxidoreductase subunit A8 [Source:HGNC Symbol;Acc:HGNC:7692] | -1,66 |
| NDUFA9 | [ENSG00000139180](https://www.ensembl.org/id/ENSG00000139180) | NADH:ubiquinone oxidoreductase subunit A9 [Source:HGNC Symbol;Acc:HGNC:7693] | -2,03 |
| NDUFAB1 | [ENSG00000004779](https://www.ensembl.org/id/ENSG00000004779) | NADH:ubiquinone oxidoreductase subunit AB1 [Source:HGNC Symbol;Acc:HGNC:7694] | -1,55 |
| NDUFAF3 | [ENSG00000178057](https://www.ensembl.org/id/ENSG00000178057) | NADH:ubiquinone oxidoreductase complex assembly factor 3 [Source:HGNC Symbol;Acc:HGNC:29918] | -1,06 |
| NDUFAF8 | [ENSG00000224877](https://www.ensembl.org/id/ENSG00000224877) | NADH:ubiquinone oxidoreductase complex assembly factor 8 [Source:HGNC Symbol;Acc:HGNC:33551] | -1,07 |
| NDUFB1 | [ENSG00000183648](https://www.ensembl.org/id/ENSG00000183648) | NADH:ubiquinone oxidoreductase subunit B1 [Source:HGNC Symbol;Acc:HGNC:7695] | -1,30 |
| NDUFB10 | [ENSG00000140990](https://www.ensembl.org/id/ENSG00000140990) | NADH:ubiquinone oxidoreductase subunit B10 [Source:HGNC Symbol;Acc:HGNC:7696] | -1,64 |
| NDUFB11 | [ENSG00000147123](https://www.ensembl.org/id/ENSG00000147123) | NADH:ubiquinone oxidoreductase subunit B11 [Source:HGNC Symbol;Acc:HGNC:20372] | -1,48 |
| NDUFB2 | [ENSG00000090266](https://www.ensembl.org/id/ENSG00000090266) | NADH:ubiquinone oxidoreductase subunit B2 [Source:HGNC Symbol;Acc:HGNC:7697] | -1,52 |
| NDUFB3 | [ENSG00000119013](https://www.ensembl.org/id/ENSG00000119013) | NADH:ubiquinone oxidoreductase subunit B3 [Source:HGNC Symbol;Acc:HGNC:7698] | -2,14 |
| NDUFB4 | [ENSG00000065518](https://www.ensembl.org/id/ENSG00000065518) | NADH:ubiquinone oxidoreductase subunit B4 [Source:HGNC Symbol;Acc:HGNC:7699] | -1,21 |
| NDUFB5 | [ENSG00000136521](https://www.ensembl.org/id/ENSG00000136521) | NADH:ubiquinone oxidoreductase subunit B5 [Source:HGNC Symbol;Acc:HGNC:7700] | -1,53 |
| NDUFB9 | [ENSG00000147684](https://www.ensembl.org/id/ENSG00000147684) | NADH:ubiquinone oxidoreductase subunit B9 [Source:HGNC Symbol;Acc:HGNC:7704] | -1,45 |
| NDUFC1 | [ENSG00000109390](https://www.ensembl.org/id/ENSG00000109390) | NADH:ubiquinone oxidoreductase subunit C1 [Source:HGNC Symbol;Acc:HGNC:7705] | -1,45 |
| NDUFC2 | [ENSG00000151366](https://www.ensembl.org/id/ENSG00000151366) | NADH:ubiquinone oxidoreductase subunit C2 [Source:HGNC Symbol;Acc:HGNC:7706] | -1,06 |
| NDUFS1 | [ENSG00000023228](https://www.ensembl.org/id/ENSG00000023228) | NADH:ubiquinone oxidoreductase core subunit S1 [Source:HGNC Symbol;Acc:HGNC:7707] | -2,41 |
| NDUFS2 | [ENSG00000158864](https://www.ensembl.org/id/ENSG00000158864) | NADH:ubiquinone oxidoreductase core subunit S2 [Source:HGNC Symbol;Acc:HGNC:7708] | -2,08 |
| NDUFS3 | [ENSG00000213619](https://www.ensembl.org/id/ENSG00000213619) | NADH:ubiquinone oxidoreductase core subunit S3 [Source:HGNC Symbol;Acc:HGNC:7710] | -1,55 |
| NDUFS4 | [ENSG00000164258](https://www.ensembl.org/id/ENSG00000164258) | NADH:ubiquinone oxidoreductase subunit S4 [Source:HGNC Symbol;Acc:HGNC:7711] | -1,28 |
| NDUFS7 | [ENSG00000115286](https://www.ensembl.org/id/ENSG00000115286) | NADH:ubiquinone oxidoreductase core subunit S7 [Source:HGNC Symbol;Acc:HGNC:7714] | -2,09 |
| NDUFS8 | [ENSG00000110717](https://www.ensembl.org/id/ENSG00000110717) | NADH:ubiquinone oxidoreductase core subunit S8 [Source:HGNC Symbol;Acc:HGNC:7715] | -1,29 |
| NDUFV1 | [ENSG00000167792](https://www.ensembl.org/id/ENSG00000167792) | NADH:ubiquinone oxidoreductase core subunit V1 [Source:HGNC Symbol;Acc:HGNC:7716] | -1,39 |
| NDUFV2 | [ENSG00000178127](https://www.ensembl.org/id/ENSG00000178127) | NADH:ubiquinone oxidoreductase core subunit V2 [Source:HGNC Symbol;Acc:HGNC:7717] | -1,74 |
| NDUFV3 | [ENSG00000160194](https://www.ensembl.org/id/ENSG00000160194) | NADH:ubiquinone oxidoreductase subunit V3 [Source:HGNC Symbol;Acc:HGNC:7719] | -1,31 |
| NEB | [ENSG00000183091](https://www.ensembl.org/id/ENSG00000183091) | nebulin [Source:HGNC Symbol;Acc:HGNC:7720] | -10,93 |
| NECAB1 | [ENSG00000123119](https://www.ensembl.org/id/ENSG00000123119) | N-terminal EF-hand calcium binding protein 1 [Source:HGNC Symbol;Acc:HGNC:20983] | 1,35 |
| NECAB3 | [ENSG00000125967](https://www.ensembl.org/id/ENSG00000125967) | N-terminal EF-hand calcium binding protein 3 [Source:HGNC Symbol;Acc:HGNC:15851] | -1,36 |
| NEDD1 | [ENSG00000139350](https://www.ensembl.org/id/ENSG00000139350) | NEDD1 gamma-tubulin ring complex targeting factor [Source:HGNC Symbol;Acc:HGNC:7723] | -1,33 |
| NEDD4L | [ENSG00000049759](https://www.ensembl.org/id/ENSG00000049759) | NEDD4 like E3 ubiquitin protein ligase [Source:HGNC Symbol;Acc:HGNC:7728] | -2,93 |
| NEFH | [ENSG00000100285](https://www.ensembl.org/id/ENSG00000100285) | neurofilament heavy chain [Source:HGNC Symbol;Acc:HGNC:7737] | -3,09 |
| NEFM | [ENSG00000104722](https://www.ensembl.org/id/ENSG00000104722) | neurofilament medium chain [Source:HGNC Symbol;Acc:HGNC:7734] | -3,60 |
| NEIL2 | [ENSG00000154328](https://www.ensembl.org/id/ENSG00000154328) | nei like DNA glycosylase 2 [Source:HGNC Symbol;Acc:HGNC:18956] | -1,14 |
| NEK10 | [ENSG00000163491](https://www.ensembl.org/id/ENSG00000163491) | NIMA related kinase 10 [Source:HGNC Symbol;Acc:HGNC:18592] | -3,01 |
| NEU3 | [ENSG00000162139](https://www.ensembl.org/id/ENSG00000162139) | neuraminidase 3 [Source:HGNC Symbol;Acc:HGNC:7760] | -1,60 |
| NEURL1 | [ENSG00000107954](https://www.ensembl.org/id/ENSG00000107954) | neuralized E3 ubiquitin protein ligase 1 [Source:HGNC Symbol;Acc:HGNC:7761] | -7,26 |
| NEURL3 | [ENSG00000163121](https://www.ensembl.org/id/ENSG00000163121) | neuralized E3 ubiquitin protein ligase 3 [Source:HGNC Symbol;Acc:HGNC:25162] | -5,16 |
| NEXMIF | [ENSG00000050030](https://www.ensembl.org/id/ENSG00000050030) | neurite extension and migration factor [Source:HGNC Symbol;Acc:HGNC:29433] | 2,97 |
| NGFR | [ENSG00000064300](https://www.ensembl.org/id/ENSG00000064300) | nerve growth factor receptor [Source:HGNC Symbol;Acc:HGNC:7809] | -1,20 |
| NHS | [ENSG00000188158](https://www.ensembl.org/id/ENSG00000188158) | NHS actin remodeling regulator [Source:HGNC Symbol;Acc:HGNC:7820] | 1,12 |
| NID1 | [ENSG00000116962](https://www.ensembl.org/id/ENSG00000116962) | nidogen 1 [Source:HGNC Symbol;Acc:HGNC:7821] | 1,01 |
| NIPSNAP2 | [ENSG00000146729](https://www.ensembl.org/id/ENSG00000146729) | nipsnap homolog 2 [Source:HGNC Symbol;Acc:HGNC:4179] | -2,45 |
| NIPSNAP3B | [ENSG00000165028](https://www.ensembl.org/id/ENSG00000165028) | nipsnap homolog 3B [Source:HGNC Symbol;Acc:HGNC:23641] | -1,04 |
| NKAIN1 | [ENSG00000084628](https://www.ensembl.org/id/ENSG00000084628) | sodium/potassium transporting ATPase interacting 1 [Source:HGNC Symbol;Acc:HGNC:25743] | -7,10 |
| NLGN1 | [ENSG00000169760](https://www.ensembl.org/id/ENSG00000169760) | neuroligin 1 [Source:HGNC Symbol;Acc:HGNC:14291] | 1,46 |
| NMRK2 | [ENSG00000077009](https://www.ensembl.org/id/ENSG00000077009) | nicotinamide riboside kinase 2 [Source:HGNC Symbol;Acc:HGNC:17871] | -3,72 |
| NNT | [ENSG00000112992](https://www.ensembl.org/id/ENSG00000112992) | nicotinamide nucleotide transhydrogenase [Source:HGNC Symbol;Acc:HGNC:7863] | -2,83 |
| NNT-AS1 | [ENSG00000248092](https://www.ensembl.org/id/ENSG00000248092) | NNT antisense RNA 1 [Source:HGNC Symbol;Acc:HGNC:49005] | -1,66 |
| NOL3 | [ENSG00000140939](https://www.ensembl.org/id/ENSG00000140939) | nucleolar protein 3 [Source:HGNC Symbol;Acc:HGNC:7869] | -1,23 |
| NOP10 | [ENSG00000182117](https://www.ensembl.org/id/ENSG00000182117) | NOP10 ribonucleoprotein [Source:HGNC Symbol;Acc:HGNC:14378] | -0,87 |
| NOS1 | [ENSG00000089250](https://www.ensembl.org/id/ENSG00000089250) | nitric oxide synthase 1 [Source:HGNC Symbol;Acc:HGNC:7872] | -6,35 |
| NOTCH2NLB | [ENSG00000286019](https://www.ensembl.org/id/ENSG00000286019) | notch 2 N-terminal like B [Source:HGNC Symbol;Acc:HGNC:53923] | 1,91 |
| NOTUM | [ENSG00000185269](https://www.ensembl.org/id/ENSG00000185269) | notum, palmitoleoyl-protein carboxylesterase [Source:HGNC Symbol;Acc:HGNC:27106] | -5,17 |
| NPAS2 | [ENSG00000170485](https://www.ensembl.org/id/ENSG00000170485) | neuronal PAS domain protein 2 [Source:HGNC Symbol;Acc:HGNC:7895] | -1,37 |
| NPHP1 | [ENSG00000144061](https://www.ensembl.org/id/ENSG00000144061) | nephrocystin 1 [Source:HGNC Symbol;Acc:HGNC:7905] | -1,54 |
| NPR3 | [ENSG00000113389](https://www.ensembl.org/id/ENSG00000113389) | natriuretic peptide receptor 3 [Source:HGNC Symbol;Acc:HGNC:7945] | 1,29 |
| NPSR1-AS1 | [ENSG00000197085](https://www.ensembl.org/id/ENSG00000197085) | NPSR1 antisense RNA 1 [Source:HGNC Symbol;Acc:HGNC:22128] | -5,75 |
| NPTX1 | [ENSG00000171246](https://www.ensembl.org/id/ENSG00000171246) | neuronal pentraxin 1 [Source:HGNC Symbol;Acc:HGNC:7952] | 2,48 |
| NPY1R | [ENSG00000164128](https://www.ensembl.org/id/ENSG00000164128) | neuropeptide Y receptor Y1 [Source:HGNC Symbol;Acc:HGNC:7956] | 1,28 |
| NPY5R | [ENSG00000164129](https://www.ensembl.org/id/ENSG00000164129) | neuropeptide Y receptor Y5 [Source:HGNC Symbol;Acc:HGNC:7958] | 1,46 |
| NR0B2 | [ENSG00000131910](https://www.ensembl.org/id/ENSG00000131910) | nuclear receptor subfamily 0 group B member 2 [Source:HGNC Symbol;Acc:HGNC:7961] | -6,21 |
| NR4A3 | [ENSG00000119508](https://www.ensembl.org/id/ENSG00000119508) | nuclear receptor subfamily 4 group A member 3 [Source:HGNC Symbol;Acc:HGNC:7982] | 2,19 |
| NRAP | [ENSG00000197893](https://www.ensembl.org/id/ENSG00000197893) | nebulin related anchoring protein [Source:HGNC Symbol;Acc:HGNC:7988] | -12,18 |
| NRDC | [ENSG00000078618](https://www.ensembl.org/id/ENSG00000078618) | nardilysin convertase [Source:HGNC Symbol;Acc:HGNC:7995] | -1,50 |
| NRG4 | [ENSG00000169752](https://www.ensembl.org/id/ENSG00000169752) | neuregulin 4 [Source:HGNC Symbol;Acc:HGNC:29862] | -4,50 |
| NRIP2 | [ENSG00000053702](https://www.ensembl.org/id/ENSG00000053702) | nuclear receptor interacting protein 2 [Source:HGNC Symbol;Acc:HGNC:23078] | 1,43 |
| NT5C1A | [ENSG00000116981](https://www.ensembl.org/id/ENSG00000116981) | 5'-nucleotidase, cytosolic IA [Source:HGNC Symbol;Acc:HGNC:17819] | -4,05 |
| NT5C3A | [ENSG00000122643](https://www.ensembl.org/id/ENSG00000122643) | 5'-nucleotidase, cytosolic IIIA [Source:HGNC Symbol;Acc:HGNC:17820] | -1,32 |
| NT5M | [ENSG00000205309](https://www.ensembl.org/id/ENSG00000205309) | 5',3'-nucleotidase, mitochondrial [Source:HGNC Symbol;Acc:HGNC:15769] | -1,75 |
| NTF4 | [ENSG00000225950](https://www.ensembl.org/id/ENSG00000225950) | neurotrophin 4 [Source:HGNC Symbol;Acc:HGNC:8024] | -8,42 |
| NTRK2 | [ENSG00000148053](https://www.ensembl.org/id/ENSG00000148053) | neurotrophic receptor tyrosine kinase 2 [Source:HGNC Symbol;Acc:HGNC:8032] | 1,26 |
| NTS | [ENSG00000133636](https://www.ensembl.org/id/ENSG00000133636) | neurotensin [Source:HGNC Symbol;Acc:HGNC:8038] | 2,99 |
| NUDT14 | [ENSG00000183828](https://www.ensembl.org/id/ENSG00000183828) | nudix hydrolase 14 [Source:HGNC Symbol;Acc:HGNC:20141] | -1,46 |
| NUDT19 | [ENSG00000213965](https://www.ensembl.org/id/ENSG00000213965) | nudix hydrolase 19 [Source:HGNC Symbol;Acc:HGNC:32036] | -1,75 |
| NUDT22 | [ENSG00000149761](https://www.ensembl.org/id/ENSG00000149761) | nudix hydrolase 22 [Source:HGNC Symbol;Acc:HGNC:28189] | -0,91 |
| NUDT3 | [ENSG00000272325](https://www.ensembl.org/id/ENSG00000272325) | nudix hydrolase 3 [Source:HGNC Symbol;Acc:HGNC:8050] | -1,12 |
| NUDT8 | [ENSG00000167799](https://www.ensembl.org/id/ENSG00000167799) | nudix hydrolase 8 [Source:HGNC Symbol;Acc:HGNC:8055] | -2,68 |
| NUP210 | [ENSG00000132182](https://www.ensembl.org/id/ENSG00000132182) | nucleoporin 210 [Source:HGNC Symbol;Acc:HGNC:30052] | -1,59 |
| NXN | [ENSG00000167693](https://www.ensembl.org/id/ENSG00000167693) | nucleoredoxin [Source:HGNC Symbol;Acc:HGNC:18008] | -1,42 |
| OBI1-AS1 | [ENSG00000234377](https://www.ensembl.org/id/ENSG00000234377) | OBI1 antisense RNA 1 [Source:HGNC Symbol;Acc:HGNC:42700] | -5,84 |
| OBSCN | [ENSG00000154358](https://www.ensembl.org/id/ENSG00000154358) | obscurin, cytoskeletal calmodulin and titin-interacting RhoGEF [Source:HGNC Symbol;Acc:HGNC:15719] | -7,38 |
| OBSCN-AS1 | [ENSG00000162913](https://www.ensembl.org/id/ENSG00000162913) | OBSCN antisense RNA 1 [Source:HGNC Symbol;Acc:HGNC:32047] | 2,93 |
| ODF3L2 | [ENSG00000181781](https://www.ensembl.org/id/ENSG00000181781) | outer dense fiber of sperm tails 3 like 2 [Source:HGNC Symbol;Acc:HGNC:26841] | -4,82 |
| OGDH | [ENSG00000105953](https://www.ensembl.org/id/ENSG00000105953) | oxoglutarate dehydrogenase [Source:HGNC Symbol;Acc:HGNC:8124] | -1,65 |
| OIP5 | [ENSG00000104147](https://www.ensembl.org/id/ENSG00000104147) | Opa interacting protein 5 [Source:HGNC Symbol;Acc:HGNC:20300] | -2,68 |
| OLIG1 | [ENSG00000184221](https://www.ensembl.org/id/ENSG00000184221) | oligodendrocyte transcription factor 1 [Source:HGNC Symbol;Acc:HGNC:16983] | -4,41 |
| OPCML | [ENSG00000183715](https://www.ensembl.org/id/ENSG00000183715) | opioid binding protein/cell adhesion molecule like [Source:HGNC Symbol;Acc:HGNC:8143] | 2,41 |
| OPLAH | [ENSG00000178814](https://www.ensembl.org/id/ENSG00000178814) | 5-oxoprolinase, ATP-hydrolysing [Source:HGNC Symbol;Acc:HGNC:8149] | -3,56 |
| OPTN | [ENSG00000123240](https://www.ensembl.org/id/ENSG00000123240) | optineurin [Source:HGNC Symbol;Acc:HGNC:17142] | -1,45 |
| OR1J1 | [ENSG00000136834](https://www.ensembl.org/id/ENSG00000136834) | olfactory receptor family 1 subfamily J member 1 [Source:HGNC Symbol;Acc:HGNC:8208] | -5,07 |
| OR2AT4 | [ENSG00000171561](https://www.ensembl.org/id/ENSG00000171561) | olfactory receptor family 2 subfamily AT member 4 [Source:HGNC Symbol;Acc:HGNC:19620] | -6,02 |
| OR2B2 | [ENSG00000168131](https://www.ensembl.org/id/ENSG00000168131) | olfactory receptor family 2 subfamily B member 2 [Source:HGNC Symbol;Acc:HGNC:13966] | -5,84 |
| OR51A7 | [ENSG00000176895](https://www.ensembl.org/id/ENSG00000176895) | olfactory receptor family 51 subfamily A member 7 [Source:HGNC Symbol;Acc:HGNC:15188] | -6,46 |
| OR51E2 | [ENSG00000167332](https://www.ensembl.org/id/ENSG00000167332) | olfactory receptor family 51 subfamily E member 2 [Source:HGNC Symbol;Acc:HGNC:15195] | 1,56 |
| OR51V1 | [ENSG00000176742](https://www.ensembl.org/id/ENSG00000176742) | olfactory receptor family 51 subfamily V member 1 [Source:HGNC Symbol;Acc:HGNC:19597] | -6,69 |
| OR7E47P | [ENSG00000290965](https://www.ensembl.org/id/ENSG00000290965) | olfactory receptor family 7 subfamily E member 47 pseudogene [Source:NCBI gene (formerly Entrezgene);Acc:26628] | -5,84 |
| ORAI1 | [ENSG00000276045](https://www.ensembl.org/id/ENSG00000276045) | ORAI calcium release-activated calcium modulator 1 [Source:HGNC Symbol;Acc:HGNC:25896] | -2,66 |
| OSBPL11 | [ENSG00000144909](https://www.ensembl.org/id/ENSG00000144909) | oxysterol binding protein like 11 [Source:HGNC Symbol;Acc:HGNC:16397] | -1,16 |
| OSGIN2 | [ENSG00000164823](https://www.ensembl.org/id/ENSG00000164823) | oxidative stress induced growth inhibitor family member 2 [Source:HGNC Symbol;Acc:HGNC:1355] | -1,40 |
| OSMR | [ENSG00000145623](https://www.ensembl.org/id/ENSG00000145623) | oncostatin M receptor [Source:HGNC Symbol;Acc:HGNC:8507] | 1,49 |
| OSTN | [ENSG00000188729](https://www.ensembl.org/id/ENSG00000188729) | osteocrin [Source:HGNC Symbol;Acc:HGNC:29961] | -4,41 |
| OTOF | [ENSG00000115155](https://www.ensembl.org/id/ENSG00000115155) | otoferlin [Source:HGNC Symbol;Acc:HGNC:8515] | -3,54 |
| OTUD1 | [ENSG00000165312](https://www.ensembl.org/id/ENSG00000165312) | OTU deubiquitinase 1 [Source:HGNC Symbol;Acc:HGNC:27346] | -2,22 |
| OTX2-AS1 | [ENSG00000248550](https://www.ensembl.org/id/ENSG00000248550) | OTX2 antisense RNA 1 (head to head) [Source:HGNC Symbol;Acc:HGNC:43906] | -4,66 |
| OXA1L | [ENSG00000155463](https://www.ensembl.org/id/ENSG00000155463) | OXA1L mitochondrial inner membrane protein [Source:HGNC Symbol;Acc:HGNC:8526] | -1,08 |
| OXCT1 | [ENSG00000083720](https://www.ensembl.org/id/ENSG00000083720) | 3-oxoacid CoA-transferase 1 [Source:HGNC Symbol;Acc:HGNC:8527] | -0,92 |
| P2RX1 | [ENSG00000108405](https://www.ensembl.org/id/ENSG00000108405) | purinergic receptor P2X 1 [Source:HGNC Symbol;Acc:HGNC:8533] | 3,12 |
| P2RX3 | [ENSG00000109991](https://www.ensembl.org/id/ENSG00000109991) | purinergic receptor P2X 3 [Source:HGNC Symbol;Acc:HGNC:8534] | -6,50 |
| P2RX5 | [ENSG00000083454](https://www.ensembl.org/id/ENSG00000083454) | purinergic receptor P2X 5 [Source:HGNC Symbol;Acc:HGNC:8536] | -4,29 |
| P2RX6 | [ENSG00000099957](https://www.ensembl.org/id/ENSG00000099957) | purinergic receptor P2X 6 [Source:HGNC Symbol;Acc:HGNC:8538] | -4,79 |
| P2RY2 | [ENSG00000175591](https://www.ensembl.org/id/ENSG00000175591) | purinergic receptor P2Y2 [Source:HGNC Symbol;Acc:HGNC:8541] | -2,77 |
| P3H2 | [ENSG00000090530](https://www.ensembl.org/id/ENSG00000090530) | prolyl 3-hydroxylase 2 [Source:HGNC Symbol;Acc:HGNC:19317] | 1,22 |
| P4HA1 | [ENSG00000122884](https://www.ensembl.org/id/ENSG00000122884) | prolyl 4-hydroxylase subunit alpha 1 [Source:HGNC Symbol;Acc:HGNC:8546] | -1,09 |
| PABPC4 | [ENSG00000090621](https://www.ensembl.org/id/ENSG00000090621) | poly(A) binding protein cytoplasmic 4 [Source:HGNC Symbol;Acc:HGNC:8557] | -1,65 |
| PACERR | [ENSG00000273129](https://www.ensembl.org/id/ENSG00000273129) | PTGS2 antisense NFKB1 complex-mediated expression regulator RNA [Source:HGNC Symbol;Acc:HGNC:50552] | 3,74 |
| PACSIN3 | [ENSG00000165912](https://www.ensembl.org/id/ENSG00000165912) | protein kinase C and casein kinase substrate in neurons 3 [Source:HGNC Symbol;Acc:HGNC:8572] | -2,36 |
| PADI2 | [ENSG00000117115](https://www.ensembl.org/id/ENSG00000117115) | peptidyl arginine deiminase 2 [Source:HGNC Symbol;Acc:HGNC:18341] | -3,12 |
| PAGE4 | [ENSG00000101951](https://www.ensembl.org/id/ENSG00000101951) | PAGE family member 4 [Source:HGNC Symbol;Acc:HGNC:4108] | -5,50 |
| PAIP2B | [ENSG00000124374](https://www.ensembl.org/id/ENSG00000124374) | poly(A) binding protein interacting protein 2B [Source:HGNC Symbol;Acc:HGNC:29200] | -4,63 |
| PAK6 | [ENSG00000137843](https://www.ensembl.org/id/ENSG00000137843) | p21 (RAC1) activated kinase 6 [Source:HGNC Symbol;Acc:HGNC:16061] | -2,66 |
| PANCR | [ENSG00000250103](https://www.ensembl.org/id/ENSG00000250103) | PITX2 adjacent non-coding RNA [Source:HGNC Symbol;Acc:HGNC:52282] | -5,10 |
| PAPOLA-DT | [ENSG00000260806](https://www.ensembl.org/id/ENSG00000260806) | PAPOLA divergent transcript [Source:HGNC Symbol;Acc:HGNC:55380] | -3,08 |
| PAQR4 | [ENSG00000162073](https://www.ensembl.org/id/ENSG00000162073) | progestin and adipoQ receptor family member 4 [Source:HGNC Symbol;Acc:HGNC:26386] | -2,73 |
| PAQR5 | [ENSG00000137819](https://www.ensembl.org/id/ENSG00000137819) | progestin and adipoQ receptor family member 5 [Source:HGNC Symbol;Acc:HGNC:29645] | 2,12 |
| PAQR9 | [ENSG00000188582](https://www.ensembl.org/id/ENSG00000188582) | progestin and adipoQ receptor family member 9 [Source:HGNC Symbol;Acc:HGNC:30131] | -4,69 |
| PAQR9-AS1 | [ENSG00000241570](https://www.ensembl.org/id/ENSG00000241570) | PAQR9 antisense RNA 1 [Source:HGNC Symbol;Acc:HGNC:50861] | -5,65 |
| PARVB | [ENSG00000188677](https://www.ensembl.org/id/ENSG00000188677) | parvin beta [Source:HGNC Symbol;Acc:HGNC:14653] | -3,42 |
| PATJ | [ENSG00000132849](https://www.ensembl.org/id/ENSG00000132849) | PATJ crumbs cell polarity complex component [Source:HGNC Symbol;Acc:HGNC:28881] | -1,14 |
| PAX1 | [ENSG00000125813](https://www.ensembl.org/id/ENSG00000125813) | paired box 1 [Source:HGNC Symbol;Acc:HGNC:8615] | -3,78 |
| PAX7 | [ENSG00000009709](https://www.ensembl.org/id/ENSG00000009709) | paired box 7 [Source:HGNC Symbol;Acc:HGNC:8621] | -6,95 |
| PAX9 | [ENSG00000198807](https://www.ensembl.org/id/ENSG00000198807) | paired box 9 [Source:HGNC Symbol;Acc:HGNC:8623] | 2,55 |
| PC | [ENSG00000173599](https://www.ensembl.org/id/ENSG00000173599) | pyruvate carboxylase [Source:HGNC Symbol;Acc:HGNC:8636] | -1,73 |
| PCAT14 | [ENSG00000280623](https://www.ensembl.org/id/ENSG00000280623) | prostate cancer associated transcript 14 [Source:HGNC Symbol;Acc:HGNC:48977] | -4,98 |
| PCAT7 | [ENSG00000231806](https://www.ensembl.org/id/ENSG00000231806) | prostate cancer associated transcript 7 [Source:HGNC Symbol;Acc:HGNC:48824] | -4,85 |
| PCBD2 | [ENSG00000132570](https://www.ensembl.org/id/ENSG00000132570) | pterin-4 alpha-carbinolamine dehydratase 2 [Source:HGNC Symbol;Acc:HGNC:24474] | -1,79 |
| PCBP4 | [ENSG00000090097](https://www.ensembl.org/id/ENSG00000090097) | poly(rC) binding protein 4 [Source:HGNC Symbol;Acc:HGNC:8652] | -1,40 |
| PCCB | [ENSG00000114054](https://www.ensembl.org/id/ENSG00000114054) | propionyl-CoA carboxylase subunit beta [Source:HGNC Symbol;Acc:HGNC:8654] | -1,24 |
| PCDHB1 | [ENSG00000171815](https://www.ensembl.org/id/ENSG00000171815) | protocadherin beta 1 [Source:HGNC Symbol;Acc:HGNC:8680] | -4,72 |
| PCMT1 | [ENSG00000120265](https://www.ensembl.org/id/ENSG00000120265) | protein-L-isoaspartate (D-aspartate) O-methyltransferase [Source:HGNC Symbol;Acc:HGNC:8728] | -1,28 |
| PCMTD1-DT | [ENSG00000228801](https://www.ensembl.org/id/ENSG00000228801) | PCMTD1 divergent transcript [Source:HGNC Symbol;Acc:HGNC:55791] | -2,64 |
| PCNT | [ENSG00000160299](https://www.ensembl.org/id/ENSG00000160299) | pericentrin [Source:HGNC Symbol;Acc:HGNC:16068] | -1,41 |
| PDCL3 | [ENSG00000115539](https://www.ensembl.org/id/ENSG00000115539) | phosducin like 3 [Source:HGNC Symbol;Acc:HGNC:28860] | -1,04 |
| PDE11A | [ENSG00000128655](https://www.ensembl.org/id/ENSG00000128655) | phosphodiesterase 11A [Source:HGNC Symbol;Acc:HGNC:8773] | -3,00 |
| PDE1C | [ENSG00000154678](https://www.ensembl.org/id/ENSG00000154678) | phosphodiesterase 1C [Source:HGNC Symbol;Acc:HGNC:8776] | 1,82 |
| PDE4A | [ENSG00000065989](https://www.ensembl.org/id/ENSG00000065989) | phosphodiesterase 4A [Source:HGNC Symbol;Acc:HGNC:8780] | -1,43 |
| PDE4B | [ENSG00000184588](https://www.ensembl.org/id/ENSG00000184588) | phosphodiesterase 4B [Source:HGNC Symbol;Acc:HGNC:8781] | 1,33 |
| PDE4C | [ENSG00000105650](https://www.ensembl.org/id/ENSG00000105650) | phosphodiesterase 4C [Source:HGNC Symbol;Acc:HGNC:8782] | 2,38 |
| PDE4DIP | [ENSG00000178104](https://www.ensembl.org/id/ENSG00000178104) | phosphodiesterase 4D interacting protein [Source:HGNC Symbol;Acc:HGNC:15580] | -3,87 |
| PDE6G | [ENSG00000185527](https://www.ensembl.org/id/ENSG00000185527) | phosphodiesterase 6G [Source:HGNC Symbol;Acc:HGNC:8789] | -3,36 |
| PDE7A | [ENSG00000205268](https://www.ensembl.org/id/ENSG00000205268) | phosphodiesterase 7A [Source:HGNC Symbol;Acc:HGNC:8791] | -2,05 |
| PDGFC | [ENSG00000145431](https://www.ensembl.org/id/ENSG00000145431) | platelet derived growth factor C [Source:HGNC Symbol;Acc:HGNC:8801] | 1,07 |
| PDGFD | [ENSG00000170962](https://www.ensembl.org/id/ENSG00000170962) | platelet derived growth factor D [Source:HGNC Symbol;Acc:HGNC:30620] | -1,58 |
| PDHA1 | [ENSG00000131828](https://www.ensembl.org/id/ENSG00000131828) | pyruvate dehydrogenase E1 subunit alpha 1 [Source:HGNC Symbol;Acc:HGNC:8806] | -1,66 |
| PDHB | [ENSG00000168291](https://www.ensembl.org/id/ENSG00000168291) | pyruvate dehydrogenase E1 subunit beta [Source:HGNC Symbol;Acc:HGNC:8808] | -1,50 |
| PDHX | [ENSG00000110435](https://www.ensembl.org/id/ENSG00000110435) | pyruvate dehydrogenase complex component X [Source:HGNC Symbol;Acc:HGNC:21350] | -2,29 |
| PDLIM3 | [ENSG00000154553](https://www.ensembl.org/id/ENSG00000154553) | PDZ and LIM domain 3 [Source:HGNC Symbol;Acc:HGNC:20767] | -1,67 |
| PDLIM4 | [ENSG00000131435](https://www.ensembl.org/id/ENSG00000131435) | PDZ and LIM domain 4 [Source:HGNC Symbol;Acc:HGNC:16501] | 0,97 |
| PDPN | [ENSG00000162493](https://www.ensembl.org/id/ENSG00000162493) | podoplanin [Source:HGNC Symbol;Acc:HGNC:29602] | -2,09 |
| PDZD4 | [ENSG00000067840](https://www.ensembl.org/id/ENSG00000067840) | PDZ domain containing 4 [Source:HGNC Symbol;Acc:HGNC:21167] | 1,64 |
| PDZRN4 | [ENSG00000165966](https://www.ensembl.org/id/ENSG00000165966) | PDZ domain containing ring finger 4 [Source:HGNC Symbol;Acc:HGNC:30552] | 1,20 |
| PEBP4 | [ENSG00000134020](https://www.ensembl.org/id/ENSG00000134020) | phosphatidylethanolamine binding protein 4 [Source:HGNC Symbol;Acc:HGNC:28319] | -3,49 |
| PENK | [ENSG00000181195](https://www.ensembl.org/id/ENSG00000181195) | proenkephalin [Source:HGNC Symbol;Acc:HGNC:8831] | -2,51 |
| PERM1 | [ENSG00000187642](https://www.ensembl.org/id/ENSG00000187642) | PPARGC1 and ESRR induced regulator, muscle 1 [Source:HGNC Symbol;Acc:HGNC:28208] | -5,65 |
| PEX5 | [ENSG00000139197](https://www.ensembl.org/id/ENSG00000139197) | peroxisomal biogenesis factor 5 [Source:HGNC Symbol;Acc:HGNC:9719] | -1,20 |
| PEX5L | [ENSG00000114757](https://www.ensembl.org/id/ENSG00000114757) | peroxisomal biogenesis factor 5 like [Source:HGNC Symbol;Acc:HGNC:30024] | -2,81 |
| PF4V1 | [ENSG00000109272](https://www.ensembl.org/id/ENSG00000109272) | platelet factor 4 variant 1 [Source:HGNC Symbol;Acc:HGNC:8862] | -3,18 |
| PFKFB1 | [ENSG00000158571](https://www.ensembl.org/id/ENSG00000158571) | 6-phosphofructo-2-kinase/fructose-2,6-biphosphatase 1 [Source:HGNC Symbol;Acc:HGNC:8872] | -5,00 |
| PFKFB2 | [ENSG00000123836](https://www.ensembl.org/id/ENSG00000123836) | 6-phosphofructo-2-kinase/fructose-2,6-biphosphatase 2 [Source:HGNC Symbol;Acc:HGNC:8873] | -1,14 |
| PFKM | [ENSG00000152556](https://www.ensembl.org/id/ENSG00000152556) | phosphofructokinase, muscle [Source:HGNC Symbol;Acc:HGNC:8877] | -2,45 |
| PFKP | [ENSG00000067057](https://www.ensembl.org/id/ENSG00000067057) | phosphofructokinase, platelet [Source:HGNC Symbol;Acc:HGNC:8878] | 1,34 |
| PGA4 | [ENSG00000229183](https://www.ensembl.org/id/ENSG00000229183) | pepsinogen A4 [Source:HGNC Symbol;Acc:HGNC:8886] | -3,91 |
| PGAM2 | [ENSG00000164708](https://www.ensembl.org/id/ENSG00000164708) | phosphoglycerate mutase 2 [Source:HGNC Symbol;Acc:HGNC:8889] | -4,30 |
| PGBD5 | [ENSG00000177614](https://www.ensembl.org/id/ENSG00000177614) | piggyBac transposable element derived 5 [Source:HGNC Symbol;Acc:HGNC:19405] | -5,01 |
| PGF | [ENSG00000119630](https://www.ensembl.org/id/ENSG00000119630) | placental growth factor [Source:HGNC Symbol;Acc:HGNC:8893] | 1,63 |
| PGLYRP2 | [ENSG00000161031](https://www.ensembl.org/id/ENSG00000161031) | peptidoglycan recognition protein 2 [Source:HGNC Symbol;Acc:HGNC:30013] | -5,38 |
| PGM1 | [ENSG00000079739](https://www.ensembl.org/id/ENSG00000079739) | phosphoglucomutase 1 [Source:HGNC Symbol;Acc:HGNC:8905] | -2,46 |
| PGM2 | [ENSG00000169299](https://www.ensembl.org/id/ENSG00000169299) | phosphoglucomutase 2 [Source:HGNC Symbol;Acc:HGNC:8906] | 1,02 |
| PGM5-AS1 | [ENSG00000224958](https://www.ensembl.org/id/ENSG00000224958) | PGM5 antisense RNA 1 [Source:HGNC Symbol;Acc:HGNC:44181] | 1,36 |
| PGP | [ENSG00000184207](https://www.ensembl.org/id/ENSG00000184207) | phosphoglycolate phosphatase [Source:HGNC Symbol;Acc:HGNC:8909] | -1,68 |
| PGPEP1 | [ENSG00000130517](https://www.ensembl.org/id/ENSG00000130517) | pyroglutamyl-peptidase I [Source:HGNC Symbol;Acc:HGNC:13568] | -1,77 |
| PGPEP1L | [ENSG00000183571](https://www.ensembl.org/id/ENSG00000183571) | pyroglutamyl-peptidase I like [Source:HGNC Symbol;Acc:HGNC:27080] | -2,24 |
| PGR | [ENSG00000082175](https://www.ensembl.org/id/ENSG00000082175) | progesterone receptor [Source:HGNC Symbol;Acc:HGNC:8910] | 1,40 |
| PHKA1 | [ENSG00000067177](https://www.ensembl.org/id/ENSG00000067177) | phosphorylase kinase regulatory subunit alpha 1 [Source:HGNC Symbol;Acc:HGNC:8925] | -2,45 |
| PHKG1 | [ENSG00000164776](https://www.ensembl.org/id/ENSG00000164776) | phosphorylase kinase catalytic subunit gamma 1 [Source:HGNC Symbol;Acc:HGNC:8930] | -2,66 |
| PHPT1 | [ENSG00000054148](https://www.ensembl.org/id/ENSG00000054148) | phosphohistidine phosphatase 1 [Source:HGNC Symbol;Acc:HGNC:30033] | -1,24 |
| PHTF2 | [ENSG00000006576](https://www.ensembl.org/id/ENSG00000006576) | putative homeodomain transcription factor 2 [Source:HGNC Symbol;Acc:HGNC:13411] | -1,66 |
| PHYH | [ENSG00000107537](https://www.ensembl.org/id/ENSG00000107537) | phytanoyl-CoA 2-hydroxylase [Source:HGNC Symbol;Acc:HGNC:8940] | -2,27 |
| PHYHIP | [ENSG00000168490](https://www.ensembl.org/id/ENSG00000168490) | phytanoyl-CoA 2-hydroxylase interacting protein [Source:HGNC Symbol;Acc:HGNC:16865] | 1,43 |
| PIANP | [ENSG00000139200](https://www.ensembl.org/id/ENSG00000139200) | PILR alpha associated neural protein [Source:HGNC Symbol;Acc:HGNC:25338] | -2,52 |
| PIEZO2 | [ENSG00000154864](https://www.ensembl.org/id/ENSG00000154864) | piezo type mechanosensitive ion channel component 2 [Source:HGNC Symbol;Acc:HGNC:26270] | -2,51 |
| PIGB | [ENSG00000069943](https://www.ensembl.org/id/ENSG00000069943) | phosphatidylinositol glycan anchor biosynthesis class B [Source:HGNC Symbol;Acc:HGNC:8959] | 1,35 |
| PINK1 | [ENSG00000158828](https://www.ensembl.org/id/ENSG00000158828) | PTEN induced kinase 1 [Source:HGNC Symbol;Acc:HGNC:14581] | -2,56 |
| PIP | [ENSG00000159763](https://www.ensembl.org/id/ENSG00000159763) | prolactin induced protein [Source:HGNC Symbol;Acc:HGNC:8993] | -5,38 |
| PIP5K1B | [ENSG00000107242](https://www.ensembl.org/id/ENSG00000107242) | phosphatidylinositol-4-phosphate 5-kinase type 1 beta [Source:HGNC Symbol;Acc:HGNC:8995] | 1,25 |
| PITPNA-AS1 | [ENSG00000236618](https://www.ensembl.org/id/ENSG00000236618) | PITPNA antisense RNA 1 [Source:HGNC Symbol;Acc:HGNC:44116] | -1,98 |
| PITX1 | [ENSG00000069011](https://www.ensembl.org/id/ENSG00000069011) | paired like homeodomain 1 [Source:HGNC Symbol;Acc:HGNC:9004] | -2,04 |
| PITX2 | [ENSG00000164093](https://www.ensembl.org/id/ENSG00000164093) | paired like homeodomain 2 [Source:HGNC Symbol;Acc:HGNC:9005] | -8,38 |
| PITX3 | [ENSG00000107859](https://www.ensembl.org/id/ENSG00000107859) | paired like homeodomain 3 [Source:HGNC Symbol;Acc:HGNC:9006] | -6,27 |
| PKD1L2 | [ENSG00000166473](https://www.ensembl.org/id/ENSG00000166473) | polycystin 1 like 2 (gene/pseudogene) [Source:HGNC Symbol;Acc:HGNC:21715] | 1,71 |
| PKD2L2-DT | [ENSG00000250159](https://www.ensembl.org/id/ENSG00000250159) | PKD2L2 divergent transcript [Source:HGNC Symbol;Acc:HGNC:55557] | -5,95 |
| PKHD1 | [ENSG00000170927](https://www.ensembl.org/id/ENSG00000170927) | PKHD1 ciliary IPT domain containing fibrocystin/polyductin [Source:HGNC Symbol;Acc:HGNC:9016] | -4,13 |
| PKIA | [ENSG00000171033](https://www.ensembl.org/id/ENSG00000171033) | cAMP-dependent protein kinase inhibitor alpha [Source:HGNC Symbol;Acc:HGNC:9017] | -7,40 |
| PKP2 | [ENSG00000057294](https://www.ensembl.org/id/ENSG00000057294) | plakophilin 2 [Source:HGNC Symbol;Acc:HGNC:9024] | -5,07 |
| PLA2G15 | [ENSG00000103066](https://www.ensembl.org/id/ENSG00000103066) | phospholipase A2 group XV [Source:HGNC Symbol;Acc:HGNC:17163] | -1,18 |
| PLA2G4C | [ENSG00000105499](https://www.ensembl.org/id/ENSG00000105499) | phospholipase A2 group IVC [Source:HGNC Symbol;Acc:HGNC:9037] | -1,44 |
| PLA2G4E | [ENSG00000188089](https://www.ensembl.org/id/ENSG00000188089) | phospholipase A2 group IVE [Source:HGNC Symbol;Acc:HGNC:24791] | -5,62 |
| PLA2G4F | [ENSG00000168907](https://www.ensembl.org/id/ENSG00000168907) | phospholipase A2 group IVF [Source:HGNC Symbol;Acc:HGNC:27396] | -7,27 |
| PLA2G7 | [ENSG00000146070](https://www.ensembl.org/id/ENSG00000146070) | phospholipase A2 group VII [Source:HGNC Symbol;Acc:HGNC:9040] | -2,43 |
| PLAAT1 | [ENSG00000127252](https://www.ensembl.org/id/ENSG00000127252) | phospholipase A and acyltransferase 1 [Source:HGNC Symbol;Acc:HGNC:14922] | -6,08 |
| PLAU | [ENSG00000122861](https://www.ensembl.org/id/ENSG00000122861) | plasminogen activator, urokinase [Source:HGNC Symbol;Acc:HGNC:9052] | 1,21 |
| PLCB4 | [ENSG00000101333](https://www.ensembl.org/id/ENSG00000101333) | phospholipase C beta 4 [Source:HGNC Symbol;Acc:HGNC:9059] | 0,96 |
| PLCD3 | [ENSG00000161714](https://www.ensembl.org/id/ENSG00000161714) | phospholipase C delta 3 [Source:HGNC Symbol;Acc:HGNC:9061] | -1,20 |
| PLCD4 | [ENSG00000115556](https://www.ensembl.org/id/ENSG00000115556) | phospholipase C delta 4 [Source:HGNC Symbol;Acc:HGNC:9062] | -4,33 |
| PLCE1 | [ENSG00000138193](https://www.ensembl.org/id/ENSG00000138193) | phospholipase C epsilon 1 [Source:HGNC Symbol;Acc:HGNC:17175] | 1,48 |
| PLEKHB1 | [ENSG00000021300](https://www.ensembl.org/id/ENSG00000021300) | pleckstrin homology domain containing B1 [Source:HGNC Symbol;Acc:HGNC:19079] | -1,27 |
| PLEKHF1 | [ENSG00000166289](https://www.ensembl.org/id/ENSG00000166289) | pleckstrin homology and FYVE domain containing 1 [Source:HGNC Symbol;Acc:HGNC:20764] | -1,44 |
| PLEKHG4B | [ENSG00000153404](https://www.ensembl.org/id/ENSG00000153404) | pleckstrin homology and RhoGEF domain containing G4B [Source:HGNC Symbol;Acc:HGNC:29399] | -4,10 |
| PLEKHH2 | [ENSG00000152527](https://www.ensembl.org/id/ENSG00000152527) | pleckstrin homology, MyTH4 and FERM domain containing H2 [Source:HGNC Symbol;Acc:HGNC:30506] | 1,29 |
| PLEKHJ1 | [ENSG00000104886](https://www.ensembl.org/id/ENSG00000104886) | pleckstrin homology domain containing J1 [Source:HGNC Symbol;Acc:HGNC:18211] | -1,03 |
| PLEKHM2 | [ENSG00000116786](https://www.ensembl.org/id/ENSG00000116786) | pleckstrin homology and RUN domain containing M2 [Source:HGNC Symbol;Acc:HGNC:29131] | -1,11 |
| PLEKHO1 | [ENSG00000023902](https://www.ensembl.org/id/ENSG00000023902) | pleckstrin homology domain containing O1 [Source:HGNC Symbol;Acc:HGNC:24310] | 1,53 |
| PLIN2 | [ENSG00000147872](https://www.ensembl.org/id/ENSG00000147872) | perilipin 2 [Source:HGNC Symbol;Acc:HGNC:248] | -1,42 |
| PLIN5 | [ENSG00000214456](https://www.ensembl.org/id/ENSG00000214456) | perilipin 5 [Source:HGNC Symbol;Acc:HGNC:33196] | -5,09 |
| PLP1 | [ENSG00000123560](https://www.ensembl.org/id/ENSG00000123560) | proteolipid protein 1 [Source:HGNC Symbol;Acc:HGNC:9086] | -1,65 |
| PLPP7 | [ENSG00000160539](https://www.ensembl.org/id/ENSG00000160539) | phospholipid phosphatase 7 (inactive) [Source:HGNC Symbol;Acc:HGNC:28174] | -2,98 |
| PLXNB3 | [ENSG00000198753](https://www.ensembl.org/id/ENSG00000198753) | plexin B3 [Source:HGNC Symbol;Acc:HGNC:9105] | 1,78 |
| PNCK | [ENSG00000130822](https://www.ensembl.org/id/ENSG00000130822) | pregnancy up-regulated nonubiquitous CaM kinase [Source:HGNC Symbol;Acc:HGNC:13415] | 2,02 |
| PNMA1 | [ENSG00000176903](https://www.ensembl.org/id/ENSG00000176903) | PNMA family member 1 [Source:HGNC Symbol;Acc:HGNC:9158] | 1,12 |
| PNMT | [ENSG00000141744](https://www.ensembl.org/id/ENSG00000141744) | phenylethanolamine N-methyltransferase [Source:HGNC Symbol;Acc:HGNC:9160] | -1,85 |
| PNPLA3 | [ENSG00000100344](https://www.ensembl.org/id/ENSG00000100344) | patatin like phospholipase domain containing 3 [Source:HGNC Symbol;Acc:HGNC:18590] | -5,60 |
| PNPLA4 | [ENSG00000006757](https://www.ensembl.org/id/ENSG00000006757) | patatin like phospholipase domain containing 4 [Source:HGNC Symbol;Acc:HGNC:24887] | -1,82 |
| PNPO | [ENSG00000108439](https://www.ensembl.org/id/ENSG00000108439) | pyridoxamine 5'-phosphate oxidase [Source:HGNC Symbol;Acc:HGNC:30260] | -1,21 |
| POLB | [ENSG00000070501](https://www.ensembl.org/id/ENSG00000070501) | DNA polymerase beta [Source:HGNC Symbol;Acc:HGNC:9174] | -1,46 |
| POLDIP2 | [ENSG00000004142](https://www.ensembl.org/id/ENSG00000004142) | DNA polymerase delta interacting protein 2 [Source:HGNC Symbol;Acc:HGNC:23781] | -1,66 |
| POLR2J | [ENSG00000005075](https://www.ensembl.org/id/ENSG00000005075) | RNA polymerase II subunit J [Source:HGNC Symbol;Acc:HGNC:9197] | -1,07 |
| POMC | [ENSG00000115138](https://www.ensembl.org/id/ENSG00000115138) | proopiomelanocortin [Source:HGNC Symbol;Acc:HGNC:9201] | -2,94 |
| POPDC2 | [ENSG00000121577](https://www.ensembl.org/id/ENSG00000121577) | popeye domain containing 2 [Source:HGNC Symbol;Acc:HGNC:17648] | -2,75 |
| POPDC3 | [ENSG00000132429](https://www.ensembl.org/id/ENSG00000132429) | popeye domain containing 3 [Source:HGNC Symbol;Acc:HGNC:17649] | -6,72 |
| POTED | [ENSG00000166351](https://www.ensembl.org/id/ENSG00000166351) | POTE ankyrin domain family member D [Source:HGNC Symbol;Acc:HGNC:23822] | -5,36 |
| POTEM | [ENSG00000222036](https://www.ensembl.org/id/ENSG00000222036) | POTE ankyrin domain family member M [Source:HGNC Symbol;Acc:HGNC:37096] | -5,65 |
| PPA1 | [ENSG00000180817](https://www.ensembl.org/id/ENSG00000180817) | inorganic pyrophosphatase 1 [Source:HGNC Symbol;Acc:HGNC:9226] | -0,90 |
| PPARGC1A | [ENSG00000109819](https://www.ensembl.org/id/ENSG00000109819) | PPARG coactivator 1 alpha [Source:HGNC Symbol;Acc:HGNC:9237] | -1,82 |
| PPARGC1B | [ENSG00000155846](https://www.ensembl.org/id/ENSG00000155846) | PPARG coactivator 1 beta [Source:HGNC Symbol;Acc:HGNC:30022] | -1,88 |
| PPDPFL | [ENSG00000168333](https://www.ensembl.org/id/ENSG00000168333) | pancreatic progenitor cell differentiation and proliferation factor like [Source:HGNC Symbol;Acc:HGNC:31745] | -11,07 |
| PPFIA4 | [ENSG00000143847](https://www.ensembl.org/id/ENSG00000143847) | PTPRF interacting protein alpha 4 [Source:HGNC Symbol;Acc:HGNC:9248] | -3,67 |
| PPID | [ENSG00000171497](https://www.ensembl.org/id/ENSG00000171497) | peptidylprolyl isomerase D [Source:HGNC Symbol;Acc:HGNC:9257] | -1,04 |
| PPIP5K1 | [ENSG00000168781](https://www.ensembl.org/id/ENSG00000168781) | diphosphoinositol pentakisphosphate kinase 1 [Source:HGNC Symbol;Acc:HGNC:29023] | -0,91 |
| PPM1H | [ENSG00000111110](https://www.ensembl.org/id/ENSG00000111110) | protein phosphatase, Mg2+/Mn2+ dependent 1H [Source:HGNC Symbol;Acc:HGNC:18583] | 1,67 |
| PPM1J | [ENSG00000155367](https://www.ensembl.org/id/ENSG00000155367) | protein phosphatase, Mg2+/Mn2+ dependent 1J [Source:HGNC Symbol;Acc:HGNC:20785] | -2,48 |
| PPP1R14B-AS1 | [ENSG00000256940](https://www.ensembl.org/id/ENSG00000256940) | PPP1R14B antisense RNA 1 [Source:HGNC Symbol;Acc:HGNC:54233] | -3,45 |
| PPP1R14C | [ENSG00000198729](https://www.ensembl.org/id/ENSG00000198729) | protein phosphatase 1 regulatory inhibitor subunit 14C [Source:HGNC Symbol;Acc:HGNC:14952] | -5,21 |
| PPP1R16A | [ENSG00000160972](https://www.ensembl.org/id/ENSG00000160972) | protein phosphatase 1 regulatory subunit 16A [Source:HGNC Symbol;Acc:HGNC:14941] | -1,58 |
| PPP1R1A | [ENSG00000135447](https://www.ensembl.org/id/ENSG00000135447) | protein phosphatase 1 regulatory inhibitor subunit 1A [Source:HGNC Symbol;Acc:HGNC:9286] | -3,30 |
| PPP1R1C | [ENSG00000150722](https://www.ensembl.org/id/ENSG00000150722) | protein phosphatase 1 regulatory inhibitor subunit 1C [Source:HGNC Symbol;Acc:HGNC:14940] | -3,92 |
| PPP1R27 | [ENSG00000182676](https://www.ensembl.org/id/ENSG00000182676) | protein phosphatase 1 regulatory subunit 27 [Source:HGNC Symbol;Acc:HGNC:16813] | -8,96 |
| PPP1R3A | [ENSG00000154415](https://www.ensembl.org/id/ENSG00000154415) | protein phosphatase 1 regulatory subunit 3A [Source:HGNC Symbol;Acc:HGNC:9291] | -10,78 |
| PPP1R42 | [ENSG00000178125](https://www.ensembl.org/id/ENSG00000178125) | protein phosphatase 1 regulatory subunit 42 [Source:HGNC Symbol;Acc:HGNC:33732] | -5,79 |
| PPP2R3A | [ENSG00000073711](https://www.ensembl.org/id/ENSG00000073711) | protein phosphatase 2 regulatory subunit B''alpha [Source:HGNC Symbol;Acc:HGNC:9307] | -3,17 |
| PPP2R3B | [ENSG00000167393](https://www.ensembl.org/id/ENSG00000167393) | protein phosphatase 2 regulatory subunit B''beta [Source:HGNC Symbol;Acc:HGNC:13417] | -3,44 |
| PPP3CB | [ENSG00000107758](https://www.ensembl.org/id/ENSG00000107758) | protein phosphatase 3 catalytic subunit beta [Source:HGNC Symbol;Acc:HGNC:9315] | -1,24 |
| PPTC7 | [ENSG00000196850](https://www.ensembl.org/id/ENSG00000196850) | protein phosphatase targeting COQ7 [Source:HGNC Symbol;Acc:HGNC:30695] | -1,06 |
| PRADC1 | [ENSG00000135617](https://www.ensembl.org/id/ENSG00000135617) | protease associated domain containing 1 [Source:HGNC Symbol;Acc:HGNC:16047] | -1,42 |
| PRAG1 | [ENSG00000275342](https://www.ensembl.org/id/ENSG00000275342) | PEAK1 related, kinase-activating pseudokinase 1 [Source:HGNC Symbol;Acc:HGNC:25438] | -2,08 |
| PRAMEF26 | [ENSG00000280267](https://www.ensembl.org/id/ENSG00000280267) | PRAME family member 26 [Source:HGNC Symbol;Acc:HGNC:49178] | -4,68 |
| PRAMEF4 | [ENSG00000243073](https://www.ensembl.org/id/ENSG00000243073) | PRAME family member 4 [Source:HGNC Symbol;Acc:HGNC:31971] | -4,68 |
| PRDX2 | [ENSG00000167815](https://www.ensembl.org/id/ENSG00000167815) | peroxiredoxin 2 [Source:HGNC Symbol;Acc:HGNC:9353] | -1,26 |
| PRDX3 | [ENSG00000165672](https://www.ensembl.org/id/ENSG00000165672) | peroxiredoxin 3 [Source:HGNC Symbol;Acc:HGNC:9354] | -1,44 |
| PRDX5 | [ENSG00000126432](https://www.ensembl.org/id/ENSG00000126432) | peroxiredoxin 5 [Source:HGNC Symbol;Acc:HGNC:9355] | -1,14 |
| PREB | [ENSG00000138073](https://www.ensembl.org/id/ENSG00000138073) | prolactin regulatory element binding [Source:HGNC Symbol;Acc:HGNC:9356] | -1,41 |
| PRECSIT | [ENSG00000255874](https://www.ensembl.org/id/ENSG00000255874) | p53 regulated carcinoma associated Stat3 activating long intergenic non-protein coding transcript [Source:HGNC Symbol;Acc:HGNC:27492] | -2,43 |
| PREP | [ENSG00000085377](https://www.ensembl.org/id/ENSG00000085377) | prolyl endopeptidase [Source:HGNC Symbol;Acc:HGNC:9358] | -1,09 |
| PRICKLE4 | [ENSG00000278224](https://www.ensembl.org/id/ENSG00000278224) | prickle planar cell polarity protein 4 [Source:HGNC Symbol;Acc:HGNC:16805] | 2,73 |
| PRIMA1 | [ENSG00000175785](https://www.ensembl.org/id/ENSG00000175785) | proline rich membrane anchor 1 [Source:HGNC Symbol;Acc:HGNC:18319] | 1,17 |
| PRKAA2 | [ENSG00000162409](https://www.ensembl.org/id/ENSG00000162409) | protein kinase AMP-activated catalytic subunit alpha 2 [Source:HGNC Symbol;Acc:HGNC:9377] | -2,66 |
| PRKAB2 | [ENSG00000131791](https://www.ensembl.org/id/ENSG00000131791) | protein kinase AMP-activated non-catalytic subunit beta 2 [Source:HGNC Symbol;Acc:HGNC:9379] | -2,02 |
| PRKACB | [ENSG00000142875](https://www.ensembl.org/id/ENSG00000142875) | protein kinase cAMP-activated catalytic subunit beta [Source:HGNC Symbol;Acc:HGNC:9381] | 1,06 |
| PRKAG2 | [ENSG00000106617](https://www.ensembl.org/id/ENSG00000106617) | protein kinase AMP-activated non-catalytic subunit gamma 2 [Source:HGNC Symbol;Acc:HGNC:9386] | 1,03 |
| PRKAG3 | [ENSG00000115592](https://www.ensembl.org/id/ENSG00000115592) | protein kinase AMP-activated non-catalytic subunit gamma 3 [Source:HGNC Symbol;Acc:HGNC:9387] | -5,73 |
| PRKAR1B-AS2 | [ENSG00000229380](https://www.ensembl.org/id/ENSG00000229380) | PRKAR1B antisense RNA 2 [Source:HGNC Symbol;Acc:HGNC:40469] | -3,59 |
| PRKCQ | [ENSG00000065675](https://www.ensembl.org/id/ENSG00000065675) | protein kinase C theta [Source:HGNC Symbol;Acc:HGNC:9410] | -6,50 |
| PRKCQ-AS1 | [ENSG00000237943](https://www.ensembl.org/id/ENSG00000237943) | PRKCQ antisense RNA 1 [Source:HGNC Symbol;Acc:HGNC:44689] | -5,46 |
| PRMT9 | [ENSG00000164169](https://www.ensembl.org/id/ENSG00000164169) | protein arginine methyltransferase 9 [Source:HGNC Symbol;Acc:HGNC:25099] | 1,29 |
| PROB1 | [ENSG00000228672](https://www.ensembl.org/id/ENSG00000228672) | proline rich basic protein 1 [Source:HGNC Symbol;Acc:HGNC:41906] | -4,20 |
| PROC | [ENSG00000115718](https://www.ensembl.org/id/ENSG00000115718) | protein C, inactivator of coagulation factors Va and VIIIa [Source:HGNC Symbol;Acc:HGNC:9451] | 3,92 |
| PROSER2-AS1 | [ENSG00000225778](https://www.ensembl.org/id/ENSG00000225778) | PROSER2 antisense RNA 1 [Source:HGNC Symbol;Acc:HGNC:27343] | -3,04 |
| PRPH2 | [ENSG00000112619](https://www.ensembl.org/id/ENSG00000112619) | peripherin 2 [Source:HGNC Symbol;Acc:HGNC:9942] | -2,60 |
| PRR15 | [ENSG00000176532](https://www.ensembl.org/id/ENSG00000176532) | proline rich 15 [Source:HGNC Symbol;Acc:HGNC:22310] | -1,93 |
| PRR16 | [ENSG00000184838](https://www.ensembl.org/id/ENSG00000184838) | proline rich 16 [Source:HGNC Symbol;Acc:HGNC:29654] | -1,37 |
| PRR19 | [ENSG00000188368](https://www.ensembl.org/id/ENSG00000188368) | proline rich 19 [Source:HGNC Symbol;Acc:HGNC:33728] | -2,60 |
| PRR33 | [ENSG00000283787](https://www.ensembl.org/id/ENSG00000283787) | proline rich 33 [Source:HGNC Symbol;Acc:HGNC:35118] | -2,20 |
| PRRT1 | [ENSG00000204314](https://www.ensembl.org/id/ENSG00000204314) | proline rich transmembrane protein 1 [Source:HGNC Symbol;Acc:HGNC:13943] | 1,33 |
| PRRX2 | [ENSG00000167157](https://www.ensembl.org/id/ENSG00000167157) | paired related homeobox 2 [Source:HGNC Symbol;Acc:HGNC:21338] | 1,57 |
| PRSS12 | [ENSG00000164099](https://www.ensembl.org/id/ENSG00000164099) | serine protease 12 [Source:HGNC Symbol;Acc:HGNC:9477] | -3,52 |
| PRSS56 | [ENSG00000237412](https://www.ensembl.org/id/ENSG00000237412) | serine protease 56 [Source:HGNC Symbol;Acc:HGNC:39433] | -10,90 |
| PRTN3 | [ENSG00000196415](https://www.ensembl.org/id/ENSG00000196415) | proteinase 3 [Source:HGNC Symbol;Acc:HGNC:9495] | -3,55 |
| PRXL2A | [ENSG00000122378](https://www.ensembl.org/id/ENSG00000122378) | peroxiredoxin like 2A [Source:HGNC Symbol;Acc:HGNC:28651] | -1,21 |
| PRY | [ENSG00000169789](https://www.ensembl.org/id/ENSG00000169789) | PTPN13 like Y-linked [Source:HGNC Symbol;Acc:HGNC:14024] | -3,25 |
| PRY2 | [ENSG00000169807](https://www.ensembl.org/id/ENSG00000169807) | PTPN13 like Y-linked 2 [Source:HGNC Symbol;Acc:HGNC:21504] | -4,77 |
| PSG8-AS1 | [ENSG00000225877](https://www.ensembl.org/id/ENSG00000225877) | PSG8 antisense RNA 1 [Source:HGNC Symbol;Acc:HGNC:52550] | -4,63 |
| PSMA8 | [ENSG00000154611](https://www.ensembl.org/id/ENSG00000154611) | proteasome 20S subunit alpha 8 [Source:HGNC Symbol;Acc:HGNC:22985] | -5,00 |
| PSME4 | [ENSG00000068878](https://www.ensembl.org/id/ENSG00000068878) | proteasome activator subunit 4 [Source:HGNC Symbol;Acc:HGNC:20635] | -1,26 |
| PSTPIP2 | [ENSG00000152229](https://www.ensembl.org/id/ENSG00000152229) | proline-serine-threonine phosphatase interacting protein 2 [Source:HGNC Symbol;Acc:HGNC:9581] | -1,23 |
| PTCD3 | [ENSG00000132300](https://www.ensembl.org/id/ENSG00000132300) | pentatricopeptide repeat domain 3 [Source:HGNC Symbol;Acc:HGNC:24717] | -1,29 |
| PTCHD1 | [ENSG00000165186](https://www.ensembl.org/id/ENSG00000165186) | patched domain containing 1 [Source:HGNC Symbol;Acc:HGNC:26392] | 2,28 |
| PTGDR2 | [ENSG00000183134](https://www.ensembl.org/id/ENSG00000183134) | prostaglandin D2 receptor 2 [Source:HGNC Symbol;Acc:HGNC:4502] | -5,44 |
| PTGER3 | [ENSG00000050628](https://www.ensembl.org/id/ENSG00000050628) | prostaglandin E receptor 3 [Source:HGNC Symbol;Acc:HGNC:9595] | 1,07 |
| PTGES2 | [ENSG00000148334](https://www.ensembl.org/id/ENSG00000148334) | prostaglandin E synthase 2 [Source:HGNC Symbol;Acc:HGNC:17822] | -1,21 |
| PTGES3L | [ENSG00000267060](https://www.ensembl.org/id/ENSG00000267060) | prostaglandin E synthase 3 like [Source:HGNC Symbol;Acc:HGNC:43943] | -2,79 |
| PTGR2 | [ENSG00000140043](https://www.ensembl.org/id/ENSG00000140043) | prostaglandin reductase 2 [Source:HGNC Symbol;Acc:HGNC:20149] | -2,73 |
| PTGR3 | [ENSG00000180011](https://www.ensembl.org/id/ENSG00000180011) | prostaglandin reductase 3 [Source:HGNC Symbol;Acc:HGNC:28697] | -1,41 |
| PTH1R | [ENSG00000160801](https://www.ensembl.org/id/ENSG00000160801) | parathyroid hormone 1 receptor [Source:HGNC Symbol;Acc:HGNC:9608] | 1,16 |
| PTP4A1 | [ENSG00000112245](https://www.ensembl.org/id/ENSG00000112245) | protein tyrosine phosphatase 4A1 [Source:HGNC Symbol;Acc:HGNC:9634] | -1,35 |
| PTP4A3 | [ENSG00000184489](https://www.ensembl.org/id/ENSG00000184489) | protein tyrosine phosphatase 4A3 [Source:HGNC Symbol;Acc:HGNC:9636] | -1,44 |
| PTPN3 | [ENSG00000070159](https://www.ensembl.org/id/ENSG00000070159) | protein tyrosine phosphatase non-receptor type 3 [Source:HGNC Symbol;Acc:HGNC:9655] | -1,98 |
| PTPRK | [ENSG00000152894](https://www.ensembl.org/id/ENSG00000152894) | protein tyrosine phosphatase receptor type K [Source:HGNC Symbol;Acc:HGNC:9674] | 1,15 |
| PTPRU | [ENSG00000060656](https://www.ensembl.org/id/ENSG00000060656) | protein tyrosine phosphatase receptor type U [Source:HGNC Symbol;Acc:HGNC:9683] | -0,88 |
| PTRHD1 | [ENSG00000184924](https://www.ensembl.org/id/ENSG00000184924) | peptidyl-tRNA hydrolase domain containing 1 [Source:HGNC Symbol;Acc:HGNC:33782] | -1,09 |
| PVALB | [ENSG00000100362](https://www.ensembl.org/id/ENSG00000100362) | parvalbumin [Source:HGNC Symbol;Acc:HGNC:9704] | -4,72 |
| PWWP3B | [ENSG00000157502](https://www.ensembl.org/id/ENSG00000157502) | PWWP domain containing 3B [Source:HGNC Symbol;Acc:HGNC:26583] | -4,71 |
| PXMP2 | [ENSG00000176894](https://www.ensembl.org/id/ENSG00000176894) | peroxisomal membrane protein 2 [Source:HGNC Symbol;Acc:HGNC:9716] | -1,39 |
| PYCR1 | [ENSG00000183010](https://www.ensembl.org/id/ENSG00000183010) | pyrroline-5-carboxylate reductase 1 [Source:HGNC Symbol;Acc:HGNC:9721] | -1,69 |
| PYGB | [ENSG00000100994](https://www.ensembl.org/id/ENSG00000100994) | glycogen phosphorylase B [Source:HGNC Symbol;Acc:HGNC:9723] | 1,49 |
| PYGM | [ENSG00000068976](https://www.ensembl.org/id/ENSG00000068976) | glycogen phosphorylase, muscle associated [Source:HGNC Symbol;Acc:HGNC:9726] | -5,20 |
| QKI | [ENSG00000112531](https://www.ensembl.org/id/ENSG00000112531) | QKI, KH domain containing RNA binding [Source:HGNC Symbol;Acc:HGNC:21100] | -1,51 |
| QPRT | [ENSG00000103485](https://www.ensembl.org/id/ENSG00000103485) | quinolinate phosphoribosyltransferase [Source:HGNC Symbol;Acc:HGNC:9755] | -2,46 |
| QRFP | [ENSG00000188710](https://www.ensembl.org/id/ENSG00000188710) | pyroglutamylated RFamide peptide [Source:HGNC Symbol;Acc:HGNC:29982] | 2,46 |
| QRFPR | [ENSG00000186867](https://www.ensembl.org/id/ENSG00000186867) | pyroglutamylated RFamide peptide receptor [Source:HGNC Symbol;Acc:HGNC:15565] | 5,30 |
| QRSL1 | [ENSG00000130348](https://www.ensembl.org/id/ENSG00000130348) | glutaminyl-tRNA amidotransferase subunit QRSL1 [Source:HGNC Symbol;Acc:HGNC:21020] | -1,34 |
| RAB12 | [ENSG00000206418](https://www.ensembl.org/id/ENSG00000206418) | RAB12, member RAS oncogene family [Source:HGNC Symbol;Acc:HGNC:31332] | -1,20 |
| RAB20 | [ENSG00000139832](https://www.ensembl.org/id/ENSG00000139832) | RAB20, member RAS oncogene family [Source:HGNC Symbol;Acc:HGNC:18260] | 1,32 |
| RAB26 | [ENSG00000167964](https://www.ensembl.org/id/ENSG00000167964) | RAB26, member RAS oncogene family [Source:HGNC Symbol;Acc:HGNC:14259] | -2,30 |
| RAB27B | [ENSG00000041353](https://www.ensembl.org/id/ENSG00000041353) | RAB27B, member RAS oncogene family [Source:HGNC Symbol;Acc:HGNC:9767] | -2,81 |
| RAB31 | [ENSG00000168461](https://www.ensembl.org/id/ENSG00000168461) | RAB31, member RAS oncogene family [Source:HGNC Symbol;Acc:HGNC:9771] | 1,13 |
| RAB32 | [ENSG00000118508](https://www.ensembl.org/id/ENSG00000118508) | RAB32, member RAS oncogene family [Source:HGNC Symbol;Acc:HGNC:9772] | 0,96 |
| RAB39A | [ENSG00000179331](https://www.ensembl.org/id/ENSG00000179331) | RAB39A, member RAS oncogene family [Source:HGNC Symbol;Acc:HGNC:16521] | -7,07 |
| RAB4A-AS1 | [ENSG00000177788](https://www.ensembl.org/id/ENSG00000177788) | RAB4A antisense RNA 1 [Source:HGNC Symbol;Acc:HGNC:55934] | -2,70 |
| RAB5IF | [ENSG00000101084](https://www.ensembl.org/id/ENSG00000101084) | RAB5 interacting factor [Source:HGNC Symbol;Acc:HGNC:15870] | -0,95 |
| RAB6B | [ENSG00000154917](https://www.ensembl.org/id/ENSG00000154917) | RAB6B, member RAS oncogene family [Source:HGNC Symbol;Acc:HGNC:14902] | 1,07 |
| RABEP1 | [ENSG00000029725](https://www.ensembl.org/id/ENSG00000029725) | rabaptin, RAB GTPase binding effector protein 1 [Source:HGNC Symbol;Acc:HGNC:17677] | -1,11 |
| RABGGTB | [ENSG00000137955](https://www.ensembl.org/id/ENSG00000137955) | Rab geranylgeranyltransferase subunit beta [Source:HGNC Symbol;Acc:HGNC:9796] | -1,07 |
| RABL6 | [ENSG00000196642](https://www.ensembl.org/id/ENSG00000196642) | RAB, member RAS oncogene family like 6 [Source:HGNC Symbol;Acc:HGNC:24703] | -1,28 |
| RAD23A | [ENSG00000179262](https://www.ensembl.org/id/ENSG00000179262) | RAD23 homolog A, nucleotide excision repair protein [Source:HGNC Symbol;Acc:HGNC:9812] | -1,67 |
| RALYL | [ENSG00000184672](https://www.ensembl.org/id/ENSG00000184672) | RALY RNA binding protein like [Source:HGNC Symbol;Acc:HGNC:27036] | -2,63 |
| RAP1GAP | [ENSG00000076864](https://www.ensembl.org/id/ENSG00000076864) | RAP1 GTPase activating protein [Source:HGNC Symbol;Acc:HGNC:9858] | 2,79 |
| RAPSN | [ENSG00000165917](https://www.ensembl.org/id/ENSG00000165917) | receptor associated protein of the synapse [Source:HGNC Symbol;Acc:HGNC:9863] | -7,10 |
| RARRES1 | [ENSG00000118849](https://www.ensembl.org/id/ENSG00000118849) | retinoic acid receptor responder 1 [Source:HGNC Symbol;Acc:HGNC:9867] | -1,62 |
| RASD2 | [ENSG00000100302](https://www.ensembl.org/id/ENSG00000100302) | RASD family member 2 [Source:HGNC Symbol;Acc:HGNC:18229] | -3,15 |
| RASGEF1A | [ENSG00000198915](https://www.ensembl.org/id/ENSG00000198915) | RasGEF domain family member 1A [Source:HGNC Symbol;Acc:HGNC:24246] | -2,62 |
| RASGEF1C | [ENSG00000146090](https://www.ensembl.org/id/ENSG00000146090) | RasGEF domain family member 1C [Source:HGNC Symbol;Acc:HGNC:27400] | -2,98 |
| RASGRP3 | [ENSG00000152689](https://www.ensembl.org/id/ENSG00000152689) | RAS guanyl releasing protein 3 [Source:HGNC Symbol;Acc:HGNC:14545] | -2,01 |
| RASL10B | [ENSG00000270885](https://www.ensembl.org/id/ENSG00000270885) | RAS like family 10 member B [Source:HGNC Symbol;Acc:HGNC:30295] | -3,44 |
| RASL12 | [ENSG00000103710](https://www.ensembl.org/id/ENSG00000103710) | RAS like family 12 [Source:HGNC Symbol;Acc:HGNC:30289] | 1,45 |
| RBFOX1 | [ENSG00000078328](https://www.ensembl.org/id/ENSG00000078328) | RNA binding fox-1 homolog 1 [Source:HGNC Symbol;Acc:HGNC:18222] | -10,32 |
| RBL1 | [ENSG00000080839](https://www.ensembl.org/id/ENSG00000080839) | RB transcriptional corepressor like 1 [Source:HGNC Symbol;Acc:HGNC:9893] | -2,10 |
| RBM20 | [ENSG00000203867](https://www.ensembl.org/id/ENSG00000203867) | RNA binding motif protein 20 [Source:HGNC Symbol;Acc:HGNC:27424] | -2,08 |
| RBM24 | [ENSG00000112183](https://www.ensembl.org/id/ENSG00000112183) | RNA binding motif protein 24 [Source:HGNC Symbol;Acc:HGNC:21539] | -1,99 |
| RBM38 | [ENSG00000132819](https://www.ensembl.org/id/ENSG00000132819) | RNA binding motif protein 38 [Source:HGNC Symbol;Acc:HGNC:15818] | -1,41 |
| RBM38-AS1 | [ENSG00000218018](https://www.ensembl.org/id/ENSG00000218018) | RBM38 antisense RNA 1 [Source:HGNC Symbol;Acc:HGNC:40725] | -2,50 |
| RBPMS2 | [ENSG00000166831](https://www.ensembl.org/id/ENSG00000166831) | RNA binding protein, mRNA processing factor 2 [Source:HGNC Symbol;Acc:HGNC:19098] | 1,88 |
| RCAN1 | [ENSG00000159200](https://www.ensembl.org/id/ENSG00000159200) | regulator of calcineurin 1 [Source:HGNC Symbol;Acc:HGNC:3040] | 1,85 |
| RCC1L | [ENSG00000274523](https://www.ensembl.org/id/ENSG00000274523) | RCC1 like [Source:HGNC Symbol;Acc:HGNC:14948] | -1,42 |
| RCSD1 | [ENSG00000198771](https://www.ensembl.org/id/ENSG00000198771) | RCSD domain containing 1 [Source:HGNC Symbol;Acc:HGNC:28310] | -2,80 |
| REEP2 | [ENSG00000132563](https://www.ensembl.org/id/ENSG00000132563) | receptor accessory protein 2 [Source:HGNC Symbol;Acc:HGNC:17975] | -1,80 |
| REL-DT | [ENSG00000228414](https://www.ensembl.org/id/ENSG00000228414) | REL divergent transcript [Source:HGNC Symbol;Acc:HGNC:49572] | -5,84 |
| RELN | [ENSG00000189056](https://www.ensembl.org/id/ENSG00000189056) | reelin [Source:HGNC Symbol;Acc:HGNC:9957] | -1,61 |
| RENO1 | [ENSG00000287431](https://www.ensembl.org/id/ENSG00000287431) | regulator of early neurogenesis 1 [Source:HGNC Symbol;Acc:HGNC:54096] | -1,95 |
| RERGL | [ENSG00000111404](https://www.ensembl.org/id/ENSG00000111404) | RERG like [Source:HGNC Symbol;Acc:HGNC:26213] | 1,91 |
| RET | [ENSG00000165731](https://www.ensembl.org/id/ENSG00000165731) | ret proto-oncogene [Source:HGNC Symbol;Acc:HGNC:9967] | -2,80 |
| RETNLB | [ENSG00000163515](https://www.ensembl.org/id/ENSG00000163515) | resistin like beta [Source:HGNC Symbol;Acc:HGNC:20388] | -6,18 |
| RETREG1 | [ENSG00000154153](https://www.ensembl.org/id/ENSG00000154153) | reticulophagy regulator 1 [Source:HGNC Symbol;Acc:HGNC:25964] | -4,48 |
| REXO4 | [ENSG00000148300](https://www.ensembl.org/id/ENSG00000148300) | REX4 homolog, 3'-5' exonuclease [Source:HGNC Symbol;Acc:HGNC:12820] | -1,00 |
| RFLNA | [ENSG00000178882](https://www.ensembl.org/id/ENSG00000178882) | refilin A [Source:HGNC Symbol;Acc:HGNC:27051] | -4,22 |
| RFPL4A | [ENSG00000223638](https://www.ensembl.org/id/ENSG00000223638) | ret finger protein like 4A [Source:HGNC Symbol;Acc:HGNC:16449] | -4,07 |
| RFPL4AL1 | [ENSG00000229292](https://www.ensembl.org/id/ENSG00000229292) | ret finger protein like 4A like 1 [Source:HGNC Symbol;Acc:HGNC:45147] | -5,90 |
| RGCC | [ENSG00000102760](https://www.ensembl.org/id/ENSG00000102760) | regulator of cell cycle [Source:HGNC Symbol;Acc:HGNC:20369] | -1,35 |
| RGL3 | [ENSG00000205517](https://www.ensembl.org/id/ENSG00000205517) | ral guanine nucleotide dissociation stimulator like 3 [Source:HGNC Symbol;Acc:HGNC:30282] | 1,23 |
| RGMA | [ENSG00000182175](https://www.ensembl.org/id/ENSG00000182175) | repulsive guidance molecule BMP co-receptor a [Source:HGNC Symbol;Acc:HGNC:30308] | -1,86 |
| RGS13 | [ENSG00000127074](https://www.ensembl.org/id/ENSG00000127074) | regulator of G protein signaling 13 [Source:HGNC Symbol;Acc:HGNC:9995] | -3,61 |
| RGS16 | [ENSG00000143333](https://www.ensembl.org/id/ENSG00000143333) | regulator of G protein signaling 16 [Source:HGNC Symbol;Acc:HGNC:9997] | 2,12 |
| RGS19 | [ENSG00000171700](https://www.ensembl.org/id/ENSG00000171700) | regulator of G protein signaling 19 [Source:HGNC Symbol;Acc:HGNC:13735] | 1,11 |
| RGS6 | [ENSG00000182732](https://www.ensembl.org/id/ENSG00000182732) | regulator of G protein signaling 6 [Source:HGNC Symbol;Acc:HGNC:10002] | 1,98 |
| RGS9BP | [ENSG00000186326](https://www.ensembl.org/id/ENSG00000186326) | regulator of G protein signaling 9 binding protein [Source:HGNC Symbol;Acc:HGNC:30304] | -7,37 |
| RHOBTB1 | [ENSG00000072422](https://www.ensembl.org/id/ENSG00000072422) | Rho related BTB domain containing 1 [Source:HGNC Symbol;Acc:HGNC:18738] | -1,30 |
| RHOXF2B | [ENSG00000203989](https://www.ensembl.org/id/ENSG00000203989) | Rhox homeobox family member 2B [Source:HGNC Symbol;Acc:HGNC:33519] | -4,68 |
| RILP | [ENSG00000167705](https://www.ensembl.org/id/ENSG00000167705) | Rab interacting lysosomal protein [Source:HGNC Symbol;Acc:HGNC:30266] | -2,12 |
| RILPL1 | [ENSG00000188026](https://www.ensembl.org/id/ENSG00000188026) | Rab interacting lysosomal protein like 1 [Source:HGNC Symbol;Acc:HGNC:26814] | -1,03 |
| RIMBP3B | [ENSG00000274600](https://www.ensembl.org/id/ENSG00000274600) | RIMS binding protein 3B [Source:HGNC Symbol;Acc:HGNC:33891] | 3,41 |
| RIMS4 | [ENSG00000101098](https://www.ensembl.org/id/ENSG00000101098) | regulating synaptic membrane exocytosis 4 [Source:HGNC Symbol;Acc:HGNC:16183] | -1,74 |
| RITA1 | [ENSG00000139405](https://www.ensembl.org/id/ENSG00000139405) | RBPJ interacting and tubulin associated 1 [Source:HGNC Symbol;Acc:HGNC:25925] | -1,40 |
| RMND5A | [ENSG00000153561](https://www.ensembl.org/id/ENSG00000153561) | required for meiotic nuclear division 5 homolog A [Source:HGNC Symbol;Acc:HGNC:25850] | -1,32 |
| RN7SL15P | [ENSG00000264573](https://www.ensembl.org/id/ENSG00000264573) | RNA, 7SL, cytoplasmic 15, pseudogene [Source:HGNC Symbol;Acc:HGNC:46031] | -4,27 |
| RN7SL372P | [ENSG00000242565](https://www.ensembl.org/id/ENSG00000242565) | RNA, 7SL, cytoplasmic 372, pseudogene [Source:HGNC Symbol;Acc:HGNC:46388] | -5,37 |
| RN7SL395P | [ENSG00000244307](https://www.ensembl.org/id/ENSG00000244307) | RNA, 7SL, cytoplasmic 395, pseudogene [Source:HGNC Symbol;Acc:HGNC:46411] | -5,06 |
| RN7SL426P | [ENSG00000263815](https://www.ensembl.org/id/ENSG00000263815) | RNA, 7SL, cytoplasmic 426, pseudogene [Source:HGNC Symbol;Acc:HGNC:46442] | -4,68 |
| RN7SL473P | [ENSG00000277452](https://www.ensembl.org/id/ENSG00000277452) | RNA, 7SL, cytoplasmic 473, pseudogene [Source:HGNC Symbol;Acc:HGNC:46489] | 3,59 |
| RN7SL497P | [ENSG00000240847](https://www.ensembl.org/id/ENSG00000240847) | RNA, 7SL, cytoplasmic 497, pseudogene [Source:HGNC Symbol;Acc:HGNC:46513] | -6,32 |
| RNF103-CHMP3 | [ENSG00000249884](https://www.ensembl.org/id/ENSG00000249884) | RNF103-CHMP3 readthrough [Source:HGNC Symbol;Acc:HGNC:38847] | -4,21 |
| RNF115 | [ENSG00000265491](https://www.ensembl.org/id/ENSG00000265491) | ring finger protein 115 [Source:HGNC Symbol;Acc:HGNC:18154] | -1,69 |
| RNF123 | [ENSG00000164068](https://www.ensembl.org/id/ENSG00000164068) | ring finger protein 123 [Source:HGNC Symbol;Acc:HGNC:21148] | -2,33 |
| RNF128 | [ENSG00000133135](https://www.ensembl.org/id/ENSG00000133135) | ring finger protein 128 [Source:HGNC Symbol;Acc:HGNC:21153] | -10,08 |
| RNF139-DT | [ENSG00000245149](https://www.ensembl.org/id/ENSG00000245149) | RNF139 divergent transcript [Source:HGNC Symbol;Acc:HGNC:48940] | 1,82 |
| RNF144A | [ENSG00000151692](https://www.ensembl.org/id/ENSG00000151692) | ring finger protein 144A [Source:HGNC Symbol;Acc:HGNC:20457] | -1,42 |
| RNF144B | [ENSG00000137393](https://www.ensembl.org/id/ENSG00000137393) | ring finger protein 144B [Source:HGNC Symbol;Acc:HGNC:21578] | -2,77 |
| RNF152 | [ENSG00000176641](https://www.ensembl.org/id/ENSG00000176641) | ring finger protein 152 [Source:HGNC Symbol;Acc:HGNC:26811] | 1,28 |
| RNF157 | [ENSG00000141576](https://www.ensembl.org/id/ENSG00000141576) | ring finger protein 157 [Source:HGNC Symbol;Acc:HGNC:29402] | -3,95 |
| RNF180 | [ENSG00000164197](https://www.ensembl.org/id/ENSG00000164197) | ring finger protein 180 [Source:HGNC Symbol;Acc:HGNC:27752] | 1,06 |
| RNF183 | [ENSG00000165188](https://www.ensembl.org/id/ENSG00000165188) | ring finger protein 183 [Source:HGNC Symbol;Acc:HGNC:28721] | -2,91 |
| RNF208 | [ENSG00000212864](https://www.ensembl.org/id/ENSG00000212864) | ring finger protein 208 [Source:HGNC Symbol;Acc:HGNC:25420] | -1,80 |
| RNF34 | [ENSG00000170633](https://www.ensembl.org/id/ENSG00000170633) | ring finger protein 34 [Source:HGNC Symbol;Acc:HGNC:17297] | -1,16 |
| RNFT1-DT | [ENSG00000267302](https://www.ensembl.org/id/ENSG00000267302) | RNFT1 divergent transcript [Source:HGNC Symbol;Acc:HGNC:51346] | -2,39 |
| RORB | [ENSG00000198963](https://www.ensembl.org/id/ENSG00000198963) | RAR related orphan receptor B [Source:HGNC Symbol;Acc:HGNC:10259] | 1,66 |
| RORC | [ENSG00000143365](https://www.ensembl.org/id/ENSG00000143365) | RAR related orphan receptor C [Source:HGNC Symbol;Acc:HGNC:10260] | -7,97 |
| ROS1 | [ENSG00000047936](https://www.ensembl.org/id/ENSG00000047936) | ROS proto-oncogene 1, receptor tyrosine kinase [Source:HGNC Symbol;Acc:HGNC:10261] | -7,04 |
| RPA2 | [ENSG00000117748](https://www.ensembl.org/id/ENSG00000117748) | replication protein A2 [Source:HGNC Symbol;Acc:HGNC:10290] | -1,03 |
| RPAP1 | [ENSG00000103932](https://www.ensembl.org/id/ENSG00000103932) | RNA polymerase II associated protein 1 [Source:HGNC Symbol;Acc:HGNC:24567] | -0,94 |
| RPL3L | [ENSG00000140986](https://www.ensembl.org/id/ENSG00000140986) | ribosomal protein L3 like [Source:HGNC Symbol;Acc:HGNC:10351] | -11,27 |
| RPP14 | [ENSG00000163684](https://www.ensembl.org/id/ENSG00000163684) | ribonuclease P/MRP subunit p14 [Source:HGNC Symbol;Acc:HGNC:30327] | -1,36 |
| RPP25L | [ENSG00000164967](https://www.ensembl.org/id/ENSG00000164967) | ribonuclease P/MRP subunit p25 like [Source:HGNC Symbol;Acc:HGNC:19909] | -2,15 |
| RPS6KA3 | [ENSG00000177189](https://www.ensembl.org/id/ENSG00000177189) | ribosomal protein S6 kinase A3 [Source:HGNC Symbol;Acc:HGNC:10432] | -0,97 |
| RPSAP58 | [ENSG00000288920](https://www.ensembl.org/id/ENSG00000288920) | ribosomal protein SA pseudogene 58 [Source:HGNC Symbol;Acc:HGNC:36809] | 1,62 |
| RPUSD4 | [ENSG00000165526](https://www.ensembl.org/id/ENSG00000165526) | RNA pseudouridine synthase D4 [Source:HGNC Symbol;Acc:HGNC:25898] | -2,55 |
| RRAGD | [ENSG00000025039](https://www.ensembl.org/id/ENSG00000025039) | Ras related GTP binding D [Source:HGNC Symbol;Acc:HGNC:19903] | -1,96 |
| RSPH6A | [ENSG00000104941](https://www.ensembl.org/id/ENSG00000104941) | radial spoke head 6 homolog A [Source:HGNC Symbol;Acc:HGNC:14241] | -6,48 |
| RSPO3 | [ENSG00000146374](https://www.ensembl.org/id/ENSG00000146374) | R-spondin 3 [Source:HGNC Symbol;Acc:HGNC:20866] | -1,56 |
| RTCA | [ENSG00000137996](https://www.ensembl.org/id/ENSG00000137996) | RNA 3'-terminal phosphate cyclase [Source:HGNC Symbol;Acc:HGNC:17981] | -0,87 |
| RTN1 | [ENSG00000139970](https://www.ensembl.org/id/ENSG00000139970) | reticulon 1 [Source:HGNC Symbol;Acc:HGNC:10467] | 1,43 |
| RTN2 | [ENSG00000125744](https://www.ensembl.org/id/ENSG00000125744) | reticulon 2 [Source:HGNC Symbol;Acc:HGNC:10468] | -4,48 |
| RTN4IP1 | [ENSG00000130347](https://www.ensembl.org/id/ENSG00000130347) | reticulon 4 interacting protein 1 [Source:HGNC Symbol;Acc:HGNC:18647] | -1,62 |
| RTP2 | [ENSG00000198471](https://www.ensembl.org/id/ENSG00000198471) | receptor transporter protein 2 [Source:HGNC Symbol;Acc:HGNC:32486] | -5,11 |
| RUNDC3A | [ENSG00000108309](https://www.ensembl.org/id/ENSG00000108309) | RUN domain containing 3A [Source:HGNC Symbol;Acc:HGNC:16984] | 3,03 |
| RWDD4 | [ENSG00000182552](https://www.ensembl.org/id/ENSG00000182552) | RWD domain containing 4 [Source:HGNC Symbol;Acc:HGNC:23750] | -0,95 |
| RXFP1 | [ENSG00000171509](https://www.ensembl.org/id/ENSG00000171509) | relaxin family peptide receptor 1 [Source:HGNC Symbol;Acc:HGNC:19718] | 3,96 |
| RXRG | [ENSG00000143171](https://www.ensembl.org/id/ENSG00000143171) | retinoid X receptor gamma [Source:HGNC Symbol;Acc:HGNC:10479] | -3,57 |
| RYR1 | [ENSG00000196218](https://www.ensembl.org/id/ENSG00000196218) | ryanodine receptor 1 [Source:HGNC Symbol;Acc:HGNC:10483] | -9,22 |
| RYR2 | [ENSG00000198626](https://www.ensembl.org/id/ENSG00000198626) | ryanodine receptor 2 [Source:HGNC Symbol;Acc:HGNC:10484] | 1,46 |
| RYR3 | [ENSG00000198838](https://www.ensembl.org/id/ENSG00000198838) | ryanodine receptor 3 [Source:HGNC Symbol;Acc:HGNC:10485] | -2,59 |
| S100A1 | [ENSG00000160678](https://www.ensembl.org/id/ENSG00000160678) | S100 calcium binding protein A1 [Source:HGNC Symbol;Acc:HGNC:10486] | -7,85 |
| SAA2 | [ENSG00000134339](https://www.ensembl.org/id/ENSG00000134339) | serum amyloid A2 [Source:HGNC Symbol;Acc:HGNC:10514] | 3,00 |
| SAG | [ENSG00000130561](https://www.ensembl.org/id/ENSG00000130561) | S-antigen visual arrestin [Source:HGNC Symbol;Acc:HGNC:10521] | -4,87 |
| SAGSIN1 | [ENSG00000286075](https://www.ensembl.org/id/ENSG00000286075) | salivary gland specific protein SAGSIN1 [Source:NCBI gene (formerly Entrezgene);Acc:122526779] | -2,15 |
| SALL3 | [ENSG00000256463](https://www.ensembl.org/id/ENSG00000256463) | spalt like transcription factor 3 [Source:HGNC Symbol;Acc:HGNC:10527] | -3,59 |
| SALL4 | [ENSG00000101115](https://www.ensembl.org/id/ENSG00000101115) | spalt like transcription factor 4 [Source:HGNC Symbol;Acc:HGNC:15924] | -2,89 |
| SALRNA1 | [ENSG00000258952](https://www.ensembl.org/id/ENSG00000258952) | senescence associated long non-coding RNA 1 [Source:HGNC Symbol;Acc:HGNC:49001] | -3,11 |
| SAMD11 | [ENSG00000187634](https://www.ensembl.org/id/ENSG00000187634) | sterile alpha motif domain containing 11 [Source:HGNC Symbol;Acc:HGNC:28706] | 1,58 |
| SAMD12 | [ENSG00000177570](https://www.ensembl.org/id/ENSG00000177570) | sterile alpha motif domain containing 12 [Source:HGNC Symbol;Acc:HGNC:31750] | 1,83 |
| SAMD13 | [ENSG00000203943](https://www.ensembl.org/id/ENSG00000203943) | sterile alpha motif domain containing 13 [Source:HGNC Symbol;Acc:HGNC:24582] | -2,71 |
| SAMD4A-AS1 | [ENSG00000285774](https://www.ensembl.org/id/ENSG00000285774) | SAMD4A antisense RNA 1 [Source:HGNC Symbol;Acc:HGNC:54803] | -3,22 |
| SAMM50 | [ENSG00000100347](https://www.ensembl.org/id/ENSG00000100347) | SAMM50 sorting and assembly machinery component [Source:HGNC Symbol;Acc:HGNC:24276] | -1,26 |
| SAR1B | [ENSG00000152700](https://www.ensembl.org/id/ENSG00000152700) | secretion associated Ras related GTPase 1B [Source:HGNC Symbol;Acc:HGNC:10535] | -1,31 |
| SBK1 | [ENSG00000188322](https://www.ensembl.org/id/ENSG00000188322) | SH3 domain binding kinase 1 [Source:HGNC Symbol;Acc:HGNC:17699] | -5,81 |
| SBK2 | [ENSG00000187550](https://www.ensembl.org/id/ENSG00000187550) | SH3 domain binding kinase family member 2 [Source:HGNC Symbol;Acc:HGNC:34416] | -10,40 |
| SBSPON | [ENSG00000164764](https://www.ensembl.org/id/ENSG00000164764) | somatomedin B and thrombospondin type 1 domain containing [Source:HGNC Symbol;Acc:HGNC:30362] | 1,70 |
| SCARNA10 | [ENSG00000239002](https://www.ensembl.org/id/ENSG00000239002) | small Cajal body-specific RNA 10 [Source:HGNC Symbol;Acc:HGNC:32567] | -3,62 |
| SCAT8 | [ENSG00000236345](https://www.ensembl.org/id/ENSG00000236345) | S-phase cancer associated transcript 8 [Source:HGNC Symbol;Acc:HGNC:40967] | -2,77 |
| SCD | [ENSG00000099194](https://www.ensembl.org/id/ENSG00000099194) | stearoyl-CoA desaturase [Source:HGNC Symbol;Acc:HGNC:10571] | 2,45 |
| SCG3 | [ENSG00000104112](https://www.ensembl.org/id/ENSG00000104112) | secretogranin III [Source:HGNC Symbol;Acc:HGNC:13707] | 3,90 |
| SCG5 | [ENSG00000166922](https://www.ensembl.org/id/ENSG00000166922) | secretogranin V [Source:HGNC Symbol;Acc:HGNC:10816] | -3,20 |
| SCGB1D2 | [ENSG00000124935](https://www.ensembl.org/id/ENSG00000124935) | secretoglobin family 1D member 2 [Source:HGNC Symbol;Acc:HGNC:18396] | -6,60 |
| SCGB2A2 | [ENSG00000110484](https://www.ensembl.org/id/ENSG00000110484) | secretoglobin family 2A member 2 [Source:HGNC Symbol;Acc:HGNC:7050] | -5,36 |
| SCHIP1 | [ENSG00000151967](https://www.ensembl.org/id/ENSG00000151967) | schwannomin interacting protein 1 [Source:HGNC Symbol;Acc:HGNC:15678] | -2,58 |
| SCIRT | [ENSG00000237686](https://www.ensembl.org/id/ENSG00000237686) | stem cell inhibitory RNA transcript [Source:HGNC Symbol;Acc:HGNC:55341] | -6,52 |
| SCN1B | [ENSG00000105711](https://www.ensembl.org/id/ENSG00000105711) | sodium voltage-gated channel beta subunit 1 [Source:HGNC Symbol;Acc:HGNC:10586] | -3,96 |
| SCN2B | [ENSG00000149575](https://www.ensembl.org/id/ENSG00000149575) | sodium voltage-gated channel beta subunit 2 [Source:HGNC Symbol;Acc:HGNC:10589] | -4,94 |
| SCN3B | [ENSG00000166257](https://www.ensembl.org/id/ENSG00000166257) | sodium voltage-gated channel beta subunit 3 [Source:HGNC Symbol;Acc:HGNC:20665] | -3,84 |
| SCN4A | [ENSG00000007314](https://www.ensembl.org/id/ENSG00000007314) | sodium voltage-gated channel alpha subunit 4 [Source:HGNC Symbol;Acc:HGNC:10591] | -4,79 |
| SCN7A | [ENSG00000136546](https://www.ensembl.org/id/ENSG00000136546) | sodium voltage-gated channel alpha subunit 7 [Source:HGNC Symbol;Acc:HGNC:10594] | -2,36 |
| SCT | [ENSG00000070031](https://www.ensembl.org/id/ENSG00000070031) | secretin [Source:HGNC Symbol;Acc:HGNC:10607] | -5,77 |
| SCTR-AS1 | [ENSG00000231013](https://www.ensembl.org/id/ENSG00000231013) | SCTR antisense RNA 1 [Source:HGNC Symbol;Acc:HGNC:40516] | -4,90 |
| SCUBE2 | [ENSG00000175356](https://www.ensembl.org/id/ENSG00000175356) | signal peptide, CUB domain and EGF like domain containing 2 [Source:HGNC Symbol;Acc:HGNC:30425] | -1,53 |
| SCX | [ENSG00000260428](https://www.ensembl.org/id/ENSG00000260428) | scleraxis bHLH transcription factor [Source:HGNC Symbol;Acc:HGNC:32322] | -1,43 |
| SDC4 | [ENSG00000124145](https://www.ensembl.org/id/ENSG00000124145) | syndecan 4 [Source:HGNC Symbol;Acc:HGNC:10661] | 1,43 |
| SDHA | [ENSG00000073578](https://www.ensembl.org/id/ENSG00000073578) | succinate dehydrogenase complex flavoprotein subunit A [Source:HGNC Symbol;Acc:HGNC:10680] | -2,17 |
| SDHB | [ENSG00000117118](https://www.ensembl.org/id/ENSG00000117118) | succinate dehydrogenase complex iron sulfur subunit B [Source:HGNC Symbol;Acc:HGNC:10681] | -1,76 |
| SDHC | [ENSG00000143252](https://www.ensembl.org/id/ENSG00000143252) | succinate dehydrogenase complex subunit C [Source:HGNC Symbol;Acc:HGNC:10682] | -1,11 |
| SDHD | [ENSG00000204370](https://www.ensembl.org/id/ENSG00000204370) | succinate dehydrogenase complex subunit D [Source:HGNC Symbol;Acc:HGNC:10683] | -1,28 |
| SDK1 | [ENSG00000146555](https://www.ensembl.org/id/ENSG00000146555) | sidekick cell adhesion molecule 1 [Source:HGNC Symbol;Acc:HGNC:19307] | 1,32 |
| SEC14L1 | [ENSG00000129657](https://www.ensembl.org/id/ENSG00000129657) | SEC14 like lipid binding 1 [Source:HGNC Symbol;Acc:HGNC:10698] | -1,03 |
| SEC14L5 | [ENSG00000103184](https://www.ensembl.org/id/ENSG00000103184) | SEC14 like lipid binding 5 [Source:HGNC Symbol;Acc:HGNC:29032] | -2,57 |
| SEMA3C | [ENSG00000075223](https://www.ensembl.org/id/ENSG00000075223) | semaphorin 3C [Source:HGNC Symbol;Acc:HGNC:10725] | -1,28 |
| SEMA4D | [ENSG00000187764](https://www.ensembl.org/id/ENSG00000187764) | semaphorin 4D [Source:HGNC Symbol;Acc:HGNC:10732] | -2,69 |
| SEMA6C | [ENSG00000143434](https://www.ensembl.org/id/ENSG00000143434) | semaphorin 6C [Source:HGNC Symbol;Acc:HGNC:10740] | -4,32 |
| SEPTIN5 | [ENSG00000184702](https://www.ensembl.org/id/ENSG00000184702) | septin 5 [Source:HGNC Symbol;Acc:HGNC:9164] | 1,10 |
| SERF1B | [ENSG00000205572](https://www.ensembl.org/id/ENSG00000205572) | small EDRK-rich factor 1B [Source:HGNC Symbol;Acc:HGNC:10756] | 1,31 |
| SERINC2 | [ENSG00000168528](https://www.ensembl.org/id/ENSG00000168528) | serine incorporator 2 [Source:HGNC Symbol;Acc:HGNC:23231] | -3,43 |
| SERPINA5 | [ENSG00000188488](https://www.ensembl.org/id/ENSG00000188488) | serpin family A member 5 [Source:HGNC Symbol;Acc:HGNC:8723] | -2,79 |
| SERPINF1 | [ENSG00000132386](https://www.ensembl.org/id/ENSG00000132386) | serpin family F member 1 [Source:HGNC Symbol;Acc:HGNC:8824] | -1,54 |
| SERTAD4 | [ENSG00000082497](https://www.ensembl.org/id/ENSG00000082497) | SERTA domain containing 4 [Source:HGNC Symbol;Acc:HGNC:25236] | 1,27 |
| SESN1 | [ENSG00000080546](https://www.ensembl.org/id/ENSG00000080546) | sestrin 1 [Source:HGNC Symbol;Acc:HGNC:21595] | -1,80 |
| SEZ6L | [ENSG00000100095](https://www.ensembl.org/id/ENSG00000100095) | seizure related 6 homolog like [Source:HGNC Symbol;Acc:HGNC:10763] | -5,63 |
| SGCD | [ENSG00000170624](https://www.ensembl.org/id/ENSG00000170624) | sarcoglycan delta [Source:HGNC Symbol;Acc:HGNC:10807] | -1,21 |
| SGCG | [ENSG00000102683](https://www.ensembl.org/id/ENSG00000102683) | sarcoglycan gamma [Source:HGNC Symbol;Acc:HGNC:10809] | -5,36 |
| SGMS2 | [ENSG00000164023](https://www.ensembl.org/id/ENSG00000164023) | sphingomyelin synthase 2 [Source:HGNC Symbol;Acc:HGNC:28395] | 1,67 |
| SGO1-AS1 | [ENSG00000231304](https://www.ensembl.org/id/ENSG00000231304) | SGO1 antisense RNA 1 [Source:HGNC Symbol;Acc:HGNC:41081] | -3,45 |
| SH2B2 | [ENSG00000160999](https://www.ensembl.org/id/ENSG00000160999) | SH2B adaptor protein 2 [Source:HGNC Symbol;Acc:HGNC:17381] | -3,03 |
| SH3BGR | [ENSG00000185437](https://www.ensembl.org/id/ENSG00000185437) | SH3 domain binding glutamate rich protein [Source:HGNC Symbol;Acc:HGNC:10822] | -1,96 |
| SH3KBP1 | [ENSG00000147010](https://www.ensembl.org/id/ENSG00000147010) | SH3 domain containing kinase binding protein 1 [Source:HGNC Symbol;Acc:HGNC:13867] | -1,18 |
| SH3RF2 | [ENSG00000156463](https://www.ensembl.org/id/ENSG00000156463) | SH3 domain containing ring finger 2 [Source:HGNC Symbol;Acc:HGNC:26299] | -1,29 |
| SHB | [ENSG00000107338](https://www.ensembl.org/id/ENSG00000107338) | SH2 domain containing adaptor protein B [Source:HGNC Symbol;Acc:HGNC:10838] | 1,17 |
| SHISA2 | [ENSG00000180730](https://www.ensembl.org/id/ENSG00000180730) | shisa family member 2 [Source:HGNC Symbol;Acc:HGNC:20366] | -3,10 |
| SHISA4 | [ENSG00000198892](https://www.ensembl.org/id/ENSG00000198892) | shisa family member 4 [Source:HGNC Symbol;Acc:HGNC:27139] | -2,81 |
| SHISA8 | [ENSG00000234965](https://www.ensembl.org/id/ENSG00000234965) | shisa family member 8 [Source:HGNC Symbol;Acc:HGNC:18351] | -3,13 |
| SHMT2 | [ENSG00000182199](https://www.ensembl.org/id/ENSG00000182199) | serine hydroxymethyltransferase 2 [Source:HGNC Symbol;Acc:HGNC:10852] | -0,84 |
| SIAH2 | [ENSG00000181788](https://www.ensembl.org/id/ENSG00000181788) | siah E3 ubiquitin protein ligase 2 [Source:HGNC Symbol;Acc:HGNC:10858] | 0,93 |
| SIM1 | [ENSG00000112246](https://www.ensembl.org/id/ENSG00000112246) | SIM bHLH transcription factor 1 [Source:HGNC Symbol;Acc:HGNC:10882] | -6,22 |
| SIRT2 | [ENSG00000068903](https://www.ensembl.org/id/ENSG00000068903) | sirtuin 2 [Source:HGNC Symbol;Acc:HGNC:10886] | -2,56 |
| SIRT3 | [ENSG00000142082](https://www.ensembl.org/id/ENSG00000142082) | sirtuin 3 [Source:HGNC Symbol;Acc:HGNC:14931] | -1,12 |
| SIRT4 | [ENSG00000089163](https://www.ensembl.org/id/ENSG00000089163) | sirtuin 4 [Source:HGNC Symbol;Acc:HGNC:14932] | -3,02 |
| SIRT5 | [ENSG00000124523](https://www.ensembl.org/id/ENSG00000124523) | sirtuin 5 [Source:HGNC Symbol;Acc:HGNC:14933] | -1,40 |
| SIX1 | [ENSG00000126778](https://www.ensembl.org/id/ENSG00000126778) | SIX homeobox 1 [Source:HGNC Symbol;Acc:HGNC:10887] | -4,20 |
| SIX4 | [ENSG00000100625](https://www.ensembl.org/id/ENSG00000100625) | SIX homeobox 4 [Source:HGNC Symbol;Acc:HGNC:10890] | -3,65 |
| SLC10A4 | [ENSG00000145248](https://www.ensembl.org/id/ENSG00000145248) | solute carrier family 10 member 4 [Source:HGNC Symbol;Acc:HGNC:22980] | -2,54 |
| SLC12A1 | [ENSG00000074803](https://www.ensembl.org/id/ENSG00000074803) | solute carrier family 12 member 1 [Source:HGNC Symbol;Acc:HGNC:10910] | -2,36 |
| SLC16A10 | [ENSG00000112394](https://www.ensembl.org/id/ENSG00000112394) | solute carrier family 16 member 10 [Source:HGNC Symbol;Acc:HGNC:17027] | -4,07 |
| SLC16A12 | [ENSG00000152779](https://www.ensembl.org/id/ENSG00000152779) | solute carrier family 16 member 12 [Source:HGNC Symbol;Acc:HGNC:23094] | -2,49 |
| SLC16A5 | [ENSG00000170190](https://www.ensembl.org/id/ENSG00000170190) | solute carrier family 16 member 5 [Source:HGNC Symbol;Acc:HGNC:10926] | -1,70 |
| SLC1A3 | [ENSG00000079215](https://www.ensembl.org/id/ENSG00000079215) | solute carrier family 1 member 3 [Source:HGNC Symbol;Acc:HGNC:10941] | -2,29 |
| SLC1A5 | [ENSG00000105281](https://www.ensembl.org/id/ENSG00000105281) | solute carrier family 1 member 5 [Source:HGNC Symbol;Acc:HGNC:10943] | 1,25 |
| SLC24A2 | [ENSG00000155886](https://www.ensembl.org/id/ENSG00000155886) | solute carrier family 24 member 2 [Source:HGNC Symbol;Acc:HGNC:10976] | -7,57 |
| SLC25A10 | [ENSG00000183048](https://www.ensembl.org/id/ENSG00000183048) | solute carrier family 25 member 10 [Source:HGNC Symbol;Acc:HGNC:10980] | -1,62 |
| SLC25A11 | [ENSG00000108528](https://www.ensembl.org/id/ENSG00000108528) | solute carrier family 25 member 11 [Source:HGNC Symbol;Acc:HGNC:10981] | -1,93 |
| SLC25A20 | [ENSG00000178537](https://www.ensembl.org/id/ENSG00000178537) | solute carrier family 25 member 20 [Source:HGNC Symbol;Acc:HGNC:1421] | -1,27 |
| SLC25A30 | [ENSG00000174032](https://www.ensembl.org/id/ENSG00000174032) | solute carrier family 25 member 30 [Source:HGNC Symbol;Acc:HGNC:27371] | -1,17 |
| SLC25A34 | [ENSG00000162461](https://www.ensembl.org/id/ENSG00000162461) | solute carrier family 25 member 34 [Source:HGNC Symbol;Acc:HGNC:27653] | -3,17 |
| SLC25A4 | [ENSG00000151729](https://www.ensembl.org/id/ENSG00000151729) | solute carrier family 25 member 4 [Source:HGNC Symbol;Acc:HGNC:10990] | -2,97 |
| SLC25A42 | [ENSG00000181035](https://www.ensembl.org/id/ENSG00000181035) | solute carrier family 25 member 42 [Source:HGNC Symbol;Acc:HGNC:28380] | -2,23 |
| SLC26A9 | [ENSG00000174502](https://www.ensembl.org/id/ENSG00000174502) | solute carrier family 26 member 9 [Source:HGNC Symbol;Acc:HGNC:14469] | -5,58 |
| SLC28A1 | [ENSG00000156222](https://www.ensembl.org/id/ENSG00000156222) | solute carrier family 28 member 1 [Source:HGNC Symbol;Acc:HGNC:11001] | -2,83 |
| SLC29A2 | [ENSG00000174669](https://www.ensembl.org/id/ENSG00000174669) | solute carrier family 29 member 2 [Source:HGNC Symbol;Acc:HGNC:11004] | -3,88 |
| SLC2A1-DT | [ENSG00000227533](https://www.ensembl.org/id/ENSG00000227533) | SLC2A1 divergent transcript [Source:HGNC Symbol;Acc:HGNC:44187] | 1,85 |
| SLC2A13 | [ENSG00000151229](https://www.ensembl.org/id/ENSG00000151229) | solute carrier family 2 member 13 [Source:HGNC Symbol;Acc:HGNC:15956] | 1,36 |
| SLC2A4 | [ENSG00000181856](https://www.ensembl.org/id/ENSG00000181856) | solute carrier family 2 member 4 [Source:HGNC Symbol;Acc:HGNC:11009] | -4,01 |
| SLC2A5 | [ENSG00000142583](https://www.ensembl.org/id/ENSG00000142583) | solute carrier family 2 member 5 [Source:HGNC Symbol;Acc:HGNC:11010] | -5,13 |
| SLC30A10 | [ENSG00000196660](https://www.ensembl.org/id/ENSG00000196660) | solute carrier family 30 member 10 [Source:HGNC Symbol;Acc:HGNC:25355] | -3,46 |
| SLC30A2 | [ENSG00000158014](https://www.ensembl.org/id/ENSG00000158014) | solute carrier family 30 member 2 [Source:HGNC Symbol;Acc:HGNC:11013] | -5,09 |
| SLC30A8 | [ENSG00000164756](https://www.ensembl.org/id/ENSG00000164756) | solute carrier family 30 member 8 [Source:HGNC Symbol;Acc:HGNC:20303] | -5,90 |
| SLC35B1 | [ENSG00000121073](https://www.ensembl.org/id/ENSG00000121073) | solute carrier family 35 member B1 [Source:HGNC Symbol;Acc:HGNC:20798] | -1,31 |
| SLC36A2 | [ENSG00000186335](https://www.ensembl.org/id/ENSG00000186335) | solute carrier family 36 member 2 [Source:HGNC Symbol;Acc:HGNC:18762] | -9,49 |
| SLC37A4 | [ENSG00000137700](https://www.ensembl.org/id/ENSG00000137700) | solute carrier family 37 member 4 [Source:HGNC Symbol;Acc:HGNC:4061] | -1,31 |
| SLC38A1 | [ENSG00000111371](https://www.ensembl.org/id/ENSG00000111371) | solute carrier family 38 member 1 [Source:HGNC Symbol;Acc:HGNC:13447] | 1,24 |
| SLC38A3 | [ENSG00000188338](https://www.ensembl.org/id/ENSG00000188338) | solute carrier family 38 member 3 [Source:HGNC Symbol;Acc:HGNC:18044] | -4,28 |
| SLC41A1 | [ENSG00000133065](https://www.ensembl.org/id/ENSG00000133065) | solute carrier family 41 member 1 [Source:HGNC Symbol;Acc:HGNC:19429] | -1,62 |
| SLC45A3 | [ENSG00000158715](https://www.ensembl.org/id/ENSG00000158715) | solute carrier family 45 member 3 [Source:HGNC Symbol;Acc:HGNC:8642] | -2,46 |
| SLC47A1 | [ENSG00000142494](https://www.ensembl.org/id/ENSG00000142494) | solute carrier family 47 member 1 [Source:HGNC Symbol;Acc:HGNC:25588] | -1,62 |
| SLC4A1 | [ENSG00000004939](https://www.ensembl.org/id/ENSG00000004939) | solute carrier family 4 member 1 (Diego blood group) [Source:HGNC Symbol;Acc:HGNC:11027] | 3,68 |
| SLC4A11 | [ENSG00000088836](https://www.ensembl.org/id/ENSG00000088836) | solute carrier family 4 member 11 [Source:HGNC Symbol;Acc:HGNC:16438] | -3,28 |
| SLC6A1 | [ENSG00000157103](https://www.ensembl.org/id/ENSG00000157103) | solute carrier family 6 member 1 [Source:HGNC Symbol;Acc:HGNC:11042] | -1,82 |
| SLC6A10P | [ENSG00000290985](https://www.ensembl.org/id/ENSG00000290985) | solute carrier family 6 member 10, pseudogene [Source:NCBI gene (formerly Entrezgene);Acc:386757] | -8,16 |
| SLC6A15 | [ENSG00000072041](https://www.ensembl.org/id/ENSG00000072041) | solute carrier family 6 member 15 [Source:HGNC Symbol;Acc:HGNC:13621] | 2,90 |
| SLC6A17 | [ENSG00000197106](https://www.ensembl.org/id/ENSG00000197106) | solute carrier family 6 member 17 [Source:HGNC Symbol;Acc:HGNC:31399] | 2,94 |
| SLC6A7 | [ENSG00000011083](https://www.ensembl.org/id/ENSG00000011083) | solute carrier family 6 member 7 [Source:HGNC Symbol;Acc:HGNC:11054] | 5,12 |
| SLC7A11-AS1 | [ENSG00000250033](https://www.ensembl.org/id/ENSG00000250033) | SLC7A11 antisense RNA 1 [Source:HGNC Symbol;Acc:HGNC:44064] | -3,52 |
| SLC8A1 | [ENSG00000183023](https://www.ensembl.org/id/ENSG00000183023) | solute carrier family 8 member A1 [Source:HGNC Symbol;Acc:HGNC:11068] | 1,53 |
| SLC8A3 | [ENSG00000100678](https://www.ensembl.org/id/ENSG00000100678) | solute carrier family 8 member A3 [Source:HGNC Symbol;Acc:HGNC:11070] | -7,57 |
| SLC9A2 | [ENSG00000115616](https://www.ensembl.org/id/ENSG00000115616) | solute carrier family 9 member A2 [Source:HGNC Symbol;Acc:HGNC:11072] | -4,14 |
| SLC9A7 | [ENSG00000065923](https://www.ensembl.org/id/ENSG00000065923) | solute carrier family 9 member A7 [Source:HGNC Symbol;Acc:HGNC:17123] | -1,85 |
| SLCO2B1 | [ENSG00000137491](https://www.ensembl.org/id/ENSG00000137491) | solute carrier organic anion transporter family member 2B1 [Source:HGNC Symbol;Acc:HGNC:10962] | -1,68 |
| SLCO5A1 | [ENSG00000137571](https://www.ensembl.org/id/ENSG00000137571) | solute carrier organic anion transporter family member 5A1 [Source:HGNC Symbol;Acc:HGNC:19046] | -1,75 |
| SLITRK4 | [ENSG00000179542](https://www.ensembl.org/id/ENSG00000179542) | SLIT and NTRK like family member 4 [Source:HGNC Symbol;Acc:HGNC:23502] | -2,62 |
| SLN | [ENSG00000170290](https://www.ensembl.org/id/ENSG00000170290) | sarcolipin [Source:HGNC Symbol;Acc:HGNC:11089] | -9,23 |
| SMAD6 | [ENSG00000137834](https://www.ensembl.org/id/ENSG00000137834) | SMAD family member 6 [Source:HGNC Symbol;Acc:HGNC:6772] | 1,67 |
| SMAD9-IT1 | [ENSG00000236711](https://www.ensembl.org/id/ENSG00000236711) | SMAD9 intronic transcript 1 [Source:HGNC Symbol;Acc:HGNC:39963] | -4,64 |
| SMCO1 | [ENSG00000214097](https://www.ensembl.org/id/ENSG00000214097) | single-pass membrane protein with coiled-coil domains 1 [Source:HGNC Symbol;Acc:HGNC:27407] | -13,50 |
| SMIM20 | [ENSG00000250317](https://www.ensembl.org/id/ENSG00000250317) | small integral membrane protein 20 [Source:HGNC Symbol;Acc:HGNC:37260] | -1,26 |
| SMIM30 | [ENSG00000214194](https://www.ensembl.org/id/ENSG00000214194) | small integral membrane protein 30 [Source:HGNC Symbol;Acc:HGNC:48953] | -1,02 |
| SMIM38 | [ENSG00000284713](https://www.ensembl.org/id/ENSG00000284713) | small integral membrane protein 38 [Source:HGNC Symbol;Acc:HGNC:54074] | 2,47 |
| SMPDL3A | [ENSG00000172594](https://www.ensembl.org/id/ENSG00000172594) | sphingomyelin phosphodiesterase acid like 3A [Source:HGNC Symbol;Acc:HGNC:17389] | -2,37 |
| SMPX | [ENSG00000091482](https://www.ensembl.org/id/ENSG00000091482) | small muscle protein X-linked [Source:HGNC Symbol;Acc:HGNC:11122] | -10,34 |
| SMTNL1 | [ENSG00000214872](https://www.ensembl.org/id/ENSG00000214872) | smoothelin like 1 [Source:HGNC Symbol;Acc:HGNC:32394] | -11,32 |
| SMTNL2 | [ENSG00000188176](https://www.ensembl.org/id/ENSG00000188176) | smoothelin like 2 [Source:HGNC Symbol;Acc:HGNC:24764] | -7,42 |
| SMYD1 | [ENSG00000115593](https://www.ensembl.org/id/ENSG00000115593) | SET and MYND domain containing 1 [Source:HGNC Symbol;Acc:HGNC:20986] | -11,38 |
| SNAI3 | [ENSG00000185669](https://www.ensembl.org/id/ENSG00000185669) | snail family transcriptional repressor 3 [Source:HGNC Symbol;Acc:HGNC:18411] | -5,94 |
| SNAI3-AS1 | [ENSG00000260630](https://www.ensembl.org/id/ENSG00000260630) | SNAI3 antisense RNA 1 [Source:HGNC Symbol;Acc:HGNC:28327] | -1,83 |
| SNAP25 | [ENSG00000132639](https://www.ensembl.org/id/ENSG00000132639) | synaptosome associated protein 25 [Source:HGNC Symbol;Acc:HGNC:11132] | 2,65 |
| SNCA | [ENSG00000145335](https://www.ensembl.org/id/ENSG00000145335) | synuclein alpha [Source:HGNC Symbol;Acc:HGNC:11138] | 1,98 |
| SNCB | [ENSG00000074317](https://www.ensembl.org/id/ENSG00000074317) | synuclein beta [Source:HGNC Symbol;Acc:HGNC:11140] | -7,83 |
| SNCG | [ENSG00000173267](https://www.ensembl.org/id/ENSG00000173267) | synuclein gamma [Source:HGNC Symbol;Acc:HGNC:11141] | 1,47 |
| SNHG7 | [ENSG00000233016](https://www.ensembl.org/id/ENSG00000233016) | small nucleolar RNA host gene 7 [Source:HGNC Symbol;Acc:HGNC:28254] | -1,22 |
| SNORC | [ENSG00000182600](https://www.ensembl.org/id/ENSG00000182600) | secondary ossification center associated regulator of chondrocyte maturation [Source:HGNC Symbol;Acc:HGNC:33763] | -1,97 |
| SNRNP25 | [ENSG00000161981](https://www.ensembl.org/id/ENSG00000161981) | small nuclear ribonucleoprotein U11/U12 subunit 25 [Source:HGNC Symbol;Acc:HGNC:14161] | -1,45 |
| SNUPN | [ENSG00000169371](https://www.ensembl.org/id/ENSG00000169371) | snurportin 1 [Source:HGNC Symbol;Acc:HGNC:14245] | -0,95 |
| SNX20 | [ENSG00000167208](https://www.ensembl.org/id/ENSG00000167208) | sorting nexin 20 [Source:HGNC Symbol;Acc:HGNC:30390] | -1,74 |
| SOCS4 | [ENSG00000180008](https://www.ensembl.org/id/ENSG00000180008) | suppressor of cytokine signaling 4 [Source:HGNC Symbol;Acc:HGNC:19392] | 0,86 |
| SORBS2 | [ENSG00000154556](https://www.ensembl.org/id/ENSG00000154556) | sorbin and SH3 domain containing 2 [Source:HGNC Symbol;Acc:HGNC:24098] | 1,82 |
| SORBS2-AS1 | [ENSG00000233110](https://www.ensembl.org/id/ENSG00000233110) | SORBS2 antisense RNA 1 [Source:HGNC Symbol;Acc:HGNC:41039] | 2,74 |
| SORL1 | [ENSG00000137642](https://www.ensembl.org/id/ENSG00000137642) | sortilin related receptor 1 [Source:HGNC Symbol;Acc:HGNC:11185] | 1,45 |
| SOX11 | [ENSG00000176887](https://www.ensembl.org/id/ENSG00000176887) | SRY-box transcription factor 11 [Source:HGNC Symbol;Acc:HGNC:11191] | -4,75 |
| SOX8 | [ENSG00000005513](https://www.ensembl.org/id/ENSG00000005513) | SRY-box transcription factor 8 [Source:HGNC Symbol;Acc:HGNC:11203] | -1,47 |
| SOX9-AS1 | [ENSG00000234899](https://www.ensembl.org/id/ENSG00000234899) | SOX9 antisense RNA 1 [Source:HGNC Symbol;Acc:HGNC:49321] | -7,60 |
| SP2-DT | [ENSG00000264920](https://www.ensembl.org/id/ENSG00000264920) | SP2 divergent transcript [Source:HGNC Symbol;Acc:HGNC:55330] | -1,99 |
| SP7 | [ENSG00000170374](https://www.ensembl.org/id/ENSG00000170374) | Sp7 transcription factor [Source:HGNC Symbol;Acc:HGNC:17321] | -5,18 |
| SPAG7 | [ENSG00000091640](https://www.ensembl.org/id/ENSG00000091640) | sperm associated antigen 7 [Source:HGNC Symbol;Acc:HGNC:11216] | -1,37 |
| SPATA22 | [ENSG00000141255](https://www.ensembl.org/id/ENSG00000141255) | spermatogenesis associated 22 [Source:HGNC Symbol;Acc:HGNC:30705] | -3,39 |
| SPATA2L | [ENSG00000158792](https://www.ensembl.org/id/ENSG00000158792) | spermatogenesis associated 2 like [Source:HGNC Symbol;Acc:HGNC:28393] | 1,58 |
| SPATA31A6 | [ENSG00000185775](https://www.ensembl.org/id/ENSG00000185775) | SPATA31 subfamily A member 6 [Source:HGNC Symbol;Acc:HGNC:32006] | -5,63 |
| SPATS1 | [ENSG00000249481](https://www.ensembl.org/id/ENSG00000249481) | spermatogenesis associated serine rich 1 [Source:HGNC Symbol;Acc:HGNC:22957] | -3,59 |
| SPC25 | [ENSG00000152253](https://www.ensembl.org/id/ENSG00000152253) | SPC25 component of NDC80 kinetochore complex [Source:HGNC Symbol;Acc:HGNC:24031] | 3,64 |
| SPECC1 | [ENSG00000128487](https://www.ensembl.org/id/ENSG00000128487) | sperm antigen with calponin homology and coiled-coil domains 1 [Source:HGNC Symbol;Acc:HGNC:30615] | 0,92 |
| SPEM3 | [ENSG00000283439](https://www.ensembl.org/id/ENSG00000283439) | SPEM family member 3 [Source:HGNC Symbol;Acc:HGNC:53651] | -6,03 |
| SPEN-AS1 | [ENSG00000179743](https://www.ensembl.org/id/ENSG00000179743) | SPEN antisense RNA 1 [Source:HGNC Symbol;Acc:HGNC:55937] | -1,58 |
| SPINK2 | [ENSG00000128040](https://www.ensembl.org/id/ENSG00000128040) | serine peptidase inhibitor Kazal type 2 [Source:HGNC Symbol;Acc:HGNC:11245] | -4,28 |
| SPINT2 | [ENSG00000167642](https://www.ensembl.org/id/ENSG00000167642) | serine peptidase inhibitor, Kunitz type 2 [Source:HGNC Symbol;Acc:HGNC:11247] | -1,65 |
| SPOCK1 | [ENSG00000152377](https://www.ensembl.org/id/ENSG00000152377) | SPARC (osteonectin), cwcv and kazal like domains proteoglycan 1 [Source:HGNC Symbol;Acc:HGNC:11251] | -1,37 |
| SPPL2A | [ENSG00000138600](https://www.ensembl.org/id/ENSG00000138600) | signal peptide peptidase like 2A [Source:HGNC Symbol;Acc:HGNC:30227] | -1,13 |
| SPRY4 | [ENSG00000187678](https://www.ensembl.org/id/ENSG00000187678) | sprouty RTK signaling antagonist 4 [Source:HGNC Symbol;Acc:HGNC:15533] | 1,49 |
| SPTB | [ENSG00000070182](https://www.ensembl.org/id/ENSG00000070182) | spectrin beta, erythrocytic [Source:HGNC Symbol;Acc:HGNC:11274] | -3,94 |
| SPX | [ENSG00000134548](https://www.ensembl.org/id/ENSG00000134548) | spexin hormone [Source:HGNC Symbol;Acc:HGNC:28139] | -4,26 |
| SRD5A2 | [ENSG00000277893](https://www.ensembl.org/id/ENSG00000277893) | steroid 5 alpha-reductase 2 [Source:HGNC Symbol;Acc:HGNC:11285] | -3,57 |
| SRL | [ENSG00000185739](https://www.ensembl.org/id/ENSG00000185739) | sarcalumenin [Source:HGNC Symbol;Acc:HGNC:11295] | -4,45 |
| SRP14-DT | [ENSG00000248508](https://www.ensembl.org/id/ENSG00000248508) | SRP14 divergent transcript [Source:HGNC Symbol;Acc:HGNC:48619] | -1,43 |
| SRPK3 | [ENSG00000184343](https://www.ensembl.org/id/ENSG00000184343) | SRSF protein kinase 3 [Source:HGNC Symbol;Acc:HGNC:11402] | -2,20 |
| SSH2 | [ENSG00000141298](https://www.ensembl.org/id/ENSG00000141298) | slingshot protein phosphatase 2 [Source:HGNC Symbol;Acc:HGNC:30580] | -2,15 |
| SSTR2 | [ENSG00000180616](https://www.ensembl.org/id/ENSG00000180616) | somatostatin receptor 2 [Source:HGNC Symbol;Acc:HGNC:11331] | 2,34 |
| SSTR4 | [ENSG00000132671](https://www.ensembl.org/id/ENSG00000132671) | somatostatin receptor 4 [Source:HGNC Symbol;Acc:HGNC:11333] | -5,47 |
| ST3GAL3 | [ENSG00000126091](https://www.ensembl.org/id/ENSG00000126091) | ST3 beta-galactoside alpha-2,3-sialyltransferase 3 [Source:HGNC Symbol;Acc:HGNC:10866] | -1,10 |
| ST3GAL3-AS1 | [ENSG00000229444](https://www.ensembl.org/id/ENSG00000229444) | ST3GAL3 antisense RNA 1 [Source:HGNC Symbol;Acc:HGNC:40529] | -10,85 |
| ST6GALNAC2 | [ENSG00000070731](https://www.ensembl.org/id/ENSG00000070731) | ST6 N-acetylgalactosaminide alpha-2,6-sialyltransferase 2 [Source:HGNC Symbol;Acc:HGNC:10867] | -2,40 |
| ST6GALNAC3 | [ENSG00000184005](https://www.ensembl.org/id/ENSG00000184005) | ST6 N-acetylgalactosaminide alpha-2,6-sialyltransferase 3 [Source:HGNC Symbol;Acc:HGNC:19343] | 1,47 |
| ST8SIA2 | [ENSG00000140557](https://www.ensembl.org/id/ENSG00000140557) | ST8 alpha-N-acetyl-neuraminide alpha-2,8-sialyltransferase 2 [Source:HGNC Symbol;Acc:HGNC:10870] | -6,84 |
| ST8SIA5 | [ENSG00000101638](https://www.ensembl.org/id/ENSG00000101638) | ST8 alpha-N-acetyl-neuraminide alpha-2,8-sialyltransferase 5 [Source:HGNC Symbol;Acc:HGNC:17827] | -4,88 |
| ST8SIA5-DT | [ENSG00000270112](https://www.ensembl.org/id/ENSG00000270112) | ST8SIA5 divergent transcript [Source:HGNC Symbol;Acc:HGNC:55267] | -3,77 |
| STAB2 | [ENSG00000136011](https://www.ensembl.org/id/ENSG00000136011) | stabilin 2 [Source:HGNC Symbol;Acc:HGNC:18629] | 3,19 |
| STAC3 | [ENSG00000185482](https://www.ensembl.org/id/ENSG00000185482) | SH3 and cysteine rich domain 3 [Source:HGNC Symbol;Acc:HGNC:28423] | -8,68 |
| STARD10 | [ENSG00000214530](https://www.ensembl.org/id/ENSG00000214530) | StAR related lipid transfer domain containing 10 [Source:HGNC Symbol;Acc:HGNC:10666] | -1,51 |
| STARD6 | [ENSG00000174448](https://www.ensembl.org/id/ENSG00000174448) | StAR related lipid transfer domain containing 6 [Source:HGNC Symbol;Acc:HGNC:18066] | -3,21 |
| STATH | [ENSG00000126549](https://www.ensembl.org/id/ENSG00000126549) | statherin [Source:HGNC Symbol;Acc:HGNC:11369] | -8,52 |
| STAU2 | [ENSG00000040341](https://www.ensembl.org/id/ENSG00000040341) | staufen double-stranded RNA binding protein 2 [Source:HGNC Symbol;Acc:HGNC:11371] | -1,46 |
| STBD1 | [ENSG00000118804](https://www.ensembl.org/id/ENSG00000118804) | starch binding domain 1 [Source:HGNC Symbol;Acc:HGNC:24854] | -1,78 |
| STIMATE | [ENSG00000213533](https://www.ensembl.org/id/ENSG00000213533) | STIM activating enhancer [Source:HGNC Symbol;Acc:HGNC:30526] | 1,06 |
| STK25 | [ENSG00000115694](https://www.ensembl.org/id/ENSG00000115694) | serine/threonine kinase 25 [Source:HGNC Symbol;Acc:HGNC:11404] | -1,34 |
| STK33 | [ENSG00000130413](https://www.ensembl.org/id/ENSG00000130413) | serine/threonine kinase 33 [Source:HGNC Symbol;Acc:HGNC:14568] | -2,90 |
| STON2 | [ENSG00000140022](https://www.ensembl.org/id/ENSG00000140022) | stonin 2 [Source:HGNC Symbol;Acc:HGNC:30652] | -2,07 |
| STPG2-AS1 | [ENSG00000251620](https://www.ensembl.org/id/ENSG00000251620) | STPG2 antisense RNA 1 [Source:HGNC Symbol;Acc:HGNC:41209] | -5,30 |
| STRADB | [ENSG00000082146](https://www.ensembl.org/id/ENSG00000082146) | STE20 related adaptor beta [Source:HGNC Symbol;Acc:HGNC:13205] | -1,56 |
| STRIP2 | [ENSG00000128578](https://www.ensembl.org/id/ENSG00000128578) | striatin interacting protein 2 [Source:HGNC Symbol;Acc:HGNC:22209] | -6,19 |
| STRIT1 | [ENSG00000240045](https://www.ensembl.org/id/ENSG00000240045) | small transmembrane regulator of ion transport 1 [Source:HGNC Symbol;Acc:HGNC:52297] | -11,18 |
| STUM | [ENSG00000203685](https://www.ensembl.org/id/ENSG00000203685) | stum, mechanosensory transduction mediator homolog [Source:HGNC Symbol;Acc:HGNC:30491] | 1,96 |
| STX19 | [ENSG00000178750](https://www.ensembl.org/id/ENSG00000178750) | syntaxin 19 [Source:HGNC Symbol;Acc:HGNC:19300] | 3,50 |
| STYK1 | [ENSG00000060140](https://www.ensembl.org/id/ENSG00000060140) | serine/threonine/tyrosine kinase 1 [Source:HGNC Symbol;Acc:HGNC:18889] | -3,39 |
| STYXL2 | [ENSG00000198842](https://www.ensembl.org/id/ENSG00000198842) | serine/threonine/tyrosine interacting like 2 [Source:HGNC Symbol;Acc:HGNC:25034] | -5,92 |
| SUCLA2 | [ENSG00000136143](https://www.ensembl.org/id/ENSG00000136143) | succinate-CoA ligase ADP-forming subunit beta [Source:HGNC Symbol;Acc:HGNC:11448] | -1,69 |
| SUCLG1 | [ENSG00000163541](https://www.ensembl.org/id/ENSG00000163541) | succinate-CoA ligase GDP/ADP-forming subunit alpha [Source:HGNC Symbol;Acc:HGNC:11449] | -1,34 |
| SUCLG2 | [ENSG00000172340](https://www.ensembl.org/id/ENSG00000172340) | succinate-CoA ligase GDP-forming subunit beta [Source:HGNC Symbol;Acc:HGNC:11450] | -1,06 |
| SUCLG2-DT | [ENSG00000241316](https://www.ensembl.org/id/ENSG00000241316) | SUCLG2 divergent transcript [Source:HGNC Symbol;Acc:HGNC:49643] | -2,30 |
| SULT1A4 | [ENSG00000213648](https://www.ensembl.org/id/ENSG00000213648) | sulfotransferase family 1A member 4 [Source:HGNC Symbol;Acc:HGNC:30004] | -2,52 |
| SUNO1 | [ENSG00000277013](https://www.ensembl.org/id/ENSG00000277013) | S-phase upregulated non-coding 1 [Source:HGNC Symbol;Acc:HGNC:55385] | -2,88 |
| SUSD2 | [ENSG00000099994](https://www.ensembl.org/id/ENSG00000099994) | sushi domain containing 2 [Source:HGNC Symbol;Acc:HGNC:30667] | 1,85 |
| SUSD4 | [ENSG00000143502](https://www.ensembl.org/id/ENSG00000143502) | sushi domain containing 4 [Source:HGNC Symbol;Acc:HGNC:25470] | -1,92 |
| SVIL | [ENSG00000197321](https://www.ensembl.org/id/ENSG00000197321) | supervillin [Source:HGNC Symbol;Acc:HGNC:11480] | -2,20 |
| SYN1 | [ENSG00000008056](https://www.ensembl.org/id/ENSG00000008056) | synapsin I [Source:HGNC Symbol;Acc:HGNC:11494] | -1,62 |
| SYNC | [ENSG00000162520](https://www.ensembl.org/id/ENSG00000162520) | syncoilin, intermediate filament protein [Source:HGNC Symbol;Acc:HGNC:28897] | -1,38 |
| SYNGR1 | [ENSG00000100321](https://www.ensembl.org/id/ENSG00000100321) | synaptogyrin 1 [Source:HGNC Symbol;Acc:HGNC:11498] | -2,17 |
| SYNPO | [ENSG00000171992](https://www.ensembl.org/id/ENSG00000171992) | synaptopodin [Source:HGNC Symbol;Acc:HGNC:30672] | -2,33 |
| SYNPO2L | [ENSG00000166317](https://www.ensembl.org/id/ENSG00000166317) | synaptopodin 2 like [Source:HGNC Symbol;Acc:HGNC:23532] | -8,49 |
| SYNPO2L-AS1 | [ENSG00000271848](https://www.ensembl.org/id/ENSG00000271848) | SYNPO2L antisense RNA 1 [Source:HGNC Symbol;Acc:HGNC:55242] | -5,18 |
| SYPL2 | [ENSG00000143028](https://www.ensembl.org/id/ENSG00000143028) | synaptophysin like 2 [Source:HGNC Symbol;Acc:HGNC:27638] | -3,40 |
| SYT5 | [ENSG00000129990](https://www.ensembl.org/id/ENSG00000129990) | synaptotagmin 5 [Source:HGNC Symbol;Acc:HGNC:11513] | -3,21 |
| SYTL2 | [ENSG00000137501](https://www.ensembl.org/id/ENSG00000137501) | synaptotagmin like 2 [Source:HGNC Symbol;Acc:HGNC:15585] | 1,81 |
| TAAR5 | [ENSG00000135569](https://www.ensembl.org/id/ENSG00000135569) | trace amine associated receptor 5 [Source:HGNC Symbol;Acc:HGNC:30236] | -5,11 |
| TACC2 | [ENSG00000138162](https://www.ensembl.org/id/ENSG00000138162) | transforming acidic coiled-coil containing protein 2 [Source:HGNC Symbol;Acc:HGNC:11523] | -2,00 |
| TACO1 | [ENSG00000136463](https://www.ensembl.org/id/ENSG00000136463) | translational activator of cytochrome c oxidase I [Source:HGNC Symbol;Acc:HGNC:24316] | -1,67 |
| TAFA5 | [ENSG00000219438](https://www.ensembl.org/id/ENSG00000219438) | TAFA chemokine like family member 5 [Source:HGNC Symbol;Acc:HGNC:21592] | -1,90 |
| TAL2 | [ENSG00000186051](https://www.ensembl.org/id/ENSG00000186051) | TAL bHLH transcription factor 2 [Source:HGNC Symbol;Acc:HGNC:11557] | -5,95 |
| TARS2 | [ENSG00000143374](https://www.ensembl.org/id/ENSG00000143374) | threonyl-tRNA synthetase 2, mitochondrial [Source:HGNC Symbol;Acc:HGNC:30740] | -1,64 |
| TARS3 | [ENSG00000185418](https://www.ensembl.org/id/ENSG00000185418) | threonyl-tRNA synthetase 3 [Source:HGNC Symbol;Acc:HGNC:24728] | -1,36 |
| TAS1R3 | [ENSG00000169962](https://www.ensembl.org/id/ENSG00000169962) | taste 1 receptor member 3 [Source:HGNC Symbol;Acc:HGNC:15661] | -2,06 |
| TATDN1 | [ENSG00000147687](https://www.ensembl.org/id/ENSG00000147687) | TatD DNase domain containing 1 [Source:HGNC Symbol;Acc:HGNC:24220] | -1,13 |
| TBC1D3 | [ENSG00000274611](https://www.ensembl.org/id/ENSG00000274611) | TBC1 domain family member 3 [Source:HGNC Symbol;Acc:HGNC:19031] | -7,75 |
| TBC1D3D | [ENSG00000274419](https://www.ensembl.org/id/ENSG00000274419) | TBC1 domain family member 3D [Source:HGNC Symbol;Acc:HGNC:28944] | -8,25 |
| TBC1D3E | [ENSG00000278599](https://www.ensembl.org/id/ENSG00000278599) | TBC1 domain family member 3E [Source:HGNC Symbol;Acc:HGNC:27071] | -7,89 |
| TBC1D3F | [ENSG00000275954](https://www.ensembl.org/id/ENSG00000275954) | TBC1 domain family member 3F [Source:HGNC Symbol;Acc:HGNC:18257] | -7,55 |
| TBC1D3K | [ENSG00000273513](https://www.ensembl.org/id/ENSG00000273513) | TBC1 domain family member 3K [Source:HGNC Symbol;Acc:HGNC:51245] | 5,55 |
| TBC1D4 | [ENSG00000136111](https://www.ensembl.org/id/ENSG00000136111) | TBC1 domain family member 4 [Source:HGNC Symbol;Acc:HGNC:19165] | -2,52 |
| TBC1D8 | [ENSG00000204634](https://www.ensembl.org/id/ENSG00000204634) | TBC1 domain family member 8 [Source:HGNC Symbol;Acc:HGNC:17791] | -1,21 |
| TBRG4 | [ENSG00000136270](https://www.ensembl.org/id/ENSG00000136270) | transforming growth factor beta regulator 4 [Source:HGNC Symbol;Acc:HGNC:17443] | -1,26 |
| TBX1 | [ENSG00000184058](https://www.ensembl.org/id/ENSG00000184058) | T-box transcription factor 1 [Source:HGNC Symbol;Acc:HGNC:11592] | -3,38 |
| TBX15 | [ENSG00000092607](https://www.ensembl.org/id/ENSG00000092607) | T-box transcription factor 15 [Source:HGNC Symbol;Acc:HGNC:11594] | -5,07 |
| TBX18 | [ENSG00000112837](https://www.ensembl.org/id/ENSG00000112837) | T-box transcription factor 18 [Source:HGNC Symbol;Acc:HGNC:11595] | 1,14 |
| TBX2 | [ENSG00000121068](https://www.ensembl.org/id/ENSG00000121068) | T-box transcription factor 2 [Source:HGNC Symbol;Acc:HGNC:11597] | 1,12 |
| TBXA2R | [ENSG00000006638](https://www.ensembl.org/id/ENSG00000006638) | thromboxane A2 receptor [Source:HGNC Symbol;Acc:HGNC:11608] | 1,35 |
| TCAP | [ENSG00000173991](https://www.ensembl.org/id/ENSG00000173991) | titin-cap [Source:HGNC Symbol;Acc:HGNC:11610] | -10,40 |
| TCEA3 | [ENSG00000204219](https://www.ensembl.org/id/ENSG00000204219) | transcription elongation factor A3 [Source:HGNC Symbol;Acc:HGNC:11615] | -2,61 |
| TCERG1L | [ENSG00000176769](https://www.ensembl.org/id/ENSG00000176769) | transcription elongation regulator 1 like [Source:HGNC Symbol;Acc:HGNC:23533] | -3,61 |
| TCF15 | [ENSG00000125878](https://www.ensembl.org/id/ENSG00000125878) | transcription factor 15 [Source:HGNC Symbol;Acc:HGNC:11627] | -3,03 |
| TDH-AS1 | [ENSG00000255020](https://www.ensembl.org/id/ENSG00000255020) | TDH antisense RNA 1 [Source:HGNC Symbol;Acc:HGNC:55510] | -5,72 |
| TEAD1 | [ENSG00000187079](https://www.ensembl.org/id/ENSG00000187079) | TEA domain transcription factor 1 [Source:HGNC Symbol;Acc:HGNC:11714] | -1,88 |
| TEAD4 | [ENSG00000197905](https://www.ensembl.org/id/ENSG00000197905) | TEA domain transcription factor 4 [Source:HGNC Symbol;Acc:HGNC:11717] | -2,09 |
| TECRL | [ENSG00000205678](https://www.ensembl.org/id/ENSG00000205678) | trans-2,3-enoyl-CoA reductase like [Source:HGNC Symbol;Acc:HGNC:27365] | -14,64 |
| TENM3 | [ENSG00000218336](https://www.ensembl.org/id/ENSG00000218336) | teneurin transmembrane protein 3 [Source:HGNC Symbol;Acc:HGNC:29944] | -3,11 |
| TENM3-AS1 | [ENSG00000177822](https://www.ensembl.org/id/ENSG00000177822) | TENM3 antisense RNA 1 [Source:HGNC Symbol;Acc:HGNC:28076] | -2,65 |
| TENT5B | [ENSG00000158246](https://www.ensembl.org/id/ENSG00000158246) | terminal nucleotidyltransferase 5B [Source:HGNC Symbol;Acc:HGNC:28273] | 1,93 |
| TFAP2D | [ENSG00000008197](https://www.ensembl.org/id/ENSG00000008197) | transcription factor AP-2 delta [Source:HGNC Symbol;Acc:HGNC:15581] | -7,10 |
| TFDP1 | [ENSG00000198176](https://www.ensembl.org/id/ENSG00000198176) | transcription factor Dp-1 [Source:HGNC Symbol;Acc:HGNC:11749] | -0,97 |
| TGFB2-AS1 | [ENSG00000232480](https://www.ensembl.org/id/ENSG00000232480) | TGFB2 antisense RNA 1 (head to head) [Source:HGNC Symbol;Acc:HGNC:50628] | -3,64 |
| TGFBI | [ENSG00000120708](https://www.ensembl.org/id/ENSG00000120708) | transforming growth factor beta induced [Source:HGNC Symbol;Acc:HGNC:11771] | 2,22 |
| THBD | [ENSG00000178726](https://www.ensembl.org/id/ENSG00000178726) | thrombomodulin [Source:HGNC Symbol;Acc:HGNC:11784] | 1,75 |
| THBS1 | [ENSG00000137801](https://www.ensembl.org/id/ENSG00000137801) | thrombospondin 1 [Source:HGNC Symbol;Acc:HGNC:11785] | 3,32 |
| THBS1-AS1 | [ENSG00000278621](https://www.ensembl.org/id/ENSG00000278621) | THBS1 antisense RNA 1 [Source:HGNC Symbol;Acc:HGNC:55224] | 2,62 |
| THBS4 | [ENSG00000113296](https://www.ensembl.org/id/ENSG00000113296) | thrombospondin 4 [Source:HGNC Symbol;Acc:HGNC:11788] | -3,59 |
| THEM4 | [ENSG00000159445](https://www.ensembl.org/id/ENSG00000159445) | thioesterase superfamily member 4 [Source:HGNC Symbol;Acc:HGNC:17947] | -1,14 |
| THNSL1 | [ENSG00000185875](https://www.ensembl.org/id/ENSG00000185875) | threonine synthase like 1 [Source:HGNC Symbol;Acc:HGNC:26160] | -1,98 |
| THNSL2 | [ENSG00000144115](https://www.ensembl.org/id/ENSG00000144115) | threonine synthase like 2 [Source:HGNC Symbol;Acc:HGNC:25602] | -1,14 |
| TICAM2-AS1 | [ENSG00000249249](https://www.ensembl.org/id/ENSG00000249249) | TICAM2 antisense RNA 1 [Source:HGNC Symbol;Acc:HGNC:55575] | -1,72 |
| TICRR | [ENSG00000140534](https://www.ensembl.org/id/ENSG00000140534) | TOPBP1 interacting checkpoint and replication regulator [Source:HGNC Symbol;Acc:HGNC:28704] | 1,87 |
| TIGD4 | [ENSG00000169989](https://www.ensembl.org/id/ENSG00000169989) | tigger transposable element derived 4 [Source:HGNC Symbol;Acc:HGNC:18335] | -4,27 |
| TIMM13 | [ENSG00000099800](https://www.ensembl.org/id/ENSG00000099800) | translocase of inner mitochondrial membrane 13 [Source:HGNC Symbol;Acc:HGNC:11816] | -1,13 |
| TIMM17A | [ENSG00000134375](https://www.ensembl.org/id/ENSG00000134375) | translocase of inner mitochondrial membrane 17A [Source:HGNC Symbol;Acc:HGNC:17315] | -1,07 |
| TIMM21 | [ENSG00000075336](https://www.ensembl.org/id/ENSG00000075336) | translocase of inner mitochondrial membrane 21 [Source:HGNC Symbol;Acc:HGNC:25010] | -1,62 |
| TIMM8B | [ENSG00000150779](https://www.ensembl.org/id/ENSG00000150779) | translocase of inner mitochondrial membrane 8 homolog B [Source:HGNC Symbol;Acc:HGNC:11818] | -1,10 |
| TK1 | [ENSG00000167900](https://www.ensembl.org/id/ENSG00000167900) | thymidine kinase 1 [Source:HGNC Symbol;Acc:HGNC:11830] | -1,98 |
| TLCD3B | [ENSG00000149926](https://www.ensembl.org/id/ENSG00000149926) | TLC domain containing 3B [Source:HGNC Symbol;Acc:HGNC:25295] | -6,14 |
| TLE2 | [ENSG00000065717](https://www.ensembl.org/id/ENSG00000065717) | TLE family member 2, transcriptional corepressor [Source:HGNC Symbol;Acc:HGNC:11838] | -1,57 |
| TLL2 | [ENSG00000095587](https://www.ensembl.org/id/ENSG00000095587) | tolloid like 2 [Source:HGNC Symbol;Acc:HGNC:11844] | -5,56 |
| TLR7 | [ENSG00000196664](https://www.ensembl.org/id/ENSG00000196664) | toll like receptor 7 [Source:HGNC Symbol;Acc:HGNC:15631] | -2,38 |
| TM4SF19 | [ENSG00000145107](https://www.ensembl.org/id/ENSG00000145107) | transmembrane 4 L six family member 19 [Source:HGNC Symbol;Acc:HGNC:25167] | -3,51 |
| TM6SF1 | [ENSG00000136404](https://www.ensembl.org/id/ENSG00000136404) | transmembrane 6 superfamily member 1 [Source:HGNC Symbol;Acc:HGNC:11860] | -3,60 |
| TMC1 | [ENSG00000165091](https://www.ensembl.org/id/ENSG00000165091) | transmembrane channel like 1 [Source:HGNC Symbol;Acc:HGNC:16513] | -5,74 |
| TMC2 | [ENSG00000149488](https://www.ensembl.org/id/ENSG00000149488) | transmembrane channel like 2 [Source:HGNC Symbol;Acc:HGNC:16527] | -3,09 |
| TMEM108 | [ENSG00000144868](https://www.ensembl.org/id/ENSG00000144868) | transmembrane protein 108 [Source:HGNC Symbol;Acc:HGNC:28451] | -2,12 |
| TMEM11 | [ENSG00000178307](https://www.ensembl.org/id/ENSG00000178307) | transmembrane protein 11 [Source:HGNC Symbol;Acc:HGNC:16823] | -1,41 |
| TMEM114 | [ENSG00000232258](https://www.ensembl.org/id/ENSG00000232258) | transmembrane protein 114 [Source:HGNC Symbol;Acc:HGNC:33227] | -5,77 |
| TMEM126A | [ENSG00000171202](https://www.ensembl.org/id/ENSG00000171202) | transmembrane protein 126A [Source:HGNC Symbol;Acc:HGNC:25382] | -1,02 |
| TMEM132B | [ENSG00000139364](https://www.ensembl.org/id/ENSG00000139364) | transmembrane protein 132B [Source:HGNC Symbol;Acc:HGNC:29397] | -2,96 |
| TMEM132E | [ENSG00000181291](https://www.ensembl.org/id/ENSG00000181291) | transmembrane protein 132E [Source:HGNC Symbol;Acc:HGNC:26991] | 3,27 |
| TMEM134 | [ENSG00000172663](https://www.ensembl.org/id/ENSG00000172663) | transmembrane protein 134 [Source:HGNC Symbol;Acc:HGNC:26142] | -1,20 |
| TMEM143 | [ENSG00000161558](https://www.ensembl.org/id/ENSG00000161558) | transmembrane protein 143 [Source:HGNC Symbol;Acc:HGNC:25603] | -2,95 |
| TMEM14B | [ENSG00000137210](https://www.ensembl.org/id/ENSG00000137210) | transmembrane protein 14B [Source:HGNC Symbol;Acc:HGNC:21384] | -0,93 |
| TMEM164 | [ENSG00000157600](https://www.ensembl.org/id/ENSG00000157600) | transmembrane protein 164 [Source:HGNC Symbol;Acc:HGNC:26217] | -0,88 |
| TMEM165 | [ENSG00000134851](https://www.ensembl.org/id/ENSG00000134851) | transmembrane protein 165 [Source:HGNC Symbol;Acc:HGNC:30760] | 0,97 |
| TMEM178B | [ENSG00000261115](https://www.ensembl.org/id/ENSG00000261115) | transmembrane protein 178B [Source:HGNC Symbol;Acc:HGNC:44112] | -3,81 |
| TMEM181 | [ENSG00000146433](https://www.ensembl.org/id/ENSG00000146433) | transmembrane protein 181 [Source:HGNC Symbol;Acc:HGNC:20958] | 1,23 |
| TMEM182 | [ENSG00000170417](https://www.ensembl.org/id/ENSG00000170417) | transmembrane protein 182 [Source:HGNC Symbol;Acc:HGNC:26391] | -4,26 |
| TMEM201 | [ENSG00000188807](https://www.ensembl.org/id/ENSG00000188807) | transmembrane protein 201 [Source:HGNC Symbol;Acc:HGNC:33719] | -1,80 |
| TMEM229A | [ENSG00000234224](https://www.ensembl.org/id/ENSG00000234224) | transmembrane protein 229A [Source:HGNC Symbol;Acc:HGNC:37279] | -4,75 |
| TMEM233 | [ENSG00000224982](https://www.ensembl.org/id/ENSG00000224982) | transmembrane protein 233 [Source:HGNC Symbol;Acc:HGNC:37219] | -4,47 |
[truncated: 32,932 more chars]
