# Supplementary material for: Role of inflammatory signaling pathways involving the CD40–CD40L–TRAF cascade in diabetes and hypertension—insights from animal and human studies
Source: Basic Res Cardiol. 2024 Mar 30;119(4):1–18. doi: 10.1007/s00395-024-01045-1 (PMC11319409; doi:10.1007/s00395-024-01045-1)
Supplement: Supplementary file 5 — Supplementary file5 (DOCX 526 KB) [file 395_2024_1045_MOESM5_ESM.docx]

| **Suppl. Table S6. RNA-Seq data: Assignment of DEGs to specific signaling clusters.** | | | | | |
| --- | --- | --- | --- | --- | --- |
| **CHD+HT vs. CHD / UP>2** | **Gene** | **Pathway/function** | **TRAF interaction** | **Log_2_ fold change** | **Ref.** |
| **CD40L-CD40-TRAF**  **signaling / interactors** | ACAT1 | Synthesis of ketone bodies / LDL clearance | TRAF4 | -2,8 | [1, 2] |
|  | ACTN3 | (Skeletal) muscle contraction | TRAF1/2 | 8127,7 | [2-4] |
|  | APOA1 | Platelet degranulation / HDL clearance / plasma lipoprotein clearance and remodeling | CD40L | 20,5 | [2, 5] |
|  | APOO | Associates with HDL, LDL, and VLDL | TRAF2 | 4,6 | [6, 7] |
|  | ARHGEF6 | RAC1 guanine nucleotide exchange factor | TRAF2 | 2,7 | [3, 7] |
|  | ATP1B1 | Cardiac conduction / cell surface interactions at the vascular wall | TRAF3/6 | 15,6 | [2, 8] |
|  | CECR2 | Chromatin remodeling / DNA damage response | TRAF7 | 20,4 | [7, 9] |
|  | CEP85 | Microtubule organizing center / involved in cell-cycle progression | TRAF2/4 | 23,9 | [3, 7, 10] |
|  | CHCHD3 | Inner mitochondrial membrane scaffold protein | TRAF1 | 5,3 | [4, 7] |
|  | COA6 | Associates with cytochrome c oxidase | TRAF6 | 3,7 | [7, 11] |
|  | COX5B | Respiratory electron transport | TRAF1 | 5,0 | [4, 7] |
|  | CRYAB | Associated with Cardiomyopathy / lens crystallins | CD40L | 6,9 | [7, 12] |
|  | CUL5 | adaptive immune system / cytokine signaling | TRAF2/6 | 3,0 | [2, 13, 14] |
|  | DBI | Lipid metabolism / diazepam binding inhibitor | TRAF3 | 2,9 | [7, 15] |
|  | DGCR6 | DiGeorge syndrome / neuronal crest cell migration | TRAF1/2 | 48,9 | [7, 16] |
|  | DUSP13 | Protein-tyrosine phosphatase / cell proliferation and differentiation | TRAF1 | 34228,2 | [3, 7] |
|  | ECH1 | Peroxisomal lipid metabolism | TRAF1/3 | 5,2 | [4, 7, 17, 18] |
|  | ECI2 | Peroxisomal lipid metabolism / Fatty acid metabolism | TRAF1 | 4,3 | [7, 17, 18] |
|  | ECSIT | IL-1 receptor signaling pathway / TRAF6 mediated induction of NF-κb pathway | TRAF6 | 2,7 | [7, 19, 20] |
|  | FGG | Fibrin clot formation / platelet degranulation and aggregation / adaptive immune system | TRAF6 | 40,6 | [2, 18] |
|  | FREM2 | Epidermal interactions | TRAF2 | 130,0 | [7, 10] |
|  | FRMD1 | Cytoskeleton | TRAF7 | 6,7 | [7, 18] |
|  | FXR1 | Associated with myopathy, RNA-binding protein | TRAF2/7 | 5,9 | [7, 18, 21] |
|  | GRAP2 | Involved in leukocyte-specific protein-tyrosine kinase signaling | TRAF1 | 19,4 | [7, 22] |
|  | HOXC8 | Differentiation of white and brown adipocytes | TRAF1 | 3,6 | [3, 7] |
|  | HPR | Haptoglobin-related protein that binds hemoglobin | CD40L | 9,0 | [5, 7] |
|  | IP6K3 | Inositol phosphate metabolism | TRAF2/3 | 61,1 | [3, 7] |
|  | IPO13 | Nuclear transport protein | CD40 | 4,0 | [7, 18] |
|  | KIAA1217 | Involved in skeletal system development | TRAF1/2 | 4,5 | [3, 4, 7] |
|  | KRT1 | Innate immune system | TRAF6 | 229,5 | [2, 23] |
|  | KRT23 | Structural integrity of epithelial cells | TRAF7 | 142,8 | [7, 18] |
|  | KRT31 | Keratin gene family | TRAF4 | 2461,7 | [3, 4, 7] |
|  | MAP2K1 | Cytokine signaling / TRAF6 mediated induction of NF-κB | TRAF6  mediated | 3,0 | [2] |
|  | MAP2K6 | Myogenesis / Cytokine signaling / TRAF6 mediated induction of NF-κB | TRAF6 | 15,8 | [2, 24] |
|  | MAPKAPK3 | Cytokine signaling / TRAF6 mediated induction of NF-κB | TRAF6  mediated | 6,1 | [2] |
|  | MAPT | Promotes microtubule assembly and stability | TRAF6 | 20,5 | [7, 25] |
|  | ME1 | NADP-dependent enzyme that generates NADPH for fatty acid biosynthesis | TRAF3 | 3,1 | [7, 15] |
|  | MEF2C | Myogenesis / Cytokine signaling / TRAF6 mediated induction of NF-κB | TRAF6 mediated | 6,0 | [2] |
|  | MOXD1 | Enable copper ion binding activity | TRAF2 | 23,0 | [7, 10] |
|  | MYOZ1 | Cardiac and skeletal muscle / associated with myopathy | TRAF1 | 63,6 | [3, 7] |
|  | NEDD4L | Innate Immune system / autophagy | TRAF3 | 6,2 | [7, 26] |
|  | OPTN | TNFR1 induced pro-apoptotic signaling / NF-κB mediated | TNFR1  mediated | 3,7 | [2] |
|  | OTUD1 | TNFR1 induced pro-apoptotic signaling / NF-κB mediated | TNFR1  mediated | 4,9 | [2] |
|  | PINK1 | Protect cells from stress-induced mitochondrial dysfunction | TRAF3/6 | 8,7 | [7, 27-29] |
|  | PITX1 | Organ development | TRAF1 | 7,9 | [3, 7] |
|  | PITX2 | Controls cell proliferation in a tissue-specific manner / morphogenesis | TRAF1 | 400,7 | [3, 7, 30] |
|  | PRKAB2 | Subunit of AMPK / response to cellular metabolic stress | TRAF1/2 | 5,8 | [3, 4, 7, 16] |
|  | PSME4 | Cytokine signaling / TNFR2 non-canonical NF-κB pathway / signaling by the B cell receptor (BCR) | TNFR2  mediated | 3,5 | [2] |
|  | RAD23A | Proteasomal degradation | TRAF2/5/6 | 4,4 | [3, 4, 7, 22, 31] |
|  | RNF128 | Inhibitor of cytokine gene transcription | CD40L | 1257,1 | [7, 32] |
|  | RNF144B | Protection of cell death | TRAF3/4 | 6,4 | [3, 7, 30] |
|  | RORC | Signaling by interleukins | TRAF5 | 67,2 | [7, 33] |
|  | SLC30A2 | Cellular zinc homeostasis | CD40 | 261,7 | [3, 7] |
|  | SMN2 | Splicing of cellular pre-mRNAs | CD40 | 2,8 | [7, 18] |
|  | SMYD2 | Fibrin clot formation / hemostasis | TRAF2 | 3,4 | [2, 34] |
|  | SNRNP25 | Splicing of cellular pre-mRNAs | TRAF1/2 | 4,4 | [3, 4, 7] |
|  | STAU2 | RNA-binding and transport protein | TRAF2 | 3,4 | [7, 10] |
|  | STRADB | Pseudokinase regulates energy-generating metabolism | TRAF6 | 8,0 | [7, 35] |
|  | TENM3 | Neuronal development | TRAF2 | 21,4 | [7, 10] |
|  | TIGD4 | Exact function is not known | TRAF2 | 21,7 | [3, 7] |
|  | TOMM70 | Import receptor of the outer mitochondrial membrane | TRAF6 | 2,5 | [7, 36] |
|  | TRPT1 | Involved in tRNA splicing | TRAF2 | 5,5 | [3, 7, 37] |
|  | UBE2D1 | Regulation of TNFR1 signaling / adaptive immune system / cytokine signaling | TRAF2/6/7 | 3,9 | [2, 38, 39] |
|  | UBE2D4 | Adaptive immune system | TRAF6/7 | 4,0 | [2, 38] |
|  | UCHL1 | Processing of ubiquitin precursors | TRAF3/4/6 | 3,5 | [7, 40, 41] |
|  | UGP2 | Enzyme important in carbohydrate interconversions | TRAF3 | 3,9 | [7, 15] |
|  | USP2 | TNFR1 induced pro-apoptotic signaling / NF-κB mediated | TRAF1/2/6 | 12,2 | [2-4, 42, 43] |
|  | VDAC1 | Channel in outer mitochondrial membrane involved in cell volume regulation and apoptosis | TRAF6 | 7,4 | [7, 44] |
|  | WWP1 | Adaptive immune system / antigen processing | TRAF4/6 | 4,9 | [2, 4, 45] |
|  | XPO4 | Mediates the nuclear export of proteins | CD40 | 21,9 | [7, 18] |
|  | ZIC1 | Acts as a transcriptional activator | TRAF1 | 91,3 | [3, 7] |
| **Immune system** | AGL | Innate immune system | - | 11,8 | [2] |
|  | AMPD3 | Innate immune system | - | 38,3 | [2] |
|  | AP1S2 | MHC class II antigen presentation | - | 3,3 | [2] |
|  | ART1 | Innate immune system | - | 2158,6 | [2] |
|  | ASB10 | Adaptive immune system | - | 9945,9 | [2] |
|  | ASB11 | Adaptive immune system | - | 966,0 | [2] |
|  | ASB12 | Adaptive immune system | - | 23,9 | [2] |
|  | ASB14 | Adaptive immune system | - | 29,2 | [2] |
|  | ASB16 | Adaptive immune system | - | 13,6 | [2] |
|  | ASB18 | Adaptive immune system | - | 568,0 | [2] |
|  | ASB4 | Adaptive immune system | - | 1081,2 | [2] |
|  | ASB5 | Adaptive immune system | - | 1014,5 | [2] |
|  | ASB8 | Adaptive immune system | - | 6,6 | [2] |
|  | ATP8A1 | Innate immune system | - | 3,8 | [2] |
|  | BTBD1 | Adaptive immune system | - | 7,1 | [2] |
|  | C19orf47 | Adaptive immune system | - | 4,2 | [2] |
|  | CAMK2A | Cardiac conduction / interferon gamma signaling | - | 246,7 | [2] |
|  | CAMK2B | Cardiac conduction / interferon gamma signaling | - | 79,1 | [2] |
|  | CAP2 | MHC class II antigen presentation / adaptive immune system / hemostasis | - | 6,5 | [2] |
|  | CCL5 | Chemokine receptor / cytokine signaling | - | 8,2 | [2] |
|  | CCR3 | Chemokine receptor | - | 683,3 | [2] |
|  | CD300E | Adaptive immune system | - | 32,6 | [2] |
|  | COX10 | HEME biosynthesis | - | 5,8 | [2] |
|  | CX3CR1 | Chemokine receptor | - | 30,1 | [2] |
|  | CYSTM1 | Innate immune system | - | 3,0 | [2] |
|  | DNASE1L1 | Innate immune system | - | 5,8 | [2] |
|  | EIF4E3 | Interferon signaling | - | 2,5 | [2] |
|  | FBXO40 | Adaptive immune system | - | 595,3 | [2] |
|  | FCGR3B | Neutrophil degranulation | - | 16,2 | [2] |
|  | GBP6 | Interferon-γ signaling | - | 26,3 | [2] |
|  | GHR | Cytokine signaling | - | 3,0 | [2] |
|  | GYG1 | Innate immune system | - | 4,8 | [2] |
|  | GZMH | Metabolism of Angiotensinogen to Angiotensins / IL1 processing / intrinsic pathway for apoptosis / cell recruitment / interleukin singling | - | 37,6 | [2] |
|  | HLA-DQA2 | MHC class II antigen presentation / interferon-γ signaling / adaptive immune system | - | 13,5 | [2] |
|  | IL12RB2 | Cytokine signaling | - | 11,5 | [2] |
|  | IL20RA | Cytokine signaling | - | 6,0 | [2] |
|  | IL32 | Cytokine signaling | - | 5,0 | [2] |
|  | KBTBD13 | Adaptive immune system | - | 11,8 | [2] |
|  | KLHL41 | Adaptive immune system | - | 5742,1 | [2] |
|  | KPNA3 | Interferon signaling | - | 3,7 | [2] |
|  | LBP | Cytokine signaling | - | 45,9 | [2] |
|  | MBP | Innate immune system | - | 6,5 | [2] |
|  | NCAM1 | Interferon-γ signaling | - | 6,6 | [2] |
|  | NCKAP1L | Innate immune system | - | 8,1 | [2] |
|  | NFAM1 | Innate immune system | - | 15,4 | [2] |
|  | OSBPL11 | MHC class II antigen presentation / adaptive immune system | - | 3,3 | [2] |
|  | PADI2 | Innate immune system | - | 317,1 | [2] |
|  | PGM1 | Innate immune system | - | 7,2 | [2] |
|  | POMC | Cytokine signaling | - | 18,4 | [2] |
|  | PPP1R14C | Cytokine signaling | - | 82,4 | [2] |
|  | PPP1R1A | Cytokine signaling | - | 11,5 | [2] |
|  | PRKN | Adaptive immune system | - | 5,7 | [2] |
|  | PTGES2 | Innate immune system | - | 3,0 | [2] |
|  | PTPN20 | Cytokine signaling | - | 5,3 | [2] |
|  | RILP | MHC class II antigen presentation / adaptive immune system | - | 4,5 | [2] |
|  | RNF123 | Adaptive immune system | - | 6,6 | [2] |
|  | RNF34 | Adaptive immune system | - | 3,2 | [2] |
|  | S100A1 | Disease of immune system / adaptive immune system | - | 433,6 | [2] |
|  | S100P | Disease of immune system / adaptive immune system | - | 29,7 | [2] |
|  | SAR1B | MHC class II antigen presentation / adaptive immune system | - | 3,0 | [2] |
|  | SCN2B | Cardiac conduction / adaptive immune system / hemostasis / cell surface interactions at the vascular wall | - | 14,7 | [2] |
|  | SLC2A5 | Innate immune system | - | 64,4 | [2] |
|  | STATH | Innate immune system | - | 425,6 | [2] |
|  | SYNGR1 | Innate immune system | - | 3,7 | [2] |
|  | TOM1 | Adaptive immune system | - | 3,2 | [2] |
|  | TRIM63 | Adaptive immune system | - | 6798,3 | [2] |
|  | TUBA8 | MHC class II antigen presentation/ adaptive immune system / hemostasis | - | 29,5 | [2] |
|  | TUBB1 | MHC class II antigen presentation / adaptive immune system / hemostasis | - | 36,6 | [2] |
|  | UBE2G1 | Adaptive immune system | - | 4,0 | [2] |
|  | WIPF3 | Innate immune system | - | 7,2 | [2] |
| **Hemostasis** | ACTN2 | Platelet degranulation | - | 59,2 | [2] |
|  | AK3 | Factors involved in megakaryocyte development and platelet production | - | 2,7 | [2] |
|  | ALDOA | Platelet degranulation | - | 8,6 | [2] |
|  | APOA2 | Plasma protein remodeling | - | 434,3 | [2] |
|  | ATP2A1 | Cardiac conduction / platelet homeostasis | - | 3051,0 | [2] |
|  | ATP2A2 | Cardiac conduction / platelet homeostasis | - | 14,1 | [2] |
|  | ATP2B2 | Cardiac conduction / platelet homeostasis | - | 13,0 | [2] |
|  | C1QBP | Fibrin clot formation / intrinsic pathway for apoptosis | - | 2,8 | [2] |
|  | CA2 | O_2_/CO_2_ exchange erythrocytes | - | 41,3 | [2] |
|  | CYB5R1 | Platelet degranulation / O_2_/CO_2_ exchange in erythrocytes | - | 10,5 | [2] |
|  | EGF | Platelet degranulation | - | 369,5 | [2] |
|  | F2 | Fibrin clot formation / platelet aggregation / cell surface interactions at the vascular wall | - | 17,3 | [2] |
|  | FABP1 | HEME degradation | - | 2244,1 | [2] |
|  | FECH | Heme biogenesis | - | 3,9 | [2] |
|  | FGB | Fibrin clot formation / platelet degranulation / disease of immune system / platelet aggregation | - | 1707,8 | [2] |
|  | HS3ST5 | Heparin metabolism | - | 125,4 | [2] |
|  | HS6ST2 | Heparin metabolism | - | 773,4 | [2] |
|  | ITGA2B | Platelet degranulation / platelet aggregation | - | 11,1 | [2] |
|  | KIF1B | Factors involved in megakaryocyte development and platelet production | - | 4,8 | [2] |
|  | MFN2 | Factors involved in megakaryocyte development and platelet production | - | 4,9 | [2] |
|  | NFE2 | Factors involved in megakaryocyte development and platelet production | - | 23,9 | [2] |
|  | NOS1 | Platelet homeostasis | - | 114,8 | [2] |
|  | ORAI1 | Cardiac conduction / platelet homeostasis / signaling by B cell receptor | - | 7,9 | [2] |
|  | P2RX5 | Platelet homeostasis | - | 384,0 | [2] |
|  | P2RX6 | Platelet homeostasis | - | 53,5 | [2] |
|  | PDPN | Platelet activation, signaling and aggregation | - | 10,3 | [2] |
|  | PF4 | Fibrin clot formation /platelet degranulation / chemokine receptor / cell surface interactions at the vascular wall | - | 61,7 | [2] |
|  | PLEK | Platelet degranulation | - | 9,1 | [2] |
|  | PPBP | Fibrin clot formation / platelet degranulation / chemokine receptor | - | 67,5 | [2] |
|  | PRKCQ | Platelet activation, signaling and aggregation | - | 1070,1 | [2] |
|  | SERPINA5 | Fibrin clot formation | - | 10,5 | [2] |
|  | SERPINC1 | Fibrin clot formation | - | 25,0 | [2] |
|  | SH2B2 | Factors involved in megakaryocyte development and platelet production | - | 10,4 | [2] |
|  | SLC8A3 | Cardiac conduction / platelet homeostasis | - | 78,3 | [2] |
|  | TTN | Platelet degradation |  | 3945,9 | [2] |
|  | TUBA4A | Platelet degranulation / MHCII class antigen presentation / adaptive immune system | - | 4,1 | [2] |
|  | UROS | Heme biosynthesis | - | 2,7 | [2] |
| **Muscle contraction** | ACTA1 | Striated muscle contraction | - | 1721,1 | [2] |
|  | ATP1A2 | Cardiac conduction | - | 5,2 | [2] |
|  | CACNB1 | Cardiac conduction | - | 5,4 | [2] |
|  | CACNG6 | Cardiac conduction | - | 3484,6 | [2] |
|  | CASQ1 | Cardiac conduction | - | 389,5 | [2] |
|  | CAV3 | Smooth muscle contraction | - | 40,6 | [2] |
|  | DES | Striated muscle contraction | - | 20,0 | [2] |
|  | FXYD4 | Cardiac conduction | - | 326,6 | [2] |
|  | KCNE5 | Cardiac conduction | - | 14,6 | [2] |
|  | KCNJ11 | Cardiac conduction | - | 27,3 | [2] |
|  | KCNJ12 | Cardiac conduction | - | 10,4 | [2] |
|  | MME | Cardiac conduction | - | 5,3 | [2] |
|  | MYBPC1 | Striated muscle contraction | - | 54789,0 | [2] |
|  | MYBPC2 | Striated muscle contraction | - | 26958,7 | [2] |
|  | MYBPC3 | Striated muscle contraction | - | 23,7 | [2] |
|  | MYH3 | Striated muscle contraction | - | 19,1 | [2] |
|  | MYH6 | Striated muscle contraction | - | 25236,7 | [2] |
|  | MYH8 | Striated muscle contraction | - | 469,5 | [2] |
|  | MYL1 | Striated muscle contraction | - | 343322,1 | [2] |
|  | MYL12A | Smooth muscle contraction | - | 8,4 | [2] |
|  | MYL2 | Striated muscle contraction | - | 327779,2 | [2] |
|  | MYL3 | Striated muscle contraction | - | 1088,6 | [2] |
|  | MYL6B | Smooth muscle contraction | - | 111,3 | [2] |
|  | MYLPF | Smooth muscle contraction | - | 14218,1 | [2] |
|  | NEB | Striated muscle contraction | - | 16100,8 | [2] |
|  | NOS1 | Cardiac conduction | - | 114,8 | [2] |
|  | RYR1 | Cardiac conduction | - | 1391,7 | [2] |
|  | RYR3 | Cardiac conduction | - | 5,4 | [2] |
|  | SCN1B | Cardiac conduction | - | 10,7 | [2] |
|  | SCN4A | Cardiac conduction | - | 20,5 | [2] |
|  | SCN7A | Cardiac conduction | - | 6,0 | [2] |
|  | SLN | Cardiac conduction | - | 1173,2 | [2] |
|  | TCAP | Striated muscle contraction | - | 1018,5 | [2] |
|  | TMOD4 | Striated muscle contraction | - | 897,1 | [2] |
|  | TNNC1 | Cardiac conduction | - | 1125,7 | [2] |
|  | TNNC2 | Striated muscle contraction | - | 784,5 | [2] |
|  | TNNI1 | Striated muscle contraction | - | 68953,8 | [2] |
|  | TNNI2 | Striated muscle contraction | - | 10600,3 | [2] |
|  | TNNI3 | Cardiac conduction | - | 7689,8 | [2] |
|  | TNNT1 | Striated muscle contraction | - | 80791,8 | [2] |
|  | TNNT2 | Striated muscle contraction | - | 39,1 | [2] |
|  | TNNT3 | Striated muscle contraction | - | 168,3 | [2] |
|  | TPM3 | Smooth muscle contraction | - | 82,7 | [2] |
|  | TRDN | Cardiac conduction | - | 571,0 | [2] |
|  | TRIM72 | Smooth muscle contraction | - | 1193,2 | [2] |
| **Metabolism of Lipids** | ACADS | Fatty acid metabolism | - | 8,8 | [2] |
| ACBD5 | Fatty acid metabolism | - | 2,6 | [2] |  |
| ACHE | Phospholipid metabolism | - | 56,1 | [2] |  |
| ACOT11 | Fatty acid metabolism | - | 3,3 | [2] |  |
| ACSL1 | Fatty acid metabolism | - | 8,8 | [2] |  |
| ACSL6 | Fatty acid metabolism | - | 6,8 | [2] |  |
| AGPAT3 | Phospholipid metabolism | - | 2,6 | [2] |  |
| ALOX5AP | Fatty acid metabolism | - | 7,3 | [2] |  |
| AMACR | Fatty acid metabolism | - | 3,8 | [2] |  |
| CES3 | LDL clearance / plasma lipoprotein clearance | - | 12,3 | [2] |  |
| CPT1B | Fatty acid metabolism | - | 39,2 | [2] |  |
| CPT2 | Fatty acid metabolism | - | 5,1 | [2] |  |
| CRAT | Fatty acid metabolism | - | 5,6 | [2] |  |
| CYP2J2 | Fatty acid metabolism | - | 163,8 | [2] |  |
| DECR1 | Fatty acid metabolism | - | 3,7 | [2] |  |
| GNPAT | Phospholipid metabolism | - | 3,4 | [2] |  |
| GPAT3 | Phospholipid metabolism | - | 5,4 | [2] |  |
| GPD1 | Phospholipid metabolism | - | 9,5 | [2] |  |
| GPD1L | Phospholipid metabolism | - | 4,2 | [2] |  |
| HADH | Phospholipid metabolism | - | 3,8 | [2] |  |
| HADHA | Phospholipid metabolism | - | 3,5 | [2] |  |
| HADHB | Phospholipid metabolism | - | 12,1 | [2] |  |
| HPGD | Fatty acid metabolism | - | 20,4 | [2] |  |
| HTD2 | Fatty acid metabolism | - | 4,5 | [2] |  |
| LPIN1 | Phospholipid metabolism | - | 2,7 | [2] |  |
| LPL | Plasma protein remodeling | - | 5,8 | [2] |  |
| MCEE | Fatty acid metabolism | - | 3,7 | [2] |  |
| MLYCD | Fatty acid metabolism | - | 3,5 | [2] |  |
| NDUFAB1 | Fatty acid metabolism | - | 4,2 | [2] |  |
| PCCB | Fatty acid metabolism | - | 3,2 | [2] |  |
| PHOSPHO1 | Phospholipid metabolism | - | 30,7 | [2] |  |
| PHYH | Fatty acid metabolism | - | 7,7 | [2] |  |
| PLA2G4C | Phospholipid metabolism | - | 4,0 | [2] |  |
| PLA2G4F | Phospholipid metabolism | - | 1228,7 | [2] |  |
| PLAAT1 | Phospholipid metabolism | - | 78,4 | [2] |  |
| PON3 | Fatty acid metabolism | - | 224,9 | [2] |  |
| PRKAA2 | Fatty acid metabolism | - | 10,5 | [2] |  |
| PTGES3L | Fatty acid metabolism | - | 10,1 | [2] |  |
| PTGR2 | Fatty acid metabolism | - | 10,6 | [2] |  |
| SLC25A20 | Fatty acid metabolism | - | 3,3 | [2] |  |
| SLC27A2 | Fatty acid metabolism | - | 23,1 | [2] |  |
| TECRL | Fatty acid metabolism | - | 34786,4 | [2] |  |
| **Developmental Biology** | CDH15 | Myogenesis | - | 2086,6 | [2] |
|  | MAPK12 | Myogenesis / innate immune system | - | 19,0 | [2] |
|  | ME2 | Myogenesis | - | 2,7 | [2] |
|  | MYF5 | Myogenesis | - | 383,7 | [2] |
|  | MYF6 | Myogenesis | - | 30415,7 | [2] |
|  | MYOD1 | Myogenesis | - | 3466,0 | [2] |
|  | MYOG | Myogenesis | - | 11600,7 | [2] |
| **Apoptosis** | BID | Intrinsic pathway for apoptosis | - | 4,2 | [2] |
|  | DAPK2 | Caspase mediated apoptosis | - | 31,5 | [2] |
|  | TP63 | Intrinsic pathway for apoptosis | - | 32,2 | [2] |

|  | |  |  |  |  |
| --- | --- | --- | --- | --- | --- |
| **CHD+HT vs. CHD DOWN<0,5** | **Gene** | **Pathway/function** | **TRAF interaction** | **Log_2_ fold change** | **Ref.** |
| **CD40L-CD40-TRAF**  **signaling / interactors** | ADAMTS4 | Proteoglycan cleavage | TRAF2 | -10,4 | [7, 18] |
|  | ARSJ | Involved in hormone biosynthesis and modulation of cell signaling | TRAF1/2 | -5,5 | [4, 7] |
|  | BCL3 | Promotes transcription of NF-κB target genes, regulation of cell proliferation | TRAF6 | -3,3 | [7, 46] |
|  | CBLB | Regulation of immune system, promotes proteasome-mediated protein degradation | TRAF2/3/6 | -2,8 | [7, 47, 48] |
|  | CREB5 | Activates transcription | TRAF2 | -3,1 | [4, 7] |
|  | CRY1 | Regulates circadian clock, regulator of physiological functions | TRAF2 | -4,6 | [7, 37] |
|  | DDX39A | Export of mRNA out of the nucleus | TRAF3/6 | -2,8 | [7, 49, 50] |
|  | ETV6 | Involved in developing vascular network | TRAF2/3 | -3,0 | [7, 18, 51] |
|  | FOSL1 | IL-1 family signaling pathways | TRAF3 | -46,1 | [7, 52] |
|  | IL1RL1 | Interleukin signaling | TRAF6 | -55,6 | [2, 53] |
|  | KPNA2 | Immune system | TRAF1 | -4,0 | [2, 54] |
|  | PCDH10 | Calcium-dependent cell-adhesion protein | TRAF3 | -4,5 | [7, 18] |
|  | PDZD4 | Ubiquitin protein ligase activity | TRAF2/3 | -3,1 | [3, 7] |
|  | PHLDA1 | Regulation of apoptosis | TRAF4/6 | -7,1 | [3, 7, 55] |
|  | PPP1R18 | Actin and phosphatase binding | TRAF2 | -4,0 | [4, 7] |
|  | PPP2R1B | Interleukin signaling / TRAF6 mediated | TRAF6  mediated | -3,5 | [2] |
|  | RCAN1 | Nervous system development | TRAF6 | -5,6 | [7, 56] |
|  | SAA1 | Interleukin signaling / TRAF6 mediated | TRAF6 mediated | -5,1 | [2] |
|  | SLC30A7 | Zinc homeostasis | CD40 | -3,1 | [7, 44] |
|  | SPHK1 | TNFα signaling and NF-κB activation | TRAF2/6 | -4,5 | [7, 57-59] |
|  | SRC | Platelet activation, signaling and aggregation | TRAF1/3/6 | -2,9 | [2, 60-63] |
|  | TGFBR1 | Cell adhesion and regulation of apoptosis | TRAF4/6 | -2,9 | [7, 64-66] |
|  | TNFRSF10B | Death receptor, Apoptosis | TRAF2 | -2,8 | [2, 67, 68] |
|  | TNFRSF10D | Death receptor, Apoptosis | - | -5,6 | [2] |
|  | TNFRSF12A | Apoptosis | TRAF1/2 | -4,7 | [7, 69, 70] |
|  | TRAF4 | Immune system, NF-κB signaling | CD40 | -4,4 | [7, 71] |
| **Immune system** | ACKR4 | Chemokine Receptor | - | -7,9 | [2] |
|  | CCL2 | Chemokine Receptor / interleukin signaling | - | -7,2 | [2] |
|  | CCL8 | Chemokine Receptor | - | -8,2 | [2] |
|  | CXCL2 | Chemokine Receptor/interleukin signaling | - | -3,7 | [2] |
|  | CXCR5 | Chemokine Receptor | - | -4,6 | [2] |
|  | IL13RA2 | Interleukin signaling | - | -23,2 | [2] |
|  | IL17RE | Interleukin signaling | - | -3,0 | [2] |
|  | LIF | Interleukin signaling | - | -7,5 | [2] |
|  | MUC1 | Interleukin signaling | - | -3,8 | [2] |
|  | MX1 | Immune system | - | -2,8 | [2] |
|  | NGF | NF-κB activation and signals survival | - | -5,8 | [2] |
|  | NOS3 | eNos activation nitric oxide stimulates guanylate cyclase | - | -3,8 | [2] |
|  | OSMR | Cytokine signaling / interleukin signaling | - | -3,4 | [2] |
|  | PIM1 | Interleukin signaling | - | -6,2 | [2] |
| **Hemostasis** | CD177 | Fibril clot assembly | - | -29,3 | [2] |
|  | HPSE2 | Heparin metabolism | - | -5,2 | [2] |
|  | IDUA | Heparin metabolism | - | -3,3 | [2] |
|  | PCDH7 | Platelet activation, signaling and aggregation | - | -4,1 | [2] |
|  | PRKCG | Platelet activation, signaling and aggregation | - | -31,1 | [2] |
|  | SDC3 | Heparin metabolism | - | -3,1 | [2] |
|  | SLC35D2 | Heparin metabolism | - | -2,6 | [2] |
|  | TBXA2R | Thromboxane signaling / Platelet signaling, activation and aggregation | - | -3,6 | [2] |
|  | THBD | Fibril clot assembly | - | -4,4 | [2] |
|  | THBS1 | Platelet activation, signaling and aggregation | - | -5,4 | [2] |
| **Muscle contraction** | COL9A2 | Collagen fibril assembly | - | -4,7 | [2] |
|  | LOXL1 | Collagen fibril assembly | - | -3,3 | [2] |
|  | MMP14 | Collagen degradation | - | -3,3 | [2] |
| **Metabolism of lipids** | CYP1B1 | Fatty acid metabolism | - | -4,8 | [2] |
|  | FGFR2 | Insulin receptor signaling | - | -7,7 | [2] |
|  | LDLR | LDL clearance | - | -6,8 | [2] |
|  | MPP2 | Fatty acid metabolism | - | -3,3 | [2] |
|  | SOAT1 | LDL clearance | - | -2,8 | [2] |
|  | SYNJ2 | Phospholipid metabolism | - | -3,0 | [2] |
| **Apoptosis** | TP53BP2 | Death receptor ligand | - | -2,8 | [2] |

| **CHD+HT+T2DM vs. CHD UP>2** | **Gene** | **Pathway/function** | **TRAF interaction** | **Log_2_ fold change** | **Ref.** |
| --- | --- | --- | --- | --- | --- |
| **Immune system** | PADI2 | Immune system | - | 5,0 | [2] |
| **Hemostasis** | P2RX1 | Platelet homeostasis / immune system | - | 3,3 | [2] |
| **Apoptosis** | LINGO1 | Death receptor signaling | - | 2,7 | [2] |

| **CHD+HT+T2DM vs. CHD DOWN<0,5** | **Gene** | **Pathway/function** | **TRAF interaction** | **Log_2_ fold change** | **Ref.** |
| --- | --- | --- | --- | --- | --- |
| **TRAF interaction/**  **TRAF signaling** | FOSL1 | IL-1 family signaling pathways | TRAF3 | -4,8 | [7, 52] |
|  | NFATC2 | immune system downstream signal events of B cell receptor (BCR) | TRAF1/2/3  /6 | -2,9 | [2, 72] |
|  | RGCC | Regulate cell cycle progression | TRAF7 | -2,0 | [7, 18] |
|  | SAA1 | Cytokine signaling / TRAF6 mediated NF-κB activation | TRAF6 | -3,1 | [2] |
| **Immune system** | FGL2 | Neutrophil degranulation | - | -2,7 | [2] |
|  | HLA-DRB5 | Cytokine signaling | - | -3,2 | [2] |
|  | PIM1 | Cytokine signaling | - | -2,5 | [2] |

|  |  |  |  |  |  |
| --- | --- | --- | --- | --- | --- |
| **CHD+HT+T2DM**  **Vs. CHD+HT**  **UP > 2** | **Gene** | **Pathway/function** | **TRAF interaction** | **Log_2_ fold change** | **Ref.** |
| **TRAF interaction/**  **TRAF signaling** | ARSJ | Involved in hormone biosynthesis and modulation of cell signaling | TRAF1/2 | 3,0 | [4, 7] |
|  | GUCY1B1 | Mediates response to nitric oxide | TRAF3 | 1,9 | [7, 15] |
|  | HEY2 | Associated with aortic aneurysm / cardiovascular development | TRAF1/4 | 2,1 | [3, 4, 7] |
|  | ID1 | Transcriptional regulator / apoptosis / angiogenesis / cellular growth | TRAF6 | 2,1 | [7, 73] |
|  | PCDH10 | Calcium-dependent cell-adhesion protein | TRAF3 | 2,3 | [7, 18] |
|  | SORL1 | Stimulates proliferation and migration of monocytes/macrophages | TRAF1 | 2,3 | [7, 18] |
| **Immune system** | CYFIP2 | Innate immune system | - | 2,3 | [2] |
|  | IL17RE | Interleukin signaling | - | 1,9 | [2] |
|  | NFASC | Neutrophil degranulation | - | 2,6 | [2] |
| **Hemostasis** | ITIH3 | Platelet activation, signaling and aggregation | - | 2,5 | [2] |
|  | P2RX1 | Immune system / platelet homeostasis | - | 4,0 | [2] |
|  | TBXA2R | Platelet activation, signaling and aggregation | - | 2,2 | [2] |
| **Muscle contraction** | CACNA1H | Smooth muscle contraction | - | 2,7 | [2] |
|  | KCNE4 | Muscle contraction | - | 2,0 | [2] |
|  | KCNJ3 | Cardiac conduction | - | 2,2 | [2] |
|  | SLC8A1 | Cardiac conduction / muscle contraction / platelet homeostasis | - | 2,1 | [2] |
| **Apoptosis** | LINGO1 | Death receptor signaling | - | 3,9 | [2] |

|  |  |  |  |  |  |
| --- | --- | --- | --- | --- | --- |
| **CHD+HT+T2DM**  **vs. CHD+HT**  **down < 0,5** | **Gene** | **Pathway/function** | **TRAF interaction** | **Log_2_ fold change** | **Ref.** |
| **TRAF interaction/**  **TRAF signaling** | ABLIM1 | Innate immune system | TRAF2 | -2,5 | [2, 37] |
|  | ACAT1 | Synthesis of ketone bodies / LDL clearance | TRAF4 | -2,8 | [1, 2] |
|  | ACTN3 | (Skeletal) muscle contraction | TRAF1/2 | -11,2 | [2-4] |
|  | AKR1B15 | Metabolism of lipids | TRAF2 | -5,5 | [2, 3] |
|  | ATP1B1 | Cardiac conduction / cell surface interactions at the vascular wall | TRAF3/6 | -4,8 | [2, 8] |
|  | CECR2 | Chromatin remodeling / DNA damage response | TRAF7 | -6,0 | [7, 9] |
|  | CEP85 | Microtubule organizing center / involved in cell-cycle progression | TRAF2/4 | -4,6 | [3, 7, 10] |
|  | DEPTOR | Negative regulation of TOR signaling | TRAF1 | -4,2 | [4, 7] |
|  | DGCR6 | DiGeorge syndrome / neuronal crest cell migration | TRAF1/2 | -4,8 | [7, 16] |
|  | DUSP13 | Protein-tyrosine phosphatase / cell proliferation and differentiation | TRAF1 | -11,3 | [3, 7] |
|  | ECI2 | Peroxisomal lipid metabolism / fatty acid metabolism | TRAF1 | -2,0 | [7, 17, 18] |
|  | ECSIT | IL-1 receptor signaling pathway / TRAF6 mediated induction of NF-κb pathway | TRAF6 | -1,8 | [7, 19, 20] |
|  | FREM2 | Epidermal interactions | TRAF2 | -6,6 | [7, 10] |
|  | FXR1 | Associated with myopathy, RNA binding protein | TRAF2/7 | -2,5 | [7, 18, 21] |
|  | GP1BB | Fibrin clot formation / platelet adhesion to exposed collagen | TRAF4 | -12,1 | [2, 74] |
|  | KIAA1217 | Involved in skeletal system development | TRAF1/2 | -2,6 | [3, 4, 7] |
|  | KLHL38 | Associated with posterior myocardial infarction | TRAF1 | -3,8 | [4, 7] |
|  | KRT23 | Structural integrity of epithelial cells | TRAF7 | -7,4 | [7, 18] |
|  | KRT31 | Keratin gene family | TRAF4 | -7,7 | [3, 4, 7] |
|  | LRRN1 | Cell recruitment (pro-inflammatory response) | TRAF1 | -3,6 | [2, 3] |
|  | MAPT | Promotes microtubule assembly and stability | TRAF6 | -4,1 | [7, 25] |
|  | MYOZ1 | Cardiac and skeletal muscle / associated with myopathy | TRAF1 | -5,5 | [3, 7] |
|  | NEDD4L | Innate Immune system / autophagy | TRAF3 | -3,9 | [7, 26] |
|  | OTUD1 | TNFR1 induced pro-apoptotic signaling / NF-κB mediated | TNFR1  mediated | -2,4 | [2] |
|  | PDE4DIP | Microtubule assembly | TRAF4 | -4,2 | [1, 2] |
|  | PINK1 | Protect cells from stress-induced mitochondrial dysfunction | TRAF3/6 | -3,0 | [7, 27-29] |
|  | PITX2 | Controls cell proliferation in a tissue-specific manner / morphogenesis | TRAF1 | -8,4 | [3, 7, 30] |
|  | RGCC | Regulate cell cycle progression | TRAF7 | -2,7 | [7, 18] |
|  | RORC | Signaling by interleukins | TRAF5 | -7,9 | [7, 33] |
|  | TEAD4 | Transcription factor | TRAF1 | -3,0 | [3, 4, 7] |
|  | TMEM178B | Integral component of membrane | TRAF2 | -4,0 | [7, 10] |
|  | TRPT1 | Involved in tRNA splicing | TRAF2 | -2,3 | [3, 7, 37] |
|  | UBE2D1 | Regulation of TNFR1 signaling / adaptive immune system / cytokine signaling | TRAF2/6/7 | -2,2 | [2, 38, 39] |
|  | UCHL1 | Processing of ubiquitin precursors | TRAF3/4/6 | -2,1 | [7, 40, 41] |
|  | VDAC1 | Channel in outer mitochondrial membrane involved in cell volume regulation and apoptosis | TRAF6 | -2,8 | [7, 44] |
|  | WWP1 | Adaptive immune system / antigen processing | TRAF4/6 | -2,4 | [2, 4, 45] |
|  | XPO4 | Mediates the nuclear export of proteins | CD40 | -4,1 | [7, 18] |
|  | ZIC1 | Transcription factor | TRAF1 | -8,2 | [3, 7] |
| **Immune system** | AGL | Neutrophil degranulation | - | -3,4 | [2] |
|  | AMPD3 | Neutrophil degranulation | - | -5,1 | [2] |
|  | ART1Fart1 | Innate immune system | - | -11,3 | [2] |
|  | ASB10 | Antigen processing / Class I MHC mediated antigen processing and presentation | - | -7,4 | [2] |
|  | ASB11 | Antigen processing / Class I MHC mediated antigen processing and presentation | - | -8,7 | [2] |
|  | ASB12 | Antigen processing / Class I MHC mediated antigen processing and presentation | - | -4,8 | [2] |
|  | ASB14 | Antigen processing / class I MHC mediated antigen processing and presentation | - | -4,2 | [2] |
|  | ASB15 | Antigen processing / Class I MHC mediated antigen processing and presentation | - | -8,1 | [2] |
|  | ASB16 | Antigen processing / Class I MHC mediated antigen processing and presentation | - | -3,1 | [2] |
|  | ASB4 | Antigen processing / Class I MHC mediated antigen processing and presentation | - | -12,1 | [2] |
|  | ASB5 | Antigen processing / Class I MHC mediated antigen processing and presentation | - | -9,8 | [2] |
|  | ASB8 | Antigen processing / Class I MHC mediated antigen processing and presentation | - | -2,2 | [2] |
|  | ATP8A1 | Neutrophil degranulation | - | -2,2 | [2] |
|  | BTBD1 | Antigen processing | - | -2,8 | [2] |
|  | C1QC | Innate immune system | - | -2,9 | [2] |
|  | CNTFR | Immune system , IL6 family signaling | - | -2,1 | [2] |
|  | CXCL9 | Chemokine receptor binds chemokine | - | -3,8 | [2] |
|  | CYBA | Class I MHC mediated antigen processing and presentation / neutrophil degranulation | - | -2,3 | [2] |
|  | DNASE1L1 | Neutrophil degranulation | - | -2,9 | [2] |
|  | FBXO2 | Antigen processing / class I MHC mediated antigen processing and presentation | - | -3,1 | [2] |
|  | FBXO40 | Antigen processing / Class I MHC mediated antigen processing and presentation | - | -9,3 | [2] |
|  | FCGR3B | Neutrophil degranulation | - | -4,8 | [2] |
|  | HLA-DQA2 | Interferon-γ signaling | - | -4,5 | [2] |
|  | IL20RA | Interleukin signaling | - | -3,8 | [2] |
|  | IL32 | Interleukin signaling | - | -3,4 | [2] |
|  | KBTBD13 | Antigen processing / Class I MHC mediated antigen processing and presentation | - | -2,7 | [2] |
|  | KLHL41 | Antigen processing / Class I MHC mediated antigen processing and presentation | - | -10,8 | [2] |
|  | LBP | Innate immune system / signaling by interleukins | - | -5,9 | [2] |
|  | LRRC14B | Neutrophil degranulation | - | -14,1 | [2] |
|  | MAPKAPK3 | Signaling by interleukins | - | -2,6 | [2] |
|  | MBP | Neutrophil degranulation | - | -2,4 | [2] |
|  | MPO | Neutrophil degranulation | - | -6,2 | [2] |
|  | MYH2 | Innate immune system | - | -10,2 | [2] |
|  | NCAM1 | Interferon-γ signaling | - | -4,6 | [2] |
|  | PGM1 | Neutrophil degranulation | - | -2,9 | [2] |
|  | PPP1R14C | Interleukin signaling / phospholipid metabolism | - | -4,9 | [2] |
|  | PYGM | Neutrophil degranulation | - | -5,6 | [2] |
|  | RNF123 | Antigen processing / class I MHC mediated antigen processing and presentation | - | -2,6 | [2] |
|  | RNF144B | Antigen processing / class I MHC mediated antigen processing and presentation | - | -3,0 | [2] |
|  | S100A1 | Class I MHC mediated antigen processing and presentation | - | -8,0 | [2] |
|  | S100A9 | Class I MHC mediated antigen processing and presentation / neutrophil degranulation | - | -4,1 | [2] |
|  | SLC16A3 | Cell surface interactions at the vascular wall | - | -2,3 | [2] |
|  | SLC2A5 | Neutrophil degranulation | - | -6,3 | [2] |
|  | SYNGR1 | Neutrophil degranulation | - | -3,0 | [2] |
|  | TOM1 | Antigen processing / Class I MHC mediated antigen processing and presentation / neutrophil degranulation | - | -2,0 | [2] |
|  | TUBA8 | MHC class II antigen presentation | - | -3,3 | [2] |
|  | TYROBP | Neutrophil degranulation | - | -3,3 | [2] |
|  | UBAC1 | antigen processing / Class I MHC mediated antigen processing and presentation | - | -2,2 | [2] |
| **Hemostasis** | CA2 | Erythrocytes take up carbon dioxide and release oxygen | - | -6,1 | [2] |
|  | CYB5R1 | Platelet degranulation | - | -3,3 | [2] |
|  | EGF | Platelet degranulation | - | -6,0 | [2] |
|  | FCER1G | Platelet adhesion to exposed collagen / neutrophil degranulation / cell surface interactions at the vascular wall | - | -3,7 | [2] |
|  | FGB | Common pathway of fibrin clot formation / Class I MHC mediated antigen processing / plug formation, platelet degranulation and presentation | - | -11,0 | [2] |
|  | HS6ST2 | Heparan sulfate / heparin metabolism | - | -5,3 | [2] |
|  | ITGA2B | Platelet degranulation / platelet aggregation plug formation | - | -4,7 | [2] |
|  | P2RX5 | Platelet calcium homeostasis / platelet homeostasis | - | -7,0 | [2] |
|  | P2RX6 | Platelet calcium homeostasis / platelet homeostasis | - | -5,2 | [2] |
|  | PF4 | Common pathway of fibrin clot formation / platelet degranulation / chemokine receptor binds chemokine / cell surface interactions at the vascular wall | - | -5,1 | [2] |
|  | PLEK | Platelet degranulation | - | -3,7 | [2] |
|  | PPBP | Common pathway of fibrin clot formation / platelet degranulation / chemokine receptor binds chemokine / cell surface interactions at the vascular wall | - | -4,6 | [2] |
|  | SERPINA5 | Common pathway of fibrin clot formation / neutrophil degranulation | - | -4,3 | [2] |
|  | TRIM63 | Antigen processing / Class I MHC mediated antigen processing and presentation | - | -14,8 | [2] |
|  | TTR | Neutrophil degranulation | - | -7,8 | [2] |
| **Muscle contraction** | ACTA1 | Striated muscle contraction / utilization of ketone bodies / ketone body metabolism | - | -8,8 | [2] |
|  | ACTN2 | Striated muscle contraction / platelet degranulation | - | -6,7 | [2] |
|  | ATP2A1 | Cardiac conduction / platelet calcium homeostasis / platelet homeostasis | - | -10,5 | [2] |
|  | ATP2A2 | Cardiac conduction / platelet calcium homeostasis / platelet homeostasis | - | -3,8 | [2] |
|  | ATP2B2 | Cardiac conduction / platelet calcium homeostasis / platelet homeostasis | - | -5,7 | [2] |
|  | CACNB1 | Cardiac conduction | - | -2,7 | [2] |
|  | CACNG6 | Cardiac conduction | - | -8,9 | [2] |
|  | CAMK2A | Cardiac conduction / interferon-γ signaling | - | -6,8 | [2] |
|  | CAMK2B | Cardiac conduction / interferon-γ signaling | - | -7,6 | [2] |
|  | CASQ1 | Cardiac conduction | - | -9,2 | [2] |
|  | CAV3 | Smooth muscle contraction | - | -3,7 | [2] |
|  | KCNE5 | Cardiac conduction | - | -5,5 | [2] |
|  | KCNJ11 | Cardiac conduction / regulation of insulin secretion | - | -4,6 | [2] |
|  | KCNJ12 | Cardiac conduction | - | -4,4 | [2] |
|  | MME | Cardiac conduction / metabolism of angiotensinogen to angiotensin’s | - | -3,2 | [2] |
|  | MME | Cardiac conduction / metabolism of angiotensinogen to angiotensin’s | - | -3,2 | [2] |
|  | MYBPC1 | Striated muscle contraction | - | -11,9 | [2] |
|  | MYBPC2 | Striated muscle contraction | - | -9,6 | [2] |
|  | MYH3 | Striated muscle contraction | - | -4,4 | [2] |
|  | MYH6 | Striated muscle contraction | - | -14,9 | [2] |
|  | MYL1 | Striated muscle contraction | - | -9,5 | [2] |
|  | MYL12A | Smooth muscle contraction | - | -3,1 | [2] |
|  | MYL2 | Striated muscle contraction | - | -14,4 | [2] |
|  | MYL3 | Striated muscle contraction | - | -9,2 | [2] |
|  | MYL6B | Smooth muscle contraction | - | -7,3 | [2] |
|  | MYLPF | Smooth muscle contraction | - | -8,1 | [2] |
|  | NEB | Striated muscle contraction | - | -11,1 | [2] |
|  | NOS1 | Cardiac conduction / platelet homeostasis / innate immune system | - | -6,6 | [2] |
|  | ORAI1 | Cardiac conduction / platelet calcium homeostasis / platelet homeostasis / signaling by the b cell receptor (BCR) | - | -3,2 | [2] |
|  | RYR1 | Cardiac conduction | - | -9,8 | [2] |
|  | RYR3 | Cardiac conduction | - | -2,8 | [2] |
|  | SCN1B | Cardiac conduction | - | -4,6 | [2] |
|  | SCN2B | Cardiac conduction / cell surface interactions at the vascular wall | - | -5,3 | [2] |
|  | SCN3B | Cardiac conduction | - | -4,4 | [2] |
|  | SCN4A | Cardiac conduction | - | -5,6 | [2] |
|  | SLC8A3 | Cardiac conduction/ platelet calcium homeostasis / platelet homeostasis | - | -7,8 | [2] |
|  | SLN | Cardiac conduction | - | -9,9 | [2] |
|  | TCAP | Striated muscle contraction | - | -10,6 | [2] |
|  | TMOD4 | Striated muscle contraction | - | -9,4 | [2] |
|  | TNNC1 | Striated muscle contraction / cardiac conduction | - | -10,3 | [2] |
|  | TNNC2 | Striated muscle contraction | - | -7,8 | [2] |
|  | TNNI1 | Striated muscle contraction | - | -14,8 | [2] |
|  | TNNI2 | Striated muscle contraction | - | -7,8 | [2] |
|  | TNNI3 | Striated muscle contraction / cardiac conduction | - | -13,2 | [2] |
|  | TNNT1 | Striated muscle contraction | - | -13,9 | [2] |
|  | TNNT3 | Striated muscle contraction | - | -6,9 | [2] |
|  | TPM3 | Striated muscle contraction / smooth | - | -7,1 | [2] |
|  | TRDN | Cardiac conduction | - | -6,2 | [2] |
|  | TRIM72 | Smooth muscle contraction | - | -8,6 | [2] |
|  | TTN | Striated muscle contraction / platelet degranulation | - | -10,9 | [2] |
| **Myogenesis** | CDH15 | Myogenesis | - | -7,5 | [2] |
|  | MAPK12 | Myogenesis / innate immune system | - | -4,9 | [2] |
|  | MYF5 | Myogenesis | - | -7,0 | [2] |
|  | MYF6 | Myogenesis | - | -8,3 | [2] |
|  | MYOD1 | Myogenesis | - | -8,9 | [2] |
|  | MYOG | Myogenesis | - | -6,7 | [2] |
| **Apoptosis** | ARHGEF4 | Cell death signaling | - | -2,5 | [2] |
|  | DAPK2 | Caspase activation apoptosis | - | -5,3 | [2] |
|  | OBSCN | Cell death signaling | - | -7,6 | [2] |
|  | PRKCQ | Apoptotic cleavage of cellular proteins / platelet activation, signaling and aggregation | - | -8,5 | [2] |
| **Metabolism of lipids** | TP63 | Apoptosis | - | -4,1 | [2] |
|  | ACAA2 | Fatty acid metabolism | - | -2,5 | [2] |
|  | ACADM | regulation of lipid metabolism by PPARα / fatty acid metabolism | - | -2,6 | [2] |
|  | ACADS | fatty acid metabolism | - | -2,6 | [2] |
|  | ACHE | Phospholipid metabolism |  | -6,8 | [2] |
|  | ACSL1 | Regulation of lipid metabolism by PPARα / fatty acid metabolism | - | -3,0 | [2] |
|  | ACSL6 | Fatty acid metabolism | - | -3,7 | [2] |
|  | ACYP2 | Fatty acid metabolism | - | -3,1 | [2] |
|  | AKR1B1 | metabolism of lipids | - | -3,0 | [2] |
|  | ANKRD1 | Regulation of lipid metabolism by PPARα | -- | -7,9 | [2] |
|  | BDH1 | Synthesis of ketone bodies / utilization of ketone bodies / ketone body metabolism | - | -4,7 | [2] |
|  | CERS1 | Sphingolipid metabolism | - | -3,5 | [2] |
|  | CES3 | LDL clearance | - | -4,8 | [2] |
|  | CPT1B | Fatty acid metabolism | - | -6,1 | [2] |
|  | CRAT | Peroximal lipid metabolism / fatty acid metabolism | - | -2,7 | [2] |
|  | CYP2J2 | Metabolism of lipids | - | -6,9 | [2] |
|  | DECR1 | Fatty acid metabolism | - | -2,0 | [2] |
|  | ESRRA | Regulation of lipid metabolism by PPARα | - | -3,1 | [2] |
|  | FABP7 | Triglycerin metabolism | - | -11,0 | [2] |
|  | FITM1 | Lipid particle organization | - | -9,0 | [2] |
|  | GNA15 | Free fatty acids regulates insulin secretion / platelet activation, signaling and aggregation / regulation of insulin secretion | - | -4,3 | [2] |
|  | GPAT3 | Phospholipid metabolism | - | -4,0 | [2] |
|  | HADHB | Fatty acid metabolism / phospholipid metabolism | - | -3,2 | [2] |
|  | HMGCS2 | Synthesis of ketone bodies / ketone body metabolism / regulation of lipid metabolism by PPARα | - | -9,7 | [2] |
|  | IDI2 | Metabolism of lipids | - | -14,6 | [2] |
|  | KCNS3 | Regulation of insulin secretion | - | -2,1 | [2] |
|  | PHOSPHO1 | Phospholipid metabolism | - | -4,3 | [2] |
|  | PHYH | Peroximal lipid metabolism / fatty acid metabolism | - | -2,6 | [2] |
|  | PLA2G4F | Hydrolysis of LPC / phospholipid metabolism | - | -7,4 | [2] |
|  | PLAAT1 | Phospholipid metabolism | - | -7,0 | [2] |
|  | PLIN2 | Lipophagy / regulation of lipid metabolism by PPARα | - | -2,4 | [2] |
|  | POMC | Metabolism of lipids / signaling by interleukins | - | -4,0 | [2] |
|  | PRKAA2 | Lipophagy / fatty acid metabolism | - | -3,0 | [2] |
|  | PRKAG3 | Lipophagy | - | -6,4 | [2] |
|  | PTGR2 | Fatty acid metabolism | - | -2,7 | [2] |
|  | STARD10 | Phospholipid metabolism | - | -1,8 | [2] |
|  | TECRL | Fatty acid metabolism | - | -15,4 | [2] |

**Suppl. Table S1:** The following databases were used for the IPA analysis: 1. BioGRID [75] for CD40L-CD40-TRAF interaction analysis, 2. Reactome [76] for pathway analysis, and 3. GeneCards [7] for gene function analysis.

1. Rozan, L.M. and W.S. El-Deiry, *Identification and characterization of proteins interacting with Traf4, an enigmatic p53 target.* Cancer Biol Ther, 2006. **5**(9): p. 1228-35.

2. Fabregat, A., et al., *Reactome diagram viewer: data structures and strategies to boost performance.* Bioinformatics (Oxford, England), 2018. **34**(7): p. 1208-1214.

3. Luck, K., et al., *A reference map of the human binary protein interactome.* Nature, 2020. **580**(7803): p. 402-408.

4. Rolland, T., et al., *A proteome-scale map of the human interactome network.* Cell, 2014. **159**(5): p. 1212-1226.

5. Raper, J., et al., *Characterization of a novel trypanosome lytic factor from human serum.* Infect Immun, 1999. **67**(4): p. 1910-6.

6. Antonicka, H., et al., *A High-Density Human Mitochondrial Proximity Interaction Network.* Cell Metab, 2020. **32**(3): p. 479-497.e9.

7. Stelzer, G., et al., *The GeneCards Suite: From Gene Data Mining to Disease Genome Sequence Analyses.* Current protocols in bioinformatics, 2016. **54**: p. 1.30.1–1.30.33.

8. Cao, W., et al., *Inducible ATP1B1 Upregulates Antiviral Innate Immune Responses by the Ubiquitination of TRAF3 and TRAF6.* J Immunol, 2021. **206**(11): p. 2668-2681.

9. Kim, J.J., et al., *Systematic bromodomain protein screens identify homologous recombination and R-loop suppression pathways involved in genome integrity.* Genes Dev, 2019. **33**(23-24): p. 1751-1774.

10. Van Quickelberghe, E., et al., *A protein-protein interaction map of the TNF-induced NF-κB signal transduction pathway.* Sci Data, 2018. **5**: p. 180289.

11. Snelling, T., et al., *Co-ordinated control of the ADP-heptose/ALPK1 signalling network by the E3 ligases TRAF6, TRAF2/c-IAP1 and LUBAC.* Biochem J, 2022. **479**(20): p. 2195-2216.

12. Rothbard, J.B., et al., *Chaperone activity of α B-crystallin is responsible for its incorrect assignment as an autoantigen in multiple sclerosis.* J Immunol, 2011. **186**(7): p. 4263-8.

13. Havugimana, P.C., et al., *Scalable multiplex co-fractionation/mass spectrometry platform for accelerated protein interactome discovery.* Nat Commun, 2022. **13**(1): p. 4043.

14. Zhu, Z., et al., *Cutting Edge: A Cullin-5-TRAF6 Interaction Promotes TRAF6 Polyubiquitination and Lipopolysaccharide Signaling.* J Immunol, 2016. **197**(1): p. 21-6.

15. Liu, Y., et al., *Mitochondrial Fission Factor Is a Novel Interacting Protein of the Critical B Cell Survival Regulator TRAF3 in B Lymphocytes.* Front Immunol, 2021. **12**: p. 670338.

16. Corominas, R., et al., *Protein interaction network of alternatively spliced isoforms from brain links genetic risk factors for autism.* Nat Commun, 2014. **5**: p. 3650.

17. Greenfeld, H., et al., *TRAF1 Coordinates Polyubiquitin Signaling to Enhance Epstein-Barr Virus LMP1-Mediated Growth and Survival Pathway Activation.* PLoS Pathog, 2015. **11**(5): p. e1004890.

18. Huttlin, E.L., et al., *Dual proteome-scale networks reveal cell-specific remodeling of the human interactome.* Cell, 2021. **184**(11): p. 3022-3040.e28.

19. Min, Y., et al., *Inhibition of TRAF6 ubiquitin-ligase activity by PRDX1 leads to inhibition of NFKB activation and autophagy activation.* Autophagy, 2018. **14**(8): p. 1347-1358.

20. Kim, M.J., et al., *p62 Negatively Regulates TLR4 Signaling via Functional Regulation of the TRAF6-ECSIT Complex.* Immune Netw, 2019. **19**(3): p. e16.

21. Sakai, Y., et al., *Protein interactome reveals converging molecular pathways among autism disorders.* Sci Transl Med, 2011. **3**(86): p. 86ra49.

22. Yachie, N., et al., *Pooled-matrix protein interaction screens using Barcode Fusion Genetics.* Mol Syst Biol, 2016. **12**(4): p. 863.

23. Yu, J., et al., *Interaction of Tumor Necrosis Factor Receptor-associated Factor 6 (TRAF6) and Vav3 in the Receptor Activator of Nuclear Factor κB (RANK) Signaling Complex Enhances Osteoclastogenesis.* J Biol Chem, 2016. **291**(39): p. 20643-60.

24. Lin, J., et al., *The scaffold protein RACK1 mediates the RANKL-dependent activation of p38 MAPK in osteoclast precursors.* Sci Signal, 2015. **8**(379): p. ra54.

25. Babu, J.R., T. Geetha, and M.W. Wooten, *Sequestosome 1/p62 shuttles polyubiquitinated tau for proteasomal degradation.* J Neurochem, 2005. **94**(1): p. 192-203.

26. Gao, P., et al., *E3 ligase Nedd4l promotes antiviral innate immunity by catalyzing K29-linked cysteine ubiquitination of TRAF3.* Nat Commun, 2021. **12**(1): p. 1194.

27. Zhou, J., et al., *Mitochondrial Protein PINK1 Positively Regulates RLR Signaling.* Front Immunol, 2019. **10**: p. 1069.

28. Murata, H., et al., *SARM1 and TRAF6 bind to and stabilize PINK1 on depolarized mitochondria.* Mol Biol Cell, 2013. **24**(18): p. 2772-84.

29. Lee, H.J., et al., *PINK1 stimulates interleukin-1β-mediated inflammatory signaling via the positive regulation of TRAF6 and TAK1.* Cell Mol Life Sci, 2012. **69**(19): p. 3301-15.

30. Zhang, Z., et al., *RNF144B inhibits LPS-induced inflammatory responses via binding TBK1.* J Leukoc Biol, 2019. **106**(6): p. 1303-1311.

31. Akhtar, N., A.K. Singh, and S. Ahmed, *MicroRNA-17 Suppresses TNF-α Signaling by Interfering with TRAF2 and cIAP2 Association in Rheumatoid Arthritis Synovial Fibroblasts.* J Immunol, 2016. **197**(6): p. 2219-28.

32. Lineberry, N.B., et al., *Cutting edge: The transmembrane E3 ligase GRAIL ubiquitinates the costimulatory molecule CD40 ligand during the induction of T cell anergy.* J Immunol, 2008. **181**(3): p. 1622-6.

33. Wang, X., et al., *TRAF5-mediated Lys-63-linked Polyubiquitination Plays an Essential Role in Positive Regulation of RORγt in Promoting IL-17A Expression.* J Biol Chem, 2015. **290**(48): p. 29086-94.

34. Wu, W., et al., *SMYD2-mediated TRAF2 methylation promotes the NF-κB signaling pathways in inflammatory diseases.* Clin Transl Med, 2021. **11**(11): p. e591.

35. Sanna, M.G., et al., *ILPIP, a novel anti-apoptotic protein that enhances XIAP-mediated activation of JNK1 and protection against apoptosis.* J Biol Chem, 2002. **277**(34): p. 30454-62.

36. Liu, X.Y., et al., *Tom70 mediates activation of interferon regulatory factor 3 on mitochondria.* Cell Res, 2010. **20**(9): p. 994-1011.

37. Rual, J.F., et al., *Towards a proteome-scale map of the human protein-protein interaction network.* Nature, 2005. **437**(7062): p. 1173-8.

38. Markson, G., et al., *Analysis of the human E2 ubiquitin conjugating enzyme protein interaction network.* Genome Res, 2009. **19**(10): p. 1905-11.

39. Pao, K.C., et al., *Activity-based E3 ligase profiling uncovers an E3 ligase with esterification activity.* Nature, 2018. **556**(7701): p. 381-385.

40. Karim, R., et al., *Human papillomavirus (HPV) upregulates the cellular deubiquitinase UCHL1 to suppress the keratinocyte's innate immune response.* PLoS Pathog, 2013. **9**(5): p. e1003384.

41. Zhao, Y., et al., *Hypermethylation of UCHL1 Promotes Metastasis of Nasopharyngeal Carcinoma by Suppressing Degradation of Cortactin (CTTN).* Cells, 2020. **9**(3).

42. Mahul-Mellier, A.L., et al., *De-ubiquitinating protease USP2a targets RIP1 and TRAF2 to mediate cell death by TNF.* Cell Death Differ, 2012. **19**(5): p. 891-9.

43. Li, Y., et al., *USP2a positively regulates TCR-induced NF-κB activation by bridging MALT1-TRAF6.* Protein Cell, 2013. **4**(1): p. 62-70.

44. Ewing, R.M., et al., *Large-scale mapping of human protein-protein interactions by mass spectrometry.* Mol Syst Biol, 2007. **3**: p. 89.

45. Lin, X.W., et al., *WW domain containing E3 ubiquitin protein ligase 1 (WWP1) negatively regulates TLR4-mediated TNF-α and IL-6 production by proteasomal degradation of TNF receptor associated factor 6 (TRAF6).* PLoS One, 2013. **8**(6): p. e67633.

46. Wang, K., et al., *BCL3 regulates RANKL-induced osteoclastogenesis by interacting with TRAF6 in bone marrow-derived macrophages.* Bone, 2018. **114**: p. 257-267.

47. Qiao, G., et al., *Negative regulation of CD40-mediated B cell responses by E3 ubiquitin ligase Casitas-B-lineage lymphoma protein-B.* J Immunol, 2007. **179**(7): p. 4473-9.

48. Xu, L., et al., *DR5-Cbl-b/c-Cbl-TRAF2 complex inhibits TRAIL-induced apoptosis by promoting TRAF2-mediated polyubiquitination of caspase-8 in gastric cancer cells.* Mol Oncol, 2017. **11**(12): p. 1733-1751.

49. Shi, P., et al., *SUMOylation of DDX39A Alters Binding and Export of Antiviral Transcripts to Control Innate Immunity.* J Immunol, 2020. **205**(1): p. 168-180.

50. Gu, L., et al., *DDX3 directly regulates TRAF3 ubiquitination and acts as a scaffold to co-ordinate assembly of signalling complexes downstream from MAVS.* Biochem J, 2017. **474**(4): p. 571-587.

51. Yang, X., et al., *Widespread Expansion of Protein Interaction Capabilities by Alternative Splicing.* Cell, 2016. **164**(4): p. 805-17.

52. Cai, B., et al., *FOSL1 Inhibits Type I Interferon Responses to Malaria and Viral Infections by Blocking TBK1 and TRAF3/TRIF Interactions.* mBio, 2017. **8**(1).

53. Schmitz, J., et al., *IL-33, an interleukin-1-like cytokine that signals via the IL-1 receptor-related protein ST2 and induces T helper type 2-associated cytokines.* Immunity, 2005. **23**(5): p. 479-90.

54. Wang, J., et al., *Toward an understanding of the protein interaction network of the human liver.* Mol Syst Biol, 2011. **7**: p. 536.

55. Han, C., et al., *PHLDA1 promotes microglia-mediated neuroinflammation via regulating K63-linked ubiquitination of TRAF6.* Brain Behav Immun, 2020. **88**: p. 640-653.

56. Lee, J.Y., et al., *Down syndrome candidate region-1 protein interacts with Tollip and positively modulates interleukin-1 receptor-mediated signaling.* Biochim Biophys Acta, 2009. **1790**(12): p. 1673-80.

57. Xia, P., et al., *Sphingosine kinase interacts with TRAF2 and dissects tumor necrosis factor-alpha signaling.* J Biol Chem, 2002. **277**(10): p. 7996-8003.

58. Park, E.S., et al., *Tumor necrosis factor (TNF) receptor-associated factor (TRAF)-interacting protein (TRIP) negatively regulates the TRAF2 ubiquitin-dependent pathway by suppressing the TRAF2-sphingosine 1-phosphate (S1P) interaction.* J Biol Chem, 2015. **290**(15): p. 9660-73.

59. Ryu, J., et al., *Sphingosine 1-phosphate as a regulator of osteoclast differentiation and osteoclast-osteoblast coupling.* Embo j, 2006. **25**(24): p. 5840-51.

60. Wong, B.R., et al., *TRANCE, a TNF family member, activates Akt/PKB through a signaling complex involving TRAF6 and c-Src.* Mol Cell, 1999. **4**(6): p. 1041-9.

61. Narita, T., et al., *CIN85 associates with TNF receptor 1 via Src and modulates TNF-alpha-induced apoptosis.* Exp Cell Res, 2005. **304**(1): p. 256-64.

62. Johnsen, I.B., et al., *The tyrosine kinase c-Src enhances RIG-I (retinoic acid-inducible gene I)-elicited antiviral signaling.* J Biol Chem, 2009. **284**(28): p. 19122-31.

63. Liu, A., et al., *TRAF6 protein couples Toll-like receptor 4 signaling to Src family kinase activation and opening of paracellular pathway in human lung microvascular endothelia.* J Biol Chem, 2012. **287**(20): p. 16132-45.

64. Zhang, L., et al., *TRAF4 promotes TGF-β receptor signaling and drives breast cancer metastasis.* Mol Cell, 2013. **51**(5): p. 559-72.

65. Sundar, R., et al., *TRAF6 promotes TGFβ-induced invasion and cell-cycle regulation via Lys63-linked polyubiquitination of Lys178 in TGFβ type I receptor.* Cell Cycle, 2015. **14**(4): p. 554-65.

66. Cao, Q., et al., *TNF receptor-associated factor 6 (TRAF6) mediates the angiotensin-induced non-canonical TGF-β pathway activation of c-kit(+) cardiac stem cells.* Am J Transl Res, 2015. **7**(11): p. 2233-43.

67. Roberts, J.Z., et al., *The SCF(Skp2) ubiquitin ligase complex modulates TRAIL-R2-induced apoptosis by regulating FLIP(L).* Cell Death Differ, 2020. **27**(9): p. 2726-2741.

68. Gonzalvez, F., et al., *TRAF2 Sets a threshold for extrinsic apoptosis by tagging caspase-8 with a ubiquitin shutoff timer.* Mol Cell, 2012. **48**(6): p. 888-99.

69. Wiley, S.R., et al., *A novel TNF receptor family member binds TWEAK and is implicated in angiogenesis.* Immunity, 2001. **15**(5): p. 837-46.

70. da Silva, S.D., et al., *TRAF2 Cooperates with Focal Adhesion Signaling to Regulate Cancer Cell Susceptibility to Anoikis.* Mol Cancer Ther, 2019. **18**(1): p. 139-146.

71. Werneburg, B.G., et al., *Molecular characterization of CD40 signaling intermediates.* J Biol Chem, 2001. **276**(46): p. 43334-42.

72. Lieberson, R., et al., *Tumor necrosis factor receptor-associated factor (TRAF)2 represses the T helper cell type 2 response through interaction with NFAT-interacting protein (NIP45).* J Exp Med, 2001. **194**(1): p. 89-98.

73. Li, X., et al., *CaMKII-mediated Beclin 1 phosphorylation regulates autophagy that promotes degradation of Id and neuroblastoma cell differentiation.* Nat Commun, 2017. **8**(1): p. 1159.

74. Arthur, J.F., et al., *TNF receptor-associated factor 4 (TRAF4) is a novel binding partner of glycoprotein Ib and glycoprotein VI in human platelets.* J Thromb Haemost, 2011. **9**(1): p. 163-72.

75. Stark, C., et al., *BioGRID: a general repository for interaction datasets.* Nucleic Acids Res, 2006. **34**(Database issue): p. D535-9.

76. Gillespie, M., et al., *The reactome pathway knowledgebase 2022.* Nucleic Acids Research, 2021. **50**(D1): p. D687-D692.
