# Supplementary material for: Role of inflammatory signaling pathways involving the CD40–CD40L–TRAF cascade in diabetes and hypertension—insights from animal and human studies
Source: Basic Res Cardiol. 2024 Mar 30;119(4):1–18. doi: 10.1007/s00395-024-01045-1 (PMC11319409; doi:10.1007/s00395-024-01045-1)
Supplement: Supplementary file 6 — Supplementary file6 (DOCX 20 KB) [file 395_2024_1045_MOESM6_ESM.docx]

| **Suppl. Table S7. Comparison of our proteomic data with our RNA-seq data.** | | | | | | |
| --- | --- | --- | --- | --- | --- | --- |
| **Name** | **ID** | **Gene description** | **OLINK-name** | **CHD + HT vs CHD - log fold Expr** |  | **CHD + HT + T2DM vs CHD - log fold Expr** |
| AngPT1 | ENSG00000154188 | angiopoietin 1] |  | no change |  | 1,127422549 |
| CA9 | ENSG00000107159 | carbonic anhydrase 9 | CAIX | -8,555989156 |  | -6,051236722 |
| CCL13 | ENSG00000181374 | C-C motif chemokine ligand 13 | MCP4 | -2,623928029 |  | no change |
| CCL2 | ENSG00000108691 | C-C motif chemokine ligand | MCP1 | no change |  | no change |
| CCl3 | ENSG00000277632 | C-C motif chemokine ligand 3 |  | no change |  | no change |
| CCl4 | ENSG00000275302 | C-C motif chemokine ligand 4 |  | no change |  | no change |
| CD244 | ENSG00000122223 | CD244 molecule |  | no change |  | no change |
| CD27 | ENSG00000139193 | CD27 molecule |  | no change |  | no change |
| CD40 | ENSG00000101017 | CD40 molecule |  | no change |  | no change |
| CD5 | ENSG00000110448 | CD5 molecule |  | 2,246290297 |  | no change |
| CD68 | ENSG00000129226 | CD68 molecule |  | no change |  | no change |
| CD70 | ENSG00000125726 | CD70 molecule |  | no change |  | no change |
| CD8A | ENSG00000153563 | CD8a molecule |  | 2,658169189 |  | no change |
| CXCL11 | ENSG00000169248 | C-X-C motif chemokine |  | no change |  | -2,906297174 |
| CXCL8 | ENSG00000169429 | C-X-C motif chemokine ligand 8 | IL8 | no change |  | 4,1058954 |
| CYBB | ENSG00000165168 | cytochrome b-245 beta chain |  | no change |  | no change |
| FASLG | ENSG00000117560 | Fas ligand |  | no change |  | no change |
| GAL | ENSG00000069482 | galanin and GMAP prepropeptide |  | no change |  | -3,29932335 |
| GZMA | ENSG00000145649 | granzyme A |  | no change |  | no change |
| ICOSLG | ENSG00000160223 | inducible T cell costimulator ligand |  | no change |  | no change |
| IL12A | ENSG00000168811 | interleukin 12A |  | no change |  | no change |
| IL12B | ENSG00000113302 | interleukin 12B |  | no change |  | no change |
| KLRD1 | ENSG00000134539 | killer cell lectin like receptor D1 |  | no change |  | no change |
| LAG3 | ENSG00000089692 | lymphocyte activating 3 |  | no change |  | -1,792308183 |
| LGALS9 | ENSG00000168961 | galectin 9 | GAL9 | no change |  | no change |
| MMP7 | ENSG00000137673 | matrix metallopeptidase 7 |  | no change |  | no change |
| NCR1 | ENSG00000189430 | natural cytotoxicity triggering receptor 1 |  | no change |  | 5,100065251 |
| PDCD1 | ENSG00000188389 | programmed cell death 1 |  | no change |  | no change |
| PGF | ENSG00000119630 | placental growth factor |  | no change |  | 1,045135477 |
| TEK | ENSG00000120156 | TEK receptor tyrosine kinase | TIE2 | no change |  | no change |
| TNF | ENSG00000232810 | tumor necrosis factor |  | no change |  | no change |
| TNFRSF21 | ENSG00000146072 | TNF receptor superfamily member 21 | DR6 | no change |  | -2,05771898 |
| TNFRSF4 | ENSG00000186827 | TNF receptor superfamily member 4 | Ox40 | no change |  | no change |
| TNFRSF9 | ENSG00000049249 | TNF receptor superfamily member 9 | CD137 | no change |  | no change |
